# Supplementary material for: Test accuracy of drug and antibody assays for predicting response to antitumour necrosis factor treatment in Crohn’s disease: a systematic review and meta-analysis
Source: BMJ Open. 2017 Jul 2;7(6):e014581. doi: 10.1136/bmjopen-2016-014581 (PMC5734585; doi:10.1136/bmjopen-2016-014581)
Supplement: Supplementary data 2 [file bmjopen-2016-014581supp002.pdf]

Supplement 1 Search strategy

Ovid MEDLINE(R) 1946 to October Week 2 2014, searched on 22/10/2014

1	adalimumab.mp.	3597
2	ADA.tw.	7105
3	infliximab.mp.	8842
4	IFX.tw.	326
5	((anti-TNF* or antiTNF* or TNF*) adj2 inhibitor*).mp.	2577
6	anti* tumo?r* necrosis* factor*.mp.	3007
7	Tumor Necrosis Factor-alpha/ and Antibodies, Monoclonal/	7682
8	anti* drug* antibod*.tw.	186
9	ADAb.tw.	19
10	1 or 2 or 3 or 4 or 5 or 6 or 7 or 8 or 9	24181
11	lisa* tracker*.mp.	1
12	(immundiagnostik* or immunodiagnostik* or immunediagnostik*).mp.	159
13	(proteomika* or promonitor*).mp.	13
14	exp Enzyme-Linked Immunosorbent Assay/	129174
15	enzyme* link* immunoassay*.mp.	2873
16	enzyme* link* immuno* assay*.mp.	158537
17	ELISA*.mp.	113426
18	11 or 12 or 13 or 14 or 15 or 16 or 17	205224
19	*Radioimmunoassay/	7091
20	(radioimmuno* or radio immuno* or radio-immuno*).mp.	101819
21	RIA.tw.	17353
22	reporter* gene* assay*.mp.	3663
23	RGA.tw.	336
24	semi* fluid* phase* enzyme* immuno*.mp.	0

25	EIA.tw.	8288
26	((homogenous* or homogeneous*) adj1 mobilit* shift* assay*).mp.	4
27	HMSA.tw.	62
28	(Biomonitor* or iLite).tw.	4102
29	(Matriks* Biotek* or Shikari*).mp.	2
30	(Prometheus* or Anser IFX or Anser ADA).mp.	258
31	19 or 20 or 21 or 22 or 23 or 24 or 25 or 26 or 27 or 28 or 29 or 30	124775
32	((monitor* or pharmacokinetic* or measur* or level* or concentration*) adj3 (adalimumab or ADA or infliximab or IFX or Anti-TNF* or Anti-Tumour Necrosis Factor*)).mp.	1087
33	Inflammatory Bowel Diseases/	14444
34	Crohn Disease/	31596
35	crohn*.tw.	32370
36	inflammator* bowel* disease*.tw.	26840
37	IBD.tw.	11936
38	33 or 34 or 35 or 36 or 37	58401
39	(((monitor* or pharmacokinetic* or measur* or level* or concentration*) adj3 (adalimumab or infliximab or Anti-TNF* or AntiTNF* or Anti-Tumour Necrosis Factor*)) and (correlat* or associat* or test performance)).mp.	218
40	10 and 18 and 38	93
41	10 and 31 and 38	19
42	32 and 38	157
43	39 or 40 or 41 or 42	367
44	Animals/ not Humans/	3983380
45	43 not 44	349

Ovid MEDLINE(R) In-Process & Other Non-Indexed Citations October 21, 2014, searched on 22/10/2014

1	adalimumab.mp.	469
2	ADA.tw.	426
3	infliximab.mp.	814
4	IFX.tw.	69
5	((anti-TNF* or antiTNF* or TNF*) adj2 inhibitor*).mp.	308
6	anti* tumo?r* necrosis* factor*.mp.	323
7	anti* drug* antibod*.tw.	39
8	ADAb.tw.	1
9	1 or 2 or 3 or 4 or 5 or 6 or 7 or 8	1824
10	lisa* tracker*.mp.	0
11	(immundiagnostik* or immunodiagnostik* or immunediagnostik*).mp.	2
12	(proteomika* or promonitor*).mp.	0
13	enzyme* link* immunoassay*.mp.	133
14	enzyme* link* immuno* assay*.mp.	3996
15	ELISA*.mp.	8044
16	10 or 11 or 12 or 13 or 14 or 15	10101
17	(radioimmuno* or radio immuno* or radio-immuno*).mp.	1176
18	RIA.tw.	386
19	reporter* gene* assay*.mp.	240
20	RGA.tw.	47
21	semi* fluid* phase* enzyme* immuno*.mp.	0
22	EIA.tw.	357
23	((homogenous* or homogeneous*) adj1 mobilit* shift* assay*).mp.	0
24	HMSA.tw.	5
25	(Biomonitor* or iLite).tw.	343

26	(Matriks* Biotek* or Shikari*).mp.	1
27	(Prometheus* or Anser IFX or Anser ADA).mp.	23
28	17 or 18 or 19 or 20 or 21 or 22 or 23 or 24 or 25 or 26 or 27	2386
29	((monitor* or pharmacokinetic* or measur* or level* or concentration*) adj3 (adalimumab or ADA or infliximab or IFX or Anti-TNF* or Anti-Tumour Necrosis Factor*)).mp.	112
30	crohn*.tw.	2478
31	inflammator* bowel* disease*.tw.	2627
32	IBD.tw.	1480
33	30 or 31 or 32	4400
34	(((monitor* or pharmacokinetic* or measur* or level* or concentration*) adj3 (adalimumab or infliximab or Anti-TNF* or AntiTNF* or Anti-Tumour Necrosis Factor*)) and (correlat* or associat* or test performance)).mp.	30
35	9 and 16 and 33	15
36	9 and 28 and 33	0
37	29 and 33	35
38	34 or 35 or 36 or 37	57

Embase Classic+Embase 1947 to 2014 Week 42, searched on 22/10/2014

1	adalimumab.tw.	7379
2	*adalimumab/	3997
3	ADA.tw.	10848
4	infliximab.tw.	13600
5	*infliximab/	8056
6	IFX.tw.	1722
7	((anti-TNF* or antiTNF* or TNF*) adj2 inhibitor*).tw.	4663
8	anti* tumo?r* necrosis* factor*.tw.	4171

9	*tumor necrosis factor alpha inhibitor/	1283
10	anti* drug* antibod*.tw.	469
11	ADAb.tw.	44
12	*drug antibody/	1528
13	1 or 2 or 3 or 4 or 5 or 6 or 7 or 8 or 9 or 10 or 11 or 12	35630
14	lisa* tracker*.tw.	11
15	(immundiagnostik* or immunodiagnostik* or immunediagnostik*).tw.	74
16	(proteomika* or promonitor*).tw.	27
17	*enzyme linked immunosorbent assay/	14622
18	enzyme* link* immunoassay*.tw.	3275
19	enzyme* link* immuno* assay*.tw.	71923
20	ELISA*.tw.	166866
21	14 or 15 or 16 or 17 or 18 or 19 or 20	207373
22	*radioimmunoassay/	17240
23	(radioimmuno* or radio immuno* or radio-immuno*).tw.	74895
24	RIA.tw.	20769
25	reporter* gene* assay*.tw.	4396
26	RGA.tw.	400
27	semi* fluid* phase* enzyme* immuno*.tw.	1
28	EIA.tw.	10836
29	((homogenous* or homogeneous*) adj1 mobilit* shift* assay*).tw.	39
30	HMSA.tw.	98
31	(Biomonitor* or iLite).tw.	5664
32	(Matriks* Biotek* or Shikari*).tw.	13
33	(Prometheus* or Anser IFX or Anser ADA).tw.	568
34	22 or 23 or 24 or 25 or 26 or 27 or 28 or 29 or 30 or 31 or 32 or 33	113752

35	((monitor* or pharmacokinetic* or measur* or level* or concentration*) adj3 (adalimumab or ADA or infliximab or IFX or Anti-TNF* or Anti-Tumour Necrosis Factor*)).tw.	2016
36	*crohn disease/	34280
37	crohn*.tw.	50039
38	inflammator* bowel* disease*.tw.	41418
39	IBD.tw.	23266
40	36 or 37 or 38 or 39	82551
41	(((monitor* or pharmacokinetic* or measur* or level* or concentration*) adj3 (adalimumab or infliximab or Anti-TNF* or AntiTNF* or Anti-Tumour Necrosis Factor*)) and (correlat* or associat* or test performance)).tw.	544
42	13 and 21 and 40	278
43	13 and 34 and 40	109
44	35 and 40	507
45	41 or 42 or 43 or 44	938
46	nonhuman/ not human/	3490973
47	45 not 46	917

Cochrane Library (Wiley), searched on 22/10/2014

#1	adalimumab:ti,ab,kw	451
#2	ADA:ti,ab	237
#3	infliximab:ti,ab,kw	767
#4	IFX:ti,ab	39
#5	((anti-TNF* or antiTNF* or TNF*) near/2 inhibitor*):ti,ab,kw	106
#6	(anti* next tumo*r* next necrosis* next factor*):ti,ab,kw	256
#7	MeSH descriptor: [Tumor Necrosis Factor-alpha] this term only	2408
#8	MeSH descriptor: [Antibodies, Monoclonal] this term only	3978
#9	#7 and #8	409

#10	(anti* next drug* next antibod*):ti,ab,kw	19
#11	(ADAb):ti,ab,kw	0
#12	#1 or #2 or #3 or #4 or #5 or #6 or #7 or #8 or #9 or #10 or #11	6714
#13	(lisa* next tracker*):ti,ab,kw	0
#14	(immundiagnostik* or immunodiagnostik* or immunediagnostik*):ti,ab,kw	0
#15	(proteomika* or promonitor*):ti,ab,kw	0
#16	MeSH descriptor: [Enzyme-Linked Immunosorbent Assay] explode all trees	2122
#17	(enzyme* next link* next immunoassay*):ti,ab,kw	84
#18	ELISA*:ti,ab,kw	2534
#19	#13 or #14 or #15 or #16 or #17 or #18	3958
#20	MeSH descriptor: [Radioimmunoassay] explode all trees	1176
#21	(radioimmuno* or radio next immuno* or radio-immuno*):ti,ab,kw	2761
#22	RIA:ti,ab	570
#23	(reporter* next gene* next assay*):ti,ab,kw	11
#24	RGA:ti,ab	8
#25	(semi* next fluid* next phase* next enzyme* next immuno*):ti,ab,kw	0
#26	EIA:ti,ab	339
#27	((homogenous* or homogeneous*) near/1 (mobilit* next shift* next assay*)):ti,ab,kw	1
#28	HMSA:ti,ab	1
#29	(Biomonitor* or iLite):ti,ab,kw	14
#30	(Matriks* next Biotek* or Shikari*):ti,ab,kw	0
#31	(Prometheus* or Anser next IFX or Anser next ADA):ti,ab,kw	23
#32	#20 or #21 or #22 or #23 or #24 or #25 or #26 or #27 or #28 or #29 or #30 or #31	3651
#33	((monitor* or pharmacokinetic* or measur* or level* or concentration*) near/3 (adalimumab or ADA or infliximab or IFX or Anti-TNF* or Anti-Tumour next Necrosis next Factor*)):ti,ab,kw	83

#34	MeSH descriptor: [Inflammatory Bowel Diseases] this term only	273
#35	MeSH descriptor: [Crohn Disease] this term only	997
#36	crohn*:ti,ab,kw	1512
#37	(inflammator* next bowel* next disease*):ti,ab,kw	798
#38	IBD:ti,ab	271
#39	#34 or #35 or #36 or #37 or #38	2037
#40	(((monitor* or pharmacokinetic* or measur* or level* or concentration*) near/3 (adalimumab or infliximab or Anti-TNF* or AntiTNF* or Anti-Tumour next Necrosis next Factor*)) and (correlat* or associat* or test next performance)):ti,ab,kw	33
#41	#12 and #19 and #39	8
#42	#12 and #32 and #39	1
#43	#33 and #39	18
#44	#40 or #41 or #42 or #43	49

All Results (49)

Cochrane Reviews (0)

All Review Protocol

Other Reviews (1)

Trials (47)

Methods Studies (0)

Technology Assessments (1)

Economic Evaluations (0)

Cochrane Groups (0)

Science Citation Index and Conference Proceedings – Science (Web of Science), searched on 22/10/2014

# 40	806	#39 OR #38 OR #37 OR #36<br>Indexes=SCI-EXPANDED, CPCI-S Timespan=All years
# 39	324	#35 AND #32<br>Indexes=SCI-EXPANDED, CPCI-S Timespan=All years
# 38	26	#35 AND #31 AND #9

		Indexes=SCI-EXPANDED, CPCI-S Timespan=All years
# 37	128	#35 AND #16 AND #9<br>Indexes=SCI-EXPANDED, CPCI-S Timespan=All years
# 36	539	TS=(((monitor* or pharmacokinetic* or measur* or level* or concentration*) near/3 (adalimumab or ADA or infliximab or IFX or Anti-TNF* or ("Anti-Tumour Necrosis" near/1 Factor*))) and (correlat* or associat* or "test performance"))<br>Indexes=SCI-EXPANDED, CPCI-S Timespan=All years
# 35	80,743	#34 OR #33<br>Indexes=SCI-EXPANDED, CPCI-S Timespan=All years
# 34	53,142	TS=(((inflammator* near/1 bowel*) near/1 disease*) or IBD)<br>Indexes=SCI-EXPANDED, CPCI-S Timespan=All years
# 33	50,398	TS=crohn*<br>Indexes=SCI-EXPANDED, CPCI-S Timespan=All years
# 32	1,366	TS=((monitor* or pharmacokinetic* or measur* or level* or concentration*) near/3 (adalimumab or ADA or infliximab or IFX or Anti-TNF* or ("Anti-Tumour Necrosis" near/1 Factor*)))<br>Indexes=SCI-EXPANDED, CPCI-S Timespan=All years
# 31	79,288	#30 OR #29 OR #28 OR #27 OR #26 OR #25 OR #24 OR #23 OR #22 OR #21 OR #20 OR #19 OR #18 OR #17<br>Indexes=SCI-EXPANDED, CPCI-S Timespan=All years
# 30	713	TS=(Prometheus* or "Anser IFX" or "Anser ADA")<br>Indexes=SCI-EXPANDED, CPCI-S Timespan=All years
# 29	10	TS=((Matriks* near/1 Biotek*) or Shikari*)<br>Indexes=SCI-EXPANDED, CPCI-S Timespan=All years
# 28	8,841	TS=(Biomonitor* or iLite)<br>Indexes=SCI-EXPANDED, CPCI-S Timespan=All years
# 27	107	TS=HMSA<br>Indexes=SCI-EXPANDED, CPCI-S Timespan=All years
# 26	11	TS=((homogenous* or homogeneous*) near/1 (mobilit* near/1 (shift* near/1 assay*)))<br>Indexes=SCI-EXPANDED, CPCI-S Timespan=All years
# 25	8,832	TS=EIA<br>Indexes=SCI-EXPANDED, CPCI-S Timespan=All years
# 24	1	TS=((semi* near/1 fluid*) near/3 (enzyme* near/1 immuno*))<br>Indexes=SCI-EXPANDED, CPCI-S Timespan=All years

# 23	0	TS=((semi* near/1 fluid*) near/2 (enzyme* near/1 immuno*))<br>Indexes=SCI-EXPANDED, CPCI-S Timespan=All years
# 22	0	TS=(semi* near/1 fluid* near/1 phase* near/1 enzyme* near/1 immuno*)<br>Indexes=SCI-EXPANDED, CPCI-S Timespan=All years
# 21	0	TS=(((semi* near/1 fluid*) near/1 phase*) near/1 (enzyme* near/1 immuno*))<br>Indexes=SCI-EXPANDED, CPCI-S Timespan=All years
# 20	1,230	TS=RGA<br>Indexes=SCI-EXPANDED, CPCI-S Timespan=All years
# 19	4,518	TS=(reporter* near/1 gene* near/1 assay*)<br>Indexes=SCI-EXPANDED, CPCI-S Timespan=All years
# 18	12,773	TS=RIA<br>Indexes=SCI-EXPANDED, CPCI-S Timespan=All years
# 17	46,937	TS=(radioimmuno* or (radio near/1 immuno*) or radio-immuno*)<br>Indexes=SCI-EXPANDED, CPCI-S Timespan=All years
# 16	146,389	#15 OR #14 OR #13 OR #12 OR #11 OR #10<br>Indexes=SCI-EXPANDED, CPCI-S Timespan=All years
# 15	113,120	TS=ELISA*<br>Indexes=SCI-EXPANDED, CPCI-S Timespan=All years
# 14	60,666	TS=((enzyme* near/1 link*) near/1 (immuno* near/1 assay))<br>Indexes=SCI-EXPANDED, CPCI-S Timespan=All years
# 13	2,850	TS=((enzyme* near/1 link*) near/1 immunoassay*)<br>Indexes=SCI-EXPANDED, CPCI-S Timespan=All years
# 12	1	TS=(proteomika* or promonitor*)<br>Indexes=SCI-EXPANDED, CPCI-S Timespan=All years
# 11	9	TS=(immundiagnostik* or immunodiagnostik* or immunediagnostik*)<br>Indexes=SCI-EXPANDED, CPCI-S Timespan=All years
# 10	0	TS=(lisa* near/1 tracker*)<br>Indexes=SCI-EXPANDED, CPCI-S Timespan=All years
# 9	32,262	#8 OR #7 OR #6 OR #5 OR #4 OR #3 OR #2 OR #1<br>Indexes=SCI-EXPANDED, CPCI-S Timespan=All years
# 8	35	TS=ADAb<br>Indexes=SCI-EXPANDED, CPCI-S Timespan=All years
# 7	2,534	TS=((anti* near/1 drug*) near/1 antibod*)<br>Indexes=SCI-EXPANDED, CPCI-S Timespan=All years

# 6	4,072	TS=((anti* near/1 tumo$r*) near/1 (necrosis* near/1 factor*))<br>Indexes=SCI-EXPANDED, CPCI-S Timespan=All years
# 5	4,065	TS=((anti-TNF* or antiTNF* or TNF*) near/2 inhibitor*)<br>Indexes=SCI-EXPANDED, CPCI-S Timespan=All years
# 4	373	TS=IFX<br>Indexes=SCI-EXPANDED, CPCI-S Timespan=All years
# 3	13,729	TS=infliximab<br>Indexes=SCI-EXPANDED, CPCI-S Timespan=All years
# 2	8,006	TS=ADA<br>Indexes=SCI-EXPANDED, CPCI-S Timespan=All years
# 1	4,973	TS=adalimumab<br>Indexes=SCI-EXPANDED, CPCI-S Timespan=All years

Index to Theses, searched on 28/10/2014

((adalimumab or infliximab or AntiTNF* or Anti-TNF* or "Anti TNF" or "Anti TNFa" or "Anti TNFalpha" or (TNF* w/2 inhibitor*) or (Anti-Tum*r w/2 Necrosis) or ("anti drug" w/2 antibod*) or ADAb) AND (crohn* or "inflammatory bowel disease" or IBD))

14 document(s) retrieved

(((adalimumab or infliximab or AntiTNF* or Anti-TNF* or "Anti TNF" or "Anti TNFa" or "Anti TNFalpha" or (TNF* w/2 inhibitor*) or (Anti-Tum*r w/2 Necrosis) or "anti drug antibody" or "anti drug antibodies" or "anti-drug antibody" or "anti-drug antibodies" or ADAb) w/10 (monitor or monitoring or monitors or monitored or pharmacokinetic or pharmacokinetics or measure or measures or measurement or measuring or level or levels or concentration or concentrations)) AND ((correlate* or correlation* or associate* or association* or "test performance")))

4 document(s) retrieved

DART-Europe, searched on 28/10/2014

(adalimumab or infliximab or AntiTNF* or Anti-TNF* or "Anti TNF" or "Anti TNFa" or "Anti TNFalpha" or (TNF* and inhibitor*) or (Anti-Tum*r and Necrosis) or ("anti drug" and antibod*) or ADAb) and (crohn* or "inflammatory bowel disease" or "inflammatory bowel diseases" or IBD)

113 document(s) retrieved

Dissertations and Theses, searched on 29/10/2014

all(((adalimumab or infliximab or AntiTNF* or Anti-TNF* or "Anti TNF" or "Anti TNFa" or "Anti TNFalpha" or (TNF* n/2 inhibitor*) or (Anti-Tum*r n/2 Necrosis) or ("anti drug" n/2 antibod*) or ADAb) AND (crohn* or "inflammatory bowel disease" or "inflammatory bowel diseases" or IBD)))

21

all(((adalimumab or infliximab or AntiTNF* or Anti-TNF* or "Anti TNF" or "Anti TNFa" or "Anti TNFalpha" or (TNF* n/2 inhibitor*) or (Anti-Tum*r n/2 Necrosis) or "anti drug antibody" or "anti drug antibodies" or "anti-drug antibody" or "anti-drug antibodies" or ADAb) n/10 (monitor or monitoring or monitors or monitored or pharmacokinetic or pharmacokinetics or measure or measures or measurement or measuring or level or levels or concentration or concentrations)) and (correlate* or correlation* or associate* or association* or "test performance"))

15

NIHR HTA Programme, searched on 29/10/2014

adalimumab

16

infliximab

23

TNF

17

PROSPERO, searched on 29/10/2014

adalimumab in All fields

OR

infliximab in All fields

OR

TNF* inhibitor* in All fields

OR

AntiTNF* in All fields

OR

Anti-TNF* in All fields

29 records

ClinicalTrials.gov, searched on 04/11/2014

Search Terms (any field): adalimumab OR infliximab OR (TNF AND (anti OR inhibitor OR blocker)) OR "anti drug antibody" OR "anti drug antibodies" OR ADAb

AND

Condition: crohn OR "inflammatory bowel disease" OR "inflammatory bowel diseases"

AND

Title: monitor OR pharmacokinetic OR measure OR measuring OR level OR concentration OR assay

14 studies

Current Controlled Trials, searched on 04/11/2014

(adalimumab OR infliximab OR TNF* OR AntiTNF* OR Anti-TNF* OR anti drug antibod* OR ADAb) AND (crohn* OR inflammatory bowel disease*) AND (monitor* OR pharmacokinetic* OR measure* OR measuring OR level* OR concentration* OR assay*)

30 studies

UKCRN Portfolio Database, searched on 04/11/2014

Specialty: Gastroenterology

Research Summary: adalimumab infliximab TNF AntiTNF Anti-TNF ADAb

'Any' selected (combines terms with Boolean OR)

4 studies

WHO ICTRP, searched on 10/11/2014

Advanced Search

In Title: adalimumab OR infliximab OR AntiTNF* OR Anti-TNF* OR TNF inhibitor* OR TNFα inhibitor* OR TNF alpha inhibitor* OR TNFalpha inhibitor* OR anti drug antibody OR anti drug antibodies OR ADAb

AND

In Condition: Crohn* OR inflammatory bowel disease*

AND

In Intervention: monitor* OR pharmacokinetic* OR measure* OR measuring OR level* OR concentration* OR assay*

39 trials found

Supplement 2 Update search for new studies

Supplement 2 Figure 1 summarises the search update undertaken to identify new studies. There were 140 citations to the systematic reviews of Nanda et al. 2013 and Paul et al. 2014.[6 7]. Amongst these there were three recent systematic reviews,[48-50] which in turn yielded a further six unique citations. Within the three recent systematic reviews there were 35 unique primary studies. We screened all citations to the systematic reviews and all studies included in the new systematic reviews. [48-50]

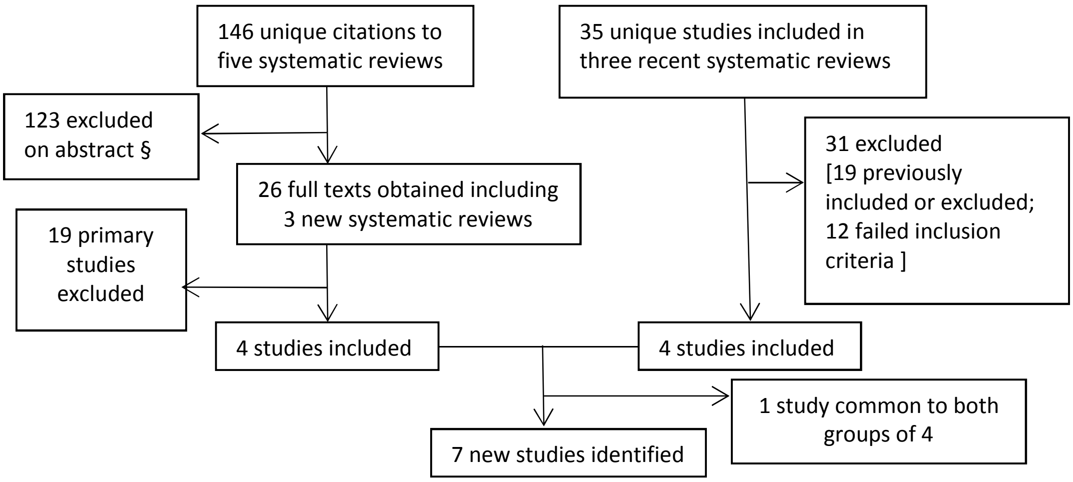

Supplement 2 Figure 1 Study flow diagram. *(Excluded studies are identified in Supplement 2 Table 1)*

Seven new studies satisfied our inclusion criteria, their main characteristics are summarised in Supplement 8 Table 1.

Supplement 2 Table 1 List of excluded studies with reasons for exclusion

Studies excluded from those included in three recent systematic reviews			Reason for exclusion
1a	Adedokuun 2014	Gastroenterology. 2014;147:1296–1307.e5.	all UC patients
2a	Ainsworth 2008	Am J Gastroenterol 2008;103(4):944-8	already included or excluded
3a	Baert 2014	Clin Gastroenterol Hepatol 2014;12(9):1474-81.e2	already included or excluded
4a	Ben-Basset 2013	Gastroenterology 2013;144(5 Suppl):S-775	already included or excluded
5a	Bortlik 2013	Journal of Crohn's & colitis 2013;7(9):736-43	already included or excluded
6a	Vande Casteele 2015	Gastroenterology 2015;148:1320–9.e3.	already included or excluded
7a	Vande Casteele 2014	Gut. 2015;64:1539–1545.	2x2 table not possible
8a	Vande Casteele 2013	Am J Gastroenterol.2013; 108:962–971.	already included or excluded
9a	Colombel 2014	Clin Gastroenterol Hepatol 12, 423	wrong drug

10a	Cornillie 2014	Gut 2011;60:A296.	already included or excluded
11a	Daperno 2013	Gastroenterology 2013;144:Tu1173.	too few CD patients
12a	Drastich 2011	Gastroenterology 2011;140:S292.	already included or excluded
13a	Drobne 2015	Clin Gastroenterol Hepatol 2015;13:514–21.e4.	2x2 table not possible
14a	Echarri 2015	J Crohns Colitis. 2015;9:S342–aS343.	2x2 table not possible
15a	Hibi 2014	J Gastroenterol 2014;49:254–62.	already included or excluded
16a	Imaeda 2014	J Gastroenterol.2014;49:100–109.	already included or excluded
17a	Imaeda 2014	J Gasroenterology 49;674-682	2x2 table not possible
18a	Marits 2014	J Crohns Colitis. 2014;8:881–889.	2x2 table not possible
19a	Maser 2006	Clin Gastroenterol Hepatol 2006;4(10):1248-54	already included or excluded
20a	Mazor 2014	Aliment Pharmacol Ther. 2014;40:620–628.	already included or excluded
21a	Murthy 2012	Gastroenterology 2012;142:S388.	all UC patients
22a	Papamichail 2015	Gastroenterology. 2015;148: S848.	all UC patients
23a	Pariente 2012	Inflamm Bowel Dis 2012;18:1199–206.	already included or excluded
24a	Paul 2013	Inflamm Bowel Dis 2013;19:2568–76.	too few CD patients
25a	Roblin 2014	Clin Gastroenterol Hepatol. 2014;12:80–84.e2.	already included or excluded
26a	Roblin 2015	Drug Levels & Biomarkers. 2015;148:S–853.	2x2 table not possible
27a	Ron 2012	Gastroenterology 2012;142:S385.	2x2 table not possible
28a	Seow 2010	Gut 2010;59:49–54	all UC patients
29a	Singh 2014	Inflamm Bowel Dis. 2014;20:1708–1713.	already included or excluded
30a	Steenholdt 2011	Scand J Gastroenterol 2011;46:310–8.	already included or excluded
31a	Tang 2014	J Crohns Colitis. 2014;8:S209–S210.	already included or excluded
**Studies excluded from citations to five systematic reviews**			**Reason for exclusion**
1	Vande Casteele 2013	American Journal of Gastroenterology 108(6): 962-971	See 8a
2	Bodini 2014	Digestive and Liver Disease 46(11): 1043-1046.	already included or excluded
3	Imaeda 2014	Journal of Gastroenterology 49(4): 674-682	See 17a
4	Marits 2014	Journal of Crohn's and Colitis 8(8): 881-889	See 18a
5	Pallagi-Kunstár 2014	World Journal of	already included or excluded

		Gastroenterology 20(17): 5031-5035	
6	Rivero Marcotegui 2014	Revista del Laboratorio Clinico 7(2): 68-72	already included or excluded
7	Roblin 2014	Clinical Gastroenterology and Hepatology 12(1): 80-84.e82	See 25a
8	Singh 2014	Inflammatory Bowel Diseases 20(10): 1708-1713	See 29a
9	Steenholdt 2014	American Journal of Gastroenterology 109(7): 1055-1064	already included or excluded
10	Steenholdt 2014	Gut 63(6): 919-927	already included or excluded
11	Ungar	Gut 63(8): 1258-1264	already included or excluded
12	Vaughn 2014	Inflammatory Bowel Diseases 20(11): 1996-2003	already included or excluded
13	Vande Casteele 2015	Gut 64(10): 1539-1545	2x2 table not possible
14	Roblin 2015	Journal of Crohn's and Colitis 9(7): 525-531	too few CD patients
15	Van Stappen 2015	Inflammatory Bowel Diseases 21(9): 2172-2177	2x2 table not possible
16	Warman 2015	European Journal of Gastroenterology and Hepatology 27(3): 242-248	too few CD patients
17	Yanai 2015	Clinical Gastroenterology and Hepatology 13(3): 522-530	2x2 table not possible
18	Yarur 2015	Clinical Gastroenterology and Hepatology 13(6): 1118-1124.e1113	too few CD patients
19	Bodini 2016	Scandinavian Journal of Gastroenterology 51(9): 1081-1086	2x2 table not possible

Supplement 3 Funnel plots and tests for publication bias

In the meta-analysis of tests for trough Infliximab levels using funnel plots and Harbord's and Peter's tests for small study bias in diagnostic odds ratios[1, 2] we found no evidence of small study bias in diagnostic odds ratios: Harbord test p = 0.312, Peters test p = 0.576. The corresponding values for tests of antibodies against Infliximab were p = 0.734 and p = 0.780.

Antibodies to Infliximab

1] Funnel plot

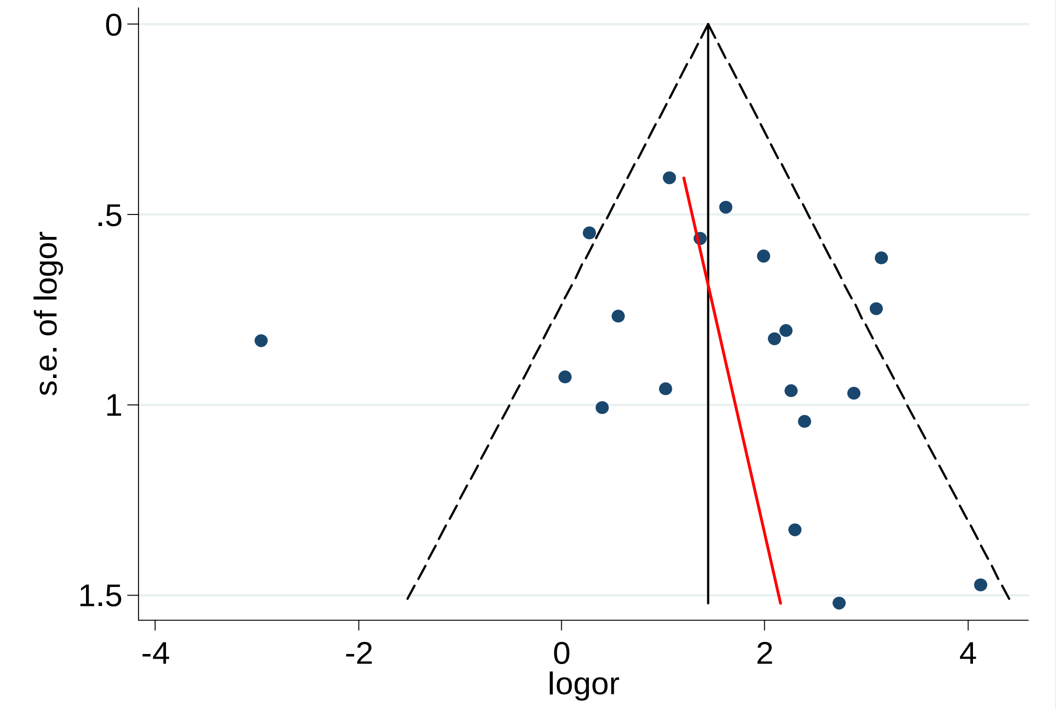

2] Egger's test for small-study effects:

Number of studies = 20

Eggers test

slope | .8614847 .8816692 0.98 0.341 -.9908337 2.713803

bias | .8517858 1.21317 0.70 0.492 -1.69699 3.400561

Test of H0: no small-study effects P = 0.492

Does not support publication bias.

1. Harbord R, Harris RJ, Sterne JAC. Updated tests for small-study effects in meta-analyses. Stata Journal 2009;9(2):197-210

2. Macaskill P, Gatsonis C, Deeks J, et al. Chapter 10: Analysing and Presenting Results. In: Deeks JJ, Bossuyt PM, Gatsonis C, eds. Cochrane Handbook for Systematic Reviews of Diagnostic Test Accuracy Version 10: The Cochrane Collaboration, 2010.

3] Harbord plot

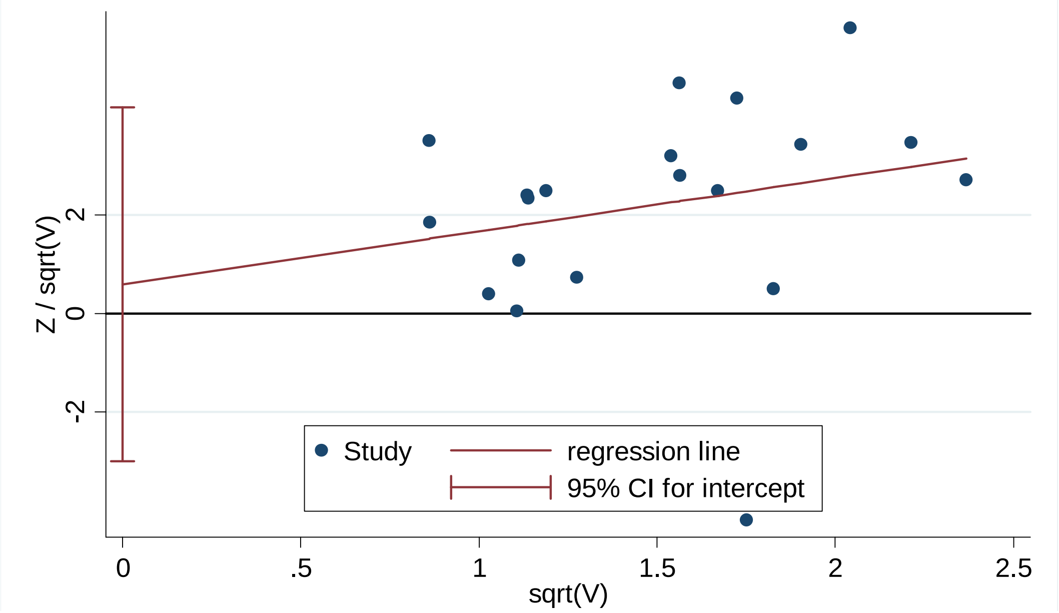

4] Harbord's modified test for small-study effects:
Number of studies = 20 Root MSE = 2.125

Z/sqrt(V)	Coef.	Std. Err.	t	P>|t|	[95% Conf. Interval]	
sqrt(V)	1.079732	1.099815	0.98	0.339	-1.230893	3.390356
bias	.5901862	1.710314	0.35	0.734	-3.003051	4.183424

Test of H0: no small-study effects P = 0.734

5] Peter's test for small-study effects:
Number of studies = 18 Root MSE = 1.459

Std_Eff	Coef.	Std. Err.	t	P>|t|	[95% Conf. Interval]	
bias	-8.626685	30.41227	-0.28	0.780	-73.09781	55.84444
constant	1.674552	.6008762	2.79	0.013	.400751	2.948352

Test of H0: no small-study effects P = 0.780

Trough Infliximab tests

1] Funnel plot

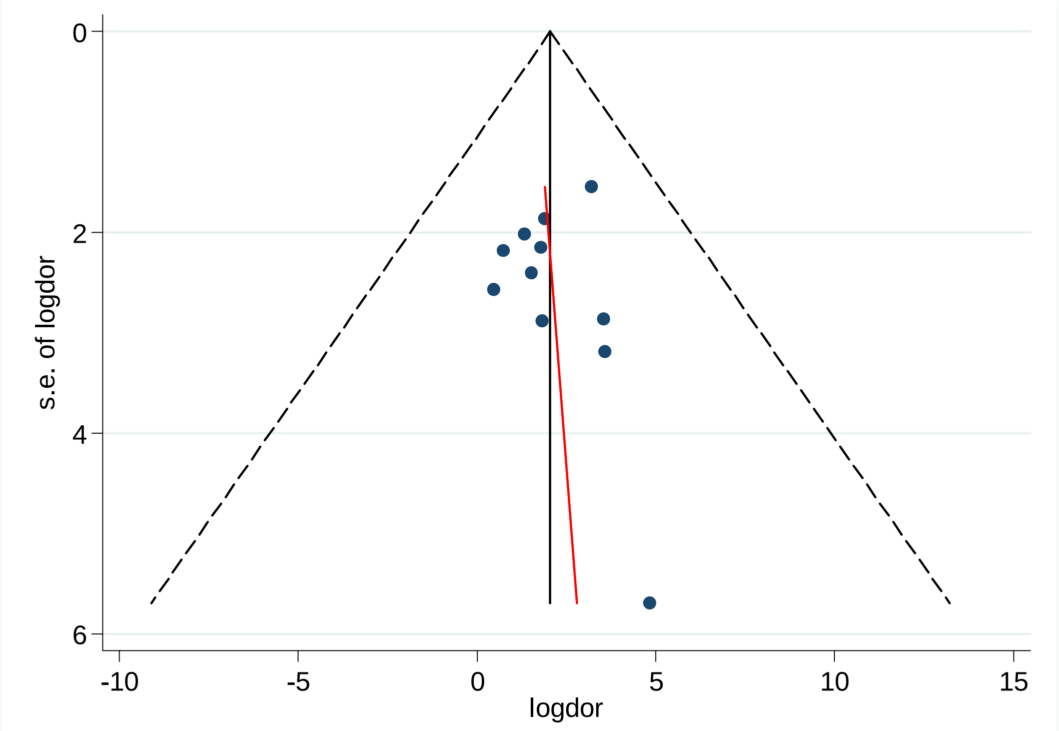

2] Egger's test for small-study effects:
Regress standard normal deviate of intervention
effect estimate against its standard error

Number of studies = 11 Root MSE = 1.907

Std_Eff	Coef.	Std. Err.	t	P>|t|	[95% Conf. Interval]	
slope	1.580826	1.251978	1.26	0.238	-1.251345	4.412998
bias	.8249369	2.088696	0.39	0.702	-3.900021	5.549894

Test of H0: no small-study effects P = 0.702

3] Harbord plot

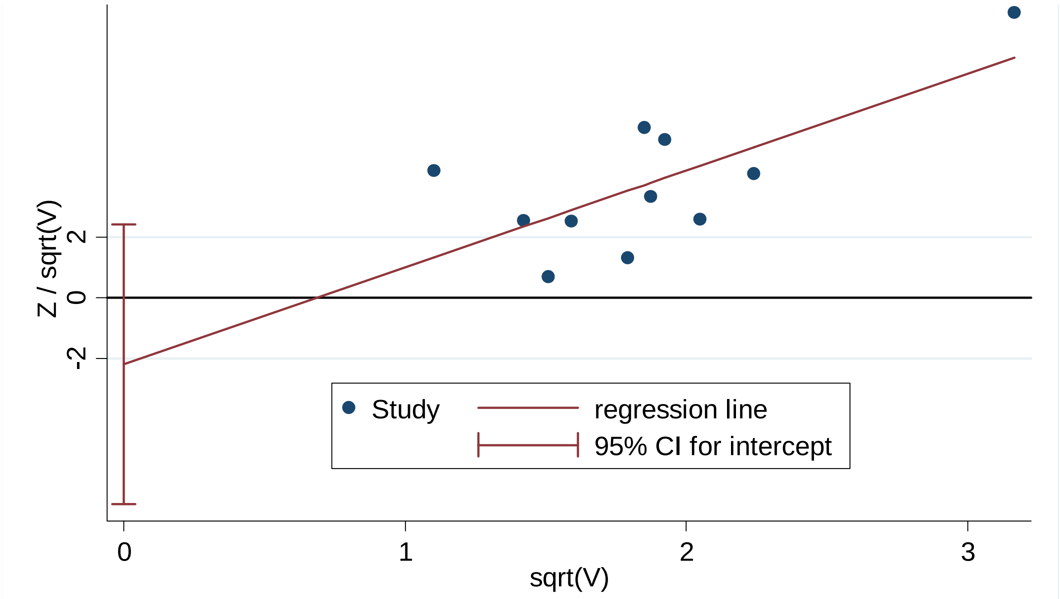

4] Harbord's modified test for small-study effects:
Regress Z/sqrt(V) on sqrt(V) where Z is efficient score and V is score variance
Number of studies = 11 Root MSE = 1.779
Test of H0: no small-study effects P = 0.312

5] Peter's test for small-study effects:
Regress intervention effect estimate on 1/Ntot, with weights S×F/Ntot
Number of studies = 11 Root MSE = 1.191

Std_Eff	Coef.	Std. Err.	t	P>|t|	[95% Conf. Interval]	
bias	-28.29877	48.81199	-0.58	0.576	-138.7192	82.12163
constant	2.738445	.725501	3.77	0.004	1.097248	4.379642

Test of H0: no small-study effects P = 0.576

Supplement 4 Excluded studies with reason

Supplement 4 Table 1 Full text exclusions with reason

Reference	Reason for exclusion
1. Afif, W., E. V. Loftus, Jr., W. A. Faubion, S. V. Kane, D. H. Bruining, K. A. Hanson and W. J. Sandborn (2010). "Clinical utility of measuring infliximab and human anti-chimeric antibody concentrations in patients with inflammatory bowel disease." American Journal of Gastroenterology 105(5): 1133-1139.	Insufficient data
2. Baert, F., M. Noman, S. Vermeire, G. Van Assche, D. H. G, A. Carbonez and P. Rutgeerts (2003). "Influence of immunogenicity on the long-term efficacy of infliximab in Crohn's disease." N Engl J Med 348(7): 601-608.	Insufficient data
3. Balzola, F., C. Bernstein, G. T. Ho and C. Lees (2010). "Clinical utility of measuring infliximab and human antichimeric antibody concentrations in patients with inflammatory bowel disease: Commentary." Inflammatory Bowel Disease Monitor 11(2): 85-86.	Commentary no original data
4. Balzola, F., G. Cullen, G. T. Ho and R. K. Russell (2013). "Clinical utility of newly developed immunoassays for serum concentrations of adalimumab and anti-adalimumab antibodies in patients with Crohn's disease." Inflammatory Bowel Disease Monitor 14(1): 19.	Commentary no original data
5. Ben-Horin, S. and Y. Chowers (2011). "Review article: loss of response to anti-TNF treatments in Crohn's disease." Aliment Pharmacol Ther 33(9): 987-995.	Review without MA
6. Billioud, V., W. J. Sandborn and L. Peyrin-Biroulet (2011). "Loss of response and need for adalimumab dose intensification in Crohn's disease: a systematic review." American Journal of Gastroenterology 106(4): 674-684.	SR without MA
7. Cassinotti A, Travis S. Incidence and clinicalsignificance of immunogenicity to infliximab inCrohn's disease: a critical systematic review. Inflamm Bowel Dis. 2009;15(8):1264-75.	Review without MA
8. Chaparro, M., I. Guerra, P. Munoz-Linares and J. P. Gisbert (2012). "Systematic review: antibodies and anti-TNF-alpha levels in inflammatory bowel disease." Aliment Pharmacol Ther 35(9): 971-986.	SR without MA
9. Colombel JF, Feagan BG, Sandborn WJ, Van Assche G, Robinson AM. Therapeutic drugmonitoring of biologics for inflammatory bowel disease. 2012;18(2):349-58.	Review without MA
10. Corstjens PL, Fidder HH, Wiesmeijer KC, et al. A rapid assay for on-site monitoring of infliximab trough levels: a feasibility study. Anal Bioanal Chem 2013;405(23):7367-75 doi: http://dx.doi.org/10.1007/s00216-013-7154-0[published Online First: Epub Date]|.	Insufficient data
11. Ebert, E. C., K. M. Das, V. Mehta and C. Rezac (2008). "Non-response to infliximab may be due to innate neutralizing anti-tumour necrosis factor-alpha antibodies." Clinical & Experimental Immunology 154(3): 325-331.	Measurement of antibodies to TNF-alpha not anti-TNFα drugs
12. Garces, S., J. Demengeot and E. Benito-Garcia (2013). "The immunogenicity of anti-TNF therapy in immune-mediated inflammatory diseases: a systematic review of the literature with a meta-analysis." Annals of the Rheumatic Diseases 72(12): 1947-1955.	>50% RA patients

13. Hamalainen, A., T. Sipponen and K. L. Kolho (2013). "Serum infliximab concentrations in pediatric inflammatory bowel disease." Scandinavian Journal of Gastroenterology 48(1): 35-41.	Insufficient data
14. Hibi, T., A. Sakuraba, M. Watanabe, S. Motoya, H. Ito, K. Motegi, Y. Kinouchi, M. Takazoe, Y. Suzuki, T. Matsumoto, K. Kawakami, T. Matsumoto, I. Hirata, S. Tanaka, T. Ashida and T. Matsui (2012). "Retrieval of serum infliximab level by shortening the maintenance infusion interval is correlated with clinical efficacy in Crohn's disease." Inflamm Bowel Dis 18(8): 1480-1487.	Insufficient data
15. Imaeda H, Bamba S, Takahashi K, et al. Relationship between serum infliximab trough levels and endoscopic activities in patients with Crohn's disease under scheduled maintenance treatment. J Gastroenterol 2014;49(4):674-82 doi: http://dx.doi.org/10.1007/s00535-013-0829-7[published Online First: Epub Date]|.	Insufficient data
16. Karmiris K, Paintaud G, Noman M, et al. Influence of trough serum levels and immunogenicity on long-term outcome of adalimumab therapy in Crohn's disease. Gastroenterology 2009;137(5):1628-40 doi: http://dx.doi.org/10.1053/j.gastro.2009.07.062[published Online First: Epub Date]|.	Insufficient data
17. Khanna, R., B. D. Sattin, W. Afif, E. I. Benchimol, E. J. Bernard, A. Bitton, B. Bressler, R. N. Fedorak, S. Ghosh, G. R. Greenberg, J. K. Marshall, R. Panaccione, E. G. Seidman, M. S. Silverberg, A. H. Steinhart, R. Sy, G. Van Assche, T. D. Walters, W. J. Sandborn and B. G. Feagan (2013). "Review article: a clinician's guide for therapeutic drug monitoring of infliximab in inflammatory bowel disease." Aliment Pharmacol Ther 38(5): 447-459.	SR without MA
18. Lazebnik, L. B. and V. E. Sagynbaeva (2013). "[Level of adalimumab and its antibody titers define the effectiveness of the biological (anticytokine) therapy in Crohn's disease]." Eksperimental'Naia i Klinicheskaia Gastroenterologiia(7): 18-22.	Non-English
19. Levesque BG, Greenberg GR, Zou G, et al. A prospective cohort study to determine the relationship between serum infliximab concentration and efficacy in patients with luminal Crohn's disease. Aliment Pharmacol Ther 2014;39(10):1126-35 doi: http://dx.doi.org/10.1111/apt.12733[published Online First: Epub Date]|.	Insufficient data
20. Lichtenstein, G. R. (2013). "Comprehensive review: antitumor necrosis factor agents in inflammatory bowel disease and factors implicated in treatment response." Therapeutic Advances in Gastroenterology 6(4): 269-293.	SR without MA
21. Malickova, K., D. Duricova, M. Bortlik, N. Machkova, I. Janatkova and M. Lukas (2011). "Serum infliximab trough levels and induction of antibodies to infliximab during the biological treatment of patients with inflammatory bowel diseases. [Czech]Serove hladiny infliximabu a indukce tvorby protilatek proti infliximabu pri biologicke lecbe nemocnych s idiopatickymi strevnimi zanety." Alergie 13(3): 216-222.	Non-English
22. Marits P, Landucci L, Sundin U, et al. Trough s-infliximab and antibodies towards infliximab in a cohort of 79 IBD patients with maintenance infliximab treatment. Journal of Crohn's & colitis 2014;8(8):881-9 doi: http://dx.doi.org/10.1016/j.crohns.2014.01.009[published Online First: Epub Date]|.	Insufficient data
23. Pallagi-Kunstar E, Farkas K, Szepes Z, et al. Utility of serum TNF-alpha, infliximab trough level, and antibody titers in inflammatory bowel disease. World J Gastroenterol 2014;20(17):5031-5 doi: http://dx.doi.org/10.3748/wjg.v20.i17.5031[published Online First: Epub Date]|.	Insufficient data
24. Paul S, Del Tedesco E, Marotte H, et al. Therapeutic drug monitoring of infliximab and mucosal healing in inflammatory bowel disease: a prospective study. Inflamm Bowel Dis 2013;19(12):2568-76 doi: http://dx.doi.org/10.1097/MIB.0b013e3182a77b41[published Online First: Epub Date]|.	Insufficient data
25. Rivero Marcotegui, A., R. Ibanez Bosch, A. Zuniga Vera, A. Arin Letamendia and M. J. Burusco Paternain (2014). "Clinical usefulness in measuring infliximab and human anti-chimeric antibodies. [Spanish]Utilidad clinica de la cuantificacion de infliximab y anticuerpos antiquimericos humanos." Revista del	patients >50% RA

Laboratorio Clinico 7(2): 68-72.	
26. Roblin, X., M. Rinaudo, E. Del Tedesco, J. M. Phelip, C. Genin, L. Peyrin-Biroulet and S. Paul (2014). "Development of an algorithm incorporating pharmacokinetics of adalimumab in inflammatory bowel diseases." American Journal of Gastroenterology 109(8): 1250-1256.	Insufficient data
27. Ruiz-Arguello B, del Agua AR, Torres N, et al. Comparison study of two commercially available methods for the determination of infliximab, adalimumab, etanercept and anti-drug antibody levels. Clin Chem Lab Med 2013;51(12):e287-9 doi: 10.1515/cclm-2013-0461[published Online First: Epub Date]|.	Insufficient data
28. Rutgeerts, P., G. D'Haens, S. Targan, E. Vasiliauskas, S. B. Hanauer, D. H. Present, L. Mayer, R. A. Van Hogezand, T. Braakman, K. L. DeWoody, T. F. Schaible and S. J. Van Deventer (1999). "Efficacy and safety of retreatment with anti-tumor necrosis factor antibody (infliximab) to maintain remission in Crohn's disease." Gastroenterology 117(4): 761-769.	Insufficient data
29. Schatz SB, Prell C, Freudenberg F, et al. PA-G-0035 Comparison of different tests for determination of infliximab levels and antibodies against infliximab in pediatric IBD patients. The 46th Annual Meeting of The European Society of Paediatric Gastroenterology, Hepatology and Nutrition. J Pediatr Gastroenterol Nutr 2013;56 suppl 2:19	Insufficient data
30. Singh N, Rosenthal CJ, Melmed GY, et al. Early infliximab trough levels are associated with persistent remission in pediatric patients with inflammatory bowel disease. Inflamm Bowel Dis 2014;20(10):1708-13 doi: http://dx.doi.org/10.1097/MIB.0000000000000137[published Online First: Epub Date]|.	Insufficient data
31. Sono, K., A. Yamada, Y. Yoshimatsu, N. Takada and Y. Suzuki (2012). "Factors associated with the loss of response to infliximab in patients with Crohn's disease." Cytokine 59(2): 410-416.	Insufficient data
32. Steenholdt C, Ainsworth MA, Tovey M, et al. Comparison of techniques for monitoring infliximab and antibodies against infliximab in Crohn's disease. Ther Drug Monit 2013;35(4):530-8 doi: http://dx.doi.org/10.1097/FTD.0b013e31828d23c3[published Online First: Epub Date]|.	Insufficient data
33. Steenholdt C, Bendtzen K, Brynskov J, et al. Clinical implications of measuring drug and anti-drug antibodies by different assays when optimizing infliximab treatment failure in Crohn's disease: post hoc analysis of a randomized controlled trial. Am J Gastroenterol 2014;109(7):1055-64 doi: http://dx.doi.org/10.1038/ajg.2014.106[published Online First: Epub Date]|.	Insufficient data
34. Steenholdt C BJ, Thomsen OØ, Munck LK, Fallingborg J, Christensen LA, Pedersen G, Kjeldsen J, Jacobsen BA, Oxholm AS, Kjellberg J, Bendtzen K, Ainsworth MA. Individualized therapy is a Long-Term Cost-Effective Method Compared to Dose Intensification in Crohn’s Disease Patients Failing Infliximab. Dig Dis Sci 2015; Published Online First on 12 Feb 2015. doi:10.1007/s10620-015-3581-4 doi: 10.1007/s10620-015-3581-4[published Online First: Epub Date]|.	Insufficient data
35. Steenholdt, C., M. Svenson, K. Bendtzen, O. O. Thomsen, J. Brynskov and M. A. Ainsworth (2011). "Severe infusion reactions to infliximab: aetiology, immunogenicity and risk factors in patients with inflammatory bowel disease." Aliment Pharmacol Ther 34(1): 51-58.	Insufficient data
36. Ungar, B., Y. Chowers, M. Yavzori, O. Picard, E. Fudim, O. Har-Noy, U. Kopylov, R. Eliakim, S. Ben-Horin and A. consortium (2014). "The temporal evolution of antidrug antibodies in patients with inflammatory bowel disease treated with infliximab." Gut 63(8): 1258-1264.	Insufficient data
37. Van Assche, G., C. Magdelaine-Beuzelin, G. D'Haens, F. Baert, M. Noman, S. Vermeire, D. Ternant, H. Watier, G. Paintaud and P. Rutgeerts (2008). "Withdrawal of immunosuppression in Crohn's disease treated with scheduled infliximab maintenance: a randomized trial." Gastroenterology 134(7): 1861-1868.	Insufficient data

38. Vande Casteele N, Buurman DJ, Sturkenboom MG, et al. Detection of infliximab levels and anti-infliximab antibodies: a comparison of three different assays. Aliment Pharmacol Ther 2012;36(8):765-71 doi: http://dx.doi.org/10.1111/apt.12030[published Online First: Epub Date]|.	Insufficient data
39. Vande Casteele N, Ferrante M, Van Assche G, et al. Trough Concentrations of Infliximab Guide Dosing for Patients with Inflammatory Bowel Disease. Gastroenterology Forthcoming 2015 doi: 10.1053/j.gastro.2015.02.031[published Online First: Epub Date]|.	Insufficient data
40. Vaughn BP, Martinez-Vazquez M, Patwardhan VR, et al. Proactive therapeutic concentration monitoring of infliximab may improve outcomes for patients with inflammatory bowel disease: results from a pilot observational study. Inflamm Bowel Dis 2014;20(11):1996-2003 doi: http://dx.doi.org/10.1097/MIB.0000000000000156[published Online First: Epub Date]|.	Insufficient data
41. Vermeire, S., M. Noman, G. Van Assche, F. Baert, G. D'Haens and P. Rutgeerts (2007). "Effectiveness of concomitant immunosuppressive therapy in suppressing the formation of antibodies to infliximab in Crohn's disease." Gut 56(9): 1226-1231.	Insufficient data
42. Wang SL, Ohrmund L, Hauenstein S, et al. Development and validation of a homogeneous mobility shift assay for the measurement of infliximab and antibodies-to-infliximab levels in patient serum. J Immunol Methods 2012;382(1-2):177-88 doi: http://dx.doi.org/10.1016/j.jim.2012.06.002[published Online First: Epub Date]|.	Insufficient data
43. Yamada, A., K. Sono, N. Hosoe, N. Takada and Y. Suzuki (2010). "Monitoring functional serum antitumor necrosis factor antibody level in Crohn's disease patients who maintained and those who lost response to anti-TNF." Inflamm Bowel Dis 16(11): 1898-1904.	Insufficient data
44. Yanai H, Hanauer SB. Assessing response and loss of response to biological therapies in IBD. Am JGastroenterol. 2011;106(4):685-98	Review without MA

Supplement 4 Table 2 Excluded abstracts with reason

**Reference**	**Reason for exclusion**
45. Abraham, B. and M. Chiorean (2012). "False positive infliximab levels detected in patients treated with adalimumab for inflammatory bowel disease." American Journal of Gastroenterology 107: S627	Insufficient data
46. Afif, W., E. V. Loftus, W. A. Faubion, K. A. Hanson and W. J. Sandborn (2009). "Clinical utility of measuring infliximab and human anti-chimeric antibody levels in patients with inflammatory bowel disease." Gastroenterology 1): A147.	Superseded by full text
47. Anonymous (2012). "New Assay Can Detect Infliximab Levels and Anti-Infliximab Antibodies From a Single Serum Sample." Clinical Advances in Hematology and Oncology 10 (10): 27.	Editorial no original data
48. Armbruster, S., M. Ally, C. Maydonovitch, J. Betteridge and G. Veerappan (2012). "The use of human anti-chimeric antibody (HACA) and infliximab levels in the management of inflammatory bowel disease." American Journal of Gastroenterology 107: S641.	Insufficient data
49. Arranz, M. D. M., E. M. Arranz, D. P. Salcedo, C. De Diego, S. G. Senent, J. P. Cordon, B. B. Garcia and J. M. S. Parga (2014). "Infliximab trough levels and antibodies: Relationship with infusion reaction, immunomodulators and biological parameters." Gastroenterology 1): S-243.	Insufficient data
50. Baert, F. J., D. Drobne, V. Ballet, I. Cleynen, G. Compernolle, P. J. Rutgeerts, G. A. Van Assche, A. Gils and S. Vermeire (2011). "Early trough	Insufficient data

levels and antibodies predict safety and success of restarting infliximab after long drug holiday." Gastroenterology 1): S62.	
51. Baert, F. J., S. Lockton, S. Hauenstein, S. Singh, A. Gils and S. Vermeire (2014). "Antibodies to adalimumab predict inflammation in crohn's patients on maintenance adalimumab therapy." Gastroenterology 1): S-242	Insufficient data
52. Ben-Bassat, O., S. Hauenstein, A. Iacono, S. P. Irwin, S. Singh and G. R. Greenberg (2013). "Serum adalimumab and immunogenicity in IBD patients after 80mg biweekly maintenance therapy." Gastroenterology 1): S771.	Insufficient data
53. Ben-Horin, S., B. Ungar, Y. Chowers, M. Yavzori, O. Picard, E. Fudim and R. Eliakim (2013). "The temporal evolution of anti-drug antibodies in IBD patients treated with infliximab." Journal of Gastroenterology and Hepatology 28: 145.	Insufficient data
54. Bodini, G., V. Savarino, P. Dulbecco, I. Baldissarro and E. Savarino (2014). "TNF-alpha levels strongly correlated with disease activity based on HBI and CDEIS in patients with crohn's disease in maintenance treatment with adalimumab." Gastroenterology 1): S-238.	Insufficient data
55. Bodini, G., V. Savarino, P. Dulbecco, I. Baldissarro and E. Savarino (2014). "The influence of anti-adalimumab antibodies on adalimumab trough levels, TNF-alpha levels and clinical outcome." Journal of Crohn's and Colitis 8: S42.	Insufficient data
56. Bodini, G., V. Savarino, P. Dulbecco, I. Baldissarro and E. V. Savarino (2014). "Elisa vs. HMSA: A comparison between two different methods for measuring adalimumab serum concentration and anti-adalimumab antibodies-preliminary data." Digestive and Liver Disease 46: S67.	Duplicate
57. Bodini, G., V. Savarino, P. Dulbecco, L. Assandri, L. Bruzzone, F. Mazza, V. Fazio, E. Giambruno, L. Gemignani and E. Savarino (2013). "Correlation between adalimumab trough serum concentration, anti-adalimumab antibodies and TNF-alpha levels with clinical outcome in patients affected by crohn's disease." Gastroenterology 1): S780.	Insufficient data
58. Bodini, G., V. Savarino, V. Fazio, L. Assandri, L. Gemignani, P. Dulbecco, E. Giambruno and E. Savarino (2012). "Relationship between drug serum concentration and clinical activity in patients with Crohn's Disease who achieved remission with adalimumab." Digestive and Liver Disease 44: S69-S70.	Duplicate
59. Bodini, G., V. Savarino, V. Fazio, L. Assandri, P. Dulbecco, L. Gemignani and E. Savarino (2012). "Relationship between drug serum concentration and clinical activity in patients with crohn disease who achieved remission with adalimumab-a prospective study." Gastroenterology 1): S388.	Insufficient data
60. Bortlik, M., D. Duricova, K. Malickova, A. Komarek, N. Machkova, E. Bouzkova, L. Hrdlicka and M. Lukas (2012). "Infliximab trough levels may predict sustained response to infliximab in patients with Crohn's disease: A single cohort study." Journal of Crohn's and Colitis 6: S153.	Superseded by full text
61. Cardile, S., A. Costa, I. Loddo, G. Morabito, C. Pidone and C. Romano (2013). "Impact of measurement of infliximab and anti-infliximab antibodies levels in pediatric inflammatory bowel disease." Digestive and Liver Disease 45: e294-e295.	Insufficient data
62. Chauhan, U., U. Dutta, D. Armstrong, E. Greenwald, J. Marshall, F. Tse, T. Xenodemetropoulos and H. Smita (2012). "Does measuring infliximab and human anti-chimeric antibody concentrations in patients with inflammatory bowel disease impact clinical management? A canadian experience." Inflamm Bowel Dis 18: S82-S83	Insufficient data
63. Chauhan, U., U. Dutta, D. Armstrong, J. Marshall, F. Tse, E. Greenwald, T. Xenodemetropoulos and S. Halder (2013). "Does measuring IFX and human anti-chimeric antibody concentrations in patients with inflammatory bowel disease impact clinical management? A Canadian experience." Journal of Crohn's and Colitis 7: S228.	Duplicate
64. Chollet-Martin, S., P. Nicaise-Roland, L. De Chaisemartin, S. Grootenboer-Mignot, G. Hayem, A. L. Pelletier, A. Amiot, V. Descamps, Y. Bouhnik and O. Meyer (2013). "Simultaneous determination of anti-infliximab antibodies	Insufficient data

and residual infliximab levels to monitor anti-TNF therapy." Annals of the Rheumatic Disease 71.	
65. Church, P., J. Guan, K. Frost, A. Muise, T. Walters and A. Griffiths (2013). "Infliximab treatment for paediatric Crohn's disease: Long-term ouCTomes at a single centre." Journal of Crohn's and Colitis 7: S198.	Insufficient data
66. Church, P., J. Guan, L. Salz, K. Frost, A. Muise, T. Walters and A. Griffiths (2012). "Long-term outcomes with infliximab treatment in children with crohn's disease at a single centre." Inflamm Bowel Dis 18: S72-S7	Insufficient data
67. Cornillie, F., S. Hanauer, R. Diamond, J. Wang, D. Zelinger, Z. Xu, S. Vermeire and P. Rutgeerts (2011). "Early serum infliximab trough level, Clinical disease activity and crp as markers of sustained benefit of infliximab treatment in crohn's disease: A post-HOC analysis of the accent1 trial." American Journal of Gastroenterology 106: S462-S463	Insufficient data
68. Corstjens, P. L., K. Wiesmeijer, G. J. Wolbink, J. Tanke, D. W. Hommes and H. Fidder (2011). "A rapid test for quantitative determination of infliximab trough levels in blood." Gastroenterology 1): S276-S277	Insufficient data
69. Daperno M, Frigerio F, Guiotto C, et al. Evaluation of the diagnostic performance of two commercially available tests for infliximab trough levels (IFX-TL) and antibodies to infliximab (ATI) titration in inflammatory bowel disease (IBD). Journal of Crohn's and Colitis 2013;7:S213-4	Insufficient data
70. Daperno, M., A. Lavagna, M. Fracchia, C. Guiotto, L. Germano, C. Rigazio, E. Ercole, M. Migliardi, R. Pellerito and R. Rocca (2013). "Infliximab trough levels (IFX-TL) are higher in patients with inflammatory bowel disease (IBD)treated with immunosuppressives: Clinical correlations of IFX-LT and antibodies to infliximab (ATI) in IBD." Gastroenterology 1): S781	Insufficient data
71. Daperno, M., A. Lavagna, M. Fracchia, C. Guiotto, L. Germano, C. Rigazio, E. Ercole, M. Migliardi, R. Pellerito and R. Rocca (2013). "Clinical correlations of infliximab trough levels (IFX-TL) and antibodies to infliximab (ATI) in inflammatory bowel disease." Journal of Crohn's and Colitis 7: S239.	Insufficient data
72. Daperno, M., F. Frigerio, C. Guiotto, G. Laura, E. Ercole, A. Lavagna, C. Rigazio, S. Arico, M. Migliardi, R. Pellerito and R. Rocca (2013). "Comparison of the performance of two commercially available tests for determination of infliximab trough levels (IFX-TL) and antibodies to infliximab (ATI), promonitor and immundiagnostik, in inflammatory bowel disease." Digestive and Liver Disease 45: S109.	Insufficient data
73. Daperno, M., F. Frigerio, C. Guiotto, L. Germano, E. Ercole, S. Arico, M. Fracchia, C. Rigazio, A. Lavagna, R. Pellerito, M. Migliardi and R. Rocca (2013). "Identical diagnostic performance of two commercially available tests for infliximab trough levels (IFX-TL) and antibodies to infliximab (ATI) titration in inflammatory bowel disease (IBD): Promonitor and immunodiagnostik tests." Gastroenterology 1): S780.	Duplicate
74. Daperno, M., M. Fracchia, C. Guiotto, L. Germano, E. Ercole, C. Rigazio, A. Lavagna, M. Migliardi, R. Pellerito and R. Rocca (2013). "Clinical implications and stability of determination of infliximab trough levels (IFX-TL) and antibodies to infliximab (ATI) in inflammatory bowel disease." Digestive and Liver Disease 45: S145.	Insufficient data
75. De Bruyn, M., T. Bessissow, T. Billiet, I. Cleynen, R. Kirkland, X. Liu, S. Hauenstein, K. Drake, S. Singh, M. Ferrante, P. Rutgeerts, G. Van Assche, I. Arijs, G. Opdenakker and S. Vermeire (2014). "Biomarker panel for prediction of mucosal healing in patients with Crohn's disease under infliximab therapy." Journal of Crohn's and Colitis 8: S45-S46.	Insufficient data
76. De Bruyn, M., T. Bessissow, T. Billiet, I. Cleynen, R. Kirkland, X. Liu, S. Hauenstein, K. Drake, S. Singh, M. Ferrante, P. J. Rutgeerts, G. A. Van Assche, I. Arijs, G. Opdenakker and S. Vermeire (2014).	Duplicate
77. Dotan, I., H. Yanai, Y. Ron, R. Kariv, S. Fishman, L. Yahav, M. Ben-Yehoyada, E. Santo and D. R. Mould (2014). "Population pharmacokinetic evaluation of adlimumab reveals patient factors that increase adalimumab clearance and shorten half-life in inflammatory bowel disease patients."	Insufficient data

Gastroenterology 1): S-243.	
78. Dotan, I., Y. Ron, H. Yanai, S. A. Becker, S. Fishman, L. Yahav, M. B. Yehoyada and D. R. Mould (2013). "Population pharmacokinetic evaluation of infliximab reveals patient factors that increase infliximab clearance and shorten half-life in inflammatory bowel disease patients." Gastroenterology 1): S774.	Insufficient data
79. Drastich, P., J. Kozeluhova, M. Jaresova and J. Spicak (2011). "Infliximab serum trough levels and deep remission in patients with IBD." Gastroenterology 1): S292	Insufficient data
80. Drobne, D., P. Bossuyt, C. Breynaert, N. Vande Casteele, G. Compernolle, M. Juergens, V. Ballet, I. Cleynen, W. Wollants, A. Gils, P. Rutgeerts, S. Vermeire and G. Van Assche (2011). "Long term evolution and impact of immunomodulator co-treatment and withdrawal on infliximab trough levels in 223 patients with Crohn's disease." Journal of Crohn's and Colitis 5 (1): S10-S11.	Insufficient data
81. Drobne, D., P. J. Bossuyt, C. Breynaert, N. V. Casteele, G. Compernolle, M. Jurgens, V. Ballet, W. J. Wollants, I. Cleynen, P. J. Rutgeerts, S. Vermeire, A. Gils and G. A. Van Assche (2011). "Crohn's disease: Infliximab trough levels and CRP during infliximab-immunomodulator combination treatment are associated with clinical ouCTome after immunomodulator withdrawal." Gastroenterology 1): S62.	Insufficient data
82. Duricova, D., K. Malickova, M. Bortlik, N. Machkova, V. Komarek, E. Bouzkova and M. Lukas (2011). "Predictors of sustained response to infliximab in patients with Crohn's disease: A single cohort study." Gastroenterology 1): S593.	Insufficient data
83. Echarri, A., R. Ferreiro, R. Fraga-Iriso, M. Barreiro-De Acosta, J. Cid, L. De-Castro, S. Pereira, A. Fernandez-Villaverde, S. Soto, D. Carpio, B. Gonzalez, E. Castro, V. Ollero and A. C. Campos (2014). "Drug trough levels and primary nonresponse to antitnf therapy in moderate-severe crohn disease. Results of the optimiza study." Gastroenterology 1): S-247.	Insufficient data
84. Egea-Pujol L, Reddy R, Patel S, et al. Homogenous mobility shift assay (HMSA) overcomes the limitations of elisa and eclia assays for monitoring infliximab (IFX), adalimumab (ADA), and associated anti-drug antibodies in serum. Am J Gastroenterol 2013;108:S548 doi: http://dx.doi.org/10.1038/ajg.2013.269[published Online First: Epub Date]|.	Insufficient data
85. Eser A, Primas C, Hauenstein S, et al. Comparison of early measurement of infliximab and antibodies-to-infliximab serum levels with standard trough analysis. Gastroenterology 2013;144(5 Suppl):S-779	Insufficient data
86. Eser A, Primas C, Haunstein S, et al. Detection of anti infliximab antibodies in patients with inflammatory bowel disease (IBD) in the presence of infliximab by homogeneous liquid phase anti infliximab mobility shift assay. Journal of Crohn's and Colitis 2013;7:S231-2	Insufficient data
87. Eser, A., C. Primas, R. Shringarpure, S. Hauenstein, S. L. Wang and W. Reinisch (2012). "Detection of anti infliximab antibodies in patients with inflammatory bowel disease (IBD) in the presence of infliximab by homogeneous liquid phase anti infliximab mobility shift assay." American Journal of Gastroenterology 107: S657	Duplicate
88. Fasanmade, A. A., C. Wagner, H. Davis, M. Graham, D. Everitt and A. Gottlieb (2002). "Comparison of the pharmacokinetics of infliximab in patients with psoriasis or Crohn's disease not receiving concomitant immunosuppressants or corticosteroids." Journal of Investigative Dermatology 119(1): 243-243.	Insufficient data
89. Fasanmade, A. A., P. Marsters, E. Munsanje, M. A. Graham, H. M. Davis and S. Van Deventer (2003). "Infliximab pharmacokinetics and improvement in fistulizing Crohn's Disease." Gastroenterology 124(4): A61-A61.	Insufficient data
90. Fasanmade, A. A., Y. W. Zhu, C. Wagner, C. Pendley and H. M. Davis (2002). "Population pharmacokinetics of single dose infliximab in patients with Crohn's disease." Clinical Pharmacology & Therapeutics 71(2): P66-P66.	Insufficient data

91. Fasanmade, A., A. Olson, W. Bao, C. Pendley, H. Davis and L. Mayer (2002). "Relationship between infliximab pharmacokinetics and improvement in Crohns disease." Gastroenterology 122(4): A617-A618.	Insufficient data
92. Feagan BG, Singh S, Lockton S, et al. Novel infliximab (IFX) and antibody-to-infliximab (ATI) assays are predictive of disease activity in patients with crohn's disease (CD). Gastroenterology 2012;142(5 Suppl):S-114	Insufficient data
93. Garces, S., J. Demengeot and E. Benito-Garcia (2012). "Clinical impact of immunogenicity of infliximab, adalimumab and etanercept: A systematic review of the literature with a meta-analysis." Annals of the Rheumatic Disease 71	Superseded by full paper
94. Garces, S., J. Demengeot, G. J. Wolbink, L. Aarden and E. Benito-Garcia (2011). "The immunogenicity of infliximab, adalimumab and etanercept in rheumatoid arthritis, ankylosing spondylitis, psoriatic arthritis, crohn's disease and ulcerative colitis a quantitative and a qualitative review." Arthritis and Rheumatism 1).	Superseded by full paper
95. Garces, S., J. Demengeot, J. C. Da Silva and L. Aarden (2011). "Bridging elisa as a screening assay to monitor immunogenicity in routine clinical practice." Arthritis and Rheumatism 1).	<50% CD
96. Garces, S., J. Demengeot, J. Canas-da-Silva and L. Aarden (2013). "Bridging ELISA as a secreening assay to monitor immunogenicity in routine clinical practice." Annals of the Rheumatic Disease 71.	Duplicate
97. Garces, S., J. Freitas, J. Canas-Silva, L. Aarden and J. Demengeot (2013). "The impact of immunogenicity on drug safety profile." Annals of the Rheumatic Diseases 72.	Insufficient data
98. Garimella, T. S., J. Z. Peng, K. Beck, P. A. Noertersheuser, K. G. Lomax, S. K. Paulson and P. F. Pollack (2006). "Pharmacokinetics of adalimumab in a long-term investigation of the induction and maintenance of remission in patients with Crohn's disease (CLASSIC I and CLASSIC II)." Gastroenterology 130(4): A481-A481.	Insufficient data
99. Goldberg R, Beswick L, Van Langenberg D, et al. Predictors of sub-therapeutic infliximab or adalimumab trough levels and anti-drug antibodies and their influence on therapeutic decisions. Journal of Crohn's and Colitis 2014;8:S223	Insufficient data
100. Greathead L, Kelleher P, Steel A. Development and validation of ELISA to measure serum anti TNFa levels. Journal of Crohn's and Colitis 2014;8:S97-8	Insufficient data
101. Guan, J., L. Salz, K. Frost, A. Muise, T. Walters and A. Griffiths (2012). "Long-term outcomes with infliximab treatment in children with crohn's disease at a single centre peter church." Inflamm Bowel Dis 18: S5-S6.	Duplicate
102. Guilday, C., D. Eastwood, Y. Zadvornova, D. Stein, A. S. Naik, K. Best, S. Skaros and L. P. Perera (2013). "Concomitant use of immunomodulator therapy results in higher serum infliximab levels compared to monotherapy without lowering serum haca levels." Gastroenterology 1): S429.	Insufficient data
103. Guiotto, C., L. Germano, M. Vizzini, R. Cerruti, F. Frigerio, M. Daperno, R. Rocca and M. Migliardi (2013). "Determination of infliximab trough levels (IFX-TL) and antibodies to Infliximab (ATI) in inflammatory bowel disease." Biochimica Clinica 37: S475.	Superseded
104. Hadigan, C. B. R., C. P. Braegger, E. Vasilauskis, J. C. Escher, M. Sinaasappel, G. D. Ferry, B. Kirschner, C. Wagner, R. Livingston, K. DeWoody and H. S. Winter (1999) Pharmacokinetics of infliximab (Anti-TNFx) in children with Crohn's disease: A multicenter trial. Journal of Pediatric Gastroenterology and Nutrition 29, 525	Insufficient data
105. Hauenstein S, Ohrmund L, Salbato J, et al. Comparison of homogeneous mobility shift assay and solid phase elisa for the measurement of drug and anti-drug antibody (ADA) levels in serum from patients treated with anti-TNF biologics. Gastroenterology 2012;142(5 Suppl):S-538	Insufficient data
106. Hauenstein, S., J. Salbato, S. Lockton and S. Singh (2013). "Characterization of neutralizing anti-drug antibody response in patients with loss of response to anti-TNF therapy." Gastroenterology 1): S418	Insufficient data

107. Hayes and Inc (2013) Use of anti-infliximab antibody levels to monitor infliximab treatment in patients with inflammatory bowel disease (IBD) (Structured abstract). Health Technology Assessment Database	Not available
108. Hester, K. D., X. Liu, J. Salbato, S. Lockton, S. Hauenstein and S. Singh (2014). "Improved homogeneous mobility shift assay (HMSA) for the detection of neutralizing antibodies (NAB) in ibd patients treated with infliximab or adalimumab." Gastroenterology 1): S-248	Insufficient data
109. Hibi, T., A. Sakuraba, M. Watanabe, S. Motoya, H. Ito, N. Sato, T. Yoshinari, K. Motegi, K. Yoshitaka, M. Takazoe, Y. Suzuki, T. Matsumoto, K. Kawakami, I. Hirata, S. Tanaka, T. Ashida and T. Matsui (2012). "Decrease in serum infliximab level precedes loss of clinical response and can be easily detected by the elevation of C-reactive protein in crohn's disease." Gastroenterology 1): S388.	Insufficient data
110. Hoekman, D. R., J. F. Brandse, T. De Meij, T. Hummel, M. Lowenberg, M. A. Benninga, G. R. D'Haens and A. Kindermann (2014). "Large variation in infliximab trough levels is associated with disease activity in paediatric inflammatory bowel disease." Gastroenterology 1): S-782.	Insufficient data
111. Hoekman, D., H. Brandse, T. De Meij, T. Hummel, M. Lowenberg, M. Benninga, G. D'Haens and A. Kindermann (2014). "Large variation in infliximab trough levels is associated with disease activity in paediatric inflammatory bowel disease." Journal of Crohn's and Colitis 8: S35.	Insufficient data
112. Huang, V. W., C. Prosser, C. Shalapay, D. K. Fedorak, N. Dhami, H. Wang, K. I. Kroeker and R. N. Fedorak (2014). "In IBD outpatients knowledge of fecal calprotectin and infliximab trough levels significantly enhances infliximab dose escalation decision making." Journal of Crohn's and Colitis 8: S255.	Insufficient data
113. Huang, V. W., N. Dhami, D. K. Fedorak, C. Prosser, C. Shalapay, K. I. Kroeker and R. N. Fedorak (2014). "Disparity between infliximab trough level and infliximab associated adverse events." Journal of Crohn's and Colitis 8: S282	Insufficient data
114. Huang, V., K. I. Kroeker, H. Wang, C. Prosser, S. Carol, N. Dhami, D. K. Fedorak and R. N. Fedorak (2014). "In IBD outpatients knowledge of fecal calprotectin and infliximab trough levels significantly alters clinical decision making." Gastroenterology 1): S241-S242.	Duplicate
115. Huang, V., N. Dhami, D. K. Fedorak, C. Prosser, S. Carol, K. I. Kroeker and R. N. Fedorak (2014). "Infliximab trough levels are correlated with infliximab-associated adverse events." Gastroenterology 1): S-1.	Insufficient data
116. Imaeda, H., A. Andoh, H. Ban, S. Bamba, M. Sasaki, T. Tsujikawa and Y. Fujiyama (2012). "The new immunoassay for the accurate determination of antibodies to infliximab, and relationship between its serum level and inflammatory values in crohn's disease." Gastroenterology 1): S349.	Superseded by full text
117. Imaeda, H., A. Andoh, K. Takahashi, T. Fujimoto, H. Ban, S. Bamba and Y. Fujiyama (2012). "Serum infliximab trough levels above 1.0 mg/ml are required to obtain clinical efficacy in patients with crohn's disease." Inflamm Bowel Dis 18: S59-S60.	Insufficient data
118. Imaeda, H., A. Andoh, S. Bamba, T. Tsujikawa and Y. Fujiyama (2011). "Development of a new Immunoassay for the accurate determinationof anti-infliximab antibodies in Crohn's Disease." Inflamm Bowel Dis 17: S42.	Duplicate
119. Imaeda, H., K. Takahashi, T. Fujimoto, S. Bamba, M. Sasaki, T. Tsujikawa, Y. Fujiyama and A. Andoh (2013). "Accurate determination of serum adalimumab and anti-adalimumab antibodies levels during maintenance therapy for crohn's disease." Gastroenterology 1): S431.	Insufficient data
120. Irving, P. M., Z. Arkir, J. Duncan, M. Sastrillo, S. Anderson and J. Sanderson (2012). "Initial experience with infliximab levels in a tertiary IBD centre." Gut 61: A238.	Insufficient data
121. Jauregui-Amezaga, A., I. Ordas, M. Gallego, A. Ramirez, S. Pino, M. C. Masamunt, M. Juan, E. Ricart, J. Yague and J. Panes (2013). "Impact of serum drug level and human anti-drug antibody measurement on management of biologic drugs in inflammatory bowel disease." Journal of	Insufficient data

Crohn's and Colitis 7: S202-S203.	
122. Juan, G., A. Alvarino, L. Oltra, N. Maroto, N. Cano, I. Ferrer and J. Hinojosa (2014). "Utility of "trough levels" determination and anti-infliximab antibodies in patients with inflammatory bowel disease. Estimation of individual pharmacokinetic parameters (PK) through population pharmacokinetic model." Journal of Crohn's and Colitis 8: S190	Insufficient data
123. Karmiris, K., G. Paintaud, D. Degenne, M. Ferrante, A. C. Duveau, M. Noman, G. A. Van Assche, S. Vermeire and P. Rutgeerts (2008). "Adalimumab trough serum levels and clinical response in a single-center cohort of inflammatory bowel disease patients: Can trough serum levels serve as a predictor for future loss of response?" Gastroenterology 134(4): A68-A68.	Insufficient data
124. Karmiris, K., M. Noman, G. Paintaud, M. Ferrante, A. C. Duveau, D. Degenne, G. A. Van Assche, S. Vermeire and P. Rutgeerts (2008). "A 3-week course of 80 mg weekly administered adalimumab as a rescue therapy for patients with Crohn's disease who lost response to 40 mg weekly: Relationship with adalimumab trough serum levels." Gastroenterology 134(4): A640-A640.	Insufficient data
125. Karsan, S. S., E. R. Cohen, S. R. Targan, A. Ippoliti, D. Q. Shih, E. A. Vasiliauskas, M. Dubinsky, D. Berel and D. P. McGovern (2012). "Analysis of clinical and serological associations, and the clinical consequences of the development of human anti-chimeric antibodies (HACAS), and low serum infliximab (IFX) levels in inflammatory bowel disease (IBD)." Gastroenterology 1): S264	Insufficient data
126. Kerr, J., A. Nair and R. Hinds (2013). "Variable practice in children with inflammatory bowel disease requiring infliximab infusions across Australia." Journal of Gastroenterology and Hepatology 28: 141.	Insufficient data
127. Kong, J. Y., C. Bundell, J. Pawlik, P. Hollingsworth and G. Forbes (2013). "Low Trough serum infliximab and antibodies to infliximab in smokers." Inflamm Bowel Dis 19(3): E35-E36.	Insufficient data
128. Kong, J. Y., C. S. Bundell, J. Pawlik, P. N. Hollingsworth and G. M. Forbes (2011). "Smoking is associated with low trough serum infliximab levels and presence of anti-infliximab antibody in maintenance treatment of inflammatory bowel disease (IBD)." Journal of Gastroenterology and Hepatology 26: 59	Insufficient data
129. Lamblin, C., A. Aubourg, D. Ternant, L. Picon, T. Lecomte and G. Paintaud (2012). "Concentration effect relationship of infliximab in Crohn's disease: Results of a cohort study." Journal of Crohn's and Colitis 6: S142-S143.	Insufficient data
130. Leclerc, M., H. Marotte, S. Paul, E. Del Tedesco, P. Gonzalo, J. M. Phelip, L. Peyrin Biroulet and X. Roblin (2014). "Persistence of antibodies to infliximab for more than two months strongly predicts loss of response to infliximab in inflammatory bowel diseases." Journal of Crohn's and Colitis 8: S226-S227.	Insufficient data
131. Li, J. L., S. K. Paulson, Y. L. Chiu, A. Robinson, K. G. Lomax and P. F. Pollack (2010). "Evaluation of potential correlations between serum adalimumab concentration and remission in patients with Crohn's disease in classic I and II." Gastroenterology 1): S101.	Insufficient data
132. Lowenberg, M., J. Brandse, L. Vos, C. Ponsioen, G. Van Den Brink and G. D'Haens (2014). "High infliximab trough levels are associated with impaired quality of life in IBD patients in clinical and biochemical remission on maintenance IFX therapy." Journal of Crohn's and Colitis 8: S262-S263	Insufficient data
133. Lowenberg, M., J. F. Brandse, L. M. Vos, C. Ponsioen, G. R. Van Den Brink and G. R. D'Haens (2014). "High infliximab trough levels are associated with impaired quality of life in IBD patients in clinical and biochemical remission on maintenance IFX therapy." Gastroenterology 1):	Insufficient data

S-450.	
134. Lukas, M., K. Malickova, M. Bortlik and D. Duricova (2009). "Anti-infliximab antibodies in routine clinical practice - Is it worth to assess them?" Gastroenterology 1): A679.	Insufficient data
135. Malickova, K., I. Janatkova, D. Duricova, M. Bortlik and M. Lukas (2011). "Serum infliximab levels, antibodies to infliximab and albumin concentrations during infliximab treatment in patients with inflammatory bowel disease." Clinical and Experimental Rheumatology 29(1): 213-213.	Insufficient data
136. Martin Arranz, M. D., E. Martin Arranz, D. Pascual-Salcedo, C. De Diego, M. Jaquotot, S. Gomez Senent, J. Poza and J. M. Suarez Parga (2014). "Infliximab trough levels and antibodies: Relationship with infusion reaction, immunomodulators and biological parameters." Journal of Crohn's and Colitis 8: S251	Insufficient data
137. Mazor Y, Kopylov U, Hur DB, et al. Evaluating adalimumab drug and antibody levels as predictors of clinical and laboratory response in crohn's disease patients. Gastroenterology 2013;144(5 Suppl):S-778	Insufficient data
138. Mazor, Y., U. Koplov, D. Ben Hur, R. Almog, M. Waterman, S. Ben-Horin and Y. Chowers (2013). "Evaluating Adalimumab drug and antibody levels as predictors of clinical and laboratory response in Crohn's disease patients." Journal of Crohn's and Colitis 7: S217.	Insufficient data
139. McTigue M, Sandborn W, Levesque B, et al. Clinical utility of next generation infliximab and antibodies to infliximab assay. Am J Gastroenterol 2013;108:S527 doi: http://dx.doi.org/10.1038/ajg.2013.269[published Online First: Epub Date]|.	Insufficient data
140. McTigue, M., W. Sandborn, B. Levesque and D. Patel (2013). "Infliximab therapeutic drug monitoring in clinical practice: Indications and utility." American Journal of Gastroenterology 108: S512.	Insufficient data
141. Morgenstern, J., E. Baestlein, L. Leifeld, P. Nguyen, J. Stein and W. Kruis (2012). "Infliximab drug levels in Crohn's disease responding to the treatment." Journal of Crohn's and Colitis 6: S125	Insufficient data
142. Noman, M., F. Baert, S. Vermeire, G. Van Assche, G. D'Haens, A. Carbonez and P. Rutgeerts (2002). "Post infusion infliximab levels determine duration of response in Crohn's disease and are directly related to infusion reactions." Gastroenterology 122(4): A100-A100.	Insufficient data
143. O'Donnell, S., J. M. Stempak and M. S. Silverberg (2014). "Is there a higher rate of infliximab dose optimization in initial responders between UC and CD cases?" Gastroenterology 1): S462-S463.	Insufficient data
144. Papamichail, K., N. V. Casteele, S. Hauenstein, F. Princen, S. Singh, M. Ferrante, G. A. Van Assche, P. J. Rutgeerts, A. Gils and S. Vermeire (2014). "Prediction of sustained remission after discontinuation of infliximab in patients with crohn's disease." Gastroenterology 1): S-457.	Insufficient data
145. Pariente, B., G. P. De Chambrun, M. Desroches, C. De Cassan, J. M. Gornet, P. Desreumaux, R. Krzysiek, D. Emilie, J. F. Colombel and M. Allez (2011). "Clinical value of measuring trough levels and human anti-chimeric antibodies in patients with inflammatory bowel disease who lost response to infliximab therapy." Gastroenterology 1): S277.	Superseded by full text
146. Pariente, B., G. Pineton De Chambrun, M. Desroches, C. De Cassan, J. Gornet, P. Desreumaux, R. Krzysiek, D. Emilie, J. Colombel and M. Allez (2011). "Clinical value of measuring trough levels and human anti-chimeric antibodies in patients with inflammatory bowel disease who lost response to infliximab therapy." Journal of Crohn's and Colitis 5 (1): S111-S112.	Duplicate
147. Paul S, Tedesco ED, Marotte H, et al. Interest of the dosage of serum concentration of infliximab and antibodies anti infliximab in the therapeutic response under infliximab in IBD. Gastroenterology 2012;142(5 Suppl):S354	Insufficient data

148. Paul, S., E. Del Tedesco, H. Marotte, L. Clavel, J. M. Phelip, L. Peyrin-Biroulet and X. Roblin (2013). "Therapeutic drug monitoring of infliximab and mucosal healing in inflammatory bowel disease: A prospective study." Gastroenterology 1): S92.	Superseded by full text
149. Paul, S., E. Del Tedesco, H. Marotte, M. Rinaudo-Gaujous, J. M. Phelip, L. Peyrin-Biroulet and X. Roblin (2013). "Infliximab concentration is associated with mucosal healing in intestinal bowel disease (IBD)." Journal of Crohn's and Colitis 7: S199-S200.	Insufficient data
150. Paulson, S. K., P. Nocrtersheuser, P. F. Pollack and R. S. Hoffman (2005). "Pharmacokinetics of adalimumab from classic, a randomized phase 3 trial for the induction of clinical remission in patients with Crohn's." Gastroenterology 128(4): A585-A585.	Insufficient data
151. Pradhan, R. S., S. Sharma, R. Thakkar, A. Robinson, J. S. Hyams, J. R. Rosh, F. Ruemmele and W. M. Awni (2013). "Relationship between adalimumab concentration and efficacy for the induction of clinical remission in pediatric patients with moderate to severe crohn's disease." Gastroenterology 1): S230-S231.	Duplicate
152. Pradhan, R., S. Sharma, R. Thakkar, A. Robinson, J. Hyams, J. Rosh, F. M. Ruemmele and W. Awni (2013). "Relationship between adalimumab concentration and efficacy for the induction of clinical remission in pediatric patients with moderate to severe Crohn's disease." Journal of Crohn's and Colitis 7: S164.	Insufficient data
153. Rai, T., U. Navaneethan, D. Dalal, B. Lashner and B. Shen (2012). "Clinical implications of measuring infliximab levels and human anti-chimeric antibodies in patients with inflammatory bowel disease." American Journal of Gastroenterology 107: S634.	Insufficient data
154. Rekvig, M., M. Gedde Dahl, J. Bratlie, N. Bolstad, B. Moum, J. Jahnsen and K. E. A. Lundin (2014). "Anti-TNFalpha drug level measurements in IBD patients." Journal of Crohn's and Colitis 8: S301-S302.	Insufficient data
155. Roblin, X., H. Marotte, E. Del Tedesco, M. Rinaudo-gaujous, J. M. Phelip and S. Paul (2013). "Residual adalimumab trough levels are associated with clinical remission and mucosal healing in IBD." Gastroenterology 1): S778.	Insufficient data
156. Roblin, X., M. Rinaudo, E. Del Tedesco, J. M. Phelip, L. Peyrin Biroulet and S. Paul (2014). "Development of an algorithm incorporating pharmacokinetics of adalimumab in inflammatory bowel diseases." Journal of Crohn's and Colitis 8: S41.	Insufficient data
157. Roblin, X., M. Rinaudo-gaujous, E. Del Tedesco, J. M. Phelip, C. Genin, L. Peyrin-Biroulet and S. Paul (2014). "Development of an algorithm incorporating pharmacokinetics of adalimumab in inflammatory bowel diseases." Gastroenterology 1): S-150.	Duplicate
158. Rosenthal, C., G. Melmed, B. Tripuraneni, J. Gebbia, S. Callejas, S. Farrior, S. Rabizadeh and M. Dubinsky (2012). "Early infliximab trough levels predict remission at one year in pediatric IBD patients." Inflamm Bowel Dis 18: S81.	Insufficient data
159. Rubin, D., S. Hauenstein and S. Singh (2013). "Post-marketing review of serum adalimumab and antibodies to adalimumab using the mobility shift assay platform." American Journal of Gastroenterology 108: S532.	Insufficient data
160. Scaldaferri, F., S. Pecere, V. Petito, G. Cammarota, M. C. Campanale, G. L. Rapaccini, A. Armuzzi, L. Guidi, A. Poscia, A. Amato, A. Sgambato, A. Gasbarrini and A. Papa (2012). "Infliximab and TNF ALFA measurement in intestinal mucosa of IBD patients: A new tool for the clinic?" Digestive and Liver Disease 44: S189-S190.	Insufficient data
161. Schatz, S. B., C. Prell, F. Freudenberg, T. Schwerd, P. Bufler and S. Koletzko (2011). "Correlation of infliximab levels and antibodies with clinical outcome in children with IBD." Journal of Pediatric Gastroenterology and Nutrition 52: E45.	Insufficient data

162. Semmler J, Pilch A, Armbruster F, et al. Development of a new immunoassay for the accurate determination of anti-infliximab antibodies in inflammatory bowel disease. Clin Chem Lab Med 2013;51 (10):eA27-8 doi: http://dx.doi.org/10.1515/cclm-2013-0737[published Online First: Epub Date]|.	Insufficient data
163. Semmler, J. M., A. Pilch, F. P. Armbruster, A. Dignass, W. Kruis and J. Stein (2014). "Development of a new immunoassay for the accurate determination of anti-infliximab antibodies in inflammatory bowel disease." Journal of Crohn's and Colitis 8: S41.	Duplicate
164. Semmler, J., A. Pilch, F. P. Armbruster, A. Dignass and J. Stein (2014). "Development of a new immunoassay for the accurate determination of anti-infliximab antibodies in inflammatory bowel disease." Clinical Chemistry and Laboratory Medicine 52: S1008.	Duplicate
165. Settesoldi, A., M. Giannotta, M. Milla, S. Genise, A. Santini, S. Bagnoli, V. Annese, A. Matucci, A. Vultaggio, G. Petroni, S. Pratesi, F. Nencini and E. Maggi (2012). "Loss of efficacy and adverse drug reactions during infliximab therapy in IBD patients are related to the appearance of anti-infliximab antibodies." Journal of Crohn's and Colitis 6: S151.	Duplicate
166. Settesoldi, A., M. Giannotta, S. Genise, A. Santini, S. Bagnoli, M. Milla, V. Annese, A. Matucci, A. Vultaggio, G. Petroni, S. Pratesi, F. Nencini and E. Maggi (2012). "Loss of efficacy and adverse drug reactions during infliximab therapy in IBD patients are related to the appearance of anti-infliximab antibodies." Digestive and Liver Disease 44: S194.	Insufficient data
167. Sharma, S., R. Pradhan, R. Thakkar, A. Robinson, J. Hyams, J. Rosh, F. M. Ruemmele and W. Awni (2013). "Relationship between adalimumab concentration and efficacy for the maintenance of clinical remission in pediatric patients with moderate to severe Crohn's disease." Journal of Crohn's and Colitis 7: S163.	Insufficient data
168. Sharma, S., R. S. Pradhan, R. Thakkar, A. Robinson, J. S. Hyams, J. R. Rosh, F. Ruemmele and W. M. Awni (2013). "Relationship between adalimumab concentration and efficacy for the maintenance of clinical remission in pediatric patients with moderate to severe crohn's disease." Gastroenterology 1): S231.	Insufficient data
169. Sorrentino, D., S. Hauenstein, M. Marino, S. Lockton, D. Zarifi, T. Del Bianco and S. Singh (2013). "Low dose infliximab for prevention of postoperative recurrence of crohn's disease: Long term follow-up and impact of infliximab trough levels and antibodies to infliximab." Gastroenterology 1): S777	Insufficient data
170. Steenholdt, C., J. Brynskov, O. Thomsen, L. K. Munck, J. Fallingborg, L. A. Christensen, G. Pedersen, J. Kjeldsen, K. Bendtzen and M. A. Ainsworth (2013). "Secondary infliximab treatment failure in crohn's disease: Therapeutic implications of measuring drug and anti-drug antibodies by three different binding assays." Gastroenterology 1): S773	Duplicate
171. Steenholdt, C., J. Brynskov, O. Thomsen, L. K. Munck, J. Fallingborg, L. A. Christensen, G. Pedersen, J. Kjeldsen, B. A. Jacobsen, A. S. Oxholm, J. Kjellberg, K. Bendtzen and M. A. Ainsworth (2013). "Treatment of secondary infliximab failure in crohn's disease based on serum levels of infliximab and antibodies against infliximab: The danish study of optimizing infliximab therapy in crohn's disease (do it crohn) randomized clinical trial." Gastroenterology 1): S22.	Superseded by full paper
172. Steenholdt, C., J. Brynskov, O. Thomsen, L. K. Munck, J. Fallingborg, L. A. Christensen, G. Pedersen, J. Kjeldsen, K. Bendtzen and M. A. Ainsworth (2013). "Secondary infliximab treatment failure in crohn's disease: Therapeutic implications of measuring drug and anti-drug antibodies by three different binding assays." Gastroenterology 1): S773.	Duplicate
173. Steenholdt, C., J. Brynskov, O. Thomsen, L. Munck, J. Fallingborg, L. Christensen, G. Pedersen, J. Kjeldsen, K. Bendtzen and M. Ainsworth (2013). "Secondary infliximab treatment failure in Crohn's disease:	Superseded by full

Therapeutic implications of measuring drug and anti-drug antibodies by three different binding assays." Journal of Crohn's and Colitis 7: S159.	text
174. Steenholdt, C., J. Brynskov, O. Thomsen, L. Munck, J. Fallingborg, L. Christensen, G. Pedersen, J. Kjeldsen, K. Bendtzen, S. Lockton, S. Hauenstein, R. Shringarpure, E. Chuang, S. Singh and M. Ainsworth (2012). "Comparison of techniques for monitoring infliximab and antibodies to infliximab in Crohn's disease patients with infliximab treatment failure." American Journal of Gastroenterology 107: S622.	Superseded by full text
175. Steenholdt, C., K. Bendtzen, J. Brynskov, O. O. Thomsen and M. A. Ainsworth (2014). "Clinical implications of measuring drug and anti-drug antibodies by different assays when optimizing infliximab treatment failure in Crohn's disease." Journal of Crohn's and Colitis 8: S291.	Duplicate
176. Steenholdt, C., K. Bendtzen, J. Brynskov, O. Thomsen and M. A. Ainsworth (2014). "Clinical implications of measuring drug and anti-drug antibodies by different assays when optimizing infliximab treatment failure in crohn's disease." Gastroenterology 1): S-240.	Superseded by full text
177. Steenholdt, C., K. Bendtzen, O. O. Thomsen, J. Brynskov and M. Ainsworth (2010). "Discriminating between response types in infliximab-treated patients with Crohn's disease: Sensitivity and specificity of combined assessment of infliximab trough levels and antidrug antibodies." Scandinavian Journal of Gastroenterology 45: 59-59.	Superseded by full text
178. Steenholdt, C., M. Svenson, K. Bendtzen, O. Thomsen, J. Brynskov and M. A. Ainsworth (2011). "Can measurements of anti-infliximab antibodies predict acute severe infusion reactions to inflixims241ab?" Gastroenterology 1): S774	Insufficient data
179. Steenholdt, C., M. Svenson, M. A. Ainsworth, O. Thomsen, J. Brynskov and K. Bendtzen (2012). "Comparison of techniques for monitoring infliximab bioavailability and immunogenicity in crohn's disease." Gastroenterology 1): S781	Insufficient data
180. Steenholdt, C., O. O. Thomsen, J. Brynskov, K. Bendtzen and M. A. Ainsworth (2010). "Discriminating between response types in infliximab-treated patients with Crohn's disease: Sensitivity and specificity of combined assessment of infliximab trough levels and anti-drug antibodies." Gastroenterology 1): S687-S688.	Insufficient data
181. Steenholdt, C., Y. Palarasah, K. Bendtzen, A. Teisner, B. Teisner, J. Brynskov and C. Nielsen (2013). "Pre-existing IgG antibodies to the Fab region of infliximab predict efficacy and safety in IBD patients naive to anti-TNF agents." Journal of Crohn's and Colitis 7: S6.	Insufficient data
182. Steenholdt, C., Y. Palarasah, K. Bendtzen, A. Teisner, J. Brynskov, B. Teisner and C. H. Nielsen (2013). "Pre-existing IGG antibodies to the fab region of infliximab predict efficacy and safety in ibd patients naive to anti-TNF agents." Scandinavian Journal of Immunology 77 (4): 333.	Insufficient data
183. Szepes, Z., E. Kunstar, K. Farkas, F. Nagy, R. Gyulai, R. Kui, A. Kinyo, A. Balint, M. Szucs, T. Wittmann and T. Molnar (2013). "Clinical utility of measuring serum TNF alpha level, anti TNF alpha levels and antibody titers in critical situations in inflammatory bowel disease and in psoriasis." Journal of Crohn's and Colitis 7: S118-S119.	Insufficient data
184. Tang, J., X. Gao, M. Zhi, H. Zhou, H. Chen, M. Zhang, Q. Yang and Z. Liang (2014). "Serum infliximab levels and early mucosal healing in Crohn's disease." Journal of Crohn's and Colitis 8: S209-S210.	Insufficient data
185. Turon, J., A. Langseder, R. Irizarry, K. Ahuja and J. R. Rosh (2013). "Clinical outcome of pediatric IBD patients after measurement of infliximab drug and anti-drug antibody levels." Gastroenterology 1): S531.	Insufficient data
186. Ungar B, Anafy A, Kopylov U, et al. The clinical and immunological significance of low level of infliximab in the absence of anti-infliximab antibodies in patients with IBD. Gastroenterology 2014;146(5 Suppl):S-245 doi: http://dx.doi.org/10.1016/S0016-5085%2814%2960862-3[published	Insufficient data

Online First: Epub Date]|.	
187. Ungar, B., A. Anafy, M. Yavzori, O. Picard, E. Fudim, U. Kopylov, Y. Ron, H. Yanai, I. Dotan, Y. Chowers, R. Eliakim and S. Ben-Horin (2014). "The clinical and immunological significance of low level of infliximab in the absence of anti-infliximab antibodies in patients with IBD." Journal of Crohn's and Colitis 8: S113.	Duplicate
188. Ungar, B., U. Kopylov, M. Yavzori, E. Fudim, O. Picard, A. Lahat, B. Avidan, A. Lang, B. Weiss, Y. Chowers, R. Eliakim and S. Ben-Horin (2014). "Predictors of formation of antibodies to infliximab (ATI) and secondary loss of response in IBD patients treated with infliximab." Journal of Crohn's and Colitis 8: S45.	Insufficient data
189. Ussia, V., L. Ceccarelli, S. Maltinti, G. Di Fluri, M. G. Mumolo, V. Bolognesi, A. Ricchiuti, M. Bellini, S. Marchi and F. Costa (2014). "A prospective assessment of antidrug antibody response over time by a new ELISA in patients with IBD treated with infliximab." Journal of Crohn's and Colitis 8: S298-S299.	Insufficient data
190. Van Der Woude, C. J., E. Bultman, J. Deuring, R. West, Z. Zelinkova and M. Peppelenbosch (2013). "Adalimumab trough levels in a prospective cohort of Crohn's disease patients." Journal of Crohn's and Colitis 7: S250.	Insufficient data
191. Van Der Woude, C. J., J. J. Deuring, R. West, Z. Zelinkova and M. P. Peppelenbosch (2013). "Adalimumab trough levels in a prospective cohort of crohn's disease patients." Gastroenterology 1): S567.	Duplicate
192. Van Moerkercke, W., C. Ackaert, G. Compernolle, M. Jurgens, I. Cleynen, G. A. Van Assche, P. J. Rutgeerts, A. Gils and S. Vermeire (2010). "High infliximab trough levels are associated with mucosal healing in Crohn's disease." Gastroenterology 1): S60.	Insufficient data
193. Van Moerkercke, W., G. Compernolle, C. Ackaert, A. Gils, S. Vermeire, M. Jurgens, I. Cleynen, G. Van Assche and P. Rutgeerts (2010). "Mucosal healing in Crohn's disease is associated with high infliximab trough levels." Journal of Crohn's and Colitis Supplements 4 (1): 30-31.	Insufficient data
194. Vande Casteele N, Peeters M, Compernolle G, et al. TNF-responsive cellular based assay reveals neutralizing capacity of anti-adalimumab antibodies in crohn's disease and ulcerative colitis patients. Gastroenterology 2014;146(5 Suppl):S-242 doi: http://dx.doi.org/10.1016/S0016-5085%2814%2960852-0[published Online First: Epub Date]|.	Insufficient data
195. Vande Casteele, N., A. Gils, G. Compernolle, V. Ballet, M. Peeters, K. Van Steen, S. Simoens, G. Van Assche, M. Ferrante, S. Vermeire and P. Rutgeerts (2013). "Drug level versus clinically based dosing of infliximab maintenance therapy in IBD: Final results of the randomized controlled taxit trial." Inflamm Bowel Dis 19: S2-S3.	Superseded by full text
196. Vande Casteele, N., G. Compernolle, V. Ballet, G. Van Assche, A. Gils, S. Vermeire and P. J. Rutgeerts (2012). "Results on the optimisation phase of the prospective controlled trough level adapted infliximab treatment (TAXIT) trial." Gastroenterology 1): S211-S212	Superseded by full text
197. Vande Casteele, N., G. Compernolle, V. Ballet, G. Van Assche, A. Gils, S. Vermeire and P. Rutgeerts (2012). "Individualised infliximab treatment using therapeutic drug monitoring: A prospective controlled Trough level Adapted infliXImab Treatment (TAXIT) trial." Journal of Crohn's and Colitis 6: S6.	Duplicate
198. Vande Casteele, N., K. Drake, S. Hauenstein, B. G. Levesque, S. Singh and W. Sandborn (2014). "Infliximab and antibody to infliximab concentrations in 7,613 patients shows indication for testing, association with loss of response and provides new insights into binding characteristics of anti-drug antibodies." Gastroenterology 1): S-242.	Insufficient data
199. Vande Casteele, N., L. Cuypers, S. Singh, L. Ohrmund, S. Hauenstein, G. Van Assche, P. J. Rutgeerts, A. Gils and S. Vermeire (2012). "Antibodies to infliximab can either be persistent or transient: A retrospective case-control study in ibd patients treated with infliximab maintenance therapy." Gastroenterology 1): S114.	Insufficient data

200. Vande Casteele, N., L. Cuypers, S. Singh, S. Hauenstein, L. Ohrmund, E. Chuang, P. Rutgeerts, A. Gils and S. Vermeire (2012). "Transient versus sustained antibodies to infliximab: Possibility to overcome low titer antibody responses by dose optimisation." Journal of Crohn's and Colitis 6: S110.	Insufficient data
201. Vande Casteele, N., M. Peeters, M. Ferrante, G. Compernolle, G. Van Assche, S. Vermeire and A. Gils (2014). "Functional cellular based assay reveals neutralising anti-drug antibodies in IBD patients treated with maintenance adalimumab." Journal of Crohn's and Colitis 8: S268-S269.	Duplicate
202. Vaughn, B. P., M. Martinez-Vazquez, V. Patwardhan, A. C. Moss, W. J. Sandborn and A. S. Cheifetz (2014). "A pilot study of optimized monotherapy with infliximab for patients with inflammatory bowel disease." Gastroenterology 1): S-55.	Superseded by full paper
203. Vaughn, B. P., M. Martinez-Vazquez, V. Patwardhan, A. C. Moss, W. J. Sandborn and A. S. Cheifetz (2014). "Prospective therapeutic drug monitoring to optimizing infliximab (IFX) maintenance therapy in patients with inflammatory bowel disease (IBD)." Gastroenterology 1): S-54.	Superseded by full paper
204. Vaughn, B., M. Matinez-Vazquez and A. Cheifetz (2013). "Infliximab dosing changes based on trough levels in a cohort of IBD patients in clinical remission." Inflamm Bowel Dis 19: S59.	Insufficient data
205. Velayos, F. S., S. Sheibani, S. Lockton, S. Hauenstein, S. Singh, J. P. Terdiman and U. Mahadevan (2013). "Prevalence of antibodies to adalimumab (ATA) and correlation between ATA and low serum drug concentration on CRP and clinical symptoms in a prospective sample of IBD patients." Gastroenterology 1): S91.	Insufficient data
206. Veres, G., J. L. Kaplan, E. De Greef, E. Chuang, D. Szabo, K. Molnar, L. Ohrmund, S. Hauenstein, S. Singh, A. Arato, G. Veereman and H. S. Winter (2012). "New assay to detect infliximab levels and anti-infliximab antibodies from a single serum sample is useful in measuring efficacy of treatment with infliximab in children with IBD." Gastroenterology 1): S386.	Insufficient data
207. Wang SL, Ohrmund L, Hauenstein S, et al. Evaluation of a novel homogeneous mobility shift assay for the measurement of human antibodies-To-Infliximab and infliximab levels in Patient serum. Am J Gastroenterol 2011;106:S475-6 doi: http://dx.doi.org/10.1038/ajg.2011.336_9[published Online First: Epub Date]|.	Insufficient data
208. Wang SL, Ohrmund L, Singh S. Measurement of human anti-chimeric antibodies (Haca) and infliximab levels in patient serum using a novel homogeneous assay. Gastroenterology 2010;1):S684-5	Insufficient data
209. Wang, S. L., L. Ohrmund, S. Hauenstein, J. Salbato, R. Reddy, P. Monk and S. Lockton (2011). "Evaluation of a novel homogeneous mobility shift assay for the measurement of human antibodies-to-infliximab and infliximab levels in patient serum." Arthritis and Rheumatism 1).	Duplicate
210. Wang, S. L., S. Hauenstein, L. Ohrmund, R. Shringarpure, D. C. Wolf, I. A. Diab, J. Salbato, R. Reddy, K. McCowen, S. Shah, S. Lockton, E. Chuang and S. Singh (2012). "Influence of trough serum drug level and immunogenicity on the lack of response to adalimumab therapy in inflammatory bowel disease patients." Arthritis and Rheumatism 64: S819-S820.	Insufficient data
211. Wang, S. L., S. Hauenstein, L. Ohrmund, R. Shringarpure, D. Wolf, I. Diab, J. Salbato, R. Reddy, K. McCowen, S. Shah, S. Lockton, E. Chuang and S. Singh (2012). "Influence of trough serum drug level and immunogenicity on the lack of response to adalimumab therapy in IBD patients presidential poster." American Journal of Gastroenterology 107: S680.	Duplicate
212. Ward MG, Kariyawasam VC, Mogan SB, et al. Clinical utility of measuring adalimumab trough levels and antibodies to adalimumab in patients with inflammatory bowel diseases. J Gastroenterol Hepatol 2013;28:100-01 doi: http://dx.doi.org/10.1111/jgh.12365-6[published Online First: Epub Date]|.	Insufficient data

213. Wolf, D. C., S. Hauenstein, S. Lockton and S. Singh (2013). "Mechanisms of loss of response to adalimumab in crohn's disease." Gastroenterology 1): S775	Insufficient data
214. Wolf, D. C., S. Lockton, S. Hauenstein, S. Carroll, S. Singh and E. Chuang (2013). "A multi-center observational study in community gastroenterology practices evaluating the clinical usage of testing for serum levels of infliximab and antibodies to infliximab." Gastroenterology 1): S423.	Insufficient data
215. Wolf, D., R. Shringarpure, S. Lockton, R. Corey, S. Woods, H. Aguilar and E. Chuang (2012). "Clinical experience with measurement of serum infliximab and antibodies to infliximab using a new homogenous mobility shift assay: Results of a multi-center observational study." American Journal of Gastroenterology 107: S658.	Insufficient data
216. Yamada, A., K. Sono, K. Takeuchi and Y. Suzuki (2013). "Clinical and basic studies to understand factors associated with the loss of response to infliximab in patients with Crohn's disease." Journal of Crohn's and Colitis 7: S239.	Insufficient data
217. Yanai, H., L. Lichtenstein, A. Assa, Y. Mazor, B. Weiss, A. Levine, Y. Ron, U. Kopylov, Y. Bujanover, Y. Rosenbach, B. Ungar, A. R. Eliakim, Y. Chowers, R. Shamir, G. Fraser, I. Dotan and S. Ben-Horin (2014). "Anti-TNF and anti-drug antibodies levels predict the ouCTomes of interventions after loss of response to adalimumab and infliximab." Gastroenterology 1): S-381.	Insufficient data
218. Yarur AJ, Deshpande AR, Sussman DA, et al. Serum adalimumab levels and antibodies correlate with endoscopic intestinal inflammation and inflammatory markers in patients with inflammatory bowel disease. Gastroenterology 2013;144(5 Suppl):S774-5	Insufficient data
219. Yarur, A., J. P. Trivella, D. A. Sussman, K. Drake, J. S. Barkin, S. Hauenstein, A. R. Deshpande, M. A. Quintero, S. Singh and M. T. Abreu (2014). "Anti-tumor necrosis factor drug levels and anti-bodies are associated with crohn's disease recurrence at the level of the ileo-colonic anastomosis after ileal resection." Gastroenterology 1): S243-S244.	Insufficient data
220. Yarur, A., K. Drake, M. Kubiliun, R. M. Dauer, D. A. Sussman, S. Hauenstein, M. A. Quintero, S. Singh, J. S. Barkin and M. T. Abreu (2014). "Anti-tumor necrosis factor levels are not associated with intestinal extent of mucosal inflammation in patients with inflammatory bowel diseases." Gastroenterology 1): S-244.	Insufficient data
221. Zelinkova, Z., M. P. Peppelenbosch, A. Van Liere-Baron, C. De Haar and C. J. Van Der Woude (2011). "Naturally-occurring autoantibodies against TNF-alpha are present in sera of inflammatory bowel disease patients and influence the response to adalimumab." Gastroenterology 1): S62.	Insufficient data

CD - Crohn’s disease; RA rheumatoid arthritis

Supplement 5 Drug cut-offs for predicting loss of or lack of regaining response

Supplement 5 Table 1 Drug cut-offs defined by ROC analysis in included studies using drug level as predictor of loss of or lack of regaining response (by assay type and drug)

Reference	Cut-off in µg/ml	Performance measures				AUC (95% CI)	Clinical marker	Drug	Assay
		**Sens**	**Spec**	**PPV**	**NPV**				
Bortlik 2013[41]	3	0.70	0.62	0.41	0.84	0.70 (0.57-0.83)	Sustained response (no treatment failure or drug intolerance, no surgery, IS introduction, steroids or Infliximab increase)	IFX	ELISA
Cornillie 2014[42]	3.5	0.64	0.78	0.56	0.83	0.75	Sustained response (CDAI score change)	IFX	ELISA
Steenholdt 2011[33]	0.5<br>2.2 (TL week 14)	0.86<br>0.79	0.85<br>0.94	NR	NR	0.93 (0.85-1.0)<br>0.93 (SE 0.04)	Maintained response (good response to induction therapy at 0, 2 and 6 weeks followed by good response to maintenance therapy)	IFX	RIA
Chiu 2013[47]	No Adalimumab concentration identified associated with clinical remission at any time point so clinical utility of measuring Adalimumab concentrations was difficult to assess	NR	NR	NR	NR	Week 4: 0.51<br>Week 24: 0.58<br>Week 56: 0.57	Clinical remission (CDAI <150)	ADA	ELISA
Imaeda 2014[36]	5.9	0.67	0.92	NR	NR	0.83 (0.80-0.95)	CRP ≤0.3mg/dL	ADA	ELISA
Mazor 2014[37]	5.85	0.68	0.71	NR	NR	0.75 (0.66-0.84)	Remission according to 2 physicians' assessment	ADA	ELISA
Roblin 2014[38]	4.85<br>4.9	0.81<br>0.66	0.67<br>0.85	0.84<br>0.88	0.57<br>0.51	0.73<br>0.77	Clinical remission (CDAI <150)<br>MH (disappearance of all ulcerations on endoscopy)	ADA	ELISA
Frederiksen 2014[39]	14.5<br>0.35<br>6.85	1.00<br>0.50<br>0.69	0.12<br>0.96<br>0.69	0.41<br>0.89<br>0.58	1.00<br>0.76<br>0.78	0.77 (0.62-0.93)	LOR (physician's global assessment)	ADA	RIA

Supplement 5 Table 2 Drug cut-offs in included studies not reporting a ROC analysis and using drug level as predictor of loss of or lack of regaining response (by assay type)

Reference	Cut-off in µg/ml	Source of cut-off	Drug	Assay
Hibi 2014[43]	1	Maser 2006[46]	IFX	ELISA
Imaeda 2012[26]	0.66	95th percentile value from 35 patients who had never received Infliximab	IFX	ELISA
Kopylov 2012[28]	Unclear	Unclear	IFX	ELISA
Maser 2006[46]	1.4	Unclear	IFX	ELISA
Yanai 2012[44] abstract	1	Unclear	IFX	ELISA
Ben Bassat 2013[45] abstract	2	Derived from data not pre-specified	IFX	HMSA
Ainsworth 2008[22]	0.5	Derived from data not pre-specified	IFX	RIA
Steenholdt 2014[23]	0.5	Steenholdt 2011[33]	IFX	RIA

Supplement 5 Table 3 Additional studies reporting drug cut-offs derived by ROC analysis but not reporting sufficient 2x2 data for using drug level as predictor of loss of or lack of regaining response (by assay type and drug)

Reference	Cut-off in µg/ml	Performance measures				AUC (95% CI)	Clinical marker	Drug	Assay
		Sens	Spec	PPV	NPV				
Goldberg R, Beswick L, Van Langenberg D, et al. Journal of Crohn's and Colitis 2014;8:S223 Abstract	3	0.90	0.37	NR	NR	0.75	Disease activity (physicians global assessment and CRP levels)	IFX	ELISA
Imaeda H, Bamba S, Takahashi K, et al. J Gastroenterol 2014;49(4):674-82	0.6<br>1.0<br>1.1<br>4.0	0.73<br>0.67<br>0.72<br>0.71	0.62<br>0.71<br>0.56<br>0.70	NR<br>NR<br>NR<br>NR	NR<br>NR<br>NR<br>NR	0.67 (0.60-0.81)<br>0.72 (0.50-0.73)<br>0.63 (0.55-0.65)<br>0.63 (0.56-0.70)	CRP ≤0.3mg/dL<br>Serum albumin (≥ 4.0mg/dL)<br>FC (≤ 300µg/g)<br>MH (Rutgeerts scoring system 0 or 1)	IFX	ELISA
Marits P, Landucci L, Sundin U, et al. Journal of Crohn's & colitis 2014;8(8):881-9	4.1	0.87	0.44	NR	NR	0.74 (SE 0.037)	Remission (HBI <5 and CRP < 3 mg/l)	IFX	ELISA
Nagore D, Ruiz Del Agua	0.8	0.86	0.75	NR	NR	0.86 (0.76-0.96)	Active disease	IFX	ELISA

Reference	Cut-off in µg/ml	Performance measures				AUC (95% CI)	Clinical marker	Drug	Assay
		Sens	Spec	PPV	NPV				
A, Pascual J, et al. Therapeutic (TU1325). Gastroenterology 2015;148(4 Suppl 1):S-860									(Promonitor)
Pallagi-Kunstar E, Farkas K, Szepes Z, et al. World J Gastroenterol 2014;20(17):5031-5	3.01	NR	NR	NR	NR	NR	Detecting anti-drug antibodies	IFX	ELISA
Paul S, Tedesco ED, Marotte H, et al.. Gastroenterology 2012;142(5 Suppl):S354	2	0.76	0.82	NR	NR	0.60	Remission (CDAI score <150)	IFX	ELISA
Paul S, Del Tedesco E, Marotte H, et al.. Inflamm Bowel Dis 2013;19(12):2568-76	0.5 (trough after optimisation minus trough before optimisation)	0.88	0.76	0.78	0.86	0.91 (0.83-1.0)	Mucosal healing (FC <250µg/g)	IFX	ELISA (
Singh N, Rosenthal CJ, Melmed GY, et al. Inflamm Bowel Dis 2014;20(10):1708-13	4<br>7	0.53<br>0.33	0.75<br>1.00	0.76<br>1.00	0.52<br>0.50	0.64 (0.51-0.75)<br>0.67 (0.58-0.75)	Week 14 Infliximab levels as predictor of week 54 clinical remission according to CDAI	IFX	ELISA
Baert F, Drobne D, Gils A, et al. Clin Gastroenterol Hepatol 2014;12(9):1474-81	2 (after re-exposure to Infliximab)	NR	NR	NR	NR	0.76 (0.62-0.90)	Long term response (clinical assessment [HBI] and CRP levels[<3mg/l])	IFX	HMSA
Levesque BG, Greenberg GR, Zou G, et al. Aliment Pharmacol Ther 2014;39(10):1126-35	3	NR	NR	NR	NR	NR	Disease activity at week 8 (≥70 point increase in CDAI and CRP >5µg/l)	IFX	HMSA
Vande Casteele N, Gils A, Singh S, et al. Am J Gastroenterol 2013;108(6):962-71	13 (TL week 6)	0.72	0.81	NR	NR	0.87 (SE 0.06)	anti-drug antibody formation	IFX	HMSA
Feagan BG, Singh S, Lockton S, et al. Gastroenterology 2012;142(5 Suppl):S-114 Abstract	3	NR	NR	NR	NR	0.74	Disease activity	IFX	HPLC based fluid phase assay
Goldberg R, Beswick L, Van Langenberg D, et al. Journal of Crohn's and	3	0.83	0.63	NR	NR	0.8	Disease activity (physicians global assessment and CRP levels)	ADA	ELISA

Reference	Cut-off in µg/ml	Performance measures				AUC (95% CI)	Clinical marker	Drug	Assay
		Sens	Spec	PPV	NPV				
Colitis 2014;8:S223 Abstract									
Karmiris K, Paintaud G, Noman M, et al. Gastroenterology 2009;137(5):1628-40	0.33	0.95	NR	0.81	NR	NR	Sustained clinical benefit (patient reporting lasting control of disease with possible dose escalation)	ADA	ELISA
Ward MG, Kariyawasam VC, Mogan SB, et al. J Gastroenterol Hepatol 2013;28:100-01 Abstract	4.9	0.83	0.65	NR	NR	0.75	Remission	ADA	LISA
arur AJ, Deshpande AR, Sussman DA, et al. Gastroenterology 2013;144(5 Suppl):S774-5 Abstract	5	NR	NR	NR	NR	0.71	Elevation of CRP	ADA	HMSA
Mazor Y, Kopylov U, Hur DB, et al. Gastroenterology 2013;144(5 Suppl):S-778 Abstract	5	NR	NR	NR	NR	0.77 (0.67-0.86)	Clinical response and normal CRP	ADA	NR

Supplement 6 Summary of quality assessment results using the QUADAS-2 tool with index questions adapted to the review for studies comparing performance of different tests

Supplement 6 Table 1Tabular presentation of QUADAS-2 results

Study	RISK OF BIAS				APPLICABILITY CONCERNS		
	PATIENT SELECTION	INDEX TEST	REFERENCE STANDARD	FLOW AND TIMING	PATIENT SELECTION	INDEX TEST	REFERENCE STANDARD
Ainsworth 2008[22]	☹	☺	☹	☺	☺	☺	☹
Baert 2014[20]	☺	☺	☹	☺	☺	☺	☹
Ben-Horin 2011[34]	☹	☺	☹	☺	☺	☹	☹
Ben-Horin 2012[17]	☹	☺	☹	☺	☺	☺	☹
Bortlik 2013[41]	☹	☺	☹	☺	☺	☺	☹
Candon 2005[18]	☹	☺	☺	☺	☺	☺	☺
Chiu 2013[47]	☹	☹	☺	☺	☺	☹	☺
Cornillie 2014 [42]	☹	☹	☺	☹	☺	☹	☺
Farrell 2003[24]	☺	☺	☹	☺	☺	☺	☹
Frederiksen 2014[39]	☹	☺	☹	☺	?	☺	☹
Hanauer 2004[25]	☹	☹	☺	☹	☺	☹	☺
Hibi 2014[43]	☹	☺	☺	☺	☺	☺	☺
Imaeda 2012[26]	☹	☺	☺	☺	☺	☺	☺
Imaeda 2014[36]	☹	☺	☺	☺	☺	☺	☺
Kopylov 2012[28]	☹	☺	☹	☺	☺	☺	☹
Maser 2006[46]	☺	☺	☺	☺	☺	☺	☺
Mazor 2014 [37]	☺	☺	☺	☺	☺	☺	☺
Pariente 2012[19]	☹	☺	?	☺	☹	☺	?
Roblin 2014[38]	☹	☺	☺	☺	☺	☺	☺
Steenholdt 2011[33]	☹	☺	☹	☺	☺	☺	☹
Steenholdt 2013[31]	☹	☺	☹	☹	☺	☺	☹
Steenholdt 2014[23]	☺	☺	☺	☺	☺	☺	☺
Van Casteele 2013[21]	☹	☺	☺	☺	☹	☺	☺
West 2008[40]	☹	☺	☹	☺	☺	☺	☹

☺Low Risk ☹High Risk ? Unclear Risk

Supplement 6 Figure 1 Graphical summary presentation of QUADAS-2 quality assessment results

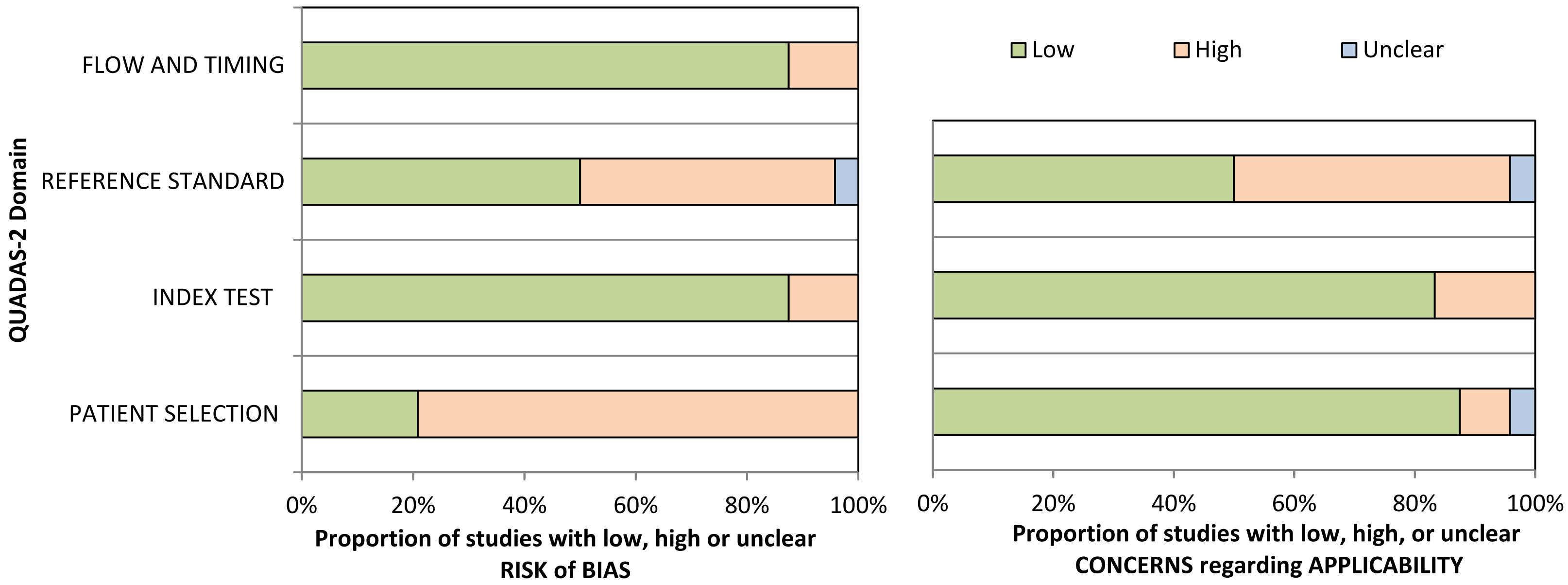

Supplement 7 Results of hierarchical meta-analysis of included studies

Supplement 7 Table 1 Test accuracy statistics from hierarchical meta-analyses

**Trough Infliximab level as predictor of loss or absence of response**				
Studies included	parameter	Point estimate	95% LCI	95% UCI
all 11 studies	Sens	0.657232	0.546288	0.753299
all 11 studies	Spec	0.80625	0.744166	0.85618
all 11 studies	DOR	7.978975	4.119972	15.45254
all 11 studies	LR+	3.392169	2.35152	4.893351
all 11 studies	LR-	0.425139	0.305104	0.592398
all 11 studies	1/LR-	2.352175	1.688056	3.277573
responder populations only	Sens	0.681452	0.592117	0.759178
responder populations only	Spec	0.790873	0.723301	0.845468
responder populations only	DOR	8.090128	4.353039	15.03551
responder populations only	LR+	3.258549	2.287802	4.641198
responder populations only	LR-	0.402781	0.298559	0.543385
responder populations only	1/LR-	2.482739	1.840315	3.349423
ELISA studies only	Sens	0.652104	0.564027	0.730877
ELISA studies only	Spec	0.789041	0.691592	0.861849
ELISA studies only	DOR	7.010794	3.450232	14.24578
ELISA studies only	LR+	3.091133	1.959085	4.877331
ELISA studies only	LR-	0.440911	0.329778	0.589495
ELISA studies only	1/LR-	2.268033	1.696367	3.032348
**Trough level of antibodies to Infliximab as predictor of loss or absence of response**				
Studies included	parameter	Point estimate	95% LCI	95% UCI
all 20 studies	Sens	0.559745	0.444812	0.668611
all 20 studies	Spec	0.792243	0.688105	0.868267
all 20 studies	DOR	4.848283	2.519589	9.329239
all 20 studies	LR+	2.694226	1.72293	4.213088
all 20 studies	LR-	0.555707	0.426575	0.72393
all 20 studies	1/LR-	1.799509	1.38135	2.344251
all studies minus outliers*	Sens	0.597	0.477	0.707
all studies minus outliers*	Spec	0.807	0.742	0.859
all studies minus outliers*	DOR	6.183	3.805	10.050
all studies minus outliers*	LR+	3.088	2.311	4.127
all studies minus outliers*	LR-	0.500	0.381	0.655

all studies minus outliers	1/LR-	2.002	1.528	2.623
responder populations only	Sens	0.570	0.445	0.687
responder populations only	Spec	0.849	0.787	0.896
responder populations only	DOR	7.460	4.544	12.250
responder populations only	LR+	3.778	2.722	5.244
responder populations only	LR-	0.506	0.388	0.660
responder populations only	1/LR-	1.974	1.514	2.574
ELISA studies only	Sens	0.482	0.355	0.611
ELISA studies only	Spec	0.880	0.841	0.911
ELISA studies only	DOR	6.830	3.872	12.050
ELISA studies only	LR+	4.022	2.805	5.768
ELISA studies only	LR-	0.589	0.459	0.755
ELISA studies only	1/LR-	1.698	1.324	2.178
**Trough Adalimumab level as predictor of loss or absence of response**				
	Parameter	Point estimate	95% LCI	95% UCI
All 5 studies	Sens	0.543476	0.246586	0.812386
All 5 studies	Spec	0.640241	0.325873	0.86758
All 5 studies	DOR	2.118592	0.172646	25.99789
All 5 studies	LR+	1.510665	0.38102	5.989464
All 5 studies	LR-	0.713051	0.229687	2.213631
All 5 studies	1/LR-	1.402424	0.451747	4.353753
All studies minus Chiu	Parameter	Point estimate	95% LCI	95% UCI
All studies minus Chiu	Sens	0.684	0.591	0.764
All studies minus Chiu	Spec	0.786	0.643	0.883
All studies minus Chiu	DOR	7.971	3.646	17.428
All studies minus Chiu	LR+	3.201	1.822	5.623
All studies minus Chiu	LR-	0.402	0.297	0.542
All studies minus Chiu	1/LR-	2.490	1.844	3.363
**Trough level of antibodies to Adalimumab as predictor of loss or absence of response**				
	Parameter	Point estimate	95% LCI	95% UCI
All 6 studies	Sens	0.471206	0.2903357	0.66
All 6 studies	Spec	0.915467	0.7939073	0.968
All 6 studies	DOR	9.65022	4.387759	21.22
All 6 studies	LR+	5.574189	2.646268	11.74
All 6 studies	LR-	0.577623	0.4208713	0.793

All 6 studies	1/LR-	1.731233	1.261422	2.376
	Parameter	Point estimate	95% LCI	95% UCI
All studies minus Mazor	Sens	0.542264	0.3611645	0.713
All studies minus Mazor	Spec	0.884874	0.7444581	0.953
All studies minus Mazor	DOR	9.105532	3.764526	22.02
All studies minus Mazor	LR+	4.710191	2.221639	9.986
All studies minus Mazor	LR-	0.517289	0.361111	0.741
All studies minus Mazor	1/LR-	1.933156	1.349505	2.769

Sens = sensitivity; Spec = specificity; DOR = diagnostic odds ratio; LR+ = positive likelihood ratio; LR- = negative likelihood ratio; 1/LR- = inverse of negative likelihood ratio.

*Outliers are Ainsworth 2008 and Steenholdt 2014

Supplement 7 Figure 1. Hierarchical meta-analysis of studies of trough levels of antibodies to Adalimumab (upper row) and of Adalimumab (lower row) for predicting loss of response or failure to regain response

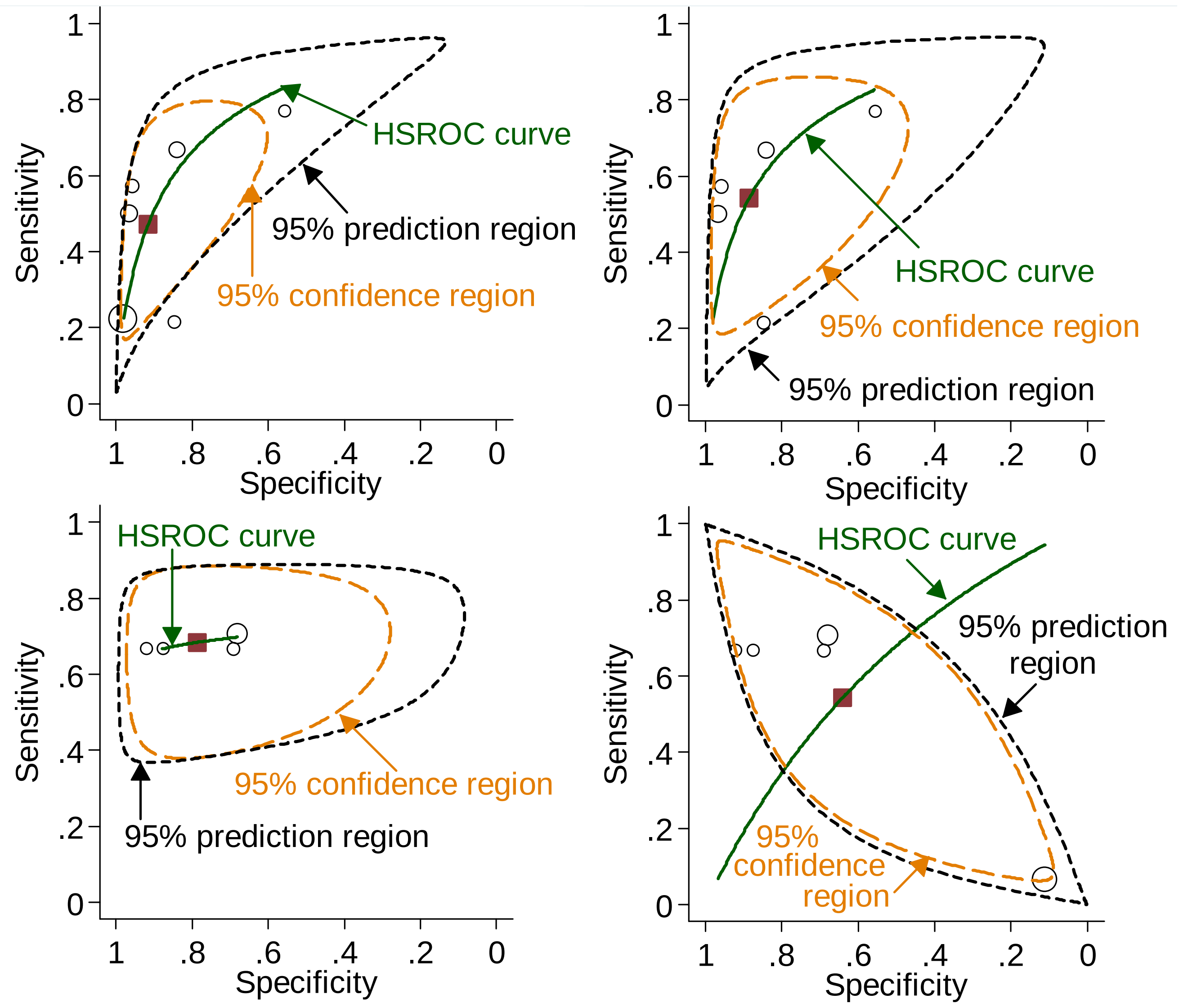

Top Upper left = all anti-Adalimumab antibody studies; upper right = anti-Adalimumab antibody studies but omitting the study of Mazor; lower left Adalimumab studies but omitting the study of patients with secondary loss of response (Chiu); lower right = all Adalimumab studies. The square symbol represents the summary point estimate on the HSROC curve. Mazor was omitted because it was a particularly large and influential study.

Supplement 8 Impact of additional studies on meta-analysis results

Supplement 8 Table 1 Characteristics of additional studies identified by search update

STUDY	DRUG	DIAGNOSIS	RESPONSE/LOR	TEST	RESPONSE MEASURE
Infliximab trough level as predictor of loss of or lack of regaining response					
Levesque 2014 [51]	IFX	CD	LOR	HMSA	≥ 70 CDAI increase
Reinisch 2016 [53]	IFX	CD	LOR	ELISA	Mucosal healing
Ungar 2016 [52]	IFX	CD	LOR	HMSA	Mucosal healing
Adalimumab trough level as predictor of loss of or lack of regaining response					
Bodini 2015 [54]	ADA	CD	LOR	HMSA	> 7 HBI
Morita 2016 [57]	ADA	CD	LOR	ELISA	Mucosal healing
Ungar 2016 [52]	ADA	CD	LOR	HMSA	Mucosal healing
Yarur 2016 [56]	ADA	IBD ~0.89 CD	LOR	HMSA	Mucosal healing
Zittan 2016 [55]	ADA	CD	LOR	HMSA	Mucosal healing

Diagnosis = study patient population; LOR = patients with loss of response; Response measure = method used for defining clinical response; ADA = Adalimumab; IFX = Infliximab; CD = Crohn's disease; IBD = inflammatory bowel disease; ELISA = enzyme linked immunoassay; HBI = Harvey-Bradshaw Index; HMSA= Homogenous Mobility Shift Assay; CDAI = Crohn's disease activity index score.

Sensitivity and specificity pairs for the new studies are shown in Supplement 8 Figure 1 together with those for earlier studies.

Infliximab trough levels

Study	TP	FP	FN	TN	assay	POP	RES	Sensitivity (95% CI)	Specificity (95% CI)
Reinisch	38	29	15	70	ELISA	LR	MH	0.72 [0.58, 0.83]	0.71 [0.61, 0.79]
Levesque	178	11	113	25	HMSA	LR	CDAI	0.61 [0.55, 0.67]	0.69 [0.52, 0.84]
Ungar 2016	38	15	7	7	HMSA	LR	MH	0.84 [0.71, 0.94]	0.32 [0.14, 0.55]
Ainsworth 2008	7	1	1	18	RIA	LR	PJ	0.88 [0.47, 1.00]	0.95 [0.74, 1.00]
Steenholdt 2014	10	7	21	31	RIA	LR	CDAI	0.32 [0.17, 0.51]	0.82 [0.66, 0.92]
Bortlik 2013	16	23	7	38	ELISA	R	PJ	0.70 [0.47, 0.87]	0.62 [0.49, 0.74]
Cornillie 2014	14	11	8	38	ELISA	R	CDAI	0.64 [0.41, 0.83]	0.78 [0.63, 0.88]
Hibi 2014	8	4	7	22	ELISA	R	CDAI	0.53 [0.27, 0.79]	0.85 [0.65, 0.96]
Imaeda 2012	9	8	8	33	ELISA	R	CDAI	0.53 [0.28, 0.77]	0.80 [0.65, 0.91]
Kopylov 2012	21	2	9	31	ELISA	R	PJ	0.70 [0.51, 0.85]	0.94 [0.80, 0.99]
Yanai 2012	7	10	7	16	ELISA	R	PJ	0.50 [0.23, 0.77]	0.62 [0.41, 0.80]
Ben-Bassat 2013	50	29	10	145	HMSA	R	HBI	0.83 [0.71, 0.92]	0.83 [0.77, 0.89]
Steenholdt 2011	18	7	3	41	RIA	R	PJ	0.86 [0.64, 0.97]	0.85 [0.72, 0.94]
Maser 2006	22	11	13	44	UC	R	HBI	0.63 [0.45, 0.79]	0.80 [0.67, 0.90]

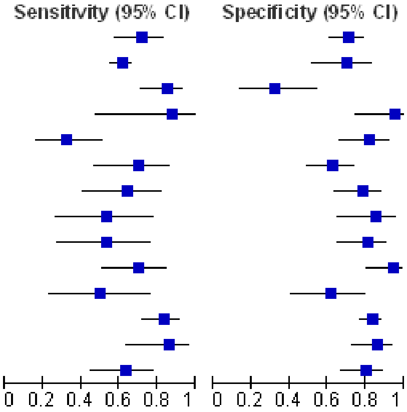

Adalimumab trough levels

Study	TP	FP	FN	TN	assay	POP	RES	Sensitivity (95% CI)	Specificity (95% CI)
Roblin 2014	16	2	8	14	ELISA	R	MH	0.67 [0.45, 0.84]	0.88 [0.62, 0.98]
Mazor 2014	48	16	20	34	ELISA	R	PJ CRP	0.71 [0.58, 0.81]	0.68 [0.53, 0.80]
Imaeda 2014	10	2	5	23	ELISA	R	CRP	0.67 [0.38, 0.88]	0.92 [0.74, 0.99]
Frederiksen 2014	12	9	6	20	RIA	R	PJ BM	0.67 [0.41, 0.87]	0.69 [0.49, 0.85]
Chiu 2013	7	7	56	98	ELISA	LR	CDAI100	0.11 [0.05, 0.22]	0.93 [0.87, 0.97]
Bodini 2015	13	2	0	8	ELISA	LR	HBI	1.00 [0.75, 1.00]	0.80 [0.44, 0.97]
Morita 2016	24	4	4	10	ELISA	LR	MH	0.86 [0.67, 0.96]	0.71 [0.42, 0.92]
Ungar 2016	36	22	6	14	ELISA	LR	MH	0.86 [0.71, 0.95]	0.39 [0.23, 0.57]
Yarur 2016	31	11	6	29	ELISA	LR	MH	0.84 [0.68, 0.94]	0.72 [0.56, 0.85]
Zittan 2016	19	3	6	32	ELISA	LR	MH	0.76 [0.55, 0.91]	0.91 [0.77, 0.98]

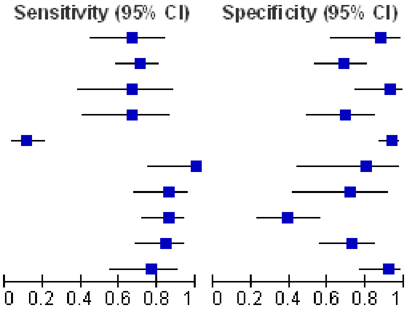

Supplement 8 Figure 1 Paired forest plots for trough anti-TNF levels for predicting loss of response or failure to regain response to Infliximab (upper, 3 new studies at the top) and Adalimumab (lower, 5 new studies at the bottom);
RES = criterion for determining clinical response, POP = study patient population, RIA = radioimmunoassay, HMSA = homogeneous mobility shift assay, ELISA = enzyme linked immunoassay, LR = patients with loss of response, R = patients with response, UC = unclear, PJ BM = physicians' judgement and biological measure; PJ = physicians' judgement, HBI = Harvey Bradshaw Index score, CDAI = Crohn's disease activity index score, CRP = C-reactive protein level, MH = mucosal healing

Meta-analysis of Infliximab trough studies

Three new studies were identified reporting test accuracy of infliximab trough levels to predict loss of response bringing the total number of studies available for meta-analysis to 14.[51-53] The meta-analysis summary estimates of test accuracy for the original eleven and of the 14 studies are summarised in Supplement 8 Table 2.

Supplement 8 Table 2 Test accuracy statistics from hierarchical meta-analyses (infliximab studies)

Trough Infliximab level as predictor of loss or absence of response				
Studies included	parameter	SummaryPoint estimate	95% LCI	95% UCI
original 11 studies	Sens	0.657232	0.546288	0.753299
original 11 studies	Spec	0.80625	0.744166	0.85618
original 11 studies	DOR	7.978975	4.119972	15.45254
original 11 studies	LR+	3.392169	2.35152	4.893351
original 11 studies	LR-	0.425139	0.305104	0.592398
original 11 studies	1/LR-	2.352175	1.688056	3.277573
**Updated analysis including three new studies**				
all 14 studies	Sens	0.674018	0.587579	0.750047
all 14 studies	Spec	0.774693	0.696482	0.837453
all 14 studies	DOR	7.109369	4.225833	11.96051
all 14 studies	LR+	2.991547	2.163908	4.135736
all 14 studies	LR-	0.420789	0.325131	0.544592
all 14 studies	1/LR-	2.376486	1.836237	3.075685
**Change in summary estimates after including 3 new studies**				
	Sens	0.016786	0.041291	-0.00325
	Spec	-0.03156	-0.04768	-0.01873
	DOR	-0.86961	0.105861	-3.49203
	LR+	-0.40062	-0.18761	-0.75762
	LR-	-0.00435	0.020027	-0.04781
	1/LR-	0.024311	0.148181	-0.20189
Sens = sensitivity; Spec = specificity; DOR = diagnostic odds ratio; LR+ = positive likelihood ratio; LR- = negative likelihood ratio; 1/LR- = inverse of negative likelihood ratio.				

Adding the three new studies has very little impact on the meta-analysis summary test statistic estimates or upon their associated uncertainty. Figure 2 shows the summary ROC plots for the 11 and 14 studies.

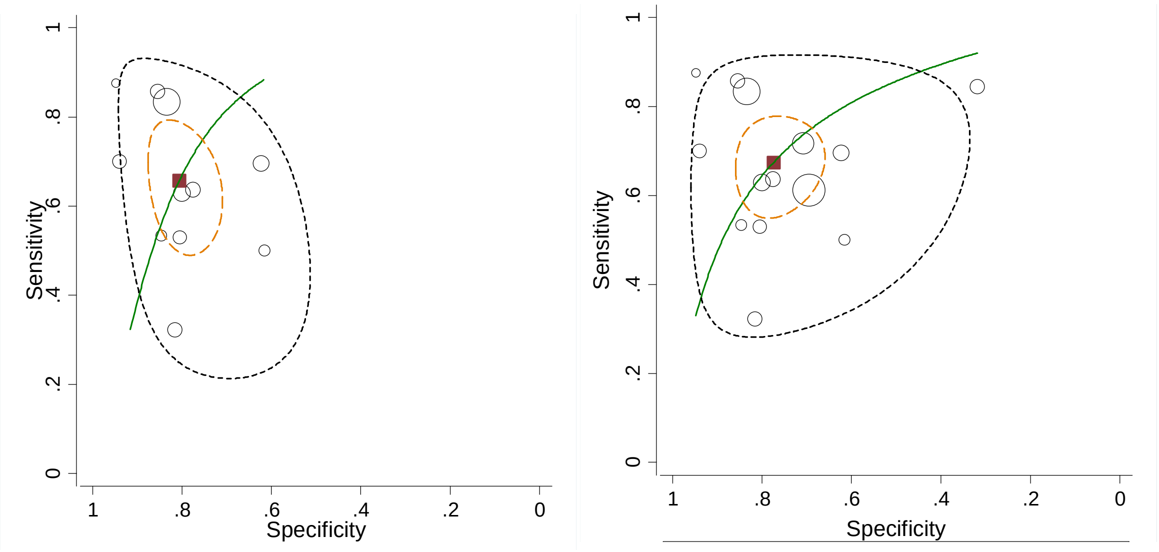

Supplement 8 Figure 2 Summary ROC plots for 11 (left) and 14 (right) studies of Infliximab trough levels

Adalimumab trough studies

Five new studies were identified reporting test accuracy of adalimumab trough levels to predict loss of response,[54-57] thereby bringing the total number of studies available for meta-analysis to nine. The meta-analysis summary estimates for the original four and for the nine studies are summarised in Supplement 8 Table 3.

Supplement 8 Table 3 Test accuracy statistics from hierarchical meta-analyses (Adalimumab studies)

**Trough Infliximab level as predictor of loss or absence of response**				
Studies included	parameter	SummaryPoint estimate	95% LCI	95% UCI
original 4 studies	Sens	0.684251	0.5914862	0.7643434
original 4 studies	Spec	0.7862228	0.6427244	0.8826122
original 4 studies	DOR	7.969987	3.64723	17.41615
original 4 studies	LR+	3.200767	1.823276	5.618956
original 4 studies	LR-	0.4016025	0.2973622	0.5423841
original 4 studies	1/LR-	2.490025	1.843712	3.362902
**Updated analysis including three new studies**				
all 9 studies	Sens	0.6357	0.547669	0.715498
all 9 studies	Spec	0.710633	0.591235	0.806565
all 9 studies	DOR	4.285374	1.929981	9.515341
all 9 studies	LR+	2.196862	1.378996	3.499796
all 9 studies	LR-	0.512642	0.363406	0.723164
all 9 studies	1/LR-	1.950679	1.382813	2.751747
**Change in summary estimates after including 5 new studies**				
	Sens	-0.04855	-0.04382	-0.04885
	Spec	-0.07559	-0.05149	-0.07605
	DOR	-3.68461	-1.71725	-7.90081
	LR+	-1.00391	-0.44428	-2.11916
	LR-	0.111039	0.066043	0.18078
	1/LR-	-0.53935	-0.4609	-0.61116
Sens = sensitivity; Spec = specificity; DOR = diagnostic odds ratio; LR+ = positive likelihood ratio; LR- = negative likelihood ratio; 1/LR- = inverse of negative likelihood ratio. Note: the outlier study of Chiu 2013_ENREF_47 has been omitted from the analyses				

With the exception of estimated DOR, most summary test statistics remain relatively unaltered by the addition of the five new studies. Introduction of the new studies has somewhat reduced the uncertainty of the estimates. The considerable heterogeneity amongst the studies is evident when comparing summary ROC plots for the four and nine studies (Supplement 8 Figure 3).

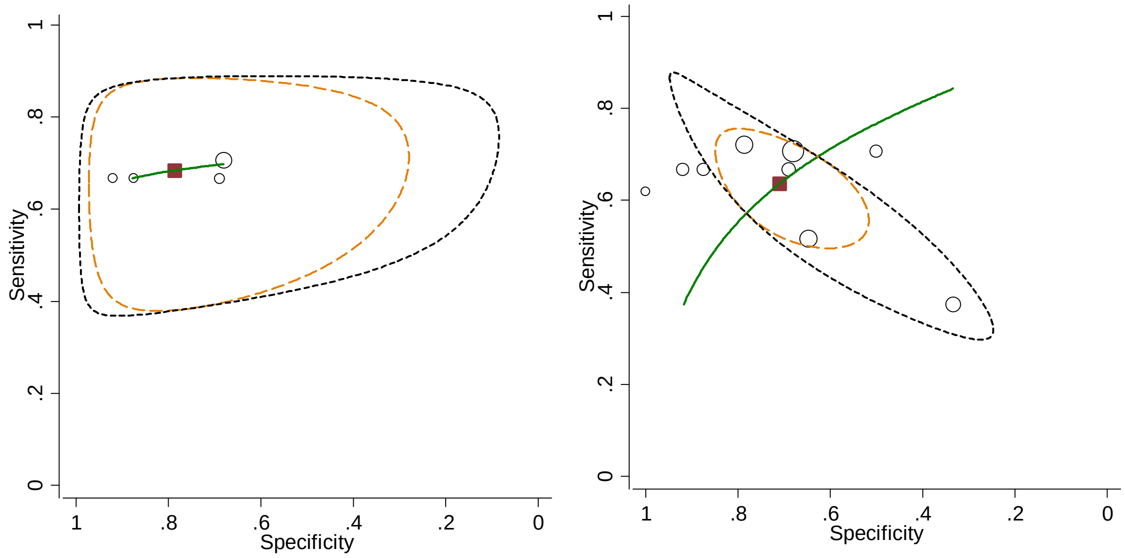

Supplement 8 Figure 3 Summary ROC plots for 4 (left) and 9 (right) studies of Adalimumab trough levels

Note: the outlier study of Chiu 2013 has been omitted from the analyses
