# Supplementary material for: Test accuracy of drug and antibody assays for predicting response to antitumour necrosis factor treatment in Crohn’s disease: a systematic review and meta-analysis
Source: BMJ Open. 2017 Jul 2;7(6):e014581. doi: 10.1136/bmjopen-2016-014581 (PMC5734585; doi:10.1136/bmjopen-2016-014581)
Supplement: Supplementary data 1 [file bmjopen-2016-014581supp001.pdf]

Diagnostic Assessment Report commissioned by the NIHR HTA Programme on behalf of the National Institute for Health and Clinical Excellence – Final Protocol

Title of project

Crohn's disease: Tests for therapeutic monitoring of TNF inhibitors (LISA-TRACKER ELISA kits, TNFα-Blocker ELISA kits, and Promonitor ELISA kits)

Name of External Assessment Group (EAG) and project lead

Produced by:	Warwick Evidence
Lead author:	Karoline Freeman
Co-authors:	Martin Connock
	Hema Mistry
	Sian Taylor-Phillips
	Rachel Court
	Alexander Tsertsvadze
	Jason Madan
	Ngianga-Bakwin Kandala
	Ramesh Arasaradnam
	Aileen Clarke
	Paul Sutcliffe
Correspondence to:	Dr Paul Sutcliffe
	Associate Professor
	Deputy Director for Warwick Evidence
	Populations, Evidence and Technologies
	Division of Health Sciences
	Warwick Medical School
	University of Warwick
	Coventry CV4 7AL
Tel:	02476 150189
Fax:	02476 528375
Email:	p.a.sutcliffe@warwick.ac.uk

Date completed: 29 October 2014

The views expressed in this protocol are those of the authors and not necessarily those of the NIHR HTA Programme. Any errors are the responsibility of the authors. The authors have no conflicts of interest.

Glossary of terms

Induction therapy	Treatment to induce remission
Maintenance therapy	Treatment to remain in remission
Remission	Period without or only mild symptoms
Biologics or biological therapy	A protein-based drug derived from living cells cultured in a laboratory
Immunosuppressant	A class of drugs that suppress or reduce the strength of the body's immune system
Resection	The removal by surgery of all or part of an organ such as the bowel
Ileostomy	Surgical procedure where the small intestine is diverted through an opening in the abdomen
Intestinal stricture	Narrowing of the intestine due to tissue scaring following inflammation
Fistulas	Channels formed from the digestive system to other parts of the digestive system or different organs
Azathioprine	Immunomodulator
Thiopurines	Group of drugs (purine antimetabolites) including azathioprine, 6-mercaptopurine and 6-thioguanine
Seton	A thread, wire, or gauze of cotton or other absorbent material passed below the skin and left with the ends protruding, to promote drainage of fluid
Methotrexate	Disease-modifying, antimetabolite

1. Plain English Summary

Crohn's disease is an uncommon long term disease involving painful and damaging inflammation of the gut lining. Damage can cause bloody stools, development of very narrow sections along the gut (strictures), and the formation of abnormal channels (fistulas) between different regions of the gut or between gut and body surface or between gut and nearby organs. Particularly distressing fistulas may occur between intestine and vagina in female patients. During a patient's life the severity of Crohn's disease fluctuates between remission (no symptoms) and relapse (active disease) and treatments aim to induce and maintain remission. Tumour necrosis factor (TNF) has been identified as a molecule important in the development of inflammation in Crohn's disease. Medicines called anti-TNF agents have been developed that counteract the action of TNF and have been found to benefit Crohn's disease patients; they are by far the most expensive medicines used for Crohn's disease and, like all Crohn's disease medicines, for some patients they are associated with unwanted side effects. Unfortunately many patients eventually develop resistance to anti-TNF agents and remission fails. One reason for failure is that some patients develop antibodies to anti-TNFs so that the amount of drug in the patient's blood decreases below levels that are effective. Test kits have been developed and marketed that allow estimation of the levels of anti-TNF and of antibodies to anti-TNF in a patient's blood sample. This information can aid clinicians and patients to decide on the best course of future treatment, and may help avoid continued use of expensive but ineffective medicine. The present project aims to examine evidence about the clinical and cost effectiveness of test kits. The current report will allow NICE to make recommendations about how well the kits work and whether the benefits are worth the cost of the tests for use in the NHS in England and Wales. The assessment will consider both potential for improvement in patients' symptoms associated with use of the tests and the cost of the tests.

2. Decision problem

The current report being undertaken for the NICE Diagnostics Assessment Programme examines the clinical and cost effectiveness of ELISA tests (LISA-TRACKER EISA kits, TNFα-Blocker ELISA kits, and Promonitor ELISA kits) for measuring patient blood levels of anti-TNF agents (Infliximab and Adalimumab; also known as TNF inhibitors) and of antibodies to these agents (i.e., anti-drug antibody levels, ADAbs) in people with Crohn's disease whose disease responds to treatment with TNF inhibitor or who experience secondary loss of response during a maintenance course of TNF inhibitor therapy.

2.1 Anti-tumour necrosis factor alpha (anti-TNFα) agents

TNFα is a small cell-signalling protein (cytokine) involved in inflammatory responses primarily by influencing regulation of various effector cells of the immune system. TNFα has been shown to have

a role in several inflammatory diseases including Crohn's disease, ulcerative colitis, rheumatoid arthritis and ankylosing spondylitis. Therapies have been developed that are directed at blocking the actions of TNFα and thereby reducing inflammation. Such anti-tumour necrosis factor alpha (anti-TNFα) agents bind to cell surface TNFα and free TNFα and block its activity. Blocking of TNFα with anti-TNF drugs has been shown to successfully reduce the inflammation for some patients with inflammatory diseases including Crohn's disease. As these drugs are expensive and can cause potentially serious adverse effects, in England, they are generally used as second or third line treatment in the management of Crohn's disease and are employed when other drugs have not worked or have caused major side effects, and when surgery is not considered the appropriate treatment option. The anti-TNF agents recommended by NICE for the treatment of Crohn's disease are infliximab (Remicade®, Schering-Plough) and adalimumab (Humira®, Abbott Laboratories). These are monoclonal antibodies introduced into the human body to bind and block TNFα. They are classed as monoclonal antibodies because they are derived from genetically engineered immune cells, which are all daughters of a single parent cell, so that in culture they generate and secrete antibodies that are all of identical structure and affinity for TNFα.

2.1.1 Infliximab

Infliximab is a chimeric (mouse-human) monoclonal antibody. It is said to be chimeric because the genetic code determining its amino acid sequences is partly derived from the mouse genome and partly from the human genome. Infliximab belongs to the IgG1 (immunoglobulin gamma type 1) group of antibody molecules (Figure 1). It should be born in mind that IgG1 molecules are globular (not linear as in the diagram) and that they are glycoproteins that have carbohydrate chains attached (not shown in Figure 1). As infliximab is generated from cultured mouse cells, the carbohydrate part of the molecules corresponds to that of mouse rather than human glycoproteins.

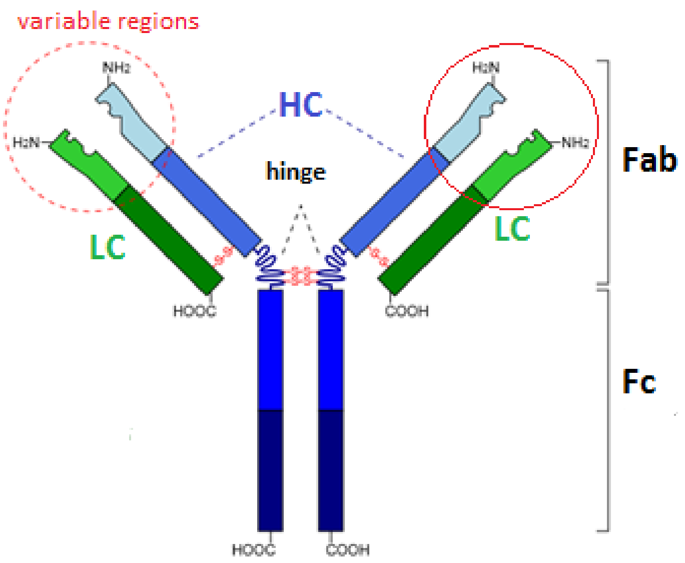

Figure 1. Diagrammatic representation of the structure of an IgG1 antibody molecule.
The molecule comprises two heavy chains (HC) and two light chains (LC); the HCs are joined together across disulphide bonds (S-S) and each LC is joined to a HC by S-S bonding. The LC and HC have a variable region (different from all other antibodies) at the amino (NH_2) end of the chain; these variable regions are responsible for binding antigen. The rest of the HC and LC are identical to other IgG1 antibodies and are called constant regions. Proteolytic enzymes papain and pepsin cut the molecule just above or below the S-S bonds holding the HC together. When below the HC S-S bond this generates an Fc (Fragment crystallising) and an Fab (Fragment antigen binding) product. When the split is above the HC S-S bond two antigen binding fragments are formed ($F(ab)_2$).

Infliximab is composed of human IgG1 heavy chain constant regions and human Kappa light chain constant regions (together representing 70% of the genetic makeup of the molecule), plus mouse-derived heavy chain and light chain variable regions (30% of the genetic makeup, 4 out of 12 domains) which carry the binding sites with high affinity and specificity to TNFα (Figure 1). Infliximab was the first anti-TNF agent that was approved and licenced for treating severe active Crohn's disease and active fistulising Crohn's disease in adults and children over the age of six. It is administered intravenously over 1–2 hours. Details of the licenced indication are given in Appendix 1.

Side effects of infliximab include:

- Allergic reaction to the infusion (or infliximab) apparent by:
    - hives (red, raised, itchy patches of skin) or other skin rashes
    - difficulty swallowing or breathing
    - pains in the chest or muscle or joint pain fever or chills
    - swelling of the face or hands
    - headaches or a sore throat
- Serious viral or bacterial infections including tuberculosis, especially in people over 65
- Skin reactions including psoriasis (red scaly patches), rashes, skin lesions, ulcers and hives, and swollen face and lips
- Worsening of heart problems
- Increased risk of cancer or lymphoma
- Liver inflammation

Many of the side effects are reversible if the drug is stopped.

2.1.2 Adalimumab

Adalimumab is a human IgG1 monoclonal antibody with Kappa light chains. It consists of purely human antibody polypeptide domains (Figure 1). However, as adalimumab is generated from cultured Chinese hamster ovary cells, the carbohydrate part of the molecules corresponds to that of hamster rather than human glycoproteins. Adalimumab is a more recent anti-TNFα therapy that was approved for treating Crohn’s disease in adults only. It is administered as a subcutaneous injection by a doctor or nurse or can be self-injected by the patient or a family member. Details of the licenced indication are given in Appendix 1.

Side effects of adalimumab include:

- Reactions to the injection including pain, swelling, redness, bruising and itching
- Allergic reaction to adalimumab including:
    - rashes or hives
    - swollen face, hands and feet
    - trouble breathing
- Greater susceptibility to infections such as colds, flu, pneumonia, sepsis and tuberculosis
- Skin reactions including psoriasis (scaly patches), eczema, other skin rashes and ulcers
- Skin cancer, lymphoma or leukaemia
- Damage to nerves (demyelination)
- Lupus

Many of the side effects are reversible if the drug is stopped.

2.2 Intervention technologies

The intervention technologies are the LISA-TRACKER ELISA kits (Theradiag / Alpha Laboratories), the TNFα-Blocker ELISA kits (Immundiagnostik AG), and the Promonitor ELISA kits (Proteomika). They estimate the following molecules in patient blood sera:

- Infliximab
- Adalimumab
- Anti-infliximab antibodies
- Anti-adalimumab antibodies

2.2.1 Anti-TNF monitoring using assays to measure the levels of anti-tumour necrosis factor-alpha agents (anti-TNFα drugs) and the anti-drug antibodies (ADAb) in the blood plasma or serum

Rationale

In some patients an initial or maintained response to anti-TNF therapy may disappear. This has been observed for all conditions in which these therapies have been used. The reasons for response failure may be various and are not fully understood, however loss of response has often been found to be associated with the generation of immune responses to the anti-TNF agent itself. In particular the patient may generate antibodies directed against the anti-TNF agent, these will bind to the administered anti-TNF agent, nullify its effectiveness and hasten its clearance from the circulation. These effects may explain or partially explain the phenomena of loss of response experienced by some patients. The generation of antibodies against infliximab may not be surprising since about 30% of the molecule has mouse identity. Adalimumab, although termed a fully humanised antibody, has potential to be antigenic since its carbohydrate moieties are mouse derived and because its binding site for anti-TNF is unique and could, according to the network hypothesis of Jerne,[1] lead to generation of antibodies directed against this "idiotypic" region of the drug.

Other patients may respond well to an induction phase of treatment with a TNF inhibitor. However, these patients may lose response in the future, may benefit from optimising dosing or may require review after 12 months of treatment with a TNF inhibitor. Management of responders could benefit from knowing levels of anti-TNF drug and anti-drug antibodies in the patients' blood.

Manufacturers and others have developed various assay procedures for anti-TNF agents and for anti-drug antibodies (ADAbs) in the belief that the levels of circulating anti-TNF and of ADAbs can provide information useful to clinicians in indicating potential reasons for treatment failure, and for dosage or treatment adjustment. The LISA-TRACKER, TNFα-Blocker, and Promonitor are particular examples of these assays and are classified as solid phase Enzyme Linked Immunosorbent Assays (ELISA assays). Other methodologies based on alternative principles of detection and measurement include: [a] radioimmunoassays; liquid phase assays [b] cell reporter assays based on genetically engineered cells incubated in culture medium; [c] mobility shift assays; liquid phase assays using size-exclusion HPLC and fluorescent dye detection. Brief descriptions of the assay methods follow.

ELISAs for infliximab and adalimumab

All three ELISA methods employ similar principles in which, typically, micro-titre plates with 96 wells coated with reagent receive the patient serum samples or various standards and calibrators. Reagents are added with wash steps between additions. The final step involves quantifying the

amount of a peroxidase label in the titre well, this amount being proportional to the amount of anti-TNF or ADAb in the patient's sample or in the calibrator standard.

The amount of peroxidase present in the well is quantified using a timed incubation with excess substrates (hydrogen peroxide + 3,3',5,5'-tetramethylbenzidine). Peroxidase catalyses the following reaction: Tetramethylbenzidine + hydrogen peroxide → chromogen + water
The incubation is stopped after an appropriate time by the addition of acid and the accumulated chromogen quantified by measuring optical density with a spectrophotometer.

The reagents used for coating the microtitre plate wells and the reagents used in subsequent steps of the assay procedure differ from each other according to manufacturer. The LISA-TRACKER assays for Infliximab and for Adalimumab are illustrated in Figure 2.

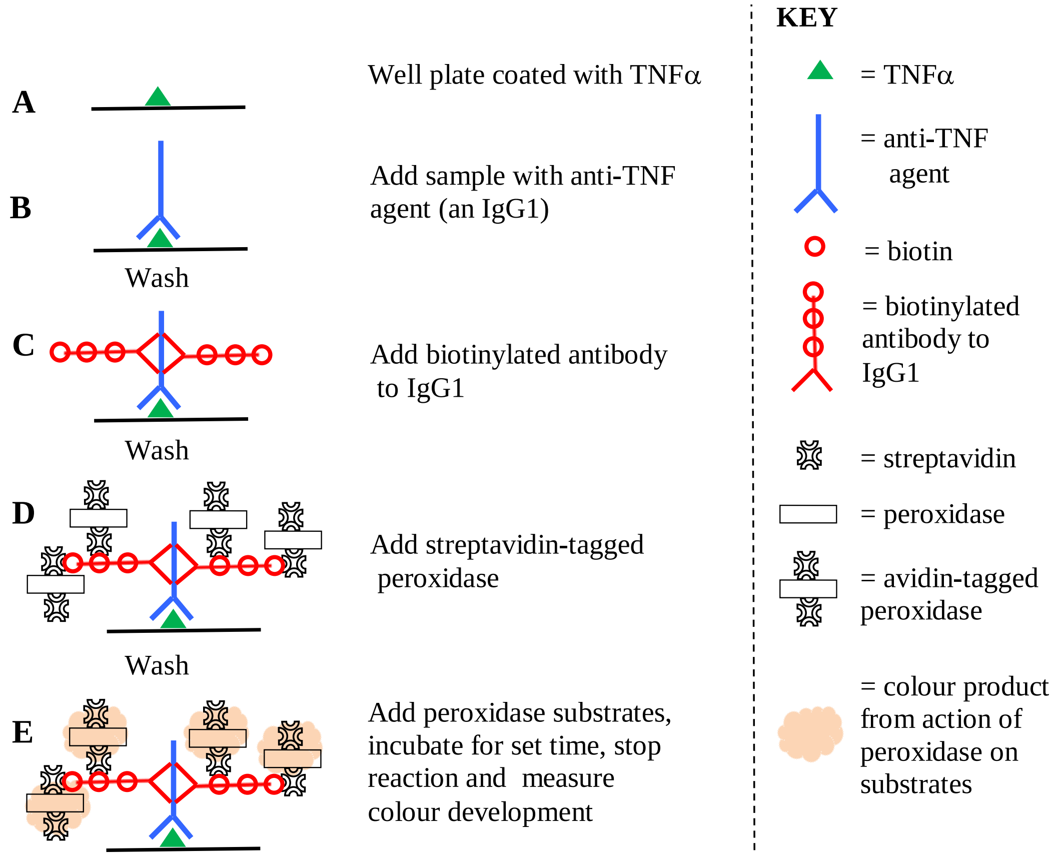

Figure 2. Diagrammatic representation of the LISA-TRACKER assay for infliximab and Adalimumab

Procedural steps C and D are detection steps that function to detect the anti-TNF that is bound to the well surface via TNFα, ensuring a quantitative relationship between anti-TNF and peroxidase. Step E quantifies the amount of peroxidase (and therefore anti-TNF) in the titre well (note: Streptavidin has four very high affinity binding sites for biotin).

Serum samples from patients may contain soluble TNFα receptors; these could compete with anti-TNF for the immobilised TNFα on the well plate and may potentially interfere with the assay. The assay quantifies free anti-TNF. Samples may contain anti-TNF bound to antibodies to anti-TNF, especially in patients who have lost a response to treatment. These anti-TNF-antibody complexes will be washed away at the first wash step leaving only free anti-TNF bound to immobilised TNFα. The amount of anti-TNF lost at the wash step is likely to vary between patients and is unknown; the practical implications of this are uncertain.

TNFα-Blocker and Promonitor differ from LISA-TRACKER in employing a single step and one reagent for detecting well-bound anti-TNF, rather than two steps (C and D in Figure 2) and two reagents. Table 1 summarises the information currently available describing the principle of these assays.

Table 1. Summary of ELISAs to be considered in this review for detection of infliximab and adalimumab

**Manufacturer (Kit)**	**Microplate pre-coat**	**Detection reagent(s)**	
LISA-TRACKER	TNFα	Biotinylated IgG1 antibody	Avidin-tagged peroxidase
TNFα-Blocker ELISA	Monoclonal anti-TNF antibody	Peroxidase labelled antibody	
Proteomika ELISA	Monoclonal anti-TNF antibody	Peroxidase labelled monoclonal anti-TNF antibody	

<u>ELISAs for anti-drug antibodies (ADAbs)</u>
These are available as commercial kits and several “in house” methods are mentioned in the literature. The majority of ELISAs only quantitatively measure “free” anti-TNF and “free” ADAbs and it is acknowledged that the level of the unmeasured “bound” anti-TNF and of “bound” ADAb may vary considerably between patients. The Immundiagnostik assays give semi-quantitative measurement of ‘total’ ADAbs. Thus for some patient samples there is an unknown and unmeasured amount of anti-TNF and of ADAb present, in addition to the measured “free” levels.

Below the LISA-TRACKER methods are reported and differences to TNFα-Blocker and Promonitor are described. The LISA-TRACKER assays for antibodies to infliximab and to adalimumab are illustrated in Figure 3.

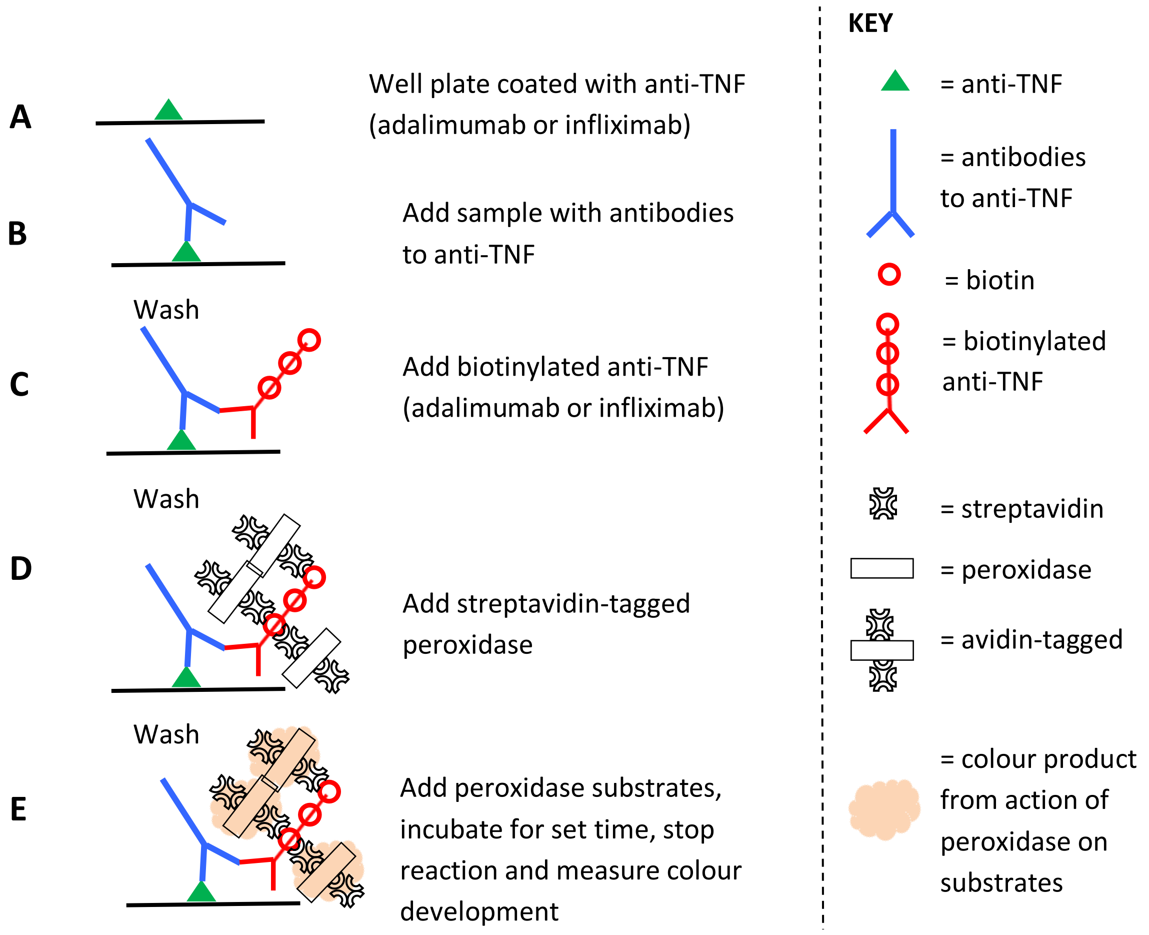

Figure 3. Diagrammatic representation of the LISA-TRACKER assay for antibodies to infliximab or to adalimumab.

Procedural steps C and D are detection steps that function to detect the sample antibodies, ensuring a quantitative relationship between anti-TNF antibodies and peroxidase. Step E quantifies the amount of peroxidase (and therefore anti-TNF antibodies) (note: Streptavidin has four very high affinity binding sites for biotin).

This assay only quantitatively estimates free antibodies to anti-TNF. Thus ADAbs bound to the drug are lost at the first wash. The amount of bound ADAb is likely to vary between patients and is unknown. Whether ADAbs directed at non-idiotypic regions of the drugs (e.g., glycoprotein moieties, variable non-idiotypic mouse regions of infliximab etc.) are detectable or present in samples appears to be uncertain.

TNFα-Blocker and Promonitor differ from LISA-TRACKER in employing a single step and reagent for detecting well-bound anti-TNF rather than two steps (C and D in Figure 2) and two reagents. Table 2 summarises the information currently available describing the principle of these assays.

Table 2. Summary of ELISAs to be considered in this review for detection of antibodies to infliximab and adalimumab

**Manufacturer (Kit)**	**Microplate pre-coat**	**Detection reagent(s)**	
LISA-TRACKER	Anti-TNF	Biotinylated anti-TNF	Avidin-tagged peroxidase
TNFα-Blocker ELISA infliximab	Infliximab F(ab)2	Peroxidase labelled infliximab	
TNFα-Blocker ELISA adalimumab	Adalimumab F(ab)2	Peroxidase labelled adalimumab	
Proteomika ELISA	Anti-TNF	Peroxidase labelled anti-TNF	

Brief overview of identified non-ELISA assay methods

There are no "gold standard" assays for measuring anti-TNF agents or for antibodies to anti-TNF agents which might provide a robust basis for comparisons between the performance of different assays. According to the US Medical Insurance assessments "candidate" gold standards have been insufficiently investigated to establish any as a gold standard, and according to Steenholdt et al. (2013)[2] it is unknown if and how these different assays compare.[3-7]

There appear to be four types of assay for measuring the levels of anti-TNF drugs and the levels of antibodies against TNF inhibitors in patient blood sera. which differ fundamentally from each other. In addition to ELISAs (solid phase assays) these are:

(a) Radioimmunoassays (RIA) – liquid phase. They appear to measure total anti-TNF and total ADAb (probably as long as the ADAb light chain is lambda class). These RIAs use 125 iodine-labelled human TNFα and 125 iodine-labelled anti-TNFs. In these assays the patient's sample is mixed with a solution containing a fixed amount of 125 iodine-labelled TNFα or 125 iodine-labelled anti-TNF further antibody (e.g., rabbit anti-human immunoglobulin λ-chain) which promotes the formation of immune complexes which are pelleted by centrifugation. Radio-iodine in the pellet is quantified in a gamma-counter. Characteristics of these assays include: i) radio-labelled reagents do not store indefinitely (125 iodine decays with a half-life of 59 days), ii) the laboratory needs to be equipped for handling hazardous (radioactive) material, iii) some staff training may be necessary, and iv) the laboratory requires a gamma counter (preferably automated for high throughput).

(b) Cell Reporter Assays. The reporter cells are genetically engineered to contain genes for two light producing enzymes "*luciferases*" (one from the firefly which can generate red light, and one from the sea pansy which can generate blue light). The firefly gene is under the control of a TNFα signalling

pathway so that when the cells are incubated in the presence of TNFα they synthesise the enzyme, after a standard incubation time appropriate substrates for the enzyme are added and the emitted red light measured with a luminometer. If anti-TNF is present the TNFα response is partially quenched and the quenching estimated. If ADAb is present, quenching by anti-TNF is reduced and this can be measured. The sea pansy gene is expressed during incubation after which appropriate substrates are added and the blue light emitted measured in the luminometer. The usefulness of the blue light measure is that it allows "normalisation" of the red light emission as interfering agents in patient blood samples equally affect both firefly and sea pansy systems. Requirements in addition to appropriate cell reporter cultures and reagents include requirement for a luminometer (although these are not necessarily routinely available) and equipment for culture of growth arrested genetically engineered cells under controlled conditions (oxygen, CO_2, humidity).

(c) The Mobility Shift Assay is a liquid phase assay based on size exclusion HPLC (SE-HPLC) which separates free probe (small size) from probe in an immune-complex (large size). The ADAb assays use fluorescent-dye-labelled anti-TNF (D*) as the probe. In the presence of antibodies to anti-TNF some D* form immune complexes with these (D*-ADAb complexes) and will exhibit a mobility shift on the SE-HPLC column relative to the D* which remains free. The amount of D* shifted to greater mobility is proportional to the amount of ADAb present. The amount of dye (*) present in the eluent stream coming from the HPLC column at different mobilities is measured with a fluorimeter.

The anti-TNF assay uses fluorescent-dye-labelled TNFα (TNF*) as the probe; in the presence of anti-TNF some TNF* forms immune-complexes with the anti-TNF and these have greater mobility on the SE-HPLC than the free TNF*. The amount of TNF* shifted to greater mobility is proportional to the amount of anti-TNF present. The amount of dye (*) present in the eluent stream coming from the HPLC column at different mobilities is measured with a fluorimeter.

In measuring ADAb the patient sample is subjected to an acid step which "unbinds" bound anti-TNF and ADAb so that all anti-TNF and ADAb are "free"; after neutralisation the sample is incubated with fluorescent-dye-labelled anti-TNF (D*) as described above. Some D* will form immune complexes with the sample ADAbs (D*-ADAb complexes) and these have a different mobility on SE-HPLC than D* thus the mobility of some of the D* is shifted, the proportion of D* shifted is dependent on the level of ADAb in the sample.

2.3 Timing and use of ELISAs

Scoping searches indicate that the anti-TNF and ADAb assays are most frequently administered just before the next administration of the anti-TNF agent. This is said to allow measurement of a "trough" level of anti-TNF and may have been adopted when ELISAs are used so as to minimise effects from

the presence of anti-TNF-ADAb immune-complexes in samples. For patients whose response to therapy has waned, the results of the tests are frequently dichotomised using a cut off assay result. Thus, on the basis of anti-TNF assays patients are classified as having therapeutic levels of anti-TNF or sub-therapeutic levels, and on the basis of ADAb assay results they are classified as having clinically significant levels of ADAbs or insignificant levels. Such classifications yield four categories of patient for whom different explanations of failed response are possible. Algorithms have been developed prescribing treatment pathways and / or further diagnostic tests (e.g., colonoscopy) based on such classification.

2.4 Target condition / indication

Anti-TNFα is commonly given to people with inflammatory bowel disease (IBD) including Crohn's disease. The general background and treatment pathway for Crohn's disease is summarised below.

2.4.1 Crohn's disease

Crohn's disease is a chronic fluctuating episodic inflammatory condition of the digestive tract; it is uncommon and is currently estimated to affect about 115,000 people in the UK.[8] Together with ulcerative colitis it comprises conditions classed as inflammatory bowel disease (IBD).

Aetiology and pathology

Crohn's disease can affect adults, adolescents or children. Crohn's disease manifests itself mainly during late adolescence or early adulthood. The first onset most commonly occurs between the ages of 16 and 30 with a second peak between the ages of 60 and 80. Women are slightly more frequently affected than men but in children it is seen more often in boys than in girls. The condition has highest prevalence among Jewish people with European descent.

Crohn's disease follows a pattern of acute disease interspersed with periods of remission. Crohn's disease causes inflammation of the lining of the digestive tract which, depending on the individual, occurs at any location from the mouth to the rectum, but most commonly affects the terminal ileum (35%) or the ileocaecal region (40%). Within individuals the disease location is fairly stable.

The main symptoms of Crohn's disease are dependent on disease location and include chronic or nocturnal diarrhoea, abdominal pain, anal lesions, rectal bleeding and weight loss. Clinical signs include pallor, cachexia, abdominal mass or tenderness, or perianal fissures, fistulas or abscesses. Systemic symptoms include malaise, anorexia or fever.[9-11] Extra-intestinal symptoms related to intestinal inflammation include spondyloarthritis (inflammatory rheumatic diseases which cause arthritis, most commonly ankylosing spondylitis), cutaneous manifestations or ocular inflammation.[11] In children, growth failure may be the primary manifestation of Crohn's disease.[12]

Classification of Crohn's disease disease states and measurement of disease activity

Several classification systems of Crohn's disease have been proposed. The Montreal[13] and Vienna[14] systems are summarised in Tables 3 and 4.

Table 3. Montreal classification of Crohn's disease

Age at diagnosis	Location	Behaviour
A1: <16 years	L1: Ileal	B1: Inflammatory
A2: 17-40 years	L2: Colonic	B2: Stricturing
A3: >40 years	L3: Ileocolonic	B3: Penetrating
	L4: Upper GI disease	P: Perianal disease

Table 4. Vienna classification of Crohn's disease

Age at diagnosis	Location	Behaviour
A1: <40 years of age	L1: Terminal ileum - limited to terminal ileum, with or without spill-over into the caecum	B1: Non-stricturing, non-penetrating
A2: ≥40 years of age	L2: Colon - any colonic location between the caecum and rectum, with no small bowel or upper GI involvement	B2: Stricturing - constant luminal narrowing demonstrated by radiological, endoscopic, or surgical-pathological methods, with pre-stenotic dilation or obstructive signs/symptoms, without the presence of penetrating disease, at any time in the course of the disease
	L3: Ileocolonic - disease of ileum and any location between the ascending colon and rectum	B3: Penetrating - occurrence of intra-abdominal or perianal fistulae, inflammatory masses, and/or abscesses at any time in the course of the disease. Perianal ulcers are included. Postoperative intra-abdominal complications and skin tags are excluded
	L4: Upper GI - any disease proximal to the terminal ileum (excluding mouth), regardless of additional involvement of the terminal ileum or colon	

"The severity of Crohn's disease is difficult to assess, and a global measure encompassing clinical, endoscopic, biochemical and pathological features is not available.[15] The most widely used disease activity measures include the Crohn's Disease Activity Index (CDAI), the Harvey-Bradshaw Index (HBI) or Simple Index (a simplified version of the CDAI), and the Perianal Disease Activity Index

(PDAI). A commonly used health related quality of life measure is the Inflammatory Bowel Disease questionnaire (IBDQ). Other measures include the Crohn's Disease Endoscopic Index of Severity (CDEIS).

The CDAI was developed in the 1970s when a need for a single index to assess disease severity was recognised. Variables measured include number of liquid stools, abdominal pain, general well-being, extra-intestinal complications, use of anti-diarrhoeal drugs, abdominal mass, haematocrit and body weight; scores range from 0 to approximately 600 (see Appendix 2 for a description of the index and the scoring system used). Values of below 150 are suggestive of quiescent disease (remission) and values above 450 are associated with very severe disease.[16] Some investigators have arbitrarily labelled CDAI scores of 150-219 as mildly active disease and scores of 220 to 450 as moderately active disease.[15]

The CDAI has been criticised for having limitations since it fails to encompass aspects of quality of life such as psychological, social, sexual wellbeing and occupational functioning. A patient with a low CDAI score may still be severely limited by these factors.[17] Substantial variability exists when different observers review the same case histories and calculate the CDAI score, although this can be reduced after discussion and education about the terminology. The calculation is based in part on a daily diary kept by the patient for seven days before the evaluation. In practice some investigators and study coordinators assist the patient to complete the diary retrospectively at the time of an evaluation visit; there is no information on the prevalence of this practice. The CDAI score may be low in patients whose primary symptom is drainage of enterocutaneous fistulas, presumably because the presence of an actively draining fistula contributes only 20 points to the score. The CDAI is therefore not an appropriate instrument for assessing the activity of draining abdominal or perianal enterocutaneous fistulas. The CDAI has been criticised for giving too much weight to 'general well-being' and 'intensity of abdominal pain' because these are relatively subjective items. However these aspects of disease are important to patients.[18] A paediatric CDAI has been developed.[18, 19]

The HBI or Simple Index is a modified/simplified version of the adult CDAI. It uses a single day's reading for diary entries and excludes three variables (body weight, haematocrit and use of drugs for diarrhoea). Code values are added together rather than summing the products of code values and coefficients. Scores range from 0 to 20. The CDAI can be predicted reasonably well from the HBI.[20] Other instruments derived from the CDAI are: the Cape Town Index (CTI), which includes parameters on subjective symptoms, physician clinical findings and laboratory data; the three-variable version of the CDAI used for survey research; and the Van Hees Index (VHI), which includes laboratory parameters, sex (male or female) and seven clinical features and excludes subjective patient related items such as well-being and pain.

The PDAI was developed to account for the morbidity and impairment of quality of life of patients with perianal disease, and to evaluate the effectiveness of perianal disease treatment. Variables include discharge, pain/restriction of activities, restriction of sexual activity, type of perianal disease (including number of fistulas) and degree of induration. Scores range from 0 to 20.[21]

The reliance on traditional disease activity measures (such as the CDAI) to measure treatment effectiveness fails to take into account the impaired quality of life experienced by Crohn's disease patients. The IBDQ is a 32 item health related quality of life measure. The questionnaire evaluates general activities of daily living, intestinal function, social performance, personal interactions and emotional status. Four-dimensional scores cluster items under bowel function, emotional function, systemic function and social function. Scores range from 32 to 224.[22]

The CDEIS was developed to take into account endoscopic data, such as lesion severity, when assessing severity of the disease. Variables include the presence or absence of deep or superficial ulceration in various segments of the intestinal tract, the surface involved (in cm), surface ulcerated (in cm) and presence of ulcerated stenosis. Scores range from 0 to 30.[23]

Clinical studies have variously defined a clinical response as a decrease in CDAI score of 50, 60, 70 or 100 points. In 2000 the FDA and EMEA suggested that a meaningful decrease in the CDAI score is a decrease of 100 points.[18]"{#19}

Working definitions of disease severity have been developed by the Practice Parameters Committee of the American College of Gastroenterology (2001).[11] These are:-

Mild-moderate disease:

- *"Mild-moderate disease applies to ambulatory patients able to tolerate oral alimentation without manifestations of dehydration, toxicity (high fevers, rigors, prostration), abdominal tenderness, painful mass, obstruction, or >10% weight loss"*

Moderate-severe disease:

- *"Moderate-severe disease applies to patients who have failed to respond to treatment for mild-moderate disease or those with more prominent symptoms of fever, significant weight loss, abdominal pain or tenderness, intermittent nausea or vomiting (without obstructive findings), or significant anaemia."*

Severe-fulminant disease:

- *"Severe-fulminant disease refers to patients with persisting symptoms despite the introduction of steroids as outpatients, or individuals presenting with high fever, persistent vomiting, evidence of intestinal obstruction, rebound tenderness, cachexia, or evidence of an abscess."*

Remission:

- *"Remission" refers to patients who are asymptomatic or without inflammatory sequelae and includes patients who have responded to acute medical intervention or have undergone surgical resection without gross evidence of residual disease. Patients requiring steroids to maintain well-being are considered to be 'steroid-dependent' and are usually not considered to be 'in remission'."*

Anti-TNF monitoring in Crohn's disease

Crohn's disease is associated with elevated levels of the immune-regulatory protein TNFα. The reasons for this elevation in Crohn's disease is still largely unknown. Anti-TNF therapies have been shown to block the action of TNFα and to improve outcomes for some patients. Patients receive anti-TNF therapy after failed attempts to improve the condition with first line glucocorticosteroids, 5-aminosalicylates, antibiotics and second line treatment (e.g., methothrexate). These patients have severe symptoms and they are at the end of the patient pathway with the only alternative option being surgery.

Like other treatment regimens anti-TNF treatment aims to induce remission (induction therapy) and prevent relapse (maintenance therapy). However failure to induce a response and relapse or loss of response are common. Approximately 10% of patients per year loose response to anti-TNF drugs.[24] The annual risk of response loss per patient has been estimated at about 13%.[25] During "episodic" infliximab therapy about 37-61% lose response.[26] Mechanisms of loss of response to anti-TNF agents and of failure to respond are still mainly unclear, however the fact that some patients generate immune responses to therapy offers one plausible contributory explanation. However other pharmacodynamics mechanisms may reduce the drug below therapeutic levels, furthermore there may be alternative secondary pathways of inflammation independent of TNFα that operate in some patients rendering anti-TNF of little use.

During scheduled infliximab therapy the incidence of antibodies is 6-16%.[27, 28] Anti-TNF antibody formation in patients treated with Infliximab has been shown to be as high as 37-61%.[29] Concomitant immunosuppressive therapy may decrease the formation of ADAbs.[26, 27, 29] Candidate risk factors for ADAb production include hereditary predisposition, a dysfunctional immune system, experience of infection(s) that trigger an abnormal response, smoking, environmental factors such as sanitation.

The ELISA assays could be used in good responders (i.e., those responding to initial induction course of anti-TNF treatment) as well as in patients with secondary loss of response (i.e., those initially responding to anti-TNF treatment but loosing this response over time). The use of these technologies provides a clinician with potentially useful information that may guide individual patient's future treatment. Such information may aid in anticipating the loss of response in responders, while for non-responders such analyses may help in estimating the likelihood of various candidate reasons for primary non-response or secondary loss of response. For example in non-responders with low levels of drug and high levels of ADAbs the loss or lack of response may be surmised to be due to rapid clearance of the drug due to action of ADAbs; on the other hand a low level of anti-TNF in the absence of ADAbs may be suggestive of non-immune mechanisms of rapid drug clearance, while high levels of drug in absence of antibodies in non-responders may be suggestive of a TNFα-independent pathology for the condition in a particular patient. Algorithms for future treatment based on anti-TNF and ADAb estimates have been published.

In theory the application of the tests in conjunction with an appropriate algorithm for treatment based on test results:

- May improve quality of life and other outcomes (e.g., faster healing of flare-ups, reduced abdominal pain and associated diarrhoea)
- May optimise the treatment plan (facilitate adoption of the most suitable future treatment for individual patients; this might involve a switch to an alternative anti-TNF or a biologic with an alternative mechanism of action)
- May minimise the risk of drug overdose and associated adverse events
- May allow earlier de-escalation of therapy, leading to a reduction in the overall drug used
- May help to reduce the amount of drugs used inappropriately, unnecessary hospital visits, risk of surgery, and associated costs

Crohn's disease: Management and Care pathway

The treatment of Crohn's disease is complex, which in general aims at: a) reducing symptoms through induction and maintenance of remission, b) minimising drug-related toxicity, and 3) reducing the risk of surgery. The management options for Crohn's disease include drug therapy (e.g., glucocorticosteroids, 5-aminosalicylate, antibiotics, immunosuppressives, TNFα inhibitors), enteral nutrition, smoking cessation and, in severe or chronic active disease, surgery (Table 5). The choice of treatment amongst the available drugs is influenced by patient age, site and activity of disease, previous drug tolerance and response to treatment, and the presence of extra-intestinal manifestations.[30, 31] Enteral nutrition is widely used as a first line treatment to facilitate growth and development in children and young people. Adjuvant therapy commonly coexists and includes

management of extra-intestinal manifestations, antibiotics, corticosteroids or immunomodulator therapy. Between 50% and 80% of people with Crohn's disease require surgery due to complications such as strictures causing symptoms of obstruction, fistula formation, perforation or failure of medical therapy.[32]

Once remission has been achieved, maintenance therapy can be considered following assessment of the course and extent of Crohn's disease, effectiveness and tolerance of previous treatments, presence of biological or endoscopic signs of inflammation, and potential for complications.

Table 5. Treatment options for patients with Crohn's disease[33]

Patient group	Treatment Line and Treatment
**Ileocaecal disease not fistulating with <100 cm of bowel affected: initial presentation or relapse**	
• mildly active	1st observation with monitoring or budesonide or 5-ASA therapy
• moderately active: initial presentation or non-corticosteroid-dependent/-refractory relapse	1st budesonide and/or 5-ASA therapy, or conventional oral corticosteroids (use previously effective treatment for relapse)
	2nd immunomodulator therapy + oral corticosteroid taper
	3rd anti-TNF therapy + oral corticosteroid taper
• moderately active: relapse corticosteroid-dependent/-refractory	1st consideration of early initiation of anti-TNF therapies + oral corticosteroid taper
	2nd surgery
• severely active: initial presentation or non-corticosteroid-dependent/-refractory relapse	1st hospitalisation + oral or intravenous conventional corticosteroids + consideration of surgery
	2nd anti-TNF therapy or surgery
• severely active: relapse corticosteroid-dependent/-refractory	1st hospitalisation + consideration of early initiation of anti-TNF therapy or surgery
**Colonic disease not fistulating: initial presentation or relapse**	
• mildly active	1st 5-ASA therapy or alternatively oral corticosteroids
	2nd surgery
• moderately or severely active:	1st oral or intravenous corticosteroids + immunomodulator therapy + consideration for surgery

initial presentation or non-corticosteroid-dependent/-refractory relapse	2nd anti-TNF therapy + consideration for surgery
	3rd surgery
• moderately or severely active: relapse corticosteroid-dependent/-refractory	1st early initiation of anti-TNF therapy or consideration for surgery
	2nd surgery
**Extensive small bowel disease (>100 cm of bowel affected) not fistulating: initial presentation or relapse**	1st oral corticosteroids + early introduction of immunomodulators
**Upper GI disease (oesophageal and/or gastroduodenal disease) not fistulating: initial presentation or relapse**	1st proton pump inhibitor
**Perianal or fistulating disease: initial presentation or relapse**	
• simple perianal fistula: symptomatic	1st loose seton + drainage of perianal abscess if present
• complex perianal fistulae	1st loose seton placement + drainage of perianal abscess if present
• non-perianal fistulae	1st multidisciplinary input + supportive care

Abbreviations: 5-ASA 5-Aminosalicylic Acid, TNF tumour necrosis factor, GI gastrointestinal

Induction of remission

Usually, at first presentation, people with active Crohn’s disease are recommended monotherapy with a conventional glucocorticosteroid (prednisolone, methylprednisolone or intravenous hydrocortisone), which is aimed at inducing remission as a first line treatment. Alternatively, treatment with budesonide, 5-ASA, or enteral nutrition may be offered to a group of people who do not choose to take or who are intolerant to glucocorticosteroid therapy.

The addition of an immunosuppressant (azathioprine, mercaptopurine or methotrexate) to a conventional glucocorticosteroid or budesonide as an add-on therapy for inducing remission is recommended for people who have active Crohn’s disease and have experienced two or more inflammatory exacerbations in a 12-month period, or in whom the glucocorticosteroid dose cannot be tapered. As advised in the current online version of the British national formulary (BNF)[34] or British National Formulary for Children (BNFC),[34] the effects of azathioprine, mercaptopurine, and methotrexate as well as levels of neutropenia (in people on azathioprine or mercaptopurine) should be monitored.

Adults with severe active Crohn's disease who fail to respond to the first line of treatment with conventional therapy (e.g., immunosuppressive drugs, corticosteroids), or who are intolerant of or have contraindications to the above-mentioned conventional therapy, anti-TNF alpha agents (infliximab and adalimumab) are recommended as treatment options within their licensed indications. The administration of anti TNF alpha agents is recommended until 12 months after the start of treatment or until treatment failure (including the need for surgery), depending on whichever occurs first. Periodic reassessment and monitoring of disease activity (at least every 12 months) is advised in order to ascertain the clinical appropriateness of ongoing treatment. Usually, treatment course needs to be initiated with the less expensive drug by considering drug administration costs, dose, and product price per dose. The use of anti-TNF-alpha drugs for the treatment of Crohn's disease is covered in the 2010 NICE technology appraisal guidance 187 (Infliximab (review) and adalimumab for the treatment of Crohn's disease).[35]

Surgery should be considered as an alternative to medical treatment early in the course of the disease for people (adults, children, and young people) whose disease is limited to the distal ileum or have growth impairment despite optimal medical treatment and/or refractory disease (children and young people).

<u>Maintenance of remission</u>

People with Crohn's disease in remission can be managed with or without maintenance treatment. The options for maintenance therapy (including treatment or no treatment) need to be discussed with patients, their parents, and/or carers. The discussion should include risk of inflammatory exacerbations (with and without drug treatment) and the potential side effects of drug treatment. People who decline to receive maintenance treatment should agree with follow-up plans (e.g., frequency and duration of visits) and receive information on symptoms related to relapse (e.g., unintended weight loss, abdominal pain, diarrhoea, general ill-health) to ensure timely consultations with their healthcare professional.

People with Crohn's disease in remission who choose to receive maintenance therapy may be offered azathioprine or mercaptopurine monotherapy if their remission was induced using a conventional glucocorticosteroid or budesonide. Methotrexate can be offered to people whose remission was induced by methotrexate or people who did not tolerate azathioprine or mercaptopurine for maintenance therapy or those who have contraindications to azathioprine or mercaptopurine. Treatment with 5-ASA can be recommended to maintain remission after surgery.

If remission has been achieved with anti-TNF medication, then maintenance with anti-TNF with or without combination with another immunomodulator can be recommended. Continuation of treatment with infliximab or adalimumab during remission is advised only if there is evidence of ongoing active disease given clinical symptoms, biological markers, including endoscopy if necessary. The balance between harms and benefits of ongoing treatment should be taken into account. People who relapse after treatment is stopped have the option to start this treatment again.

3 Decision questions and objectives

3.1 Decision questions

The decision questions for this project are shown in the box below:

1. *Does concurrent testing of TNF inhibitor levels and antibodies to TNF inhibitors represent a clinically and cost-effective use of NHS resources in people with Crohn's disease whose disease responds to treatment with TNF inhibitor?*
Testing will be carried out:
a) 3 to 4 months after start of treatment or
b) 3 to 4 months and every 12 months from start of treatment

2. *Does concurrent testing of TNF inhibitor levels and antibodies to TNF inhibitors represent a clinically and cost-effective use of NHS resources in people with Crohn's disease who experience secondary loss of response during maintenance treatment with TNF inhibitor?*

3. *Does testing of TNF inhibitor levels followed by reflex testing of antibodies to TNF inhibitors if drug level is undetectable represent a clinically and cost-effective use of NHS resources in people with Crohn's disease whose disease responds to treatment with TNF inhibitor?*
Testing will be carried out:
a) 3 to 4 months after start of treatment or
b) 3 to 4 months and every 12 months from start of treatment

4. *Does testing of TNF inhibitor levels followed by reflex testing of antibodies to TNF inhibitors if drug level is undetectable represent a clinically and cost-effective use of NHS resources in people with Crohn's disease who experience secondary loss of response during maintenance treatment with TNF inhibitor?*

3.2 Objectives

Given these decision questions the four main objectives for this report are:

A) To provide a technical description, and (where evidence allows) an evaluation, of the listed intervention tests used for Crohn's disease in therapeutic monitoring of TNF inhibitors (infliximab and adalimumab) and their respective antibodies. This will include what the assays measure and the mechanisms of the assays.

In addition, published studies which include a comparison (including relative test performance) of two or more intervention tests, or which compare an intervention test with a test method which can be used to perform a linked evidence assessment will be reviewed and critiqued. Data submitted by the manufacturers will be used to supplement published studies if deemed of sufficient detail and quality.

B) To describe algorithms used in studies which include data on one or more intervention test or on a test which allows a linked evidence approach to be performed (i.e., algorithms used in studies identified in Objective C). The studies are required to provide an algorithm and report clinical outcomes for the management of patients with Crohn's disease following measurement of serum levels of anti-TNF drug and anti-drug antibodies. To compare the algorithms used following therapeutic drug monitoring to the algorithms specified in the TAXIT study for responders,[36] and in the reporting of secondary loss of response (algorithm adapted from the study by Scott and Lichtenstein, 2014[37]).

C) To systematically review the literature comparing the clinical effectiveness of [a] the intervention assays for anti-TNF agents and/ or for ADAbs used in conjunction with a treatment algorithm in Crohn's patients treated with infliximab or adalimumab; with [b] standard care (no tests performed or test-informed algorithm used) in Crohn's disease patients treated with infliximab or adalimumab. Where evidence exists on the comparison of standard care with other test assays used in conjunction with an algorithm, this will be assessed and critiqued and test performance will be compared with that of the study interventions (LISA-TRACKER ELISA kits, TNFα-Blocker ELISA kits, and Promonitor ELISA kits) (see Objective A).

D) To assess the cost-effectiveness of employing anti-TNF monitoring with LISA-TRACKER ELISA kits, TNFα-Blocker ELISA kits, and Promonitor ELISA kits in patients with Crohn's disease compared with standard care (no anti-TNF monitoring). Where direct evidence is unavailable for this comparison, or where such a comparison is not well supported with evidence, a linked approach to evidence will be considered (see Objective C above) in which evidence of clinical effectiveness is taken from studies using alternative test methodology and an assessment is made of the relative performance this methodology relative to the intervention assays.

4. Methods for assessing clinical effectiveness

Systematic review methods will follow the principles outlined in the Centre for Reviews and Dissemination (CRD) guidance for undertaking reviews in health care[38] and the NICE Diagnostic Assessment Programme manual.[39]

4.1 Identification and selection of studies

4.1.1 Search strategies for clinical effectiveness

Scoping searches have been undertaken to inform the development of the search strategies. Additional phrases were added to the scoping searches to broaden the search to find other relevant articles that had no terms for the test name or type of test (e.g., Baert et al., 2003[26]) or population (e.g., Vande Casteele et al., 2012[40]) in title, abstract or indexing. Additional searches will be carried out where necessary. Searches for studies for cost and quality of life will be developed separately. An iterative procedure was used, with reference to scoping searches undertaken by information specialists at NICE. A copy of the main draft search strategy that is likely to be used in the major databases is provided in Appendix 3. This strategy may be further refined and other appropriate concepts may be added. This search strategy developed for Medline will be adapted as appropriate for other databases. All retrieved papers will be screened for potential inclusion.

The search strategy will comprise the following main elements:

- Searching of electronic bibliographic databases
- Contact with experts in the field
- Scrutiny of references of included studies
- Screening of manufacturer's and other relevant organisations' websites for relevant publications

Bibliographic databases will include:
MEDLINE; MEDLINE In-Process & Other Non-Indexed Citations; EMBASE; Cochrane Library (including Cochrane Systematic Reviews, DARE, CENTRAL, NHS EED, and HTA databases); Science Citation Index and Conference Proceedings (Web of Science); Index to Theses; DART-Europe; Dissertations & Theses; NIHR Health Technology Assessment Programme; PROSPERO (International Prospective Register of Systematic Reviews).

The following trial and patent databases will also be searched: Current Controlled Trials; ClinicalTrials.gov; UKCRN Portfolio Database; WHO International Clinical Trials Registry Platform; Espacenet (European Patent Office); Patentdocs (US Patents database).

Specific conference proceedings, to be selected with input from clinical experts and Specialist Committee Members, will be checked for the last five years.

The online resources of various health services research agencies, regulatory bodies, professional societies and manufacturers will be consulted via the Internet. These are likely to include:

- International Network of Agencies for Health Technology Assessment (INAHTA) Publication http://www.inahta.org/
- FDA medical devices: http://www.fda.gov/MedicalDevices/DeviceRegulationandGuidance/Databases/default.htm
- European Commission medical devices http://ec.europa.eu/health/medical-devices/
- Theradiag http://www.theradiag.com/en/
- Immundiagnostik http://www.immundiagnostik.com/en
- Proteomika http://www.proteomika.com/
- American college of gastroenterology http://gi.org/

This will be supplemented by web searching on specific test names using Google and a meta-search engine.

The reference lists of included studies and relevant review articles will be checked. Citation searches of selected included studies will be undertaken using Scopus. Identified references will be downloaded in Endnote X7 software. Included papers will be checked for errata using PubMed.

4.1.2 Inclusion and exclusion of relevant studies

Inclusion of relevant studies to address Objective A

Detailed information will be sought from manufacturers regarding mechanisms and reactants (in particular specificities and properties of antibodies and other reagents) employed in ELISA tests and radioimmunoassay, mobility shift assays and cell reporter tests (if used for a linked evidence approach).

In addition published studies which describe the intervention tests and tests used for a linked evidence approach will be identified. Those providing useful information about test mechanisms that is different or additional to that supplied by manufacturers of tests will be included. Assessment of inclusion will be based on the judgement of two reviewers.

Studies which compare test performance of two or more tests will be included either if they compare two or more intervention tests, or compare an intervention test with a test method which can be used to perform a linked evidence assessment.

All study designs will be considered for inclusion.

Inclusion criteria for studies to address Objective B

Studies that report an algorithm with the use of one of the intervention tests for the management of patients with Crohn's disease following measurement of serum levels of anti-TNF drug and anti-drug antibodies (infliximab or adalimumab). All study designs will be considered for inclusion.

Inclusion criteria for studies to address Objective C

Studies that satisfy the following criteria will be included:

Population	Crohn's disease patients (adults and children) receiving infliximab or adalimumab. If the evidence on Crohn's disease patients is limited, mixed patient groups containing Crohn's disease and ulcerative colitis patients will be included even if results are not reported separately. The limitations following from this will be discussed.
Intervention	Use of LISA-TRACKER ELISA kits, TNFα-Blocker ELISA kits, and Promonitor ELISA kits to estimate plasma or sera levels of anti-TNF agents and / or of ADAbs in which test results are employed in conjunction with a treatment algorithm (Table 6). Other assay methods will be considered should a linked evidence approach be adopted (Table 6).
Comparator	Standard care (Treatment decisions made on clinical judgement without measuring levels of TNF inhibitor and antibodies to TNF inhibitors).
Outcome	Any patient outcome (e.g., CDAI score based response rate, any measure of change in severity of Crohn's disease including physicians global assessment; Duration of response, relapse and remission; Rates of hospitalisation; Rates of surgical intervention; Time to surgical intervention; Adverse effects of treatment; Health related quality of life; and secondary if two strategies compared are found clinically equivalent: Time to result; Number of inconclusive results; Frequency of dose adjustment; Frequency of treatment switch).

Study design All study designs will be considered for inclusion.

Healthcare setting Secondary and tertiary care.

Meeting abstracts will be included if they provide sufficient data on type of ELISA assay, patient group, algorithm, measurements from assays and clinical outcomes.

Table 6. Assay methods included as interventions in the review

LISA-TRACKER assay kits (Theradig/Alpha Laboratories)

- LISA-TRACKER Adalimumab (LTA002)
- LISA-TRACKER Infliximab (LTI002)
- LISA-TRACKER anti-Adalimumab (LTA003)
- LISA-TRACKER anti-Infliximab (LTI003)
- LISA-TRACKER Duo Adalimumab (LTA005)
- LISA-TRACKER Duo Infliximab (LTI005)

Immundiagnostik TNFα-Blocker ELISA kits (Immundiagnostik/BioHit Healthcare):

- Immundiagnostik TNFα-Blocker ADA, antibodies against infliximab (e.g. Remicade®) ELISA (K9650)
- Immundiagnostik TNFα-Blocker ADA, antibodies against adalimumab (e.g. Humira®) ELISA (K9652)
- Immundiagnostik TNFα-Blocker ADA, TOTAL antibodies against infliximab (e.g. Remicade®) ELISA (K9654)
- Immundiagnostik TNFα-Blocker ADA, TOTAL antibodies against adalimumab (e.g. Humira®) ELISA (K9651)
- Immundiagnostik TNFα-Blocker monitoring, infliximab drug level (e.g. Remicade®) ELISA (K9655)
- Immundiagnostik TNFα-Blocker monitoring, adalimumab drug level (e.g. Humira®) ELISA (K9657)

Promonitor ELISA kits (Proteomika):

- Promonitor-ADL ELISA (5080230000)
- Promonitor-IFX ELISA (5060230000)
- Promonitor-ANTI-ADL ELISA (5090230000)
- Promonitor-ANTI-IFX ELISA (5070230000)

For Objective C test methods that are not included as an intervention but have evidence comparing it

to an intervention test and evidence reporting clinical outcomes, should be included for the purpose of performing linked evidence modelling only (including: radioimmunoassays, cell reporter assays, liquid-phase mobility shift assays and in-house ELISAs).

4.2 Review strategy

The general principles recommended in the PRISMA statement will be considered.[41] Records rejected at full text stage and reasons for exclusion will be documented. Two reviewers will independently screen the titles and abstracts of all records identified by the searches and discrepancies will be resolved through discussion. Disagreement will be resolved by retrieval of the full publication and consensus agreement. Full copies of all studies deemed potentially relevant, will be obtained and two reviewers will independently assess these for inclusion; any disagreements will be resolved by consensus or discussion with a third reviewer.

4.3 Data extraction strategy

Data will be extracted by one reviewer, using a piloted, data extraction form. A second reviewer will check the extracted data and any disagreements will be resolved by consensus or discussion with a third reviewer. Examples of data extraction sheets for patient-based and diagnostic accuracy studies are provided in Appendix 4.

4.4 Quality assessment strategy

Where appropriate, the quality of diagnostic accuracy studies will be assessed using QUADAS-2 (see Appendix 5).[42] As a broad range of study designs have been identified in the scoping searches, the use of a single checklist, in contrast to individual checklists for each study design, is considered appropriate. The Downs and Black checklist[43] will therefore be used to assess the quality of non-randomised studies meeting the inclusion criteria (see Appendix 5). This 27-item checklist provides both an overall score for study quality and a profile of scores not only for the quality of reporting, internal validity (bias and confounding) and power, but also for external validity. RCTs will be quality appraised using the Cochrane risk of bias tool (see Appendix 5).[44] The results of the quality assessment will provide an overall description of the quality of the included studies and will provide a transparent method of recommendation for design of any future studies. Quality assessment will be undertaken by one reviewer and checked by a second reviewer, any disagreements will be resolved by a third reviewer through discussion.

4.5 Methods of analysis/synthesis

Objective A

Narrative descriptions of tests in tables and texts will be undertaken.

Objective B

Algorithms will be narratively described and compared to the algorithm used in the TAXIT study (for good responders),[36] and the algorithm adapted from Scott and Lichtenstein (2014) (for secondary loss of response).[37] Non-compliant patients may be considered additionally in the algorithms. Time of testing, sequence of testing (drug and antibodies), sequence of analysis as well as thresholds used in the algorithms will be considered to address the research questions.

Objective C

Depending on the available evidence, analyses will be stratified according to the type of ELISA assay, type of drug (infliximab or adalimumab) and patient group (patients with secondary loss of response and patients with good response to anti-TNF treatment).

Study, treatment, population, and outcome characteristics will be summarised and compared qualitatively and, where possible, quantitatively in text, graphically and in evidence tables. Pooling studies results by meta-analysis will be considered. Where meta-analysis is considered unsuitable for some or all of the data identified (e.g., due to the heterogeneity and/or small numbers of studies), we will employ a narrative synthesis. Typically, this will involve the use of text, graphs and tables (as appropriate) to summarise data. These will allow the reader to consider any outcomes in the light of differences in study designs and potential sources of bias for each of the studies being reviewed. Studies will be organised by objective addressed. A detailed commentary on the major methodological problems or biases that affected the studies will also be included, together with a description of how this may have affected the individual study results.

For Objective C we aim to identify studies that compare treatment decisions made on clinical judgement without measuring levels of TNF inhibitor and antibodies to TNF inhibitors with treatment decisions based on measurement of TNF inhibitor and antibodies to TNF inhibitors. We will consider using a linked-evidence approach[45] in which studies report patient management informed by measurement of anti-TNF and antibodies by other methods (e.g., radioimmunoassay, liquid-phase mobility shift assay, in-house ELISAs); this will require an assessment of evidence relating to the comparable performance of ELISA assays with radioimmunoassay, liquid-phase mobility shift assays and in-house ELISAs.

In studies where an ELISA has been used but there is no comparator arm, or the comparator arm is a convenience sample (retrospective/historical population), outcomes will be listed and appraised. Time of testing, sequence of testing (drug and antibodies) and sequence of analysis will be considered to address the research questions.

5. Methods for synthesising cost-effectiveness evidence

5.1 Identifying and reviewing published cost-effectiveness studies

Published cost-effectiveness studies will be reviewed. All papers which present findings on the costs and outcomes of LISA-TRACKER ELISA kits, TNFα-Blocker ELISA kits, and Promonitor ELISA kits for measuring levels of TNF inhibitors and of anti-drug antibodies will be reviewed in detail. Information on assay procedures additional to ELISA methods will be sought for the purposes of providing data for a linked approach to evidence synthesis should this be required.

5.1.1 Search strategy and data extraction

A comprehensive search of the literature for published economic evaluations (including any existing models), cost studies and quality of life (utility) studies will be performed. The search strategy used will be based on the strategy developed for the clinical effectiveness review (see Appendix 3).

Databases will include:

- MEDLINE (Ovid)
- MEDLINE In-Process Citations and Daily Update (Ovid)
- EMBASE (Ovid)
- NHS Economic Evaluation Database (NHS EED) (Cochrane Library)
- Science Citation Index (Web of Knowledge)
- Cost-effectiveness analysis (CEA) registry
- Research Papers in Economics (REPAC)

Additional searches will be performed where necessary to identify other relevant information to support the development of an economic model for this project, these may be directed towards - costs, utilities and transition probabilities as required.

Data will be extracted by one reviewer and checked by a second, using a standardised data extraction form for the economic studies; this will be developed to summarise the main characteristics of the studies and to capture useful data that can inform the economic model. Any discrepancies will be resolved by discussion. If this is not feasible, a third reviewer will be consulted.

The quality of any full economic evaluation studies will be assessed using the CHEERS checklist (see Appendix 5).[46] Any studies containing an economic model will be further assessed using the framework for the quality assessment of decision analytic modelling (see Appendix 5).[47]

5.2 Evaluation of costs, quality of life and cost-effectiveness

5.2.1 Model structure, time horizon and transition probabilities

In developing the economic model we will consult the previous Health Technology Assessment report (HTA) conducted by Dretzke and colleagues (2011).[48] The main aim of this HTA report was to assess the cost-effectiveness of anti-TNFs in the management of moderate-to-severe Crohn's disease in the UK National Health Service (NHS). The authors developed a Markov model from an NHS and Personal Social Services (PSS) perspective to estimate the incremental cost per quality-adjusted life year (QALY) gained for both adalimumab and infliximab compared with standard care. The assumptions used in the model for the appraisal of Infliximab (review) and adalimumab for the treatment of Crohn's disease (technology appraisal 187)[48] may be used to inform the development of a de novo model. We will create a Markov-type model to assess the cost-effectiveness of LISA-TRACKER ELISA kits, TNFα-Blocker ELISA kits, and Promonitor ELISA kits compared with standard care. The perspective of the model will be that of the NHS and PSS. To assess the cost-effectiveness, the intervention tests (LISA-TRACKER ELISA kits, TNFα-Blocker ELISA kits, and Promonitor ELISA kits) will be compared with standard care in the following populations:

- In patients with secondary loss of response to anti-TNF treatment
- In patients who respond well to anti-TNF treatment

The following comparisons will be made where possible:

- Concurrent versus reflex testing
- Testing conducted every 3 to 4 months versus testing conducted at 3 to 4 months then yearly (in patients who respond well to anti-TNF treatment)

If data permits, we will compare the different LISA-TRACKER ELISA kits, TNFα-Blocker ELISA kits, and Promonitor ELISA kits with each other. In the absence of sufficient clinical data for specific ELISAs we will assume equal assay performance and compare ELISAs on the basis of cost only.

If data permits, a linked evidence approach will be adopted to compare LISA-TRACKER ELISA kits, TNFα-Blocker ELISA kits, and Promonitor ELISA kits with standard care in which clinical outcomes for the intervention arm are taken from studies in which the assay procedure was not one of the intervention assays; this will involve an assessment of the comparability of LISA-TRACKER ELISA kits, TNFα-Blocker ELISA kits, or Promonitor ELISA kits performance with that of the alternative procedure.

The model will have a one-year time horizon in line with the previous HTA report[48] and other studies we have found during our initial scoping search (e.g., Velayos et al., 2013).[49]

It is anticipated that information from the clinical effectiveness analyses will help inform the probabilities for each of the clinical pathways. Sensitivity analyses will be conducted in areas of uncertainty.

5.2.2 Resource use and costs

Resource use and costs will be estimated in line with the DAP programme manual. Information on resource use and costs associated with the different patient pathways (e.g., comparing clinical pathways followed when LISA-TRACKER ELISA kits, TNFα-Blocker ELISA kits, or Promonitor ELISA kits are employed, versus standard care pathway etc.) will be collected from systematic reviews of the literature, discussions with individual manufacturers and hospitals and if need be, by eliciting expert clinical advice. Any remaining gaps for resource use parameters will be filled by assumptions made by the research team.

Unit costs data will be based on national data were possible. For the different LISA-TRACKER ELISA kits, TNFα-Blocker ELISA kits, and Promonitor ELISA kits, costs will be from published list prices from the NHS supply chain, from the NHS reference costs,[50] or discussions with individual manufacturers or hospitals. Costs of consultations with secondary care staff will be drawn from Unit Costs of Health and Social Care[51] and drug costs will be obtained from the British National Formulary.[34]

5.2.3 Health outcomes

Health outcomes and utility data will be derived from the literature review including the previous HTA report and other sources. If direct measurements of utility or choice-based multi-attribute utility scales (such as the EQ-5D or SF-6D) suitable for calculation of QALYs for the economic model are not reported, we may need to use one of the algorithms for mapping from a clinical measure (e.g. CDAI) to a measure of utility. If insufficient information is available for utilities it may have to be elicited from an expert clinical panel or by assumptions made by the research team.

5.2.4 Cost-effectiveness analysis

The results of the cost-effectiveness analysis will be presented as an incremental cost per QALY gained for LISA-TRACKER ELISA kits, TNFα-Blocker ELISA kits, and Promonitor ELISA kits compared with standard care. If the data allows us to compare LISA-TRACKER ELISA kits, TNFα-Blocker ELISA kits, and Promonitor ELISA kits with each other, then we will undertake a rank comparison and exclude any options which are dominated or extended dominated. It may be necessary, in the absence of suitable clinical outcome data, to rank ELISAs on the basis of cost only.

We will use both simple and probabilistic sensitivity analysis to explore the robustness of the results and to estimate the impact of uncertainty over model parameters. The simple sensitivity analysis will

be used to assess the robustness of the results to changes in deterministic parameters such as costs, and utilities. The results from the probabilistic sensitivity analysis will be presented as cost-effectiveness acceptability curves. Decisions regarding mutually exclusive alternatives will be reflected using cost-effectiveness planes and cost-effectiveness acceptability curves or frontiers.

If a longer time horizon is chosen (more than one year), both costs and outcomes will be discounted using the recommended 3.5% discount rate by HM Treasury.

6. Handling of information from manufacturers

All data submitted by the manufacturers/sponsors will only be considered if received by the External Assessment Group before 27 January 2015. Data arriving after this date will not be considered. Any data that meets the inclusion criteria stated will be extracted and quality assessed as stated in the methods section of this protocol.

Any 'commercial in confidence' data provided by manufacturers, and specified as such, will be highlighted in blue and underlined in the assessment report (followed by company name in parentheses). Any 'academic in confidence' data provided by manufacturers, and specified as such, will be highlighted in yellow and underlined in the assessment report. All confidential data used in the cost-effectiveness models will also be highlighted.

7. Competing interests of authors and advisors

None of the authors have any competing interests.

8. Timetable/milestones

Draft assessment protocol	**06/10/2014**
Final protocol	**28/10/2014**
Progress report	**27/01/2015**
Draft assessment report	**24/03/2015**
Final assessment report	**23/04/2015**

9. Team members' contributions

Warwick Evidence is an External Assessment Group located within Warwick Medical School. Warwick Evidence brings together experts in clinical and cost effectiveness reviewing, medical statistics, health economics and modelling. The team planned for the work include:

Lead: Mrs Karoline Freeman

Title: Research Fellow
Address: Warwick Evidence, Populations, Evidence and Technologies, Division of Health Sciences, Warwick Medical School, University of Warwick, Coventry CV4 7AL
Tel: 02476 574026
Email: K.Freeman@warwick.ac.uk
Contribution: Protocol development, assessment for eligibility, quality assessment of trials, data extraction, data entry, and report writing

Name: Dr Martin Connock
Title: Senior Research Fellow
Address: Warwick Evidence, Populations, Evidence and Technologies, Division of Health Sciences, Warwick Medical School, University of Warwick, Coventry CV4 7AL
Tel: 02476 574940
Email: M.Connock@warwick.ac.uk
Contribution: Protocol development, assessment for eligibility, quality assessment of trials, data analysis, statistical modelling, and report writing

Name: Dr Hema Mistry
Title: Health economist
Address: Warwick Evidence, Populations, Evidence and Technologies, Division of Health Sciences, Warwick Medical School, University of Warwick, Coventry CV4 7AL
Tel: 02476 574490
Email: Hema.Mistry@warwick.ac.uk
Contribution: Protocol development, health economics modeller, data analysis, and report writing

Name: Dr Sian Taylor-Phillips
Title: Senior Research Fellow
Address: Warwick Evidence, Populations, Evidence and Technologies, Division of Health Sciences, Warwick Medical School, University of Warwick, Coventry CV4 7AL
Tel: 02476 575882
Email: S.Taylor-Phillips@warwick.ac.uk
Contribution: Protocol development, data analysis, and report writing

Name: Ms Rachel Court
Title: Information Specialist
Address: Warwick Evidence, Populations, Evidence and Technologies, Division of Health Sciences, Warwick Medical School, University of Warwick, Coventry CV4 7AL

Tel:	02476 522427
Email:	R.A.Court@warwick.ac.uk
Contribution:	Protocol development, develop search strategy and undertake the electronic literature searches

Name:	Dr Alexander Tsertsvadze
Title:	Senior Research Fellow
Address:	Warwick Evidence, Populations, Evidence and Technologies, Division of Health Sciences, Warwick Medical School, University of Warwick, Coventry CV4 7AL
Tel:	02476 574505
Email:	a_tsertsvadze@hotmail.com
Contribution:	Assessment for eligibility, quality assessment of trials, data extraction, data analysis, and report writing

Name:	Dr Jason Madan
Title:	Assistant Professor in Health Economics
Address:	Clinical Trials Unit, University of Warwick, Coventry CV4 7AL
Tel:	024761 51254
Email:	j.j.madan@warwick.ac.uk
Contribution:	Provide health economic modelling support, data analysis, and report writing

Name:	Dr Ngianga-Bakwin Kandala
Title:	Principal Research Fellow
Address:	Warwick Evidence, Populations, Evidence and Technologies, Division of Health Sciences, Warwick Medical School, University of Warwick, Coventry CV4 7AL
Tel:	02476 575054
Email:	N-B.Kandala@warwick.ac.uk
Contribution:	Data analysis and statistical modelling

Name:	Professor Aileen Clarke
Title:	Director of Warwick Evidence
Address:	Warwick Evidence, Populations, Evidence and Technologies, Division of Health Sciences, Warwick Medical School, University of Warwick, Coventry CV4 7AL
Tel:	02476 150189
Email:	Aileen.Clarke@warwick.ac.uk
Contribution:	Co-ordinate review process, protocol development, synthesis of findings and report writing

Name: Dr Paul Sutcliffe

Title: Associate Professor

Address: Warwick Evidence, Populations, Evidence and Technologies, Division of Health Sciences, Warwick Medical School, University of Warwick, Coventry CV4 7AL

Tel: 02476 574505

Email: p.a.sutcliffe@warwick.ac.uk

Contribution: Co-ordinate review process, protocol development, assessment for eligibility, quality assessment of trials, data extraction, data entry, data analysis, and report writing

9.1 Expert advisors

Name: Dr Ramesh P Arasaradnam

Title: Hon Assoc. Prof of Medicine and Consultant Gastroenterologist

Address: Clinical Sciences Research Institute, Clifford Bridge Road, Coventry CV2 2DX

Tel: 02476 966087

Email: r.arasaradnam@warwick.ac.uk

Contribution: Provide expert clinical advice on Crohn's and care pathways

Name: Dr Ahmed Naher

Title: Academic clinical fellow in clinical pharmacology and therapeutics

Address: Institute of Translational Medicine, University of Liverpool

Tel: 07949170357

Email: al.naher@gmail.com

Contribution: Provide expert advice on Crohn's and care pathways

10. References

1. Jerne NK. Towards a network theory of the immune system. *Annales d'immunologie*. 1974;**125c**(1-2):373-89.

2. Steenholdt C. Use of infliximab and anti-infliximab antibody measurements to evaluate and optimize efficacy and safety of infliximab maintenance therapy in Crohn's disease. *Danish medical journal*. 2013;**60**(4):B4616.

3. Allez M, Karmiris K, Louis E, Van Assche G, Ben-Horin S, Klein A, *et al.* Report of the ECCO pathogenesis workshop on anti-TNF therapy failures in inflammatory bowel diseases: definitions, frequency and pharmacological aspects. *Journal of Crohn's & colitis*. 2010;**4**(4):355-66.

4. Ben-Horin S, Chowers Y. Review article: loss of response to anti-TNF treatments in Crohn's disease. *Alimentary pharmacology & therapeutics*. 2011;**33**(9):987-95.

5. Cassinotti A, Travis S. Incidence and clinical significance of immunogenicity to infliximab in Crohn's disease: a critical systematic review. *Inflammatory bowel diseases*. 2009;**15**(8):1264-75.

6. Lee LY, Sanderson JD, Irving PM. Anti-infliximab antibodies in inflammatory bowel disease: prevalence, infusion reactions, immunosuppression and response, a meta-analysis. *European journal of gastroenterology & hepatology*. 2012;**24**(9):1078-85.

7. Chaparro M, Guerra I, Munoz-Linares P, Gisbert JP. Systematic review: antibodies and anti-TNF-alpha levels in inflammatory bowel disease. *Alimentary pharmacology & therapeutics*. 2012;**35**(9):971-86.

8. NHS choices. Crohn's disease. 2013 [cited 06/10/2014]; Available from: http://www.nhs.uk/Conditions/Crohns-disease/Pages/Introduction.aspx.

9. Jewell DP. Crohn's disease. *Medicine*. 2007;**35**(5):283-9.

10. Carter MJ, Lobo AJ, Travis SP. Guidelines for the management of inflammatory bowel disease in adults. *Gut*. 2004;**53 Suppl 5**:V1-16.

11. Hanauer SB, Sandborn W. Management of Crohn's disease in adults. *The American journal of gastroenterology*. 2001;**96**(3):635-43.

12. Jenkins HR. Inflammatory bowel disease. *Archives of disease in childhood*. 2001;**85**(5):435-7.

13. Silverberg MS, Satsangi J, Ahmad T, Arnott ID, Bernstein CN, Brant SR, *et al.* Toward an integrated clinical, molecular and serological classification of inflammatory bowel disease: report of a Working Party of the 2005 Montreal World Congress of Gastroenterology. *Canadian journal of gastroenterology = Journal canadien de gastroenterologie*. 2005;**19 Suppl A**:5a-36a.

14. Gasche C, Scholmerich J, Brynskov J, D'Haens G, Hanauer SB, Irvine EJ, *et al.* A simple classification of Crohn's disease: report of the Working Party for the World Congresses of Gastroenterology, Vienna 1998. *Inflammatory bowel diseases*. 2000;**6**(1):8-15.

15. Sostegni R, Daperno M, Scaglione N, Lavagna A, Rocca R, Pera A. Review article: Crohn's disease: monitoring disease activity. *Alimentary pharmacology & therapeutics*. 2003;**17 Suppl 2**:11-7.
16. Best WR, Becktel JM, Singleton JW, Kern F, Jr. Development of a Crohn's disease activity index. National Cooperative Crohn's Disease Study. *Gastroenterology*. 1976;**70**(3):439-44.
17. Yoshida EM. The Crohn's Disease Activity Index, its derivatives and the Inflammatory Bowel Disease Questionnaire: a review of instruments to assess Crohn's disease. *Canadian journal of gastroenterology = Journal canadien de gastroenterologie*. 1999;**13**(1):65-73.
18. Sandborn WJ, Feagan BG, Hanauer SB, Lochs H, Lofberg R, Modigliani R, *et al.* A review of activity indices and efficacy endpoints for clinical trials of medical therapy in adults with Crohn's disease. *Gastroenterology*. 2002;**122**(2):512-30.
19. Hyams J, Crandall W, Kugathasan S, Griffiths A, Olson A, Johanns J, *et al.* Induction and maintenance infliximab therapy for the treatment of moderate-to-severe Crohn's disease in children. *Gastroenterology*. 2007;**132**(3):863-73; quiz 1165-6.
20. Best WR. Predicting the Crohn's disease activity index from the Harvey-Bradshaw Index. *Inflammatory bowel diseases*. 2006;**12**(4):304-10.
21. Irvine EJ. Usual therapy improves perianal Crohn's disease as measured by a new disease activity index. McMaster IBD Study Group. *Journal of clinical gastroenterology*. 1995;**20**(1):27-32.
22. Irvine EJ, Feagan B, Rochon J, Archambault A, Fedorak RN, Groll A, *et al.* Quality of life: a valid and reliable measure of therapeutic efficacy in the treatment of inflammatory bowel disease. Canadian Crohn's Relapse Prevention Trial Study Group. *Gastroenterology*. 1994;**106**(2):287-96.
23. Mary JY, Modigliani R. Development and validation of an endoscopic index of the severity for Crohn's disease: a prospective multicentre study. Groupe d'Etudes Therapeutiques des Affections Inflammatoires du Tube Digestif (GETAID). *Gut*. 1989;**30**(7):983-9.
24. Schnitzler F, Fidder H, Ferrante M, Noman M, Arijs I, Van Assche G, *et al.* Long-term outcome of treatment with infliximab in 614 patients with Crohn's disease: results from a single-centre cohort. *Gut*. 2009;**58**(4):492-500.
25. Gisbert JP, Panes J. Loss of response and requirement of infliximab dose intensification in Crohn's disease: a review. *The American journal of gastroenterology*. 2009;**104**(3):760-7.
26. Baert F, Noman M, Vermeire S, Van Assche G, G DH, Carbonez A, *et al.* Influence of immunogenicity on the long-term efficacy of infliximab in Crohn's disease. *The New England journal of medicine*. 2003;**348**(7):601-8.
27. Hanauer SB, Feagan BG, Lichtenstein GR, Mayer LF, Schreiber S, Colombel JF, *et al.* Maintenance infliximab for Crohn's disease: the ACCENT I randomised trial. *Lancet*. 2002;**359**(9317):1541-9.
28. Maser EA, Villela R, Silverberg MS, Greenberg GR. Association of trough serum infliximab to clinical outcome after scheduled maintenance treatment for Crohn's disease. *Clinical*

gastroenterology and hepatology : the official clinical practice journal of the American Gastroenterological Association. 2006;**4**(10):1248-54.

29. Hanauer SB, Wagner CL, Bala M, Mayer L, Travers S, Diamond RH, *et al.* Incidence and importance of antibody responses to infliximab after maintenance or episodic treatment in Crohn's disease. *Clinical gastroenterology and hepatology : the official clinical practice journal of the American Gastroenterological Association*. 2004;**2**(7):542-53.

30. Lichtenstein GR, Hanauer SB, Sandborn WJ. Management of Crohn's disease in adults. *The American journal of gastroenterology*. 2009;**104**(2):465-83; quiz 4, 84.

31. Dignass A, Van Assche G, Lindsay JO, Lemann M, Soderholm J, Colombel JF, *et al.* The second European evidence-based Consensus on the diagnosis and management of Crohn's disease: Current management. *Journal of Crohn's & colitis*. 2010;**4**(1):28-62.

32. National Institute for Health and Care Excellence. Crohn's disease: Management in adults, children and young people. CG152. 2012 [cited 06/10/2014]; Available from: https://www.nice.org.uk/guidance/cg152.

33. BMJ Best Practice. Crohn's disease. 2014 [cited 06/10/2014]; Available from: http://bestpractice.bmj.com/best-practice/monograph/42/treatment/details.html.

34. British Medical Assocation and Royal Pharmaceutical Society of Great Britain. British National Formulary and British National Formulary for Children. [cited 06/10/2014]; Available from: http://www.bnf.org/bnf/index.htm.

35. National Institute for Health and Care Excellence. Infliximab (review) and adalimumab for the treatment of Crohn's disease. TA187. 2010 [cited 06/10/2014]; Available from: http://www.nice.org.uk/guidance/TA187.

36. Vande Casteele N, Gils A, Ballet V, Compernolle G, Peeters M, Van Steen K, *et al.* Randomised Controlled Trial of Drug Level Versus Clinically Based Dosing of Infliximab Maintenance Therapy in IBD: Final Results of the TAXIT Study (OP001). *United European Gastroenterology Journal*. 2013;**1**(1s):A1.

37. Scott FI, Lichtenstein GR. Therapeutic Drug Monitoring of Anti-TNF Therapy in Inflammatory Bowel Disease. *Curr Treat Options Gastroenterol*. 2014;**12**(1):59-75.

38. NHS Centre for Reviews and Dissemination. Undertaking systematic reviews of research on effectiveness: CRD guidelines for those carrying out or commissioning reviews. CRD Report 4. 1999.

39. National Institute for Health and Care Excellence. Diagnostics Assessment Programme manual. London, UK: National Institute for Health and Care Excellence; 2011 [cited 16/10/2014]. Available from: http://www.nice.org.uk/media/A0B/97/DAPManualFINAL.pdf.

40. Vande Casteele N, Buurman DJ, Sturkenboom MG, Kleibeuker JH, Vermeire S, Rispens T, *et al.* Detection of infliximab levels and anti-infliximab antibodies: a comparison of three different assays. *Alimentary pharmacology & therapeutics*. 2012;**36**(8):765-71.

41. Moher D, Liberati A, Tetzlaff J, Altman DG, The PRISMA Group. Preferred Reporting Items for Systematic Reviews and Meta-Analyses: The PRISMA Statement. *BMJ*. 2009;**339**:b2535.

42. Whiting PF, Rutjes AWS, Westwood ME, Mallett S, Deeks JJ, Reitsma JB, *et al.* QUADAS-2: A Revised Tool for the Quality Assessment of Diagnostic Accuracy Studies. *Annals of Internal Medicine*. 2011;**155**(8):529-36.

43. Downs SH, Black N. The feasibility of creating a checklist for the assessment of the methodological quality both of randomised and non-randomised studies of health care interventions. *Journal of epidemiology and community health*. 1998;**52**(6):377-84.

44. Higgins JP, Altman DG, Gotzsche PC, Juni P, Moher D, Oxman AD, *et al.* The Cochrane Collaboration's tool for assessing risk of bias in randomised trials. *Bmj*. 2011;**343**:d5928.

45. Merlin T, Lehman S, Hiller J, Ryan P. The "linked evidence approach" to assess medical tests: A critical analysis. *International Journal of Technology Assessment in Health Care*. 2013;**29**(03):343-50.

46. Husereau D, Drummond M, Petrou S, Carswell C, Moher D, Greenberg D, *et al.* Consolidated Health Economic Evaluation Reporting Standards (CHEERS) statement. *Int J Technol Assess Health Care*. 2013;**29**(2):117-22.

47. Philips Z, Ginnelly L, Sculpher M, Claxton K, Golder S, Riemsma R, *et al.* Review of guidelines for good practice in decision-analytic modelling in health technology assessment. *Health Technology Assessment*. 2004;**8**(36):1-158.

48. Dretzke J, Edlin R, Round J, Connock M, Hulme C, Czeczot J, *et al.* A systematic review and economic evaluation of the use of tumour necrosis factor-alpha (TNF-α) inhibitors, adalimumab and infliximab, for Crohn's disease. *Health Technology Assessment*. 2011;**15**(6):1-244.

49. Velayos FS, Kahn JG, Sandborn WJ, Feagan BG. A test-based strategy is more cost effective than empiric dose escalation for patients with Crohn's disease who lose responsiveness to infliximab. *Clinical Gastroenterology and Hepatology*. 2013;**11**(6):654-66.

50. Department of Health. NHS reference costs 2012 to 2013. 2013 [cited 18/03/2014]; Available from: https://www.gov.uk/government/publications/nhs-reference-costs-2012-to-2013.

51. Curtis L. Unit Costs of Health and Social Care 2013 [cited 18/03/2014]; Available from: http://www.pssru.ac.uk/project-pages/unit-costs/2013/.

52. European Medicines Agency. Remicade : EPAR - Product Information : Annex I - Summary of product characteristics. 2014 [cited 06/10/2014]; Available from: http://www.ema.europa.eu/docs/en_GB/document_library/EPAR_-_Product_Information/human/000240/WC500050888.pdf.

53. European Medicines Agency. Humira : EPAR - Product Information : Annex I - Summary of product characteristics. 2014 [cited 06/10/2014]; Available from: http://www.emea.eu.int/humandocs/PDFs/EPAR/Humira/H-481-PI-en.pdf.

Appendix 1. Licenced indications for Infliximab and Adalimumab in Crohn's disease

The licence indication for Crohn's disease detailed in the European Medicines Agency Summary of Product Characteristics (Remicade)[52] is as follows:
"Adult Crohn's disease: Remicade is indicated for:

- treatment of moderately to severely active Crohn's disease, in adult patients who have not responded despite a full and adequate course of therapy with a corticosteroid and/or an immunosuppressant; or who are intolerant to or have medical contraindications for such therapies;
- treatment of fistulising, active Crohn's disease, in adult patients who have not responded despite a full and adequate course of therapy with conventional treatment (including antibiotics, drainage and immunosuppressive therapy).

Paediatric Crohn's disease
Remicade is indicated for treatment of severe, active Crohn's disease, in children and adolescents aged 6 to 17 years, who have not responded to conventional therapy including a corticosteroid, an immunomodulator and primary nutrition therapy; or who are intolerant to or have contraindications for such therapies. Remicade has been studied only in combination with conventional immunosuppressive therapy.

Moderately to severely active Crohn's disease
5 mg/kg given as an intravenous infusion followed by an additional 5 mg/kg infusion 2 weeks after the first infusion. If a patient does not respond after 2 doses, no additional treatment with infliximab should be given. Available data do not support further infliximab treatment, in patients not responding within 6 weeks of the initial infusion.

In responding patients, the alternative strategies for continued treatment are:

- Maintenance: Additional infusions of 5 mg/kg at 6 weeks after the initial dose, followed by infusions every 8 weeks or
- Re-administration: Infusion of 5 mg/kg if signs and symptoms of the disease recur

Fistulising, active Crohn's disease
5 mg/kg given as an intravenous infusion followed by additional 5 mg/kg infusions at 2 and 6 weeks after the first infusion. If a patient does not respond after 3 doses, no additional treatment with infliximab should be given.

In responding patients, the alternative strategies for continued treatment are:

- Maintenance: Additional infusions of 5 mg/kg every 8 weeks or
- Re-administration: Infusion of 5 mg/kg if signs and symptoms of the disease recur followed by infusions of 5 mg/kg every 8 weeks.

Although comparative data are lacking, limited data in patients who initially responded to 5 mg/kg but who lost response indicate that some patients may regain response with dose escalation. Continued therapy should be carefully reconsidered in patients who show no evidence of therapeutic benefit after dose adjustment.

In Crohn's disease, experience with re-administration if signs and symptoms of disease recur is limited and comparative data on the benefit/risk of the alternative strategies for continued treatment are lacking.

Crohn's disease (6 to 17 years)
5 mg/kg given as an intravenous infusion followed by additional 5 mg/kg infusion doses at 2 and 6 weeks after the first infusion, then every 8 weeks thereafter. Available data do not support further infliximab treatment in children and adolescents not responding within the first 10 weeks of treatment.

Some patients may require a shorter dosing interval to maintain clinical benefit, while for others a longer dosing interval may be sufficient. Patients who have had their dose interval shortened to less than 8 weeks may be at greater risk for adverse reactions. Continued therapy with a shortened interval should be carefully considered in those patients who show no evidence of additional therapeutic benefit after a change in dosing interval."

The Adalimumbab licence indication for Crohn's disease detailed in the European Medicines Agency Summary of Product Characteristics (Humira)[53] is as follows:

Paediatric Crohn's Disease
Humira is indicated for the treatment of severe active Crohn's disease in paediatric patients (from 6 years of age) who have had an inadequate response to conventional therapy including primary nutrition therapy, a corticosteroid, and an immunomodulator, or who are intolerant to or have contraindications for such therapies.

Paediatric Crohn's disease patients < 40 kg:
The recommended Humira induction dose regimen for paediatric subjects with severe Crohn's disease is 40 mg at Week 0 followed by 20 mg at Week 2. In case there is a need for a more rapid response to

therapy, the regimen 80 mg at Week 0 (dose can be administered as two injections in one day), 40 mg at Week 2 can be used, with the awareness that the risk for adverse events may be higher with use of the higher induction dose.

After induction treatment, the recommended dose is 20 mg every other week via subcutaneous injection. Some subjects who experience insufficient response may benefit from an increase in dosing frequency to 20 mg Humira every week.

Paediatric Crohn's disease patients ≥ 40 kg:
The recommended Humira induction dose regimen for paediatric subjects with severe Crohn's disease is 80 mg at Week 0 followed by 40 mg at Week 2. In case there is a need for a more rapid response to therapy, the regimen 160 mg at Week 0 (dose can be administered as four injections in one day or as two injections per day for two consecutive days), 80 mg at Week 2 can be used, with the awareness that the risk for adverse events may be higher with use of the higher induction dose.

After induction treatment, the recommended dose is 40 mg every other week via subcutaneous injection. Some subjects who experience insufficient response may benefit from an increase in dosing frequency to 40 mg Humira every week.

Continued therapy should be carefully considered in a subject not responding by Week 12. A 40 mg pen and a 40 mg prefilled syringe are also available for patients to administer a full 40 mg dose. There is no relevant use of Humira in children aged less than 6 years in this indication.

Appendix 2. The CDAI Calculation of Crohn's Disease Activity Index (adapted from Best et al., 1976)[16]

Variable	Description	Scoring	Multiplier
No. of liquid stools	Sum of 7 days		x 2
Abdominal pain	Sum of 7 days' ratings	0=none<br>1=mild<br>2=moderate<br>3=severe	x 5
General well-being	Sum of 7 days' ratings	0=generally well<br>1=slightly under par<br>2=poor<br>3=very poor<br>4=terrible	x 7
Extraintestinal complications	Number of complications listed	Arthritis/arthralgia, iritis/uveitis, erythema nodosum, pyoderma gangrenosum, aphtous stomatitis, anal fissure/fistula/abscess, fever >37.8 °C	x 20
Anti-diarrhoeal drugs	Use in the previous 7 days	0=no<br>1=yes	x 30
Abdominal mass		0= no<br>2=questionable<br>5=definite	x 10
Haematocrit	Expected-observed Hct	Men: 47-observed<br>Women: 42-observed	x 6
Body weight	Ideal/observed ratio	(1-(ideal/observed)) x 100	x 1 (NOT< -10)

Appendix 3. Draft search strategy

Ovid MEDLINE(R) 1946 to October Week 2 2014, searched on 22/10/2014

1	adalimumab.mp.	3597
2	ADA.tw.	7105
3	infliximab.mp.	8842
4	IFX.tw.	326
5	((anti-TNF* or antiTNF* or TNF*) adj2 inhibitor*).mp.	2577
6	anti* tumo?r* necrosis* factor*.mp.	3007
7	Tumor Necrosis Factor-alpha/ and Antibodies, Monoclonal/	7682
8	anti* drug* antibod*.tw.	186
9	ADAb.tw.	19
10	1 or 2 or 3 or 4 or 5 or 6 or 7 or 8 or 9	24181
11	lisa* tracker*.mp.	1
12	(immundiagnostik* or immunodiagnostik* or immunediagnostik*).mp.	159
13	(proteomika* or promonitor*).mp.	13
14	exp Enzyme-Linked Immunosorbent Assay/	129174
15	enzyme* link* immunoassay*.mp.	2873
16	enzyme* link* immuno* assay*.mp.	158537
17	ELISA*.mp.	113426
18	11 or 12 or 13 or 14 or 15 or 16 or 17	205224
19	*Radioimmunoassay/	7091
20	(radioimmuno* or radio immuno* or radio-immuno*).mp.	101819
21	RIA.tw.	17353
22	reporter* gene* assay*.mp.	3663
23	RGA.tw.	336
24	semi* fluid* phase* enzyme* immuno*.mp.	0
25	EIA.tw.	8288
26	((homogenous* or homogeneous*) adj1 mobilit* shift* assay*).mp.	4
27	HMSA.tw.	62
28	(Biomonitor* or iLite).tw.	4102
29	(Matriks* Biotek* or Shikari*).mp.	2
30	(Prometheus* or Anser IFX or Anser ADA).mp.	258
31	19 or 20 or 21 or 22 or 23 or 24 or 25 or 26 or 27 or 28 or 29 or 30	124775
32	((monitor* or pharmacokinetic* or measur* or level* or concentration*) adj3 (adalimumab or ADA or infliximab or IFX or Anti-TNF* or Anti-Tumour Necrosis Factor*)).mp.	1087

33	Inflammatory Bowel Diseases/	14444
34	Crohn Disease/	31596
35	crohn*.tw.	32370
36	inflammator* bowel* disease*.tw.	26840
37	IBD.tw.	11936
38	33 or 34 or 35 or 36 or 37	58401
39	(((monitor* or pharmacokinetic* or measur* or level* or concentration*) adj3 (adalimumab or infliximab or Anti-TNF* or AntiTNF* or Anti-Tumour Necrosis Factor*)) and (correlat* or associat* or test performance)).mp.	218
40	10 and 18 and 38	93
41	10 and 31 and 38	19
42	32 and 38	157
43	39 or 40 or 41 or 42	367
44	Animals/ not Humans/	3983380
45	43 not 44	349

Appendix 4. Data extraction form for clinical effectiveness studies

Data extraction form anti-TNF drug monitoring

Name of first reviewer: *Name of second reviewer:*

**Study details**			
Study ID (Endnote ref)			
First author surname			
Year of publication			
Country			
Study design			
Publication (full/abstract)			
Study setting			
Number of centres (by arm)			
Duration of study			
Follow up period			
Funding			
**Aim of the study**			
**Inclusion/exclusion criteria for patients**			
Inclusion criteria:			
Exclusion criteria:			
**Study flow (consort diagram)**			
**Item**	**Anti-TNF monitoring arm**	**Clinical judgement arm**	**All**
N of Screened			
N of excluded (ineligible)			
N of enrolled/included (eligible)			
N of non-participants at study entry (those refused, etc…)			
N Study sample at baseline randomised (if applicable)			
Withdrawals			
Lost to follow up/drop outs (sample attrition)			
**Participants (characteristics and numbers)**			
**Item**	**Anti-TNF monitoring arm N (%)**	**Clinical judgement arm N (%)**	**All**
Total number of participants at baseline (% CD)			
N (%) followed up			
N (%) included in analysis			
Patient group (responders / secondary loss of response)			
Age Mean (SD/range)<br>Median (range) years			
Sex Women n (%)			
Diagnostic criteria for CD			
Children n (%)			
Crohn's Disease Activity Score (CDAI) Mean (SD)			
N (%) patients in remission			

N (%) patients with active CD			
CD classification (Vienna / Montreal)			
Disease duration (years)			
Smoking n (%)			
Previous surgery n (%)			
Concomitant treatment (specify) n (%)			
Treatment duration at anti-TNF failure (days)			
Line of therapy<br>1st<br>2nd<br>3rd			
Previous anti-TNF therapy n (%)			
CRP (mg/mL)			
Calprotectin (µg/g)			
**Treatment**			
**Item**	**Anti-TNF monitoring arm**	**Clinical judgement arm**	
Anti-TNF drug (name)			
Anti-TNF dose			
Duration of treatment			
**Intervention test assay (please specify):**<br>**Technical aspects of test assay:**			
Manufacturer			
Time of anti-TNF, antibody measurement			
Assay type			
Assay name			
Type of ELISA (bridging / capture)			
Anti-TNF alpha detection:			
Micro plate pre-coat			
Drug detection (free / total)			
Detection reagents (one-step / two-step)			
Assay range			
Limit of detection			
Reagents			
Antibody reagent specificity for antigen			
Structural class of immunoglobulin of antibody			
Anti-body detection:			
Micro plate pre-coat			
Anti-body detection (free / total)			
Incubation times			
Assay range			
Limit of detection			
Standards/calibrators			
**Outcomes reported**			
**Item**	**Anti-TNF**	**Clinical**	**All**

	monitoring arm	judgement arm	
Primary outcome(s)			
Secondary study outcomes			
Timing of assessments (including info on parallel or sequential)			
Time to test result			
Number of inconclusive results n (%)			
Frequency of dose adjustment n (%)			
Frequency of treatment switch n (%)			
Measure of disease activity (e.g., CDAI, others?)			
Rates of<br>a) response y/n<br>b) relapse y/n<br>c) remission y/n			
Describe definition of progression:			
Describe definition of remission:			
Duration of<br>a) response<br>b) relapse<br>c) remission			
Rates of hospitalisation n (%)			
Rates of surgical intervention n (%)			
Time to surgical intervention y/n			
Health related quality of life y/n			
Length of follow up reported y/n			
Proportion progressing to surgery n (%)			
Time to surgical intervention			
**Incidence of adverse effects of treatment:**			
**Item**	**Anti-TNF monitoring arm**	**Clinical judgement arm**	**P value**

**Dose monitoring**		
**Item (Please define if necessary)**	**Anti-TNF monitoring arm**	**Clinical judgement arm**
Time of anti-TNF/ antibody measurement		
Frequency of anti-TNF/ antibody measurement		
Assay type		
Assay name		
Threshold of infliximab / adalimumab (therapeutic / sub-therapeutic) (in µg/mL)		
Limit of quantification of anti-TNF antibodies (in U/mL [arbitrary unit/mL]) for Ab		

detectable / non-detectable		
Algorithm specified for management y/n (specify)		
Algorithm provided		
Number of patients outside therapeutic range		
Mean anti-TNF (mg/m^3/wk) (SD)		
Number of patients dose increased		
Number of patients dose reduced		
Other		
**Health related quality of life**		
**Item**	**Anti-TNF monitoring arm**	**Clinical judgement arm**
**Test comparison**		
**Tests**		
Intervention test		
Comparison test 1 (specify)		
Comparison test 2 (specify)		
Comparison test 3 (specify)		
Comparison test 1: test specifications (if ELISA use items for intervention assay test above)		
Comparison test 2: test specifications (if ELISA use items for intervention assay test above)		
Comparison test 3: test specifications (if ELISA use items for intervention assay test above)		
Details of any repeat measurements (to check reliability, performance across different laboratories)		
**Selection and storage of patients/plasma samples**		
Description of method of selection		
Description of method and duration of storage		
Number of clinical samples		
Number of calibrator samples (spiked) for anti-TNF		
Number of calibrator samples (spiked) for antibodies		
Number of blank (control) samples		
Total number of plasma samples		

Results of comparison			
Item	Intervention test vs test comparison 1	Intervention test vs test comparison 2	Intervention test vs test comparison 3
**Correlation of drug measurement:**			
Regression method			
Linearity test/cusum test?			
R^2 (95%CI)			
Slope (95%CI)			
Intercept (95%CI)			
**From Bland-Altman plot for drug measurement:**			
Percent bias (95%CI)			
Upper limit of agreement			
Lower limit of agreement			
Details of outliers			
Visually is there a pattern between the mean value and the difference? (If no pattern are statistics from Bland-Altman plot interpretable)			
N (%) samples outside limits of quantification, if yes specify decision for them			
N (%) false positives			
N (%) false negatives			
**Correlation of antibody measurement:**			
Regression method			
Linearity test/cusum test?			
R^2 (95%CI)			
Slope (95%CI)			
Intercept (95%CI)			
**From Bland-Altman plot for antibody measurement:**			
Percent bias (95%CI)			
Upper limit of agreement			
Lower limit of agreement			
Details of outliers			
Visually is there a pattern between the mean value and the difference? (If no pattern are statistics from Bland-Altman plot interpretable)			
N (%) samples outside limits of quantification, if yes specify decision for them			
N (%) false positives			
N (%) false negatives			
**Authors' conclusion**			
**Reviewer's conclusion**			

Appendix 5. Quality assessment forms

A – QUADAS-2[42] tool with index questions adapted to the review for studies comparing performance of different tests

Name of first reviewer: Name of second reviewer:

Phase 1: State the review question

Patients (setting, intended use of index test, presentation, prior testing):
Index test(s):
Reference standard:

Phase 2: Draw a flow diagram for the primary study

Phase 3: Risk of bias and applicability judgements

QUADAS-2 is structured so that four key domains are each rated in terms of the risk of bias and the concern regarding applicability to the review question (as stated in Phase 1). Each key domain has a set of signalling questions to help reach the judgements regarding bias and applicability.

Domain 1: Patient selection

**A. Risk of bias**	
Describe methods of patient selection:	
**Was a consecutive or random sample of patients enrolled?**	
**Did the study avoid inappropriate exclusions?**	
**Could the selection of patients have introduced bias?**<br><br>**Risk:**	
**B. Concerns regarding applicability**	
Describe included patients (prior testing, presentation, intended use of intervention test and setting):<br><br>Range of drug / antibody concentrations:	
**Is there concern that the included patients or range of drug / antibody concentrations do not match the review question?**<br><br>**Concern:**	

Domain 2: Index test(s)

**A. Risk of bias**	
Describe the intervention test and how it was conducted and interpreted:	
Were the number of failed results and measurement repeats reported?	
**Could the conduct or interpretation of the intervention test have introduced bias?**<br><br>**Risk:**	

**B. Concerns regarding applicability**<br><br>Describe the preparation and storage of the sample before the intervention test was applied:
**Is there concern that the intervention test, its conduct, or interpretation differ from the review question?**<br><br>**Concern:**

Domain 3: Reference standard (Comparison test)

**A. Risk of bias**	
Describe the comparison test and how it was conducted and interpreted:	
**Is the comparison test likely to correctly classify the target condition?**	
**Could the comparison test, its conduct, or its interpretation have introduced bias?**<br><br>**Risk:**	
**B. Concerns regarding applicability**	
**Is there concern that the target condition as defined by the comparison test does not match the review question?**<br><br>**Concern:**	

Domain 4: Flow and timing

**A. Risk of bias**	
Describe any patients who did not receive the intervention test and/or comparison test(s) or who were excluded from the Bland-Altman plot:<br><br>Describe the time interval and any interventions between intervention test and comparison test(s):	
**Was there an appropriate interval between intervention test and comparison test(s)?**	
**Were both intervention test and reference standard conducted on all samples?**	
**Did patients receive the same comparison test(s)?**	
**Were all patients included in the Bland-Altman plot?**	
**Could the patient flow have introduced bias?**<br><br>**Risk:**	

B – Cochrane Collaboration's tool for assessing risk of bias for a randomised controlled trial (adapted from Higgins et al., 2011[44])

First author surname and year of publication:

Name of first reviewer: ***Name of second reviewer:***

**Domain**	**Description**	**Review authors' judgement**
**Sequence generation**	Describe the method used to generate the allocation sequence in sufficient detail to allow an assessment of whether it should produce comparable groups	Was the allocation sequence adequately generated?
**Allocation concealment**	Describe the method used to conceal the allocation sequence in sufficient detail to determine whether intervention allocations could have been foreseen in advance of, or during, enrolment	Was allocation adequately concealed?
**Blinding of participants, personnel and outcome assessors** *Assessments should be made for each main outcome (or class of outcomes)*	Describe all measures used, if any, to blind study participants and personnel from knowledge of which intervention a participant received. Provide any information relating to whether the intended blinding was effective	Was knowledge of the allocated intervention adequately prevented during the study?
**Incomplete outcome data** *Assessments should be made for each main outcome (or class of outcomes)*	Describe the completeness of outcome data for each main outcome, including attrition and exclusions from the analysis. State whether attrition and exclusions were reported, the numbers in each intervention group (compared with total randomized participants), reasons for attrition/exclusions where reported, and any re-inclusions in analyses performed by the review authors	Were incomplete outcome data adequately addressed?
**Selective outcome reporting**	State how the possibility of selective outcome reporting was examined by the review authors, and what was found	Are reports of the study free of suggestion of selective outcome reporting?
**Other sources of bias**	State any important concerns about bias not addressed in the other domains in the tool. If particular questions/entries were pre-specified in the review's protocol, responses should be provided for each question/entry	Was the study apparently free of other problems that could put it at a high risk of bias?

Summary assessment of the risk of bias across domains (please highlight overall risk of bias rating)

**Risk of bias across key domains**	**Interpretation**	**Summary risk of bias**
Low risk of bias for all key domains	Plausible bias unlikely to seriously alter the results	Low risk of bias
Unclear risk of bias for one or more key domains	Plausible bias that raises some doubt about the results	Unclear risk of bias
High risk of bias for one or more key domains	Plausible bias that seriously weakens confidence in the results	High risk of bias

C – Downs and Black checklist[43] for non-randomised primary clinical studies

First author (year) study ID:

Name of first reviewer: *Name of second reviewer:*

Reporting	Rating
1. Is the hypothesis/aim/objective of the study clearly described? **(Yes/No)**	
2. Are the main outcomes to be measured clearly described in the Introduction or Methods section? **(Yes/No)** *If the main outcomes are first mentioned in the Results section, the question should be answered "No"*	
3. Are the characteristics of the patients included in the study clearly described? **(Yes/No)** *In cohort studies and trials, inclusion and/or exclusion criteria should be given. In case-control studies, a case-definition and the source for controls should be givenFsan*	
4. Are the interventions of interest clearly described? **(Yes/No)** *Treatments and placebo (where relevant) that are to be compared should be clearly described*	
5. Are the distributions of principal confounders in each group of subjects to be compared clearly described? **(Yes/Partially/No)** *A list of principal confounders is provided*	
6. Are the main findings of the study clearly described? **(Yes/No)** *Simple outcome data (including denominators and numerators) should be reported for all major findings so that the reader can check the major analyses and conclusions (This question does not cover statistical tests which are considered below)*	
7. Does the study provide estimates of the random variability in the data for the main outcomes? **(Yes/No)** *In non-normally distributed data the inter-quartile range of results should be reported. In normally distributed data the standard error, standard deviation or confidence intervals should be reported. If the distribution of the data is not described, it must be assumed that the estimates used were appropriate and the question should be answered "Yes"*	
8. Have all important adverse events that may be a consequence of the intervention been reported? **(Yes/No)** *This should be answered "Yes" if the study demonstrates that there was a comprehensive attempt to measure adverse events. (A list of possible adverse events is provided)*	
9. Have the characteristics of patients lost to follow-up been described? **(Yes/No)** *This should be answered "Yes" where there were no losses to follow-up or where losses to follow-up were so small that findings would be unaffected by their inclusion. This should be answered "No" where a study does not report the number of patients lost to follow-up*	
10. Have actual probability values been reported (e.g., 0.035 rather than <0.05) for the main outcomes except where the probability value is less than 0.001? **(Yes/No)**	
**External validity**	**Rating**
11. Were the subjects asked to participate in the study representative of the entire population from which they were recruited? **(Yes/No/Unable to determine)** *The study must identify the source population for patients and describe how the patients were selected. Patients would be representative if they comprised the entire source population, an unselected sample of*	

consecutive patients, or a random sample. Random sampling is only feasible where a list of all members of the relevant	
12. Were those subjects who were prepared to participate representative of the entire population from which they were recruited? **(Yes/No/Unable to determine)** *The proportion of those asked who agreed should be stated. Validation that the sample was representative would include demonstrating that the distribution of the main confounding factors was the same in the study sample and the source population*	
13. Were the staff, places, and facilities where the patients were treated, representative of the treatment the majority of patients receive? **(Yes/No/Unable to determine)** *For the question to be answered "Yes" the study should demonstrate that the intervention was representative of that in use in the source population. The question should be answered "No" if, for example, the intervention was undertaken in a specialist centre unrepresentative of the hospitals most of the source population would attend*	
**Internal validity – bias**	**Rating**
14. Was an attempt made to blind study subjects to the intervention they have received? **(Yes/No/Unable to determine)** *For studies where the patients would have no way of knowing which intervention they received, this should be answered "Yes"*	
15. Was an attempt made to blind those measuring the main outcomes of the intervention? **(Yes/No/Unable to determine)**	
16. If any of the results of the study were based on "data dredging", was this made clear? **(Yes/No/Unable to determine)** *Any analyses that had not been planned at the outset of the study should be clearly indicated. If no retrospective unplanned subgroup analyses were reported, then answer "Yes"*	
17. In trials and cohort studies, do the analyses adjust for different lengths of follow-up of patients, or in case-control studies, is the time period between the intervention and outcome the same for cases and controls? **(Yes/No/Unable to determine)** *Where follow-up was the same for all study patients the answer should "Yes". If different lengths of follow-up were adjusted for by, for example, survival analysis the answer should be "Yes". Studies where differences in follow-up are ignored should be answered "No"*	
18. Were the statistical tests used to assess the main outcomes appropriate? **(Yes/No/Unable to determine)** *The statistical techniques used must be appropriate to the data. For example nonparametric methods should be used for small sample sizes. Where little statistical analysis has been undertaken but where there is no evidence of bias, the question should be answered "Yes". If the distribution of the data (normal or not) is not described it must be assumed that the estimates used were appropriate and the question should be answered "Yes"*	
19. Was compliance with the intervention/s reliable? **(Yes/No/Unable to determine)** *Where there was non-compliance with the allocated treatment or where there was contamination of one group, the question should be answered "No". For studies where the effect of any misclassification was likely to bias any association to the null, the question should be answered "Yes"*	

20. Were the main outcome measures used accurate valid and reliable? **(Yes/No/Unable to determine)** *For studies where the outcome measures are clearly described, the question should be answered "Yes". For studies which refer to other work or that demonstrates the outcome measures are accurate, the question should be answered as "Yes"*	
**Internal validity - confounding (selection bias)**	**Rating**
21. Were the patients in different intervention groups (trials and cohort studies) or were the cases and controls (case-control studies) recruited from the same population? **(Yes/No/Unable to determine)** *For example, patients for all comparison groups should be selected from the same hospital. The question should be answered "Unable to determine" for cohort and case-control studies where there is no information concerning the source of patients included in the study*	
22. Were study subjects in different intervention groups (trials and cohort studies) or were the cases and controls (case-control studies) recruited over the same period of time? **(Yes/No/Unable to determine)** *For a study which does not specify the time period over which patients were recruited, the question should be answered as "Unable to determine"*	
23. Were the subjects randomised to intervention groups? **(Yes/No/Unable to determine)** *Studies which state that subjects were randomised should be answered "Yes" except where method of randomisation would not ensure random allocation. For example alternate allocation would score "No" because it is predictable*	
24. Was the randomised intervention assignment concealed from both patients and health care staff until recruitment was complete and irrevocable? **(Yes/No/Unable to determine)** *All non-randomised studies should be answered "No". If assignment was concealed from patients but not from staff, it should be answered "No"*	
25. Was there adequate adjustment for confounding in the analyses from which the main findings were drawn? **(Yes/No/Unable to determine)** *This question should be answered "No" for trials if: the main conclusions of the study were based on analyses of treatment rather than intention to treat; the distribution of known confounders in the different treatment groups was not described; or the distribution of known confounders differed between the treatment groups but was not taken into account in the analyses. In nonrandomised studies if the effect of the main confounders was not investigated or confounding was demonstrated but no adjustment was made in the final analyses the question should be answered as "No"*	
26. Were losses of patients to follow-up taken into account? **(Yes/No/Unable to determine)** *If the numbers of patients lost to follow-up are not reported, the question should be answered as "Unable to determine". If the proportion lost to follow-up was too small to affect the main findings, the question should be answered "Yes"*	
**Power**	**Rating**
27. Did the study have sufficient power to detect a clinically important effect where the probability value for a difference being due to chance is less than 5%? **(Yes/No/Unable to determine)***	

D – Critical appraisal of the economic evaluation studies using the CHEERS checklist (adapted from Husereau et al, 2013[46])

Title and abstract				
1 Title: Identify the study as an economic evaluation, or use more specific terms such as ``cost-effectiveness analysis``, and describe the interventions compared.				
2 Abstract: Provide a structured summary of objectives, methods including study design and inputs, results including base case and uncertainty analyses, and conclusions.				
Introduction				
3 Background & objectives: Provide an explicit statement of the broader context for the study. Present the study question and its relevance for health policy or practice decisions.				
Methods				
4 Target Population and Subgroups: Describe characteristics of the base case population and subgroups analysed including why they were chosen.				
5 Setting and Location: State relevant aspects of the system(s) in which the decision(s) need(s) to be made.				
6 Study perspective: Describe the perspective of the study and relate this to the costs being evaluated.				
7 Comparators: Describe the interventions or strategies being compared and state why they were chosen.				
8 Time Horizon: State the time horizon(s) over which costs and consequences are being evaluated and say why appropriate.				
9 Discount Rate: Report the choice of discount rate(s) used for costs and outcomes and say why appropriate.				

10 Choice of Health Outcomes: Describe what outcomes were used as the measure(s) of benefit in the evaluation and their relevance for the type of analysis performed.				
11a Measurement of Effectiveness - Single Study-Based Estimates: Describe fully the design features of the single effectiveness study and why the single study was a sufficient source of clinical effectiveness data.				
11b Measurement of Effectiveness - Synthesis-based Estimates: Describe fully the methods used for identification of included studies and clinical effectiveness data synthesis of clinical effectiveness data.				
12 Measurement and Valuation of Preference-based Outcomes: If applicable, describe the population and methods used to elicit preferences for health outcomes.				
13a Estimating Resources and Costs - Single Study-based Economic evaluation: Describe approaches used to estimate resource use associated with the alternative interventions. Describe primary or secondary research methods for valuing each resource item in terms of its unit cost. Describe any adjustments made to approximate to opportunity costs.				
13b Estimating Resources and Costs - Model-based Economic Evaluation: Describe approaches and data sources used to estimate resource use associated with model health states. Describe primary or secondary research methods for valuing each resource item in terms of its unit cost. Describe any adjustments made to approximate to opportunity costs.				
14 Currency, Price Date and Conversion: Report the dates of the estimated resource quantities				

and unit costs. Describe methods for adjusting estimated unit costs to the year of reported costs if necessary. Describe methods for converting costs into a common currency base and the exchange rate.				
15 Choice of Model: Describe and give reasons for the specific type of decision-analytic model used. Providing a figure to show model structure is strongly recommended.				
16 Assumptions: Describe all structural or other assumptions underpinning the decision-analytic model.				
17 Analytic Methods: Describe all analytic methods supporting the evaluation. This could include methods for dealing with skewed, missing or censored data, extrapolation methods, methods for pooling data, approaches to validate a model, and methods for handling population heterogeneity and uncertainty.				
Results				
18 Study parameters: Report the values, ranges, references, and if used, probability distributions for all parameters. Report reasons or sources for distributions used to represent uncertainty where appropriate. We strongly recommend the use of a table to show the input values.				
19. Incremental costs and outcomes: For each intervention, report mean values for the main categories of estimated costs and outcomes of interest, as well as mean differences between the comparator groups. If applicable, report incremental cost-effectiveness ratios.				
20a Characterizing Uncertainty - Single study-based economic evaluation: Describe the effects of sampling uncertainty for the estimated incremental cost and incremental effectiveness,				

parameters together with the impact of methodological assumptions.				
20b Characterizing Uncertainty - Model-based economic evaluation: Describe the effects on the results of uncertainty for all input parameters, and uncertainty related to the structure of the model and assumptions.				
21 Characterizing Heterogeneity: If applicable, report differences in costs, outcomes or in cost-effectiveness that can be explained by variations between subgroups of patients with different baseline characteristics or other observed variability in effects that are not reducible by more information.				
Discussion				
22 Study Findings, Limitations, Generalizability, and Current Knowledge: Summarize key study findings and describe how they support the conclusions reached. Discuss limitations and the generalizability of the findings and how the findings fit with current knowledge.				
Other				
23 Source of Funding: Describe how the study was funded and the role of the funder in the identification, design, conduct and reporting of the analysis. Describe other non-monetary sources of support.				
24 Conflicts of Interest: Describe any potential for conflict of interest among study contributors in accordance with journal policy. In the absence of a journal policy, we recommend authors comply with International Committee of Medical Journal Editors' recommendations.				

*Key: Y = yes, No = no, N/A = not applicable and * = partially completed*
