# Supplementary material for: Test accuracy of drug and antibody assays for predicting response to antitumour necrosis factor treatment in Crohn’s disease: a systematic review and meta-analysis
Source: BMJ Open. 2017 Jul 2;7(6):e014581. doi: 10.1136/bmjopen-2016-014581 (PMC5734585; doi:10.1136/bmjopen-2016-014581)
Supplement: Author's manuscript [file bmjopen-2016-014581.draft_revisions.pdf]

BMJ Open

Test accuracy of drug and antibody assays for predicting response to anti-Tumour Necrosis Factor treatment in Crohn's disease: a systematic review and meta-analysis

Journal:	*BMJ Open*
Manuscript ID	bmjopen-2016-014581
Article Type:	Research
Date Submitted by the Author:	19-Oct-2016

Complete List of Authors:	Freeman, Karoline; University of Warwick Warwick Medical School<br>Taylor-Phillips, Sian; University of Warwick, Warwick Medical School<br>Connock, Martin; University of Warwick, Division of Health Sciences, Warwick Medical School<br>Court, Rachel; Warwick University, Division of Health Sciences<br>Tsertsvadze, Alexander; University of Warwick Warwick Medical School<br>Shyangdan, Deepson; University of Warwick Warwick Medical School<br>Auguste, Peter; University of Warwick Warwick Medical School<br>Mistry, Hema; University of Warwick, Warwick Evidence<br>Arasaradnam, Ramesh; University Hospitals Coventry and Warwickshire NHS Trust, Gastroenterology<br>Sutcliffe, Paul; University of Warwick, Division of Health Sciences, Warwick Medical School<br>Clarke, Aileen; University of Warwick, Division of Health Sciences

**Primary Subject Heading**:	Gastroenterology and hepatology
Secondary Subject Heading:	Diagnostics
Keywords:	Inflammatory bowel disease < GASTROENTEROLOGY, Gastroenterology < INTERNAL MEDICINE, Adult gastroenterology < GASTROENTEROLOGY, meta-analysis, Systematic review, Infliximab

SCHOLARONE™ Manuscripts

Test accuracy of drug and antibody assays for predicting response to anti-Tumour Necrosis Factor treatment in Crohn's disease: a systematic review and meta-analysis

Karoline Freeman[1] K.Freeman@warwick.ac.uk

Sian Taylor-Phillips[1] S.Taylor-Phillips@warwick.ac.uk

Martin Connock[1] M.Connock@warwick.ac.uk>

Rachel Court[1] R.A.Court@warwick.ac.uk

Alexander Tsertsvadze[1] A.Tsertsvadze.1@warwick.ac.uk

Deepson Shyangdan[1] deepsonshyangdan@hotmail.com

Peter Auguste[1] P.Auguste@warwick.ac.uk

Hema Mistry[1] Hema.Mistry@warwick.ac.uk

Ramesh Arasaradnam[1,2] R.Arasaradnam@warwick.ac.uk

Paul Sutcliffe[1] P.A.Sutcliffe@warwick.ac.uk

Aileen Clarke[1] Aileen.Clarke@warwick.ac.uk

1. Warwick Medical School, University of Warwick, Coventry, UK
2. Gastroenterology, University Hospital Coventry and Warwickshire, Coventry, UK

Corresponding author: Sian Taylor-Phillips

Address: Warwick Medical School, The University of Warwick, Coventry, CV4 7AL

e-mail: s.taylor-phillips@warwick.ac.uk

Telephone: +44(0)7725000262

Keywords: Crohn disease, anti-TNF, meta-analysis, predictive value, sensitivity, specificity

Word count: 2475

For peer review only

ABSTRACT

Objective: To present meta-analytic test accuracy estimates of levels of anti-TNF and antibodies to anti-TNF to predict loss of response or lack of regaining response in anti-TNF managed Crohn's disease patients.

Methods: MEDLINE, Embase, the Cochrane Library and Science Citation Index were searched from inception to October / November 2014 to identify studies which reported 2x2 table data of the association between response and clinical status. Hierarchical / bivariate meta-analysis was undertaken with the user-written "metandi" package of Harbord and Whiting using Stata 11 software, for Infliximab, Adalimumab, anti-Infliximab and anti-Adalimumab levels as predictors of loss of response. Prevalence of Crohn's disease in included studies was meta-analysed using a random effects model in MetaAnalyst software to calculate positive and negative predictive values.

Results: 31 studies were included in the review. Studies were heterogeneous with respect to type of test used, criteria for establishing response and loss of response, and population examined. Meta-analytic results for sensitivity and specificity were 65.7% and 80.6% for Infliximab trough levels and 56% and 79% for Antibodies to Infliximab, respectively. Pooled results for Adalimumab trough levels and antibodies to Adalimumab were similar. Pooled positive and negative predictive values ranged between 70% and 80% implying that between 20% and 30% of tests results may be incorrect in predicting loss of response.

Conclusion: The available evidence suggests that these tests have modest predictive accuracy for clinical status. More clinical trial evidence from test-treat studies is required before the clinical utility of the tests can be reliably evaluated.

Strengths and Limitations of this study

- This is the first study to provide to summarise predictive accuracy of tests for loss of response to Crohns disease, in a clinically relevant manner
- We included more studies than previous meta-analyses
- We investigated drug and antibody levels for both infliximab and adulimumab
- Many of the included studies had a high risk of bias
- There was insufficient data for sub-group analyses for some types of test

For peer review only - http://bmjopen.bmj.com/site/about/guidelines.xhtml

INTRODUCTION

Anti-TNFα agents, including Infliximab [Remicade®, Merck Sharp & Dohme Ltd.] and Adalimumab [Humira®, AbbVie], are well-established second or third line therapies for people with Crohn's disease (CD). Failure to respond during induction therapy, and loss of response after initial success, are widely documented.[1-5] One suggested mechanism for this is the production of antibodies which neutralise the anti-TNFα agents and hasten their clearance from the circulation thus reducing drug availability. The treatment strategy for loss of response is usually to escalate the drug dosage or to shorten the dosage interval. If this fails, a switch to an alternative anti-TNF agent can be tried in order to minimise the influence of anti-drug antibodies directed against the first agent. Another suggested underlying mechanism for loss of response is that cytokines other than TNFα may become the major inflammatory agents. This suggestion arises from the observation that some patients have a loss of response to anti-TNF despite the presence of therapeutic drug levels and an absence of anti-TNF antibodies. For such patients the continued use of anti-TNFs may be considered futile and a switch to different biological therapies or other agents may represent the preferred strategy.

The potential role of anti-TNF antibodies and of sub-therapeutic drug levels in loss of response has provided the impetus for the development of assays for both anti-TNF drugs and for antibodies and a plethora of studies using such assays has been produced, exploring the association between either levels of antibodies to anti-TNF agents and clinical response or levels of drugs and clinical response. Studies have measured loss of response to the administered anti-TNF agent or failure to regain response after a change in treatment. By dichotomising the outcomes at various detectable levels of drug and of antibodies to anti-TNF, the diagnostic value of these tests in predicting loss of response or lack of regaining response has been assessed.

Several authors have meta-analysed studies which have reported the association between levels of antibodies to anti-TNF agents and clinical status.[6-9] These authors have presented pooled relative risk or odds ratio statistics for clinical state (e.g. response or loss of response) investigating positive versus negative test result patients (i.e. antibodies to anti-TNF agent present or absent), or conversely for test result (positive or negative) in patients with response versus those without response. Although these pooled statistics provide useful information on the association between antibody levels and clinical status, they do not address the question of test accuracy when tests are used as a predictor of patients' clinical response status which is the perspective likely to be adopted by clinicians for patients receiving treatment that may be predicated on test results. Primary studies frequently report test accuracy analysis such as receiver operating characteristic curves and test accuracy measures such as sensitivity and specificity. When viewed as diagnostic tests[10] it becomes possible to perform alternative meta-analysis so as to obtain pooled estimates of test accuracy. The predictive accuracy of such tests is of considerable practical interest. Our objective therefore is to present the meta-analytic results in terms of pooled test accuracy estimates. A particular advantage of this method is that it allows for investigation

For peer review only

of the co-variance of associations or, from the perspective of a predictive test, the covariance between sensitivity and specificity, thus giving a more complete picture of the value of these tests in clinical practice.

METHODS

Search for studies

An iterative procedure was used to develop the initial MEDLINE search, which was subsequently adapted appropriately for other databases and sources. We searched multiple bibliographic databases including MEDLINE, Embase, the Cochrane Library and Science Citation Index from inception to October / November 2014. Searches of other online resources including trial registries were also undertaken. Full details of the search strategies used, with exact search dates, are provided in Supplement 1. Reference lists of included studies and relevant review articles were checked. Citation searches of selected included studies were undertaken.

Study eligibility criteria

We included studies of patients with Crohn's disease treated with Infliximab or Adalimumab. The intervention of interest was a test measuring serum anti-TNFα (Infliximab or Adalumimab) and / or anti-Infliximab or anti-Adalimumab antibody levels. Studies reporting clinical status (i.e., response or lack of response) as an outcome were eligible for inclusion. The reported results had to allow the cross-tabulation of dichotomous test response with clinical status by means of two-by-two tables in order to calculate the diagnostic test accuracy parameters. All primary study designs were included.

Study selection

Two reviewers independently assessed titles and abstracts for inclusion using a pre-piloted form. All potentially relevant publications were retrieved and examined independently. Any disagreements regarding inclusion/exclusion were discussed and resolved with a third reviewer. The study selection process and reasons for exclusion at full text screening level are presented in the PRISMA study flow diagram (see Figure 1).

Quality assessment

Studies were quality assessed using a modified QUADAS-2 checklist.[11] Items included were method of patient selection, blinding of index test results, exclusion of uninterpretable test results from 2x2 table data and method of assessment of clinical status (the reference case).

Evidence synthesis and statistical methods

Patient numbers within extracted two by two data tables were used to generate Forest plots of paired sensitivity and specificity (accompanied by 95% CIs) using Review Manager (RevMan 5.1; Nordic Cochrane Centre, Copenhagen, Denmark) for four different tests: (1) Infliximab levels as predictor of

For peer review only

loss of or lack of regaining response, (2) Antibodies to Infliximab as predictor of loss of or lack of regaining response, (3) Adalimumab levels as predictor of loss of or lack of regaining response, and (4) Antibodies to Adalumimab as predictor of loss of or lack of regaining response. Hierarchical / bivariate[12] meta-analysis was undertaken with the user-written "metandi" package of Harbord and Whiting[13] using Stata 11 software. Positive and negative predictive values were calculated[14] at the pooled prevalence of loss of response in the test population. Prevalence was meta-analysed using a random effects model in MetaAnalyst software.[15] For meta-analyses which incorporated 10 or more studies we examined the risk of publication bias (Appendix 5) mindful of the caveats relating to this in diagnostic test accuracy studies.[16]

The protocol for this review was registered with reference PROSPERO 2014:CRD42014015278. The full protocol is included in appendix 1.

RESULTS

We identified 2429 records of which 31 were eligible for inclusion Of these 24 were full-text reports and 7 were conference abstracts. The PRISMA flow diagram is detailed in Figure 1. Eleven of the 31 studies examined Infliximab trough levels, 20 examined trough level of antibodies to Infliximab and five and six studies respectively investigated Adalimumab levels and antibodies to Adalimumab. (Table 1.) The range of anti-TNF cut-offs used for the dichotimisation of test outcomes is illustrated in Supplement 2 (Tables S1-S3). The risk of bias of studies varied. The greatest threat to validity was high risk of bias in patient selection which was present in nearly 80% of included studies (Supplement 3).

The studies were heterogeneous with respect to type of test used (e.g. commercial or in-house ELISA, RIA, HMSA), criteria for establishing response or lack of regaining response (e.g. use of the CDAI or the physician's global assessment score), and population examined (responders or patients with secondary loss of response). Sensitivity and specificity pairs are summarised in Figures 2 for antibodies to anti-TNF and Figure 3 for anti-TNF trough levels.

The paired Forest plots show that sensitivity and spcificity of using anti-TNFs or antibodies produced against anti-TNFs to predict response or loss of response varies greatly among studies with senstivity revealing generally greater variation. None of the presented covariates (population, assay type, response criterion) appear to explain the observed variation.

For peer review only

Table 1 Major features of studies included for hierarchical meta-analyses

STUDY	DRUG	DIAGNOSIS	RESPONSE/LOR	TEST	RESPONSE MEASURE
Infliximab trough level as predictor of loss of or lack of regaining response					
Ainsworth 2008[17]	IFX	CD	LOR	RIA	PJ
Ben-Basset 2013[18] abstract	IFX	IBD ~.93 CD	Resp	HMSA	HBI
Bortlik 2013[19]	IFX	CD	Resp	ELISA	PJ
Cornillie 2014 #410}	IFX	CD	Resp	ELISA	CDAI
Hibi 2014[20]	IFX	CD	Resp	ELISA	CDAI
Imaeda 2012[21]	IFX	CD	Resp	ELISA	CDAI
Kopylov 2012[22]	IFX	CD	Resp	ELISA	PJ
Maser 2006[23]	IFX	CD	Resp	ELISA	HBI
Steenholdt 2011[24]	IFX	CD	Resp	RIA	PJ
Steenholdt 2014[25]	IFX	CD	LOR	RIA	CDAI
Yanai 2012[26] abstract	IFX	CD	Resp	ELISA	PJ
Trough antibodies to Infliximab as predictor of loss of or lack of regaining response					
Ainsworth 2008[17]	IFX	CD	LOR	RIA	PJ
Baert 2014[27]	IFX	IBD ~0.8 CD	LOR	HMSA	PJ
Ben-Horin 2011[28]	IFX	IBD ~0.82 CD	Resp	NR	ST
Ben-Horin 2012[29]	IFX ADA	IBD ~0.9 CD	LOR	ELISA	PJ
Bodini 2014[30] abstract	IFX	CD	Resp	HMSA	HBI
Candon 2005[31]	IFX	CD	LOR	ELISA	UC
Dauer 2013[32] abstract	IFX	CD ~.83 CD	Resp	NR	PJ
Farrell 2003[33]	IFX	CD	Resp	ELISA	PJ
Hanauer 2004[34]	IFX	CD	Resp	ELISA	CDAI
Imaeda 2012[21]	IFX	CD	Resp	ELISA	CDAI
Kong 2011[35] abstract	IFX	IBD ~.83 CD	Resp	ELISA	PJ
Kopylov 2012[22]	IFX	CD	Resp	ELISA	PJ
Marzo 2014[36] abstract	IFX	NR	Resp	ELISA	CDAI
Nagore 2015[37] abstract	IFX	IBD ~.86 CD	Resp	ELISA	PJ
Pariente 2012[38]	IFX	CD & UC	LOR	ELISA	PJ or HBI
Steenholdt 2011[24]	IFX	CD	Resp	RIA	PJ ST
Steenholdt 2013[39]	IFX	CD	Resp	ELISA	PJ
Steenholdt 2014[25]	IFX	CD	LOR	RIA	CDAI
Vande Casteele 2013[40]	IFX	IBD ~0.70 CD	LOR	HMSA	CRP TC
Vande Casteele 2013[40]	IFX	IBD ~0.70 CD	Resp	HMSA	CRP TC
Adalimumab trough level as predictor of loss of or lack of regaining response					
Chiu 2013[41]	ADA	CD	LOR	ELISA	CDAI
Frederiksen 2014[42]	ADA	IBD	Resp	RIA	PJ BM
Imaeda 2014[43]	ADA	CD	Resp	ELISA	CRP
Mazor 2014[44]	ADA	CD	Resp	ELISA	PJ + CRP
Roblin 2014[45]	ADA	CD	Resp	ELISA	CDAI
Trough antibodies to Adalimumab as predictor of loss of or lack of regaining response					
Frederiksen 2014[42]	ADA	IBD	Resp	RIA	PJ BM
Imaeda 2014[43]	ADA	CD	Resp	ELISA	CRP
Mazor 2014 [44]	ADA	CD	Resp	ELISA	PJ + CRP
West 2008[46]	ADA	CD	Resp	RIA	PJ
Ben-Horin 2012[29]	IFX ADA	IBD ~0.9 CD	LOR	ELISA	SA
Roblin 2014[45]	ADA	CD	Resp	ELISA	CDAI

Diagnosis = study patient population; LOR = patients with loss of response ; Response = responding patients; Response measure = method used for defining clinical response; abs = abstract; ADA = Adalimumab; IFX = Infliximab; CD = Crohn's disease; IBD = inflammatory bowel disease; ELISA = enzyme linked immunoassay; RIA = radioimmunoassay; CDAI = Crohn's disease activity index score; CRP = C reactive protein level; PJ = physicians' judgement ; PJ BM = physicians' judgement and biological measure; HBI = Harvey Bradshaw Index score; SA = switch anti-TNF; ST = stop anti-TNF; TC = treatment change

For peer review only - http://bmjopen.bmj.com/site/about/guidelines.xhtml

Infliximab trough level tests for loss of response or lack of regaining response

Of eleven included studies, two were reported only as abstracts (Ben-Basset, 2013[18] and Yanai, 2012[26]). The Meta-analysis (Figure 4) yielded a pooled summary point of 0.66 sensitivity and 0.81 specificity (other test accuracy statistics are summarised in Supplement 4 Table S4). Sensitivity analysis in which only studies of responder populations were included generated very similar results as did analysis that only included studies with ELISA tests.

Antibodies to Infliximab tests for loss of response or lack of regaining response

Of twenty included studies, five were reported as abstracts.[30 32 35-37] Sensitivity and specificity pairs are summarised in Figure 5. The pooled summary point sensitivity and specificity were 0.56 and 0.79 respectively (Figure 5). Only minor differences were introduced in the test accuracy outcomes (e.g. 0.60 and 0.81 for sensitivity and specificity respectively) in a sensitivity analysis when two influential studies were omitted from the analysis.[34 40] Similarly, sensitivity analyses in which only ELISA studies and only responder studies were included had little effect although in the former there was an improvement in specificity at the expense of sensitivity (Figure 5).

Adalimumab and anti-Adalimumab antibody trough levels as tests for loss of response or lack of regaining response

Far fewer studies of Adalimumab-treated patients were available compared to Infliximab (Table 1). Meta-analysis of Adalimumab-treated patients yielded slightly lower test accuracy statistics with wider uncertainty around them compared to those found for Infliximab studies (Supplement 4 Table S4 and Figure S1).

Predictive values of drug and anti-drug antibody tests for LOR or failure to regain response

In the Cochrane Handbook for Systematic Reviews of Diagnostic Test Accuracy, Bossuyt et al. (2013) [14] suggest that predictive values are more widely and readily appreciated than alternative test accuracy statistics such as sensitivity and specificity. Negative and positive predictive values vary according to prevalence of the condition being tested for (in this case lack of response). We have meta-analysed the prevalence across the included studies and used this with its 95% CI as a guide to the likely prevalence range across which the tests would be performed in practice. The predictive values for each type of test across the relevant prevalence ranges are summarised in Figure 6. As prevalence increases positive predictive value increases and negative predictive value decreases.

Although pooled prevalence varies somewhat amongst the four collections of studies the resulting positive and negative predictive values are similar and range between about 70% and 80% implying that between 20% and 30% of positive and negative test results are likely to be incorrect.

For peer review only - http://bmjopen.bmj.com/site/about/guidelines.xhtml

DISCUSSION

The meta-analysis results indicate that the test accuracy of tests for predicting lack of response was moderate and that about 20 to 30% of test results are likely to be incorrect. There was no evidence of publication bias for either the Infliximab tests or tests for antibodies to Infliximab. The number of studies on Adalimumab treated patients was too small to draw firm conclusions but the available evidence suggests very similar performance to the tests for Infliximab and for antibodies to Infliximab.

The sensitivity analyses indicated that the variation seen in the Forest plots and ROC space could not be explained by test type, population and response criterion used. Test performance is dependent on cut-offs used for anti-TNF and antibodies to anti-TNF agents. However, this was not investigated in sensitivity analyses as cut-offs vary by test type as well as within different types of tests and an agreed cut-off that is transferable between studies and populations has yet to be identified.

Our meta-analyses included studies using different tests for measuring levels of anti-TNF agents and antibodies to anti-TNFs. Although radioimmunoassay and HMSA tests were used in some of our included studies the bulk of the tests employed were ELISA tests (26/42, 62%) encompassing various commercial ELISA kits and ELISAs developed “in house” by investigators. Several full publications and abstracts have addressed the issue of whether different test methods (e.g. solid phase ELISAs, liquid phase assays such as RIA or HMSA) deliver the same quantitative estimates of drug and antibody levels in patient samples. [21 22 25 30 40 43 47-65] Because there is no consensus about what constitutes a gold standard test, it is difficult to draw conclusions from these studies other than that differences in performance have been documented. Interestingly, the observed variation in our meta-analysis could not be explained by the different tests used.

Although the accuracy of the tests for predicting lack of response was found to be moderate this does not necessarily mean they must lack clinical utility. However, clinicians are likely to be interested in a combined assessment of anti-TNF levels and antibodies to anti-TNF, for which limited accuracy data is available.[21 25 43] And because diagnostic tests may alter clinical decisions and actions, evidence beyond test accuracy is required to evaluate clinical value.[66] Such evidence is best obtained in randomised trials (i.e. test and treat investigations) but this is currently sparse.[66]

Two recent RCTs have compared clinical outcomes between patients whose treatment was directed by algorithms informed by tests for Infliximab and/or antibodies to Infliximab versus patients who received treatment uninformed by testing.[25 67] In the TAXIT trial[67] IBD patients responding to Infliximab had their dose regimen optimised according to a test-algorithm with the aim to bring patients within the therapeutic range and prevent loss of response. However after randomisation to clinically-based or test-based dosing, no clinical benefit was observed for CD patients at one year. Steenholdt et

For peer review only - http://bmjopen.bmj.com/site/about/guidelines.xhtml

al. (2014)[25] investigated patients who had lost response to Infliximab in order to predict the reason for loss of response and adjust treatment accordingly. In this study no clinical benefit was observed for the test-algorithm group of patients and the control group who all received intensification. It is notable in this study that for many patients (14/33; 42%) clinicians failed to implement the test-algorithm directive, implying that they may have lacked confidence in the test results or that they considered other factors of overriding importance; as pointed out by Ferranti di Ruffano et al. (2012)[66]. Such phenomena (lack of equipoise) complicate assessments of test value. Both of these RCTs reported cost savings in the test-algorithm arm associated with reduced use of Infliximab.

This is the first meta-analysis of predictive accuracy of these tests and offers an alternative perspective to earlier meta-analyses. We were able to include more studies than in earlier meta-analyses and have looked at both drug tests as well as tests for anti-drug antibodies, and have included studies of patients receiving either Infliximab or Adalimumab therapies.

The meta-analysis results should be viewed with some caution because of the high risk of bias in many of the included studies, and because the lack of sufficient numbers of studies precluded subgroup meta-analyses of some types of test (e.g. RIA, HMSA).

CONCLUSIONS

The available evidence suggests that these tests have modest predictive accuracy for clinical status and that about 20 to 30% of test results would be likely to be incorrect. However, higher quality studies are required to enable differentiation between different types of test, and in published trials the tests have been used for adjusting dose or treatment of patients whose clinical status has already been defined by other criteria. More clinical trial evidence from test-treat studies is required before the clinical utility of the tests can be reliably evaluated.

For peer review only

For peer review only - http://bmjopen.bmj.com/site/about/guidelines.xhtml

Competing interests: Aileen Clarke is one of the editors of the Health Technology Assessment journal. All other authors have no conflicts of interest.

Source of funding: This work was commissioned by the NIHR HTA Programme as project number 14/69/03

Aileen Clarke and Sian Taylor-Phillips are partly supported by the National Institute for Health Research (NIHR) Collaboration for Leadership in Applied Health Research and Care West Midlands at the University Hospitals Birmingham NHS Foundation Trust.

Data sharing: All data is available from authors upon request

Contributions: KF and MC drafted the paper. RC developed the search strategy and undertook searches. MC, KF, STP, AT and DS conducted the systematic review. MC conducted the data analysis. PS and AC provided project management and funding acquisition. RA provided clinical comment and guidance. KF, MC, STP, RC, AT, DS, HM, PA, PS, AC and RA contributed to protocol development, commented on drafts of the paper and approved the final version.

References

1. Ben-Horin S, Chowers Y. Tailoring anti-TNF therapy in IBD: drug levels and disease activity. Nature Reviews Gastroenterology & Hepatology 2014;**11**(4):243-55 doi: http://dx.doi.org/10.1038/nrgastro.2013.253[published Online First: Epub Date]|.
2. Targan SR, Hanauer SB, van Deventer SJ, et al. A short-term study of chimeric monoclonal antibody cA2 to tumor necrosis factor alpha for Crohn's disease. Crohn's Disease cA2 Study Group. N Engl J Med 1997;**337**(15):1029-35 doi: 10.1056/nejm199710093371502[published Online First: Epub Date]|.
3. Hanauer SB, Sandborn WJ, Rutgeerts P, et al. Human anti-tumor necrosis factor monoclonal antibody (adalimumab) in Crohn's disease: the CLASSIC-I trial. Gastroenterology 2006;**130**(2):323-33 doi: 10.1053/j.gastro.2005.11.030[published Online First: Epub Date]|.
4. Gisbert JP, Panes J. Loss of response and requirement of infliximab dose intensification in Crohn's disease: a review. Am J Gastroenterol 2009;**104**(3):760-7 doi: 10.1038/ajg.2008.88[published Online First: Epub Date]|.
5. de Boer N, Lowenberg M, Hoentjen F. Management of Crohn's disease in poor responders to adalimumab. Clin Exp Gastroenterol 2014;**7**:83-92 doi: http://dx.doi.org/10.2147/CEG.S47627[published Online First: Epub Date]|.
6. Nanda KS, Cheifetz AS, Moss AC. Impact of antibodies to infliximab on clinical outcomes and serum infliximab levels in patients with inflammatory bowel disease (IBD): a meta-analysis. Am J Gastroenterol 2013;**108**(1):40-7 doi: http://dx.doi.org/10.1038/ajg.2012.363[published Online First: Epub Date]|.
7. Paul S, Moreau AC, Del Tedesco E, et al. Pharmacokinetics of adalimumab in inflammatory bowel diseases: a systematic review and meta-analysis. Inflamm Bowel Dis 2014;**20**(7):1288-95 doi: http://dx.doi.org/10.1097/MIB.0000000000000037[published Online First: Epub Date]|.
8. Garces S, Demengeot J, Benito-Garcia E. The immunogenicity of anti-TNF therapy in immune-mediated inflammatory diseases: a systematic review of the literature with a meta-analysis. Ann Rheum Dis 2013;**72**(12):1947-55 doi: http://dx.doi.org/10.1136/annrheumdis-2012-202220[published Online First: Epub Date]|.
9. Lee LY, Sanderson JD, Irving PM. Anti-infliximab antibodies in inflammatory bowel disease: prevalence, infusion reactions, immunosuppression and response, a meta-analysis. Eur J Gastroenterol Hepatol 2012;**24**(9):1078-85 doi: 10.1097/MEG.0b013e32835558cf[published Online First: Epub Date]|.

For peer review only - http://bmjopen.bmj.com/site/about/guidelines.xhtml

10. Pepe NS. *The statistical evaluation of medical tests for classification and prediction*. New York: Oxford University Press, 2003.
11. Whiting PF, Rutjes AWS, Westwood ME, et al. QUADAS-2: A Revised Tool for the Quality Assessment of Diagnostic Accuracy Studies. Ann Intern Med 2011;**155**(8):529-36 doi: 10.7326/0003-4819-155-8-201110180-00009[published Online First: Epub Date]|.
12. Harbord RM, Deeks JJ, Egger M, et al. A unification of models for meta-analysis of diagnostic accuracy studies. Biostatistics 2007;**8**(2):239-51 doi: 10.1093/biostatistics/kxl004[published Online First: Epub Date]|.
13. Harbord R, Whiting P. metandi: Meta-analysis of diagnostic accuracy using hierarchical logistic regression. Stata Journal 2009;**9**(2):211-29
14. Bossuyt PM, Davenport C, Deeks JJ, et al. Chapter 11: Interpreting results and drawing conclusions. In: Deeks JJ, Bossuyt PM, Gatsonis C, eds. Cochrane Handbook for Systematic Reviews of Diagnostic Test Accuracy Version 09: The Cochrane Collaboration, 2013.
15. Wallace BC, Schmid CH, Lau J, et al. Meta-Analyst: software for meta-analysis of binary, continuous and diagnostic data. BMC Med Res Methodol 2009;**9**:80 doi: 10.1186/1471-2288-9-80[published Online First: Epub Date]|.
16. Deeks JJ, Macaskill P, Irwig L. The performance of tests of publication bias and other sample size effects in systematic reviews of diagnostic test accuracy was assessed. J Clin Epidemiol 2005;**58**(9):882-93 doi: 10.1016/j.jclinepi.2005.01.016[published Online First: Epub Date]|.
17. Ainsworth MA, Bendtzen K, Brynskov J. Tumor necrosis factor-alpha binding capacity and anti-infliximab antibodies measured by fluid-phase radioimmunoassays as predictors of clinical efficacy of infliximab in Crohn's disease. Am J Gastroenterol 2008;**103**(4):944-8
18. Ben-Bassat O, Romanova A, Iacono A, et al. Association of serum infliximab and antibodies to infliximab to long-term clinical outcome and mucosal healing in crohn's disease. Gastroenterology 2013;**144**(5 Suppl):S-775
19. Bortlik M, Duricova D, Malickova K, et al. Infliximab trough levels may predict sustained response to infliximab in patients with Crohn's disease. Journal of Crohn's & colitis 2013;**7**(9):736-43 doi: http://dx.doi.org/10.1016/j.crohns.2012.10.019[published Online First: Epub Date]|.
20. Hibi T, Sakuraba A, Watanabe M, et al. C-reactive protein is an indicator of serum infliximab level in predicting loss of response in patients with Crohn's disease. J Gastroenterol 2014;**49**(2):254-62 doi: http://dx.doi.org/10.1007/s00535-013-0807-0[published Online First: Epub Date]|.
21. Imaeda H, Andoh A, Fujiyama Y. Development of a new immunoassay for the accurate determination of anti-infliximab antibodies in inflammatory bowel disease. J Gastroenterol 2012;**47**(2):136-43 doi: http://dx.doi.org/10.1007/s00535-011-0474-y[published Online First: Epub Date]|.
22. Kopylov U, Mazor Y, Yavzori M, et al. Clinical utility of antihuman lambda chain-based enzyme-linked immunosorbent assay (ELISA) versus double antigen ELISA for the detection of anti-infliximab antibodies. Inflamm Bowel Dis 2012;**18**(9):1628-33 doi: http://dx.doi.org/10.1002/ibd.21919[published Online First: Epub Date]|.
23. Maser EA, Villela R, Silverberg MS, et al. Association of trough serum infliximab to clinical outcome after scheduled maintenance treatment for Crohn's disease. Clin Gastroenterol Hepatol 2006;**4**(10):1248-54 doi: 10.1016/j.cgh.2006.06.025[published Online First: Epub Date]|.
24. Steenholdt C, Bendtzen K, Brynskov J, et al. Cut-off levels and diagnostic accuracy of infliximab trough levels and anti-infliximab antibodies in Crohn's disease. Scand J Gastroenterol 2011;**46**(3):310-8 doi: http://dx.doi.org/10.3109/00365521.2010.536254[published Online First: Epub Date]|.
25. Steenholdt C, Brynskov J, Thomsen OO, et al. Individualised therapy is more cost-effective than dose intensification in patients with Crohn's disease who lose response to anti-TNF treatment: a randomised, controlled trial. Gut 2014;**63**(6):919-27 doi: http://dx.doi.org/10.1136/gutjnl-2013-305279[published Online First: Epub Date]|.
26. Yanai H, Mlynarsky L, Ron Y, et al. The questionable value of infliximab trough levels during prolonged maintenance therapy. Gastroenterology 2012;**142**(5 Suppl):S788-9
27. Baert F, Drobne D, Gils A, et al. Early trough levels and antibodies to infliximab predict safety and success of reinitiation of infliximab therapy. Clin Gastroenterol Hepatol 2014;**12**(9):1474-81.e2 doi: http://dx.doi.org/10.1016/j.cgh.2014.01.033[published Online First: Epub Date]|.
28. Ben-Horin S, Yavzori M, Katz L, et al. The immunogenic part of infliximab is the F(ab')2, but measuring antibodies to the intact infliximab molecule is more clinically useful. Gut 2011;**60**(1):41-8 doi: http://dx.doi.org/10.1136/gut.2009.201533[published Online First: Epub Date]|.

For peer review only

29. Ben-Horin S, Mazor Y, Yanai H, et al. The decline of anti-drug antibody titres after discontinuation of anti-TNFs: implications for predicting re-induction outcome in IBD. Aliment Pharmacol Ther 2012;**35**(6):714-22 doi: http://dx.doi.org/10.1111/j.1365-2036.2012.04997.x[published Online First: Epub Date]|.
30. Bodini G, Savarino V, Dulbecco P, et al. ELISA vs. HMSA: A comparison between two different methods for the evaluation of adalimumab serum concentration and anti-adalimumab antibodies Preliminary data. Journal of Crohn's and Colitis 2014;**8**:S278
31. Candon S, Mosca A, Ruemmele F, et al. Clinical and biological consequences of immunization to infliximab in pediatric Crohn's disease. Clin Immunol 2006;**118**(1):11-9
32. Dauer RM, Yarur AJ, Abreu MT. Infliximab re-induction outcomes after a failure to treatment. Gastroenterology 2013;**144**(5 Suppl):S-430
33. Farrell RJ, Alsahli M, Jeen YT, et al. Intravenous hydrocortisone premedication reduces antibodies to infliximab in Crohn's disease: a randomized controlled trial. Gastroenterology 2003;**124**(4):917-24 doi: 10.1053/gast.2003.50145[published Online First: Epub Date]|.
34. Hanauer SB, Wagner CL, Bala M, et al. Incidence and importance of antibody responses to infliximab after maintenance or episodic treatment in Crohn's disease. Clin Gastroenterol Hepatol 2004;**2**(7):542-53
35. Kong JY, Bundell CS, Pawlik J, et al. Trough serum infliximab level, anti-infliximab antibody status and response to infliximab maintenance treatment in inflammatory bowel disease (IBD). J Gastroenterol Hepatol 2011;**26**:59-60 doi: http://dx.doi.org/10.1111/j.1440-1746.2011.06824.x[published Online First: Epub Date]|.
36. Marzo M, Armuzzi A, Felice C, et al. Role of trough levels and antibodies to infliximab in the evaluation of loss of response and infusion reactions to infliximab therapy in inflammatory bowel disease. Dig Liver Dis 2014;**46**:S77
37. Nagore D, Ruiz Del Agua A, Pascual J, et al. Therapeutic Cut-off of Infliximab in Patients with Inflammatory Bowel Diseases (TU1325). Gastroenterology 2015;**148**(4 Suppl 1):S-860
38. Pariente B, Pineton de Chambrun G, Krzysiek R, et al. Trough levels and antibodies to infliximab may not predict response to intensification of infliximab therapy in patients with inflammatory bowel disease. Inflamm Bowel Dis 2012;**18**(7):1199-206 doi: http://dx.doi.org/10.1002/ibd.21839[published Online First: Epub Date]|.
39. Steenholdt C, Palarasah Y, Bendtzen K, et al. Pre-existing IgG antibodies cross-reacting with the Fab region of infliximab predict efficacy and safety of infliximab therapy in inflammatory bowel disease. Aliment Pharmacol Ther 2013;**37**(12):1172-83 doi: http://dx.doi.org/10.1111/apt.12330[published Online First: Epub Date]|.
40. Vande Casteele N, Gils A, Singh S, et al. Antibody response to infliximab and its impact on pharmacokinetics can be transient. Am J Gastroenterol 2013;**108**(6):962-71 doi: http://dx.doi.org/10.1038/ajg.2013.12[published Online First: Epub Date]|.
41. Chiu YL, Rubin DT, Vermeire S, et al. Serum adalimumab concentration and clinical remission in patients with Crohn's disease. Inflamm Bowel Dis 2013;**19**(6):1112-22 doi: http://dx.doi.org/10.1097/MIB.0b013e3182813242[published Online First: Epub Date]|.
42. Frederiksen MT, Ainsworth MA, Brynskov J, et al. Antibodies Against Infliximab Are Associated with De Novo Development of Antibodies to Adalimumab and Therapeutic Failure in Infliximab-to-Adalimumab Switchers with IBD. Inflamm Bowel Dis 2014;**20**(10):1714-21 doi: http://dx.doi.org/10.1097/MIB.0000000000000138[published Online First: Epub Date]|.
43. Imaeda H, Takahashi K, Fujimoto T, et al. Clinical utility of newly developed immunoassays for serum concentrations of adalimumab and anti-adalimumab antibodies in patients with Crohn's disease. J Gastroenterol 2014;**49**(1):100-9 doi: http://dx.doi.org/10.1007/s00535-013-0803-4[published Online First: Epub Date]|.
44. Mazor Y, Almog R, Kopylov U, et al. Adalimumab drug and antibody levels as predictors of clinical and laboratory response in patients with Crohn's disease. Aliment Pharmacol Ther 2014;**40**(6):620-8 doi: http://dx.doi.org/10.1111/apt.12869[published Online First: Epub Date]|.
45. Roblin X, Marotte H, Rinaudo M, et al. Association between pharmacokinetics of adalimumab and mucosal healing in patients with inflammatory bowel diseases. Clin Gastroenterol Hepatol 2014;**12**(1):80-84.e2 doi: http://dx.doi.org/10.1016/j.cgh.2013.07.010[published Online First: Epub Date]|.
46. West RL, Zelinkova Z, Wolbink GJ, et al. Immunogenicity negatively influences the outcome of adalimumab treatment in Crohn's disease. Aliment Pharmacol Ther 2008;**28**(9):1122-6 doi: 10.1111/j.1365-2036.2008.03828.x[published Online First: Epub Date]|.

For peer review only

47. Corstjens PL, Fidder HH, Wiesmeijer KC, et al. A rapid assay for on-site monitoring of infliximab trough levels: a feasibility study. Anal Bioanal Chem 2013;**405**(23):7367-75 doi: http://dx.doi.org/10.1007/s00216-013-7154-0[published Online First: Epub Date]|.
48. Steenholdt C, Ainsworth MA, Tovey M, et al. Comparison of techniques for monitoring infliximab and antibodies against infliximab in Crohn's disease. Ther Drug Monit 2013;**35**(4):530-8 doi: http://dx.doi.org/10.1097/FTD.0b013e31828d23c3[published Online First: Epub Date]|.
49. Vande Casteele N, Buurman DJ, Sturkenboom MG, et al. Detection of infliximab levels and anti-infliximab antibodies: a comparison of three different assays. Aliment Pharmacol Ther 2012;**36**(8):765-71 doi: http://dx.doi.org/10.1111/apt.12030[published Online First: Epub Date]|.
50. Wang SL, Ohrmund L, Hauenstein S, et al. Development and validation of a homogeneous mobility shift assay for the measurement of infliximab and antibodies-to-infliximab levels in patient serum. J Immunol Methods 2012;**382**(1-2):177-88 doi: http://dx.doi.org/10.1016/j.jim.2012.06.002[published Online First: Epub Date]|.
51. Steenholdt C, Bendtzen K, Brynskov J, et al. Clinical implications of measuring drug and anti-drug antibodies by different assays when optimizing infliximab treatment failure in Crohn's disease: post hoc analysis of a randomized controlled trial. Am J Gastroenterol 2014;**109**(7):1055-64 doi: http://dx.doi.org/10.1038/ajg.2014.106[published Online First: Epub Date]|.
52. Ruiz-Arguello B, del Agua AR, Torres N, et al. Comparison study of two commercially available methods for the determination of infliximab, adalimumab, etanercept and anti-drug antibody levels. Clin Chem Lab Med 2013;**51**(12):e287-9 doi: 10.1515/cclm-2013-0461[published Online First: Epub Date]|.
53. Daperno M, Frigerio F, Guiotto C, et al. Evaluation of the diagnostic performance of two commercially available tests for infliximab trough levels (IFX-TL) and antibodies to infliximab (ATI) titration in inflammatory bowel disease (IBD). Journal of Crohn's and Colitis 2013;**7**:S213-4
54. Egea-Pujol L, Reddy R, Patel S, et al. Homogenous mobility shift assay (HMSA) overcomes the limitations of elisa and eclia assays for monitoring infliximab (IFX), adalimumab (ADA), and associated anti-drug antibodies in serum. Am J Gastroenterol 2013;**108**:S548 doi: http://dx.doi.org/10.1038/ajg.2013.269[published Online First: Epub Date]|.
55. Eser A, Primas C, Hauenstein S, et al. Comparison of early measurement of infliximab and antibodies-to-infliximab serum levels with standard trough analysis. Gastroenterology 2013;**144**(5 Suppl):S-779
56. Eser A, Primas C, Haunstein S, et al. Detection of anti infliximab antibodies in patients with inflammatory bowel disease (IBD) in the presence of infliximab by homogeneous liquid phase anti infliximab mobility shift assay. Journal of Crohn's and Colitis 2013;**7**:S231-2
57. Greathead L, Kelleher P, Steel A. Development and validation of ELISA to measure serum anti TNFa levels. Journal of Crohn's and Colitis 2014;**8**:S97-8
58. Hauenstein S, Ohrmund L, Salbato J, et al. Comparison of homogeneous mobility shift assay and solid phase elisa for the measurement of drug and anti-drug antibody (ADA) levels in serum from patients treated with anti-TNF biologics. Gastroenterology 2012;**142**(5 Suppl):S-538
59. McTigue M, Sandborn W, Levesque B, et al. Clinical utility of next generation infliximab and antibodies to infliximab assay. Am J Gastroenterol 2013;**108**:S527 doi: http://dx.doi.org/10.1038/ajg.2013.269[published Online First: Epub Date]|.
60. Semmler J, Pilch A, Armbruster F, et al. Development of a new immunoassay for the accurate determination of anti-infliximab antibodies in inflammatory bowel disease. Clin Chem Lab Med 2013;**51 (10)**:eA27-8 doi: http://dx.doi.org/10.1515/cclm-2013-0737[published Online First: Epub Date]|.
61. Ungar B, Anafy A, Kopylov U, et al. The clinical and immunological significance of low level of infliximab in the absence of anti-infliximab antibodies in patients with IBD. Gastroenterology 2014;**146**(5 Suppl):S-245 doi: http://dx.doi.org/10.1016/S0016-5085%2814%2960862-3[published Online First: Epub Date]|.
62. Vande Casteele N, Peeters M, Compernolle G, et al. TNF-responsive cellular based assay reveals neutralizing capacity of anti-adalimumab antibodies in crohn's disease and ulcerative colitis patients. Gastroenterology 2014;**146**(5 Suppl):S-242 doi: http://dx.doi.org/10.1016/S0016-5085%2814%2960852-0[published Online First: Epub Date]|.
63. Wang SL, Ohrmund L, Singh S. Measurement of human anti-chimeric antibodies (Haca) and infliximab levels in patient serum using a novel homogeneous assay. Gastroenterology 2010;**1)**:S684-5
64. Schatz SB, Prell C, Freudenberg F, et al. PA-G-0035 Comparison of different tests for determination of infliximab levels and antibodies against infliximab in pediatric IBD patients. The 46th Annual Meeting of The European Society of Paediatric Gastroenterology, Hepatology and Nutrition. J Pediatr Gastroenterol Nutr 2013;**56 suppl 2**:19

65. Wang SL, Ohrmund L, Hauenstein S, et al. Evaluation of a novel homogeneous mobility shift assay for the measurement of human antibodies-To-Infliximab and infliximab levels in Patient serum. Am J Gastroenterol 2011;**106**:S475-6 doi: http://dx.doi.org/10.1038/ajg.2011.336_9[published Online First: Epub Date]|.
66. Ferrante di Ruffano L, Hyde CJ, McCaffery KJ, et al. Assessing the value of diagnostic tests: a framework for designing and evaluating trials. BMJ 2012;**344**:e686 doi: 10.1136/bmj.e686[published Online First: Epub Date]|.
67. Vande Casteele N, Ferrante M, Van Assche G, et al. Trough Concentrations of Infliximab Guide Dosing for Patients with Inflammatory Bowel Disease. Gastroenterology Forthcoming 2015 doi: 10.1053/j.gastro.2015.02.031[published Online First: Epub Date]|.
68. Cornillie F, Hanauer SB, Diamond RH, et al. Postinduction serum infliximab trough level and decrease of C-reactive protein level are associated with durable sustained response to infliximab: a retrospective analysis of the ACCENT I trial. Gut 2014;**63**(11):1721-7 doi: http://dx.doi.org/10.1136/gutjnl-2012-304094[published Online First: Epub Date]|.
69. Goldberg R, Beswick L, Van Langenberg D, et al. Predictors of sub-therapeutic infliximab or adalimumab trough levels and anti-drug antibodies and their influence on therapeutic decisions. Journal of Crohn's and Colitis 2014;**8**:S223
70. Imaeda H, Bamba S, Takahashi K, et al. Relationship between serum infliximab trough levels and endoscopic activities in patients with Crohn's disease under scheduled maintenance treatment. J Gastroenterol 2014;**49**(4):674-82 doi: http://dx.doi.org/10.1007/s00535-013-0829-7[published Online First: Epub Date]|.
71. Marits P, Landucci L, Sundin U, et al. Trough s-infliximab and antibodies towards infliximab in a cohort of 79 IBD patients with maintenance infliximab treatment. Journal of Crohn's & colitis 2014;**8**(8):881-9 doi: http://dx.doi.org/10.1016/j.crohns.2014.01.009[published Online First: Epub Date]|.
72. Pallagi-Kunstar E, Farkas K, Szepes Z, et al. Utility of serum TNF-alpha, infliximab trough level, and antibody titers in inflammatory bowel disease. World J Gastroenterol 2014;**20**(17):5031-5 doi: http://dx.doi.org/10.3748/wjg.v20.i17.5031[published Online First: Epub Date]|.
73. Paul S, Tedesco ED, Marotte H, et al. Interest of the dosage of serum concentration of infliximab and antibodies anti infliximab in the therapeutic response under infliximab in IBD. Gastroenterology 2012;**142**(5 Suppl):S354
74. Paul S, Del Tedesco E, Marotte H, et al. Therapeutic drug monitoring of infliximab and mucosal healing in inflammatory bowel disease: a prospective study. Inflamm Bowel Dis 2013;**19**(12):2568-76 doi: http://dx.doi.org/10.1097/MIB.0b013e3182a77b41[published Online First: Epub Date]|.
75. Singh N, Rosenthal CJ, Melmed GY, et al. Early infliximab trough levels are associated with persistent remission in pediatric patients with inflammatory bowel disease. Inflamm Bowel Dis 2014;**20**(10):1708-13 doi: http://dx.doi.org/10.1097/MIB.0000000000000137[published Online First: Epub Date]|.
76. Levesque BG, Greenberg GR, Zou G, et al. A prospective cohort study to determine the relationship between serum infliximab concentration and efficacy in patients with luminal Crohn's disease. Aliment Pharmacol Ther 2014;**39**(10):1126-35 doi: http://dx.doi.org/10.1111/apt.12733[published Online First: Epub Date]|.
77. Feagan BG, Singh S, Lockton S, et al. Novel infliximab (IFX) and antibody-to-infliximab (ATI) assays are predictive of disease activity in patients with crohn's disease (CD). Gastroenterology 2012;**142**(5 Suppl):S-114
78. Karmiris K, Paintaud G, Noman M, et al. Influence of trough serum levels and immunogenicity on long-term outcome of adalimumab therapy in Crohn's disease. Gastroenterology 2009;**137**(5):1628-40 doi: http://dx.doi.org/10.1053/j.gastro.2009.07.062[published Online First: Epub Date]|.
79. Ward MG, Kariyawasam VC, Mogan SB, et al. Clinical utility of measuring adalimumab trough levels and antibodies to adalimumab in patients with inflammatory bowel diseases. J Gastroenterol Hepatol 2013;**28**:100-01 doi: http://dx.doi.org/10.1111/jgh.12365-6[published Online First: Epub Date]|.
80. Yarur AJ, Deshpande AR, Sussman DA, et al. Serum adalimumab levels and antibodies correlate with endoscopic intestinal inflammation and inflammatory markers in patients with inflammatory bowel disease. Gastroenterology 2013;**144**(5 Suppl):S774-5
81. Mazor Y, Kopylov U, Hur DB, et al. Evaluating adalimumab drug and antibody levels as predictors of clinical and laboratory response in crohn's disease patients. Gastroenterology 2013;**144**(5 Suppl):S-778
82. Harbord R, Harris RJ, Sterne JAC. Updated tests for small-study effects in meta-analyses. Stata Journal 2009;**9**(2):197-210

For peer review only - http://bmjopen.bmj.com/site/about/guidelines.xhtml

83. Macaskill P, Gatsonis C, Deeks J, et al. Chapter 10: Analysing and Presenting Results. In: Deeks JJ, Bossuyt PM, Gatsonis C, eds. Cochrane Handbook for Systematic Reviews of Diagnostic Test Accuracy Version 10: The Cochrane Collaboration, 2010.

For peer review only

Figure legend

Figure 1 PRISMA study flow diagram

Figure 2 Anti-TNF antibody levels for predicting loss of response or failure to regain response

Figure 3 Trough anti-TNF levels for predicting loss of response or failure to regain response

Figure 4 Hierarchical meta-analysis of trough Infliximab levels for predicting loss of response or failure to regain response

Figure 5 Hierarchical meta-analysis of trough levels of antibodies to Infliximab for predicting loss of response or failure to regain response

Figure 6 Positive and negative predictive values according to prevalence of lack of response using the pooled summary ROC model estimates of sensitivity and specificity

For peer review only - http://bmjopen.bmj.com/site/about/guidelines.xhtml

For peer review only

For peer review only - http://bmjopen.bmj.com/site/about/guidelines.xhtml

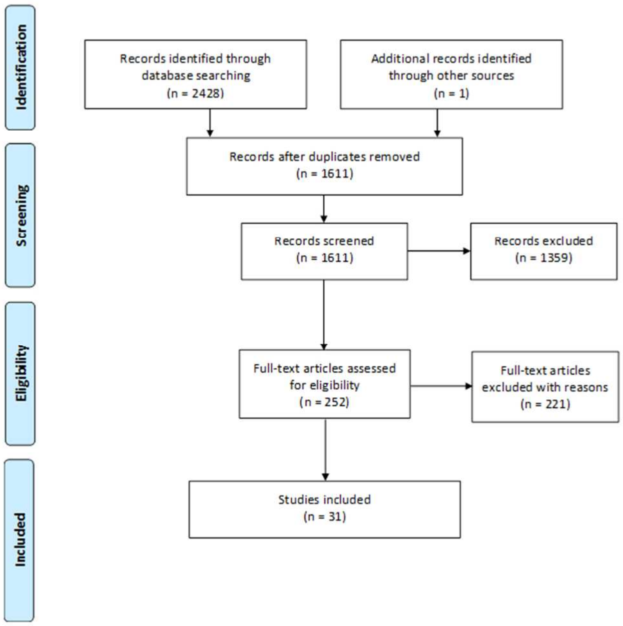

Figure 1 PRISMA study flow diagram

254x190mm (96 x 96 DPI)

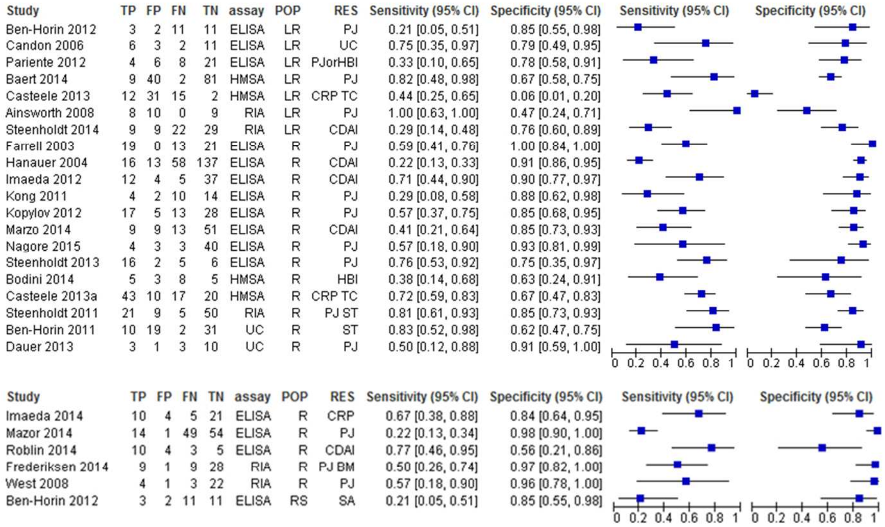

Figure 2 Anti-TNF antibody levels for predicting loss of response or failure to regain response

254x190mm (96 x 96 DPI)

Study	TP	FP	FN	TN	assay	POP	RES	Sensitivity (95% CI)	Specificity (95% CI)
Ainsworth 2008	7	1	1	18	RIA	LR	PJ	0.88 [0.47, 1.00]	0.95 [0.74, 1.00]
Steenholdt 2014	10	7	21	31	RIA	LR	CDAI	0.32 [0.17, 0.51]	0.82 [0.66, 0.92]
Bortlik 2013	16	23	7	38	ELISA	R	PJ	0.70 [0.47, 0.87]	0.62 [0.49, 0.74]
Cornillie 2014	14	11	8	38	ELISA	R	CDAI	0.64 [0.41, 0.83]	0.78 [0.63, 0.88]
Hibi 2014	8	4	7	22	ELISA	R	CDAI	0.53 [0.27, 0.79]	0.85 [0.65, 0.96]
Imaeda 2012	9	8	8	33	ELISA	R	CDAI	0.53 [0.28, 0.77]	0.80 [0.65, 0.91]
Kopylov 2012	21	2	9	31	ELISA	R	PJ	0.70 [0.51, 0.85]	0.94 [0.80, 0.99]
Yanai 2012	7	10	7	16	ELISA	R	PJ	0.50 [0.23, 0.77]	0.62 [0.41, 0.80]
Ben-Bassat 2013	50	29	10	145	HMSA	R	HBI	0.83 [0.71, 0.92]	0.83 [0.77, 0.89]
Steenholdt 2011	18	7	3	41	RIA	R	PJ	0.86 [0.64, 0.97]	0.85 [0.72, 0.94]
Maser 2006	22	11	13	44	UC	R	HBI	0.63 [0.45, 0.79]	0.80 [0.67, 0.90]

Study	TP	FP	FN	TN	assay	POP	RES	Sensitivity (95% CI)	Specificity (95% CI)
Chiu 2013	7	7	56	98	ELISA	LR	PJ CRP	0.11 [0.05, 0.22]	0.93 [0.87, 0.97]
Imaeda 2014	10	2	5	23	ELISA	R	CRP	0.67 [0.38, 0.88]	0.92 [0.74, 0.99]
Mazor 2014	48	16	20	34	ELISA	R	PJ CRP	0.71 [0.58, 0.81]	0.68 [0.53, 0.80]
Roblin 2014	16	2	8	14	ELISA	R	MH	0.67 [0.45, 0.84]	0.88 [0.62, 0.98]
Frederiksen 2014	12	9	6	20	RIA	R	PJ BM	0.67 [0.41, 0.87]	0.69 [0.49, 0.85]

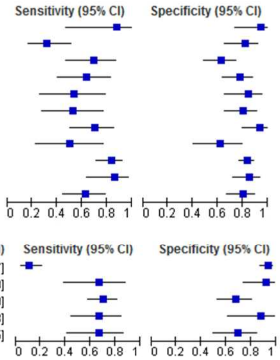

Figure 3 Trough anti-TNF levels for predicting loss of response or failure to regain response

254x190mm (96 x 96 DPI)

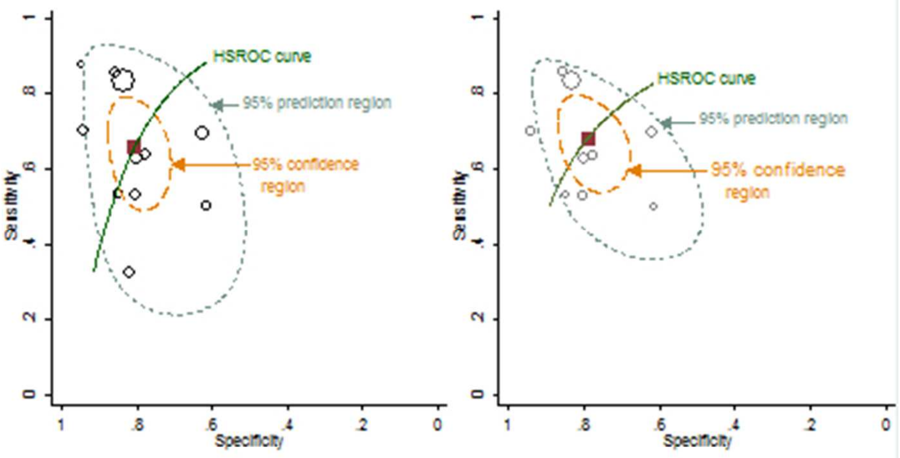

Figure 4 Hierarchical meta-analysis of trough Infliximab levels for predicting loss of response or failure to regain response

158x114mm (72 x 72 DPI)

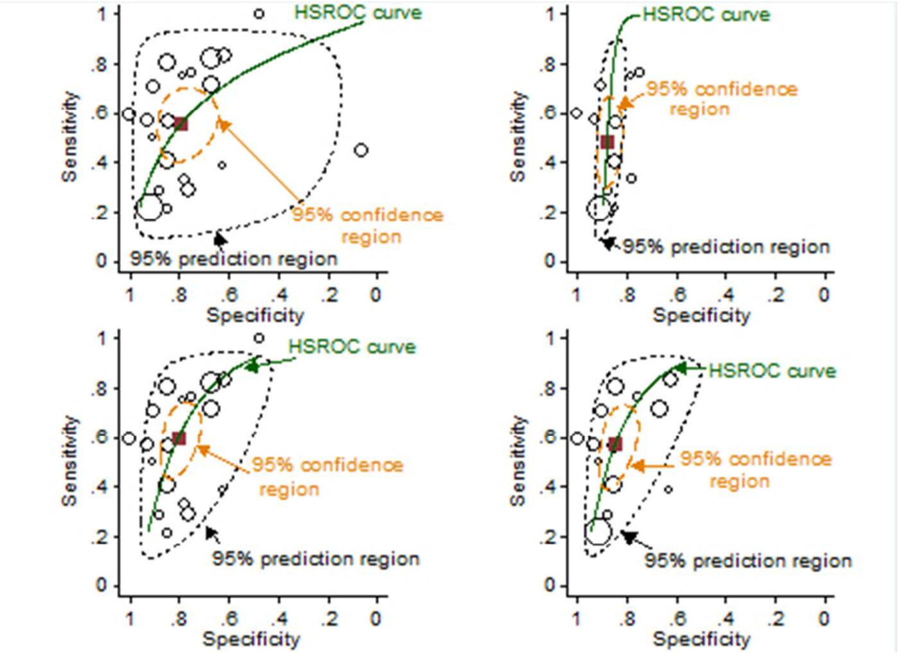

Figure 5 Hierarchical meta-analysis of trough levels of antibodies to Infliximab for predicting loss of response or failure to regain response

158x114mm (72 x 72 DPI)

For peer review only - http://bmjopen.bmj.com/site/about/guidelines.xhtml

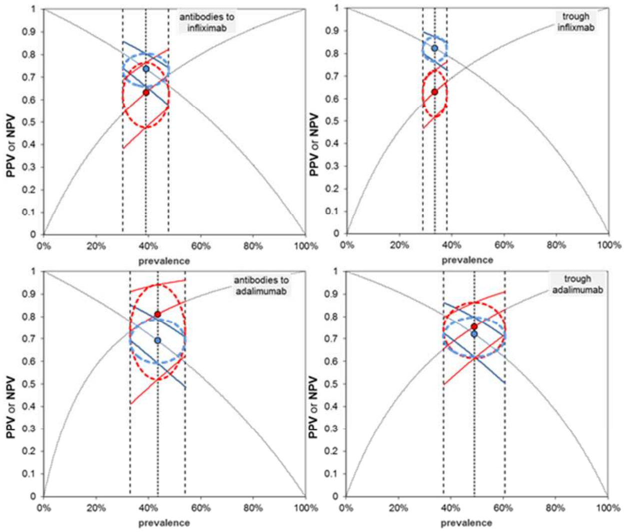

Figure 6 Positive and negative predictive values according to prevalence of lack of response using the pooled summary ROC model estimates of sensitivity and specificity

254x190mm (96 x 96 DPI)

Supplement 1 Search strategy

10	anti* drug* antibod*.tw.	469
11	ADAb.tw.	44
12	*drug antibody/	1528
13	1 or 2 or 3 or 4 or 5 or 6 or 7 or 8 or 9 or 10 or 11 or 12	35630
14	lisa* tracker*.tw.	11
15	(immundiagnostik* or immunodiagnostik* or immunediagnostik*).tw.	74
16	(proteomika* or promonitor*).tw.	27
17	*enzyme linked immunosorbent assay/	14622
18	enzyme* link* immunoassay*.tw.	3275
19	enzyme* link* immuno* assay*.tw.	71923
20	ELISA*.tw.	166866
21	14 or 15 or 16 or 17 or 18 or 19 or 20	207373
22	*radioimmunoassay/	17240
23	(radioimmuno* or radio immuno* or radio-immuno*).tw.	74895
24	RIA.tw.	20769
25	reporter* gene* assay*.tw.	4396
26	RGA.tw.	400
27	semi* fluid* phase* enzyme* immuno*.tw.	1
28	EIA.tw.	10836
29	((homogenous* or homogeneous*) adj1 mobilit* shift* assay*).tw.	39
30	HMSA.tw.	98
31	(Biomonitor* or iLite).tw.	5664
32	(Matriks* Biotek* or Shikari*).tw.	13
33	(Prometheus* or Anser IFX or Anser ADA).tw.	568
34	22 or 23 or 24 or 25 or 26 or 27 or 28 or 29 or 30 or 31 or 32 or 33	113752

For peer review only

35	((monitor* or pharmacokinetic* or measur* or level* or concentration*) adj3 (adalimumab or ADA or infliximab or IFX or Anti-TNF* or Anti-Tumour Necrosis Factor*)).tw.	2016
36	*crohn disease/	34280
37	crohn*.tw.	50039
38	inflammator* bowel* disease*.tw.	41418
39	IBD.tw.	23266
40	36 or 37 or 38 or 39	82551
41	(((monitor* or pharmacokinetic* or measur* or level* or concentration*) adj3 (adalimumab or infliximab or Anti-TNF* or AntiTNF* or Anti-Tumour Necrosis Factor*)) and (correlat* or associat* or test performance)).tw.	544
42	13 and 21 and 40	278
43	13 and 34 and 40	109
44	35 and 40	507
45	41 or 42 or 43 or 44	938
46	nonhuman/ not human/	3490973
47	45 not 46	917

Cochrane Library (Wiley), searched on 22/10/2014

#1	adalimumab:ti,ab,kw	451
#2	ADA:ti,ab	237
#3	infliximab:ti,ab,kw	767
#4	IFX:ti,ab	39
#5	((anti-TNF* or antiTNF* or TNF*) near/2 inhibitor*):ti,ab,kw	106
#6	(anti* next tumo*r* next necrosis* next factor*):ti,ab,kw	256
#7	MeSH descriptor: [Tumor Necrosis Factor-alpha] this term only	2408
#8	MeSH descriptor: [Antibodies, Monoclonal] this term only	3978
#9	#7 and #8	409

For peer review only

#10	(anti* next drug* next antibod*):ti,ab,kw	19
#11	(ADAb):ti,ab,kw	0
#12	#1 or #2 or #3 or #4 or #5 or #6 or #7 or #8 or #9 or #10 or #11	6714
#13	(lisa* next tracker*):ti,ab,kw	0
#14	(immundiagnostik* or immunodiagnostik* or immunediagnostik*):ti,ab,kw	0
#15	(proteomika* or promonitor*):ti,ab,kw	0
#16	MeSH descriptor: [Enzyme-Linked Immunosorbent Assay] explode all trees	2122
#17	(enzyme* next link* next immunoassay*):ti,ab,kw	84
#18	ELISA*:ti,ab,kw	2534
#19	#13 or #14 or #15 or #16 or #17 or #18	3958
#20	MeSH descriptor: [Radioimmunoassay] explode all trees	1176
#21	(radioimmuno* or radio next immuno* or radio-immuno*):ti,ab,kw	2761
#22	RIA:ti,ab	570
#23	(reporter* next gene* next assay*):ti,ab,kw	11
#24	RGA:ti,ab	8
#25	(semi* next fluid* next phase* next enzyme* next immuno*):ti,ab,kw	0
#26	EIA:ti,ab	339
#27	((homogenous* or homogeneous*) near/1 (mobilit* next shift* next assay*)):ti,ab,kw	1
#28	HMSA:ti,ab	1
#29	(Biomonitor* or iLite):ti,ab,kw	14
#30	(Matriks* next Biotek* or Shikari*):ti,ab,kw	0
#31	(Prometheus* or Anser next IFX or Anser next ADA):ti,ab,kw	23
#32	#20 or #21 or #22 or #23 or #24 or #25 or #26 or #27 or #28 or #29 or #30 or #31	3651
#33	((monitor* or pharmacokinetic* or measur* or level* or concentration*) near/3 (adalimumab or ADA or infliximab or IFX or Anti-TNF* or Anti-Tumour next Necrosis next Factor*)):ti,ab,kw	83

For peer review only

#34	MeSH descriptor: [Inflammatory Bowel Diseases] this term only	273
#35	MeSH descriptor: [Crohn Disease] this term only	997
#36	crohn*:ti,ab,kw	1512
#37	(inflammator* next bowel* next disease*):ti,ab,kw	798
#38	IBD:ti,ab	271
#39	#34 or #35 or #36 or #37 or #38	2037
#40	(((monitor* or pharmacokinetic* or measur* or level* or concentration*) near/3 (adalimumab or infliximab or Anti-TNF* or AntiTNF* or Anti-Tumour next Necrosis next Factor*)) and (correlat* or associat* or test next performance)):ti,ab,kw	33
#41	#12 and #19 and #39	8
#42	#12 and #32 and #39	1
#43	#33 and #39	18
#44	#40 or #41 or #42 or #43	49

All Results (49)

Cochrane Reviews (0)

All Review Protocol

Other Reviews (1)

Trials (47)

Methods Studies (0)

Technology Assessments (1)

Economic Evaluations (0)

Cochrane Groups (0)

Science Citation Index and Conference Proceedings – Science (Web of Science), searched on 22/10/2014

# 40	806	#39 OR #38 OR #37 OR #36<br>Indexes=SCI-EXPANDED, CPCI-S Timespan=All years
# 39	324	#35 AND #32<br>Indexes=SCI-EXPANDED, CPCI-S Timespan=All years
# 38	26	#35 AND #31 AND #9

		Indexes=SCI-EXPANDED, CPCI-S Timespan=All years
# 37	128	#35 AND #16 AND #9<br>Indexes=SCI-EXPANDED, CPCI-S Timespan=All years
# 36	539	TS=(((monitor* or pharmacokinetic* or measur* or level* or concentration*) near/3 (adalimumab or ADA or infliximab or IFX or Anti-TNF* or ("Anti-Tumour Necrosis" near/1 Factor*))) and (correlat* or associat* or "test performance"))<br>Indexes=SCI-EXPANDED, CPCI-S Timespan=All years
# 35	80,743	#34 OR #33<br>Indexes=SCI-EXPANDED, CPCI-S Timespan=All years
# 34	53,142	TS=(((inflammator* near/1 bowel*) near/1 disease*) or IBD)<br>Indexes=SCI-EXPANDED, CPCI-S Timespan=All years
# 33	50,398	TS=crohn*<br>Indexes=SCI-EXPANDED, CPCI-S Timespan=All years
# 32	1,366	TS=((monitor* or pharmacokinetic* or measur* or level* or concentration*) near/3 (adalimumab or ADA or infliximab or IFX or Anti-TNF* or ("Anti-Tumour Necrosis" near/1 Factor*)))<br>Indexes=SCI-EXPANDED, CPCI-S Timespan=All years
# 31	79,288	#30 OR #29 OR #28 OR #27 OR #26 OR #25 OR #24 OR #23 OR #22 OR #21 OR #20 OR #19 OR #18 OR #17<br>Indexes=SCI-EXPANDED, CPCI-S Timespan=All years
# 30	713	TS=(Prometheus* or "Anser IFX" or "Anser ADA")<br>Indexes=SCI-EXPANDED, CPCI-S Timespan=All years
# 29	10	TS=((Matriks* near/1 Biotek*) or Shikari*)<br>Indexes=SCI-EXPANDED, CPCI-S Timespan=All years
# 28	8,841	TS=(Biomonitor* or iLite)<br>Indexes=SCI-EXPANDED, CPCI-S Timespan=All years
# 27	107	TS=HMSA<br>Indexes=SCI-EXPANDED, CPCI-S Timespan=All years
# 26	11	TS=((homogenous* or homogeneous*) near/1 (mobilit* near/1 (shift* near/1 assay*)))<br>Indexes=SCI-EXPANDED, CPCI-S Timespan=All years
# 25	8,832	TS=EIA<br>Indexes=SCI-EXPANDED, CPCI-S Timespan=All years
# 24	1	TS=((semi* near/1 fluid*) near/3 (enzyme* near/1 immuno*))<br>Indexes=SCI-EXPANDED, CPCI-S Timespan=All years

For peer review only

# 23	0	TS=((semi* near/1 fluid*) near/2 (enzyme* near/1 immuno*))<br>Indexes=SCI-EXPANDED, CPCI-S Timespan=All years
# 22	0	TS=(semi* near/1 fluid* near/1 phase* near/1 enzyme* near/1 immuno*)<br>Indexes=SCI-EXPANDED, CPCI-S Timespan=All years
# 21	0	TS=(((semi* near/1 fluid*) near/1 phase*) near/1 (enzyme* near/1 immuno*))<br>Indexes=SCI-EXPANDED, CPCI-S Timespan=All years
# 20	1,230	TS=RGA<br>Indexes=SCI-EXPANDED, CPCI-S Timespan=All years
# 19	4,518	TS=(reporter* near/1 gene* near/1 assay*)<br>Indexes=SCI-EXPANDED, CPCI-S Timespan=All years
# 18	12,773	TS=RIA<br>Indexes=SCI-EXPANDED, CPCI-S Timespan=All years
# 17	46,937	TS=(radioimmuno* or (radio near/1 immuno*) or radio-immuno*)<br>Indexes=SCI-EXPANDED, CPCI-S Timespan=All years
# 16	146,389	#15 OR #14 OR #13 OR #12 OR #11 OR #10<br>Indexes=SCI-EXPANDED, CPCI-S Timespan=All years
# 15	113,120	TS=ELISA*<br>Indexes=SCI-EXPANDED, CPCI-S Timespan=All years
# 14	60,666	TS=((enzyme* near/1 link*) near/1 (immuno* near/1 assay))<br>Indexes=SCI-EXPANDED, CPCI-S Timespan=All years
# 13	2,850	TS=((enzyme* near/1 link*) near/1 immunoassay*)<br>Indexes=SCI-EXPANDED, CPCI-S Timespan=All years
# 12	1	TS=(proteomika* or promonitor*)<br>Indexes=SCI-EXPANDED, CPCI-S Timespan=All years
# 11	9	TS=(immundiagnostik* or immunodiagnostik* or immunediagnostik*)<br>Indexes=SCI-EXPANDED, CPCI-S Timespan=All years
# 10	0	TS=(lisa* near/1 tracker*)<br>Indexes=SCI-EXPANDED, CPCI-S Timespan=All years
# 9	32,262	#8 OR #7 OR #6 OR #5 OR #4 OR #3 OR #2 OR #1<br>Indexes=SCI-EXPANDED, CPCI-S Timespan=All years
# 8	35	TS=ADAb<br>Indexes=SCI-EXPANDED, CPCI-S Timespan=All years
# 7	2,534	TS=((anti* near/1 drug*) near/1 antibod*)<br>Indexes=SCI-EXPANDED, CPCI-S Timespan=All years

For peer review only - http://bmjopen.bmj.com/site/about/guidelines.xhtml

# 6	4,072	TS=((anti* near/1 tumo$r*) near/1 (necrosis* near/1 factor*)) Indexes=SCI-EXPANDED, CPCI-S Timespan=All years
# 5	4,065	TS=((anti-TNF* or antiTNF* or TNF*) near/2 inhibitor*) Indexes=SCI-EXPANDED, CPCI-S Timespan=All years
# 4	373	TS=IFX Indexes=SCI-EXPANDED, CPCI-S Timespan=All years
# 3	13,729	TS=infliximab Indexes=SCI-EXPANDED, CPCI-S Timespan=All years
# 2	8,006	TS=ADA Indexes=SCI-EXPANDED, CPCI-S Timespan=All years
# 1	4,973	TS=adalimumab Indexes=SCI-EXPANDED, CPCI-S Timespan=All years

Index to Theses, searched on 28/10/2014

((adalimumab or infliximab or AntiTNF* or Anti-TNF* or "Anti TNF" or "Anti TNFa" or "Anti TNFalpha" or (TNF* w/2 inhibitor*) or (Anti-Tum*r w/2 Necrosis) or ("anti drug" w/2 antibod*) or ADAb) AND (crohn* or "inflammatory bowel disease" or IBD))

14 document(s) retrieved

(((adalimumab or infliximab or AntiTNF* or Anti-TNF* or "Anti TNF" or "Anti TNFa" or "Anti TNFalpha" or (TNF* w/2 inhibitor*) or (Anti-Tum*r w/2 Necrosis) or "anti drug antibody" or "anti drug antibodies" or "anti-drug antibody" or "anti-drug antibodies" or ADAb) w/10 (monitor or monitoring or monitors or monitored or pharmacokinetic or pharmacokinetics or measure or measures or measurement or measuring or level or levels or concentration or concentrations)) AND ((correlate* or correlation* or associate* or association* or "test performance")))

4 document(s) retrieved

DART-Europe, searched on 28/10/2014

(adalimumab or infliximab or AntiTNF* or Anti-TNF* or "Anti TNF" or "Anti TNFa" or "Anti TNFalpha" or (TNF* and inhibitor*) or (Anti-Tum*r and Necrosis) or ("anti drug" and antibod*) or ADAb) and (crohn* or "inflammatory bowel disease" or "inflammatory bowel diseases" or IBD)

113 document(s) retrieved

Dissertations and Theses, searched on 29/10/2014

all(((adalimumab or infliximab or AntiTNF* or Anti-TNF* or "Anti TNF" or "Anti TNFa" or "Anti TNFalpha" or (TNF* n/2 inhibitor*) or (Anti-Tum*r n/2 Necrosis) or ("anti drug" n/2 antibod*) or ADAb) AND (crohn* or "inflammatory bowel disease" or "inflammatory bowel diseases" or IBD)))

21

For peer review only

all(((adalimumab or infliximab or AntiTNF* or Anti-TNF* or "Anti TNF" or "Anti TNFa" or "Anti TNFalpha" or (TNF* n/2 inhibitor*) or (Anti-Tum*r n/2 Necrosis) or "anti drug antibody" or "anti drug antibodies" or "anti-drug antibody" or "anti-drug antibodies" or ADAb) n/10 (monitor or monitoring or monitors or monitored or pharmacokinetic or pharmacokinetics or measure or measures or measurement or measuring or level or levels or concentration or concentrations)) and (correlate* or correlation* or associate* or association* or "test performance"))

15

NIHR HTA Programme, searched on 29/10/2014

adalimumab

16

infliximab

23

TNF

17

PROSPERO, searched on 29/10/2014

adalimumab in All fields

OR

infliximab in All fields

OR

TNF* inhibitor* in All fields

OR

AntiTNF* in All fields

OR

Anti-TNF* in All fields

29 records

ClinicalTrials.gov, searched on 04/11/2014

Search Terms (any field): adalimumab OR infliximab OR (TNF AND (anti OR inhibitor OR blocker)) OR "anti drug antibody" OR "anti drug antibodies" OR ADAb

AND

Condition: crohn OR "inflammatory bowel disease" OR "inflammatory bowel diseases"

AND

Title: monitor OR pharmacokinetic OR measure OR measuring OR level OR concentration OR assay

14 studies

Current Controlled Trials, searched on 04/11/2014

For peer review only

(adalimumab OR infliximab OR TNF* OR AntiTNF* OR Anti-TNF* OR anti drug antibod* OR ADAb) AND (crohn* OR inflammatory bowel disease*) AND (monitor* OR pharmacokinetic* OR measure* OR measuring OR level* OR concentration* OR assay*)

30 studies

UKCRN Portfolio Database, searched on 04/11/2014

Specialty: Gastroenterology

Research Summary: adalimumab infliximab TNF AntiTNF Anti-TNF ADAb

'Any' selected (combines terms with Boolean OR)

4 studies

WHO ICTRP, searched on 10/11/2014

Advanced Search

In Title: adalimumab OR infliximab OR AntiTNF* OR Anti-TNF* OR TNF inhibitor* OR TNFα inhibitor* OR TNF alpha inhibitor* OR TNFalpha inhibitor* OR anti drug antibody OR anti drug antibodies OR ADAb

AND

In Condition: Crohn* OR inflammatory bowel disease*

AND

In Intervention: monitor* OR pharmacokinetic* OR measure* OR measuring OR level* OR concentration* OR assay*

39 trials found

For peer review only

Supplement 2 Drug cut-offs for predicting loss of or lack of regaining response

Table S1 Drug cut-offs defined by ROC analysis in included studies using drug level as predictor of loss of or lack of regaining response (by assay type and drug)

Reference	Cut-off in µg/ml	Performance measures				AUC (95% CI)	Clinical marker	Drug	Assay
		Sens	Spec	PPV	NPV				
Bortlik 2013[19]	3	0.70	0.62	0.41	0.84	0.70 (0.57-0.83)	Sustained response (no treatment failure or drug intolerance, no surgery, IS introduction, steroids or Infliximab increase)	IFX	ELISA
Cornillie 2014[68]	3.5	0.64	0.78	0.56	0.83	0.75	Sustained response (CDAI score change)	IFX	ELISA
Steenholdt 2011[24]	0.5<br>2.2 (TL week 14)	0.86<br>0.79	0.85<br>0.94	NR	NR	0.93 (0.85-1.0)<br>0.93 (SE 0.04)	Maintained response (good response to induction therapy at 0, 2 and 6 weeks followed by good response to maintenance therapy)	IFX	RIA
Chiu 2013[41]	No Adalimumab concentration identified associated with clinical remission at any time point so clinical utility of measuring Adalimumab concentrations was difficult to assess	NR	NR	NR	NR	Week 4: 0.51<br>Week 24: 0.58<br>Week 56: 0.57	Clinical remission (CDAI <150)	ADA	ELISA
Imaeda 2014[43]	5.9	0.67	0.92	NR	NR	0.83 (0.80-0.95)	CRP ≤0.3mg/dL	ADA	ELISA
Mazor 2014[44]	5.85	0.68	0.71	NR	NR	0.75 (0.66-0.84)	Remission according to 2 physicians' assessment	ADA	ELISA
Roblin 2014[45]	4.85<br>4.9	0.81<br>0.66	0.67<br>0.85	0.84<br>0.88	0.57<br>0.51	0.73<br>0.77	Clinical remission (CDAI <150)<br>MH (disappearance of all ulcerations on endoscopy)	ADA	ELISA
Frederiksen 2014[42]	14.5<br>0.35<br>6.85	1.00<br>0.50<br>0.69	0.12<br>0.96<br>0.69	0.41<br>0.89<br>0.58	1.00<br>0.76<br>0.78	0.77 (0.62-0.93)	LOR (physician's global assessment)	ADA	RIA

For peer review only

Table S2 Drug cut-offs in included studies not reporting a ROC analysis and using drug level as predictor of loss of or lack of regaining response (by assay type)

**Reference**	**Cut-off in µg/ml**	**Source of cut-off**	**Drug**	**Assay**
Hibi 2014[20]	1	Maser 2006[23]	IFX	ELISA
Imaeda 2012[21]	0.66	95th percentile value from 35 patients who had never received Infliximab	IFX	ELISA
Kopylov 2012[22]	Unclear	Unclear	IFX	ELISA
Maser 2006[23]	1.4	Unclear	IFX	ELISA
Yanai 2012[26] abstract	1	Unclear	IFX	ELISA
Ben Bassat 2013[18] abstract	2	Derived from data not pre-specified	IFX	HMSA
Ainsworth 2008[17]	0.5	Derived from data not pre-specified	IFX	RIA
Steenholdt 2014[25]	0.5	Steenholdt 2011[24]	IFX	RIA

Table S3 Additional studies reporting drug cut-offs derived by ROC analysis but not reporting sufficient 2x2 data for using drug level as predictor of loss of or lack of regaining response (by assay type and drug)

**Reference**	**Cut-off in µg/ml**	**Performance measures**				**AUC (95% CI)**	**Clinical marker**	**Drug**	**Assay**
		**Sens**	**Spec**	**PPV**	**NPV**				
Goldberg 2014[69] Abstract	3	0.90	0.37	NR	NR	0.75	Disease activity (physicians global assessment and CRP levels)	IFX	ELISA
Imaeda 2014[70]	0.6<br>1.0<br>1.1<br>4.0	0.73<br>0.67<br>0.72<br>0.71	0.62<br>0.71<br>0.56<br>0.70	NR<br>NR<br>NR<br>NR	NR<br>NR<br>NR<br>NR	0.67 (0.60-0.81)<br>0.72 (0.50-0.73)<br>0.63 (0.55-0.65)<br>0.63 (0.56-0.70)	CRP ≤0.3mg/dL<br>Serum albumin (≥ 4.0mg/dL)<br>FC (≤ 300µg/g)<br>MH (Rutgeerts scoring system 0 or 1)	IFX	ELISA
Marits 2014[71]	4.1	0.87	0.44	NR	NR	0.74 (SE 0.037)	Remission (HBI <5 and CRP < 3 mg/l)	IFX	ELISA
Nagore 2015[37]	0.8	0.86	0.75	NR	NR	0.86 (0.76-0.96)	Active disease	IFX	ELISA (Promonitor)
Pallagi-Kunstar 2014[72]	3.01	NR	NR	NR	NR	NR	Detecting anti-drug antibodies	IFX	ELISA
Paul 2012[73] abstract	2	0.76	0.82	NR	NR	0.60	Remission (CDAI score <150)	IFX	ELISA
Paul 2013[74]	0.5 (trough after optimisation minus trough before	0.88	0.76	0.78	0.86	0.91 (0.83-1.0)	Mucosal healing (FC <250µg/g)	IFX	ELISA (

For peer review only

Reference	Cut-off in µg/ml	Performance measures				AUC (95% CI)	Clinical marker	Drug	Assay
		Sens	Spec	PPV	NPV				
	optimisation)								
Singh 2014[75]	4<br>7	0.53<br>0.33	0.75<br>1.00	0.76<br>1.00	0.52<br>0.50	0.64 (0.51-0.75)<br>0.67 (0.58-0.75)	Week 14 Infliximab levels as predictor of week 54 clinical remission according to CDAI	IFX	ELISA
Baert 2014[27]	2 (after re-exposure to Infliximab)	NR	NR	NR	NR	0.76 (0.62-0.90)	Long term response (clinical assessment [HBI] and CRP levels[<3mg/l])	IFX	HMSA
Levesque 2014[76]	3	NR	NR	NR	NR	NR	Disease activity at week 8 (≥70 point increase in CDAI and CRP >5µg/l)	IFX	HMSA
Vande Casteele 2013[40]	13 (TL week 6)	0.72	0.81	NR	NR	0.87 (SE 0.06)	anti-drug antibody formation	IFX	HMSA
Feagan 2012[77] Abstract	3	NR	NR	NR	NR	0.74	Disease activity	IFX	HPLC based fluid phase assay
Goldberg 2014[69] Abstract	3	0.83	0.63	NR	NR	0.8	Disease activity (physicians global assessment and CRP levels)	ADA	ELISA
Karmiris 2009[78]	0.33	0.95	NR	0.81	NR	NR	Sustained clinical benefit (patient reporting lasting control of disease with possible dose escalation)	ADA	ELISA
Ward 2013[79] Abstract	4.9	0.83	0.65	NR	NR	0.75	Remission	ADA	LISA
Yarur 2013[80] Abstract	5	NR	NR	NR	NR	0.71	Elevation of CRP	ADA	HMSA
Mazor 2013[81] Abstract	5	NR	NR	NR	NR	0.77 (0.67-0.86)	Clinical response and normal CRP	ADA	NR

For peer review only

Supplement 3 Summary of quality assessment results using the QUADAS-2 tool with index questions adapted to the review for studies comparing performance of different tests

Tabular presentation of QUADAS-2 results

Study	RISK OF BIAS				APPLICABILITY CONCERNS		
	PATIENT SELECTION	INDEX TEST	REFERENCE STANDARD	FLOW AND TIMING	PATIENT SELECTION	INDEX TEST	REFERENCE STANDARD
Ainsworth 2008[17]	☹	☺	☹	☺	☺	☺	☹
Baert 2014[27]	☺	☺	☹	☺	☺	☺	☹
Ben-Horin 2011[28]	☹	☺	☹	☺	☺	☹	☹
Ben-Horin 2012[29]	☹	☺	☹	☺	☺	☺	☹
Bortlik 2013[19]	☹	☺	☹	☺	☺	☺	☹
Candon 2005[31]	☹	☺	☺	☺	☺	☺	☺
Chiu 2013[41]	☹	☹	☺	☺	☺	☹	☺
Cornillie 2014 #410}	☹	☹	☺	☹	☺	☹	☺
Farrell 2003[33]	☺	☺	☹	☺	☺	☺	☹
Frederiksen 2014[42]	☹	☺	☹	☺	?	☺	☹
Hanauer 2004[34]	☹	☹	☺	☹	☺	☹	☺
Hibi 2014[20]	☹	☺	☺	☺	☺	☺	☺
Imaeda 2012[21]	☹	☺	☺	☺	☺	☺	☺
Imaeda 2014[43]	☹	☺	☺	☺	☺	☺	☺
Kopylov 2012[22]	☹	☺	☹	☺	☺	☺	☹
Maser 2006[23]	☺	☺	☺	☺	☺	☺	☺
Mazor 2014 [44]	☺	☺	☺	☺	☺	☺	☺
Pariente 2012[38]	☹	☺	?	☺	☹	☺	?
Roblin 2014[45]	☹	☺	☺	☺	☺	☺	☺
Steenholdt 2011[24]	☹	☺	☹	☺	☺	☺	☹
Steenholdt 2013[39]	☹	☺	☹	☹	☺	☺	☹
Steenholdt 2014[25]	☺	☺	☺	☺	☺	☺	☺
Van Casteele 2013[40]	☹	☺	☺	☺	☹	☺	☺
West 2008[46]	☹	☺	☹	☺	☺	☺	☹

☺Low Risk ☹High Risk ? Unclear Risk

Graphical summary presentation of QUADAS-2 quality assessment results

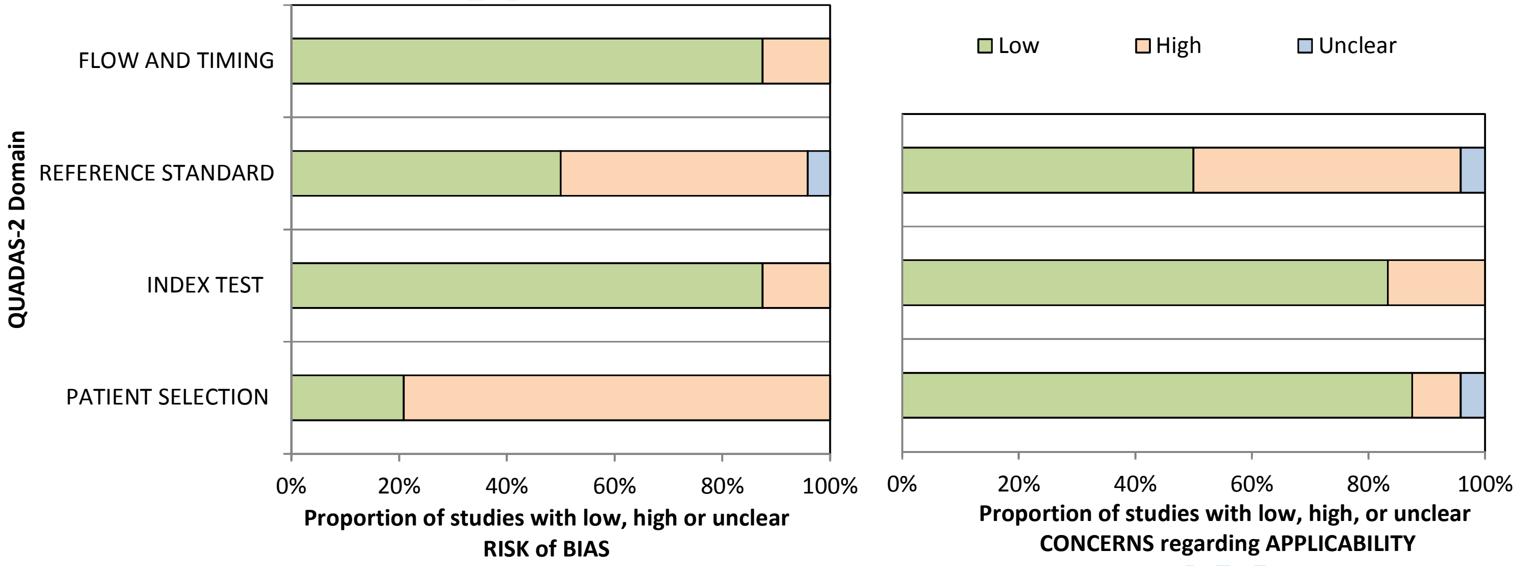

For peer review only - http://bmjopen.bmj.com/site/about/guidelines.xhtml

Supplement 4 Results of hierarchical meta-analysis of included studies

Table S4 Test accuracy statistics from hierarchical meta-analyses

Trough Infliximab level as predictor of loss or absence of response				
Studies included	parameter	Point estimate	95% LCI	95% UCI
all 11 studies	Sens	0.657232	0.546288	0.753299
all 11 studies	Spec	0.80625	0.744166	0.85618
all 11 studies	DOR	7.978975	4.119972	15.45254
all 11 studies	LR+	3.392169	2.35152	4.893351
all 11 studies	LR-	0.425139	0.305104	0.592398
all 11 studies	1/LR-	2.352175	1.688056	3.277573
responder populations only	Sens	0.681452	0.592117	0.759178
responder populations only	Spec	0.790873	0.723301	0.845468
responder populations only	DOR	8.090128	4.353039	15.03551
responder populations only	LR+	3.258549	2.287802	4.641198
responder populations only	LR-	0.402781	0.298559	0.543385
responder populations only	1/LR-	2.482739	1.840315	3.349423
ELISA studies only	Sens	0.652104	0.564027	0.730877
ELISA studies only	Spec	0.789041	0.691592	0.861849
ELISA studies only	DOR	7.010794	3.450232	14.24578
ELISA studies only	LR+	3.091133	1.959085	4.877331
ELISA studies only	LR-	0.440911	0.329778	0.589495
ELISA studies only	1/LR-	2.268033	1.696367	3.032348
**Trough level of antibodies to Infliximab as predictor of loss or absence of response**				
Studies included	parameter	Point estimate	95% LCI	95% UCI
all 20 studies	Sens	0.559745	0.444812	0.668611
all 20 studies	Spec	0.792243	0.688105	0.868267
all 20 studies	DOR	4.848283	2.519589	9.329239
all 20 studies	LR+	2.694226	1.72293	4.213088
all 20 studies	LR-	0.555707	0.426575	0.72393
all 20 studies	1/LR-	1.799509	1.38135	2.344251
all studies minus outliers	Sens	0.597	0.477	0.707
all studies minus outliers	Spec	0.807	0.742	0.859
all studies minus outliers	DOR	6.183	3.805	10.050
all studies minus outliers	LR+	3.088	2.311	4.127
all studies minus outliers	LR-	0.500	0.381	0.655

For peer review only

all studies minus outliers	1/LR-	2.002	1.528	2.623
responder populations only	Sens	0.570	0.445	0.687
responder populations only	Spec	0.849	0.787	0.896
responder populations only	DOR	7.460	4.544	12.250
responder populations only	LR+	3.778	2.722	5.244
responder populations only	LR-	0.506	0.388	0.660
responder populations only	1/LR-	1.974	1.514	2.574
ELISA studies only	Sens	0.482	0.355	0.611
ELISA studies only	Spec	0.880	0.841	0.911
ELISA studies only	DOR	6.830	3.872	12.050
ELISA studies only	LR+	4.022	2.805	5.768
ELISA studies only	LR-	0.589	0.459	0.755
ELISA studies only	1/LR-	1.698	1.324	2.178
**Trough Adalimumab level as predictor of loss or absence of response**				
	Parameter	Point estimate	95% LCI	95% UCI
All 5 studies	Sens	0.543476	0.246586	0.812386
All 5 studies	Spec	0.640241	0.325873	0.86758
All 5 studies	DOR	2.118592	0.172646	25.99789
All 5 studies	LR+	1.510665	0.38102	5.989464
All 5 studies	LR-	0.713051	0.229687	2.213631
All 5 studies	1/LR-	1.402424	0.451747	4.353753
All studies minus Chiu	Parameter	Point estimate	95% LCI	95% UCI
All studies minus Chiu	Sens	0.684	0.591	0.764
All studies minus Chiu	Spec	0.786	0.643	0.883
All studies minus Chiu	DOR	7.971	3.646	17.428
All studies minus Chiu	LR+	3.201	1.822	5.623
All studies minus Chiu	LR-	0.402	0.297	0.542
All studies minus Chiu	1/LR-	2.490	1.844	3.363
**Trough level of antibodies to Adalimumab as predictor of loss or absence of response**				
	Parameter	Point estimate	95% LCI	95% UCI
All 6 studies	Sens	0.471206	0.2903357	0.66
All 6 studies	Spec	0.915467	0.7939073	0.968
All 6 studies	DOR	9.65022	4.387759	21.22
All 6 studies	LR+	5.574189	2.646268	11.74
All 6 studies	LR-	0.577623	0.4208713	0.793

For peer review only

All 6 studies	1/LR-	1.731233	1.261422	2.376
	Parameter	Point estimate	95% LCI	95% UCI
All studies minus Mazor	Sens	0.542264	0.3611645	0.713
All studies minus Mazor	Spec	0.884874	0.7444581	0.953
All studies minus Mazor	DOR	9.105532	3.764526	22.02
All studies minus Mazor	LR+	4.710191	2.221639	9.986
All studies minus Mazor	LR-	0.517289	0.361111	0.741
All studies minus Mazor	1/LR-	1.933156	1.349505	2.769
Sens = sensitivity; Spec = specificity; DOR = diagnostic odds ratio; LR+ = positive likelihood ratio; LR- = negative likelihood ratio; 1/LR- = inverse of negative likelihood ratio.				

For peer review only

Supplement 4 Figure S1. Hierarchical meta-analysis of studies of trough levels of antibodies to Adalimumab (upper row) and of Adalimumab (lower row) for predicting loss of response or failure to regain response

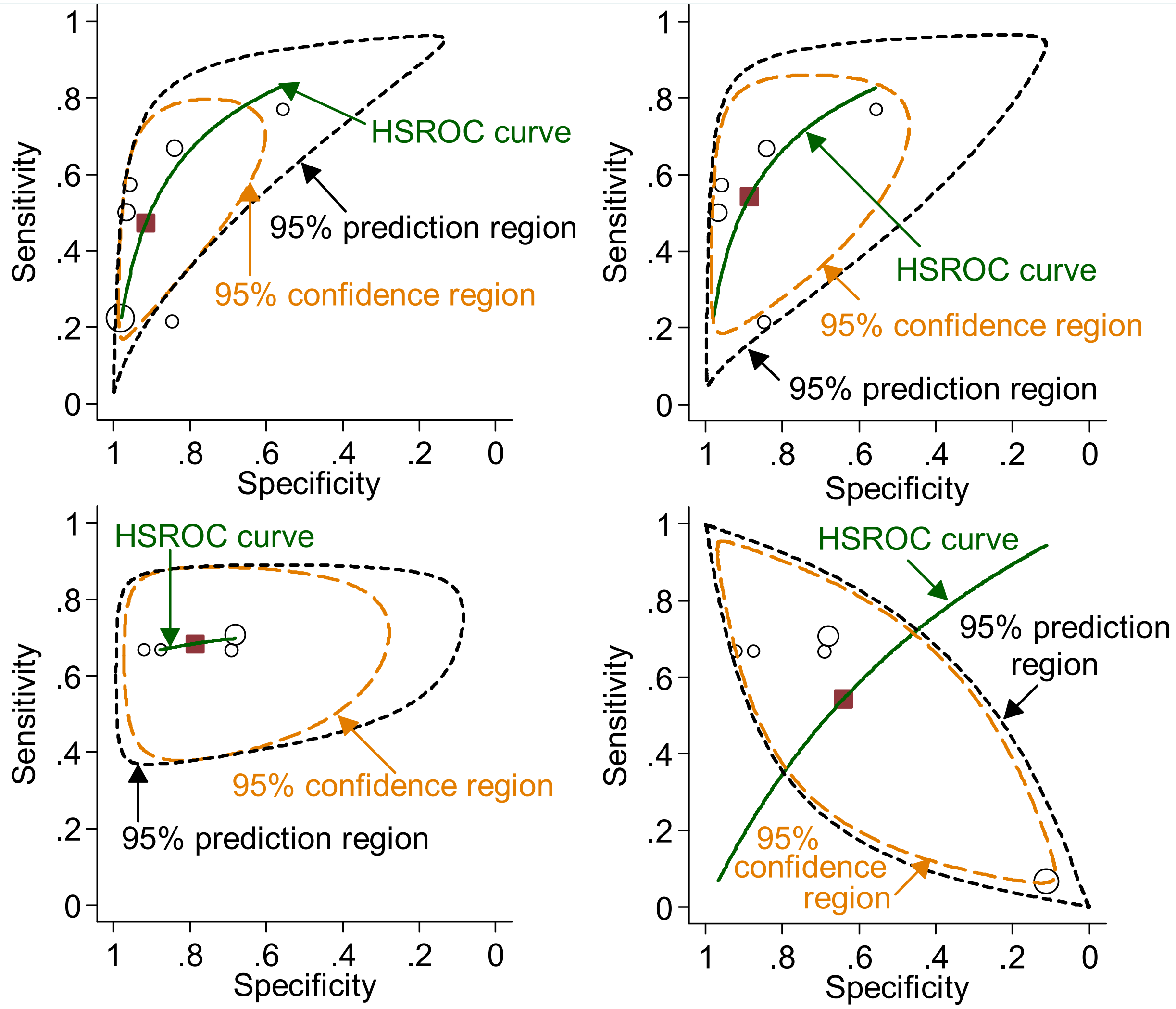

Top Upper left = all anti-Adalimumab antibody studies; upper right = anti-Adalimumab antibody studies but omitting the study of Mazor; lower left Adalimumab studies but omitting the study of patients with secondary loss of response (Chui); lower right = all Adalimumab studies. The square symbol represents the summary point estimate on the HSROC curve.

Supplement 5 Funnel plots and tests for publication bias

In the meta-analysis of tests for trough Infliximab levels using funnel plots and Harbord's and Peter's tests for small study bias in diagnostic odds ratios[82 83] we found no evidence of small study bias in diagnostic odds ratios: Harbord test p = 0.312, Peters test p = 0.576. The corresponding values for tests of antibodies against Infliximab were p = 0.734 and p = 0.780.

Antibodies to Infliximab

1] Funnel plot

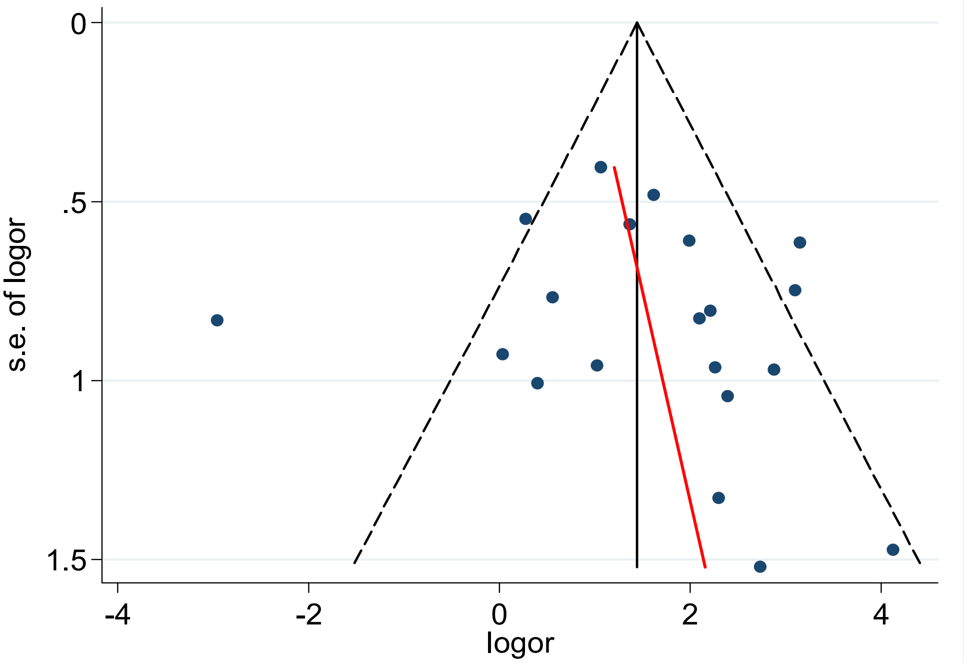

2] Egger's test for small-study effects:

Number of studies = 20

Eggers test

slope | .8614847 .8816692 0.98 0.341 -.9908337 2.713803

bias | .8517858 1.21317 0.70 0.492 -1.69699 3.400561

Test of H0: no small-study effects P = 0.492

Does not support publication bias.

For peer review only - http://bmjopen.bmj.com/site/about/guidelines.xhtml

3] Harbord plot

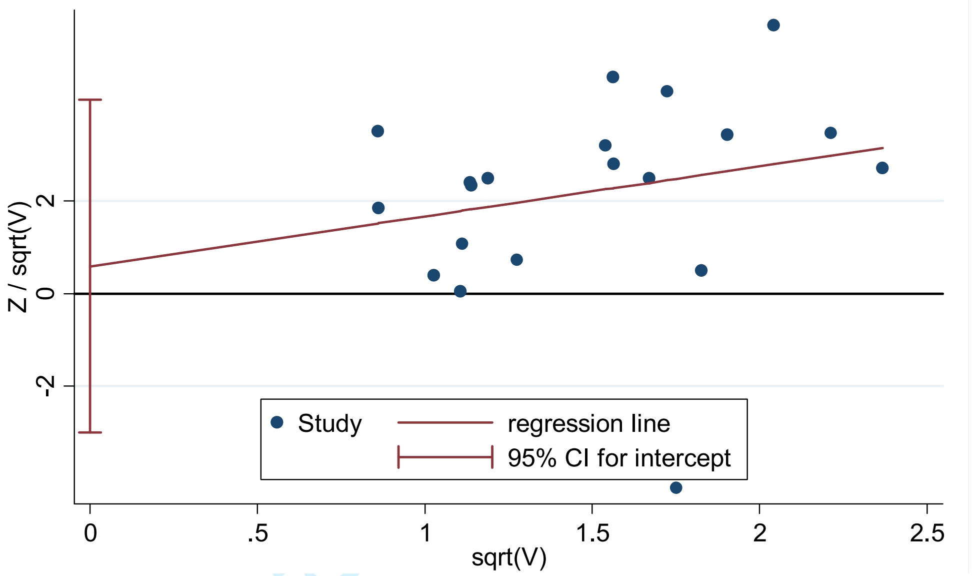

4] Harbord's modified test for small-study effects:
Number of studies = 20 Root MSE = 2.125

Z/sqrt(V)	Coef.	Std. Err.	t	P>|t|	[95% Conf. Interval]	
sqrt(V)	1.079732	1.099815	0.98	0.339	-1.230893	3.390356
bias	.5901862	1.710314	0.35	0.734	-3.003051	4.183424

Test of H0: no small-study effects P = 0.734

5] Peter's test for small-study effects:
Number of studies = 18 Root MSE = 1.459

Std_Eff	Coef.	Std. Err.	t	P>|t|	[95% Conf. Interval]	
bias	-8.626685	30.41227	-0.28	0.780	-73.09781	55.84444
constant	1.674552	.6008762	2.79	0.013	.400751	2.948352

Test of H0: no small-study effects P = 0.780

For peer review only

Trough Infliximab tests

1] Funnel plot

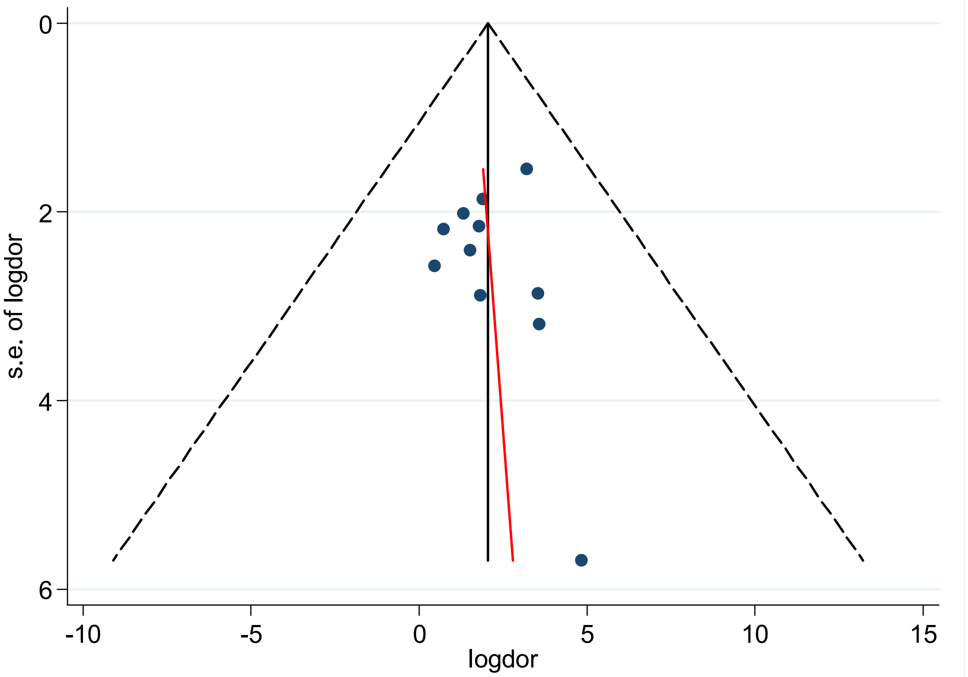

2] Egger's test for small-study effects:
Regress standard normal deviate of intervention
effect estimate against its standard error

Number of studies = 11 Root MSE = 1.907

Std_Eff	Coef.	Std. Err.	t	P>|t|	[95% Conf. Interval]	
slope	1.580826	1.251978	1.26	0.238	-1.251345	4.412998
bias	.8249369	2.088696	0.39	0.702	-3.900021	5.549894

Test of H0: no small-study effects P = 0.702

For peer review only - http://bmjopen.bmj.com/site/about/guidelines.xhtml

3] Harbord plot

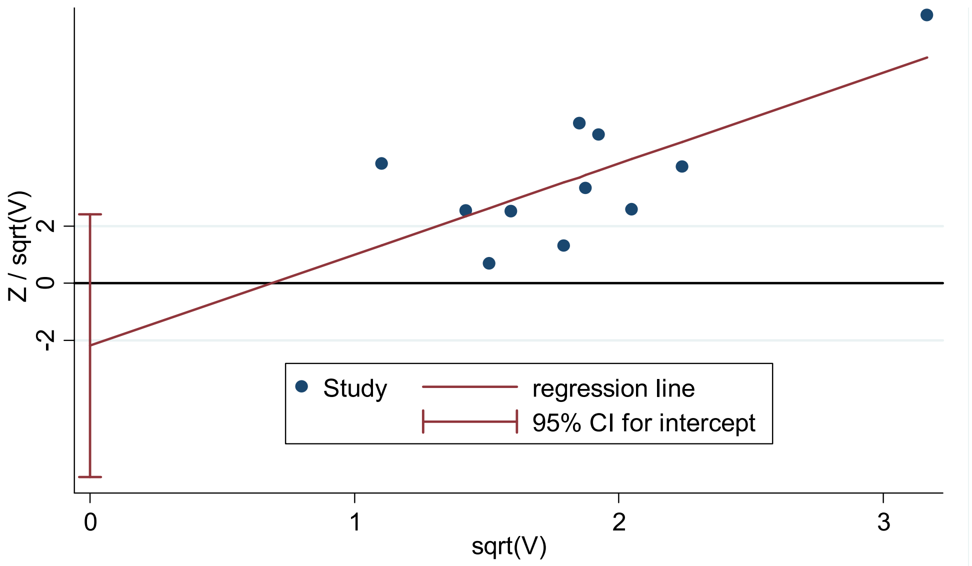

4] Harbord's modified test for small-study effects:
Regress Z/sqrt(V) on sqrt(V) where Z is efficient score and V is score variance
Number of studies = 11 Root MSE = 1.779
Test of H0: no small-study effects P = 0.312

5] Peter's test for small-study effects:
Regress intervention effect estimate on 1/Ntot, with weights S×F/Ntot
Number of studies = 11 Root MSE = 1.191

Std_Eff	Coef.	Std. Err.	t	P>|t|	[95% Conf. Interval]	
bias	-28.29877	48.81199	-0.58	0.576	-138.7192	82.12163
constant	2.738445	.725501	3.77	0.004	1.097248	4.379642

Test of H0: no small-study effects P = 0.576

For peer review only

Diagnostic Assessment Report commissioned by the NIHR HTA Programme on behalf of the National Institute for Health and Clinical Excellence – Final Protocol

Title of project

Crohn's disease: Tests for therapeutic monitoring of TNF inhibitors (LISA-TRACKER ELISA kits, TNFα-Blocker ELISA kits, and Promonitor ELISA kits)

Name of External Assessment Group (EAG) and project lead

Produced by: Warwick Evidence

Lead author: Karoline Freeman

Co-authors: Martin Connock
Hema Mistry
Sian Taylor-Phillips
Rachel Court
Alexander Tsertsvadze
Jason Madan
Ngianga-Bakwin Kandala
Ramesh Arasaradnam
Aileen Clarke
Paul Sutcliffe

Correspondence to: Dr Paul Sutcliffe
Associate Professor
Deputy Director for Warwick Evidence
Populations, Evidence and Technologies
Division of Health Sciences
Warwick Medical School
University of Warwick
Coventry CV4 7AL

Tel: 02476 150189

Fax: 02476 528375

Email: p.a.sutcliffe@warwick.ac.uk

Date completed: 29 October 2014

The views expressed in this protocol are those of the authors and not necessarily those of the NIHR HTA Programme. Any errors are the responsibility of the authors. The authors have no conflicts of interest.

For peer review only - http://bmjopen.bmj.com/site/about/guidelines.xhtml

Glossary of terms

Induction therapy	Treatment to induce remission
Maintenance therapy	Treatment to remain in remission
Remission	Period without or only mild symptoms
Biologics or biological therapy	A protein-based drug derived from living cells cultured in a laboratory
Immunosuppressant	A class of drugs that suppress or reduce the strength of the body's immune system
Resection	The removal by surgery of all or part of an organ such as the bowel
Ileostomy	Surgical procedure where the small intestine is diverted through an opening in the abdomen
Intestinal stricture	Narrowing of the intestine due to tissue scaring following inflammation
Fistulas	Channels formed from the digestive system to other parts of the digestive system or different organs
Azathioprine	Immunomodulator
Thiopurines	Group of drugs (purine antimetabolites) including azathioprine, 6-mercaptopurine and 6-thioguanine
Seton	A thread, wire, or gauze of cotton or other absorbent material passed below the skin and left with the ends protruding, to promote drainage of fluid
Methotrexate	Disease-modifying, antimetabolite

For peer review only

For peer review only - http://bmjopen.bmj.com/site/about/guidelines.xhtml

1. Plain English Summary

Crohn's disease is an uncommon long term disease involving painful and damaging inflammation of the gut lining. Damage can cause bloody stools, development of very narrow sections along the gut (strictures), and the formation of abnormal channels (fistulas) between different regions of the gut or between gut and body surface or between gut and nearby organs. Particularly distressing fistulas may occur between intestine and vagina in female patients. During a patient's life the severity of Crohn's disease fluctuates between remission (no symptoms) and relapse (active disease) and treatments aim to induce and maintain remission. Tumour necrosis factor (TNF) has been identified as a molecule important in the development of inflammation in Crohn's disease. Medicines called anti-TNF agents have been developed that counteract the action of TNF and have been found to benefit Crohn's disease patients; they are by far the most expensive medicines used for Crohn's disease and, like all Crohn's disease medicines, for some patients they are associated with unwanted side effects. Unfortunately many patients eventually develop resistance to anti-TNF agents and remission fails. One reason for failure is that some patients develop antibodies to anti-TNFs so that the amount of drug in the patient's blood decreases below levels that are effective. Test kits have been developed and marketed that allow estimation of the levels of anti-TNF and of antibodies to anti-TNF in a patient's blood sample. This information can aid clinicians and patients to decide on the best course of future treatment, and may help avoid continued use of expensive but ineffective medicine. The present project aims to examine evidence about the clinical and cost effectiveness of test kits. The current report will allow NICE to make recommendations about how well the kits work and whether the benefits are worth the cost of the tests for use in the NHS in England and Wales. The assessment will consider both potential for improvement in patients' symptoms associated with use of the tests and the cost of the tests.

2. Decision problem

The current report being undertaken for the NICE Diagnostics Assessment Programme examines the clinical and cost effectiveness of ELISA tests (LISA-TRACKER EISA kits, TNFα-Blocker ELISA kits, and Promonitor ELISA kits) for measuring patient blood levels of anti-TNF agents (Infliximab and Adalimumab; also known as TNF inhibitors) and of antibodies to these agents (i.e., anti-drug antibody levels, ADAbs) in people with Crohn's disease whose disease responds to treatment with TNF inhibitor or who experience secondary loss of response during a maintenance course of TNF inhibitor therapy.

2.1 Anti-tumour necrosis factor alpha (anti-TNFα) agents

TNFα is a small cell-signalling protein (cytokine) involved in inflammatory responses primarily by influencing regulation of various effector cells of the immune system. TNFα has been shown to have

For peer review only - http://bmjopen.bmj.com/site/about/guidelines.xhtml

a role in several inflammatory diseases including Crohn's disease, ulcerative colitis, rheumatoid arthritis and ankylosing spondylitis. Therapies have been developed that are directed at blocking the actions of TNFα and thereby reducing inflammation. Such anti-tumour necrosis factor alpha (anti-TNFα) agents bind to cell surface TNFα and free TNFα and block its activity. Blocking of TNFα with anti-TNF drugs has been shown to successfully reduce the inflammation for some patients with inflammatory diseases including Crohn's disease. As these drugs are expensive and can cause potentially serious adverse effects, in England, they are generally used as second or third line treatment in the management of Crohn's disease and are employed when other drugs have not worked or have caused major side effects, and when surgery is not considered the appropriate treatment option. The anti-TNF agents recommended by NICE for the treatment of Crohn's disease are infliximab (Remicade®, Schering-Plough) and adalimumab (Humira®, Abbott Laboratories). These are monoclonal antibodies introduced into the human body to bind and block TNFα. They are classed as monoclonal antibodies because they are derived from genetically engineered immune cells, which are all daughters of a single parent cell, so that in culture they generate and secrete antibodies that are all of identical structure and affinity for TNFα.

2.1.1 Infliximab

Infliximab is a chimeric (mouse-human) monoclonal antibody. It is said to be chimeric because the genetic code determining its amino acid sequences is partly derived from the mouse genome and partly from the human genome. Infliximab belongs to the IgG1 (immunoglobulin gamma type 1) group of antibody molecules (Figure 1). It should be born in mind that IgG1 molecules are globular (not linear as in the diagram) and that they are glycoproteins that have carbohydrate chains attached (not shown in Figure 1). As infliximab is generated from cultured mouse cells, the carbohydrate part of the molecules corresponds to that of mouse rather than human glycoproteins.

For peer review only

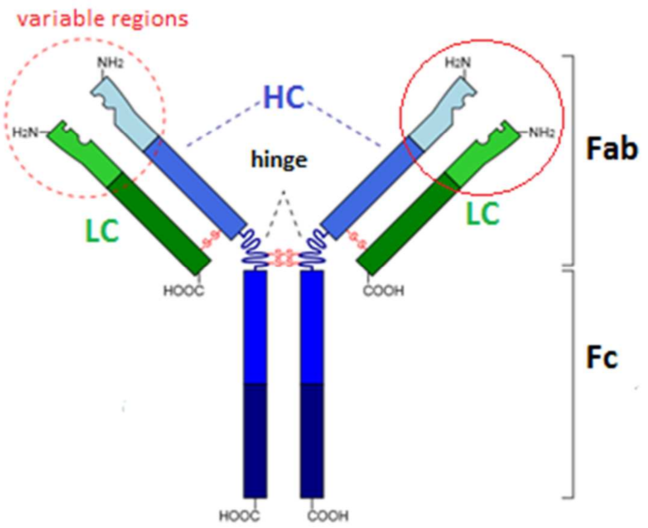

Figure 1. Diagrammatic representation of the structure of an IgG1 antibody molecule. *The molecule comprises two heavy chains (HC) and two light chains (LC); the HCs are joined together across disulphide bonds (S-S) and each LC is joined to a HC by S-S bonding. The LC and HC have a variable region (different from all other antibodies) at the amino (NH_2) end of the chain; these variable regions are responsible for binding antigen. The rest of the HC and LC are identical to other IgG1 antibodies and are called constant regions. Proteolytic enzymes papain and pepsin cut the molecule just above or below the S-S bonds holding the HC together. When below the HC S-S bond this generates an Fc (Fragment crystallising) and an Fab (Fragment antigen binding) product. When the split is above the HC S-S bond two antigen binding fragments are formed ($F(ab)_2$).*

Infliximab is composed of human IgG1 heavy chain constant regions and human Kappa light chain constant regions (together representing 70% of the genetic makeup of the molecule), plus mouse-derived heavy chain and light chain variable regions (30% of the genetic makeup, 4 out of 12 domains) which carry the binding sites with high affinity and specificity to TNFα (Figure 1). Infliximab was the first anti-TNF agent that was approved and licenced for treating severe active Crohn's disease and active fistulising Crohn's disease in adults and children over the age of six. It is administered intravenously over 1–2 hours. Details of the licenced indication are given in Appendix 1.

Side effects of infliximab include:

- Allergic reaction to the infusion (or infliximab) apparent by:
    - hives (red, raised, itchy patches of skin) or other skin rashes
    - difficulty swallowing or breathing
    - pains in the chest or muscle or joint pain fever or chills
    - swelling of the face or hands
    - headaches or a sore throat
- Serious viral or bacterial infections including tuberculosis, especially in people over 65
- Skin reactions including psoriasis (red scaly patches), rashes, skin lesions, ulcers and hives, and swollen face and lips
- Worsening of heart problems
- Increased risk of cancer or lymphoma
- Liver inflammation

Many of the side effects are reversible if the drug is stopped.

For peer review only - http://bmjopen.bmj.com/site/about/guidelines.xhtml

2.1.2 Adalimumab

Adalimumab is a human IgG1 monoclonal antibody with Kappa light chains. It consists of purely human antibody polypeptide domains (Figure 1). However, as adalimumab is generated from cultured Chinese hamster ovary cells, the carbohydrate part of the molecules corresponds to that of hamster rather than human glycoproteins. Adalimumab is a more recent anti-TNFα therapy that was approved for treating Crohn's disease in adults only. It is administered as a subcutaneous injection by a doctor or nurse or can be self-injected by the patient or a family member. Details of the licenced indication are given in Appendix 1.

Side effects of adalimumab include:

- Reactions to the injection including pain, swelling, redness, bruising and itching
- Allergic reaction to adalimumab including:
    - rashes or hives
    - swollen face, hands and feet
    - trouble breathing
- Greater susceptibility to infections such as colds, flu, pneumonia, sepsis and tuberculosis
- Skin reactions including psoriasis (scaly patches), eczema, other skin rashes and ulcers
- Skin cancer, lymphoma or leukaemia
- Damage to nerves (demyelination)
- Lupus

Many of the side effects are reversible if the drug is stopped.

2.2 Intervention technologies

The intervention technologies are the LISA-TRACKER ELISA kits (Theradiag / Alpha Laboratories), the TNFα-Blocker ELISA kits (Immundiagnostik AG), and the Promonitor ELISA kits (Proteomika). They estimate the following molecules in patient blood sera:

- Infliximab
- Adalimumab
- Anti-infliximab antibodies
- Anti-adalimumab antibodies

For peer review only - http://bmjopen.bmj.com/site/about/guidelines.xhtml

For peer review only

2.2.1 Anti-TNF monitoring using assays to measure the levels of anti-tumour necrosis factor-alpha agents (anti-TNFα drugs) and the anti-drug antibodies (ADAb) in the blood plasma or serum

Rationale

In some patients an initial or maintained response to anti-TNF therapy may disappear. This has been observed for all conditions in which these therapies have been used. The reasons for response failure may be various and are not fully understood, however loss of response has often been found to be associated with the generation of immune responses to the anti-TNF agent itself. In particular the patient may generate antibodies directed against the anti-TNF agent, these will bind to the administered anti-TNF agent, nullify its effectiveness and hasten its clearance from the circulation. These effects may explain or partially explain the phenomena of loss of response experienced by some patients. The generation of antibodies against infliximab may not be surprising since about 30% of the molecule has mouse identity. Adalimumab, although termed a fully humanised antibody, has potential to be antigenic since its carbohydrate moieties are mouse derived and because its binding site for anti-TNF is unique and could, according to the network hypothesis of Jerne,[1] lead to generation of antibodies directed against this "idiotypic" region of the drug.

Other patients may respond well to an induction phase of treatment with a TNF inhibitor. However, these patients may lose response in the future, may benefit from optimising dosing or may require review after 12 months of treatment with a TNF inhibitor. Management of responders could benefit from knowing levels of anti-TNF drug and anti-drug antibodies in the patients' blood.

Manufacturers and others have developed various assay procedures for anti-TNF agents and for anti-drug antibodies (ADAbs) in the belief that the levels of circulating anti-TNF and of ADAbs can provide information useful to clinicians in indicating potential reasons for treatment failure, and for dosage or treatment adjustment. The LISA-TRACKER, TNFα-Blocker, and Promonitor are particular examples of these assays and are classified as solid phase Enzyme Linked Immunosorbent Assays (ELISA assays). Other methodologies based on alternative principles of detection and measurement include: [a] radioimmunoassays; liquid phase assays [b] cell reporter assays based on genetically engineered cells incubated in culture medium; [c] mobility shift assays; liquid phase assays using size-exclusion HPLC and fluorescent dye detection. Brief descriptions of the assay methods follow.

ELISAs for infliximab and adalimumab

All three ELISA methods employ similar principles in which, typically, micro-titre plates with 96 wells coated with reagent receive the patient serum samples or various standards and calibrators. Reagents are added with wash steps between additions. The final step involves quantifying the

For peer review only - http://bmjopen.bmj.com/site/about/guidelines.xhtml

amount of a peroxidase label in the titre well, this amount being proportional to the amount of anti-TNF or ADAb in the patient's sample or in the calibrator standard.

The amount of peroxidase present in the well is quantified using a timed incubation with excess substrates (hydrogen peroxide + 3,3',5,5'-tetramethylbenzidine). Peroxidase catalyses the following reaction: Tetramethylbenzidine + hydrogen peroxide → chromogen + water
The incubation is stopped after an appropriate time by the addition of acid and the accumulated chromogen quantified by measuring optical density with a spectrophotometer.

The reagents used for coating the microtitre plate wells and the reagents used in subsequent steps of the assay procedure differ from each other according to manufacturer. The LISA-TRACKER assays for Infliximab and for Adalimumab are illustrated in Figure 2.

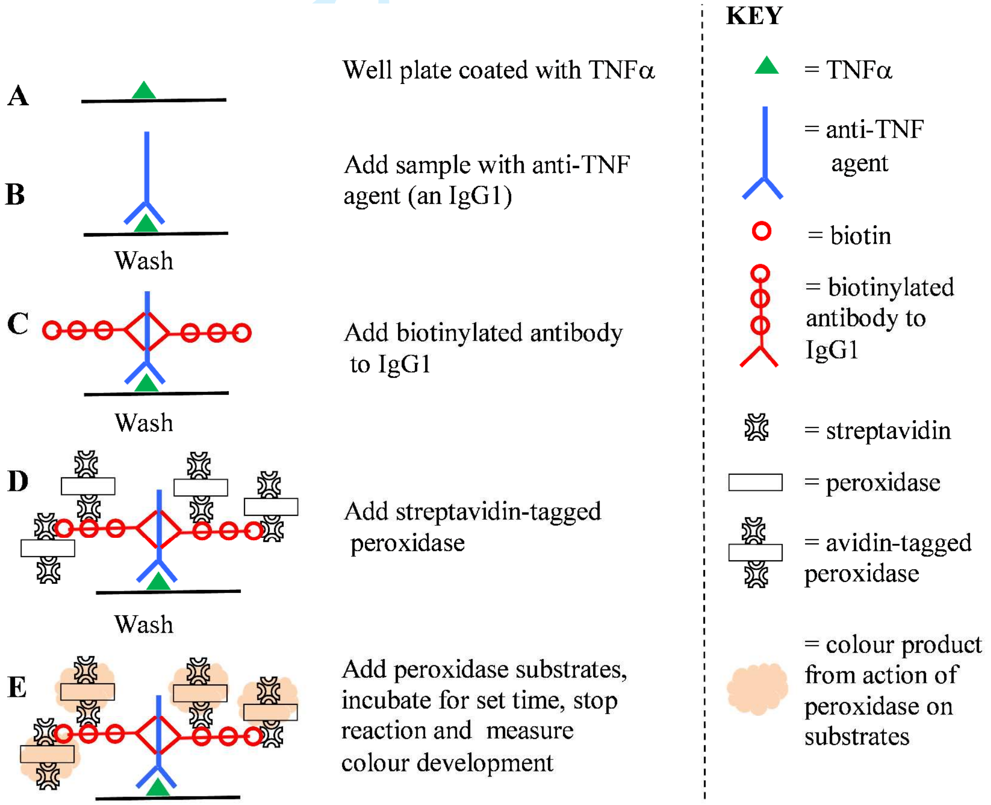

Figure 2. Diagrammatic representation of the LISA-TRACKER assay for infliximab and Adalimumab

Procedural steps C and D are detection steps that function to detect the anti-TNF that is bound to the well surface via TNFα, ensuring a quantitative relationship between anti-TNF and peroxidase. Step E quantifies the amount of peroxidase (and therefore anti-TNF) in the titre well (note: Streptavidin has four very high affinity binding sites for biotin).

For peer review only - http://bmjopen.bmj.com/site/about/guidelines.xhtml

Serum samples from patients may contain soluble TNFα receptors; these could compete with anti-TNF for the immobilised TNFα on the well plate and may potentially interfere with the assay. The assay quantifies free anti-TNF. Samples may contain anti-TNF bound to antibodies to anti-TNF, especially in patients who have lost a response to treatment. These anti-TNF-antibody complexes will be washed away at the first wash step leaving only free anti-TNF bound to immobilised TNFα. The amount of anti-TNF lost at the wash step is likely to vary between patients and is unknown; the practical implications of this are uncertain.

TNFα-Blocker and Promonitor differ from LISA-TRACKER in employing a single step and one reagent for detecting well-bound anti-TNF, rather than two steps (C and D in Figure 2) and two reagents. Table 1 summarises the information currently available describing the principle of these assays.

Table 1. Summary of ELISAs to be considered in this review for detection of infliximab and adalimumab

Manufacturer (Kit)	Microplate pre-coat	Detection reagent(s)	
LISA-TRACKER	TNFα	Biotinylated IgG1 antibody	Avidin-tagged peroxidase
TNFα-Blocker ELISA	Monoclonal anti-TNF antibody	Peroxidase labelled antibody	
Proteomika ELISA	Monoclonal anti-TNF antibody	Peroxidase labelled monoclonal anti-TNF antibody	

<u>ELISAs for anti-drug antibodies (ADAbs)</u>

These are available as commercial kits and several "in house" methods are mentioned in the literature. The majority of ELISAs only quantitatively measure "free" anti-TNF and "free" ADAbs and it is acknowledged that the level of the unmeasured "bound" anti-TNF and of "bound" ADAb may vary considerably between patients. The Immundiagnostik assays give semi-quantitative measurement of 'total' ADAbs. Thus for some patient samples there is an unknown and unmeasured amount of anti-TNF and of ADAb present, in addition to the measured "free" levels.

Below the LISA-TRACKER methods are reported and differences to TNFα-Blocker and Promonitor are described. The LISA-TRACKER assays for antibodies to infliximab and to adalimumab are illustrated in Figure 3.

For peer review only - http://bmjopen.bmj.com/site/about/guidelines.xhtml

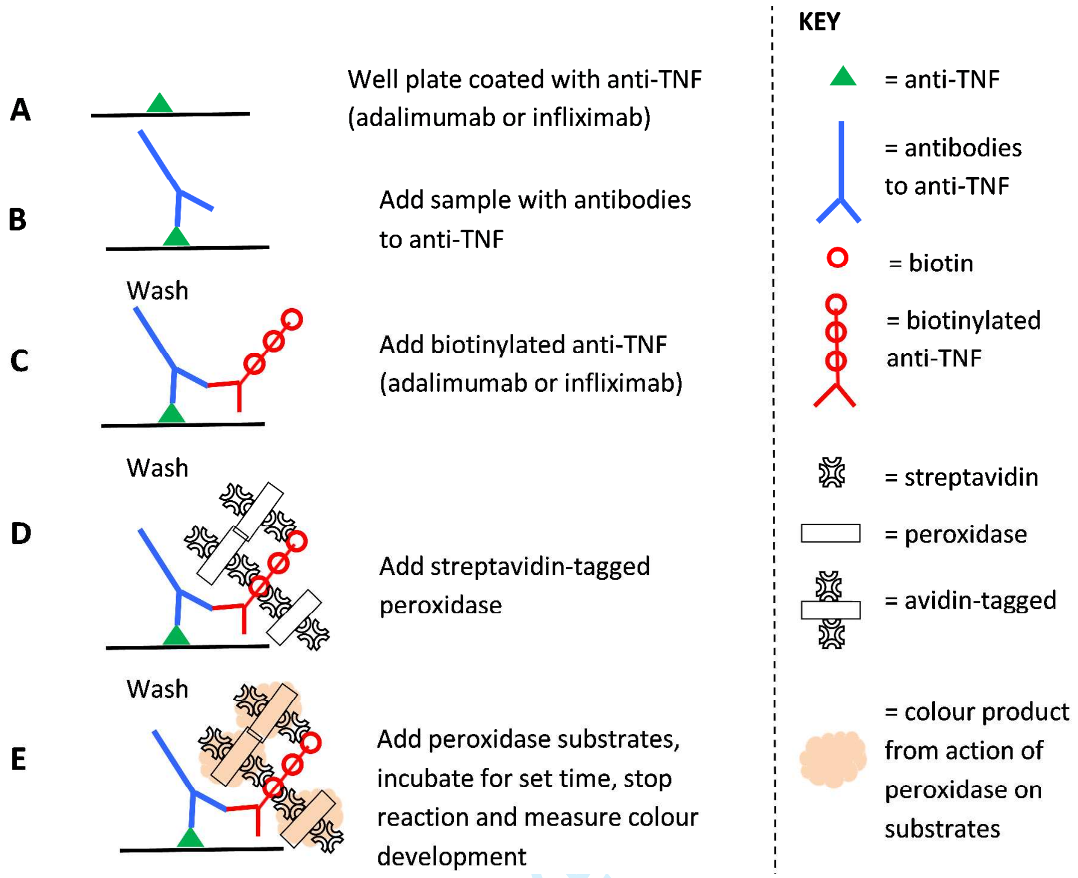

Figure 3. Diagrammatic representation of the LISA-TRACKER assay for antibodies to infliximab or to adalimumab.

Procedural steps C and D are detection steps that function to detect the sample antibodies, ensuring a quantitative relationship between anti-TNF antibodies and peroxidase. Step E quantifies the amount of peroxidase (and therefore anti-TNF antibodies) (note: Streptavidin has four very high affinity binding sites for biotin).

This assay only quantitatively estimates free antibodies to anti-TNF. Thus ADAbs bound to the drug are lost at the first wash. The amount of bound ADAb is likely to vary between patients and is unknown. Whether ADAbs directed at non-idiotypic regions of the drugs (e.g., glycoprotein moieties, variable non-idiotypic mouse regions of infliximab etc.) are detectable or present in samples appears to be uncertain.

TNFα-Blocker and Promonitor differ from LISA-TRACKER in employing a single step and reagent for detecting well-bound anti-TNF rather than two steps (C and D in Figure 2) and two reagents. Table 2 summarises the information currently available describing the principle of these assays.

For peer review only - http://bmjopen.bmj.com/site/about/guidelines.xhtml

Table 2. Summary of ELISAs to be considered in this review for detection of antibodies to infliximab and adalimumab

Manufacturer (Kit)	Microplate pre-coat	Detection reagent(s)	
LISA-TRACKER	Anti-TNF	Biotinylated anti-TNF	Avidin-tagged peroxidase
TNFα-Blocker ELISA infliximab	Infliximab F(ab)2	Peroxidase labelled infliximab	
TNFα-Blocker ELISA adalimumab	Adalimumab F(ab)2	Peroxidase labelled adalimumab	
Proteomika ELISA	Anti-TNF	Peroxidase labelled anti-TNF	

<u>Brief overview of identified non-ELISA assay methods</u>

There are no "gold standard" assays for measuring anti-TNF agents or for antibodies to anti-TNF agents which might provide a robust basis for comparisons between the performance of different assays. According to the US Medical Insurance assessments "candidate" gold standards have been insufficiently investigated to establish any as a gold standard, and according to Steenholdt et al. (2013)[2] it is unknown if and how these different assays compare.[3-7]

There appear to be four types of assay for measuring the levels of anti-TNF drugs and the levels of antibodies against TNF inhibitors in patient blood sera. which differ fundamentally from each other. In addition to ELISAs (solid phase assays) these are:

(a) Radioimmunoassays (RIA) – liquid phase. They appear to measure total anti-TNF and total ADAb (probably as long as the ADAb light chain is lambda class). These RIAs use 125 iodine-labelled human TNFα and 125 iodine-labelled anti-TNFs. In these assays the patient's sample is mixed with a solution containing a fixed amount of 125 iodine-labelled TNFα or 125 iodine-labelled anti-TNF further antibody (e.g., rabbit anti-human immunoglobulin λ-chain) which promotes the formation of immune complexes which are pelleted by centrifugation. Radio-iodine in the pellet is quantified in a gamma-counter. Characteristics of these assays include: i) radio-labelled reagents do not store indefinitely (125 iodine decays with a half-life of 59 days), ii) the laboratory needs to be equipped for handling hazardous (radioactive) material, iii) some staff training may be necessary, and iv) the laboratory requires a gamma counter (preferably automated for high throughput).

(b) Cell Reporter Assays. The reporter cells are genetically engineered to contain genes for two light producing enzymes "*luciferases*" (one from the firefly which can generate red light, and one from the sea pansy which can generate blue light). The firefly gene is under the control of a TNFα signalling

For peer review only - http://bmjopen.bmj.com/site/about/guidelines.xhtml

pathway so that when the cells are incubated in the presence of TNFα they synthesise the enzyme, after a standard incubation time appropriate substrates for the enzyme are added and the emitted red light measured with a luminometer. If anti-TNF is present the TNFα response is partially quenched and the quenching estimated. If ADAb is present, quenching by anti-TNF is reduced and this can be measured. The sea pansy gene is expressed during incubation after which appropriate substrates are added and the blue light emitted measured in the luminometer. The usefulness of the blue light measure is that it allows "normalisation" of the red light emission as interfering agents in patient blood samples equally affect both firefly and sea pansy systems. Requirements in addition to appropriate cell reporter cultures and reagents include requirement for a luminometer (although these are not necessarily routinely available) and equipment for culture of growth arrested genetically engineered cells under controlled conditions (oxygen, CO_2, humidity).

(c) The Mobility Shift Assay is a liquid phase assay based on size exclusion HPLC (SE-HPLC) which separates free probe (small size) from probe in an immune-complex (large size). The ADAb assays use fluorescent-dye-labelled anti-TNF (D*) as the probe. In the presence of antibodies to anti-TNF some D* form immune complexes with these (D*-ADAb complexes) and will exhibit a mobility shift on the SE-HPLC column relative to the D* which remains free. The amount of D* shifted to greater mobility is proportional to the amount of ADAb present. The amount of dye (*) present in the eluent stream coming from the HPLC column at different mobilities is measured with a fluorimeter.

The anti-TNF assay uses fluorescent-dye-labelled TNFα (TNF*) as the probe; in the presence of anti-TNF some TNF* forms immune-complexes with the anti-TNF and these have greater mobility on the SE-HPLC than the free TNF*. The amount of TNF* shifted to greater mobility is proportional to the amount of anti-TNF present. The amount of dye (*) present in the eluent stream coming from the HPLC column at different mobilities is measured with a fluorimeter.

In measuring ADAb the patient sample is subjected to an acid step which "unbinds" bound anti-TNF and ADAb so that all anti-TNF and ADAb are "free"; after neutralisation the sample is incubated with fluorescent-dye-labelled anti-TNF (D*) as described above. Some D* will form immune complexes with the sample ADAbs (D*-ADAb complexes) and these have a different mobility on SE-HPLC than D* thus the mobility of some of the D* is shifted, the proportion of D* shifted is dependent on the level of ADAb in the sample.

2.3 Timing and use of ELISAs

Scoping searches indicate that the anti-TNF and ADAb assays are most frequently administered just before the next administration of the anti-TNF agent. This is said to allow measurement of a "trough" level of anti-TNF and may have been adopted when ELISAs are used so as to minimise effects from

For peer review only - http://bmjopen.bmj.com/site/about/guidelines.xhtml

the presence of anti-TNF-ADAb immune-complexes in samples. For patients whose response to therapy has waned, the results of the tests are frequently dichotomised using a cut off assay result. Thus, on the basis of anti-TNF assays patients are classified as having therapeutic levels of anti-TNF or sub-therapeutic levels, and on the basis of ADAb assay results they are classified as having clinically significant levels of ADAbs or insignificant levels. Such classifications yield four categories of patient for whom different explanations of failed response are possible. Algorithms have been developed prescribing treatment pathways and / or further diagnostic tests (e.g., colonoscopy) based on such classification.

2.4 Target condition / indication

Anti-TNFα is commonly given to people with inflammatory bowel disease (IBD) including Crohn's disease. The general background and treatment pathway for Crohn's disease is summarised below.

2.4.1 Crohn's disease

Crohn's disease is a chronic fluctuating episodic inflammatory condition of the digestive tract; it is uncommon and is currently estimated to affect about 115,000 people in the UK.[8] Together with ulcerative colitis it comprises conditions classed as inflammatory bowel disease (IBD).

Aetiology and pathology

Crohn's disease can affect adults, adolescents or children. Crohn's disease manifests itself mainly during late adolescence or early adulthood. The first onset most commonly occurs between the ages of 16 and 30 with a second peak between the ages of 60 and 80. Women are slightly more frequently affected than men but in children it is seen more often in boys than in girls. The condition has highest prevalence among Jewish people with European descent.

Crohn's disease follows a pattern of acute disease interspersed with periods of remission. Crohn's disease causes inflammation of the lining of the digestive tract which, depending on the individual, occurs at any location from the mouth to the rectum, but most commonly affects the terminal ileum (35%) or the ileocaecal region (40%). Within individuals the disease location is fairly stable.

The main symptoms of Crohn's disease are dependent on disease location and include chronic or nocturnal diarrhoea, abdominal pain, anal lesions, rectal bleeding and weight loss. Clinical signs include pallor, cachexia, abdominal mass or tenderness, or perianal fissures, fistulas or abscesses. Systemic symptoms include malaise, anorexia or fever.[9-11] Extra-intestinal symptoms related to intestinal inflammation include spondyloarthritis (inflammatory rheumatic diseases which cause arthritis, most commonly ankylosing spondylitis), cutaneous manifestations or ocular inflammation.[11] In children, growth failure may be the primary manifestation of Crohn's disease.[12]

For peer review only - http://bmjopen.bmj.com/site/about/guidelines.xhtml

Classification of Crohn’s disease disease states and measurement of disease activity

Several classification systems of Crohn’s disease have been proposed. The Montreal[13] and Vienna[14] systems are summarised in Tables 3 and 4.

Table 3. Montreal classification of Crohn’s disease

Age at diagnosis	Location	Behaviour
A1: <16 years	L1: Ileal	B1: Inflammatory
A2: 17-40 years	L2: Colonic	B2: Stricturing
A3: >40 years	L3: Ileocolonic	B3: Penetrating
	L4: Upper GI disease	P: Perianal disease

Table 4. Vienna classification of Crohn’s disease

Age at diagnosis	Location	Behaviour
A1: <40 years of age	L1: Terminal ileum - limited to terminal ileum, with or without spill-over into the caecum	B1: Non-stricturing, non-penetrating
A2: ≥40 years of age	L2: Colon - any colonic location between the caecum and rectum, with no small bowel or upper GI involvement	B2: Stricturing - constant luminal narrowing demonstrated by radiological, endoscopic, or surgical-pathological methods, with pre-stenotic dilation or obstructive signs/symptoms, without the presence of penetrating disease, at any time in the course of the disease
	L3: Ileocolonic - disease of ileum and any location between the ascending colon and rectum	B3: Penetrating - occurrence of intra-abdominal or perianal fistulae, inflammatory masses, and/or abscesses at any time in the course of the disease. Perianal ulcers are included. Postoperative intra-abdominal complications and skin tags are excluded
	L4: Upper GI - any disease proximal to the terminal ileum (excluding mouth), regardless of additional involvement of the terminal ileum or colon	

For peer review only

“The severity of Crohn’s disease is difficult to assess, and a global measure encompassing clinical, endoscopic, biochemical and pathological features is not available.[15] The most widely used disease activity measures include the Crohn’s Disease Activity Index (CDAI), the Harvey-Bradshaw Index (HBI) or Simple Index (a simplified version of the CDAI), and the Perianal Disease Activity Index

For peer review only - http://bmjopen.bmj.com/site/about/guidelines.xhtml

(PDAI). A commonly used health related quality of life measure is the Inflammatory Bowel Disease questionnaire (IBDQ). Other measures include the Crohn's Disease Endoscopic Index of Severity (CDEIS).

The CDAI was developed in the 1970s when a need for a single index to assess disease severity was recognised. Variables measured include number of liquid stools, abdominal pain, general well-being, extra-intestinal complications, use of anti-diarrhoeal drugs, abdominal mass, haematocrit and body weight; scores range from 0 to approximately 600 (see Appendix 2 for a description of the index and the scoring system used). Values of below 150 are suggestive of quiescent disease (remission) and values above 450 are associated with very severe disease.[16] Some investigators have arbitrarily labelled CDAI scores of 150-219 as mildly active disease and scores of 220 to 450 as moderately active disease.[15]

The CDAI has been criticised for having limitations since it fails to encompass aspects of quality of life such as psychological, social, sexual wellbeing and occupational functioning. A patient with a low CDAI score may still be severely limited by these factors.[17] Substantial variability exists when different observers review the same case histories and calculate the CDAI score, although this can be reduced after discussion and education about the terminology. The calculation is based in part on a daily diary kept by the patient for seven days before the evaluation. In practice some investigators and study coordinators assist the patient to complete the diary retrospectively at the time of an evaluation visit; there is no information on the prevalence of this practice. The CDAI score may be low in patients whose primary symptom is drainage of enterocutaneous fistulas, presumably because the presence of an actively draining fistula contributes only 20 points to the score. The CDAI is therefore not an appropriate instrument for assessing the activity of draining abdominal or perianal enterocutaneous fistulas. The CDAI has been criticised for giving too much weight to 'general well-being' and 'intensity of abdominal pain' because these are relatively subjective items. However these aspects of disease are important to patients.[18] A paediatric CDAI has been developed.[18, 19]

The HBI or Simple Index is a modified/simplified version of the adult CDAI. It uses a single day's reading for diary entries and excludes three variables (body weight, haematocrit and use of drugs for diarrhoea). Code values are added together rather than summing the products of code values and coefficients. Scores range from 0 to 20. The CDAI can be predicted reasonably well from the HBI.[20] Other instruments derived from the CDAI are: the Cape Town Index (CTI), which includes parameters on subjective symptoms, physician clinical findings and laboratory data; the three-variable version of the CDAI used for survey research; and the Van Hees Index (VHI), which includes laboratory parameters, sex (male or female) and seven clinical features and excludes subjective patient related items such as well-being and pain.

For peer review only - http://bmjopen.bmj.com/site/about/guidelines.xhtml

The PDAI was developed to account for the morbidity and impairment of quality of life of patients with perianal disease, and to evaluate the effectiveness of perianal disease treatment. Variables include discharge, pain/restriction of activities, restriction of sexual activity, type of perianal disease (including number of fistulas) and degree of induration. Scores range from 0 to 20.[21]

The reliance on traditional disease activity measures (such as the CDAI) to measure treatment effectiveness fails to take into account the impaired quality of life experienced by Crohn's disease patients. The IBDQ is a 32 item health related quality of life measure. The questionnaire evaluates general activities of daily living, intestinal function, social performance, personal interactions and emotional status. Four-dimensional scores cluster items under bowel function, emotional function, systemic function and social function. Scores range from 32 to 224.[22]

The CDEIS was developed to take into account endoscopic data, such as lesion severity, when assessing severity of the disease. Variables include the presence or absence of deep or superficial ulceration in various segments of the intestinal tract, the surface involved (in cm), surface ulcerated (in cm) and presence of ulcerated stenosis. Scores range from 0 to 30.[23]

Clinical studies have variously defined a clinical response as a decrease in CDAI score of 50, 60, 70 or 100 points. In 2000 the FDA and EMEA suggested that a meaningful decrease in the CDAI score is a decrease of 100 points.[18]"{#19}

Working definitions of disease severity have been developed by the Practice Parameters Committee of the American College of Gastroenterology (2001).[11] These are:-

Mild-moderate disease:

- *"Mild-moderate disease applies to ambulatory patients able to tolerate oral alimentation without manifestations of dehydration, toxicity (high fevers, rigors, prostration), abdominal tenderness, painful mass, obstruction, or >10% weight loss"*

Moderate-severe disease:

- *"Moderate-severe disease applies to patients who have failed to respond to treatment for mild-moderate disease or those with more prominent symptoms of fever, significant weight loss, abdominal pain or tenderness, intermittent nausea or vomiting (without obstructive findings), or significant anaemia."*

Severe-fulminant disease:

For peer review only - http://bmjopen.bmj.com/site/about/guidelines.xhtml

- *"Severe-fulminant disease refers to patients with persisting symptoms despite the introduction of steroids as outpatients, or individuals presenting with high fever, persistent vomiting, evidence of intestinal obstruction, rebound tenderness, cachexia, or evidence of an abscess."*

Remission:

- *"Remission" refers to patients who are asymptomatic or without inflammatory sequelae and includes patients who have responded to acute medical intervention or have undergone surgical resection without gross evidence of residual disease. Patients requiring steroids to maintain well-being are considered to be 'steroid-dependent' and are usually not considered to be 'in remission'."*

Anti-TNF monitoring in Crohn's disease

Crohn's disease is associated with elevated levels of the immune-regulatory protein TNFα. The reasons for this elevation in Crohn's disease is still largely unknown. Anti-TNF therapies have been shown to block the action of TNFα and to improve outcomes for some patients. Patients receive anti-TNF therapy after failed attempts to improve the condition with first line glucocorticosteroids, 5-aminosalicylates, antibiotics and second line treatment (e.g., methothrexate). These patients have severe symptoms and they are at the end of the patient pathway with the only alternative option being surgery.

Like other treatment regimens anti-TNF treatment aims to induce remission (induction therapy) and prevent relapse (maintenance therapy). However failure to induce a response and relapse or loss of response are common. Approximately 10% of patients per year loose response to anti-TNF drugs.[24] The annual risk of response loss per patient has been estimated at about 13%.[25] During "episodic" infliximab therapy about 37-61% lose response.[26] Mechanisms of loss of response to anti-TNF agents and of failure to respond are still mainly unclear, however the fact that some patients generate immune responses to therapy offers one plausible contributory explanation. However other pharmacodynamics mechanisms may reduce the drug below therapeutic levels, furthermore there may be alternative secondary pathways of inflammation independent of TNFα that operate in some patients rendering anti-TNF of little use.

During scheduled infliximab therapy the incidence of antibodies is 6-16%.[27, 28] Anti-TNF antibody formation in patients treated with Infliximab has been shown to be as high as 37-61%.[29] Concomitant immunosuppressive therapy may decrease the formation of ADAbs.[26, 27, 29] Candidate risk factors for ADAb production include hereditary predisposition, a dysfunctional immune system, experience of infection(s) that trigger an abnormal response, smoking, environmental factors such as sanitation.

For peer review only - http://bmjopen.bmj.com/site/about/guidelines.xhtml

The ELISA assays could be used in good responders (i.e., those responding to initial induction course of anti-TNF treatment) as well as in patients with secondary loss of response (i.e., those initially responding to anti-TNF treatment but loosing this response over time). The use of these technologies provides a clinician with potentially useful information that may guide individual patient's future treatment. Such information may aid in anticipating the loss of response in responders, while for non-responders such analyses may help in estimating the likelihood of various candidate reasons for primary non-response or secondary loss of response. For example in non-responders with low levels of drug and high levels of ADAbs the loss or lack of response may be surmised to be due to rapid clearance of the drug due to action of ADAbs; on the other hand a low level of anti-TNF in the absence of ADAbs may be suggestive of non-immune mechanisms of rapid drug clearance, while high levels of drug in absence of antibodies in non-responders may be suggestive of a TNFα-independent pathology for the condition in a particular patient. Algorithms for future treatment based on anti-TNF and ADAb estimates have been published.

In theory the application of the tests in conjunction with an appropriate algorithm for treatment based on test results:

- May improve quality of life and other outcomes (e.g., faster healing of flare-ups, reduced abdominal pain and associated diarrhoea)
- May optimise the treatment plan (facilitate adoption of the most suitable future treatment for individual patients; this might involve a switch to an alternative anti-TNF or a biologic with an alternative mechanism of action)
- May minimise the risk of drug overdose and associated adverse events
- May allow earlier de-escalation of therapy, leading to a reduction in the overall drug used
- May help to reduce the amount of drugs used inappropriately, unnecessary hospital visits, risk of surgery, and associated costs

<u>Crohn's disease: Management and Care pathway</u>

The treatment of Crohn's disease is complex, which in general aims at: a) reducing symptoms through induction and maintenance of remission, b) minimising drug-related toxicity, and 3) reducing the risk of surgery. The management options for Crohn's disease include drug therapy (e.g., glucocorticosteroids, 5-aminosalicylate, antibiotics, immunosuppressives, TNFα inhibitors), enteral nutrition, smoking cessation and, in severe or chronic active disease, surgery (Table 5). The choice of treatment amongst the available drugs is influenced by patient age, site and activity of disease, previous drug tolerance and response to treatment, and the presence of extra-intestinal manifestations.[30, 31] Enteral nutrition is widely used as a first line treatment to facilitate growth and development in children and young people. Adjuvant therapy commonly coexists and includes

For peer review only - http://bmjopen.bmj.com/site/about/guidelines.xhtml

management of extra-intestinal manifestations, antibiotics, corticosteroids or immunomodulator therapy. Between 50% and 80% of people with Crohn's disease require surgery due to complications such as strictures causing symptoms of obstruction, fistula formation, perforation or failure of medical therapy.[32]

Once remission has been achieved, maintenance therapy can be considered following assessment of the course and extent of Crohn's disease, effectiveness and tolerance of previous treatments, presence of biological or endoscopic signs of inflammation, and potential for complications.

Table 5. Treatment options for patients with Crohn's disease[33]

Patient group	Treatment Line and Treatment
**Ileocaecal disease not fistulating with <100 cm of bowel affected: initial presentation or relapse**	
• mildly active	1st observation with monitoring or budesonide or 5-ASA therapy
• moderately active: initial presentation or non-corticosteroid-dependent/-refractory relapse	1st budesonide and/or 5-ASA therapy, or conventional oral corticosteroids (use previously effective treatment for relapse)
	2nd immunomodulator therapy + oral corticosteroid taper
	3rd anti-TNF therapy + oral corticosteroid taper
• moderately active: relapse corticosteroid-dependent/-refractory	1st consideration of early initiation of anti-TNF therapies + oral corticosteroid taper
	2nd surgery
• severely active: initial presentation or non-corticosteroid-dependent/-refractory relapse	1st hospitalisation + oral or intravenous conventional corticosteroids + consideration of surgery
	2nd anti-TNF therapy or surgery
• severely active: relapse corticosteroid-dependent/-refractory	1st hospitalisation + consideration of early initiation of anti-TNF therapy or surgery
**Colonic disease not fistulating: initial presentation or relapse**	
• mildly active	1st 5-ASA therapy or alternatively oral corticosteroids
	2nd surgery
• moderately or severely active:	1st oral or intravenous corticosteroids + immunomodulator therapy + consideration for surgery

For peer review only

For peer review only - http://bmjopen.bmj.com/site/about/guidelines.xhtml

initial presentation or non-corticosteroid-dependent/-refractory relapse	2nd anti-TNF therapy + consideration for surgery
	3rd surgery
• moderately or severely active: relapse corticosteroid-dependent/-refractory	1st early initiation of anti-TNF therapy or consideration for surgery
	2nd surgery
**Extensive small bowel disease (>100 cm of bowel affected) not fistulating: initial presentation or relapse**	1st oral corticosteroids + early introduction of immunomodulators
**Upper GI disease (oesophageal and/or gastroduodenal disease) not fistulating: initial presentation or relapse**	1st proton pump inhibitor
**Perianal or fistulating disease: initial presentation or relapse**	
• simple perianal fistula: symptomatic	1st loose seton + drainage of perianal abscess if present
• complex perianal fistulae	1st loose seton placement + drainage of perianal abscess if present
• non-perianal fistulae	1st multidisciplinary input + supportive care

Abbreviations: 5-ASA 5-Aminosalicylic Acid, TNF tumour necrosis factor, GI gastrointestinal

For peer review only

Induction of remission

Usually, at first presentation, people with active Crohn's disease are recommended monotherapy with a conventional glucocorticosteroid (prednisolone, methylprednisolone or intravenous hydrocortisone), which is aimed at inducing remission as a first line treatment. Alternatively, treatment with budesonide, 5-ASA, or enteral nutrition may be offered to a group of people who do not choose to take or who are intolerant to glucocorticosteroid therapy.

The addition of an immunosuppressant (azathioprine, mercaptopurine or methotrexate) to a conventional glucocorticosteroid or budesonide as an add-on therapy for inducing remission is recommended for people who have active Crohn's disease and have experienced two or more inflammatory exacerbations in a 12-month period, or in whom the glucocorticosteroid dose cannot be tapered. As advised in the current online version of the British national formulary (BNF)[34] or British National Formulary for Children (BNFC),[34] the effects of azathioprine, mercaptopurine, and methotrexate as well as levels of neutropenia (in people on azathioprine or mercaptopurine) should be monitored.

For peer review only - http://bmjopen.bmj.com/site/about/guidelines.xhtml

Adults with severe active Crohn's disease who fail to respond to the first line of treatment with conventional therapy (e.g., immunosuppressive drugs, corticosteroids), or who are intolerant of or have contraindications to the above-mentioned conventional therapy, anti-TNF alpha agents (infliximab and adalimumab) are recommended as treatment options within their licensed indications. The administration of anti TNF alpha agents is recommended until 12 months after the start of treatment or until treatment failure (including the need for surgery), depending on whichever occurs first. Periodic reassessment and monitoring of disease activity (at least every 12 months) is advised in order to ascertain the clinical appropriateness of ongoing treatment. Usually, treatment course needs to be initiated with the less expensive drug by considering drug administration costs, dose, and product price per dose. The use of anti-TNF-alpha drugs for the treatment of Crohn's disease is covered in the 2010 NICE technology appraisal guidance 187 (Infliximab (review) and adalimumab for the treatment of Crohn's disease).[35]

Surgery should be considered as an alternative to medical treatment early in the course of the disease for people (adults, children, and young people) whose disease is limited to the distal ileum or have growth impairment despite optimal medical treatment and/or refractory disease (children and young people).

Maintenance of remission

People with Crohn's disease in remission can be managed with or without maintenance treatment. The options for maintenance therapy (including treatment or no treatment) need to be discussed with patients, their parents, and/or carers. The discussion should include risk of inflammatory exacerbations (with and without drug treatment) and the potential side effects of drug treatment. People who decline to receive maintenance treatment should agree with follow-up plans (e.g., frequency and duration of visits) and receive information on symptoms related to relapse (e.g., unintended weight loss, abdominal pain, diarrhoea, general ill-health) to ensure timely consultations with their healthcare professional.

People with Crohn's disease in remission who choose to receive maintenance therapy may be offered azathioprine or mercaptopurine monotherapy if their remission was induced using a conventional glucocorticosteroid or budesonide. Methotrexate can be offered to people whose remission was induced by methotrexate or people who did not tolerate azathioprine or mercaptopurine for maintenance therapy or those who have contraindications to azathioprine or mercaptopurine. Treatment with 5-ASA can be recommended to maintain remission after surgery.

For peer review only - http://bmjopen.bmj.com/site/about/guidelines.xhtml

If remission has been achieved with anti-TNF medication, then maintenance with anti-TNF with or without combination with another immunomodulator can be recommended. Continuation of treatment with infliximab or adalimumab during remission is advised only if there is evidence of ongoing active disease given clinical symptoms, biological markers, including endoscopy if necessary. The balance between harms and benefits of ongoing treatment should be taken into account. People who relapse after treatment is stopped have the option to start this treatment again.

3 Decision questions and objectives

3.1 Decision questions

The decision questions for this project are shown in the box below:

1. *Does concurrent testing of TNF inhibitor levels and antibodies to TNF inhibitors represent a clinically and cost-effective use of NHS resources in people with Crohn's disease whose disease responds to treatment with TNF inhibitor?*
Testing will be carried out:
a) 3 to 4 months after start of treatment or
b) 3 to 4 months and every 12 months from start of treatment

2. *Does concurrent testing of TNF inhibitor levels and antibodies to TNF inhibitors represent a clinically and cost-effective use of NHS resources in people with Crohn's disease who experience secondary loss of response during maintenance treatment with TNF inhibitor?*

3. *Does testing of TNF inhibitor levels followed by reflex testing of antibodies to TNF inhibitors if drug level is undetectable represent a clinically and cost-effective use of NHS resources in people with Crohn's disease whose disease responds to treatment with TNF inhibitor?*
Testing will be carried out:
a) 3 to 4 months after start of treatment or
b) 3 to 4 months and every 12 months from start of treatment

4. *Does testing of TNF inhibitor levels followed by reflex testing of antibodies to TNF inhibitors if drug level is undetectable represent a clinically and cost-effective use of NHS resources in people with Crohn's disease who experience secondary loss of response during maintenance treatment with TNF inhibitor?*

3.2 Objectives

Given these decision questions the four main objectives for this report are:

For peer review only - http://bmjopen.bmj.com/site/about/guidelines.xhtml

A) To provide a technical description, and (where evidence allows) an evaluation, of the listed intervention tests used for Crohn's disease in therapeutic monitoring of TNF inhibitors (infliximab and adalimumab) and their respective antibodies. This will include what the assays measure and the mechanisms of the assays.

In addition, published studies which include a comparison (including relative test performance) of two or more intervention tests, or which compare an intervention test with a test method which can be used to perform a linked evidence assessment will be reviewed and critiqued. Data submitted by the manufacturers will be used to supplement published studies if deemed of sufficient detail and quality.

B) To describe algorithms used in studies which include data on one or more intervention test or on a test which allows a linked evidence approach to be performed (i.e., algorithms used in studies identified in Objective C). The studies are required to provide an algorithm and report clinical outcomes for the management of patients with Crohn's disease following measurement of serum levels of anti-TNF drug and anti-drug antibodies. To compare the algorithms used following therapeutic drug monitoring to the algorithms specified in the TAXIT study for responders,[36] and in the reporting of secondary loss of response (algorithm adapted from the study by Scott and Lichtenstein, 2014[37]).

C) To systematically review the literature comparing the clinical effectiveness of [a] the intervention assays for anti-TNF agents and/ or for ADAbs used in conjunction with a treatment algorithm in Crohn's patients treated with infliximab or adalimumab; with [b] standard care (no tests performed or test-informed algorithm used) in Crohn's disease patients treated with infliximab or adalimumab. Where evidence exists on the comparison of standard care with other test assays used in conjunction with an algorithm, this will be assessed and critiqued and test performance will be compared with that of the study interventions (LISA-TRACKER ELISA kits, TNFα-Blocker ELISA kits, and Promonitor ELISA kits) (see Objective A).

D) To assess the cost-effectiveness of employing anti-TNF monitoring with LISA-TRACKER ELISA kits, TNFα-Blocker ELISA kits, and Promonitor ELISA kits in patients with Crohn's disease compared with standard care (no anti-TNF monitoring). Where direct evidence is unavailable for this comparison, or where such a comparison is not well supported with evidence, a linked approach to evidence will be considered (see Objective C above) in which evidence of clinical effectiveness is taken from studies using alternative test methodology and an assessment is made of the relative performance this methodology relative to the intervention assays.

For peer review only - http://bmjopen.bmj.com/site/about/guidelines.xhtml

4. Methods for assessing clinical effectiveness

Systematic review methods will follow the principles outlined in the Centre for Reviews and Dissemination (CRD) guidance for undertaking reviews in health care[38] and the NICE Diagnostic Assessment Programme manual.[39]

4.1 Identification and selection of studies

4.1.1 Search strategies for clinical effectiveness

Scoping searches have been undertaken to inform the development of the search strategies. Additional phrases were added to the scoping searches to broaden the search to find other relevant articles that had no terms for the test name or type of test (e.g., Baert et al., 2003[26]) or population (e.g., Vande Casteele et al., 2012[40]) in title, abstract or indexing. Additional searches will be carried out where necessary. Searches for studies for cost and quality of life will be developed separately. An iterative procedure was used, with reference to scoping searches undertaken by information specialists at NICE. A copy of the main draft search strategy that is likely to be used in the major databases is provided in Appendix 3. This strategy may be further refined and other appropriate concepts may be added. This search strategy developed for Medline will be adapted as appropriate for other databases. All retrieved papers will be screened for potential inclusion.

The search strategy will comprise the following main elements:

- Searching of electronic bibliographic databases
- Contact with experts in the field
- Scrutiny of references of included studies
- Screening of manufacturer's and other relevant organisations' websites for relevant publications

Bibliographic databases will include:

MEDLINE; MEDLINE In-Process & Other Non-Indexed Citations; EMBASE; Cochrane Library (including Cochrane Systematic Reviews, DARE, CENTRAL, NHS EED, and HTA databases); Science Citation Index and Conference Proceedings (Web of Science); Index to Theses; DART-Europe; Dissertations & Theses; NIHR Health Technology Assessment Programme; PROSPERO (International Prospective Register of Systematic Reviews).

The following trial and patent databases will also be searched: Current Controlled Trials; ClinicalTrials.gov; UKCRN Portfolio Database; WHO International Clinical Trials Registry Platform; Espacenet (European Patent Office); Patentdocs (US Patents database).

For peer review only - http://bmjopen.bmj.com/site/about/guidelines.xhtml

Specific conference proceedings, to be selected with input from clinical experts and Specialist Committee Members, will be checked for the last five years.

The online resources of various health services research agencies, regulatory bodies, professional societies and manufacturers will be consulted via the Internet. These are likely to include:

- International Network of Agencies for Health Technology Assessment (INAHTA) Publication http://www.inahta.org/
- FDA medical devices: http://www.fda.gov/MedicalDevices/DeviceRegulationandGuidance/Databases/default.htm
- European Commission medical devices http://ec.europa.eu/health/medical-devices/
- Theradiag http://www.theradiag.com/en/
- Immundiagnostik http://www.immundiagnostik.com/en
- Proteomika http://www.proteomika.com/
- American college of gastroenterology http://gi.org/

This will be supplemented by web searching on specific test names using Google and a meta-search engine.

The reference lists of included studies and relevant review articles will be checked. Citation searches of selected included studies will be undertaken using Scopus. Identified references will be downloaded in Endnote X7 software. Included papers will be checked for errata using PubMed.

4.1.2 Inclusion and exclusion of relevant studies

Inclusion of relevant studies to address Objective A

Detailed information will be sought from manufacturers regarding mechanisms and reactants (in particular specificities and properties of antibodies and other reagents) employed in ELISA tests and radioimmunoassay, mobility shift assays and cell reporter tests (if used for a linked evidence approach).

In addition published studies which describe the intervention tests and tests used for a linked evidence approach will be identified. Those providing useful information about test mechanisms that is different or additional to that supplied by manufacturers of tests will be included. Assessment of inclusion will be based on the judgement of two reviewers.

For peer review only - http://bmjopen.bmj.com/site/about/guidelines.xhtml

Studies which compare test performance of two or more tests will be included either if they compare two or more intervention tests, or compare an intervention test with a test method which can be used to perform a linked evidence assessment.

All study designs will be considered for inclusion.

Inclusion criteria for studies to address Objective B

Studies that report an algorithm with the use of one of the intervention tests for the management of patients with Crohn's disease following measurement of serum levels of anti-TNF drug and anti-drug antibodies (infliximab or adalimumab). All study designs will be considered for inclusion.

Inclusion criteria for studies to address Objective C

Studies that satisfy the following criteria will be included:

Population	Crohn's disease patients (adults and children) receiving infliximab or adalimumab. If the evidence on Crohn's disease patients is limited, mixed patient groups containing Crohn's disease and ulcerative colitis patients will be included even if results are not reported separately. The limitations following from this will be discussed.
Intervention	Use of LISA-TRACKER ELISA kits, TNFα-Blocker ELISA kits, and Promonitor ELISA kits to estimate plasma or sera levels of anti-TNF agents and / or of ADAbs in which test results are employed in conjunction with a treatment algorithm (Table 6). Other assay methods will be considered should a linked evidence approach be adopted (Table 6).
Comparator	Standard care (Treatment decisions made on clinical judgement without measuring levels of TNF inhibitor and antibodies to TNF inhibitors).
Outcome	Any patient outcome (e.g., CDAI score based response rate, any measure of change in severity of Crohn's disease including physicians global assessment; Duration of response, relapse and remission; Rates of hospitalisation; Rates of surgical intervention; Time to surgical intervention; Adverse effects of treatment; Health related quality of life; and secondary if two strategies compared are found clinically equivalent: Time to result; Number of inconclusive results; Frequency of dose adjustment; Frequency of treatment switch).

For peer review only

For peer review only - http://bmjopen.bmj.com/site/about/guidelines.xhtml

Study design All study designs will be considered for inclusion.

Healthcare setting Secondary and tertiary care.

Meeting abstracts will be included if they provide sufficient data on type of ELISA assay, patient group, algorithm, measurements from assays and clinical outcomes.

Table 6. Assay methods included as interventions in the review

LISA-TRACKER assay kits (Theradig/Alpha Laboratories)

- LISA-TRACKER Adalimumab (LTA002)
- LISA-TRACKER Infliximab (LTI002)
- LISA-TRACKER anti-Adalimumab (LTA003)
- LISA-TRACKER anti-Infliximab (LTI003)
- LISA-TRACKER Duo Adalimumab (LTA005)
- LISA-TRACKER Duo Infliximab (LTI005)

Immundiagnostik TNFα-Blocker ELISA kits (Immundiagnostik/BioHit Healthcare):

- Immundiagnostik TNFα-Blocker ADA, antibodies against infliximab (e.g. Remicade®) ELISA (K9650)
- Immundiagnostik TNFα-Blocker ADA, antibodies against adalimumab (e.g. Humira®) ELISA (K9652)
- Immundiagnostik TNFα-Blocker ADA, TOTAL antibodies against infliximab (e.g. Remicade®) ELISA (K9654)
- Immundiagnostik TNFα-Blocker ADA, TOTAL antibodies against adalimumab (e.g. Humira®) ELISA (K9651)
- Immundiagnostik TNFα-Blocker monitoring, infliximab drug level (e.g. Remicade®) ELISA (K9655)
- Immundiagnostik TNFα-Blocker monitoring, adalimumab drug level (e.g. Humira®) ELISA (K9657)

Promonitor ELISA kits (Proteomika):

- Promonitor-ADL ELISA (5080230000)
- Promonitor-IFX ELISA (5060230000)
- Promonitor-ANTI-ADL ELISA (5090230000)
- Promonitor-ANTI-IFX ELISA (5070230000)

For Objective C test methods that are not included as an intervention but have evidence comparing it

For peer review only - http://bmjopen.bmj.com/site/about/guidelines.xhtml

to an intervention test and evidence reporting clinical outcomes, should be included for the purpose of performing linked evidence modelling only (including: radioimmunoassays, cell reporter assays, liquid-phase mobility shift assays and in-house ELISAs).

4.2 Review strategy

The general principles recommended in the PRISMA statement will be considered.[41] Records rejected at full text stage and reasons for exclusion will be documented. Two reviewers will independently screen the titles and abstracts of all records identified by the searches and discrepancies will be resolved through discussion. Disagreement will be resolved by retrieval of the full publication and consensus agreement. Full copies of all studies deemed potentially relevant, will be obtained and two reviewers will independently assess these for inclusion; any disagreements will be resolved by consensus or discussion with a third reviewer.

4.3 Data extraction strategy

Data will be extracted by one reviewer, using a piloted, data extraction form. A second reviewer will check the extracted data and any disagreements will be resolved by consensus or discussion with a third reviewer. Examples of data extraction sheets for patient-based and diagnostic accuracy studies are provided in Appendix 4.

4.4 Quality assessment strategy

Where appropriate, the quality of diagnostic accuracy studies will be assessed using QUADAS-2 (see Appendix 5).[42] As a broad range of study designs have been identified in the scoping searches, the use of a single checklist, in contrast to individual checklists for each study design, is considered appropriate. The Downs and Black checklist[43] will therefore be used to assess the quality of non-randomised studies meeting the inclusion criteria (see Appendix 5). This 27-item checklist provides both an overall score for study quality and a profile of scores not only for the quality of reporting, internal validity (bias and confounding) and power, but also for external validity. RCTs will be quality appraised using the Cochrane risk of bias tool (see Appendix 5).[44] The results of the quality assessment will provide an overall description of the quality of the included studies and will provide a transparent method of recommendation for design of any future studies. Quality assessment will be undertaken by one reviewer and checked by a second reviewer, any disagreements will be resolved by a third reviewer through discussion.

4.5 Methods of analysis/synthesis

Objective A

Narrative descriptions of tests in tables and texts will be undertaken.

For peer review only - http://bmjopen.bmj.com/site/about/guidelines.xhtml

Objective B

Algorithms will be narratively described and compared to the algorithm used in the TAXIT study (for good responders),[36] and the algorithm adapted from Scott and Lichtenstein (2014) (for secondary loss of response).[37] Non-compliant patients may be considered additionally in the algorithms. Time of testing, sequence of testing (drug and antibodies), sequence of analysis as well as thresholds used in the algorithms will be considered to address the research questions.

Objective C

Depending on the available evidence, analyses will be stratified according to the type of ELISA assay, type of drug (infliximab or adalimumab) and patient group (patients with secondary loss of response and patients with good response to anti-TNF treatment).

Study, treatment, population, and outcome characteristics will be summarised and compared qualitatively and, where possible, quantitatively in text, graphically and in evidence tables. Pooling studies results by meta-analysis will be considered. Where meta-analysis is considered unsuitable for some or all of the data identified (e.g., due to the heterogeneity and/or small numbers of studies), we will employ a narrative synthesis. Typically, this will involve the use of text, graphs and tables (as appropriate) to summarise data. These will allow the reader to consider any outcomes in the light of differences in study designs and potential sources of bias for each of the studies being reviewed. Studies will be organised by objective addressed. A detailed commentary on the major methodological problems or biases that affected the studies will also be included, together with a description of how this may have affected the individual study results.

For Objective C we aim to identify studies that compare treatment decisions made on clinical judgement without measuring levels of TNF inhibitor and antibodies to TNF inhibitors with treatment decisions based on measurement of TNF inhibitor and antibodies to TNF inhibitors. We will consider using a linked-evidence approach[45] in which studies report patient management informed by measurement of anti-TNF and antibodies by other methods (e.g., radioimmunoassay, liquid-phase mobility shift assay, in-house ELISAs); this will require an assessment of evidence relating to the comparable performance of ELISA assays with radioimmunoassay, liquid-phase mobility shift assays and in-house ELISAs.

In studies where an ELISA has been used but there is no comparator arm, or the comparator arm is a convenience sample (retrospective/historical population), outcomes will be listed and appraised. Time of testing, sequence of testing (drug and antibodies) and sequence of analysis will be considered to address the research questions.

For peer review only - http://bmjopen.bmj.com/site/about/guidelines.xhtml

5. Methods for synthesising cost-effectiveness evidence

5.1 Identifying and reviewing published cost-effectiveness studies

Published cost-effectiveness studies will be reviewed. All papers which present findings on the costs and outcomes of LISA-TRACKER ELISA kits, TNFα-Blocker ELISA kits, and Promonitor ELISA kits for measuring levels of TNF inhibitors and of anti-drug antibodies will be reviewed in detail. Information on assay procedures additional to ELISA methods will be sought for the purposes of providing data for a linked approach to evidence synthesis should this be required.

5.1.1 Search strategy and data extraction

A comprehensive search of the literature for published economic evaluations (including any existing models), cost studies and quality of life (utility) studies will be performed. The search strategy used will be based on the strategy developed for the clinical effectiveness review (see Appendix 3).

Databases will include:

- MEDLINE (Ovid)
- MEDLINE In-Process Citations and Daily Update (Ovid)
- EMBASE (Ovid)
- NHS Economic Evaluation Database (NHS EED) (Cochrane Library)
- Science Citation Index (Web of Knowledge)
- Cost-effectiveness analysis (CEA) registry
- Research Papers in Economics (REPAC)

Additional searches will be performed where necessary to identify other relevant information to support the development of an economic model for this project, these may be directed towards - costs, utilities and transition probabilities as required.

Data will be extracted by one reviewer and checked by a second, using a standardised data extraction form for the economic studies; this will be developed to summarise the main characteristics of the studies and to capture useful data that can inform the economic model. Any discrepancies will be resolved by discussion. If this is not feasible, a third reviewer will be consulted.

The quality of any full economic evaluation studies will be assessed using the CHEERS checklist (see Appendix 5).[46] Any studies containing an economic model will be further assessed using the framework for the quality assessment of decision analytic modelling (see Appendix 5).[47]

For peer review only - http://bmjopen.bmj.com/site/about/guidelines.xhtml

5.2 Evaluation of costs, quality of life and cost-effectiveness

5.2.1 Model structure, time horizon and transition probabilities

In developing the economic model we will consult the previous Health Technology Assessment report (HTA) conducted by Dretzke and colleagues (2011).[48] The main aim of this HTA report was to assess the cost-effectiveness of anti-TNFs in the management of moderate-to-severe Crohn's disease in the UK National Health Service (NHS). The authors developed a Markov model from an NHS and Personal Social Services (PSS) perspective to estimate the incremental cost per quality-adjusted life year (QALY) gained for both adalimumab and infliximab compared with standard care. The assumptions used in the model for the appraisal of Infliximab (review) and adalimumab for the treatment of Crohn's disease (technology appraisal 187)[48] may be used to inform the development of a de novo model. We will create a Markov-type model to assess the cost-effectiveness of LISA-TRACKER ELISA kits, TNFα-Blocker ELISA kits, and Promonitor ELISA kits compared with standard care. The perspective of the model will be that of the NHS and PSS. To assess the cost-effectiveness, the intervention tests (LISA-TRACKER ELISA kits, TNFα-Blocker ELISA kits, and Promonitor ELISA kits) will be compared with standard care in the following populations:

- In patients with secondary loss of response to anti-TNF treatment
- In patients who respond well to anti-TNF treatment

The following comparisons will be made where possible:

- Concurrent versus reflex testing
- Testing conducted every 3 to 4 months versus testing conducted at 3 to 4 months then yearly (in patients who respond well to anti-TNF treatment)

If data permits, we will compare the different LISA-TRACKER ELISA kits, TNFα-Blocker ELISA kits, and Promonitor ELISA kits with each other. In the absence of sufficient clinical data for specific ELISAs we will assume equal assay performance and compare ELISAs on the basis of cost only.

If data permits, a linked evidence approach will be adopted to compare LISA-TRACKER ELISA kits, TNFα-Blocker ELISA kits, and Promonitor ELISA kits with standard care in which clinical outcomes for the intervention arm are taken from studies in which the assay procedure was not one of the intervention assays; this will involve an assessment of the comparability of LISA-TRACKER ELISA kits, TNFα-Blocker ELISA kits, or Promonitor ELISA kits performance with that of the alternative procedure.

The model will have a one-year time horizon in line with the previous HTA report[48] and other studies we have found during our initial scoping search (e.g., Velayos et al., 2013).[49]

For peer review only - http://bmjopen.bmj.com/site/about/guidelines.xhtml

It is anticipated that information from the clinical effectiveness analyses will help inform the probabilities for each of the clinical pathways. Sensitivity analyses will be conducted in areas of uncertainty.

5.2.2 Resource use and costs

Resource use and costs will be estimated in line with the DAP programme manual. Information on resource use and costs associated with the different patient pathways (e.g., comparing clinical pathways followed when LISA-TRACKER ELISA kits, TNFα-Blocker ELISA kits, or Promonitor ELISA kits are employed, versus standard care pathway etc.) will be collected from systematic reviews of the literature, discussions with individual manufacturers and hospitals and if need be, by eliciting expert clinical advice. Any remaining gaps for resource use parameters will be filled by assumptions made by the research team.

Unit costs data will be based on national data were possible. For the different LISA-TRACKER ELISA kits, TNFα-Blocker ELISA kits, and Promonitor ELISA kits, costs will be from published list prices from the NHS supply chain, from the NHS reference costs,[50] or discussions with individual manufacturers or hospitals. Costs of consultations with secondary care staff will be drawn from Unit Costs of Health and Social Care[51] and drug costs will be obtained from the British National Formulary.[34]

5.2.3 Health outcomes

Health outcomes and utility data will be derived from the literature review including the previous HTA report and other sources. If direct measurements of utility or choice-based multi-attribute utility scales (such as the EQ-5D or SF-6D) suitable for calculation of QALYs for the economic model are not reported, we may need to use one of the algorithms for mapping from a clinical measure (e.g. CDAI) to a measure of utility. If insufficient information is available for utilities it may have to be elicited from an expert clinical panel or by assumptions made by the research team.

5.2.4 Cost-effectiveness analysis

The results of the cost-effectiveness analysis will be presented as an incremental cost per QALY gained for LISA-TRACKER ELISA kits, TNFα-Blocker ELISA kits, and Promonitor ELISA kits compared with standard care. If the data allows us to compare LISA-TRACKER ELISA kits, TNFα-Blocker ELISA kits, and Promonitor ELISA kits with each other, then we will undertake a rank comparison and exclude any options which are dominated or extended dominated. It may be necessary, in the absence of suitable clinical outcome data, to rank ELISAs on the basis of cost only.

We will use both simple and probabilistic sensitivity analysis to explore the robustness of the results and to estimate the impact of uncertainty over model parameters. The simple sensitivity analysis will

For peer review only - http://bmjopen.bmj.com/site/about/guidelines.xhtml

be used to assess the robustness of the results to changes in deterministic parameters such as costs, and utilities. The results from the probabilistic sensitivity analysis will be presented as cost-effectiveness acceptability curves. Decisions regarding mutually exclusive alternatives will be reflected using cost-effectiveness planes and cost-effectiveness acceptability curves or frontiers.

If a longer time horizon is chosen (more than one year), both costs and outcomes will be discounted using the recommended 3.5% discount rate by HM Treasury.

6. Handling of information from manufacturers

All data submitted by the manufacturers/sponsors will only be considered if received by the External Assessment Group before 27 January 2015. Data arriving after this date will not be considered. Any data that meets the inclusion criteria stated will be extracted and quality assessed as stated in the methods section of this protocol.

Any 'commercial in confidence' data provided by manufacturers, and specified as such, will be highlighted in blue and underlined in the assessment report (followed by company name in parentheses). Any 'academic in confidence' data provided by manufacturers, and specified as such, will be highlighted in yellow and underlined in the assessment report. All confidential data used in the cost-effectiveness models will also be highlighted.

7. Competing interests of authors and advisors

None of the authors have any competing interests.

8. Timetable/milestones

Draft assessment protocol	**06/10/2014**
Final protocol	**28/10/2014**
Progress report	**27/01/2015**
Draft assessment report	**24/03/2015**
Final assessment report	**23/04/2015**

9. Team members' contributions

Warwick Evidence is an External Assessment Group located within Warwick Medical School. Warwick Evidence brings together experts in clinical and cost effectiveness reviewing, medical statistics, health economics and modelling. The team planned for the work include:

Lead: Mrs Karoline Freeman

For peer review only - http://bmjopen.bmj.com/site/about/guidelines.xhtml

Title: Research Fellow

Address: Warwick Evidence, Populations, Evidence and Technologies, Division of Health Sciences, Warwick Medical School, University of Warwick, Coventry CV4 7AL

Tel: 02476 574026

Email: K.Freeman@warwick.ac.uk

Contribution: Protocol development, assessment for eligibility, quality assessment of trials, data extraction, data entry, and report writing

Name: Dr Martin Connock

Title: Senior Research Fellow

Address: Warwick Evidence, Populations, Evidence and Technologies, Division of Health Sciences, Warwick Medical School, University of Warwick, Coventry CV4 7AL

Tel: 02476 574940

Email: M.Connock@warwick.ac.uk

Contribution: Protocol development, assessment for eligibility, quality assessment of trials, data analysis, statistical modelling, and report writing

Name: Dr Hema Mistry

Title: Health economist

Address: Warwick Evidence, Populations, Evidence and Technologies, Division of Health Sciences, Warwick Medical School, University of Warwick, Coventry CV4 7AL

Tel: 02476 574490

Email: Hema.Mistry@warwick.ac.uk

Contribution: Protocol development, health economics modeller, data analysis, and report writing

Name: Dr Sian Taylor-Phillips

Title: Senior Research Fellow

Address: Warwick Evidence, Populations, Evidence and Technologies, Division of Health Sciences, Warwick Medical School, University of Warwick, Coventry CV4 7AL

Tel: 02476 575882

Email: S.Taylor-Phillips@warwick.ac.uk

Contribution: Protocol development, data analysis, and report writing

Name: Ms Rachel Court

Title: Information Specialist

Address: Warwick Evidence, Populations, Evidence and Technologies, Division of Health Sciences, Warwick Medical School, University of Warwick, Coventry CV4 7AL

For peer review only - http://bmjopen.bmj.com/site/about/guidelines.xhtml

Tel: 02476 522427
Email: R.A.Court@warwick.ac.uk
Contribution: Protocol development, develop search strategy and undertake the electronic literature searches

Name: Dr Alexander Tsertsvadze
Title: Senior Research Fellow
Address: Warwick Evidence, Populations, Evidence and Technologies, Division of Health Sciences, Warwick Medical School, University of Warwick, Coventry CV4 7AL
Tel: 02476 574505
Email: a_tsertsvadze@hotmail.com
Contribution: Assessment for eligibility, quality assessment of trials, data extraction, data analysis, and report writing

Name: Dr Jason Madan
Title: Assistant Professor in Health Economics
Address: Clinical Trials Unit, University of Warwick, Coventry CV4 7AL
Tel: 024761 51254
Email: j.j.madan@warwick.ac.uk
Contribution: Provide health economic modelling support, data analysis, and report writing

Name: Dr Ngianga-Bakwin Kandala
Title: Principal Research Fellow
Address: Warwick Evidence, Populations, Evidence and Technologies, Division of Health Sciences, Warwick Medical School, University of Warwick, Coventry CV4 7AL
Tel: 02476 575054
Email: N-B.Kandala@warwick.ac.uk
Contribution: Data analysis and statistical modelling

Name: Professor Aileen Clarke
Title: Director of Warwick Evidence
Address: Warwick Evidence, Populations, Evidence and Technologies, Division of Health Sciences, Warwick Medical School, University of Warwick, Coventry CV4 7AL
Tel: 02476 150189
Email: Aileen.Clarke@warwick.ac.uk
Contribution: Co-ordinate review process, protocol development, synthesis of findings and report writing

For peer review only - http://bmjopen.bmj.com/site/about/guidelines.xhtml

Name: Dr Paul Sutcliffe

Title: Associate Professor

Address: Warwick Evidence, Populations, Evidence and Technologies, Division of Health Sciences, Warwick Medical School, University of Warwick, Coventry CV4 7AL

Tel: 02476 574505

Email: p.a.sutcliffe@warwick.ac.uk

Contribution: Co-ordinate review process, protocol development, assessment for eligibility, quality assessment of trials, data extraction, data entry, data analysis, and report writing

9.1 Expert advisors

Name: Dr Ramesh P Arasaradnam

Title: Hon Assoc. Prof of Medicine and Consultant Gastroenterologist

Address: Clinical Sciences Research Institute, Clifford Bridge Road, Coventry CV2 2DX

Tel: 02476 966087

Email: r.arasaradnam@warwick.ac.uk

Contribution: Provide expert clinical advice on Crohn’s and care pathways

Name: Dr Ahmed Naher

Title: Academic clinical fellow in clinical pharmacology and therapeutics

Address: Institute of Translational Medicine, University of Liverpool

Tel: 07949170357

Email: al.naher@gmail.com

Contribution: Provide expert advice on Crohn’s and care pathways

For peer review only

For peer review only - http://bmjopen.bmj.com/site/about/guidelines.xhtml

10. References

1. Jerne NK. Towards a network theory of the immune system. *Annales d'immunologie*. 1974;**125c**(1-2):373-89.

2. Steenholdt C. Use of infliximab and anti-infliximab antibody measurements to evaluate and optimize efficacy and safety of infliximab maintenance therapy in Crohn's disease. *Danish medical journal*. 2013;**60**(4):B4616.

3. Allez M, Karmiris K, Louis E, Van Assche G, Ben-Horin S, Klein A*, et al.* Report of the ECCO pathogenesis workshop on anti-TNF therapy failures in inflammatory bowel diseases: definitions, frequency and pharmacological aspects. *Journal of Crohn's & colitis*. 2010;**4**(4):355-66.

4. Ben-Horin S, Chowers Y. Review article: loss of response to anti-TNF treatments in Crohn's disease. *Alimentary pharmacology & therapeutics*. 2011;**33**(9):987-95.

5. Cassinotti A, Travis S. Incidence and clinical significance of immunogenicity to infliximab in Crohn's disease: a critical systematic review. *Inflammatory bowel diseases*. 2009;**15**(8):1264-75.

6. Lee LY, Sanderson JD, Irving PM. Anti-infliximab antibodies in inflammatory bowel disease: prevalence, infusion reactions, immunosuppression and response, a meta-analysis. *European journal of gastroenterology & hepatology*. 2012;**24**(9):1078-85.

7. Chaparro M, Guerra I, Munoz-Linares P, Gisbert JP. Systematic review: antibodies and anti-TNF-alpha levels in inflammatory bowel disease. *Alimentary pharmacology & therapeutics*. 2012;**35**(9):971-86.

8. NHS choices. Crohn's disease. 2013 [cited 06/10/2014]; Available from: http://www.nhs.uk/Conditions/Crohns-disease/Pages/Introduction.aspx.

9. Jewell DP. Crohn's disease. *Medicine*. 2007;**35**(5):283-9.

10. Carter MJ, Lobo AJ, Travis SP. Guidelines for the management of inflammatory bowel disease in adults. *Gut*. 2004;**53 Suppl 5**:V1-16.

11. Hanauer SB, Sandborn W. Management of Crohn's disease in adults. *The American journal of gastroenterology*. 2001;**96**(3):635-43.

12. Jenkins HR. Inflammatory bowel disease. *Archives of disease in childhood*. 2001;**85**(5):435-7.

13. Silverberg MS, Satsangi J, Ahmad T, Arnott ID, Bernstein CN, Brant SR*, et al.* Toward an integrated clinical, molecular and serological classification of inflammatory bowel disease: report of a Working Party of the 2005 Montreal World Congress of Gastroenterology. *Canadian journal of gastroenterology = Journal canadien de gastroenterologie*. 2005;**19 Suppl A**:5a-36a.

14. Gasche C, Scholmerich J, Brynskov J, D'Haens G, Hanauer SB, Irvine EJ*, et al.* A simple classification of Crohn's disease: report of the Working Party for the World Congresses of Gastroenterology, Vienna 1998. *Inflammatory bowel diseases*. 2000;**6**(1):8-15.

For peer review only - http://bmjopen.bmj.com/site/about/guidelines.xhtml

15. Sostegni R, Daperno M, Scaglione N, Lavagna A, Rocca R, Pera A. Review article: Crohn's disease: monitoring disease activity. *Alimentary pharmacology & therapeutics*. 2003;**17 Suppl 2**:11-7.

16. Best WR, Becktel JM, Singleton JW, Kern F, Jr. Development of a Crohn's disease activity index. National Cooperative Crohn's Disease Study. *Gastroenterology*. 1976;**70**(3):439-44.

17. Yoshida EM. The Crohn's Disease Activity Index, its derivatives and the Inflammatory Bowel Disease Questionnaire: a review of instruments to assess Crohn's disease. *Canadian journal of gastroenterology = Journal canadien de gastroenterologie*. 1999;**13**(1):65-73.

18. Sandborn WJ, Feagan BG, Hanauer SB, Lochs H, Lofberg R, Modigliani R*, et al.* A review of activity indices and efficacy endpoints for clinical trials of medical therapy in adults with Crohn's disease. *Gastroenterology*. 2002;**122**(2):512-30.

19. Hyams J, Crandall W, Kugathasan S, Griffiths A, Olson A, Johanns J*, et al.* Induction and maintenance infliximab therapy for the treatment of moderate-to-severe Crohn's disease in children. *Gastroenterology*. 2007;**132**(3):863-73; quiz 1165-6.

20. Best WR. Predicting the Crohn's disease activity index from the Harvey-Bradshaw Index. *Inflammatory bowel diseases*. 2006;**12**(4):304-10.

21. Irvine EJ. Usual therapy improves perianal Crohn's disease as measured by a new disease activity index. McMaster IBD Study Group. *Journal of clinical gastroenterology*. 1995;**20**(1):27-32.

22. Irvine EJ, Feagan B, Rochon J, Archambault A, Fedorak RN, Groll A*, et al.* Quality of life: a valid and reliable measure of therapeutic efficacy in the treatment of inflammatory bowel disease. Canadian Crohn's Relapse Prevention Trial Study Group. *Gastroenterology*. 1994;**106**(2):287-96.

23. Mary JY, Modigliani R. Development and validation of an endoscopic index of the severity for Crohn's disease: a prospective multicentre study. Groupe d'Etudes Therapeutiques des Affections Inflammatoires du Tube Digestif (GETAID). *Gut*. 1989;**30**(7):983-9.

24. Schnitzler F, Fidder H, Ferrante M, Noman M, Arijs I, Van Assche G*, et al.* Long-term outcome of treatment with infliximab in 614 patients with Crohn's disease: results from a single-centre cohort. *Gut*. 2009;**58**(4):492-500.

25. Gisbert JP, Panes J. Loss of response and requirement of infliximab dose intensification in Crohn's disease: a review. *The American journal of gastroenterology*. 2009;**104**(3):760-7.

26. Baert F, Noman M, Vermeire S, Van Assche G, G DH, Carbonez A*, et al.* Influence of immunogenicity on the long-term efficacy of infliximab in Crohn's disease. *The New England journal of medicine*. 2003;**348**(7):601-8.

27. Hanauer SB, Feagan BG, Lichtenstein GR, Mayer LF, Schreiber S, Colombel JF*, et al.* Maintenance infliximab for Crohn's disease: the ACCENT I randomised trial. *Lancet*. 2002;**359**(9317):1541-9.

28. Maser EA, Villela R, Silverberg MS, Greenberg GR. Association of trough serum infliximab to clinical outcome after scheduled maintenance treatment for Crohn's disease. *Clinical*

For peer review only - http://bmjopen.bmj.com/site/about/guidelines.xhtml

gastroenterology and hepatology : the official clinical practice journal of the American Gastroenterological Association. 2006;**4**(10):1248-54.

29. Hanauer SB, Wagner CL, Bala M, Mayer L, Travers S, Diamond RH*, et al.* Incidence and importance of antibody responses to infliximab after maintenance or episodic treatment in Crohn's disease*. Clinical gastroenterology and hepatology : the official clinical practice journal of the American Gastroenterological Association*. 2004;**2**(7):542-53.

30. Lichtenstein GR, Hanauer SB, Sandborn WJ. Management of Crohn's disease in adults*. The American journal of gastroenterology*. 2009;**104**(2):465-83; quiz 4, 84.

31. Dignass A, Van Assche G, Lindsay JO, Lemann M, Soderholm J, Colombel JF*, et al.* The second European evidence-based Consensus on the diagnosis and management of Crohn's disease: Current management*. Journal of Crohn's & colitis*. 2010;**4**(1):28-62.

32. National Institute for Health and Care Excellence. Crohn's disease: Management in adults, children and young people. CG152. 2012 [cited 06/10/2014]; Available from: https://www.nice.org.uk/guidance/cg152.

33. BMJ Best Practice. Crohn's disease. 2014 [cited 06/10/2014]; Available from: http://bestpractice.bmj.com/best-practice/monograph/42/treatment/details.html.

34. British Medical Assocation and Royal Pharmaceutical Society of Great Britain. British National Formulary and British National Formulary for Children. [cited 06/10/2014]; Available from: http://www.bnf.org/bnf/index.htm.

35. National Institute for Health and Care Excellence. Infliximab (review) and adalimumab for the treatment of Crohn's disease. TA187. 2010 [cited 06/10/2014]; Available from: http://www.nice.org.uk/guidance/TA187.

36. Vande Casteele N, Gils A, Ballet V, Compernolle G, Peeters M, Van Steen K*, et al.* Randomised Controlled Trial of Drug Level Versus Clinically Based Dosing of Infliximab Maintenance Therapy in IBD: Final Results of the TAXIT Study (OP001)*. United European Gastroenterology Journal*. 2013;**1**(1s):A1.

37. Scott FI, Lichtenstein GR. Therapeutic Drug Monitoring of Anti-TNF Therapy in Inflammatory Bowel Disease*. Curr Treat Options Gastroenterol*. 2014;**12**(1):59-75.

38. NHS Centre for Reviews and Dissemination. Undertaking systematic reviews of research on effectiveness: CRD guidelines for those carrying out or commissioning reviews. CRD Report 4. 1999.

39. National Institute for Health and Care Excellence. Diagnostics Assessment Programme manual. London, UK: National Institute for Health and Care Excellence; 2011 [cited 16/10/2014]. Available from: http://www.nice.org.uk/media/A0B/97/DAPManualFINAL.pdf.

40. Vande Casteele N, Buurman DJ, Sturkenboom MG, Kleibeuker JH, Vermeire S, Rispens T*, et al.* Detection of infliximab levels and anti-infliximab antibodies: a comparison of three different assays*. Alimentary pharmacology & therapeutics*. 2012;**36**(8):765-71.

For peer review only - http://bmjopen.bmj.com/site/about/guidelines.xhtml

41. Moher D, Liberati A, Tetzlaff J, Altman DG, The PRISMA Group. Preferred Reporting Items for Systematic Reviews and Meta-Analyses: The PRISMA Statement. *BMJ*. 2009;**339**:b2535.

42. Whiting PF, Rutjes AWS, Westwood ME, Mallett S, Deeks JJ, Reitsma JB*, et al.* QUADAS-2: A Revised Tool for the Quality Assessment of Diagnostic Accuracy Studies. *Annals of Internal Medicine*. 2011;**155**(8):529-36.

43. Downs SH, Black N. The feasibility of creating a checklist for the assessment of the methodological quality both of randomised and non-randomised studies of health care interventions. *Journal of epidemiology and community health*. 1998;**52**(6):377-84.

44. Higgins JP, Altman DG, Gotzsche PC, Juni P, Moher D, Oxman AD*, et al.* The Cochrane Collaboration's tool for assessing risk of bias in randomised trials. *Bmj*. 2011;**343**:d5928.

45. Merlin T, Lehman S, Hiller J, Ryan P. The "linked evidence approach" to assess medical tests: A critical analysis. *International Journal of Technology Assessment in Health Care*. 2013;**29**(03):343-50.

46. Husereau D, Drummond M, Petrou S, Carswell C, Moher D, Greenberg D*, et al.* Consolidated Health Economic Evaluation Reporting Standards (CHEERS) statement. *Int J Technol Assess Health Care*. 2013;**29**(2):117-22.

47. Philips Z, Ginnelly L, Sculpher M, Claxton K, Golder S, Riemsma R*, et al.* Review of guidelines for good practice in decision-analytic modelling in health technology assessment. *Health Technology Assessment*. 2004;**8**(36):1-158.

48. Dretzke J, Edlin R, Round J, Connock M, Hulme C, Czeczot J*, et al.* A systematic review and economic evaluation of the use of tumour necrosis factor-alpha (TNF-α) inhibitors, adalimumab and infliximab, for Crohn's disease. *Health Technology Assessment*. 2011;**15**(6):1-244.

49. Velayos FS, Kahn JG, Sandborn WJ, Feagan BG. A test-based strategy is more cost effective than empiric dose escalation for patients with Crohn's disease who lose responsiveness to infliximab. *Clinical Gastroenterology and Hepatology*. 2013;**11**(6):654-66.

50. Department of Health. NHS reference costs 2012 to 2013. 2013 [cited 18/03/2014]; Available from: https://www.gov.uk/government/publications/nhs-reference-costs-2012-to-2013.

51. Curtis L. Unit Costs of Health and Social Care 2013 [cited 18/03/2014]; Available from: http://www.pssru.ac.uk/project-pages/unit-costs/2013/.

52. European Medicines Agency. Remicade : EPAR - Product Information : Annex I - Summary of product characteristics. 2014 [cited 06/10/2014]; Available from: http://www.ema.europa.eu/docs/en_GB/document_library/EPAR_-_Product_Information/human/000240/WC500050888.pdf.

53. European Medicines Agency. Humira : EPAR - Product Information : Annex I - Summary of product characteristics. 2014 [cited 06/10/2014]; Available from: http://www.emea.eu.int/humandocs/PDFs/EPAR/Humira/H-481-PI-en.pdf.

For peer review only - http://bmjopen.bmj.com/site/about/guidelines.xhtml

Appendix 1. Licenced indications for Infliximab and Adalimumab in Crohn's disease

The licence indication for Crohn's disease detailed in the European Medicines Agency Summary of Product Characteristics (Remicade)[52] is as follows:

"Adult Crohn's disease: Remicade is indicated for:

- treatment of moderately to severely active Crohn's disease, in adult patients who have not responded despite a full and adequate course of therapy with a corticosteroid and/or an immunosuppressant; or who are intolerant to or have medical contraindications for such therapies;
- treatment of fistulising, active Crohn's disease, in adult patients who have not responded despite a full and adequate course of therapy with conventional treatment (including antibiotics, drainage and immunosuppressive therapy).

Paediatric Crohn's disease

Remicade is indicated for treatment of severe, active Crohn's disease, in children and adolescents aged 6 to 17 years, who have not responded to conventional therapy including a corticosteroid, an immunomodulator and primary nutrition therapy; or who are intolerant to or have contraindications for such therapies. Remicade has been studied only in combination with conventional immunosuppressive therapy.

Moderately to severely active Crohn's disease

5 mg/kg given as an intravenous infusion followed by an additional 5 mg/kg infusion 2 weeks after the first infusion. If a patient does not respond after 2 doses, no additional treatment with infliximab should be given. Available data do not support further infliximab treatment, in patients not responding within 6 weeks of the initial infusion.

In responding patients, the alternative strategies for continued treatment are:

- Maintenance: Additional infusions of 5 mg/kg at 6 weeks after the initial dose, followed by infusions every 8 weeks or
- Re-administration: Infusion of 5 mg/kg if signs and symptoms of the disease recur

Fistulising, active Crohn's disease

5 mg/kg given as an intravenous infusion followed by additional 5 mg/kg infusions at 2 and 6 weeks after the first infusion. If a patient does not respond after 3 doses, no additional treatment with infliximab should be given.

For peer review only - http://bmjopen.bmj.com/site/about/guidelines.xhtml

In responding patients, the alternative strategies for continued treatment are:

- Maintenance: Additional infusions of 5 mg/kg every 8 weeks or
- Re-administration: Infusion of 5 mg/kg if signs and symptoms of the disease recur followed by infusions of 5 mg/kg every 8 weeks.

Although comparative data are lacking, limited data in patients who initially responded to 5 mg/kg but who lost response indicate that some patients may regain response with dose escalation. Continued therapy should be carefully reconsidered in patients who show no evidence of therapeutic benefit after dose adjustment.

In Crohn's disease, experience with re-administration if signs and symptoms of disease recur is limited and comparative data on the benefit/risk of the alternative strategies for continued treatment are lacking.

Crohn's disease (6 to 17 years)

5 mg/kg given as an intravenous infusion followed by additional 5 mg/kg infusion doses at 2 and 6 weeks after the first infusion, then every 8 weeks thereafter. Available data do not support further infliximab treatment in children and adolescents not responding within the first 10 weeks of treatment.

Some patients may require a shorter dosing interval to maintain clinical benefit, while for others a longer dosing interval may be sufficient. Patients who have had their dose interval shortened to less than 8 weeks may be at greater risk for adverse reactions. Continued therapy with a shortened interval should be carefully considered in those patients who show no evidence of additional therapeutic benefit after a change in dosing interval."

The Adalimumbab licence indication for Crohn's disease detailed in the European Medicines Agency Summary of Product Characteristics (Humira)[53] is as follows:

Paediatric Crohn's Disease

Humira is indicated for the treatment of severe active Crohn's disease in paediatric patients (from 6 years of age) who have had an inadequate response to conventional therapy including primary nutrition therapy, a corticosteroid, and an immunomodulator, or who are intolerant to or have contraindications for such therapies.

Paediatric Crohn's disease patients < 40 kg:

The recommended Humira induction dose regimen for paediatric subjects with severe Crohn's disease is 40 mg at Week 0 followed by 20 mg at Week 2. In case there is a need for a more rapid response to

For peer review only - http://bmjopen.bmj.com/site/about/guidelines.xhtml

therapy, the regimen 80 mg at Week 0 (dose can be administered as two injections in one day), 40 mg at Week 2 can be used, with the awareness that the risk for adverse events may be higher with use of the higher induction dose.

After induction treatment, the recommended dose is 20 mg every other week via subcutaneous injection. Some subjects who experience insufficient response may benefit from an increase in dosing frequency to 20 mg Humira every week.

Paediatric Crohn's disease patients ≥ 40 kg:
The recommended Humira induction dose regimen for paediatric subjects with severe Crohn's disease is 80 mg at Week 0 followed by 40 mg at Week 2. In case there is a need for a more rapid response to therapy, the regimen 160 mg at Week 0 (dose can be administered as four injections in one day or as two injections per day for two consecutive days), 80 mg at Week 2 can be used, with the awareness that the risk for adverse events may be higher with use of the higher induction dose.

After induction treatment, the recommended dose is 40 mg every other week via subcutaneous injection. Some subjects who experience insufficient response may benefit from an increase in dosing frequency to 40 mg Humira every week.

Continued therapy should be carefully considered in a subject not responding by Week 12. A 40 mg pen and a 40 mg prefilled syringe are also available for patients to administer a full 40 mg dose. There is no relevant use of Humira in children aged less than 6 years in this indication.

For peer review only

For peer review only - http://bmjopen.bmj.com/site/about/guidelines.xhtml

Appendix 2. The CDAI Calculation of Crohn's Disease Activity Index (adapted from Best et al., 1976)[16]

Variable	Description	Scoring	Multiplier
No. of liquid stools	Sum of 7 days		x 2
Abdominal pain	Sum of 7 days' ratings	0=none<br>1=mild<br>2=moderate<br>3=severe	x 5
General well-being	Sum of 7 days' ratings	0=generally well<br>1=slightly under par<br>2=poor<br>3=very poor<br>4=terrible	x 7
Extraintestinal complications	Number of complications listed	Arthritis/arthralgia, iritis/uveitis, erythema nodosum, pyoderma gangrenosum, aphtous stomatitis, anal fissure/fistula/abscess, fever >37.8 °C	x 20
Anti-diarrhoeal drugs	Use in the previous 7 days	0=no<br>1=yes	x 30
Abdominal mass		0= no<br>2=questionable<br>5=definite	x 10
Haematocrit	Expected-observed Hct	Men: 47-observed<br>Women: 42-observed	x 6
Body weight	Ideal/observed ratio	(1-(ideal/observed)) x 100	x 1 (NOT< -10)

For peer review only

For peer review only - http://bmjopen.bmj.com/site/about/guidelines.xhtml

Appendix 3. Draft search strategy

Ovid MEDLINE(R) 1946 to October Week 2 2014, searched on 22/10/2014

1	adalimumab.mp.	3597
2	ADA.tw.	7105
3	infliximab.mp.	8842
4	IFX.tw.	326
5	((anti-TNF* or antiTNF* or TNF*) adj2 inhibitor*).mp.	2577
6	anti* tumo?r* necrosis* factor*.mp.	3007
7	Tumor Necrosis Factor-alpha/ and Antibodies, Monoclonal/	7682
8	anti* drug* antibod*.tw.	186
9	ADAb.tw.	19
10	1 or 2 or 3 or 4 or 5 or 6 or 7 or 8 or 9	24181
11	lisa* tracker*.mp.	1
12	(immundiagnostik* or immunodiagnostik* or immunediagnostik*).mp.	159
13	(proteomika* or promonitor*).mp.	13
14	exp Enzyme-Linked Immunosorbent Assay/	129174
15	enzyme* link* immunoassay*.mp.	2873
16	enzyme* link* immuno* assay*.mp.	158537
17	ELISA*.mp.	113426
18	11 or 12 or 13 or 14 or 15 or 16 or 17	205224
19	*Radioimmunoassay/	7091
20	(radioimmuno* or radio immuno* or radio-immuno*).mp.	101819
21	RIA.tw.	17353
22	reporter* gene* assay*.mp.	3663
23	RGA.tw.	336
24	semi* fluid* phase* enzyme* immuno*.mp.	0
25	EIA.tw.	8288
26	((homogenous* or homogeneous*) adj1 mobilit* shift* assay*).mp.	4
27	HMSA.tw.	62
28	(Biomonitor* or iLite).tw.	4102
29	(Matriks* Biotek* or Shikari*).mp.	2
30	(Prometheus* or Anser IFX or Anser ADA).mp.	258
31	19 or 20 or 21 or 22 or 23 or 24 or 25 or 26 or 27 or 28 or 29 or 30	124775
32	((monitor* or pharmacokinetic* or measur* or level* or concentration*) adj3 (adalimumab or ADA or infliximab or IFX or Anti-TNF* or Anti-Tumour Necrosis Factor*)).mp.	1087

For peer review only - http://bmjopen.bmj.com/site/about/guidelines.xhtml

33	Inflammatory Bowel Diseases/	14444
34	Crohn Disease/	31596
35	crohn*.tw.	32370
36	inflammator* bowel* disease*.tw.	26840
37	IBD.tw.	11936
38	33 or 34 or 35 or 36 or 37	58401
39	(((monitor* or pharmacokinetic* or measur* or level* or concentration*) adj3 (adalimumab or infliximab or Anti-TNF* or AntiTNF* or Anti-Tumour Necrosis Factor*)) and (correlat* or associat* or test performance)).mp.	218
40	10 and 18 and 38	93
41	10 and 31 and 38	19
42	32 and 38	157
43	39 or 40 or 41 or 42	367
44	Animals/ not Humans/	3983380
45	43 not 44	349

For peer review only - http://bmjopen.bmj.com/site/about/guidelines.xhtml

Appendix 4. Data extraction form for clinical effectiveness studies

Data extraction form anti-TNF drug monitoring

Name of first reviewer: *Name of second reviewer:*

**Study details**			
Study ID (Endnote ref)			
First author surname			
Year of publication			
Country			
Study design			
Publication (full/abstract)			
Study setting			
Number of centres (by arm)			
Duration of study			
Follow up period			
Funding			
**Aim of the study**			
**Inclusion/exclusion criteria for patients**			
Inclusion criteria:			
Exclusion criteria:			
**Study flow (consort diagram)**			
**Item**	**Anti-TNF monitoring arm**	**Clinical judgement arm**	**All**
N of Screened			
N of excluded (ineligible)			
N of enrolled/included (eligible)			
N of non-participants at study entry (those refused, etc…)			
N Study sample at baseline randomised (if applicable)			
Withdrawals			
Lost to follow up/drop outs (sample attrition)			
**Participants (characteristics and numbers)**			
**Item**	**Anti-TNF monitoring arm N (%)**	**Clinical judgement arm N (%)**	**All**
Total number of participants at baseline (% CD)			
N (%) followed up			
N (%) included in analysis			
Patient group (responders / secondary loss of response)			
Age Mean (SD/range)<br>Median (range) years			
Sex Women n (%)			
Diagnostic criteria for CD			
Children n (%)			
Crohn's Disease Activity Score (CDAI) Mean (SD)			
N (%) patients in remission			

For peer review only - http://bmjopen.bmj.com/site/about/guidelines.xhtml

N (%) patients with active CD			
CD classification (Vienna / Montreal)			
Disease duration (years)			
Smoking n (%)			
Previous surgery n (%)			
Concomitant treatment (specify) n (%)			
Treatment duration at anti-TNF failure (days)			
Line of therapy<br>1st<br>2nd<br>3rd			
Previous anti-TNF therapy n (%)			
CRP (mg/mL)			
Calprotectin (µg/g)			

**Treatment**		
**Item**	**Anti-TNF monitoring arm**	**Clinical judgement arm**
Anti-TNF drug (name)		
Anti-TNF dose		
Duration of treatment		

**Intervention test assay (please specify):**<br>**Technical aspects of test assay:**	
Manufacturer	
Time of anti-TNF, antibody measurement	
Assay type	
Assay name	
Type of ELISA (bridging / capture)	
Anti-TNF alpha detection:	
Micro plate pre-coat	
Drug detection (free / total)	
Detection reagents (one-step / two-step)	
Assay range	
Limit of detection	
Reagents	
Antibody reagent specificity for antigen	
Structural class of immunoglobulin of antibody	
Anti-body detection:	
Micro plate pre-coat	
Anti-body detection (free / total)	
Incubation times	
Assay range	
Limit of detection	
Standards/calibrators	

**Outcomes reported**			
**Item**	**Anti-TNF**	**Clinical**	**All**

For peer review only - http://bmjopen.bmj.com/site/about/guidelines.xhtml

	monitoring arm	judgement arm	
Primary outcome(s)			
Secondary study outcomes			
Timing of assessments (including info on parallel or sequential)			
Time to test result			
Number of inconclusive results n (%)			
Frequency of dose adjustment n (%)			
Frequency of treatment switch n (%)			
Measure of disease activity (e.g., CDAI, others?)			
Rates of<br>a) response y/n<br>b) relapse y/n<br>c) remission y/n			
Describe definition of progression:			
Describe definition of remission:			
Duration of<br>a) response<br>b) relapse<br>c) remission			
Rates of hospitalisation n (%)			
Rates of surgical intervention n (%)			
Time to surgical intervention y/n			
Health related quality of life y/n			
Length of follow up reported y/n			
Proportion progressing to surgery n (%)			
Time to surgical intervention			
**Incidence of adverse effects of treatment:**			
**Item**	**Anti-TNF monitoring arm**	**Clinical judgement arm**	**P value**

**Dose monitoring**		
**Item (Please define if necessary)**	**Anti-TNF monitoring arm**	**Clinical judgement arm**
Time of anti-TNF/ antibody measurement		
Frequency of anti-TNF/ antibody measurement		
Assay type		
Assay name		
Threshold of infliximab / adalimumab (therapeutic / sub-therapeutic) (in µg/mL)		
Limit of quantification of anti-TNF antibodies (in U/mL [arbitrary unit/mL]) for Ab		

detectable / non-detectable		
Algorithm specified for management y/n (specify)		
Algorithm provided		
Number of patients outside therapeutic range		
Mean anti-TNF (mg/m³/wk) (SD)		
Number of patients dose increased		
Number of patients dose reduced		
Other		
**Health related quality of life**		
**Item**	**Anti-TNF monitoring arm**	**Clinical judgement arm**
**Test comparison**		
**Tests**		
Intervention test		
Comparison test 1 (specify)		
Comparison test 2 (specify)		
Comparison test 3 (specify)		
Comparison test 1: test specifications (if ELISA use items for intervention assay test above)		
Comparison test 2: test specifications (if ELISA use items for intervention assay test above)		
Comparison test 3: test specifications (if ELISA use items for intervention assay test above)		
Details of any repeat measurements (to check reliability, performance across different laboratories)		
**Selection and storage of patients/plasma samples**		
Description of method of selection		
Description of method and duration of storage		
Number of clinical samples		
Number of calibrator samples (spiked) for anti-TNF		
Number of calibrator samples (spiked) for antibodies		
Number of blank (control) samples		
Total number of plasma samples		

For peer review only - http://bmjopen.bmj.com/site/about/guidelines.xhtml

Results of comparison			
Item	Intervention test vs test comparison 1	Intervention test vs test comparison 2	Intervention test vs test comparison 3
**Correlation of drug measurement:**			
Regression method			
Linearity test/cusum test?			
R^2 (95%CI)			
Slope (95%CI)			
Intercept (95%CI)			
**From Bland-Altman plot for drug measurement:**			
Percent bias (95%CI)			
Upper limit of agreement			
Lower limit of agreement			
Details of outliers			
Visually is there a pattern between the mean value and the difference? (If no pattern are statistics from Bland-Altman plot interpretable)			
N (%) samples outside limits of quantification, if yes specify decision for them			
N (%) false positives			
N (%) false negatives			
**Correlation of antibody measurement:**			
Regression method			
Linearity test/cusum test?			
R^2 (95%CI)			
Slope (95%CI)			
Intercept (95%CI)			
**From Bland-Altman plot for antibody measurement:**			
Percent bias (95%CI)			
Upper limit of agreement			
Lower limit of agreement			
Details of outliers			
Visually is there a pattern between the mean value and the difference? (If no pattern are statistics from Bland-Altman plot interpretable)			
N (%) samples outside limits of quantification, if yes specify decision for them			
N (%) false positives			
N (%) false negatives			
**Authors' conclusion**			
**Reviewer's conclusion**			

For peer review only - http://bmjopen.bmj.com/site/about/guidelines.xhtml

Appendix 5. Quality assessment forms

A – QUADAS-2[42] tool with index questions adapted to the review for studies comparing performance of different tests

Name of first reviewer: Name of second reviewer:

Phase 1: State the review question

Patients (setting, intended use of index test, presentation, prior testing):
Index test(s):
Reference standard:

Phase 2: Draw a flow diagram for the primary study

Phase 3: Risk of bias and applicability judgements

QUADAS-2 is structured so that four key domains are each rated in terms of the risk of bias and the concern regarding applicability to the review question (as stated in Phase 1). Each key domain has a set of signalling questions to help reach the judgements regarding bias and applicability.

Domain 1: Patient selection

**A. Risk of bias**	
Describe methods of patient selection:	
**Was a consecutive or random sample of patients enrolled?**	
**Did the study avoid inappropriate exclusions?**	
**Could the selection of patients have introduced bias?**<br><br>**Risk:**	
**B. Concerns regarding applicability**	
Describe included patients (prior testing, presentation, intended use of intervention test and setting):<br><br>Range of drug / antibody concentrations:	
**Is there concern that the included patients or range of drug / antibody concentrations do not match the review question?**<br><br>**Concern:**	

Domain 2: Index test(s)

**A. Risk of bias**	
Describe the intervention test and how it was conducted and interpreted:	
Were the number of failed results and measurement repeats reported?	
**Could the conduct or interpretation of the intervention test have introduced bias?**<br><br>**Risk:**	

For peer review only - http://bmjopen.bmj.com/site/about/guidelines.xhtml

**B. Concerns regarding applicability**<br><br>Describe the preparation and storage of the sample before the intervention test was applied:
**Is there concern that the intervention test, its conduct, or interpretation differ from the review question?**<br><br>**Concern:**

Domain 3: Reference standard (Comparison test)

**A. Risk of bias**	
Describe the comparison test and how it was conducted and interpreted:	
**Is the comparison test likely to correctly classify the target condition?**	
**Could the comparison test, its conduct, or its interpretation have introduced bias?**<br><br>**Risk:**	
**B. Concerns regarding applicability**	
**Is there concern that the target condition as defined by the comparison test does not match the review question?**<br><br>**Concern:**	

Domain 4: Flow and timing

**A. Risk of bias**	
Describe any patients who did not receive the intervention test and/or comparison test(s) or who were excluded from the Bland-Altman plot:<br><br>Describe the time interval and any interventions between intervention test and comparison test(s):	
**Was there an appropriate interval between intervention test and comparison test(s)?**	
**Were both intervention test and reference standard conducted on all samples?**	
**Did patients receive the same comparison test(s)?**	
**Were all patients included in the Bland-Altman plot?**	
**Could the patient flow have introduced bias?**<br><br>**Risk:**	

For peer review only - http://bmjopen.bmj.com/site/about/guidelines.xhtml

B – Cochrane Collaboration's tool for assessing risk of bias for a randomised controlled trial (adapted from Higgins et al., 2011[44])

First author surname and year of publication:
Name of first reviewer: ***Name of second reviewer:***

**Domain**	**Description**	**Review authors' judgement**
**Sequence generation**	Describe the method used to generate the allocation sequence in sufficient detail to allow an assessment of whether it should produce comparable groups	Was the allocation sequence adequately generated?
**Allocation concealment**	Describe the method used to conceal the allocation sequence in sufficient detail to determine whether intervention allocations could have been foreseen in advance of, or during, enrolment	Was allocation adequately concealed?
**Blinding of participants, personnel and outcome assessors** *Assessments should be made for each main outcome (or class of outcomes)*	Describe all measures used, if any, to blind study participants and personnel from knowledge of which intervention a participant received. Provide any information relating to whether the intended blinding was effective	Was knowledge of the allocated intervention adequately prevented during the study?
**Incomplete outcome data** *Assessments should be made for each main outcome (or class of outcomes)*	Describe the completeness of outcome data for each main outcome, including attrition and exclusions from the analysis. State whether attrition and exclusions were reported, the numbers in each intervention group (compared with total randomized participants), reasons for attrition/exclusions where reported, and any re-inclusions in analyses performed by the review authors	Were incomplete outcome data adequately addressed?
**Selective outcome reporting**	State how the possibility of selective outcome reporting was examined by the review authors, and what was found	Are reports of the study free of suggestion of selective outcome reporting?
**Other sources of bias**	State any important concerns about bias not addressed in the other domains in the tool. If particular questions/entries were pre-specified in the review's protocol, responses should be provided for each question/entry	Was the study apparently free of other problems that could put it at a high risk of bias?

For peer review only - http://bmjopen.bmj.com/site/about/guidelines.xhtml

Summary assessment of the risk of bias across domains (please highlight overall risk of bias rating)

**Risk of bias across key domains**	**Interpretation**	**Summary risk of bias**
Low risk of bias for all key domains	Plausible bias unlikely to seriously alter the results	Low risk of bias
Unclear risk of bias for one or more key domains	Plausible bias that raises some doubt about the results	Unclear risk of bias
High risk of bias for one or more key domains	Plausible bias that seriously weakens confidence in the results	High risk of bias

For peer review only

For peer review only - http://bmjopen.bmj.com/site/about/guidelines.xhtml

C – Downs and Black checklist[43] for non-randomised primary clinical studies

First author (year) study ID:

Name of first reviewer: *Name of second reviewer:*

**Reporting**	**Rating**
1. Is the hypothesis/aim/objective of the study clearly described? **(Yes/No)**	
2. Are the main outcomes to be measured clearly described in the Introduction or Methods section? **(Yes/No)** *If the main outcomes are first mentioned in the Results section, the question should be answered "No"*	
3. Are the characteristics of the patients included in the study clearly described? **(Yes/No)** *In cohort studies and trials, inclusion and/or exclusion criteria should be given. In case-control studies, a case-definition and the source for controls should be givenFsan*	
4. Are the interventions of interest clearly described? **(Yes/No)** *Treatments and placebo (where relevant) that are to be compared should be clearly described*	
5. Are the distributions of principal confounders in each group of subjects to be compared clearly described? **(Yes/Partially/No)** *A list of principal confounders is provided*	
6. Are the main findings of the study clearly described? **(Yes/No)** *Simple outcome data (including denominators and numerators) should be reported for all major findings so that the reader can check the major analyses and conclusions (This question does not cover statistical tests which are considered below)*	
7. Does the study provide estimates of the random variability in the data for the main outcomes? **(Yes/No)** *In non-normally distributed data the inter-quartile range of results should be reported. In normally distributed data the standard error, standard deviation or confidence intervals should be reported. If the distribution of the data is not described, it must be assumed that the estimates used were appropriate and the question should be answered "Yes"*	
8. Have all important adverse events that may be a consequence of the intervention been reported? **(Yes/No)** *This should be answered "Yes" if the study demonstrates that there was a comprehensive attempt to measure adverse events. (A list of possible adverse events is provided)*	
9. Have the characteristics of patients lost to follow-up been described? **(Yes/No)** *This should be answered "Yes" where there were no losses to follow-up or where losses to follow-up were so small that findings would be unaffected by their inclusion. This should be answered "No" where a study does not report the number of patients lost to follow-up*	
10. Have actual probability values been reported (e.g., 0.035 rather than <0.05) for the main outcomes except where the probability value is less than 0.001? **(Yes/No)**	
**External validity**	**Rating**
11. Were the subjects asked to participate in the study representative of the entire population from which they were recruited? **(Yes/No/Unable to determine)** *The study must identify the source population for patients and describe how the patients were selected. Patients would be representative if they comprised the entire source population, an unselected sample of*	

For peer review only - http://bmjopen.bmj.com/site/about/guidelines.xhtml

consecutive patients, or a random sample. Random sampling is only feasible where a list of all members of the relevant	
12. Were those subjects who were prepared to participate representative of the entire population from which they were recruited? **(Yes/No/Unable to determine)** *The proportion of those asked who agreed should be stated. Validation that the sample was representative would include demonstrating that the distribution of the main confounding factors was the same in the study sample and the source population*	
13. Were the staff, places, and facilities where the patients were treated, representative of the treatment the majority of patients receive? **(Yes/No/Unable to determine)** *For the question to be answered "Yes" the study should demonstrate that the intervention was representative of that in use in the source population. The question should be answered "No" if, for example, the intervention was undertaken in a specialist centre unrepresentative of the hospitals most of the source population would attend*	
**Internal validity – bias**	**Rating**
14. Was an attempt made to blind study subjects to the intervention they have received? **(Yes/No/Unable to determine)** *For studies where the patients would have no way of knowing which intervention they received, this should be answered "Yes"*	
15. Was an attempt made to blind those measuring the main outcomes of the intervention? **(Yes/No/Unable to determine)**	
16. If any of the results of the study were based on "data dredging", was this made clear? **(Yes/No/Unable to determine)** *Any analyses that had not been planned at the outset of the study should be clearly indicated. If no retrospective unplanned subgroup analyses were reported, then answer "Yes"*	
17. In trials and cohort studies, do the analyses adjust for different lengths of follow-up of patients, or in case-control studies, is the time period between the intervention and outcome the same for cases and controls? **(Yes/No/Unable to determine)** *Where follow-up was the same for all study patients the answer should "Yes". If different lengths of follow-up were adjusted for by, for example, survival analysis the answer should be "Yes". Studies where differences in follow-up are ignored should be answered "No"*	
18. Were the statistical tests used to assess the main outcomes appropriate? **(Yes/No/Unable to determine)** *The statistical techniques used must be appropriate to the data. For example nonparametric methods should be used for small sample sizes. Where little statistical analysis has been undertaken but where there is no evidence of bias, the question should be answered "Yes". If the distribution of the data (normal or not) is not described it must be assumed that the estimates used were appropriate and the question should be answered "Yes"*	
19. Was compliance with the intervention/s reliable? **(Yes/No/Unable to determine)** *Where there was non-compliance with the allocated treatment or where there was contamination of one group, the question should be answered "No". For studies where the effect of any misclassification was likely to bias any association to the null, the question should be answered "Yes"*	

For peer review only - http://bmjopen.bmj.com/site/about/guidelines.xhtml

20. Were the main outcome measures used accurate valid and reliable? **(Yes/No/Unable to determine)** *For studies where the outcome measures are clearly described, the question should be answered "Yes". For studies which refer to other work or that demonstrates the outcome measures are accurate, the question should be answered as "Yes"*	
**Internal validity - confounding (selection bias)**	**Rating**
21. Were the patients in different intervention groups (trials and cohort studies) or were the cases and controls (case-control studies) recruited from the same population? **(Yes/No/Unable to determine)** *For example, patients for all comparison groups should be selected from the same hospital. The question should be answered "Unable to determine" for cohort and case-control studies where there is no information concerning the source of patients included in the study*	
22. Were study subjects in different intervention groups (trials and cohort studies) or were the cases and controls (case-control studies) recruited over the same period of time? **(Yes/No/Unable to determine)** *For a study which does not specify the time period over which patients were recruited, the question should be answered as "Unable to determine"*	
23. Were the subjects randomised to intervention groups? **(Yes/No/Unable to determine)** *Studies which state that subjects were randomised should be answered "Yes" except where method of randomisation would not ensure random allocation. For example alternate allocation would score "No" because it is predictable*	
24. Was the randomised intervention assignment concealed from both patients and health care staff until recruitment was complete and irrevocable? **(Yes/No/Unable to determine)** *All non-randomised studies should be answered "No". If assignment was concealed from patients but not from staff, it should be answered "No"*	
25. Was there adequate adjustment for confounding in the analyses from which the main findings were drawn? **(Yes/No/Unable to determine)** *This question should be answered "No" for trials if: the main conclusions of the study were based on analyses of treatment rather than intention to treat; the distribution of known confounders in the different treatment groups was not described; or the distribution of known confounders differed between the treatment groups but was not taken into account in the analyses. In nonrandomised studies if the effect of the main confounders was not investigated or confounding was demonstrated but no adjustment was made in the final analyses the question should be answered as "No"*	
26. Were losses of patients to follow-up taken into account? **(Yes/No/Unable to determine)** *If the numbers of patients lost to follow-up are not reported, the question should be answered as "Unable to determine". If the proportion lost to follow-up was too small to affect the main findings, the question should be answered "Yes"*	
**Power**	**Rating**
27. Did the study have sufficient power to detect a clinically important effect where the probability value for a difference being due to chance is less than 5%? **(Yes/No/Unable to determine)***	

For peer review only

For peer review only - http://bmjopen.bmj.com/site/about/guidelines.xhtml

D – Critical appraisal of the economic evaluation studies using the CHEERS checklist (adapted from Husereau et al, 2013[46])

Title and abstract				
1 Title: Identify the study as an economic evaluation, or use more specific terms such as ``cost-effectiveness analysis``, and describe the interventions compared.				
2 Abstract: Provide a structured summary of objectives, methods including study design and inputs, results including base case and uncertainty analyses, and conclusions.				
Introduction				
3 Background & objectives: Provide an explicit statement of the broader context for the study. Present the study question and its relevance for health policy or practice decisions.				
Methods				
4 Target Population and Subgroups: Describe characteristics of the base case population and subgroups analysed including why they were chosen.				
5 Setting and Location: State relevant aspects of the system(s) in which the decision(s) need(s) to be made.				
6 Study perspective: Describe the perspective of the study and relate this to the costs being evaluated.				
7 Comparators: Describe the interventions or strategies being compared and state why they were chosen.				
8 Time Horizon: State the time horizon(s) over which costs and consequences are being evaluated and say why appropriate.				
9 Discount Rate: Report the choice of discount rate(s) used for costs and outcomes and say why appropriate.				

For peer review only

For peer review only - http://bmjopen.bmj.com/site/about/guidelines.xhtml

10 Choice of Health Outcomes: Describe what outcomes were used as the measure(s) of benefit in the evaluation and their relevance for the type of analysis performed.				
11a Measurement of Effectiveness - Single Study-Based Estimates: Describe fully the design features of the single effectiveness study and why the single study was a sufficient source of clinical effectiveness data.				
11b Measurement of Effectiveness - Synthesis-based Estimates: Describe fully the methods used for identification of included studies and clinical effectiveness data synthesis of clinical effectiveness data.				
12 Measurement and Valuation of Preference-based Outcomes: If applicable, describe the population and methods used to elicit preferences for health outcomes.				
13a Estimating Resources and Costs - Single Study-based Economic evaluation: Describe approaches used to estimate resource use associated with the alternative interventions. Describe primary or secondary research methods for valuing each resource item in terms of its unit cost. Describe any adjustments made to approximate to opportunity costs.				
13b Estimating Resources and Costs - Model-based Economic Evaluation: Describe approaches and data sources used to estimate resource use associated with model health states. Describe primary or secondary research methods for valuing each resource item in terms of its unit cost. Describe any adjustments made to approximate to opportunity costs.				
14 Currency, Price Date and Conversion: Report the dates of the estimated resource quantities				

For peer review only - http://bmjopen.bmj.com/site/about/guidelines.xhtml

and unit costs. Describe methods for adjusting estimated unit costs to the year of reported costs if necessary. Describe methods for converting costs into a common currency base and the exchange rate.				
15 Choice of Model: Describe and give reasons for the specific type of decision-analytic model used. Providing a figure to show model structure is strongly recommended.				
16 Assumptions: Describe all structural or other assumptions underpinning the decision-analytic model.				
17 Analytic Methods: Describe all analytic methods supporting the evaluation. This could include methods for dealing with skewed, missing or censored data, extrapolation methods, methods for pooling data, approaches to validate a model, and methods for handling population heterogeneity and uncertainty.				
Results				
18 Study parameters: Report the values, ranges, references, and if used, probability distributions for all parameters. Report reasons or sources for distributions used to represent uncertainty where appropriate. We strongly recommend the use of a table to show the input values.				
19. Incremental costs and outcomes: For each intervention, report mean values for the main categories of estimated costs and outcomes of interest, as well as mean differences between the comparator groups. If applicable, report incremental cost-effectiveness ratios.				
20a Characterizing Uncertainty - Single study-based economic evaluation: Describe the effects of sampling uncertainty for the estimated incremental cost and incremental effectiveness,				

For peer review only - http://bmjopen.bmj.com/site/about/guidelines.xhtml

parameters together with the impact of methodological assumptions.				
20b Characterizing Uncertainty - Model-based economic evaluation: Describe the effects on the results of uncertainty for all input parameters, and uncertainty related to the structure of the model and assumptions.				
21 Characterizing Heterogeneity: If applicable, report differences in costs, outcomes or in cost-effectiveness that can be explained by variations between subgroups of patients with different baseline characteristics or other observed variability in effects that are not reducible by more information.				
Discussion				
22 Study Findings, Limitations, Generalizability, and Current Knowledge: Summarize key study findings and describe how they support the conclusions reached. Discuss limitations and the generalizability of the findings and how the findings fit with current knowledge.				
Other				
23 Source of Funding: Describe how the study was funded and the role of the funder in the identification, design, conduct and reporting of the analysis. Describe other non-monetary sources of support.				
24 Conflicts of Interest: Describe any potential for conflict of interest among study contributors in accordance with journal policy. In the absence of a journal policy, we recommend authors comply with International Committee of Medical Journal Editors' recommendations.				

*Key: Y = yes, No = no, N/A = not applicable and * = partially completed*

For peer review only

For peer review only - http://bmjopen.bmj.com/site/about/guidelines.xhtml

PRISMA 2009 Checklist

Section/topic	#	Checklist item	Reported on page #
**TITLE**			
Title	1	Identify the report as a systematic review, meta-analysis, or both.	1
**ABSTRACT**			
Structured summary	2	Provide a structured summary including, as applicable: background; objectives; data sources; study eligibility criteria, participants, and interventions; study appraisal and synthesis methods; results; limitations; conclusions and implications of key findings; systematic review registration number.	2
**INTRODUCTION**			
Rationale	3	Describe the rationale for the review in the context of what is already known.	4
Objectives	4	Provide an explicit statement of questions being addressed with reference to participants, interventions, comparisons, outcomes, and study design (PICOS).	5
**METHODS**			
Protocol and registration	5	Indicate if a review protocol exists, if and where it can be accessed (e.g., Web address), and, if available, provide registration information including registration number.	6
Eligibility criteria	6	Specify study characteristics (e.g., PICOS, length of follow-up) and report characteristics (e.g., years considered, language, publication status) used as criteria for eligibility, giving rationale.	5
Information sources	7	Describe all information sources (e.g., databases with dates of coverage, contact with study authors to identify additional studies) in the search and date last searched.	5
Search	8	Present full electronic search strategy for at least one database, including any limits used, such that it could be repeated.	Supplementary material
Study selection	9	State the process for selecting studies (i.e., screening, eligibility, included in systematic review, and, if applicable, included in the meta-analysis).	5
Data collection process	10	Describe method of data extraction from reports (e.g., piloted forms, independently, in duplicate) and any processes for obtaining and confirming data from investigators.	5
Data items	11	List and define all variables for which data were sought (e.g., PICOS, funding sources) and any assumptions and simplifications made.	Supplementary material
Risk of bias in individual studies	12	Describe methods used for assessing risk of bias of individual studies (including specification of whether this was done at the study or outcome level), and how this information is to be used in any data synthesis.	5
Summary measures	13	State the principal summary measures (e.g., risk ratio, difference in means).	6
Synthesis of results	14	Describe the methods of handling data and combining results of studies, if done, including measures of consistency (e.g., I^2) for each meta-analysis.	5,6

For peer review only

PRISMA 2009 Checklist

Section/topic	#	Checklist item	Reported on page #
Risk of bias across studies	15	Specify any assessment of risk of bias that may affect the cumulative evidence (e.g., publication bias, selective reporting within studies).	6
Additional analyses	16	Describe methods of additional analyses (e.g., sensitivity or subgroup analyses, meta-regression), if done, indicating which were pre-specified.	6
**RESULTS**			
Study selection	17	Give numbers of studies screened, assessed for eligibility, and included in the review, with reasons for exclusions at each stage, ideally with a flow diagram.	Fig1
Study characteristics	18	For each study, present characteristics for which data were extracted (e.g., study size, PICOS, follow-up period) and provide the citations.	Table 1 and supplementary material
Risk of bias within studies	19	Present data on risk of bias of each study and, if available, any outcome level assessment (see item 12).	Supplementary material
Results of individual studies	20	For all outcomes considered (benefits or harms), present, for each study: (a) simple summary data for each intervention group (b) effect estimates and confidence intervals, ideally with a forest plot.	Figures 2-6
Synthesis of results	21	Present results of each meta-analysis done, including confidence intervals and measures of consistency.	Fig 5-6
Risk of bias across studies	22	Present results of any assessment of risk of bias across studies (see Item 15).	Supplementary material
Additional analysis	23	Give results of additional analyses, if done (e.g., sensitivity or subgroup analyses, meta-regression [see Item 16]).	
**DISCUSSION**			
Summary of evidence	24	Summarize the main findings including the strength of evidence for each main outcome; consider their relevance to key groups (e.g., healthcare providers, users, and policy makers).	9
Limitations	25	Discuss limitations at study and outcome level (e.g., risk of bias), and at review-level (e.g., incomplete retrieval of identified research, reporting bias).	9
Conclusions	26	Provide a general interpretation of the results in the context of other evidence, and implications for future research.	9
**FUNDING**			
Funding	27	Describe sources of funding for the systematic review and other support (e.g., supply of data); role of funders for the systematic review.	online

For peer review only

PRISMA 2009 Checklist

From: Moher D, Liberati A, Tetzlaff J, Altman DG, The PRISMA Group (2009). Preferred Reporting Items for Systematic Reviews and Meta-Analyses: The PRISMA Statement. PLoS Med 6(6): e1000097. doi:10.1371/journal.pmed1000097

For more information, visit: **www.prisma-statement.org**.

For peer review only

For peer review only - http://bmjopen.bmj.com/site/about/guidelines.xhtml

BMJ Open

Test accuracy of drug and antibody assays for predicting response to anti-Tumour Necrosis Factor treatment in Crohn’s disease: a systematic review and meta-analysis

Journal:	*BMJ Open*
Manuscript ID	bmjopen-2016-014581.R1
Article Type:	Research
Date Submitted by the Author:	09-Feb-2017
Complete List of Authors:	Freeman, Karoline; University of Warwick Warwick Medical School<br>Taylor-Phillips, Sian; University of Warwick, Warwick Medical School<br>Connock, Martin; University of Warwick, Division of Health Sciences, Warwick Medical School<br>Court, Rachel; Warwick University, Division of Health Sciences<br>Tsertsvadze, Alexander; University of Warwick Warwick Medical School<br>Shyangdan, Deepson; University of Warwick Warwick Medical School<br>Auguste, Peter; University of Warwick Warwick Medical School<br>Mistry, Hema; University of Warwick, Warwick Evidence<br>Arasaradnam, Ramesh; University Hospitals Coventry and Warwickshire NHS Trust, Gastroenterology<br>Sutcliffe, Paul; University of Warwick, Division of Health Sciences, Warwick Medical School<br>Clarke, Aileen; University of Warwick, Division of Health Sciences
**Primary Subject Heading**:	Gastroenterology and hepatology
Secondary Subject Heading:	Diagnostics
Keywords:	Inflammatory bowel disease < GASTROENTEROLOGY, Gastroenterology < INTERNAL MEDICINE, Adult gastroenterology < GASTROENTEROLOGY, meta-analysis, Systematic review, Infliximab

SCHOLARONE™ Manuscripts

For peer review only - http://bmjopen.bmj.com/site/about/guidelines.xhtml

Test accuracy of drug and antibody assays for predicting response to anti-Tumour Necrosis Factor treatment in Crohn's disease: a systematic review and meta-analysis

Karoline Freeman[1] K.Freeman@warwick.ac.uk
Sian Taylor-Phillips[1] S.Taylor-Phillips@warwick.ac.uk
Martin Connock[1] M.Connock@warwick.ac.uk>
Rachel Court[1] R.A.Court@warwick.ac.uk
Alexander Tsertsvadze[1] A.Tsertsvadze.1@warwick.ac.uk
Deepson Shyangdan[1] deepsonshyangdan@hotmail.com
Peter Auguste[1] P.Auguste@warwick.ac.uk
Hema Mistry[1] Hema.Mistry@warwick.ac.uk
Ramesh Arasaradnam[1,2] R.Arasaradnam@warwick.ac.uk
Paul Sutcliffe[1] P.A.Sutcliffe@warwick.ac.uk
Aileen Clarke[1] Aileen.Clarke@warwick.ac.uk

1. Warwick Medical School, University of Warwick, Coventry, UK
2. Gastroenterology, University Hospital Coventry and Warwickshire, Coventry, UK

Corresponding author: Sian Taylor-Phillips
Address: Warwick Medical School, The University of Warwick, Coventry, CV4 7AL
e-mail: s.taylor-phillips@warwick.ac.uk
Telephone: +44(0)7725000262

Keywords: Crohn disease, anti-TNF, meta-analysis, predictive value, sensitivity, specificity

Word count:3034

For peer review only

ABSTRACT

Objective: To present meta-analytic test accuracy estimates of levels of anti-TNF and antibodies to anti-TNF to predict loss of response or lack of regaining response in anti-TNF managed Crohn's disease patients.

Methods: MEDLINE, Embase, the Cochrane Library and Science Citation Index were searched from inception to October / November 2014 to identify studies which reported 2x2 table data of the association between levels of anti-TNF or its antibodies and clinical status. Hierarchical / bivariate meta-analysis was undertaken with the user-written "metandi" package of Harbord and Whiting using Stata 11 software, for Infliximab, Adalimumab, anti-Infliximab and anti-Adalimumab levels as predictors of loss of response. Prevalence of Crohn's disease in included studies was meta-analysed using a random effects model in MetaAnalyst software to calculate positive and negative predictive values. The search was updated in January 2017.

Results: 31 studies were included in the review. Studies were heterogeneous with respect to type of test used, criteria for establishing response and loss of response, population examined, and results. Meta-analytic summary point estimates for sensitivity and specificity were 65.7% and 80.6% for Infliximab trough levels and 56% and 79% for antibodies to Infliximab, respectively. Pooled results for Adalimumab trough levels and antibodies to Adalimumab were similar. Pooled positive and negative predictive values ranged between 70% and 80% implying that between 20% and 30% of both positive and negative test results may be incorrect in predicting loss of response.

Conclusion: The available evidence suggests that these tests have modest predictive accuracy for clinical status, direct test accuracy comparisons in the same population are needed. More clinical trial evidence from test-treat studies is required before the clinical utility of the tests can be reliably evaluated.

Strengths and Limitations of this study

- This is the first study to summarise predictive accuracy of tests for loss of response to anti-TNF drugs for managing Crohn's disease, in a clinically relevant manner
- We included more studies than previous meta-analyses
- We investigated drug and antibody levels for both Infliximab and Adalimumab
- Many of the included studies had a high risk of bias
- There was insufficient data for sub-group analyses for some types of test

For peer review only - http://bmjopen.bmj.com/site/about/guidelines.xhtml

INTRODUCTION

Anti-Tumour Necrosis Factor (anti-TNFα) agents, including Infliximab [Remicade®, Merck Sharp & Dohme Ltd.] and Adalimumab [Humira®, AbbVie], are well-established second or third line therapies for people with Crohn's disease (CD). Failure to respond during induction therapy, and loss of response after initial success, are widely documented.[1-5] One suggested mechanism for this is the production of antibodies which neutralise the anti-TNFα agents and hasten their clearance from the circulation, thus reducing drug availability. The treatment strategy for loss of response is usually to escalate the drug dosage or to shorten the dosage interval. If this fails, a switch to an alternative anti-TNF agent can be tried in order to minimise the influence of anti-drug antibodies directed against the first agent. Another suggested underlying mechanism for loss of response is that cytokines other than TNFα may become the major inflammatory agents. This suggestion arises from the observation that some patients have a loss of response to anti-TNF despite the presence of therapeutic drug levels and an absence of anti-TNF antibodies. For such patients the continued use of anti-TNFs may be considered futile and a switch to different biological therapies or other agents may represent the preferred strategy.

The potential role of anti-TNF antibodies and of sub-therapeutic drug levels in loss of response has provided the impetus for the development of assays for both anti-TNF drugs and for antibodies and a plethora of studies using such assays have been produced, exploring the association between either levels of antibodies to anti-TNF agents and clinical response or levels of drugs and clinical response. Studies have measured loss of response to the administered anti-TNF agent or failure to regain response after a change in treatment. By dichotomising the outcomes at various detectable levels of drug and of antibodies to anti-TNF, the diagnostic value of these tests in predicting loss of response or lack of regaining response has been assessed.

Several authors have meta-analysed studies which have reported the association between levels of antibodies to anti-TNF agents and clinical status.[6-9] These authors have presented pooled relative risk or odds ratio statistics for clinical state (e.g. response or loss of response) investigating positive versus negative test result patients (i.e. antibodies to anti-TNF agent present or absent), or conversely for test result (positive or negative) in patients with response versus those without response. Although these pooled statistics provide useful information on the association between antibody levels and clinical status, they do not address the question of test accuracy when tests are used as a predictor of patients' clinical response status which is the perspective likely to be adopted by clinicians for patients receiving treatment that may be predicated on test results. Primary studies frequently report test accuracy analysis such as receiver operating characteristic curves and test accuracy measures such as sensitivity and specificity. When viewed as diagnostic tests[10] it becomes possible to perform alternative meta-analysis so as to obtain pooled estimates of test accuracy. The predictive accuracy of such tests is of considerable practical interest. Our objective therefore is to present the meta-analytic results in terms of pooled test accuracy estimates. A particular advantage of this method is that it allows for investigation

For peer review only

of the co-variance of associations or, from the perspective of a predictive test, the covariance between sensitivity and specificity, thus giving a more complete picture of the value of these tests in clinical practice.

METHODS

Search for studies

An iterative procedure was used to develop the initial MEDLINE search, which was subsequently adapted appropriately for other databases and online resources. We searched multiple bibliographic databases including MEDLINE, Embase, the Cochrane Library and Science Citation Index from inception to October / November 2014. Searches of other online resources including trial registries were also undertaken. Full details of the search strategies used, with exact search dates, are provided in Supplement 1. Reference lists of included studies and relevant review articles were checked. Citation searches of selected included studies were undertaken. An update of the search was undertaken in January 2017 (Supplement 2 Figure 1 and Supplement 2 Table 1).

Study eligibility criteria

We included studies of patients with Crohn's disease treated with Infliximab or Adalimumab. Studies with mixed Crohn's and ulcerative colitis (UC) populations were included if the proportion of Crohn's patients was at least 70%. The intervention of interest was a test measuring serum anti-TNFα (Infliximab or Adalimumab) and / or anti-Infliximab or anti-Adalimumab antibody levels. Studies reporting clinical status (i.e., response or lack of response) as an outcome were eligible for inclusion. The reported results had to allow for cross-tabulation of dichotomous test outcome with clinical status by means of two-by-two tables in order to calculate the diagnostic test accuracy parameters. All primary study designs were included.

Study selection

Two reviewers independently assessed titles and abstracts for inclusion using a pre-piloted form. All potentially relevant publications were retrieved and examined independently. Any disagreements regarding inclusion/exclusion were discussed and resolved with a third reviewer. The study selection process and reasons for exclusion at full text screening level are presented in the PRISMA study flow diagram (see Figure 1).

Quality assessment

Studies were quality assessed using a modified QUADAS-2 checklist.[11] Items included were method of patient selection, blinding of index test results, exclusion of uninterpretable test results from 2x2 table data and method of assessment of clinical status (the reference case).

Evidence synthesis and statistical methods

For peer review only

Patient numbers within extracted two by two data tables were used to generate Forest plots of paired sensitivity and specificity (accompanied by 95% CIs) using Review Manager (RevMan 5.1; Nordic Cochrane Centre, Copenhagen, Denmark) for four different tests: (1) Infliximab levels as predictor of loss of or lack of regaining response, (2) antibodies to Infliximab as predictor of loss of or lack of regaining response, (3) Adalimumab levels as predictor of loss of or lack of regaining response, and (4) antibodies to Adalimumab as predictor of loss of or lack of regaining response. Hierarchical / bivariate[12] meta-analysis was undertaken with the user-written “metandi” package of Harbord and Whiting[13] using Stata 11 software. Positive and negative predictive values were calculated[14] at the pooled prevalence of loss of response in the test population. Prevalence was meta-analysed using a random effects model in MetaAnalyst software.[15] For meta-analyses which incorporated 10 or more studies we examined the risk of publication bias (Supplement 3) mindful of the caveats relating to this in diagnostic test accuracy studies.[16]

The protocol for this review was registered on PROSPERO 2014:CRD42014015278. The full protocol is included in appendix 1.

RESULTS

We identified 2429 records of which 31 were eligible for inclusion (see Supplement 4 Table 1 and Supplement 4 Table 2 for excluded studies with reason). Of these 24 were full-text reports and 7 were conference abstracts. The PRISMA flow diagram is detailed in Figure 1. Eleven of the 31 studies examined Infliximab trough levels, 20 examined levels of antibodies to Infliximab and five and six studies respectively investigated Adalimumab levels and antibodies to Adalimumab. (Table 1.) The range of anti-TNF cut-offs used for the dichotimisation of test outcomes is illustrated in Supplement 5 (Supplement 5 Tables 1-3). The risk of bias of studies varied. The greatest threat to validity was high risk of bias in patient selection, for example studies did not enrol a consecutive or randomly selected patient group. This was present in nearly 80% of included studies (Supplement 6 Table 1 and Supplement 6 Figure 1).

The studies were heterogeneous with respect to type of test used (e.g. commercial or in-house ELISA, RIA, HMSA), criteria for establishing response or lack of regaining response (e.g. use of the CDAI or the physician’s global assessment score), and population examined (responders or patients with secondary loss of response). Sensitivity and specificity pairs are summarised in Figure 2 for antibodies to anti-TNF and Figure 3 for anti-TNF trough levels.

The paired Forest plots show that sensitivity and specificity of using anti-TNFs or antibodies produced against anti-TNFs to predict response or loss of response varies greatly among studies with senstivity revealing generally greater variation. Sensitivity analysis suggests assay type may explain some of the variation in results between studies of anti-infliximab antibodies, however there was considerable

For peer review only

heterogeneity between numerous study covariates (population, assay type, response criterion) and we do not know whether these might fully explain the large differences in results between studies.

For peer review only

Table 1 Major features of studies included for hierarchical meta-analyses

STUDY	DRUG	DIAGNOSIS	RESPONSE/LOR	TEST	RESPONSE MEASURE
Trough antibodies to Infliximab as predictor of loss of or lack of regaining response					
Ben-Horin 2012[17]	IFX ADA	IBD ~0.9 CD	LOR	ELISA	PJ
Candon 2005[18]	IFX	CD	LOR	ELISA	UC
Pariente 2012[19]	IFX	CD & UC	LOR	ELISA	PJ or HBI
Baert 2014[20]	IFX	IBD ~0.8 CD	LOR	HMSA	PJ
Vande Casteele 2013[21]	IFX	IBD ~0.70 CD	LOR	HMSA	CRP TC
Ainsworth 2008[22]	IFX	CD	LOR	RIA	PJ
Steenholdt 2014[23]	IFX	CD	LOR	RIA	CDAI
Farrell 2003[24]	IFX	CD	Resp	ELISA	PJ
Hanauer 2004[25]	IFX	CD	Resp	ELISA	CDAI
Imaeda 2012[26]	IFX	CD	Resp	ELISA	CDAI
Kong 2011[27] abstract	IFX	IBD ~.83 CD	Resp	ELISA	PJ
Kopylov 2012[28]	IFX	CD	Resp	ELISA	PJ
Marzo 2014[29] abstract	IFX	NR	Resp	ELISA	CDAI
Nagore 2015[30] abstract	IFX	IBD ~.86 CD	Resp	ELISA	PJ
Steenholdt 2013[31]	IFX	CD	Resp	ELISA	PJ
Bodini 2014[32] abstract	IFX	CD	Resp	HMSA	HBI
Vande Casteele 2013[21]	IFX	IBD ~0.70 CD	Resp	HMSA	CRP TC
Steenholdt 2011[33]	IFX	CD	Resp	RIA	PJ ST
Ben-Horin 2011[34]	IFX	IBD ~0.82 CD	Resp	NR	ST
Dauer 2013[35] abstract	IFX	CD ~.83 CD	Resp	NR	PJ
Trough antibodies to Adalimumab as predictor of loss of or lack of regaining response					
Imaeda 2014[36]	ADA	CD	Resp	ELISA	CRP
Mazor 2014 [37]	ADA	CD	Resp	ELISA	PJ + CRP
Roblin 2014[38]	ADA	CD	Resp	ELISA	CDAI
Frederiksen 2014[39]	ADA	IBD	Resp	RIA	PJ BM
West 2008[40]	ADA	CD	Resp	RIA	PJ
Ben-Horin 2012[17]	IFX ADA	IBD ~0.9 CD	LOR	ELISA	SA
Infliximab trough level as predictor of loss of or lack of regaining response					
Ainsworth 2008[22]	IFX	CD	LOR	RIA	PJ
Steenholdt 2014[23]	IFX	CD	LOR	RIA	CDAI
Bortlik 2013[41]	IFX	CD	Resp	ELISA	PJ
Cornillie 2014 [42]	IFX	CD	Resp	ELISA	CDAI
Hibi 2014[43]	IFX	CD	Resp	ELISA	CDAI
Imaeda 2012[26]	IFX	CD	Resp	ELISA	CDAI
Kopylov 2012[28]	IFX	CD	Resp	ELISA	PJ
Yanai 2012[44] abstract	IFX	CD	Resp	ELISA	PJ
Ben-Basset 2013[45] abstract	IFX	IBD ~.93 CD	Resp	HMSA	HBI
Steenholdt 2011[33]	IFX	CD	Resp	RIA	PJ
Maser 2006[46]	IFX	CD	Resp	ELISA	HBI
Adalimumab trough level as predictor of loss of or lack of regaining response					
Chiu 2013[47]	ADA	CD	LOR	ELISA	CDAI
Imaeda 2014[36]	ADA	CD	Resp	ELISA	CRP
Mazor 2014[37]	ADA	CD	Resp	ELISA	PJ + CRP
Roblin 2014[38]	ADA	CD	Resp	ELISA	CDAI
Frederiksen 2014[39]	ADA	IBD	Resp	RIA	PJ BM

Diagnosis = study patient population; LOR = patients with loss of response ; Response = responding patients; Response measure = method used for defining clinical response; ADA = Adalimumab; IFX = Infliximab; CD = Crohn's disease; IBD = inflammatory bowel disease; NR=Not Reported; ELISA = enzyme linked immunoassay; HMSA= Homogenous Mobility Shift Assay; RIA = radioimmunoassay; CDAI = Crohn's disease activity index score; CRP = C reactive protein level; PJ = physicians' judgement; PJ BM = physicians' judgement and biological measure; HBI = Harvey Bradshaw Index score; SA = switch anti-TNF; ST = stop anti-TNF; TC = treatment change.

For peer review only

Infliximab trough level tests for loss of response or lack of regaining response

Of eleven included studies, two were reported only as abstracts (Ben-Basset, 2013[45] and Yanai, 2012[44]). The Meta-analysis (Figure 4) yielded a pooled summary point of 66% sensitivity and 81% specificity (other test accuracy statistics are summarised in Supplement 7 Table 1). Sensitivity analysis in which only studies of responder populations were included generated very similar results as did analysis that only included studies with ELISA tests.

Antibodies to Infliximab tests for loss of response or lack of regaining response

Of twenty included studies, five were reported as abstracts.[27 29 30 32 35] Sensitivity and specificity pairs are summarised in Figure 5. The pooled summary points for sensitivity and specificity were 56% and 79% respectively (Figure 5). Only minor differences were introduced in the test accuracy outcomes (e.g. 60% and 81% for sensitivity and specificity respectively) in a sensitivity analysis when two influential studies were omitted from the analysis.[21 25] Sensitivity analyses in which only responder studies were included had little effect. Sensitivity analysis in which only ELISA studies were included showed an improvement in specificity at the expense of sensitivity, and a reduction in the heterogeneity of specificity measurements (Figure 5).

Adalimumab or anti-Adalimumab antibody levels as tests for loss of response or lack of regaining response

Far fewer studies of Adalimumab-treated patients were available compared to Infliximab (Table 1). Meta-analysis of Adalimumab-treated patients yielded slightly lower test accuracy statistics with wider uncertainty around them compared to those found for Infliximab studies (Supplement 7 Table 1 and Supplement 7 Figure 1).

Combined assessment of anti-TNF levels and antibodies to anti-TNF

Three independent studies reported both drug and antibody test results by individual in relation to the individual's clinical status, response / loss of response [23 26] or regaining response / not regaining response.[36] These studies allowed calculation of the number of patients in each of the two clinical states distributed to each of the four possible combinations of test result.[23 26 36] The results summarised in Table 2 and Table 3 indicate the probability of loss of response to anti-TNF and Table 4 summarises the probability of not regaining response to Infliximab according to each possible test result category. These test results are reasonably similar to those from our meta-analysis of single test studies. This comparison should be viewed in the light of the considerable uncertainty which exists because of the small number of studies measuring both drug and antibody levels in the same individuals, and their small size.

For peer review only - http://bmjopen.bmj.com/site/about/guidelines.xhtml

Table 2 Combined assessment of Adalimumab and anti-Adalimumab levels for responders receiving Adalimumab

Imaeda 2014[36]	ADAbs +	ADAbs –	TOTAL	Population & anti-TNFα therapy; Tests
Anti-TNFα –	LOR = 8<br>RESP = 0	LOR = 2<br>RESP = 2	LOR = 10<br>RESP = 2	Responders on Adalimumab maintenance.<br>ELISA. Prevalence of LOR = 37.5%
Anti-TNFα +	LOR = 2<br>RESP = 4	LOR = 3<br>RESP = 19	LOR = 5<br>RESP = 23	
TOTAL	LOR = 10<br>RESP = 4	LOR = 5<br>RESP = 21	LOR = 15<br>RESP = 25	

The probability of a patient returning each of the four possible test result combinations was:
ADAbs +/ Anti-TNFα – = 0.200; ADAbs +/Anti-TNFα + = 0.150; ADAbs –/Anti-TNFα – = 0.10; ADAbs –/Anti-TNFα + = 0.550.
The probabilities of losing response according to category of test result were: 1.00, 0.333, 0.500 and 0.136 respectively. ADAbs – anti-drug antibodies; RESP – responders; LOR – loss of response

Table 3 Combined assessment of Infliximab and anti-Infliximab for responders receiving Infliximab

Imaeda 2012[26]	ADAbs +	ADAbs –	TOTAL	Population & anti-TNFα therapy; Tests
Anti-TNFα –	LOR = 9<br>RESP = 1	LOR = 0<br>RESP = 7	LOR = 9<br>RESP = 8	Responders on Infliximab maintenance.<br>ELISA. Prevalence of LOR = 29.3%
Anti-TNFα +	LOR = 3<br>RESP = 3	LOR = 5<br>RESP = 30	LOR = 8<br>RESP = 33	
TOTAL	LOR = 12<br>RESP = 4	LOR = 5<br>RESP = 37	LOR = 17<br>RESP = 41	

The probability of a patient returning each of the four possible test result combinations was:
ADAbs +/ Anti-TNFα – = 0.172; ADAbs +/ Anti-TNFα + = 0.103; ADAbs – /Anti-TNFα – = 0.121; ADAbs –/Anti-TNFα + = 0.603.
The probabilities of losing response according to category of test result were: 0.900, 0.500, 0.000 and 0.143 respectively. ADAbs – anti-drug antibodies; RESP – responders; LOR – loss of response

Table 4 Combined assessment of Infliximab and anti-Infliximab for people with loss of response receiving Infliximab

Steenholdt 2014[23]	ADAbs +	ADAbs –	TOTAL	Population & anti-TNFα therapy; Tests
Anti-TNFα –	NOR = 8<br>RESP = 6	NOR = 2<br>RESP = 1	NOR = 10<br>RESP = 7	Failure on Infliximab, continued failure or gain of response at 12 weeks.<br>RIA. Prevalence of NOR = 44.9%
Anti-TNFα +	NOR = 1<br>RESP = 3	NOR = 20<br>RESP = 28	NOR = 21<br>RESP = 31	
TOTAL	NOR = 9<br>RESP = 9	NOR = 22<br>RESP = 29	NOR = 31<br>RESP = 38	

The probability of a patient returning each of the four possible test result combinations was:
ADAbs +/ Anti-TNFα – = 0.203; ADAbs +/ Anti-TNFα + = 0.058; ADAbs – /Anti-TNFα – = 0.0.043; ADAbs – /Anti-TNFα + = 0.696.
The probabilities of failing to gain a response according to category of test result were: 0.571, 0.250, 0.667 and 0.417 respectively. ADAbs – anti-drug antibodies; RESP – responders; LOR – loss of response; NOR – no regain of response

Predictive values of drug and anti-drug antibody tests for LOR or failure to regain response

For peer review only

In the Cochrane Handbook for Systematic Reviews of Diagnostic Test Accuracy, Bossuyt et al. (2013) [14] suggest that predictive values are more widely and readily appreciated than alternative test accuracy statistics such as sensitivity and specificity. Negative and positive predictive values vary according to prevalence of the condition being tested for (in this case lack of response). We have meta-analysed the prevalence across the included studies and used this with its 95% CI as a guide to the approximate prevalence in which the tests would be performed in practice. The predictive values for each type of test across the relevant prevalence ranges are summarised in Figure 6. As prevalence increases positive predictive value increases and negative predictive value decreases.

Although pooled prevalence varies somewhat amongst the four collections of studies the resulting positive and negative predictive values are similar and range between about 70% and 80% implying that between 20% and 30% of positive and negative test results are likely to be incorrect.

In January 2017 we updated our included studies by searching all citations of, and included studies in, five relevant systematic reviews (see Supplement 2 Figure 1).[6 7 48-50] After removal of duplicates and the application of our inclusion criteria this yielded three[51-53] and five [52 54-57] additional studies respectively for trough Infliximab and trough Adalimumab levels (Supplement 8 Table 1). Addition of the former to our meta-analysis had almost no influence on our estimates of test accuracy (Supplement 8 Figure 1, Supplement 8 Table 2, Supplement 8 Figure 2); the addition of the Adalimumab studies to our meta-analysis also had very little influence on our estimates of test accuracy except a modest reduction in their uncertainty despite doubling the number of available studies (Supplement 8 Figure 1, Supplement 8 Table 3, Supplement 8 Figure 3).

For peer review only

DISCUSSION

The meta-analysis results indicate that the accuracy of tests for predicting lack of response was moderate and that about 20 to 30% of both positive and negative test results are likely to be incorrect, with large unexplained heterogeneity between studies. The number of studies on Adalimumab treated patients was too small to draw firm conclusions but the available evidence suggests similar performance to the tests for Infliximab and for antibodies to Infliximab.

The sensitivity analyses indicated that much of the variation seen in the Forest plots and ROC space could not be explained by our measures of test type and population. Test performance is dependent on cut-offs used for anti-TNF and antibodies to anti-TNF agents and on the time of testing. However, this was not investigated in sensitivity analyses as cut-offs vary by test type as well as within different types of tests and an agreed cut-off that is transferable between studies and populations has yet to be identified. Furthermore, time of testing was not investigated as all but one study [47] reported that anti TNFs levels considered in the studies were trough levels

Updating the searches found an extra seven studies, however these made no meaningful difference to the test accuracy estimates. The study designs were largely similar to those in the previous studies. However, there appears to have been a recent waning of interest in anti-drug antibodies, possibly attributable to publication of studies indicating their transitory and varying persistence during treatment, while interest in endoscopic healing as an outcome appears to have increased. Additional single arm test accuracy studies may not add significant further understanding in this field. Of more value would be head to head test accuracy comparisons in the same population, and studies integrating drug levels with other predictive factors to enable more accurate predictions of loss of response.

Our meta-analyses included studies using different tests for measuring levels of anti-TNF agents and antibodies to anti-TNFs. Although radioimmunoassay and HMSA tests were used in some of our included studies the bulk of the tests employed were ELISA tests (26/42, 62%) encompassing various commercial ELISA kits and ELISAs developed “in house” by investigators. Several full publications and abstracts have addressed the issue of whether different test methods (e.g. solid phase ELISAs, liquid phase assays such as RIA or HMSA) deliver the same quantitative estimates of drug and antibody levels in patient samples. [21 23 26 28 32 36 58-76] Because there is no consensus about what constitutes a gold standard test, it is difficult to draw conclusions from these studies other than that some differences in performance have been documented. Interestingly, the observed variation in our meta-analysis could not be explained by the different tests used.

Although the accuracy of the tests for predicting lack of response was found to be moderate this does not necessarily mean they must lack clinical utility. However, clinicians are likely to be interested in a combined assessment of anti-TNF levels and antibodies to anti-TNF, for which limited accuracy data is

For peer review only

available.[23 26 36] Diagnostic tests may alter clinical decisions and actions, so evidence beyond test accuracy is required to evaluate clinical value.[77] Such evidence is best obtained in randomised trials (i.e. test and treat investigations) but this is currently sparse.[77]

Two recent RCTs have compared clinical outcomes between patients whose treatment was directed by algorithms informed by tests for Infliximab and/or antibodies to Infliximab versus patients who received treatment uninformed by testing.[23 78] In the TAXIT trial[78] IBD patients responding to Infliximab had their dose regimen optimised according to a test-algorithm with the aim to bring patients within the therapeutic range and prevent loss of response. However after randomisation to clinically-based or test-based dosing, no clinical benefit was observed for CD patients at one year. Steenholdt et al. (2014)[23] investigated patients who had lost response to Infliximab, using a test-algorithm to predict the reason for loss of response and adjust treatment accordingly. In this equivalence study no difference in clinical benefit was observed for the test-algorithm group relative to the control group who were prescribed dose intensification. It is notable in this study that for many patients (14/33; 42%) clinicians failed to implement the test-algorithm directive, implying that they may have lacked confidence in the test results or that they considered other factors of overriding importance; as pointed out by Ferrante di Ruffano et al. (2012)[77]. Such phenomena (lack of equipoise) complicate assessments of test value. Both of these RCTs reported cost savings in the test-algorithm arm associated with reduced use of Infliximab.

This is the first meta-analysis of predictive accuracy of these tests and offers an alternative perspective to earlier meta-analyses. We were able to include more studies than in earlier meta-analyses and have looked at both drug tests as well as tests for anti-drug antibodies, and have included studies of patients receiving either Infliximab or Adalimumab therapies. There was significant heterogeneity between studies, including in the test, outcome measurement and findings, making clinical interpretation difficult.

The meta-analysis results should be viewed with some caution because of the high risk of bias in many of the included studies, and because the lack of sufficient numbers of studies precluded subgroup meta-analyses of some types of test (e.g. RIA, HMSA).

CONCLUSIONS

The available evidence suggests that these tests have modest predictive accuracy for clinical status and that about 20 to 30% of test results would be likely to be incorrect. However, higher quality head to head test accuracy studies are required to enable differentiation between different types of tests and cut-offs, with consistent outcome measurement in the same population. In published trials the tests have been used for adjusting dose or treatment of patients whose clinical status has already been defined by

For peer review only - http://bmjopen.bmj.com/site/about/guidelines.xhtml

other criteria. More clinical trial evidence from test-treat studies is required before the clinical utility of the tests can be reliably evaluated.

For peer review only

Competing interests: None

Source of funding: This work was commissioned by the NIHR HTA Programme as project number 14/69/03

Aileen Clarke and Sian Taylor-Phillips are partly supported by the National Institute for Health Research (NIHR) Collaboration for Leadership in Applied Health Research and Care West Midlands at the University Hospitals Birmingham NHS Foundation Trust.

Data sharing: All data is available from authors upon request

Contributions: KF and MC drafted the paper. RC developed the search strategy and undertook searches. MC, KF, STP, AT and DS conducted the systematic review. MC conducted the data analysis. PS and AC provided project management and funding acquisition. RA provided clinical comment and guidance. KF, MC, STP, RC, AT, DS, HM, PA, PS, AC and RA contributed to protocol development, commented on drafts of the paper and approved the final version.

References

1. Ben-Horin S, Chowers Y. Tailoring anti-TNF therapy in IBD: drug levels and disease activity. Nature Reviews Gastroenterology & Hepatology 2014;**11**(4):243-55 doi: http://dx.doi.org/10.1038/nrgastro.2013.253[published Online First: Epub Date]|.

2. Targan SR, Hanauer SB, van Deventer SJ, et al. A short-term study of chimeric monoclonal antibody cA2 to tumor necrosis factor alpha for Crohn's disease. Crohn's Disease cA2 Study Group. N Engl J Med 1997;**337**(15):1029-35 doi: 10.1056/nejm199710093371502[published Online First: Epub Date]|.

3. Hanauer SB, Sandborn WJ, Rutgeerts P, et al. Human anti-tumor necrosis factor monoclonal antibody (adalimumab) in Crohn's disease: the CLASSIC-I trial. Gastroenterology 2006;**130**(2):323-33 doi: 10.1053/j.gastro.2005.11.030[published Online First: Epub Date]|.

4. Gisbert JP, Panes J. Loss of response and requirement of infliximab dose intensification in Crohn's disease: a review. Am J Gastroenterol 2009;**104**(3):760-7 doi: 10.1038/ajg.2008.88[published Online First: Epub Date]|.

5. de Boer N, Lowenberg M, Hoentjen F. Management of Crohn's disease in poor responders to adalimumab. Clin Exp Gastroenterol 2014;**7**:83-92 doi: http://dx.doi.org/10.2147/CEG.S47627[published Online First: Epub Date]|.

6. Nanda KS, Cheifetz AS, Moss AC. Impact of antibodies to infliximab on clinical outcomes and serum infliximab levels in patients with inflammatory bowel disease (IBD): a meta-analysis. Am J Gastroenterol 2013;**108**(1):40-7 doi: http://dx.doi.org/10.1038/ajg.2012.363[published Online First: Epub Date]|.

7. Paul S, Moreau AC, Del Tedesco E, et al. Pharmacokinetics of adalimumab in inflammatory bowel diseases: a systematic review and meta-analysis. Inflamm Bowel Dis 2014;**20**(7):1288-95 doi: http://dx.doi.org/10.1097/MIB.0000000000000037[published Online First: Epub Date]|.

8. Garces S, Demengeot J, Benito-Garcia E. The immunogenicity of anti-TNF therapy in immune-mediated inflammatory diseases: a systematic review of the literature with a meta-analysis. Ann Rheum Dis

For peer review only - http://bmjopen.bmj.com/site/about/guidelines.xhtml

2013;**72**(12):1947-55 doi: http://dx.doi.org/10.1136/annrheumdis-2012-202220[published Online First: Epub Date]|.

9. Lee LY, Sanderson JD, Irving PM. Anti-infliximab antibodies in inflammatory bowel disease: prevalence, infusion reactions, immunosuppression and response, a meta-analysis. Eur J Gastroenterol Hepatol 2012;**24**(9):1078-85 doi: 10.1097/MEG.0b013e32835558cf[published Online First: Epub Date]|.

10. Pepe NS. *The statistical evaluation of medical tests for classification and prediction*. New York: Oxford University Press, 2003.

11. Whiting PF, Rutjes AWS, Westwood ME, et al. QUADAS-2: A Revised Tool for the Quality Assessment of Diagnostic Accuracy Studies. Ann Intern Med 2011;**155**(8):529-36 doi: 10.7326/0003-4819-155-8-201110180-00009[published Online First: Epub Date]|.

12. Harbord RM, Deeks JJ, Egger M, et al. A unification of models for meta-analysis of diagnostic accuracy studies. Biostatistics 2007;**8**(2):239-51 doi: 10.1093/biostatistics/kxl004[published Online First: Epub Date]|.

13. Harbord R, Whiting P. metandi: Meta-analysis of diagnostic accuracy using hierarchical logistic regression. Stata Journal 2009;**9**(2):211-29

14. Bossuyt PM, Davenport C, Deeks JJ, et al. Chapter 11: Interpreting results and drawing conclusions. In: Deeks JJ, Bossuyt PM, Gatsonis C, eds. Cochrane Handbook for Systematic Reviews of Diagnostic Test Accuracy Version 09: The Cochrane Collaboration, 2013.

15. Wallace BC, Schmid CH, Lau J, et al. Meta-Analyst: software for meta-analysis of binary, continuous and diagnostic data. BMC Med Res Methodol 2009;**9**:80 doi: 10.1186/1471-2288-9-80[published Online First: Epub Date]|.

16. Deeks JJ, Macaskill P, Irwig L. The performance of tests of publication bias and other sample size effects in systematic reviews of diagnostic test accuracy was assessed. J Clin Epidemiol 2005;**58**(9):882-93 doi: 10.1016/j.jclinepi.2005.01.016[published Online First: Epub Date]|.

17. Ben-Horin S, Mazor Y, Yanai H, et al. The decline of anti-drug antibody titres after discontinuation of anti-TNFs: implications for predicting re-induction outcome in IBD. Aliment Pharmacol Ther 2012;**35**(6):714-22 doi: http://dx.doi.org/10.1111/j.1365-2036.2012.04997.x[published Online First: Epub Date]|.

18. Candon S, Mosca A, Ruemmele F, et al. Clinical and biological consequences of immunization to infliximab in pediatric Crohn's disease. Clin Immunol 2006;**118**(1):11-9

19. Pariente B, Pineton de Chambrun G, Krzysiek R, et al. Trough levels and antibodies to infliximab may not predict response to intensification of infliximab therapy in patients with inflammatory bowel disease. Inflamm Bowel Dis 2012;**18**(7):1199-206 doi: http://dx.doi.org/10.1002/ibd.21839[published Online First: Epub Date]|.

20. Baert F, Drobne D, Gils A, et al. Early trough levels and antibodies to infliximab predict safety and success of reinitiation of infliximab therapy. Clin Gastroenterol Hepatol 2014;**12**(9):1474-81.e2 doi: http://dx.doi.org/10.1016/j.cgh.2014.01.033[published Online First: Epub Date]|.

21. Vande Casteele N, Gils A, Singh S, et al. Antibody response to infliximab and its impact on pharmacokinetics can be transient. Am J Gastroenterol 2013;**108**(6):962-71 doi: http://dx.doi.org/10.1038/ajg.2013.12[published Online First: Epub Date]|.

22. Ainsworth MA, Bendtzen K, Brynskov J. Tumor necrosis factor-alpha binding capacity and anti-infliximab antibodies measured by fluid-phase radioimmunoassays as predictors of clinical efficacy of infliximab in Crohn's disease. Am J Gastroenterol 2008;**103**(4):944-8

23. Steenholdt C, Brynskov J, Thomsen OO, et al. Individualised therapy is more cost-effective than dose intensification in patients with Crohn's disease who lose response to anti-TNF treatment: a randomised,

For peer review only

controlled trial. Gut 2014;**63**(6):919-27 doi: http://dx.doi.org/10.1136/gutjnl-2013-305279[published Online First: Epub Date]|.

24. Farrell RJ, Alsahli M, Jeen YT, et al. Intravenous hydrocortisone premedication reduces antibodies to infliximab in Crohn's disease: a randomized controlled trial. Gastroenterology 2003;**124**(4):917-24 doi: 10.1053/gast.2003.50145[published Online First: Epub Date]|.

25. Hanauer SB, Wagner CL, Bala M, et al. Incidence and importance of antibody responses to infliximab after maintenance or episodic treatment in Crohn's disease. Clin Gastroenterol Hepatol 2004;**2**(7):542-53

26. Imaeda H, Andoh A, Fujiyama Y. Development of a new immunoassay for the accurate determination of anti-infliximab antibodies in inflammatory bowel disease. J Gastroenterol 2012;**47**(2):136-43 doi: http://dx.doi.org/10.1007/s00535-011-0474-y[published Online First: Epub Date]|.

27. Kong JY, Bundell CS, Pawlik J, et al. Trough serum infliximab level, anti-infliximab antibody status and response to infliximab maintenance treatment in inflammatory bowel disease (IBD). J Gastroenterol Hepatol 2011;**26**:59-60 doi: http://dx.doi.org/10.1111/j.1440-1746.2011.06824.x[published Online First: Epub Date]|.

28. Kopylov U, Mazor Y, Yavzori M, et al. Clinical utility of antihuman lambda chain-based enzyme-linked immunosorbent assay (ELISA) versus double antigen ELISA for the detection of anti-infliximab antibodies. Inflamm Bowel Dis 2012;**18**(9):1628-33 doi: http://dx.doi.org/10.1002/ibd.21919[published Online First: Epub Date]|.

29. Marzo M, Armuzzi A, Felice C, et al. Role of trough levels and antibodies to infliximab in the evaluation of loss of response and infusion reactions to infliximab therapy in inflammatory bowel disease. Dig Liver Dis 2014;**46**:S77

30. Nagore D, Ruiz Del Agua A, Pascual J, et al. Therapeutic Cut-off of Infliximab in Patients with Inflammatory Bowel Diseases (TU1325). Gastroenterology 2015;**148**(4 Suppl 1):S-860

31. Steenholdt C, Palarasah Y, Bendtzen K, et al. Pre-existing IgG antibodies cross-reacting with the Fab region of infliximab predict efficacy and safety of infliximab therapy in inflammatory bowel disease. Aliment Pharmacol Ther 2013;**37**(12):1172-83 doi: http://dx.doi.org/10.1111/apt.12330[published Online First: Epub Date]|.

32. Bodini G, Savarino V, Dulbecco P, et al. ELISA vs. HMSA: A comparison between two different methods for the evaluation of adalimumab serum concentration and anti-adalimumab antibodies Preliminary data. Journal of Crohn's and Colitis 2014;**8**:S278

33. Steenholdt C, Bendtzen K, Brynskov J, et al. Cut-off levels and diagnostic accuracy of infliximab trough levels and anti-infliximab antibodies in Crohn's disease. Scand J Gastroenterol 2011;**46**(3):310-8 doi: http://dx.doi.org/10.3109/00365521.2010.536254[published Online First: Epub Date]|.

34. Ben-Horin S, Yavzori M, Katz L, et al. The immunogenic part of infliximab is the F(ab')2, but measuring antibodies to the intact infliximab molecule is more clinically useful. Gut 2011;**60**(1):41-8 doi: http://dx.doi.org/10.1136/gut.2009.201533[published Online First: Epub Date]|.

35. Dauer RM, Yarur AJ, Abreu MT. Infliximab re-induction outcomes after a failure to treatment. Gastroenterology 2013;**144**(5 Suppl):S-430

36. Imaeda H, Takahashi K, Fujimoto T, et al. Clinical utility of newly developed immunoassays for serum concentrations of adalimumab and anti-adalimumab antibodies in patients with Crohn's disease. J Gastroenterol 2014;**49**(1):100-9 doi: http://dx.doi.org/10.1007/s00535-013-0803-4[published Online First: Epub Date]|.

37. Mazor Y, Almog R, Kopylov U, et al. Adalimumab drug and antibody levels as predictors of clinical and laboratory response in patients with Crohn's disease. Aliment Pharmacol Ther 2014;**40**(6):620-8 doi: http://dx.doi.org/10.1111/apt.12869[published Online First: Epub Date]|.

For peer review only - http://bmjopen.bmj.com/site/about/guidelines.xhtml

38. Roblin X, Marotte H, Rinaudo M, et al. Association between pharmacokinetics of adalimumab and mucosal healing in patients with inflammatory bowel diseases. Clin Gastroenterol Hepatol 2014;**12**(1):80-84.e2 doi: http://dx.doi.org/10.1016/j.cgh.2013.07.010[published Online First: Epub Date]|.

39. Frederiksen MT, Ainsworth MA, Brynskov J, et al. Antibodies Against Infliximab Are Associated with De Novo Development of Antibodies to Adalimumab and Therapeutic Failure in Infliximab-to-Adalimumab Switchers with IBD. Inflamm Bowel Dis 2014;**20**(10):1714-21 doi: http://dx.doi.org/10.1097/MIB.0000000000000138[published Online First: Epub Date]|.

40. West RL, Zelinkova Z, Wolbink GJ, et al. Immunogenicity negatively influences the outcome of adalimumab treatment in Crohn's disease. Aliment Pharmacol Ther 2008;**28**(9):1122-6 doi: 10.1111/j.1365-2036.2008.03828.x[published Online First: Epub Date]|.

41. Bortlik M, Duricova D, Malickova K, et al. Infliximab trough levels may predict sustained response to infliximab in patients with Crohn's disease. Journal of Crohn's & colitis 2013;**7**(9):736-43 doi: http://dx.doi.org/10.1016/j.crohns.2012.10.019[published Online First: Epub Date]|.

42. Cornillie F, Hanauer SB, Diamond RH, et al. Postinduction serum infliximab trough level and decrease of C-reactive protein level are associated with durable sustained response to infliximab: a retrospective analysis of the ACCENT I trial. Gut 2014;**63**(11):1721-7 doi: http://dx.doi.org/10.1136/gutjnl-2012-304094[published Online First: Epub Date]|.

43. Hibi T, Sakuraba A, Watanabe M, et al. C-reactive protein is an indicator of serum infliximab level in predicting loss of response in patients with Crohn's disease. J Gastroenterol 2014;**49**(2):254-62 doi: http://dx.doi.org/10.1007/s00535-013-0807-0[published Online First: Epub Date]|.

44. Yanai H, Mlynarsky L, Ron Y, et al. The questionable value of infliximab trough levels during prolonged maintenance therapy. Gastroenterology 2012;**142**(5 Suppl):S788-9

45. Ben-Bassat O, Romanova A, Iacono A, et al. Association of serum infliximab and antibodies to infliximab to long-term clinical outcome and mucosal healing in crohn's disease. Gastroenterology 2013;**144**(5 Suppl):S-775

46. Maser EA, Villela R, Silverberg MS, et al. Association of trough serum infliximab to clinical outcome after scheduled maintenance treatment for Crohn's disease. Clin Gastroenterol Hepatol 2006;**4**(10):1248-54 doi: 10.1016/j.cgh.2006.06.025[published Online First: Epub Date]|.

47. Chiu YL, Rubin DT, Vermeire S, et al. Serum adalimumab concentration and clinical remission in patients with Crohn's disease. Inflamm Bowel Dis 2013;**19**(6):1112-22 doi: http://dx.doi.org/10.1097/MIB.0b013e3182813242[published Online First: Epub Date]|.

48. Barnes EL, Allegretti JR. Are Anti-Tumor Necrosis Factor Trough Levels Predictive of Mucosal Healing in Patients With Inflammatory Bowel Disease?: A Systematic Review and Meta-Analysis. J Clin Gastroenterol 2016;**50**(9):733-41 doi: 10.1097/MCG.0000000000000441[published Online First: Epub Date]|.

49. Moore C, Corbett G, Moss AC. Systematic Review and Meta-Analysis: Serum Infliximab Levels During Maintenance Therapy and Outcomes in Inflammatory Bowel Disease. J Crohns Colitis 2016;**10**(5):619-25 doi: 10.1093/ecco-jcc/jjw007[published Online First: Epub Date]|.

50. Silva-Ferreira F, Afonso J, Pinto-Lopes P, et al. A Systematic Review on Infliximab and Adalimumab Drug Monitoring: Levels, Clinical Outcomes and Assays. Inflamm Bowel Dis 2016;**22**(9):2289-301 doi: 10.1097/MIB.0000000000000855[published Online First: Epub Date]|.

51. Levesque BG, Greenberg GR, Zou G, et al. A prospective cohort study to determine the relationship between serum infliximab concentration and efficacy in patients with luminal Crohn's disease. Aliment Pharmacol Ther 2014;**39**(10):1126-35 doi: http://dx.doi.org/10.1111/apt.12733[published Online First: Epub Date]|.

For peer review only

52. Ungar B, Levy I, Yavne Y, et al. Optimizing Anti-TNF-alpha Therapy: Serum Levels of Infliximab and Adalimumab Are Associated With Mucosal Healing in Patients With Inflammatory Bowel Diseases. Clin Gastroenterol Hepatol 2016;**14**(4):550-57 e2 doi: 10.1016/j.cgh.2015.10.025[published Online First: Epub Date]|.

53. Reinisch W, Reinink AR, Higgins PD. Factors associated with poor outcomes in adults with newly diagnosed ulcerative colitis. Clin Gastroenterol Hepatol 2015;**13**(4):635-42 doi: 10.1016/j.cgh.2014.03.037[published Online First: Epub Date]|.

54. Bodini G, Giannini EG, Furnari M, et al. Comparison of Two Different Techniques to Assess Adalimumab Trough Levels in Patients with Crohn's Disease. J Gastrointestin Liver Dis 2015;**24**(4):451-6 doi: 10.15403/jgld.2014.1121.244.adb[published Online First: Epub Date]|.

55. Zittan E, Kabakchiev B, Milgrom R, et al. Higher Adalimumab Drug Levels are Associated with Mucosal Healing in Patients with Crohn's Disease. J Crohns Colitis 2016;**10**(5):510-5 doi: 10.1093/ecco-jcc/jjw014[published Online First: Epub Date]|.

56. Yarur AJ, Jain A, Hauenstein SI, et al. Higher Adalimumab Levels Are Associated with Histologic and Endoscopic Remission in Patients with Crohn's Disease and Ulcerative Colitis. Inflamm Bowel Dis 2016;**22**(2):409-15 doi: 10.1097/MIB.0000000000000689[published Online First: Epub Date]|.

57. Morita Y, Imaeda H, Nishida A, et al. Association between serum adalimumab concentrations and endoscopic disease activity in patients with Crohn's disease. J Gastroenterol Hepatol 2016;**31**(11):1831-36 doi: 10.1111/jgh.13400[published Online First: Epub Date]|.

58. Corstjens PL, Fidder HH, Wiesmeijer KC, et al. A rapid assay for on-site monitoring of infliximab trough levels: a feasibility study. Anal Bioanal Chem 2013;**405**(23):7367-75 doi: http://dx.doi.org/10.1007/s00216-013-7154-0[published Online First: Epub Date]|.

59. Steenholdt C, Ainsworth MA, Tovey M, et al. Comparison of techniques for monitoring infliximab and antibodies against infliximab in Crohn's disease. Ther Drug Monit 2013;**35**(4):530-8 doi: http://dx.doi.org/10.1097/FTD.0b013e31828d23c3[published Online First: Epub Date]|.

60. Vande Casteele N, Buurman DJ, Sturkenboom MG, et al. Detection of infliximab levels and anti-infliximab antibodies: a comparison of three different assays. Aliment Pharmacol Ther 2012;**36**(8):765-71 doi: http://dx.doi.org/10.1111/apt.12030[published Online First: Epub Date]|.

61. Wang SL, Ohrmund L, Hauenstein S, et al. Development and validation of a homogeneous mobility shift assay for the measurement of infliximab and antibodies-to-infliximab levels in patient serum. J Immunol Methods 2012;**382**(1-2):177-88 doi: http://dx.doi.org/10.1016/j.jim.2012.06.002[published Online First: Epub Date]|.

62. Steenholdt C, Bendtzen K, Brynskov J, et al. Clinical implications of measuring drug and anti-drug antibodies by different assays when optimizing infliximab treatment failure in Crohn's disease: post hoc analysis of a randomized controlled trial. Am J Gastroenterol 2014;**109**(7):1055-64 doi: http://dx.doi.org/10.1038/ajg.2014.106[published Online First: Epub Date]|.

63. Ruiz-Arguello B, del Agua AR, Torres N, et al. Comparison study of two commercially available methods for the determination of infliximab, adalimumab, etanercept and anti-drug antibody levels. Clin Chem Lab Med 2013;**51**(12):e287-9 doi: 10.1515/cclm-2013-0461[published Online First: Epub Date]|.

64. Daperno M, Frigerio F, Guiotto C, et al. Evaluation of the diagnostic performance of two commercially available tests for infliximab trough levels (IFX-TL) and antibodies to infliximab (ATI) titration in inflammatory bowel disease (IBD). Journal of Crohn's and Colitis 2013;**7**:S213-4

65. Egea-Pujol L, Reddy R, Patel S, et al. Homogenous mobility shift assay (HMSA) overcomes the limitations of elisa and eclia assays for monitoring infliximab (IFX), adalimumab (ADA), and associated anti-drug antibodies in serum. Am J Gastroenterol 2013;**108**:S548 doi: http://dx.doi.org/10.1038/ajg.2013.269[published Online First: Epub Date]|.

For peer review only

66. Eser A, Primas C, Hauenstein S, et al. Comparison of early measurement of infliximab and antibodies-to-infliximab serum levels with standard trough analysis. Gastroenterology 2013;**144**(5 Suppl):S-779

67. Eser A, Primas C, Haunstein S, et al. Detection of anti infliximab antibodies in patients with inflammatory bowel disease (IBD) in the presence of infliximab by homogeneous liquid phase anti infliximab mobility shift assay. Journal of Crohn's and Colitis 2013;**7**:S231-2

68. Greathead L, Kelleher P, Steel A. Development and validation of ELISA to measure serum anti TNFa levels. Journal of Crohn's and Colitis 2014;**8**:S97-8

69. Hauenstein S, Ohrmund L, Salbato J, et al. Comparison of homogeneous mobility shift assay and solid phase elisa for the measurement of drug and anti-drug antibody (ADA) levels in serum from patients treated with anti-TNF biologics. Gastroenterology 2012;**142**(5 Suppl):S-538

70. McTigue M, Sandborn W, Levesque B, et al. Clinical utility of next generation infliximab and antibodies to infliximab assay. Am J Gastroenterol 2013;**108**:S527 doi: http://dx.doi.org/10.1038/ajg.2013.269[published Online First: Epub Date]|.

71. Semmler J, Pilch A, Armbruster F, et al. Development of a new immunoassay for the accurate determination of anti-infliximab antibodies in inflammatory bowel disease. Clin Chem Lab Med 2013;**51 (10)**:eA27-8 doi: http://dx.doi.org/10.1515/cclm-2013-0737[published Online First: Epub Date]|.

72. Ungar B, Anafy A, Kopylov U, et al. The clinical and immunological significance of low level of infliximab in the absence of anti-infliximab antibodies in patients with IBD. Gastroenterology 2014;**146**(5 Suppl):S-245 doi: http://dx.doi.org/10.1016/S0016-5085%2814%2960862-3[published Online First: Epub Date]|.

73. Vande Casteele N, Peeters M, Compernolle G, et al. TNF-responsive cellular based assay reveals neutralizing capacity of anti-adalimumab antibodies in crohn's disease and ulcerative colitis patients. Gastroenterology 2014;**146**(5 Suppl):S-242 doi: http://dx.doi.org/10.1016/S0016-5085%2814%2960852-0[published Online First: Epub Date]|.

74. Wang SL, Ohrmund L, Singh S. Measurement of human anti-chimeric antibodies (Haca) and infliximab levels in patient serum using a novel homogeneous assay. Gastroenterology 2010;**1)**:S684-5

75. Schatz SB, Prell C, Freudenberg F, et al. PA-G-0035 Comparison of different tests for determination of infliximab levels and antibodies against infliximab in pediatric IBD patients. The 46th Annual Meeting of The European Society of Paediatric Gastroenterology, Hepatology and Nutrition. J Pediatr Gastroenterol Nutr 2013;**56 suppl 2**:19

76. Wang SL, Ohrmund L, Hauenstein S, et al. Evaluation of a novel homogeneous mobility shift assay for the measurement of human antibodies-To-Infliximab and infliximab levels in Patient serum. Am J Gastroenterol 2011;**106**:S475-6 doi: http://dx.doi.org/10.1038/ajg.2011.336_9[published Online First: Epub Date]|.

77. Ferrante di Ruffano L, Hyde CJ, McCaffery KJ, et al. Assessing the value of diagnostic tests: a framework for designing and evaluating trials. BMJ 2012;**344**:e686 doi: 10.1136/bmj.e686[published Online First: Epub Date]|.

78. Vande Casteele N, Ferrante M, Van Assche G, et al. Trough Concentrations of Infliximab Guide Dosing for Patients with Inflammatory Bowel Disease. Gastroenterology Forthcoming 2015 doi: 10.1053/j.gastro.2015.02.031[published Online First: Epub Date]|.

For peer review only

Figure legend

Figure 1 PRISMA study flow diagram

Figure 2 Paired forest plots for anti-TNF antibody levels for predicting loss of response or failure to regain response to Infliximab (top) and Adalimumab (bottom)
RES = criterion for determining clinical response, POP = study patient population, RIA = radioimmunoassay, LR = patients with loss of response, R = patients with response, HMSA = homogeneous mobility shift assay, ELISA = enzyme linked immunoassay, UC = unclear, PJ BM = physicians' judgement and biological measure; PJ = physicians' judgement, HBI = Harvey Bradshaw Index score, CDAI = Crohn's disease activity index score, TC = treatment change, ST = stop anti-TNF therapy, CRP = C-reactive protein level, RS = restart anti-TNF after drug holiday, SA = switch anti-TNF

Figure 3 Paired forest plots for trough anti-TNF levels for predicting loss of response or failure to regain response to Infliximab (top) and Adalimumab (bottom)
RES = criterion for determining clinical response, POP = study patient population, RIA = radioimmunoassay, HMSA = homogeneous mobility shift assay, ELISA = enzyme linked immunoassay, LR = patients with loss of response, R = patients with response, UC = unclear, PJ BM = physicians' judgement and biological measure; PJ = physicians' judgement, HBI = Harvey Bradshaw Index score, CDAI = Crohn's disease activity index score, CRP = C-reactive protein level, MH = mucosal healing

Figure 4 Hierarchical meta-analysis of trough Infliximab levels for predicting loss of response or failure to regain response.
Left = all 11 studies, right = responder studies only (n = 9). *The square symbol represents the summary point estimate on the HSROC curve*

Figure 5 Hierarchical meta-analysis of trough levels of antibodies to Infliximab for predicting loss of response or failure to regain response
Top Left = all 20 studies, top right = ELISA studies only (n = 9), lower left all studies minus two influential studies (n=18),[22 23] lower right = responder studies only (n=13). The square symbol represents the summary point estimate on the HSROC curve.

Figure 6 Positive and negative predictive values according to prevalence of lack of response using the pooled summary ROC model estimates of sensitivity and specificity
Data points = PPV and NPV at sROC pooled sensitivity and specificity and pooled prevalence. Vertical dashed lines = pooled prevalence and 95% CIs. Thick curves = PPV and NPV at upper and lower CIs for sensitivity and specificity across the pooled prevalence and its 95% CI. The dashed line ellipses encompass predictive values determined from 95% CIs of prevalence and 95% CI for PPV and NPV at the point prevalence estimate

For peer review only

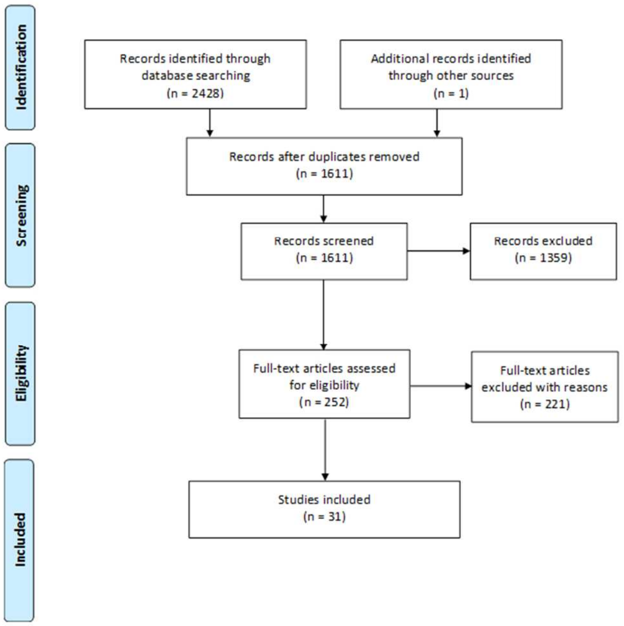

Figure 1 PRISMA study flow diagram

254x190mm (96 x 96 DPI)

For peer review only - http://bmjopen.bmj.com/site/about/guidelines.xhtml

Study	TP	FP	FN	TN	assay	POP	RES	Sensitivity (95% CI)	Specificity (95% CI)
Ben-Horin 2012	3	2	11	11	ELISA	LR	PJ	0.21 [0.05, 0.51]	0.85 [0.55, 0.98]
Candon 2006	6	3	2	11	ELISA	LR	UC	0.75 [0.35, 0.97]	0.79 [0.49, 0.95]
Pariente 2012	4	6	8	21	ELISA	LR	PJorHBI	0.33 [0.10, 0.65]	0.78 [0.58, 0.91]
Baert 2014	9	40	2	81	HMSA	LR	PJ	0.82 [0.48, 0.98]	0.67 [0.58, 0.75]
Casteele 2013	12	31	15	2	HMSA	LR	CRP TC	0.44 [0.25, 0.65]	0.06 [0.01, 0.20]
Ainsworth 2008	8	10	0	9	RIA	LR	PJ	1.00 [0.63, 1.00]	0.47 [0.24, 0.71]
Steenholdt 2014	9	9	22	29	RIA	LR	CDAI	0.29 [0.14, 0.48]	0.76 [0.60, 0.89]
Farrell 2003	19	0	13	21	ELISA	R	PJ	0.59 [0.41, 0.76]	1.00 [0.84, 1.00]
Hanauer 2004	16	13	58	137	ELISA	R	CDAI	0.22 [0.13, 0.33]	0.91 [0.86, 0.95]
Imaeda 2012	12	4	5	37	ELISA	R	CDAI	0.71 [0.44, 0.90]	0.90 [0.77, 0.97]
Kong 2011	4	2	10	14	ELISA	R	PJ	0.29 [0.08, 0.58]	0.88 [0.62, 0.98]
Kopylov 2012	17	5	13	28	ELISA	R	PJ	0.57 [0.37, 0.75]	0.85 [0.68, 0.95]
Marzo 2014	9	9	13	51	ELISA	R	CDAI	0.41 [0.21, 0.64]	0.85 [0.73, 0.93]
Nagore 2015	4	3	3	40	ELISA	R	PJ	0.57 [0.18, 0.90]	0.93 [0.81, 0.99]
Steenholdt 2013	16	2	5	6	ELISA	R	PJ	0.76 [0.53, 0.92]	0.75 [0.35, 0.97]
Bodini 2014	5	3	8	5	HMSA	R	HBI	0.38 [0.14, 0.68]	0.63 [0.24, 0.91]
Casteele 2013a	43	10	17	20	HMSA	R	CRP TC	0.72 [0.59, 0.83]	0.67 [0.47, 0.83]
Steenholdt 2011	21	9	5	50	RIA	R	PJ ST	0.81 [0.61, 0.93]	0.85 [0.73, 0.93]
Ben-Horin 2011	10	19	2	31	UC	R	ST	0.83 [0.52, 0.98]	0.62 [0.47, 0.75]
Dauer 2013	3	1	3	10	UC	R	PJ	0.50 [0.12, 0.88]	0.91 [0.59, 1.00]

Study	TP	FP	FN	TN	assay	POP	RES	Sensitivity (95% CI)	Specificity (95% CI)
Imaeda 2014	10	4	5	21	ELISA	R	CRP	0.67 [0.38, 0.88]	0.84 [0.64, 0.95]
Mazor 2014	14	1	49	54	ELISA	R	PJ	0.22 [0.13, 0.34]	0.98 [0.90, 1.00]
Roblin 2014	10	4	3	5	ELISA	R	CDAI	0.77 [0.46, 0.95]	0.56 [0.21, 0.86]
Frederiksen 2014	9	1	9	28	RIA	R	PJ BM	0.50 [0.26, 0.74]	0.97 [0.82, 1.00]
West 2008	4	1	3	22	RIA	R	PJ	0.57 [0.18, 0.90]	0.96 [0.78, 1.00]
Ben-Horin 2012	3	2	11	11	ELISA	RS	SA	0.21 [0.05, 0.51]	0.85 [0.55, 0.98]

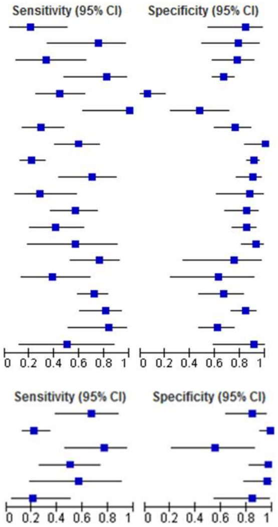

Figure 2 Anti-TNF antibody levels for predicting loss of response or failure to regain response

254x190mm (96 x 96 DPI)

For peer review only - http://bmjopen.bmj.com/site/about/guidelines.xhtml

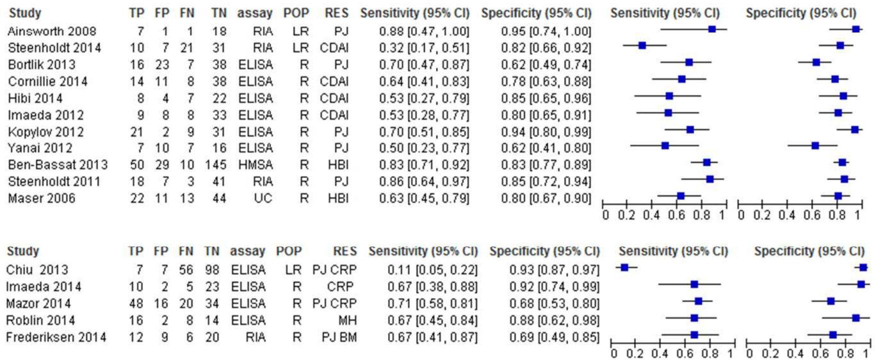

Figure 3 Trough anti-TNF levels for predicting loss of response or failure to regain response

254x190mm (96 x 96 DPI)

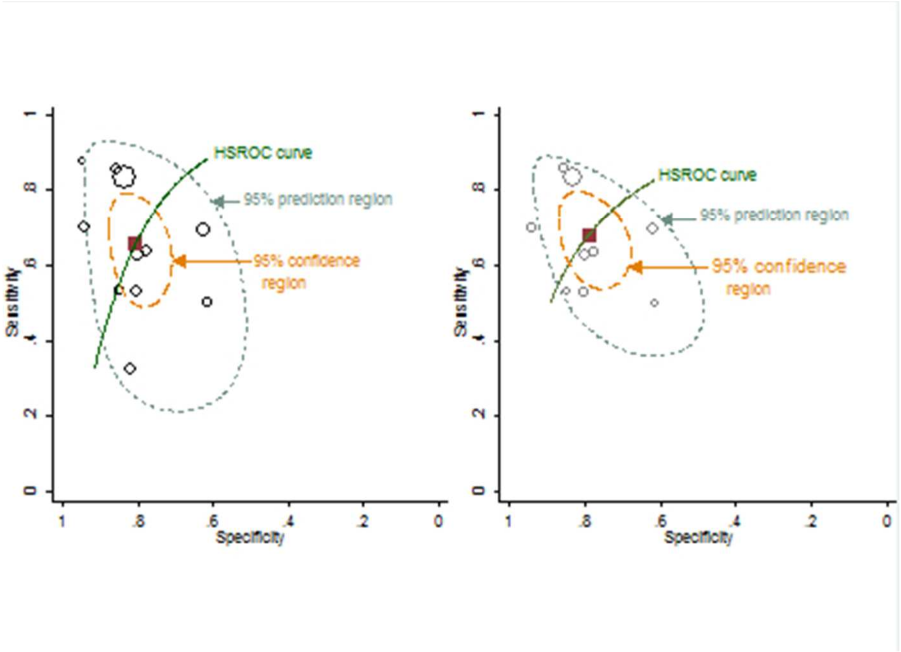

Figure 4 Hierarchical meta-analysis of trough Infliximab levels for predicting loss of response or failure to regain response

158x114mm (72 x 72 DPI)

view only

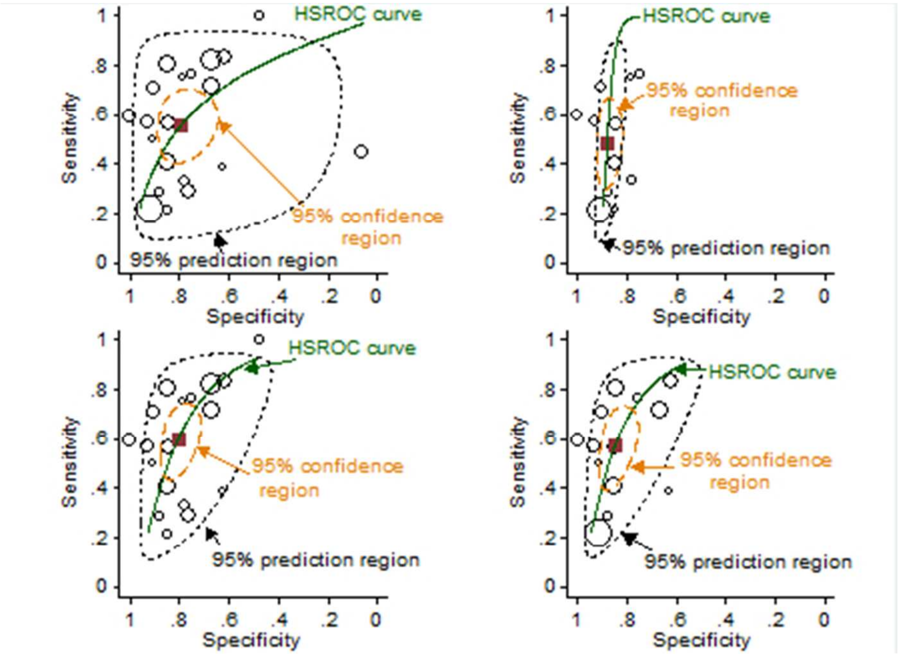

Figure 5 Hierarchical meta-analysis of trough levels of antibodies to Infliximab for predicting loss of response or failure to regain response

158x114mm (72 x 72 DPI)

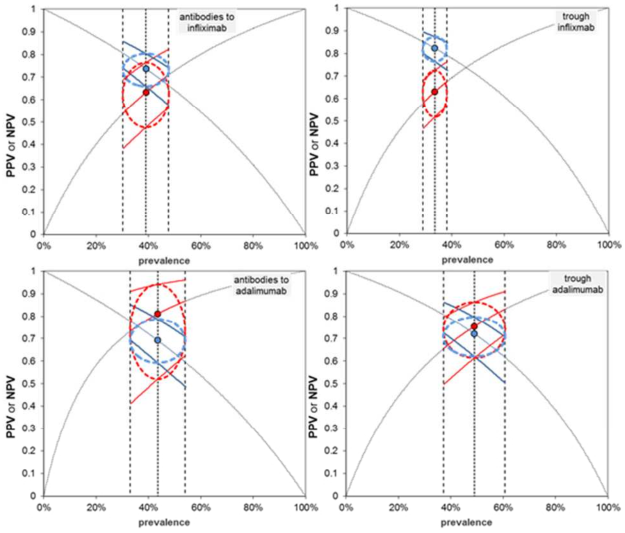

Figure 6 Positive and negative predictive values according to prevalence of lack of response using the pooled summary ROC model estimates of sensitivity and specificity

254x190mm (96 x 96 DPI)

Supplement 1 Search strategy

Ovid MEDLINE(R) 1946 to October Week 2 2014, searched on 22/10/2014

1	adalimumab.mp.	3597
2	ADA.tw.	7105
3	infliximab.mp.	8842
4	IFX.tw.	326
5	((anti-TNF* or antiTNF* or TNF*) adj2 inhibitor*).mp.	2577
6	anti* tumo?r* necrosis* factor*.mp.	3007
7	Tumor Necrosis Factor-alpha/ and Antibodies, Monoclonal/	7682
8	anti* drug* antibod*.tw.	186
9	ADAb.tw.	19
10	1 or 2 or 3 or 4 or 5 or 6 or 7 or 8 or 9	24181
11	lisa* tracker*.mp.	1
12	(immundiagnostik* or immunodiagnostik* or immunediagnostik*).mp.	159
13	(proteomika* or promonitor*).mp.	13
14	exp Enzyme-Linked Immunosorbent Assay/	129174
15	enzyme* link* immunoassay*.mp.	2873
16	enzyme* link* immuno* assay*.mp.	158537
17	ELISA*.mp.	113426
18	11 or 12 or 13 or 14 or 15 or 16 or 17	205224
19	*Radioimmunoassay/	7091
20	(radioimmuno* or radio immuno* or radio-immuno*).mp.	101819
21	RIA.tw.	17353
22	reporter* gene* assay*.mp.	3663
23	RGA.tw.	336
24	semi* fluid* phase* enzyme* immuno*.mp.	0

For peer review only

25	EIA.tw.	8288
26	((homogenous* or homogeneous*) adj1 mobilit* shift* assay*).mp.	4
27	HMSA.tw.	62
28	(Biomonitor* or iLite).tw.	4102
29	(Matriks* Biotek* or Shikari*).mp.	2
30	(Prometheus* or Anser IFX or Anser ADA).mp.	258
31	19 or 20 or 21 or 22 or 23 or 24 or 25 or 26 or 27 or 28 or 29 or 30	124775
32	((monitor* or pharmacokinetic* or measur* or level* or concentration*) adj3 (adalimumab or ADA or infliximab or IFX or Anti-TNF* or Anti-Tumour Necrosis Factor*)).mp.	1087
33	Inflammatory Bowel Diseases/	14444
34	Crohn Disease/	31596
35	crohn*.tw.	32370
36	inflammator* bowel* disease*.tw.	26840
37	IBD.tw.	11936
38	33 or 34 or 35 or 36 or 37	58401
39	(((monitor* or pharmacokinetic* or measur* or level* or concentration*) adj3 (adalimumab or infliximab or Anti-TNF* or AntiTNF* or Anti-Tumour Necrosis Factor*)) and (correlat* or associat* or test performance)).mp.	218
40	10 and 18 and 38	93
41	10 and 31 and 38	19
42	32 and 38	157
43	39 or 40 or 41 or 42	367
44	Animals/ not Humans/	3983380
45	43 not 44	349

For peer review only

Ovid MEDLINE(R) In-Process & Other Non-Indexed Citations October 21, 2014, searched on 22/10/2014

1	adalimumab.mp.	469
2	ADA.tw.	426
3	infliximab.mp.	814
4	IFX.tw.	69
5	((anti-TNF* or antiTNF* or TNF*) adj2 inhibitor*).mp.	308
6	anti* tumo?r* necrosis* factor*.mp.	323
7	anti* drug* antibod*.tw.	39
8	ADAb.tw.	1
9	1 or 2 or 3 or 4 or 5 or 6 or 7 or 8	1824
10	lisa* tracker*.mp.	0
11	(immundiagnostik* or immunodiagnostik* or immunediagnostik*).mp.	2
12	(proteomika* or promonitor*).mp.	0
13	enzyme* link* immunoassay*.mp.	133
14	enzyme* link* immuno* assay*.mp.	3996
15	ELISA*.mp.	8044
16	10 or 11 or 12 or 13 or 14 or 15	10101
17	(radioimmuno* or radio immuno* or radio-immuno*).mp.	1176
18	RIA.tw.	386
19	reporter* gene* assay*.mp.	240
20	RGA.tw.	47
21	semi* fluid* phase* enzyme* immuno*.mp.	0
22	EIA.tw.	357
23	((homogenous* or homogeneous*) adj1 mobilit* shift* assay*).mp.	0
24	HMSA.tw.	5
25	(Biomonitor* or iLite).tw.	343

For peer review only

26	(Matriks* Biotek* or Shikari*).mp.	1
27	(Prometheus* or Anser IFX or Anser ADA).mp.	23
28	17 or 18 or 19 or 20 or 21 or 22 or 23 or 24 or 25 or 26 or 27	2386
29	((monitor* or pharmacokinetic* or measur* or level* or concentration*) adj3 (adalimumab or ADA or infliximab or IFX or Anti-TNF* or Anti-Tumour Necrosis Factor*)).mp.	112
30	crohn*.tw.	2478
31	inflammator* bowel* disease*.tw.	2627
32	IBD.tw.	1480
33	30 or 31 or 32	4400
34	(((monitor* or pharmacokinetic* or measur* or level* or concentration*) adj3 (adalimumab or infliximab or Anti-TNF* or AntiTNF* or Anti-Tumour Necrosis Factor*)) and (correlat* or associat* or test performance)).mp.	30
35	9 and 16 and 33	15
36	9 and 28 and 33	0
37	29 and 33	35
38	34 or 35 or 36 or 37	57

Embase Classic+Embase 1947 to 2014 Week 42, searched on 22/10/2014

1	adalimumab.tw.	7379
2	*adalimumab/	3997
3	ADA.tw.	10848
4	infliximab.tw.	13600
5	*infliximab/	8056
6	IFX.tw.	1722
7	((anti-TNF* or antiTNF* or TNF*) adj2 inhibitor*).tw.	4663
8	anti* tumo?r* necrosis* factor*.tw.	4171

For peer review only

9	*tumor necrosis factor alpha inhibitor/	1283
10	anti* drug* antibod*.tw.	469
11	ADAb.tw.	44
12	*drug antibody/	1528
13	1 or 2 or 3 or 4 or 5 or 6 or 7 or 8 or 9 or 10 or 11 or 12	35630
14	lisa* tracker*.tw.	11
15	(immundiagnostik* or immunodiagnostik* or immunediagnostik*).tw.	74
16	(proteomika* or promonitor*).tw.	27
17	*enzyme linked immunosorbent assay/	14622
18	enzyme* link* immunoassay*.tw.	3275
19	enzyme* link* immuno* assay*.tw.	71923
20	ELISA*.tw.	166866
21	14 or 15 or 16 or 17 or 18 or 19 or 20	207373
22	*radioimmunoassay/	17240
23	(radioimmuno* or radio immuno* or radio-immuno*).tw.	74895
24	RIA.tw.	20769
25	reporter* gene* assay*.tw.	4396
26	RGA.tw.	400
27	semi* fluid* phase* enzyme* immuno*.tw.	1
28	EIA.tw.	10836
29	((homogenous* or homogeneous*) adj1 mobilit* shift* assay*).tw.	39
30	HMSA.tw.	98
31	(Biomonitor* or iLite).tw.	5664
32	(Matriks* Biotek* or Shikari*).tw.	13
33	(Prometheus* or Anser IFX or Anser ADA).tw.	568
34	22 or 23 or 24 or 25 or 26 or 27 or 28 or 29 or 30 or 31 or 32 or 33	113752

For peer review only

35	((monitor* or pharmacokinetic* or measur* or level* or concentration*) adj3 (adalimumab or ADA or infliximab or IFX or Anti-TNF* or Anti-Tumour Necrosis Factor*)).tw.	2016
36	*crohn disease/	34280
37	crohn*.tw.	50039
38	inflammator* bowel* disease*.tw.	41418
39	IBD.tw.	23266
40	36 or 37 or 38 or 39	82551
41	(((monitor* or pharmacokinetic* or measur* or level* or concentration*) adj3 (adalimumab or infliximab or Anti-TNF* or AntiTNF* or Anti-Tumour Necrosis Factor*)) and (correlat* or associat* or test performance)).tw.	544
42	13 and 21 and 40	278
43	13 and 34 and 40	109
44	35 and 40	507
45	41 or 42 or 43 or 44	938
46	nonhuman/ not human/	3490973
47	45 not 46	917

Cochrane Library (Wiley), searched on 22/10/2014

#1	adalimumab:ti,ab,kw	451
#2	ADA:ti,ab	237
#3	infliximab:ti,ab,kw	767
#4	IFX:ti,ab	39
#5	((anti-TNF* or antiTNF* or TNF*) near/2 inhibitor*):ti,ab,kw	106
#6	(anti* next tumo*r* next necrosis* next factor*):ti,ab,kw	256
#7	MeSH descriptor: [Tumor Necrosis Factor-alpha] this term only	2408
#8	MeSH descriptor: [Antibodies, Monoclonal] this term only	3978
#9	#7 and #8	409

For peer review only

#10	(anti* next drug* next antibod*):ti,ab,kw	19
#11	(ADAb):ti,ab,kw	0
#12	#1 or #2 or #3 or #4 or #5 or #6 or #7 or #8 or #9 or #10 or #11	6714
#13	(lisa* next tracker*):ti,ab,kw	0
#14	(immundiagnostik* or immunodiagnostik* or immunediagnostik*):ti,ab,kw	0
#15	(proteomika* or promonitor*):ti,ab,kw	0
#16	MeSH descriptor: [Enzyme-Linked Immunosorbent Assay] explode all trees	2122
#17	(enzyme* next link* next immunoassay*):ti,ab,kw	84
#18	ELISA*:ti,ab,kw	2534
#19	#13 or #14 or #15 or #16 or #17 or #18	3958
#20	MeSH descriptor: [Radioimmunoassay] explode all trees	1176
#21	(radioimmuno* or radio next immuno* or radio-immuno*):ti,ab,kw	2761
#22	RIA:ti,ab	570
#23	(reporter* next gene* next assay*):ti,ab,kw	11
#24	RGA:ti,ab	8
#25	(semi* next fluid* next phase* next enzyme* next immuno*):ti,ab,kw	0
#26	EIA:ti,ab	339
#27	((homogenous* or homogeneous*) near/1 (mobilit* next shift* next assay*)):ti,ab,kw	1
#28	HMSA:ti,ab	1
#29	(Biomonitor* or iLite):ti,ab,kw	14
#30	(Matriks* next Biotek* or Shikari*):ti,ab,kw	0
#31	(Prometheus* or Anser next IFX or Anser next ADA):ti,ab,kw	23
#32	#20 or #21 or #22 or #23 or #24 or #25 or #26 or #27 or #28 or #29 or #30 or #31	3651
#33	((monitor* or pharmacokinetic* or measur* or level* or concentration*) near/3 (adalimumab or ADA or infliximab or IFX or Anti-TNF* or Anti-Tumour next Necrosis next Factor*)):ti,ab,kw	83

For peer review only

#34	MeSH descriptor: [Inflammatory Bowel Diseases] this term only	273
#35	MeSH descriptor: [Crohn Disease] this term only	997
#36	crohn*:ti,ab,kw	1512
#37	(inflammator* next bowel* next disease*):ti,ab,kw	798
#38	IBD:ti,ab	271
#39	#34 or #35 or #36 or #37 or #38	2037
#40	(((monitor* or pharmacokinetic* or measur* or level* or concentration*) near/3 (adalimumab or infliximab or Anti-TNF* or AntiTNF* or Anti-Tumour next Necrosis next Factor*)) and (correlat* or associat* or test next performance)):ti,ab,kw	33
#41	#12 and #19 and #39	8
#42	#12 and #32 and #39	1
#43	#33 and #39	18
#44	#40 or #41 or #42 or #43	49

All Results (49)

Cochrane Reviews (0)

All Review Protocol

Other Reviews (1)

Trials (47)

Methods Studies (0)

Technology Assessments (1)

Economic Evaluations (0)

Cochrane Groups (0)

Science Citation Index and Conference Proceedings – Science (Web of Science), searched on 22/10/2014

# 40	806	#39 OR #38 OR #37 OR #36<br>Indexes=SCI-EXPANDED, CPCI-S Timespan=All years
# 39	324	#35 AND #32<br>Indexes=SCI-EXPANDED, CPCI-S Timespan=All years
# 38	26	#35 AND #31 AND #9

For peer review only

		Indexes=SCI-EXPANDED, CPCI-S Timespan=All years
# 37	128	#35 AND #16 AND #9<br>Indexes=SCI-EXPANDED, CPCI-S Timespan=All years
# 36	539	TS=(((monitor* or pharmacokinetic* or measur* or level* or concentration*) near/3 (adalimumab or ADA or infliximab or IFX or Anti-TNF* or ("Anti-Tumour Necrosis" near/1 Factor*))) and (correlat* or associat* or "test performance"))<br>Indexes=SCI-EXPANDED, CPCI-S Timespan=All years
# 35	80,743	#34 OR #33<br>Indexes=SCI-EXPANDED, CPCI-S Timespan=All years
# 34	53,142	TS=(((inflammator* near/1 bowel*) near/1 disease*) or IBD)<br>Indexes=SCI-EXPANDED, CPCI-S Timespan=All years
# 33	50,398	TS=crohn*<br>Indexes=SCI-EXPANDED, CPCI-S Timespan=All years
# 32	1,366	TS=((monitor* or pharmacokinetic* or measur* or level* or concentration*) near/3 (adalimumab or ADA or infliximab or IFX or Anti-TNF* or ("Anti-Tumour Necrosis" near/1 Factor*)))<br>Indexes=SCI-EXPANDED, CPCI-S Timespan=All years
# 31	79,288	#30 OR #29 OR #28 OR #27 OR #26 OR #25 OR #24 OR #23 OR #22 OR #21 OR #20 OR #19 OR #18 OR #17<br>Indexes=SCI-EXPANDED, CPCI-S Timespan=All years
# 30	713	TS=(Prometheus* or "Anser IFX" or "Anser ADA")<br>Indexes=SCI-EXPANDED, CPCI-S Timespan=All years
# 29	10	TS=((Matriks* near/1 Biotek*) or Shikari*)<br>Indexes=SCI-EXPANDED, CPCI-S Timespan=All years
# 28	8,841	TS=(Biomonitor* or iLite)<br>Indexes=SCI-EXPANDED, CPCI-S Timespan=All years
# 27	107	TS=HMSA<br>Indexes=SCI-EXPANDED, CPCI-S Timespan=All years
# 26	11	TS=((homogenous* or homogeneous*) near/1 (mobilit* near/1 (shift* near/1 assay*)))<br>Indexes=SCI-EXPANDED, CPCI-S Timespan=All years
# 25	8,832	TS=EIA<br>Indexes=SCI-EXPANDED, CPCI-S Timespan=All years
# 24	1	TS=((semi* near/1 fluid*) near/3 (enzyme* near/1 immuno*))<br>Indexes=SCI-EXPANDED, CPCI-S Timespan=All years

For peer review only

# 23	0	TS=((semi* near/1 fluid*) near/2 (enzyme* near/1 immuno*)) Indexes=SCI-EXPANDED, CPCI-S Timespan=All years
# 22	0	TS=(semi* near/1 fluid* near/1 phase* near/1 enzyme* near/1 immuno*) Indexes=SCI-EXPANDED, CPCI-S Timespan=All years
# 21	0	TS=(((semi* near/1 fluid*) near/1 phase*) near/1 (enzyme* near/1 immuno*)) Indexes=SCI-EXPANDED, CPCI-S Timespan=All years
# 20	1,230	TS=RGA Indexes=SCI-EXPANDED, CPCI-S Timespan=All years
# 19	4,518	TS=(reporter* near/1 gene* near/1 assay*) Indexes=SCI-EXPANDED, CPCI-S Timespan=All years
# 18	12,773	TS=RIA Indexes=SCI-EXPANDED, CPCI-S Timespan=All years
# 17	46,937	TS=(radioimmuno* or (radio near/1 immuno*) or radio-immuno*) Indexes=SCI-EXPANDED, CPCI-S Timespan=All years
# 16	146,389	#15 OR #14 OR #13 OR #12 OR #11 OR #10 Indexes=SCI-EXPANDED, CPCI-S Timespan=All years
# 15	113,120	TS=ELISA* Indexes=SCI-EXPANDED, CPCI-S Timespan=All years
# 14	60,666	TS=((enzyme* near/1 link*) near/1 (immuno* near/1 assay)) Indexes=SCI-EXPANDED, CPCI-S Timespan=All years
# 13	2,850	TS=((enzyme* near/1 link*) near/1 immunoassay*) Indexes=SCI-EXPANDED, CPCI-S Timespan=All years
# 12	1	TS=(proteomika* or promonitor*) Indexes=SCI-EXPANDED, CPCI-S Timespan=All years
# 11	9	TS=(immundiagnostik* or immunodiagnostik* or immunediagnostik*) Indexes=SCI-EXPANDED, CPCI-S Timespan=All years
# 10	0	TS=(lisa* near/1 tracker*) Indexes=SCI-EXPANDED, CPCI-S Timespan=All years
# 9	32,262	#8 OR #7 OR #6 OR #5 OR #4 OR #3 OR #2 OR #1 Indexes=SCI-EXPANDED, CPCI-S Timespan=All years
# 8	35	TS=ADAb Indexes=SCI-EXPANDED, CPCI-S Timespan=All years
# 7	2,534	TS=((anti* near/1 drug*) near/1 antibod*) Indexes=SCI-EXPANDED, CPCI-S Timespan=All years

For peer review only

# 6	4,072	TS=((anti* near/1 tumo$r*) near/1 (necrosis* near/1 factor*))<br>Indexes=SCI-EXPANDED, CPCI-S Timespan=All years
# 5	4,065	TS=((anti-TNF* or antiTNF* or TNF*) near/2 inhibitor*)<br>Indexes=SCI-EXPANDED, CPCI-S Timespan=All years
# 4	373	TS=IFX<br>Indexes=SCI-EXPANDED, CPCI-S Timespan=All years
# 3	13,729	TS=infliximab<br>Indexes=SCI-EXPANDED, CPCI-S Timespan=All years
# 2	8,006	TS=ADA<br>Indexes=SCI-EXPANDED, CPCI-S Timespan=All years
# 1	4,973	TS=adalimumab<br>Indexes=SCI-EXPANDED, CPCI-S Timespan=All years

Index to Theses, searched on 28/10/2014

((adalimumab or infliximab or AntiTNF* or Anti-TNF* or "Anti TNF" or "Anti TNFa" or "Anti TNFalpha" or (TNF* w/2 inhibitor*) or (Anti-Tum*r w/2 Necrosis) or ("anti drug" w/2 antibod*) or ADAb) AND (crohn* or "inflammatory bowel disease" or IBD))

14 document(s) retrieved

(((adalimumab or infliximab or AntiTNF* or Anti-TNF* or "Anti TNF" or "Anti TNFa" or "Anti TNFalpha" or (TNF* w/2 inhibitor*) or (Anti-Tum*r w/2 Necrosis) or "anti drug antibody" or "anti drug antibodies" or "anti-drug antibody" or "anti-drug antibodies" or ADAb) w/10 (monitor or monitoring or monitors or monitored or pharmacokinetic or pharmacokinetics or measure or measures or measurement or measuring or level or levels or concentration or concentrations)) AND ((correlate* or correlation* or associate* or association* or "test performance")))

4 document(s) retrieved

DART-Europe, searched on 28/10/2014

(adalimumab or infliximab or AntiTNF* or Anti-TNF* or "Anti TNF" or "Anti TNFa" or "Anti TNFalpha" or (TNF* and inhibitor*) or (Anti-Tum*r and Necrosis) or ("anti drug" and antibod*) or ADAb) and (crohn* or "inflammatory bowel disease" or "inflammatory bowel diseases" or IBD)

113 document(s) retrieved

Dissertations and Theses, searched on 29/10/2014

all(((adalimumab or infliximab or AntiTNF* or Anti-TNF* or "Anti TNF" or "Anti TNFa" or "Anti TNFalpha" or (TNF* n/2 inhibitor*) or (Anti-Tum*r n/2 Necrosis) or ("anti drug" n/2 antibod*) or ADAb) AND (crohn* or "inflammatory bowel disease" or "inflammatory bowel diseases" or IBD)))

21

For peer review only

all(((adalimumab or infliximab or AntiTNF* or Anti-TNF* or "Anti TNF" or "Anti TNFa" or "Anti TNFalpha" or (TNF* n/2 inhibitor*) or (Anti-Tum*r n/2 Necrosis) or "anti drug antibody" or "anti drug antibodies" or "anti-drug antibody" or "anti-drug antibodies" or ADAb) n/10 (monitor or monitoring or monitors or monitored or pharmacokinetic or pharmacokinetics or measure or measures or measurement or measuring or level or levels or concentration or concentrations)) and (correlate* or correlation* or associate* or association* or "test performance"))

15

NIHR HTA Programme, searched on 29/10/2014

adalimumab

16

infliximab

23

TNF

17

PROSPERO, searched on 29/10/2014

adalimumab in All fields

OR

infliximab in All fields

OR

TNF* inhibitor* in All fields

OR

AntiTNF* in All fields

OR

Anti-TNF* in All fields

29 records

ClinicalTrials.gov, searched on 04/11/2014

Search Terms (any field): adalimumab OR infliximab OR (TNF AND (anti OR inhibitor OR blocker)) OR "anti drug antibody" OR "anti drug antibodies" OR ADAb

AND

Condition: crohn OR "inflammatory bowel disease" OR "inflammatory bowel diseases"

AND

Title: monitor OR pharmacokinetic OR measure OR measuring OR level OR concentration OR assay

14 studies

For peer review only

Current Controlled Trials, searched on 04/11/2014

(adalimumab OR infliximab OR TNF* OR AntiTNF* OR Anti-TNF* OR anti drug antibod* OR ADAb) AND (crohn* OR inflammatory bowel disease*) AND (monitor* OR pharmacokinetic* OR measure* OR measuring OR level* OR concentration* OR assay*)

30 studies

UKCRN Portfolio Database, searched on 04/11/2014

Specialty: Gastroenterology

Research Summary: adalimumab infliximab TNF AntiTNF Anti-TNF ADAb

'Any' selected (combines terms with Boolean OR)

4 studies

WHO ICTRP, searched on 10/11/2014

Advanced Search

In Title: adalimumab OR infliximab OR AntiTNF* OR Anti-TNF* OR TNF inhibitor* OR TNFα inhibitor* OR TNF alpha inhibitor* OR TNFalpha inhibitor* OR anti drug antibody OR anti drug antibodies OR ADAb

AND

In Condition: Crohn* OR inflammatory bowel disease*

AND

In Intervention: monitor* OR pharmacokinetic* OR measure* OR measuring OR level* OR concentration* OR assay*

39 trials found

For peer review only

Supplement 2 Update search for new studies

Supplement 2 Figure 1 summarises the search update undertaken to identify new studies. There were 140 citations to the systematic reviews of Nanda et al. 2013 and Paul et al. 2014.[6 7]. Amongst these there were three recent systematic reviews,[48-50] which in turn yielded a further six unique citations. Within the three recent systematic reviews there were 35 unique primary studies. We screened all citations to the systematic reviews and all studies included in the new systematic reviews. [48-50]

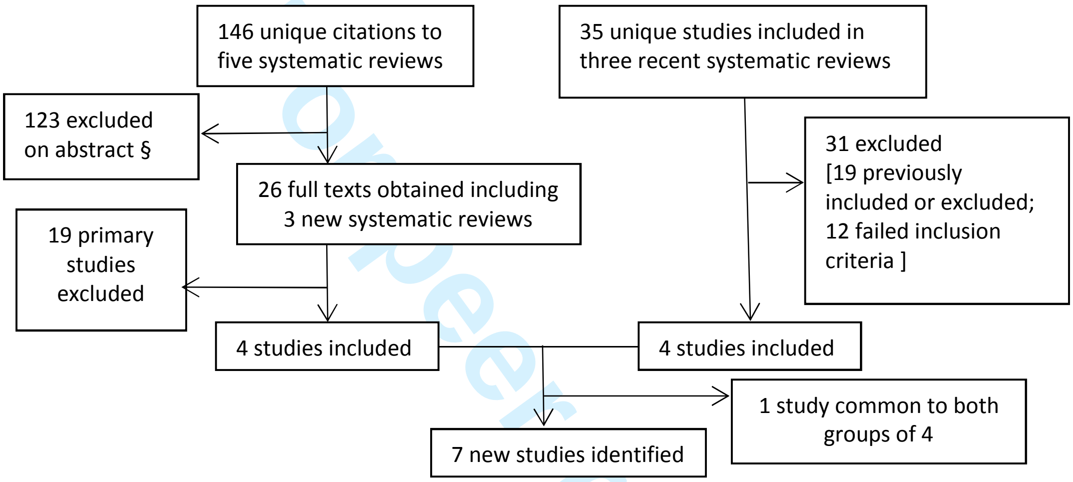

Supplement 2 Figure 1 Study flow diagram. *(Excluded studies are identified in Supplement 2 Table 1)*

Seven new studies satisfied our inclusion criteria, their main characteristics are summarised in Supplement 8 Table 1.

Supplement 2 Table 1 List of excluded studies with reasons for exclusion

Studies excluded from those included in three recent systematic reviews			Reason for exclusion
1a	Adedokuun 2014	Gastroenterology. 2014;147:1296–1307.e5.	all UC patients
2a	Ainsworth 2008	Am J Gastroenterol 2008;103(4):944-8	already included or excluded
3a	Baert 2014	Clin Gastroenterol Hepatol 2014;12(9):1474-81.e2	already included or excluded
4a	Ben-Basset 2013	Gastroenterology 2013;144(5 Suppl):S-775	already included or excluded
5a	Bortlik 2013	Journal of Crohn's & colitis 2013;7(9):736-43	already included or excluded
6a	Vande Casteele 2015	Gastroenterology 2015;148:1320–9.e3.	already included or excluded
7a	Vande Casteele 2014	Gut. 2015;64:1539–1545.	2x2 table not possible
8a	Vande Casteele 2013	Am J Gastroenterol.2013; 108:962–971.	already included or excluded
9a	Colombel 2014	Clin Gastroenterol Hepatol 12, 423	wrong drug

For peer review only

10a	Cornillie 2014	Gut 2011;60:A296.	already included or excluded
11a	Daperno 2013	Gastroenterology 2013;144:Tu1173.	too few CD patients
12a	Drastich 2011	Gastroenterology 2011;140:S292.	already included or excluded
13a	Drobne 2015	Clin Gastroenterol Hepatol 2015;13:514–21.e4.	2x2 table not possible
14a	Echarri 2015	J Crohns Colitis. 2015;9:S342–aS343.	2x2 table not possible
15a	Hibi 2014	J Gastroenterol 2014;49:254–62.	already included or excluded
16a	Imaeda 2014	J Gastroenterol.2014;49:100–109.	already included or excluded
17a	Imaeda 2014	J Gasroenterology 49;674-682	2x2 table not possible
18a	Marits 2014	J Crohns Colitis. 2014;8:881–889.	2x2 table not possible
19a	Maser 2006	Clin Gastroenterol Hepatol 2006;4(10):1248-54	already included or excluded
20a	Mazor 2014	Aliment Pharmacol Ther. 2014;40:620–628.	already included or excluded
21a	Murthy 2012	Gastroenterology 2012;142:S388.	all UC patients
22a	Papamichail 2015	Gastroenterology. 2015;148: S848.	all UC patients
23a	Pariente 2012	Inflamm Bowel Dis 2012;18:1199–206.	already included or excluded
24a	Paul 2013	Inflamm Bowel Dis 2013;19:2568–76.	too few CD patients
25a	Roblin 2014	Clin Gastroenterol Hepatol. 2014;12:80–84.e2.	already included or excluded
26a	Roblin 2015	Drug Levels & Biomarkers. 2015;148:S–853.	2x2 table not possible
27a	Ron 2012	Gastroenterology 2012;142:S385.	2x2 table not possible
28a	Seow 2010	Gut 2010;59:49–54	all UC patients
29a	Singh 2014	Inflamm Bowel Dis. 2014;20:1708–1713.	already included or excluded
30a	Steenholdt 2011	Scand J Gastroenterol 2011;46:310–8.	already included or excluded
31a	Tang 2014	J Crohns Colitis. 2014;8:S209–S210.	already included or excluded
**Studies excluded from citations to five systematic reviews**			**Reason for exclusion**
1	Vande Casteele 2013	American Journal of Gastroenterology 108(6): 962-971	See 8a
2	Bodini 2014	Digestive and Liver Disease 46(11): 1043-1046.	already included or excluded
3	Imaeda 2014	Journal of Gastroenterology 49(4): 674-682	See 17a
4	Marits 2014	Journal of Crohn's and Colitis 8(8): 881-889	See 18a
5	Pallagi-Kunstár 2014	World Journal of	already included or excluded

For peer review only

		Gastroenterology 20(17): 5031-5035	
6	Rivero Marcotegui 2014	Revista del Laboratorio Clinico 7(2): 68-72	already included or excluded
7	Roblin 2014	Clinical Gastroenterology and Hepatology 12(1): 80-84.e82	See 25a
8	Singh 2014	Inflammatory Bowel Diseases 20(10): 1708-1713	See 29a
9	Steenholdt 2014	American Journal of Gastroenterology 109(7): 1055-1064	already included or excluded
10	Steenholdt 2014	Gut 63(6): 919-927	already included or excluded
11	Ungar	Gut 63(8): 1258-1264	already included or excluded
12	Vaughn 2014	Inflammatory Bowel Diseases 20(11): 1996-2003	already included or excluded
13	Vande Casteele 2015	Gut 64(10): 1539-1545	2x2 table not possible
14	Roblin 2015	Journal of Crohn's and Colitis 9(7): 525-531	too few CD patients
15	Van Stappen 2015	Inflammatory Bowel Diseases 21(9): 2172-2177	2x2 table not possible
16	Warman 2015	European Journal of Gastroenterology and Hepatology 27(3): 242-248	too few CD patients
17	Yanai 2015	Clinical Gastroenterology and Hepatology 13(3): 522-530	2x2 table not possible
18	Yarur 2015	Clinical Gastroenterology and Hepatology 13(6): 1118-1124.e1113	too few CD patients
19	Bodini 2016	Scandinavian Journal of Gastroenterology 51(9): 1081-1086	2x2 table not possible

For peer review only

Supplement 3 Funnel plots and tests for publication bias

In the meta-analysis of tests for trough Infliximab levels using funnel plots and Harbord's and Peter's tests for small study bias in diagnostic odds ratios[1, 2] we found no evidence of small study bias in diagnostic odds ratios: Harbord test p = 0.312, Peters test p = 0.576. The corresponding values for tests of antibodies against Infliximab were p = 0.734 and p = 0.780.

Antibodies to Infliximab

1] Funnel plot

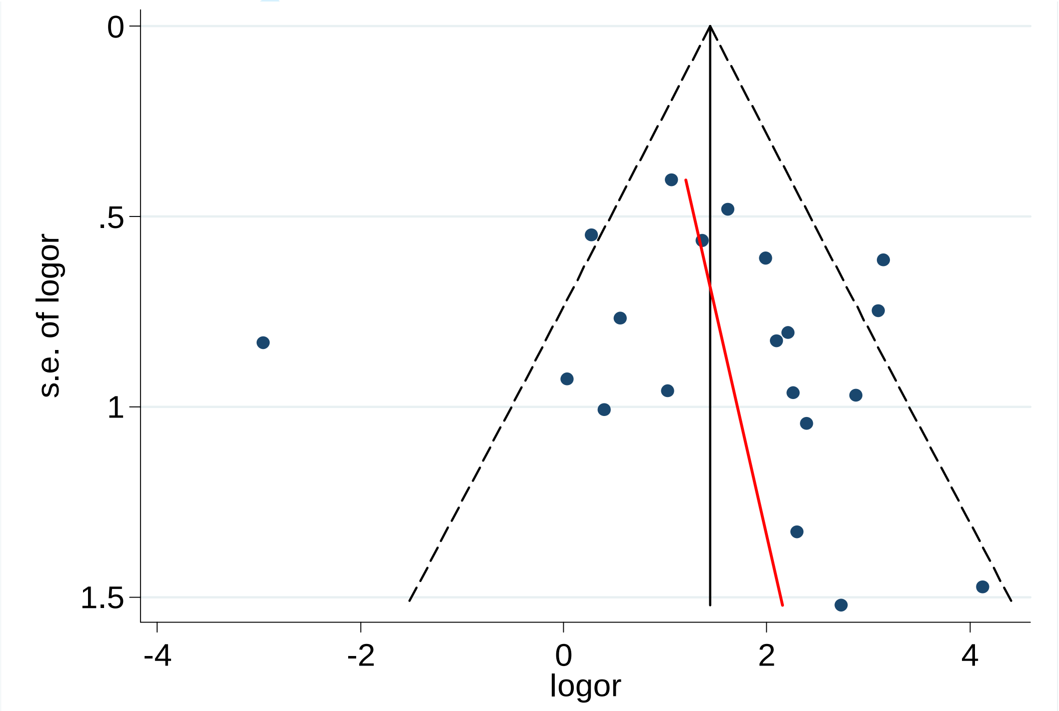

2] Egger's test for small-study effects:

Number of studies = 20

Eggers test

slope | .8614847 .8816692 0.98 0.341 -.9908337 2.713803

bias | .8517858 1.21317 0.70 0.492 -1.69699 3.400561

Test of H0: no small-study effects P = 0.492

Does not support publication bias.

1. Harbord R, Harris RJ, Sterne JAC. Updated tests for small-study effects in meta-analyses. Stata Journal 2009;9(2):197-210

2. Macaskill P, Gatsonis C, Deeks J, et al. Chapter 10: Analysing and Presenting Results. In: Deeks JJ, Bossuyt PM, Gatsonis C, eds. Cochrane Handbook for Systematic Reviews of Diagnostic Test Accuracy Version 10: The Cochrane Collaboration, 2010.

3] Harbord plot

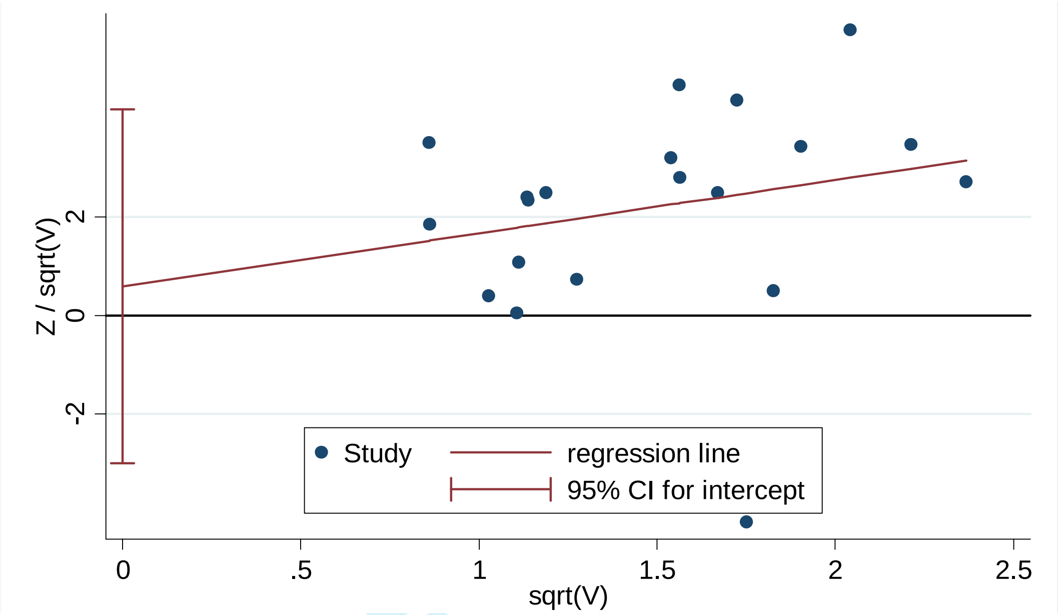

4] Harbord's modified test for small-study effects:

```
Number of studies = 20              Root MSE   = 2.125
 Z/sqrt(V) |   Coef.  Std. Err.   t  P>|t|   [95% Conf. Interval]
-------------+----------------------------------------------------------------
   sqrt(V) |  1.079732  1.099815   0.98  0.339  -1.230893  3.390356
     bias |  .5901862  1.710314   0.35  0.734  -3.003051  4.183424
------------------------------------------------------------------------------
Test of H0: no small-study effects      P = 0.734
```

5] Peter's test for small-study effects:

```
Number of studies = 18              Root MSE   =  1.459
   Std_Eff |   Coef.  Std. Err.   t  P>|t|   [95% Conf. Interval]
     bias | -8.626685  30.41227  -0.28  0.780  -73.09781  55.84444
  constant |  1.674552  .6008762   2.79  0.013   .400751  2.948352
Test of H0: no small-study effects      P = 0.780
```

Peer review only

Trough Infliximab tests

1] Funnel plot

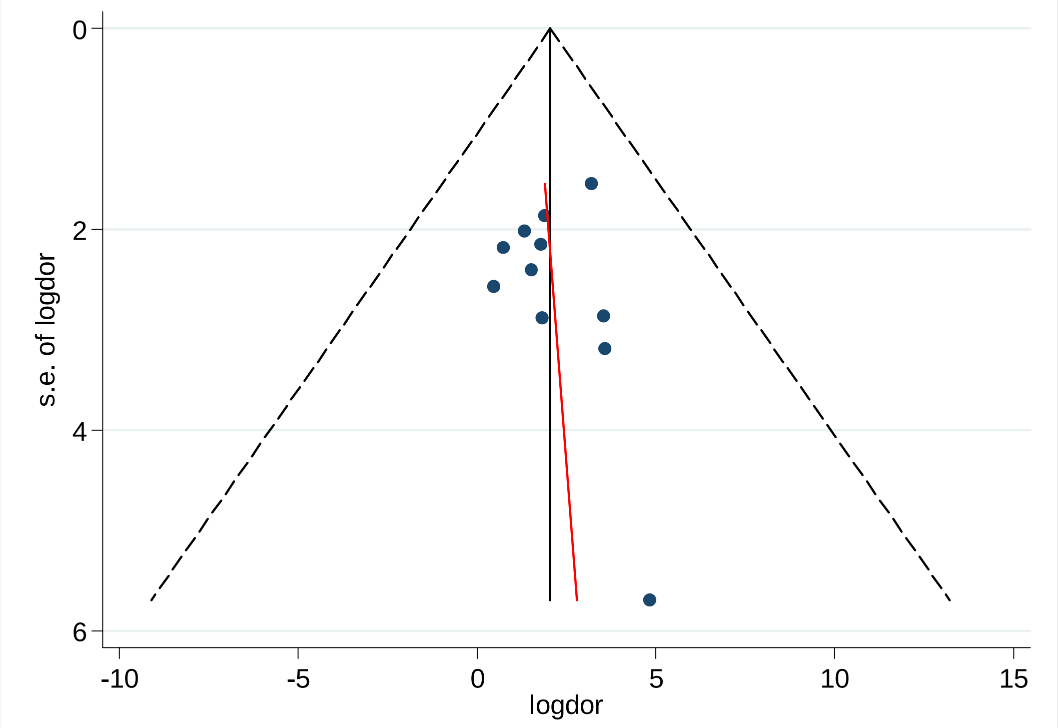

2] Egger's test for small-study effects:
Regress standard normal deviate of intervention
effect estimate against its standard error

Number of studies = 11 Root MSE = 1.907

Std_Eff	Coef.	Std. Err.	t	P>|t|	[95% Conf. Interval]	
slope	1.580826	1.251978	1.26	0.238	-1.251345	4.412998
bias	.8249369	2.088696	0.39	0.702	-3.900021	5.549894

Test of H0: no small-study effects P = 0.702

review only

3] Harbord plot

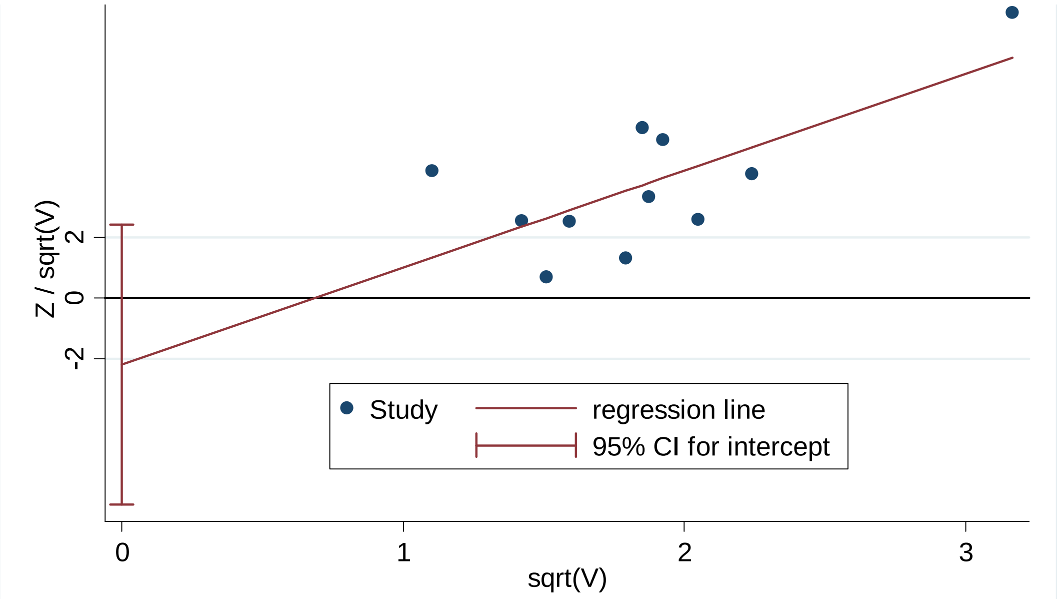

4] Harbord's modified test for small-study effects:
Regress Z/sqrt(V) on sqrt(V) where Z is efficient score and V is score variance
Number of studies = 11 Root MSE = 1.779
Test of H0: no small-study effects P = 0.312

5] Peter's test for small-study effects:
Regress intervention effect estimate on 1/Ntot, with weights S×F/Ntot
Number of studies = 11 Root MSE = 1.191

Std_Eff	Coef.	Std. Err.	t	P>|t|	[95% Conf. Interval]	
bias	-28.29877	48.81199	-0.58	0.576	-138.7192	82.12163
constant	2.738445	.725501	3.77	0.004	1.097248	4.379642

Test of H0: no small-study effects P = 0.576

For peer review only

Supplement 4 Excluded studies with reason

Supplement 4 Table 1 Full text exclusions with reason

Reference	Reason for exclusion
1. Afif, W., E. V. Loftus, Jr., W. A. Faubion, S. V. Kane, D. H. Bruining, K. A. Hanson and W. J. Sandborn (2010). "Clinical utility of measuring infliximab and human anti-chimeric antibody concentrations in patients with inflammatory bowel disease." American Journal of Gastroenterology 105(5): 1133-1139.	Insufficient data
2. Baert, F., M. Noman, S. Vermeire, G. Van Assche, D. H. G, A. Carbonez and P. Rutgeerts (2003). "Influence of immunogenicity on the long-term efficacy of infliximab in Crohn's disease." N Engl J Med 348(7): 601-608.	Insufficient data
3. Balzola, F., C. Bernstein, G. T. Ho and C. Lees (2010). "Clinical utility of measuring infliximab and human antichimeric antibody concentrations in patients with inflammatory bowel disease: Commentary." Inflammatory Bowel Disease Monitor 11(2): 85-86.	Commentary no original data
4. Balzola, F., G. Cullen, G. T. Ho and R. K. Russell (2013). "Clinical utility of newly developed immunoassays for serum concentrations of adalimumab and anti-adalimumab antibodies in patients with Crohn's disease." Inflammatory Bowel Disease Monitor 14(1): 19.	Commentary no original data
5. Ben-Horin, S. and Y. Chowers (2011). "Review article: loss of response to anti-TNF treatments in Crohn's disease." Aliment Pharmacol Ther 33(9): 987-995.	Review without MA
6. Billioud, V., W. J. Sandborn and L. Peyrin-Biroulet (2011). "Loss of response and need for adalimumab dose intensification in Crohn's disease: a systematic review." American Journal of Gastroenterology 106(4): 674-684.	SR without MA
7. Cassinotti A, Travis S. Incidence and clinicalsignificance of immunogenicity to infliximab inCrohn's disease: a critical systematic review. Inflamm Bowel Dis. 2009;15(8):1264-75.	Review without MA
8. Chaparro, M., I. Guerra, P. Munoz-Linares and J. P. Gisbert (2012). "Systematic review: antibodies and anti-TNF-alpha levels in inflammatory bowel disease." Aliment Pharmacol Ther 35(9): 971-986.	SR without MA
9. Colombel JF, Feagan BG, Sandborn WJ, Van Assche G, Robinson AM. Therapeutic drugmonitoring of biologics for inflammatory bowel disease. 2012;18(2):349-58.	Review without MA
10. Corstjens PL, Fidder HH, Wiesmeijer KC, et al. A rapid assay for on-site monitoring of infliximab trough levels: a feasibility study. Anal Bioanal Chem 2013;405(23):7367-75 doi: http://dx.doi.org/10.1007/s00216-013-7154-0[published Online First: Epub Date]|.	Insufficient data
11. Ebert, E. C., K. M. Das, V. Mehta and C. Rezac (2008). "Non-response to infliximab may be due to innate neutralizing anti-tumour necrosis factor-alpha antibodies." Clinical & Experimental Immunology 154(3): 325-331.	Measurement of antibodies to TNF-alpha not anti-TNFα drugs
12. Garces, S., J. Demengeot and E. Benito-Garcia (2013). "The immunogenicity of anti-TNF therapy in immune-mediated inflammatory diseases: a systematic review of the literature with a meta-analysis." Annals of the Rheumatic Diseases 72(12): 1947-1955.	>50% RA patients

For peer review only

13. Hamalainen, A., T. Sipponen and K. L. Kolho (2013). "Serum infliximab concentrations in pediatric inflammatory bowel disease." Scandinavian Journal of Gastroenterology 48(1): 35-41.	Insufficient data
14. Hibi, T., A. Sakuraba, M. Watanabe, S. Motoya, H. Ito, K. Motegi, Y. Kinouchi, M. Takazoe, Y. Suzuki, T. Matsumoto, K. Kawakami, T. Matsumoto, I. Hirata, S. Tanaka, T. Ashida and T. Matsui (2012). "Retrieval of serum infliximab level by shortening the maintenance infusion interval is correlated with clinical efficacy in Crohn's disease." Inflamm Bowel Dis 18(8): 1480-1487.	Insufficient data
15. Imaeda H, Bamba S, Takahashi K, et al. Relationship between serum infliximab trough levels and endoscopic activities in patients with Crohn's disease under scheduled maintenance treatment. J Gastroenterol 2014;49(4):674-82 doi: http://dx.doi.org/10.1007/s00535-013-0829-7[published Online First: Epub Date]|.	Insufficient data
16. Karmiris K, Paintaud G, Noman M, et al. Influence of trough serum levels and immunogenicity on long-term outcome of adalimumab therapy in Crohn's disease. Gastroenterology 2009;137(5):1628-40 doi: http://dx.doi.org/10.1053/j.gastro.2009.07.062[published Online First: Epub Date]|.	Insufficient data
17. Khanna, R., B. D. Sattin, W. Afif, E. I. Benchimol, E. J. Bernard, A. Bitton, B. Bressler, R. N. Fedorak, S. Ghosh, G. R. Greenberg, J. K. Marshall, R. Panaccione, E. G. Seidman, M. S. Silverberg, A. H. Steinhart, R. Sy, G. Van Assche, T. D. Walters, W. J. Sandborn and B. G. Feagan (2013). "Review article: a clinician's guide for therapeutic drug monitoring of infliximab in inflammatory bowel disease." Aliment Pharmacol Ther 38(5): 447-459.	SR without MA
18. Lazebnik, L. B. and V. E. Sagynbaeva (2013). "[Level of adalimumab and its antibody titers define the effectiveness of the biological (anticytokine) therapy in Crohn's disease]." Eksperimental'Naia i Klinicheskaia Gastroenterologiia(7): 18-22.	Non-English
19. Levesque BG, Greenberg GR, Zou G, et al. A prospective cohort study to determine the relationship between serum infliximab concentration and efficacy in patients with luminal Crohn's disease. Aliment Pharmacol Ther 2014;39(10):1126-35 doi: http://dx.doi.org/10.1111/apt.12733[published Online First: Epub Date]|.	Insufficient data
20. Lichtenstein, G. R. (2013). "Comprehensive review: antitumor necrosis factor agents in inflammatory bowel disease and factors implicated in treatment response." Therapeutic Advances in Gastroenterology 6(4): 269-293.	SR without MA
21. Malickova, K., D. Duricova, M. Bortlik, N. Machkova, I. Janatkova and M. Lukas (2011). "Serum infliximab trough levels and induction of antibodies to infliximab during the biological treatment of patients with inflammatory bowel diseases. [Czech]Serove hladiny infliximabu a indukce tvorby protilatek proti infliximabu pri biologicke lecbe nemocnych s idiopatickymi strevnimi zanety." Alergie 13(3): 216-222.	Non-English
22. Marits P, Landucci L, Sundin U, et al. Trough s-infliximab and antibodies towards infliximab in a cohort of 79 IBD patients with maintenance infliximab treatment. Journal of Crohn's & colitis 2014;8(8):881-9 doi: http://dx.doi.org/10.1016/j.crohns.2014.01.009[published Online First: Epub Date]|.	Insufficient data
23. Pallagi-Kunstar E, Farkas K, Szepes Z, et al. Utility of serum TNF-alpha, infliximab trough level, and antibody titers in inflammatory bowel disease. World J Gastroenterol 2014;20(17):5031-5 doi: http://dx.doi.org/10.3748/wjg.v20.i17.5031[published Online First: Epub Date]|.	Insufficient data
24. Paul S, Del Tedesco E, Marotte H, et al. Therapeutic drug monitoring of infliximab and mucosal healing in inflammatory bowel disease: a prospective study. Inflamm Bowel Dis 2013;19(12):2568-76 doi: http://dx.doi.org/10.1097/MIB.0b013e3182a77b41[published Online First: Epub Date]|.	Insufficient data
25. Rivero Marcotegui, A., R. Ibanez Bosch, A. Zuniga Vera, A. Arin Letamendia and M. J. Burusco Paternain (2014). "Clinical usefulness in measuring infliximab and human anti-chimeric antibodies. [Spanish]Utilidad clinica de la cuantificacion de infliximab y anticuerpos antiquimericos humanos." Revista del	patients >50% RA

For peer review only

Laboratorio Clinico 7(2): 68-72.	
26. Roblin, X., M. Rinaudo, E. Del Tedesco, J. M. Phelip, C. Genin, L. Peyrin-Biroulet and S. Paul (2014). "Development of an algorithm incorporating pharmacokinetics of adalimumab in inflammatory bowel diseases." American Journal of Gastroenterology 109(8): 1250-1256.	Insufficient data
27. Ruiz-Arguello B, del Agua AR, Torres N, et al. Comparison study of two commercially available methods for the determination of infliximab, adalimumab, etanercept and anti-drug antibody levels. Clin Chem Lab Med 2013;51(12):e287-9 doi: 10.1515/cclm-2013-0461[published Online First: Epub Date]|.	Insufficient data
28. Rutgeerts, P., G. D'Haens, S. Targan, E. Vasiliauskas, S. B. Hanauer, D. H. Present, L. Mayer, R. A. Van Hogezand, T. Braakman, K. L. DeWoody, T. F. Schaible and S. J. Van Deventer (1999). "Efficacy and safety of retreatment with anti-tumor necrosis factor antibody (infliximab) to maintain remission in Crohn's disease." Gastroenterology 117(4): 761-769.	Insufficient data
29. Schatz SB, Prell C, Freudenberg F, et al. PA-G-0035 Comparison of different tests for determination of infliximab levels and antibodies against infliximab in pediatric IBD patients. The 46th Annual Meeting of The European Society of Paediatric Gastroenterology, Hepatology and Nutrition. J Pediatr Gastroenterol Nutr 2013;56 suppl 2:19	Insufficient data
30. Singh N, Rosenthal CJ, Melmed GY, et al. Early infliximab trough levels are associated with persistent remission in pediatric patients with inflammatory bowel disease. Inflamm Bowel Dis 2014;20(10):1708-13 doi: http://dx.doi.org/10.1097/MIB.0000000000000137[published Online First: Epub Date]|.	Insufficient data
31. Sono, K., A. Yamada, Y. Yoshimatsu, N. Takada and Y. Suzuki (2012). "Factors associated with the loss of response to infliximab in patients with Crohn's disease." Cytokine 59(2): 410-416.	Insufficient data
32. Steenholdt C, Ainsworth MA, Tovey M, et al. Comparison of techniques for monitoring infliximab and antibodies against infliximab in Crohn's disease. Ther Drug Monit 2013;35(4):530-8 doi: http://dx.doi.org/10.1097/FTD.0b013e31828d23c3[published Online First: Epub Date]|.	Insufficient data
33. Steenholdt C, Bendtzen K, Brynskov J, et al. Clinical implications of measuring drug and anti-drug antibodies by different assays when optimizing infliximab treatment failure in Crohn's disease: post hoc analysis of a randomized controlled trial. Am J Gastroenterol 2014;109(7):1055-64 doi: http://dx.doi.org/10.1038/ajg.2014.106[published Online First: Epub Date]|.	Insufficient data
34. Steenholdt C BJ, Thomsen OØ, Munck LK, Fallingborg J, Christensen LA, Pedersen G, Kjeldsen J, Jacobsen BA, Oxholm AS, Kjellberg J, Bendtzen K, Ainsworth MA. Individualized therapy is a Long-Term Cost-Effective Method Compared to Dose Intensification in Crohn's Disease Patients Failing Infliximab. Dig Dis Sci 2015; Published Online First on 12 Feb 2015. doi:10.1007/s10620-015-3581-4 doi: 10.1007/s10620-015-3581-4[published Online First: Epub Date]|.	Insufficient data
35. Steenholdt, C., M. Svenson, K. Bendtzen, O. O. Thomsen, J. Brynskov and M. A. Ainsworth (2011). "Severe infusion reactions to infliximab: aetiology, immunogenicity and risk factors in patients with inflammatory bowel disease." Aliment Pharmacol Ther 34(1): 51-58.	Insufficient data
36. Ungar, B., Y. Chowers, M. Yavzori, O. Picard, E. Fudim, O. Har-Noy, U. Kopylov, R. Eliakim, S. Ben-Horin and A. consortium (2014). "The temporal evolution of antidrug antibodies in patients with inflammatory bowel disease treated with infliximab." Gut 63(8): 1258-1264.	Insufficient data
37. Van Assche, G., C. Magdelaine-Beuzelin, G. D'Haens, F. Baert, M. Noman, S. Vermeire, D. Ternant, H. Watier, G. Paintaud and P. Rutgeerts (2008). "Withdrawal of immunosuppression in Crohn's disease treated with scheduled infliximab maintenance: a randomized trial." Gastroenterology 134(7): 1861-1868.	Insufficient data

For peer review only

38. Vande Casteele N, Buurman DJ, Sturkenboom MG, et al. Detection of infliximab levels and anti-infliximab antibodies: a comparison of three different assays. Aliment Pharmacol Ther 2012;36(8):765-71 doi: http://dx.doi.org/10.1111/apt.12030[published Online First: Epub Date]|.	Insufficient data
39. Vande Casteele N, Ferrante M, Van Assche G, et al. Trough Concentrations of Infliximab Guide Dosing for Patients with Inflammatory Bowel Disease. Gastroenterology Forthcoming 2015 doi: 10.1053/j.gastro.2015.02.031[published Online First: Epub Date]|.	Insufficient data
40. Vaughn BP, Martinez-Vazquez M, Patwardhan VR, et al. Proactive therapeutic concentration monitoring of infliximab may improve outcomes for patients with inflammatory bowel disease: results from a pilot observational study. Inflamm Bowel Dis 2014;20(11):1996-2003 doi: http://dx.doi.org/10.1097/MIB.0000000000000156[published Online First: Epub Date]|.	Insufficient data
41. Vermeire, S., M. Noman, G. Van Assche, F. Baert, G. D'Haens and P. Rutgeerts (2007). "Effectiveness of concomitant immunosuppressive therapy in suppressing the formation of antibodies to infliximab in Crohn's disease." Gut 56(9): 1226-1231.	Insufficient data
42. Wang SL, Ohrmund L, Hauenstein S, et al. Development and validation of a homogeneous mobility shift assay for the measurement of infliximab and antibodies-to-infliximab levels in patient serum. J Immunol Methods 2012;382(1-2):177-88 doi: http://dx.doi.org/10.1016/j.jim.2012.06.002[published Online First: Epub Date]|.	Insufficient data
43. Yamada, A., K. Sono, N. Hosoe, N. Takada and Y. Suzuki (2010). "Monitoring functional serum antitumor necrosis factor antibody level in Crohn's disease patients who maintained and those who lost response to anti-TNF." Inflamm Bowel Dis 16(11): 1898-1904.	Insufficient data
44. Yanai H, Hanauer SB. Assessing response and loss of response to biological therapies in IBD. Am JGastroenterol. 2011;106(4):685-98	Review without MA

Supplement 4 Table 2 Excluded abstracts with reason

**Reference**	**Reason for exclusion**
45. Abraham, B. and M. Chiorean (2012). "False positive infliximab levels detected in patients treated with adalimumab for inflammatory bowel disease." American Journal of Gastroenterology 107: S627	Insufficient data
46. Afif, W., E. V. Loftus, W. A. Faubion, K. A. Hanson and W. J. Sandborn (2009). "Clinical utility of measuring infliximab and human anti-chimeric antibody levels in patients with inflammatory bowel disease." Gastroenterology 1): A147.	Superseded by full text
47. Anonymous (2012). "New Assay Can Detect Infliximab Levels and Anti-Infliximab Antibodies From a Single Serum Sample." Clinical Advances in Hematology and Oncology 10 (10): 27.	Editorial no original data
48. Armbruster, S., M. Ally, C. Maydonovitch, J. Betteridge and G. Veerappan (2012). "The use of human anti-chimeric antibody (HACA) and infliximab levels in the management of inflammatory bowel disease." American Journal of Gastroenterology 107: S641.	Insufficient data
49. Arranz, M. D. M., E. M. Arranz, D. P. Salcedo, C. De Diego, S. G. Senent, J. P. Cordon, B. B. Garcia and J. M. S. Parga (2014). "Infliximab trough levels and antibodies: Relationship with infusion reaction, immunomodulators and biological parameters." Gastroenterology 1): S-243.	Insufficient data
50. Baert, F. J., D. Drobne, V. Ballet, I. Cleynen, G. Compernolle, P. J. Rutgeerts, G. A. Van Assche, A. Gils and S. Vermeire (2011). "Early trough	Insufficient data

For peer review only

	levels and antibodies predict safety and success of restarting infliximab after long drug holiday." Gastroenterology 1): S62.	
51.	Baert, F. J., S. Lockton, S. Hauenstein, S. Singh, A. Gils and S. Vermeire (2014). "Antibodies to adalimumab predict inflammation in crohn's patients on maintenance adalimumab therapy." Gastroenterology 1): S-242	Insufficient data
52.	Ben-Bassat, O., S. Hauenstein, A. Iacono, S. P. Irwin, S. Singh and G. R. Greenberg (2013). "Serum adalimumab and immunogenicity in IBD patients after 80mg biweekly maintenance therapy." Gastroenterology 1): S771.	Insufficient data
53.	Ben-Horin, S., B. Ungar, Y. Chowers, M. Yavzori, O. Picard, E. Fudim and R. Eliakim (2013). "The temporal evolution of anti-drug antibodies in IBD patients treated with infliximab." Journal of Gastroenterology and Hepatology 28: 145.	Insufficient data
54.	Bodini, G., V. Savarino, P. Dulbecco, I. Baldissarro and E. Savarino (2014). "TNF-alpha levels strongly correlated with disease activity based on HBI and CDEIS in patients with crohn's disease in maintenance treatment with adalimumab." Gastroenterology 1): S-238.	Insufficient data
55.	Bodini, G., V. Savarino, P. Dulbecco, I. Baldissarro and E. Savarino (2014). "The influence of anti-adalimumab antibodies on adalimumab trough levels, TNF-alpha levels and clinical outcome." Journal of Crohn's and Colitis 8: S42.	Insufficient data
56.	Bodini, G., V. Savarino, P. Dulbecco, I. Baldissarro and E. V. Savarino (2014). "Elisa vs. HMSA: A comparison between two different methods for measuring adalimumab serum concentration and anti-adalimumab antibodies-preliminary data." Digestive and Liver Disease 46: S67.	Duplicate
57.	Bodini, G., V. Savarino, P. Dulbecco, L. Assandri, L. Bruzzone, F. Mazza, V. Fazio, E. Giambruno, L. Gemignani and E. Savarino (2013). "Correlation between adalimumab trough serum concentration, anti-adalimumab antibodies and TNF-alpha levels with clinical outcome in patients affected by crohn's disease." Gastroenterology 1): S780.	Insufficient data
58.	Bodini, G., V. Savarino, V. Fazio, L. Assandri, L. Gemignani, P. Dulbecco, E. Giambruno and E. Savarino (2012). "Relationship between drug serum concentration and clinical activity in patients with Crohn's Disease who achieved remission with adalimumab." Digestive and Liver Disease 44: S69-S70.	Duplicate
59.	Bodini, G., V. Savarino, V. Fazio, L. Assandri, P. Dulbecco, L. Gemignani and E. Savarino (2012). "Relationship between drug serum concentration and clinical activity in patients with crohn disease who achieved remission with adalimumab-a prospective study." Gastroenterology 1): S388.	Insufficient data
60.	Bortlik, M., D. Duricova, K. Malickova, A. Komarek, N. Machkova, E. Bouzkova, L. Hrdlicka and M. Lukas (2012). "Infliximab trough levels may predict sustained response to infliximab in patients with Crohn's disease: A single cohort study." Journal of Crohn's and Colitis 6: S153.	Superseded by full text
61.	Cardile, S., A. Costa, I. Loddo, G. Morabito, C. Pidone and C. Romano (2013). "Impact of measurement of infliximab and anti-infliximab antibodies levels in pediatric inflammatory bowel disease." Digestive and Liver Disease 45: e294-e295.	Insufficient data
62.	Chauhan, U., U. Dutta, D. Armstrong, E. Greenwald, J. Marshall, F. Tse, T. Xenodemetropoulos and H. Smita (2012). "Does measuring infliximab and human anti-chimeric antibody concentrations in patients with inflammatory bowel disease impact clinical management? A canadian experience." Inflamm Bowel Dis 18: S82-S83	Insufficient data
63.	Chauhan, U., U. Dutta, D. Armstrong, J. Marshall, F. Tse, E. Greenwald, T. Xenodemetropoulos and S. Halder (2013). "Does measuring IFX and human anti-chimeric antibody concentrations in patients with inflammatory bowel disease impact clinical management? A Canadian experience." Journal of Crohn's and Colitis 7: S228.	Duplicate
64.	Chollet-Martin, S., P. Nicaise-Roland, L. De Chaisemartin, S. Grootenboer-Mignot, G. Hayem, A. L. Pelletier, A. Amiot, V. Descamps, Y. Bouhnik and O. Meyer (2013). "Simultaneous determination of anti-infliximab antibodies	Insufficient data

For peer review only

and residual infliximab levels to monitor anti-TNF therapy." Annals of the Rheumatic Disease 71.	
65. Church, P., J. Guan, K. Frost, A. Muise, T. Walters and A. Griffiths (2013). "Infliximab treatment for paediatric Crohn's disease: Long-term ouCTomes at a single centre." Journal of Crohn's and Colitis 7: S198.	Insufficient data
66. Church, P., J. Guan, L. Salz, K. Frost, A. Muise, T. Walters and A. Griffiths (2012). "Long-term outcomes with infliximab treatment in children with crohn's disease at a single centre." Inflamm Bowel Dis 18: S72-S7	Insufficient data
67. Cornillie, F., S. Hanauer, R. Diamond, J. Wang, D. Zelinger, Z. Xu, S. Vermeire and P. Rutgeerts (2011). "Early serum infliximab trough level, Clinical disease activity and crp as markers of sustained benefit of infliximab treatment in crohn's disease: A post-HOC analysis of the accent1 trial." American Journal of Gastroenterology 106: S462-S463	Insufficient data
68. Corstjens, P. L., K. Wiesmeijer, G. J. Wolbink, J. Tanke, D. W. Hommes and H. Fidder (2011). "A rapid test for quantitative determination of infliximab trough levels in blood." Gastroenterology 1): S276-S277	Insufficient data
69. Daperno M, Frigerio F, Guiotto C, et al. Evaluation of the diagnostic performance of two commercially available tests for infliximab trough levels (IFX-TL) and antibodies to infliximab (ATI) titration in inflammatory bowel disease (IBD). Journal of Crohn's and Colitis 2013;7:S213-4	Insufficient data
70. Daperno, M., A. Lavagna, M. Fracchia, C. Guiotto, L. Germano, C. Rigazio, E. Ercole, M. Migliardi, R. Pellerito and R. Rocca (2013). "Infliximab trough levels (IFX-TL) are higher in patients with inflammatory bowel disease (IBD)treated with immunosuppressives: Clinical correlations of IFX-LT and antibodies to infliximab (ATI) in IBD." Gastroenterology 1): S781	Insufficient data
71. Daperno, M., A. Lavagna, M. Fracchia, C. Guiotto, L. Germano, C. Rigazio, E. Ercole, M. Migliardi, R. Pellerito and R. Rocca (2013). "Clinical correlations of infliximab trough levels (IFX-TL) and antibodies to infliximab (ATI) in inflammatory bowel disease." Journal of Crohn's and Colitis 7: S239.	Insufficient data
72. Daperno, M., F. Frigerio, C. Guiotto, G. Laura, E. Ercole, A. Lavagna, C. Rigazio, S. Arico, M. Migliardi, R. Pellerito and R. Rocca (2013). "Comparison of the performance of two commercially available tests for determination of infliximab trough levels (IFX-TL) and antibodies to infliximab (ATI), promonitor and immundiagnostik, in inflammatory bowel disease." Digestive and Liver Disease 45: S109.	Insufficient data
73. Daperno, M., F. Frigerio, C. Guiotto, L. Germano, E. Ercole, S. Arico, M. Fracchia, C. Rigazio, A. Lavagna, R. Pellerito, M. Migliardi and R. Rocca (2013). "Identical diagnostic performance of two commercially available tests for infliximab trough levels (IFX-TL) and antibodies to infliximab (ATI) titration in inflammatory bowel disease (IBD): Promonitor and immunodiagnostik tests." Gastroenterology 1): S780.	Duplicate
74. Daperno, M., M. Fracchia, C. Guiotto, L. Germano, E. Ercole, C. Rigazio, A. Lavagna, M. Migliardi, R. Pellerito and R. Rocca (2013). "Clinical implications and stability of determination of infliximab trough levels (IFX-TL) and antibodies to infliximab (ATI) in inflammatory bowel disease." Digestive and Liver Disease 45: S145.	Insufficient data
75. De Bruyn, M., T. Bessissow, T. Billiet, I. Cleynen, R. Kirkland, X. Liu, S. Hauenstein, K. Drake, S. Singh, M. Ferrante, P. Rutgeerts, G. Van Assche, I. Arijs, G. Opdenakker and S. Vermeire (2014). "Biomarker panel for prediction of mucosal healing in patients with Crohn's disease under infliximab therapy." Journal of Crohn's and Colitis 8: S45-S46.	Insufficient data
76. De Bruyn, M., T. Bessissow, T. Billiet, I. Cleynen, R. Kirkland, X. Liu, S. Hauenstein, K. Drake, S. Singh, M. Ferrante, P. J. Rutgeerts, G. A. Van Assche, I. Arijs, G. Opdenakker and S. Vermeire (2014).	Duplicate
77. Dotan, I., H. Yanai, Y. Ron, R. Kariv, S. Fishman, L. Yahav, M. Ben-Yehoyada, E. Santo and D. R. Mould (2014). "Population pharmacokinetic evaluation of adlimumab reveals patient factors that increase adalimumab clearance and shorten half-life in inflammatory bowel disease patients."	Insufficient data

For peer review only

Gastroenterology 1): S-243.	
78. Dotan, I., Y. Ron, H. Yanai, S. A. Becker, S. Fishman, L. Yahav, M. B. Yehoyada and D. R. Mould (2013). "Population pharmacokinetic evaluation of infliximab reveals patient factors that increase infliximab clearance and shorten half-life in inflammatory bowel disease patients." Gastroenterology 1): S774.	Insufficient data
79. Drastich, P., J. Kozeluhova, M. Jaresova and J. Spicak (2011). "Infliximab serum trough levels and deep remission in patients with IBD." Gastroenterology 1): S292	Insufficient data
80. Drobne, D., P. Bossuyt, C. Breynaert, N. Vande Casteele, G. Compernolle, M. Juergens, V. Ballet, I. Cleynen, W. Wollants, A. Gils, P. Rutgeerts, S. Vermeire and G. Van Assche (2011). "Long term evolution and impact of immunomodulator co-treatment and withdrawal on infliximab trough levels in 223 patients with Crohn's disease." Journal of Crohn's and Colitis 5 (1): S10-S11.	Insufficient data
81. Drobne, D., P. J. Bossuyt, C. Breynaert, N. V. Casteele, G. Compernolle, M. Jurgens, V. Ballet, W. J. Wollants, I. Cleynen, P. J. Rutgeerts, S. Vermeire, A. Gils and G. A. Van Assche (2011). "Crohn's disease: Infliximab trough levels and CRP during infliximab-immunomodulator combination treatment are associated with clinical ouCTome after immunomodulator withdrawal." Gastroenterology 1): S62.	Insufficient data
82. Duricova, D., K. Malickova, M. Bortlik, N. Machkova, V. Komarek, E. Bouzkova and M. Lukas (2011). "Predictors of sustained response to infliximab in patients with Crohn's disease: A single cohort study." Gastroenterology 1): S593.	Insufficient data
83. Echarri, A., R. Ferreiro, R. Fraga-Iriso, M. Barreiro-De Acosta, J. Cid, L. De-Castro, S. Pereira, A. Fernandez-Villaverde, S. Soto, D. Carpio, B. Gonzalez, E. Castro, V. Ollero and A. C. Campos (2014). "Drug trough levels and primary nonresponse to antitnf therapy in moderate-severe crohn disease. Results of the optimiza study." Gastroenterology 1): S-247.	Insufficient data
84. Egea-Pujol L, Reddy R, Patel S, et al. Homogenous mobility shift assay (HMSA) overcomes the limitations of elisa and eclia assays for monitoring infliximab (IFX), adalimumab (ADA), and associated anti-drug antibodies in serum. Am J Gastroenterol 2013;108:S548 doi: http://dx.doi.org/10.1038/ajg.2013.269[published Online First: Epub Date]|.	Insufficient data
85. Eser A, Primas C, Hauenstein S, et al. Comparison of early measurement of infliximab and antibodies-to-infliximab serum levels with standard trough analysis. Gastroenterology 2013;144(5 Suppl):S-779	Insufficient data
86. Eser A, Primas C, Haunstein S, et al. Detection of anti infliximab antibodies in patients with inflammatory bowel disease (IBD) in the presence of infliximab by homogeneous liquid phase anti infliximab mobility shift assay. Journal of Crohn's and Colitis 2013;7:S231-2	Insufficient data
87. Eser, A., C. Primas, R. Shringarpure, S. Hauenstein, S. L. Wang and W. Reinisch (2012). "Detection of anti infliximab antibodies in patients with inflammatory bowel disease (IBD) in the presence of infliximab by homogeneous liquid phase anti infliximab mobility shift assay." American Journal of Gastroenterology 107: S657	Duplicate
88. Fasanmade, A. A., C. Wagner, H. Davis, M. Graham, D. Everitt and A. Gottlieb (2002). "Comparison of the pharmacokinetics of infliximab in patients with psoriasis or Crohn's disease not receiving concomitant immunosuppressants or corticosteroids." Journal of Investigative Dermatology 119(1): 243-243.	Insufficient data
89. Fasanmade, A. A., P. Marsters, E. Munsanje, M. A. Graham, H. M. Davis and S. Van Deventer (2003). "Infliximab pharmacokinetics and improvement in fistulizing Crohn's Disease." Gastroenterology 124(4): A61-A61.	Insufficient data
90. Fasanmade, A. A., Y. W. Zhu, C. Wagner, C. Pendley and H. M. Davis (2002). "Population pharmacokinetics of single dose infliximab in patients with Crohn's disease." Clinical Pharmacology & Therapeutics 71(2): P66-P66.	Insufficient data

For peer review only

91. Fasanmade, A., A. Olson, W. Bao, C. Pendley, H. Davis and L. Mayer (2002). "Relationship between infliximab pharmacokinetics and improvement in Crohns disease." Gastroenterology 122(4): A617-A618.	Insufficient data
92. Feagan BG, Singh S, Lockton S, et al. Novel infliximab (IFX) and antibody-to-infliximab (ATI) assays are predictive of disease activity in patients with crohn's disease (CD). Gastroenterology 2012;142(5 Suppl):S-114	Insufficient data
93. Garces, S., J. Demengeot and E. Benito-Garcia (2012). "Clinical impact of immunogenicity of infliximab, adalimumab and etanercept: A systematic review of the literature with a meta-analysis." Annals of the Rheumatic Disease 71	Superseded by full paper
94. Garces, S., J. Demengeot, G. J. Wolbink, L. Aarden and E. Benito-Garcia (2011). "The immunogenicity of infliximab, adalimumab and etanercept in rheumatoid arthritis, ankylosing spondylitis, psoriatic arthritis, crohn's disease and ulcerative colitis a quantitative and a qualitative review." Arthritis and Rheumatism 1).	Superseded by full paper
95. Garces, S., J. Demengeot, J. C. Da Silva and L. Aarden (2011). "Bridging elisa as a screening assay to monitor immunogenicity in routine clinical practice." Arthritis and Rheumatism 1).	<50% CD
96. Garces, S., J. Demengeot, J. Canas-da-Silva and L. Aarden (2013). "Bridging ELISA as a secreening assay to monitor immunogenicity in routine clinical practice." Annals of the Rheumatic Disease 71.	Duplicate
97. Garces, S., J. Freitas, J. Canas-Silva, L. Aarden and J. Demengeot (2013). "The impact of immunogenicity on drug safety profile." Annals of the Rheumatic Diseases 72.	Insufficient data
98. Garimella, T. S., J. Z. Peng, K. Beck, P. A. Noertersheuser, K. G. Lomax, S. K. Paulson and P. F. Pollack (2006). "Pharmacokinetics of adalimumab in a long-term investigation of the induction and maintenance of remission in patients with Crohn's disease (CLASSIC I and CLASSIC II)." Gastroenterology 130(4): A481-A481.	Insufficient data
99. Goldberg R, Beswick L, Van Langenberg D, et al. Predictors of sub-therapeutic infliximab or adalimumab trough levels and anti-drug antibodies and their influence on therapeutic decisions. Journal of Crohn's and Colitis 2014;8:S223	Insufficient data
100. Greathead L, Kelleher P, Steel A. Development and validation of ELISA to measure serum anti TNFa levels. Journal of Crohn's and Colitis 2014;8:S97-8	Insufficient data
101. Guan, J., L. Salz, K. Frost, A. Muise, T. Walters and A. Griffiths (2012). "Long-term outcomes with infliximab treatment in children with crohn's disease at a single centre peter church." Inflamm Bowel Dis 18: S5-S6.	Duplicate
102. Guilday, C., D. Eastwood, Y. Zadvornova, D. Stein, A. S. Naik, K. Best, S. Skaros and L. P. Perera (2013). "Concomitant use of immunomodulator therapy results in higher serum infliximab levels compared to monotherapy without lowering serum haca levels." Gastroenterology 1): S429.	Insufficient data
103. Guiotto, C., L. Germano, M. Vizzini, R. Cerruti, F. Frigerio, M. Daperno, R. Rocca and M. Migliardi (2013). "Determination of infliximab trough levels (IFX-TL) and antibodies to Infliximab (ATI) in inflammatory bowel disease." Biochimica Clinica 37: S475.	Superseded
104. Hadigan, C. B. R., C. P. Braegger, E. Vasilauskis, J. C. Escher, M. Sinaasappel, G. D. Ferry, B. Kirschner, C. Wagner, R. Livingston, K. DeWoody and H. S. Winter (1999) Pharmacokinetics of infliximab (Anti-TNFx) in children with Crohn's disease: A multicenter trial. Journal of Pediatric Gastroenterology and Nutrition 29, 525	Insufficient data
105. Hauenstein S, Ohrmund L, Salbato J, et al. Comparison of homogeneous mobility shift assay and solid phase elisa for the measurement of drug and anti-drug antibody (ADA) levels in serum from patients treated with anti-TNF biologics. Gastroenterology 2012;142(5 Suppl):S-538	Insufficient data
106. Hauenstein, S., J. Salbato, S. Lockton and S. Singh (2013). "Characterization of neutralizing anti-drug antibody response in patients with loss of response to anti-TNF therapy." Gastroenterology 1): S418	Insufficient data

For peer review only

107. Hayes and Inc (2013) Use of anti-infliximab antibody levels to monitor infliximab treatment in patients with inflammatory bowel disease (IBD) (Structured abstract). Health Technology Assessment Database	Not available
108. Hester, K. D., X. Liu, J. Salbato, S. Lockton, S. Hauenstein and S. Singh (2014). "Improved homogeneous mobility shift assay (HMSA) for the detection of neutralizing antibodies (NAB) in ibd patients treated with infliximab or adalimumab." Gastroenterology 1): S-248	Insufficient data
109. Hibi, T., A. Sakuraba, M. Watanabe, S. Motoya, H. Ito, N. Sato, T. Yoshinari, K. Motegi, K. Yoshitaka, M. Takazoe, Y. Suzuki, T. Matsumoto, K. Kawakami, I. Hirata, S. Tanaka, T. Ashida and T. Matsui (2012). "Decrease in serum infliximab level precedes loss of clinical response and can be easily detected by the elevation of C-reactive protein in crohn's disease." Gastroenterology 1): S388.	Insufficient data
110. Hoekman, D. R., J. F. Brandse, T. De Meij, T. Hummel, M. Lowenberg, M. A. Benninga, G. R. D'Haens and A. Kindermann (2014). "Large variation in infliximab trough levels is associated with disease activity in paediatric inflammatory bowel disease." Gastroenterology 1): S-782.	Insufficient data
111. Hoekman, D., H. Brandse, T. De Meij, T. Hummel, M. Lowenberg, M. Benninga, G. D'Haens and A. Kindermann (2014). "Large variation in infliximab trough levels is associated with disease activity in paediatric inflammatory bowel disease." Journal of Crohn's and Colitis 8: S35.	Insufficient data
112. Huang, V. W., C. Prosser, C. Shalapay, D. K. Fedorak, N. Dhami, H. Wang, K. I. Kroeker and R. N. Fedorak (2014). "In IBD outpatients knowledge of fecal calprotectin and infliximab trough levels significantly enhances infliximab dose escalation decision making." Journal of Crohn's and Colitis 8: S255.	Insufficient data
113. Huang, V. W., N. Dhami, D. K. Fedorak, C. Prosser, C. Shalapay, K. I. Kroeker and R. N. Fedorak (2014). "Disparity between infliximab trough level and infliximab associated adverse events." Journal of Crohn's and Colitis 8: S282	Insufficient data
114. Huang, V., K. I. Kroeker, H. Wang, C. Prosser, S. Carol, N. Dhami, D. K. Fedorak and R. N. Fedorak (2014). "In IBD outpatients knowledge of fecal calprotectin and infliximab trough levels significantly alters clinical decision making." Gastroenterology 1): S241-S242.	Duplicate
115. Huang, V., N. Dhami, D. K. Fedorak, C. Prosser, S. Carol, K. I. Kroeker and R. N. Fedorak (2014). "Infliximab trough levels are correlated with infliximab-associated adverse events." Gastroenterology 1): S-1.	Insufficient data
116. Imaeda, H., A. Andoh, H. Ban, S. Bamba, M. Sasaki, T. Tsujikawa and Y. Fujiyama (2012). "The new immunoassay for the accurate determination of antibodies to infliximab, and relationship between its serum level and inflammatory values in crohn's disease." Gastroenterology 1): S349.	Superseded by full text
117. Imaeda, H., A. Andoh, K. Takahashi, T. Fujimoto, H. Ban, S. Bamba and Y. Fujiyama (2012). "Serum infliximab trough levels above 1.0 mg/ml are required to obtain clinical efficacy in patients with crohn's disease." Inflamm Bowel Dis 18: S59-S60.	Insufficient data
118. Imaeda, H., A. Andoh, S. Bamba, T. Tsujikawa and Y. Fujiyama (2011). "Development of a new Immunoassay for the accurate determinationof anti-infliximab antibodies in Crohn's Disease." Inflamm Bowel Dis 17: S42.	Duplicate
119. Imaeda, H., K. Takahashi, T. Fujimoto, S. Bamba, M. Sasaki, T. Tsujikawa, Y. Fujiyama and A. Andoh (2013). "Accurate determination of serum adalimumab and anti-adalimumab antibodies levels during maintenance therapy for crohn's disease." Gastroenterology 1): S431.	Insufficient data
120. Irving, P. M., Z. Arkir, J. Duncan, M. Sastrillo, S. Anderson and J. Sanderson (2012). "Initial experience with infliximab levels in a tertiary IBD centre." Gut 61: A238.	Insufficient data
121. Jauregui-Amezaga, A., I. Ordas, M. Gallego, A. Ramirez, S. Pino, M. C. Masamunt, M. Juan, E. Ricart, J. Yague and J. Panes (2013). "Impact of serum drug level and human anti-drug antibody measurement on management of biologic drugs in inflammatory bowel disease." Journal of	Insufficient data

For peer review only

Crohn's and Colitis 7: S202-S203.	
122. Juan, G., A. Alvarino, L. Oltra, N. Maroto, N. Cano, I. Ferrer and J. Hinojosa (2014). "Utility of "trough levels" determination and anti-infliximab antibodies in patients with inflammatory bowel disease. Estimation of individual pharmacokinetic parameters (PK) through population pharmacokinetic model." Journal of Crohn's and Colitis 8: S190	Insufficient data
123. Karmiris, K., G. Paintaud, D. Degenne, M. Ferrante, A. C. Duveau, M. Noman, G. A. Van Assche, S. Vermeire and P. Rutgeerts (2008). "Adalimumab trough serum levels and clinical response in a single-center cohort of inflammatory bowel disease patients: Can trough serum levels serve as a predictor for future loss of response?" Gastroenterology 134(4): A68-A68.	Insufficient data
124. Karmiris, K., M. Noman, G. Paintaud, M. Ferrante, A. C. Duveau, D. Degenne, G. A. Van Assche, S. Vermeire and P. Rutgeerts (2008). "A 3-week course of 80 mg weekly administered adalimumab as a rescue therapy for patients with Crohn's disease who lost response to 40 mg weekly: Relationship with adalimumab trough serum levels." Gastroenterology 134(4): A640-A640.	Insufficient data
125. Karsan, S. S., E. R. Cohen, S. R. Targan, A. Ippoliti, D. Q. Shih, E. A. Vasiliauskas, M. Dubinsky, D. Berel and D. P. McGovern (2012). "Analysis of clinical and serological associations, and the clinical consequences of the development of human anti-chimeric antibodies (HACAS), and low serum infliximab (IFX) levels in inflammatory bowel disease (IBD)." Gastroenterology 1): S264	Insufficient data
126. Kerr, J., A. Nair and R. Hinds (2013). "Variable practice in children with inflammatory bowel disease requiring infliximab infusions across Australia." Journal of Gastroenterology and Hepatology 28: 141.	Insufficient data
127. Kong, J. Y., C. Bundell, J. Pawlik, P. Hollingsworth and G. Forbes (2013). "Low Trough serum infliximab and antibodies to infliximab in smokers." Inflamm Bowel Dis 19(3): E35-E36.	Insufficient data
128. Kong, J. Y., C. S. Bundell, J. Pawlik, P. N. Hollingsworth and G. M. Forbes (2011). "Smoking is associated with low trough serum infliximab levels and presence of anti-infliximab antibody in maintenance treatment of inflammatory bowel disease (IBD)." Journal of Gastroenterology and Hepatology 26: 59	Insufficient data
129. Lamblin, C., A. Aubourg, D. Ternant, L. Picon, T. Lecomte and G. Paintaud (2012). "Concentration effect relationship of infliximab in Crohn's disease: Results of a cohort study." Journal of Crohn's and Colitis 6: S142-S143.	Insufficient data
130. Leclerc, M., H. Marotte, S. Paul, E. Del Tedesco, P. Gonzalo, J. M. Phelip, L. Peyrin Biroulet and X. Roblin (2014). "Persistence of antibodies to infliximab for more than two months strongly predicts loss of response to infliximab in inflammatory bowel diseases." Journal of Crohn's and Colitis 8: S226-S227.	Insufficient data
131. Li, J. L., S. K. Paulson, Y. L. Chiu, A. Robinson, K. G. Lomax and P. F. Pollack (2010). "Evaluation of potential correlations between serum adalimumab concentration and remission in patients with Crohn's disease in classic I and II." Gastroenterology 1): S101.	Insufficient data
132. Lowenberg, M., J. Brandse, L. Vos, C. Ponsioen, G. Van Den Brink and G. D'Haens (2014). "High infliximab trough levels are associated with impaired quality of life in IBD patients in clinical and biochemical remission on maintenance IFX therapy." Journal of Crohn's and Colitis 8: S262-S263	Insufficient data
133. Lowenberg, M., J. F. Brandse, L. M. Vos, C. Ponsioen, G. R. Van Den Brink and G. R. D'Haens (2014). "High infliximab trough levels are associated with impaired quality of life in IBD patients in clinical and biochemical remission on maintenance IFX therapy." Gastroenterology 1):	Insufficient data

For peer review only

S-450.	
134. Lukas, M., K. Malickova, M. Bortlik and D. Duricova (2009). "Anti-infliximab antibodies in routine clinical practice - Is it worth to assess them?" Gastroenterology 1): A679.	Insufficient data
135. Malickova, K., I. Janatkova, D. Duricova, M. Bortlik and M. Lukas (2011). "Serum infliximab levels, antibodies to infliximab and albumin concentrations during infliximab treatment in patients with inflammatory bowel disease." Clinical and Experimental Rheumatology 29(1): 213-213.	Insufficient data
136. Martin Arranz, M. D., E. Martin Arranz, D. Pascual-Salcedo, C. De Diego, M. Jaquotot, S. Gomez Senent, J. Poza and J. M. Suarez Parga (2014). "Infliximab trough levels and antibodies: Relationship with infusion reaction, immunomodulators and biological parameters." Journal of Crohn's and Colitis 8: S251	Insufficient data
137. Mazor Y, Kopylov U, Hur DB, et al. Evaluating adalimumab drug and antibody levels as predictors of clinical and laboratory response in crohn's disease patients. Gastroenterology 2013;144(5 Suppl):S-778	Insufficient data
138. Mazor, Y., U. Koplov, D. Ben Hur, R. Almog, M. Waterman, S. Ben-Horin and Y. Chowers (2013). "Evaluating Adalimumab drug and antibody levels as predictors of clinical and laboratory response in Crohn's disease patients." Journal of Crohn's and Colitis 7: S217.	Insufficient data
139. McTigue M, Sandborn W, Levesque B, et al. Clinical utility of next generation infliximab and antibodies to infliximab assay. Am J Gastroenterol 2013;108:S527 doi: http://dx.doi.org/10.1038/ajg.2013.269[published Online First: Epub Date]|.	Insufficient data
140. McTigue, M., W. Sandborn, B. Levesque and D. Patel (2013). "Infliximab therapeutic drug monitoring in clinical practice: Indications and utility." American Journal of Gastroenterology 108: S512.	Insufficient data
141. Morgenstern, J., E. Baestlein, L. Leifeld, P. Nguyen, J. Stein and W. Kruis (2012). "Infliximab drug levels in Crohn's disease responding to the treatment." Journal of Crohn's and Colitis 6: S125	Insufficient data
142. Noman, M., F. Baert, S. Vermeire, G. Van Assche, G. D'Haens, A. Carbonez and P. Rutgeerts (2002). "Post infusion infliximab levels determine duration of response in Crohn's disease and are directly related to infusion reactions." Gastroenterology 122(4): A100-A100.	Insufficient data
143. O'Donnell, S., J. M. Stempak and M. S. Silverberg (2014). "Is there a higher rate of infliximab dose optimization in initial responders between UC and CD cases?" Gastroenterology 1): S462-S463.	Insufficient data
144. Papamichail, K., N. V. Casteele, S. Hauenstein, F. Princen, S. Singh, M. Ferrante, G. A. Van Assche, P. J. Rutgeerts, A. Gils and S. Vermeire (2014). "Prediction of sustained remission after discontinuation of infliximab in patients with crohn's disease." Gastroenterology 1): S-457.	Insufficient data
145. Pariente, B., G. P. De Chambrun, M. Desroches, C. De Cassan, J. M. Gornet, P. Desreumaux, R. Krzysiek, D. Emilie, J. F. Colombel and M. Allez (2011). "Clinical value of measuring trough levels and human anti-chimeric antibodies in patients with inflammatory bowel disease who lost response to infliximab therapy." Gastroenterology 1): S277.	Superseded by full text
146. Pariente, B., G. Pineton De Chambrun, M. Desroches, C. De Cassan, J. Gornet, P. Desreumaux, R. Krzysiek, D. Emilie, J. Colombel and M. Allez (2011). "Clinical value of measuring trough levels and human anti-chimeric antibodies in patients with inflammatory bowel disease who lost response to infliximab therapy." Journal of Crohn's and Colitis 5 (1): S111-S112.	Duplicate
147. Paul S, Tedesco ED, Marotte H, et al. Interest of the dosage of serum concentration of infliximab and antibodies anti infliximab in the therapeutic response under infliximab in IBD. Gastroenterology 2012;142(5 Suppl):S354	Insufficient data

For peer review only

148. Paul, S., E. Del Tedesco, H. Marotte, L. Clavel, J. M. Phelip, L. Peyrin-Biroulet and X. Roblin (2013). "Therapeutic drug monitoring of infliximab and mucosal healing in inflammatory bowel disease: A prospective study." Gastroenterology 1): S92.	Superseded by full text
149. Paul, S., E. Del Tedesco, H. Marotte, M. Rinaudo-Gaujous, J. M. Phelip, L. Peyrin-Biroulet and X. Roblin (2013). "Infliximab concentration is associated with mucosal healing in intestinal bowel disease (IBD)." Journal of Crohn's and Colitis 7: S199-S200.	Insufficient data
150. Paulson, S. K., P. Nocrtersheuser, P. F. Pollack and R. S. Hoffman (2005). "Pharmacokinetics of adalimumab from classic, a randomized phase 3 trial for the induction of clinical remission in patients with Crohn's." Gastroenterology 128(4): A585-A585.	Insufficient data
151. Pradhan, R. S., S. Sharma, R. Thakkar, A. Robinson, J. S. Hyams, J. R. Rosh, F. Ruemmele and W. M. Awni (2013). "Relationship between adalimumab concentration and efficacy for the induction of clinical remission in pediatric patients with moderate to severe crohn's disease." Gastroenterology 1): S230-S231.	Duplicate
152. Pradhan, R., S. Sharma, R. Thakkar, A. Robinson, J. Hyams, J. Rosh, F. M. Ruemmele and W. Awni (2013). "Relationship between adalimumab concentration and efficacy for the induction of clinical remission in pediatric patients with moderate to severe Crohn's disease." Journal of Crohn's and Colitis 7: S164.	Insufficient data
153. Rai, T., U. Navaneethan, D. Dalal, B. Lashner and B. Shen (2012). "Clinical implications of measuring infliximab levels and human anti-chimeric antibodies in patients with inflammatory bowel disease." American Journal of Gastroenterology 107: S634.	Insufficient data
154. Rekvig, M., M. Gedde Dahl, J. Bratlie, N. Bolstad, B. Moum, J. Jahnsen and K. E. A. Lundin (2014). "Anti-TNFalpha drug level measurements in IBD patients." Journal of Crohn's and Colitis 8: S301-S302.	Insufficient data
155. Roblin, X., H. Marotte, E. Del Tedesco, M. Rinaudo-gaujous, J. M. Phelip and S. Paul (2013). "Residual adalimumab trough levels are associated with clinical remission and mucosal healing in IBD." Gastroenterology 1): S778.	Insufficient data
156. Roblin, X., M. Rinaudo, E. Del Tedesco, J. M. Phelip, L. Peyrin Biroulet and S. Paul (2014). "Development of an algorithm incorporating pharmacokinetics of adalimumab in inflammatory bowel diseases." Journal of Crohn's and Colitis 8: S41.	Insufficient data
157. Roblin, X., M. Rinaudo-gaujous, E. Del Tedesco, J. M. Phelip, C. Genin, L. Peyrin-Biroulet and S. Paul (2014). "Development of an algorithm incorporating pharmacokinetics of adalimumab in inflammatory bowel diseases." Gastroenterology 1): S-150.	Duplicate
158. Rosenthal, C., G. Melmed, B. Tripuraneni, J. Gebbia, S. Callejas, S. Farrior, S. Rabizadeh and M. Dubinsky (2012). "Early infliximab trough levels predict remission at one year in pediatric IBD patients." Inflamm Bowel Dis 18: S81.	Insufficient data
159. Rubin, D., S. Hauenstein and S. Singh (2013). "Post-marketing review of serum adalimumab and antibodies to adalimumab using the mobility shift assay platform." American Journal of Gastroenterology 108: S532.	Insufficient data
160. Scaldaferri, F., S. Pecere, V. Petito, G. Cammarota, M. C. Campanale, G. L. Rapaccini, A. Armuzzi, L. Guidi, A. Poscia, A. Amato, A. Sgambato, A. Gasbarrini and A. Papa (2012). "Infliximab and TNF ALFA measurement in intestinal mucosa of IBD patients: A new tool for the clinic?" Digestive and Liver Disease 44: S189-S190.	Insufficient data
161. Schatz, S. B., C. Prell, F. Freudenberg, T. Schwerd, P. Bufler and S. Koletzko (2011). "Correlation of infliximab levels and antibodies with clinical outcome in children with IBD." Journal of Pediatric Gastroenterology and Nutrition 52: E45.	Insufficient data

For peer review only

162. Semmler J, Pilch A, Armbruster F, et al. Development of a new immunoassay for the accurate determination of anti-infliximab antibodies in inflammatory bowel disease. Clin Chem Lab Med 2013;51 (10):eA27-8 doi: http://dx.doi.org/10.1515/cclm-2013-0737[published Online First: Epub Date]|.	Insufficient data
163. Semmler, J. M., A. Pilch, F. P. Armbruster, A. Dignass, W. Kruis and J. Stein (2014). "Development of a new immunoassay for the accurate determination of anti-infliximab antibodies in inflammatory bowel disease." Journal of Crohn's and Colitis 8: S41.	Duplicate
164. Semmler, J., A. Pilch, F. P. Armbruster, A. Dignass and J. Stein (2014). "Development of a new immunoassay for the accurate determination of anti-infliximab antibodies in inflammatory bowel disease." Clinical Chemistry and Laboratory Medicine 52: S1008.	Duplicate
165. Settesoldi, A., M. Giannotta, M. Milla, S. Genise, A. Santini, S. Bagnoli, V. Annese, A. Matucci, A. Vultaggio, G. Petroni, S. Pratesi, F. Nencini and E. Maggi (2012). "Loss of efficacy and adverse drug reactions during infliximab therapy in IBD patients are related to the appearance of anti-infliximab antibodies." Journal of Crohn's and Colitis 6: S151.	Duplicate
166. Settesoldi, A., M. Giannotta, S. Genise, A. Santini, S. Bagnoli, M. Milla, V. Annese, A. Matucci, A. Vultaggio, G. Petroni, S. Pratesi, F. Nencini and E. Maggi (2012). "Loss of efficacy and adverse drug reactions during infliximab therapy in IBD patients are related to the appearance of anti-infliximab antibodies." Digestive and Liver Disease 44: S194.	Insufficient data
167. Sharma, S., R. Pradhan, R. Thakkar, A. Robinson, J. Hyams, J. Rosh, F. M. Ruemmele and W. Awni (2013). "Relationship between adalimumab concentration and efficacy for the maintenance of clinical remission in pediatric patients with moderate to severe Crohn's disease." Journal of Crohn's and Colitis 7: S163.	Insufficient data
168. Sharma, S., R. S. Pradhan, R. Thakkar, A. Robinson, J. S. Hyams, J. R. Rosh, F. Ruemmele and W. M. Awni (2013). "Relationship between adalimumab concentration and efficacy for the maintenance of clinical remission in pediatric patients with moderate to severe crohn's disease." Gastroenterology 1): S231.	Insufficient data
169. Sorrentino, D., S. Hauenstein, M. Marino, S. Lockton, D. Zarifi, T. Del Bianco and S. Singh (2013). "Low dose infliximab for prevention of postoperative recurrence of crohn's disease: Long term follow-up and impact of infliximab trough levels and antibodies to infliximab." Gastroenterology 1): S777	Insufficient data
170. Steenholdt, C., J. Brynskov, O. Thomsen, L. K. Munck, J. Fallingborg, L. A. Christensen, G. Pedersen, J. Kjeldsen, K. Bendtzen and M. A. Ainsworth (2013). "Secondary infliximab treatment failure in crohn's disease: Therapeutic implications of measuring drug and anti-drug antibodies by three different binding assays." Gastroenterology 1): S773	Duplicate
171. Steenholdt, C., J. Brynskov, O. Thomsen, L. K. Munck, J. Fallingborg, L. A. Christensen, G. Pedersen, J. Kjeldsen, B. A. Jacobsen, A. S. Oxholm, J. Kjellberg, K. Bendtzen and M. A. Ainsworth (2013). "Treatment of secondary infliximab failure in crohn's disease based on serum levels of infliximab and antibodies against infliximab: The danish study of optimizing infliximab therapy in crohn's disease (do it crohn) randomized clinical trial." Gastroenterology 1): S22.	Superseded by full paper
172. Steenholdt, C., J. Brynskov, O. Thomsen, L. K. Munck, J. Fallingborg, L. A. Christensen, G. Pedersen, J. Kjeldsen, K. Bendtzen and M. A. Ainsworth (2013). "Secondary infliximab treatment failure in crohn's disease: Therapeutic implications of measuring drug and anti-drug antibodies by three different binding assays." Gastroenterology 1): S773.	Duplicate
173. Steenholdt, C., J. Brynskov, O. Thomsen, L. Munck, J. Fallingborg, L. Christensen, G. Pedersen, J. Kjeldsen, K. Bendtzen and M. Ainsworth (2013). "Secondary infliximab treatment failure in Crohn's disease:	Superseded by full

Therapeutic implications of measuring drug and anti-drug antibodies by three different binding assays." Journal of Crohn's and Colitis 7: S159.	text
174. Steenholdt, C., J. Brynskov, O. Thomsen, L. Munck, J. Fallingborg, L. Christensen, G. Pedersen, J. Kjeldsen, K. Bendtzen, S. Lockton, S. Hauenstein, R. Shringarpure, E. Chuang, S. Singh and M. Ainsworth (2012). "Comparison of techniques for monitoring infliximab and antibodies to infliximab in Crohn's disease patients with infliximab treatment failure." American Journal of Gastroenterology 107: S622.	Superseded by full text
175. Steenholdt, C., K. Bendtzen, J. Brynskov, O. O. Thomsen and M. A. Ainsworth (2014). "Clinical implications of measuring drug and anti-drug antibodies by different assays when optimizing infliximab treatment failure in Crohn's disease." Journal of Crohn's and Colitis 8: S291.	Duplicate
176. Steenholdt, C., K. Bendtzen, J. Brynskov, O. Thomsen and M. A. Ainsworth (2014). "Clinical implications of measuring drug and anti-drug antibodies by different assays when optimizing infliximab treatment failure in crohn's disease." Gastroenterology 1): S-240.	Superseded by full text
177. Steenholdt, C., K. Bendtzen, O. O. Thomsen, J. Brynskov and M. Ainsworth (2010). "Discriminating between response types in infliximab-treated patients with Crohn's disease: Sensitivity and specificity of combined assessment of infliximab trough levels and antidrug antibodies." Scandinavian Journal of Gastroenterology 45: 59-59.	Superseded by full text
178. Steenholdt, C., M. Svenson, K. Bendtzen, O. Thomsen, J. Brynskov and M. A. Ainsworth (2011). "Can measurements of anti-infliximab antibodies predict acute severe infusion reactions to inflixims241ab?" Gastroenterology 1): S774	Insufficient data
179. Steenholdt, C., M. Svenson, M. A. Ainsworth, O. Thomsen, J. Brynskov and K. Bendtzen (2012). "Comparison of techniques for monitoring infliximab bioavailability and immunogenicity in crohn's disease." Gastroenterology 1): S781	Insufficient data
180. Steenholdt, C., O. O. Thomsen, J. Brynskov, K. Bendtzen and M. A. Ainsworth (2010). "Discriminating between response types in infliximab-treated patients with Crohn's disease: Sensitivity and specificity of combined assessment of infliximab trough levels and anti-drug antibodies." Gastroenterology 1): S687-S688.	Insufficient data
181. Steenholdt, C., Y. Palarasah, K. Bendtzen, A. Teisner, B. Teisner, J. Brynskov and C. Nielsen (2013). "Pre-existing IgG antibodies to the Fab region of infliximab predict efficacy and safety in IBD patients naive to anti-TNF agents." Journal of Crohn's and Colitis 7: S6.	Insufficient data
182. Steenholdt, C., Y. Palarasah, K. Bendtzen, A. Teisner, J. Brynskov, B. Teisner and C. H. Nielsen (2013). "Pre-existing IGG antibodies to the fab region of infliximab predict efficacy and safety in ibd patients naive to anti-TNF agents." Scandinavian Journal of Immunology 77 (4): 333.	Insufficient data
183. Szepes, Z., E. Kunstar, K. Farkas, F. Nagy, R. Gyulai, R. Kui, A. Kinyo, A. Balint, M. Szucs, T. Wittmann and T. Molnar (2013). "Clinical utility of measuring serum TNF alpha level, anti TNF alpha levels and antibody titers in critical situations in inflammatory bowel disease and in psoriasis." Journal of Crohn's and Colitis 7: S118-S119.	Insufficient data
184. Tang, J., X. Gao, M. Zhi, H. Zhou, H. Chen, M. Zhang, Q. Yang and Z. Liang (2014). "Serum infliximab levels and early mucosal healing in Crohn's disease." Journal of Crohn's and Colitis 8: S209-S210.	Insufficient data
185. Turon, J., A. Langseder, R. Irizarry, K. Ahuja and J. R. Rosh (2013). "Clinical outcome of pediatric IBD patients after measurement of infliximab drug and anti-drug antibody levels." Gastroenterology 1): S531.	Insufficient data
186. Ungar B, Anafy A, Kopylov U, et al. The clinical and immunological significance of low level of infliximab in the absence of anti-infliximab antibodies in patients with IBD. Gastroenterology 2014;146(5 Suppl):S-245 doi: http://dx.doi.org/10.1016/S0016-5085%2814%2960862-3[published	Insufficient data

Online First: Epub Date]|.	
187. Ungar, B., A. Anafy, M. Yavzori, O. Picard, E. Fudim, U. Kopylov, Y. Ron, H. Yanai, I. Dotan, Y. Chowers, R. Eliakim and S. Ben-Horin (2014). "The clinical and immunological significance of low level of infliximab in the absence of anti-infliximab antibodies in patients with IBD." Journal of Crohn's and Colitis 8: S113.	Duplicate
188. Ungar, B., U. Kopylov, M. Yavzori, E. Fudim, O. Picard, A. Lahat, B. Avidan, A. Lang, B. Weiss, Y. Chowers, R. Eliakim and S. Ben-Horin (2014). "Predictors of formation of antibodies to infliximab (ATI) and secondary loss of response in IBD patients treated with infliximab." Journal of Crohn's and Colitis 8: S45.	Insufficient data
189. Ussia, V., L. Ceccarelli, S. Maltinti, G. Di Fluri, M. G. Mumolo, V. Bolognesi, A. Ricchiuti, M. Bellini, S. Marchi and F. Costa (2014). "A prospective assessment of antidrug antibody response over time by a new ELISA in patients with IBD treated with infliximab." Journal of Crohn's and Colitis 8: S298-S299.	Insufficient data
190. Van Der Woude, C. J., E. Bultman, J. Deuring, R. West, Z. Zelinkova and M. Peppelenbosch (2013). "Adalimumab trough levels in a prospective cohort of Crohn's disease patients." Journal of Crohn's and Colitis 7: S250.	Insufficient data
191. Van Der Woude, C. J., J. J. Deuring, R. West, Z. Zelinkova and M. P. Peppelenbosch (2013). "Adalimumab trough levels in a prospective cohort of crohn's disease patients." Gastroenterology 1): S567.	Duplicate
192. Van Moerkercke, W., C. Ackaert, G. Compernolle, M. Jurgens, I. Cleynen, G. A. Van Assche, P. J. Rutgeerts, A. Gils and S. Vermeire (2010). "High infliximab trough levels are associated with mucosal healing in Crohn's disease." Gastroenterology 1): S60.	Insufficient data
193. Van Moerkercke, W., G. Compernolle, C. Ackaert, A. Gils, S. Vermeire, M. Jurgens, I. Cleynen, G. Van Assche and P. Rutgeerts (2010). "Mucosal healing in Crohn's disease is associated with high infliximab trough levels." Journal of Crohn's and Colitis Supplements 4 (1): 30-31.	Insufficient data
194. Vande Casteele N, Peeters M, Compernolle G, et al. TNF-responsive cellular based assay reveals neutralizing capacity of anti-adalimumab antibodies in crohn's disease and ulcerative colitis patients. Gastroenterology 2014;146(5 Suppl):S-242 doi: http://dx.doi.org/10.1016/S0016-5085%2814%2960852-0[published Online First: Epub Date]|.	Insufficient data
195. Vande Casteele, N., A. Gils, G. Compernolle, V. Ballet, M. Peeters, K. Van Steen, S. Simoens, G. Van Assche, M. Ferrante, S. Vermeire and P. Rutgeerts (2013). "Drug level versus clinically based dosing of infliximab maintenance therapy in IBD: Final results of the randomized controlled taxit trial." Inflamm Bowel Dis 19: S2-S3.	Superseded by full text
196. Vande Casteele, N., G. Compernolle, V. Ballet, G. Van Assche, A. Gils, S. Vermeire and P. J. Rutgeerts (2012). "Results on the optimisation phase of the prospective controlled trough level adapted infliximab treatment (TAXIT) trial." Gastroenterology 1): S211-S212	Superseded by full text
197. Vande Casteele, N., G. Compernolle, V. Ballet, G. Van Assche, A. Gils, S. Vermeire and P. Rutgeerts (2012). "Individualised infliximab treatment using therapeutic drug monitoring: A prospective controlled Trough level Adapted infliXImab Treatment (TAXIT) trial." Journal of Crohn's and Colitis 6: S6.	Duplicate
198. Vande Casteele, N., K. Drake, S. Hauenstein, B. G. Levesque, S. Singh and W. Sandborn (2014). "Infliximab and antibody to infliximab concentrations in 7,613 patients shows indication for testing, association with loss of response and provides new insights into binding characteristics of anti-drug antibodies." Gastroenterology 1): S-242.	Insufficient data
199. Vande Casteele, N., L. Cuypers, S. Singh, L. Ohrmund, S. Hauenstein, G. Van Assche, P. J. Rutgeerts, A. Gils and S. Vermeire (2012). "Antibodies to infliximab can either be persistent or transient: A retrospective case-control study in ibd patients treated with infliximab maintenance therapy." Gastroenterology 1): S114.	Insufficient data

200. Vande Casteele, N., L. Cuypers, S. Singh, S. Hauenstein, L. Ohrmund, E. Chuang, P. Rutgeerts, A. Gils and S. Vermeire (2012). "Transient versus sustained antibodies to infliximab: Possibility to overcome low titer antibody responses by dose optimisation." Journal of Crohn's and Colitis 6: S110.	Insufficient data
201. Vande Casteele, N., M. Peeters, M. Ferrante, G. Compernolle, G. Van Assche, S. Vermeire and A. Gils (2014). "Functional cellular based assay reveals neutralising anti-drug antibodies in IBD patients treated with maintenance adalimumab." Journal of Crohn's and Colitis 8: S268-S269.	Duplicate
202. Vaughn, B. P., M. Martinez-Vazquez, V. Patwardhan, A. C. Moss, W. J. Sandborn and A. S. Cheifetz (2014). "A pilot study of optimized monotherapy with infliximab for patients with inflammatory bowel disease." Gastroenterology 1): S-55.	Superseded by full paper
203. Vaughn, B. P., M. Martinez-Vazquez, V. Patwardhan, A. C. Moss, W. J. Sandborn and A. S. Cheifetz (2014). "Prospective therapeutic drug monitoring to optimizing infliximab (IFX) maintenance therapy in patients with inflammatory bowel disease (IBD)." Gastroenterology 1): S-54.	Superseded by full paper
204. Vaughn, B., M. Matinez-Vazquez and A. Cheifetz (2013). "Infliximab dosing changes based on trough levels in a cohort of IBD patients in clinical remission." Inflamm Bowel Dis 19: S59.	Insufficient data
205. Velayos, F. S., S. Sheibani, S. Lockton, S. Hauenstein, S. Singh, J. P. Terdiman and U. Mahadevan (2013). "Prevalence of antibodies to adalimumab (ATA) and correlation between ATA and low serum drug concentration on CRP and clinical symptoms in a prospective sample of IBD patients." Gastroenterology 1): S91.	Insufficient data
206. Veres, G., J. L. Kaplan, E. De Greef, E. Chuang, D. Szabo, K. Molnar, L. Ohrmund, S. Hauenstein, S. Singh, A. Arato, G. Veereman and H. S. Winter (2012). "New assay to detect infliximab levels and anti-infliximab antibodies from a single serum sample is useful in measuring efficacy of treatment with infliximab in children with IBD." Gastroenterology 1): S386.	Insufficient data
207. Wang SL, Ohrmund L, Hauenstein S, et al. Evaluation of a novel homogeneous mobility shift assay for the measurement of human antibodies-To-Infliximab and infliximab levels in Patient serum. Am J Gastroenterol 2011;106:S475-6 doi: http://dx.doi.org/10.1038/ajg.2011.336_9[published Online First: Epub Date]|.	Insufficient data
208. Wang SL, Ohrmund L, Singh S. Measurement of human anti-chimeric antibodies (Haca) and infliximab levels in patient serum using a novel homogeneous assay. Gastroenterology 2010;1):S684-5	Insufficient data
209. Wang, S. L., L. Ohrmund, S. Hauenstein, J. Salbato, R. Reddy, P. Monk and S. Lockton (2011). "Evaluation of a novel homogeneous mobility shift assay for the measurement of human antibodies-to-infliximab and infliximab levels in patient serum." Arthritis and Rheumatism 1).	Duplicate
210. Wang, S. L., S. Hauenstein, L. Ohrmund, R. Shringarpure, D. C. Wolf, I. A. Diab, J. Salbato, R. Reddy, K. McCowen, S. Shah, S. Lockton, E. Chuang and S. Singh (2012). "Influence of trough serum drug level and immunogenicity on the lack of response to adalimumab therapy in inflammatory bowel disease patients." Arthritis and Rheumatism 64: S819-S820.	Insufficient data
211. Wang, S. L., S. Hauenstein, L. Ohrmund, R. Shringarpure, D. Wolf, I. Diab, J. Salbato, R. Reddy, K. McCowen, S. Shah, S. Lockton, E. Chuang and S. Singh (2012). "Influence of trough serum drug level and immunogenicity on the lack of response to adalimumab therapy in IBD patients presidential poster." American Journal of Gastroenterology 107: S680.	Duplicate
212. Ward MG, Kariyawasam VC, Mogan SB, et al. Clinical utility of measuring adalimumab trough levels and antibodies to adalimumab in patients with inflammatory bowel diseases. J Gastroenterol Hepatol 2013;28:100-01 doi: http://dx.doi.org/10.1111/jgh.12365-6[published Online First: Epub Date]|.	Insufficient data

213. Wolf, D. C., S. Hauenstein, S. Lockton and S. Singh (2013). "Mechanisms of loss of response to adalimumab in crohn's disease." Gastroenterology 1): S775	Insufficient data
214. Wolf, D. C., S. Lockton, S. Hauenstein, S. Carroll, S. Singh and E. Chuang (2013). "A multi-center observational study in community gastroenterology practices evaluating the clinical usage of testing for serum levels of infliximab and antibodies to infliximab." Gastroenterology 1): S423.	Insufficient data
215. Wolf, D., R. Shringarpure, S. Lockton, R. Corey, S. Woods, H. Aguilar and E. Chuang (2012). "Clinical experience with measurement of serum infliximab and antibodies to infliximab using a new homogenous mobility shift assay: Results of a multi-center observational study." American Journal of Gastroenterology 107: S658.	Insufficient data
216. Yamada, A., K. Sono, K. Takeuchi and Y. Suzuki (2013). "Clinical and basic studies to understand factors associated with the loss of response to infliximab in patients with Crohn's disease." Journal of Crohn's and Colitis 7: S239.	Insufficient data
217. Yanai, H., L. Lichtenstein, A. Assa, Y. Mazor, B. Weiss, A. Levine, Y. Ron, U. Kopylov, Y. Bujanover, Y. Rosenbach, B. Ungar, A. R. Eliakim, Y. Chowers, R. Shamir, G. Fraser, I. Dotan and S. Ben-Horin (2014). "Anti-TNF and anti-drug antibodies levels predict the ouCTomes of interventions after loss of response to adalimumab and infliximab." Gastroenterology 1): S-381.	Insufficient data
218. Yarur AJ, Deshpande AR, Sussman DA, et al. Serum adalimumab levels and antibodies correlate with endoscopic intestinal inflammation and inflammatory markers in patients with inflammatory bowel disease. Gastroenterology 2013;144(5 Suppl):S774-5	Insufficient data
219. Yarur, A., J. P. Trivella, D. A. Sussman, K. Drake, J. S. Barkin, S. Hauenstein, A. R. Deshpande, M. A. Quintero, S. Singh and M. T. Abreu (2014). "Anti-tumor necrosis factor drug levels and anti-bodies are associated with crohn's disease recurrence at the level of the ileo-colonic anastomosis after ileal resection." Gastroenterology 1): S243-S244.	Insufficient data
220. Yarur, A., K. Drake, M. Kubiliun, R. M. Dauer, D. A. Sussman, S. Hauenstein, M. A. Quintero, S. Singh, J. S. Barkin and M. T. Abreu (2014). "Anti-tumor necrosis factor levels are not associated with intestinal extent of mucosal inflammation in patients with inflammatory bowel diseases." Gastroenterology 1): S-244.	Insufficient data
221. Zelinkova, Z., M. P. Peppelenbosch, A. Van Liere-Baron, C. De Haar and C. J. Van Der Woude (2011). "Naturally-occurring autoantibodies against TNF-alpha are present in sera of inflammatory bowel disease patients and influence the response to adalimumab." Gastroenterology 1): S62.	Insufficient data

CD - Crohn's disease; RA rheumatoid arthritis

For peer review only

Supplement 5 Drug cut-offs for predicting loss of or lack of regaining response

Supplement 5 Table 1 Drug cut-offs defined by ROC analysis in included studies using drug level as predictor of loss of or lack of regaining response (by assay type and drug)

Reference	Cut-off in µg/ml	Performance measures				AUC (95% CI)	Clinical marker	Drug	Assay
		Sens	Spec	PPV	NPV				
Bortlik 2013[41]	3	0.70	0.62	0.41	0.84	0.70 (0.57-0.83)	Sustained response (no treatment failure or drug intolerance, no surgery, IS introduction, steroids or Infliximab increase)	IFX	ELISA
Cornillie 2014[42]	3.5	0.64	0.78	0.56	0.83	0.75	Sustained response (CDAI score change)	IFX	ELISA
Steenholdt 2011[33]	0.5<br><br>2.2 (TL week 14)	0.86<br><br>0.79	0.85<br><br>0.94	NR	NR	0.93 (0.85-1.0)<br><br>0.93 (SE 0.04)	Maintained response (good response to induction therapy at 0, 2 and 6 weeks followed by good response to maintenance therapy)	IFX	RIA
Chiu 2013[47]	No Adalimumab concentration identified associated with clinical remission at any time point so clinical utility of measuring Adalimumab concentrations was difficult to assess	NR	NR	NR	NR	Week 4: 0.51<br>Week 24: 0.58<br>Week 56: 0.57	Clinical remission (CDAI <150)	ADA	ELISA
Imaeda 2014[36]	5.9	0.67	0.92	NR	NR	0.83 (0.80-0.95)	CRP ≤0.3mg/dL	ADA	ELISA
Mazor 2014[37]	5.85	0.68	0.71	NR	NR	0.75 (0.66-0.84)	Remission according to 2 physicians' assessment	ADA	ELISA
Roblin 2014[38]	4.85<br>4.9	0.81<br>0.66	0.67<br>0.85	0.84<br>0.88	0.57<br>0.51	0.73<br>0.77	Clinical remission (CDAI <150)<br>MH (disappearance of all ulcerations on endoscopy)	ADA	ELISA
Frederiksen 2014[39]	14.5<br>0.35<br>6.85	1.00<br>0.50<br>0.69	0.12<br>0.96<br>0.69	0.41<br>0.89<br>0.58	1.00<br>0.76<br>0.78	0.77 (0.62-0.93)	LOR (physician's global assessment)	ADA	RIA

For peer review only

Supplement 5 Table 2 Drug cut-offs in included studies not reporting a ROC analysis and using drug level as predictor of loss of or lack of regaining response (by assay type)

Reference	Cut-off in µg/ml	Source of cut-off	Drug	Assay
Hibi 2014[43]	1	Maser 2006[46]	IFX	ELISA
Imaeda 2012[26]	0.66	95th percentile value from 35 patients who had never received Infliximab	IFX	ELISA
Kopylov 2012[28]	Unclear	Unclear	IFX	ELISA
Maser 2006[46]	1.4	Unclear	IFX	ELISA
Yanai 2012[44] abstract	1	Unclear	IFX	ELISA
Ben Bassat 2013[45] abstract	2	Derived from data not pre-specified	IFX	HMSA
Ainsworth 2008[22]	0.5	Derived from data not pre-specified	IFX	RIA
Steenholdt 2014[23]	0.5	Steenholdt 2011[33]	IFX	RIA

Supplement 5 Table 3 Additional studies reporting drug cut-offs derived by ROC analysis but not reporting sufficient 2x2 data for using drug level as predictor of loss of or lack of regaining response (by assay type and drug)

Reference	Cut-off in µg/ml	Performance measures				AUC (95% CI)	Clinical marker	Drug	Assay
		Sens	Spec	PPV	NPV				
Goldberg R, Beswick L, Van Langenberg D, et al. Journal of Crohn's and Colitis 2014;8:S223 Abstract	3	0.90	0.37	NR	NR	0.75	Disease activity (physicians global assessment and CRP levels)	IFX	ELISA
Imaeda H, Bamba S, Takahashi K, et al. J Gastroenterol 2014;49(4):674-82	0.6<br>1.0<br>1.1<br>4.0	0.73<br>0.67<br>0.72<br>0.71	0.62<br>0.71<br>0.56<br>0.70	NR<br>NR<br>NR<br>NR	NR<br>NR<br>NR<br>NR	0.67 (0.60-0.81)<br>0.72 (0.50-0.73)<br>0.63 (0.55-0.65)<br>0.63 (0.56-0.70)	CRP ≤0.3mg/dL<br>Serum albumin (≥ 4.0mg/dL)<br>FC (≤ 300µg/g)<br>MH (Rutgeerts scoring system 0 or 1)	IFX	ELISA
Marits P, Landucci L, Sundin U, et al. Journal of Crohn's & colitis 2014;8(8):881-9	4.1	0.87	0.44	NR	NR	0.74 (SE 0.037)	Remission (HBI <5 and CRP < 3 mg/l)	IFX	ELISA
Nagore D, Ruiz Del Agua	0.8	0.86	0.75	NR	NR	0.86 (0.76-0.96)	Active disease	IFX	ELISA

For peer review only

Reference	Cut-off in µg/ml	Performance measures				AUC (95% CI)	Clinical marker	Drug	Assay
		Sens	Spec	PPV	NPV				
A, Pascual J, et al. Therapeutic (TU1325). Gastroenterology 2015;148(4 Suppl 1):S-860									(Promonitor)
Pallagi-Kunstar E, Farkas K, Szepes Z, et al. World J Gastroenterol 2014;20(17):5031-5	3.01	NR	NR	NR	NR	NR	Detecting anti-drug antibodies	IFX	ELISA
Paul S, Tedesco ED, Marotte H, et al.. Gastroenterology 2012;142(5 Suppl):S354	2	0.76	0.82	NR	NR	0.60	Remission (CDAI score <150)	IFX	ELISA
Paul S, Del Tedesco E, Marotte H, et al.. Inflamm Bowel Dis 2013;19(12):2568-76	0.5 (trough after optimisation minus trough before optimisation)	0.88	0.76	0.78	0.86	0.91 (0.83-1.0)	Mucosal healing (FC <250µg/g)	IFX	ELISA (
Singh N, Rosenthal CJ, Melmed GY, et al. Inflamm Bowel Dis 2014;20(10):1708-13	4<br>7	0.53<br>0.33	0.75<br>1.00	0.76<br>1.00	0.52<br>0.50	0.64 (0.51-0.75)<br>0.67 (0.58-0.75)	Week 14 Infliximab levels as predictor of week 54 clinical remission according to CDAI	IFX	ELISA
Baert F, Drobne D, Gils A, et al. Clin Gastroenterol Hepatol 2014;12(9):1474-81	2 (after re-exposure to Infliximab)	NR	NR	NR	NR	0.76 (0.62-0.90)	Long term response (clinical assessment [HBI] and CRP levels[<3mg/l])	IFX	HMSA
Levesque BG, Greenberg GR, Zou G, et al. Aliment Pharmacol Ther 2014;39(10):1126-35	3	NR	NR	NR	NR	NR	Disease activity at week 8 (≥70 point increase in CDAI and CRP >5µg/l)	IFX	HMSA
Vande Casteele N, Gils A, Singh S, et al. Am J Gastroenterol 2013;108(6):962-71	13 (TL week 6)	0.72	0.81	NR	NR	0.87 (SE 0.06)	anti-drug antibody formation	IFX	HMSA
Feagan BG, Singh S, Lockton S, et al. Gastroenterology 2012;142(5 Suppl):S-114 Abstract	3	NR	NR	NR	NR	0.74	Disease activity	IFX	HPLC based fluid phase assay
Goldberg R, Beswick L, Van Langenberg D, et al. Journal of Crohn's and	3	0.83	0.63	NR	NR	0.8	Disease activity (physicians global assessment and CRP levels)	ADA	ELISA

For peer review only

Reference	Cut-off in μg/ml	Performance measures				AUC (95% CI)	Clinical marker	Drug	Assay
		Sens	Spec	PPV	NPV				
Colitis 2014;8:S223 Abstract									
Karmiris K, Paintaud G, Noman M, et al. Gastroenterology 2009;137(5):1628-40	0.33	0.95	NR	0.81	NR	NR	Sustained clinical benefit (patient reporting lasting control of disease with possible dose escalation)	ADA	ELISA
Ward MG, Kariyawasam VC, Mogan SB, et al. J Gastroenterol Hepatol 2013;28:100-01 Abstract	4.9	0.83	0.65	NR	NR	0.75	Remission	ADA	LISA
arur AJ, Deshpande AR, Sussman DA, et al. Gastroenterology 2013;144(5 Suppl):S774-5 Abstract	5	NR	NR	NR	NR	0.71	Elevation of CRP	ADA	HMSA
Mazor Y, Kopylov U, Hur DB, et al. Gastroenterology 2013;144(5 Suppl):S-778 Abstract	5	NR	NR	NR	NR	0.77 (0.67-0.86)	Clinical response and normal CRP	ADA	NR

For peer review only

Supplement 6 Summary of quality assessment results using the QUADAS-2 tool with index questions adapted to the review for studies comparing performance of different tests

Supplement 6 Table 1Tabular presentation of QUADAS-2 results

Study	RISK OF BIAS				APPLICABILITY CONCERNS		
	PATIENT SELECTION	INDEX TEST	REFERENCE STANDARD	FLOW AND TIMING	PATIENT SELECTION	INDEX TEST	REFERENCE STANDARD
Ainsworth 2008[22]	☹	☺	☹	☺	☺	☺	☹
Baert 2014[20]	☺	☺	☹	☺	☺	☺	☹
Ben-Horin 2011[34]	☹	☺	☹	☺	☺	☹	☹
Ben-Horin 2012[17]	☹	☺	☹	☺	☺	☺	☹
Bortlik 2013[41]	☹	☺	☹	☺	☺	☺	☹
Candon 2005[18]	☹	☺	☺	☺	☺	☺	☺
Chiu 2013[47]	☹	☹	☺	☺	☺	☹	☺
Cornillie 2014 [42]	☹	☹	☺	☹	☺	☹	☺
Farrell 2003[24]	☺	☺	☹	☺	☺	☺	☹
Frederiksen 2014[39]	☹	☺	☹	☺	?	☺	☹
Hanauer 2004[25]	☹	☹	☺	☹	☺	☹	☺
Hibi 2014[43]	☹	☺	☺	☺	☺	☺	☺
Imaeda 2012[26]	☹	☺	☺	☺	☺	☺	☺
Imaeda 2014[36]	☹	☺	☺	☺	☺	☺	☺
Kopylov 2012[28]	☹	☺	☹	☺	☺	☺	☹
Maser 2006[46]	☺	☺	☺	☺	☺	☺	☺
Mazor 2014 [37]	☺	☺	☺	☺	☺	☺	☺
Pariente 2012[19]	☹	☺	?	☺	☹	☺	?
Roblin 2014[38]	☹	☺	☺	☺	☺	☺	☺
Steenholdt 2011[33]	☹	☺	☹	☺	☺	☺	☹
Steenholdt 2013[31]	☹	☺	☹	☹	☺	☺	☹
Steenholdt 2014[23]	☺	☺	☺	☺	☺	☺	☺
Van Casteele 2013[21]	☹	☺	☺	☺	☹	☺	☺
West 2008[40]	☹	☺	☹	☺	☺	☺	☹

☺Low Risk ☹High Risk ? Unclear Risk

Supplement 6 Figure 1 Graphical summary presentation of QUADAS-2 quality assessment results

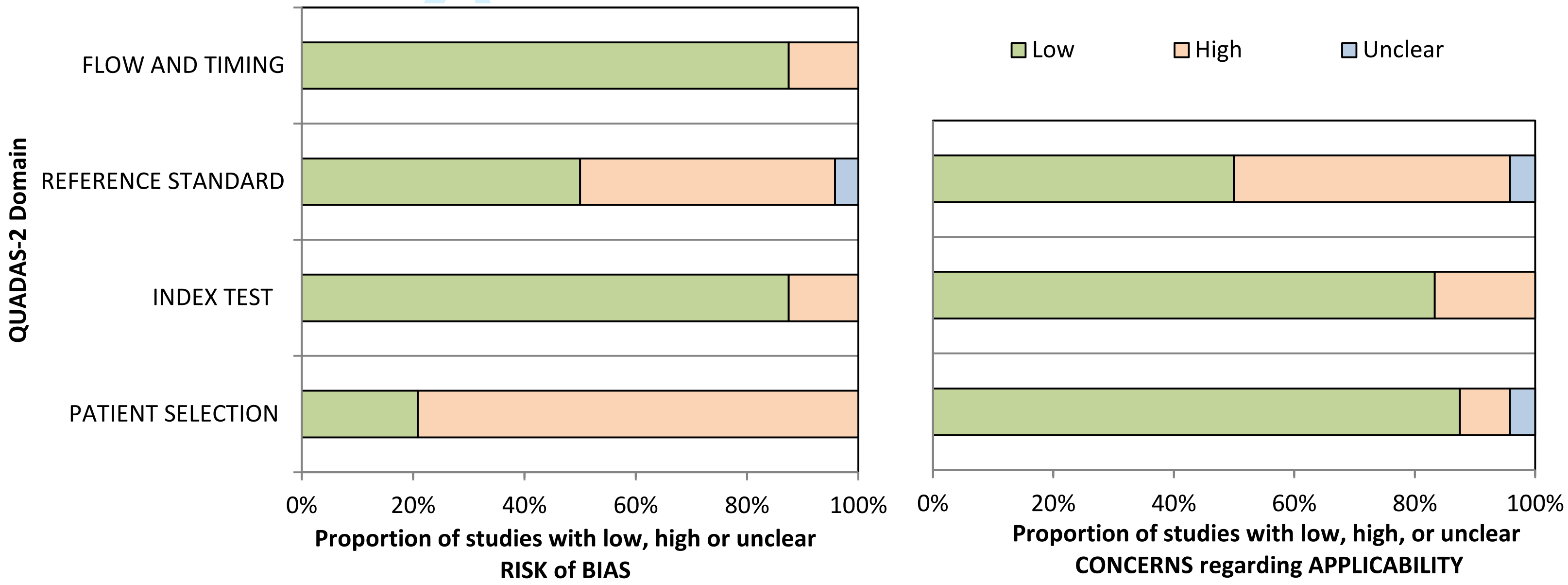

Supplement 7 Results of hierarchical meta-analysis of included studies

Supplement 7 Table 1 Test accuracy statistics from hierarchical meta-analyses

Trough Infliximab level as predictor of loss or absence of response				
Studies included	parameter	Point estimate	95% LCI	95% UCI
all 11 studies	Sens	0.657232	0.546288	0.753299
all 11 studies	Spec	0.80625	0.744166	0.85618
all 11 studies	DOR	7.978975	4.119972	15.45254
all 11 studies	LR+	3.392169	2.35152	4.893351
all 11 studies	LR-	0.425139	0.305104	0.592398
all 11 studies	1/LR-	2.352175	1.688056	3.277573
responder populations only	Sens	0.681452	0.592117	0.759178
responder populations only	Spec	0.790873	0.723301	0.845468
responder populations only	DOR	8.090128	4.353039	15.03551
responder populations only	LR+	3.258549	2.287802	4.641198
responder populations only	LR-	0.402781	0.298559	0.543385
responder populations only	1/LR-	2.482739	1.840315	3.349423
ELISA studies only	Sens	0.652104	0.564027	0.730877
ELISA studies only	Spec	0.789041	0.691592	0.861849
ELISA studies only	DOR	7.010794	3.450232	14.24578
ELISA studies only	LR+	3.091133	1.959085	4.877331
ELISA studies only	LR-	0.440911	0.329778	0.589495
ELISA studies only	1/LR-	2.268033	1.696367	3.032348
**Trough level of antibodies to Infliximab as predictor of loss or absence of response**				
Studies included	parameter	Point estimate	95% LCI	95% UCI
all 20 studies	Sens	0.559745	0.444812	0.668611
all 20 studies	Spec	0.792243	0.688105	0.868267
all 20 studies	DOR	4.848283	2.519589	9.329239
all 20 studies	LR+	2.694226	1.72293	4.213088
all 20 studies	LR-	0.555707	0.426575	0.72393
all 20 studies	1/LR-	1.799509	1.38135	2.344251
all studies minus outliers*	Sens	0.597	0.477	0.707
all studies minus outliers*	Spec	0.807	0.742	0.859
all studies minus outliers*	DOR	6.183	3.805	10.050
all studies minus outliers*	LR+	3.088	2.311	4.127
all studies minus outliers*	LR-	0.500	0.381	0.655

For peer review only

all studies minus outliers	1/LR-	2.002	1.528	2.623
responder populations only	Sens	0.570	0.445	0.687
responder populations only	Spec	0.849	0.787	0.896
responder populations only	DOR	7.460	4.544	12.250
responder populations only	LR+	3.778	2.722	5.244
responder populations only	LR-	0.506	0.388	0.660
responder populations only	1/LR-	1.974	1.514	2.574
ELISA studies only	Sens	0.482	0.355	0.611
ELISA studies only	Spec	0.880	0.841	0.911
ELISA studies only	DOR	6.830	3.872	12.050
ELISA studies only	LR+	4.022	2.805	5.768
ELISA studies only	LR-	0.589	0.459	0.755
ELISA studies only	1/LR-	1.698	1.324	2.178
**Trough Adalimumab level as predictor of loss or absence of response**				
	Parameter	Point estimate	95% LCI	95% UCI
All 5 studies	Sens	0.543476	0.246586	0.812386
All 5 studies	Spec	0.640241	0.325873	0.86758
All 5 studies	DOR	2.118592	0.172646	25.99789
All 5 studies	LR+	1.510665	0.38102	5.989464
All 5 studies	LR-	0.713051	0.229687	2.213631
All 5 studies	1/LR-	1.402424	0.451747	4.353753
All studies minus Chiu	Parameter	Point estimate	95% LCI	95% UCI
All studies minus Chiu	Sens	0.684	0.591	0.764
All studies minus Chiu	Spec	0.786	0.643	0.883
All studies minus Chiu	DOR	7.971	3.646	17.428
All studies minus Chiu	LR+	3.201	1.822	5.623
All studies minus Chiu	LR-	0.402	0.297	0.542
All studies minus Chiu	1/LR-	2.490	1.844	3.363
**Trough level of antibodies to Adalimumab as predictor of loss or absence of response**				
	Parameter	Point estimate	95% LCI	95% UCI
All 6 studies	Sens	0.471206	0.2903357	0.66
All 6 studies	Spec	0.915467	0.7939073	0.968
All 6 studies	DOR	9.65022	4.387759	21.22
All 6 studies	LR+	5.574189	2.646268	11.74
All 6 studies	LR-	0.577623	0.4208713	0.793

For peer review only

All 6 studies	1/LR-	1.731233	1.261422	2.376
	Parameter	Point estimate	95% LCI	95% UCI
All studies minus Mazor	Sens	0.542264	0.3611645	0.713
All studies minus Mazor	Spec	0.884874	0.7444581	0.953
All studies minus Mazor	DOR	9.105532	3.764526	22.02
All studies minus Mazor	LR+	4.710191	2.221639	9.986
All studies minus Mazor	LR-	0.517289	0.361111	0.741
All studies minus Mazor	1/LR-	1.933156	1.349505	2.769

Sens = sensitivity; Spec = specificity; DOR = diagnostic odds ratio; LR+ = positive likelihood ratio; LR- = negative likelihood ratio; 1/LR- = inverse of negative likelihood ratio.

*Outliers are Ainsworth 2008 and Steenholdt 2014

For peer review only

Supplement 7 Figure 1. Hierarchical meta-analysis of studies of trough levels of antibodies to Adalimumab (upper row) and of Adalimumab (lower row) for predicting loss of response or failure to regain response

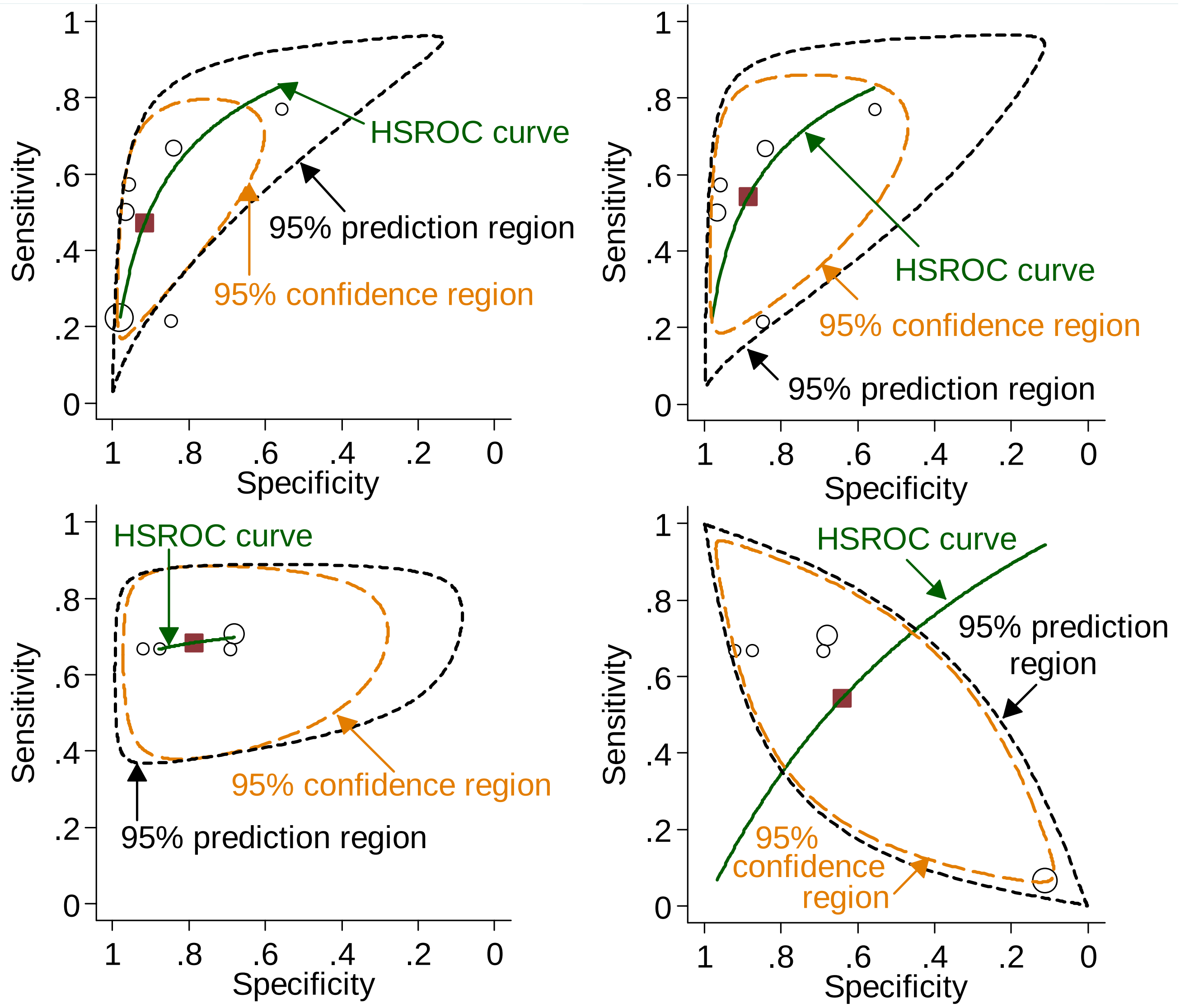

Top Upper left = all anti-Adalimumab antibody studies; upper right = anti-Adalimumab antibody studies but omitting the study of Mazor; lower left Adalimumab studies but omitting the study of patients with secondary loss of response (Chiu); lower right = all Adalimumab studies. The square symbol represents the summary point estimate on the HSROC curve. Mazor was omitted because it was a particularly large and influential study.

Supplement 8 Impact of additional studies on meta-analysis results

Supplement 8 Table 1 Characteristics of additional studies identified by search update

STUDY	DRUG	DIAGNOSIS	RESPONSE/LOR	TEST	RESPONSE MEASURE
Infliximab trough level as predictor of loss of or lack of regaining response					
Levesque 2014 [51]	IFX	CD	LOR	HMSA	≥ 70 CDAI increase
Reinisch 2016 [53]	IFX	CD	LOR	ELISA	Mucosal healing
Ungar 2016 [52]	IFX	CD	LOR	HMSA	Mucosal healing
Adalimumab trough level as predictor of loss of or lack of regaining response					
Bodini 2015 [54]	ADA	CD	LOR	HMSA	> 7 HBI
Morita 2016 [57]	ADA	CD	LOR	ELISA	Mucosal healing
Ungar 2016 [52]	ADA	CD	LOR	HMSA	Mucosal healing
Yarur 2016 [56]	ADA	IBD ~0.89 CD	LOR	HMSA	Mucosal healing
Zittan 2016 [55]	ADA	CD	LOR	HMSA	Mucosal healing

Diagnosis = study patient population; LOR = patients with loss of response; Response measure = method used for defining clinical response; ADA = Adalimumab; IFX = Infliximab; CD = Crohn's disease; IBD = inflammatory bowel disease; ELISA = enzyme linked immunoassay; HBI = Harvey-Bradshaw Index; HMSA= Homogenous Mobility Shift Assay; CDAI = Crohn's disease activity index score.

Sensitivity and specificity pairs for the new studies are shown in Supplement 8 Figure 1 together with those for earlier studies.

For peer review only

Infliximab trough levels

Study	TP	FP	FN	TN	assay	POP	RES	Sensitivity (95% CI)	Specificity (95% CI)
Reinisch	38	29	15	70	ELISA	LR	MH	0.72 [0.58, 0.83]	0.71 [0.61, 0.79]
Levesque	178	11	113	25	HMSA	LR	CDAI	0.61 [0.55, 0.67]	0.69 [0.52, 0.84]
Ungar 2016	38	15	7	7	HMSA	LR	MH	0.84 [0.71, 0.94]	0.32 [0.14, 0.55]
Ainsworth 2008	7	1	1	18	RIA	LR	PJ	0.88 [0.47, 1.00]	0.95 [0.74, 1.00]
Steenholdt 2014	10	7	21	31	RIA	LR	CDAI	0.32 [0.17, 0.51]	0.82 [0.66, 0.92]
Bortlik 2013	16	23	7	38	ELISA	R	PJ	0.70 [0.47, 0.87]	0.62 [0.49, 0.74]
Cornillie 2014	14	11	8	38	ELISA	R	CDAI	0.64 [0.41, 0.83]	0.78 [0.63, 0.88]
Hibi 2014	8	4	7	22	ELISA	R	CDAI	0.53 [0.27, 0.79]	0.85 [0.65, 0.96]
Imaeda 2012	9	8	8	33	ELISA	R	CDAI	0.53 [0.28, 0.77]	0.80 [0.65, 0.91]
Kopylov 2012	21	2	9	31	ELISA	R	PJ	0.70 [0.51, 0.85]	0.94 [0.80, 0.99]
Yanai 2012	7	10	7	16	ELISA	R	PJ	0.50 [0.23, 0.77]	0.62 [0.41, 0.80]
Ben-Bassat 2013	50	29	10	145	HMSA	R	HBI	0.83 [0.71, 0.92]	0.83 [0.77, 0.89]
Steenholdt 2011	18	7	3	41	RIA	R	PJ	0.86 [0.64, 0.97]	0.85 [0.72, 0.94]
Maser 2006	22	11	13	44	UC	R	HBI	0.63 [0.45, 0.79]	0.80 [0.67, 0.90]

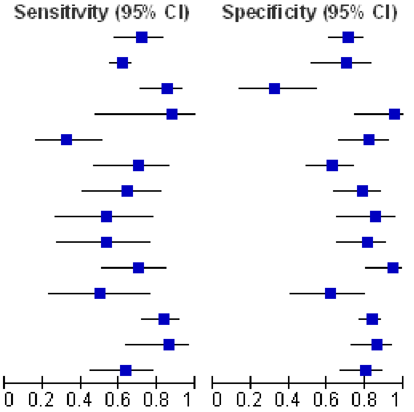

Adalimumab trough levels

Study	TP	FP	FN	TN	assay	POP	RES	Sensitivity (95% CI)	Specificity (95% CI)
Roblin 2014	16	2	8	14	ELISA	R	MH	0.67 [0.45, 0.84]	0.88 [0.62, 0.98]
Mazor 2014	48	16	20	34	ELISA	R	PJ CRP	0.71 [0.58, 0.81]	0.68 [0.53, 0.80]
Imaeda 2014	10	2	5	23	ELISA	R	CRP	0.67 [0.38, 0.88]	0.92 [0.74, 0.99]
Frederiksen 2014	12	9	6	20	RIA	R	PJ BM	0.67 [0.41, 0.87]	0.69 [0.49, 0.85]
Chiu 2013	7	7	56	98	ELISA	LR	CDAI100	0.11 [0.05, 0.22]	0.93 [0.87, 0.97]
Bodini 2015	13	2	0	8	ELISA	LR	HBI	1.00 [0.75, 1.00]	0.80 [0.44, 0.97]
Morita 2016	24	4	4	10	ELISA	LR	MH	0.86 [0.67, 0.96]	0.71 [0.42, 0.92]
Ungar 2016	36	22	6	14	ELISA	LR	MH	0.86 [0.71, 0.95]	0.39 [0.23, 0.57]
Yarur 2016	31	11	6	29	ELISA	LR	MH	0.84 [0.68, 0.94]	0.72 [0.56, 0.85]
Zittan 2016	19	3	6	32	ELISA	LR	MH	0.76 [0.55, 0.91]	0.91 [0.77, 0.98]

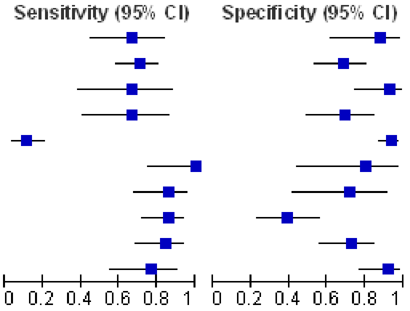

Supplement 8 Figure 1 Paired forest plots for trough anti-TNF levels for predicting loss of response or failure to regain response to Infliximab (upper, 3 new studies at the top) and Adalimumab (lower, 5 new studies at the bottom);
RES = criterion for determining clinical response, POP = study patient population, RIA = radioimmunoassay, HMSA = homogeneous mobility shift assay, ELISA = enzyme linked immunoassay, LR = patients with loss of response, R = patients with response, UC = unclear, PJ BM = physicians' judgement and biological measure; PJ = physicians' judgement, HBI = Harvey Bradshaw Index score, CDAI = Crohn's disease activity index score, CRP = C-reactive protein level, MH = mucosal healing

Meta-analysis of Infliximab trough studies

Three new studies were identified reporting test accuracy of infliximab trough levels to predict loss of response bringing the total number of studies available for meta-analysis to 14.[51-53] The meta-analysis summary estimates of test accuracy for the original eleven and of the 14 studies are summarised in Supplement 8 Table 2.

For peer review only - http://bmjopen.bmj.com/site/about/guidelines.xhtml

Supplement 8 Table 2 Test accuracy statistics from hierarchical meta-analyses (infliximab studies)

**Trough Infliximab level as predictor of loss or absence of response**				
Studies included	parameter	SummaryPoint estimate	95% LCI	95% UCI
original 11 studies	Sens	0.657232	0.546288	0.753299
original 11 studies	Spec	0.80625	0.744166	0.85618
original 11 studies	DOR	7.978975	4.119972	15.45254
original 11 studies	LR+	3.392169	2.35152	4.893351
original 11 studies	LR-	0.425139	0.305104	0.592398
original 11 studies	1/LR-	2.352175	1.688056	3.277573
**Updated analysis including three new studies**				
all 14 studies	Sens	0.674018	0.587579	0.750047
all 14 studies	Spec	0.774693	0.696482	0.837453
all 14 studies	DOR	7.109369	4.225833	11.96051
all 14 studies	LR+	2.991547	2.163908	4.135736
all 14 studies	LR-	0.420789	0.325131	0.544592
all 14 studies	1/LR-	2.376486	1.836237	3.075685
**Change in summary estimates after including 3 new studies**				
	Sens	0.016786	0.041291	-0.00325
	Spec	-0.03156	-0.04768	-0.01873
	DOR	-0.86961	0.105861	-3.49203
	LR+	-0.40062	-0.18761	-0.75762
	LR-	-0.00435	0.020027	-0.04781
	1/LR-	0.024311	0.148181	-0.20189
Sens = sensitivity; Spec = specificity; DOR = diagnostic odds ratio; LR+ = positive likelihood ratio; LR- = negative likelihood ratio; 1/LR- = inverse of negative likelihood ratio.				

Adding the three new studies has very little impact on the meta-analysis summary test statistic estimates or upon their associated uncertainty. Figure 2 shows the summary ROC plots for the 11 and 14 studies.

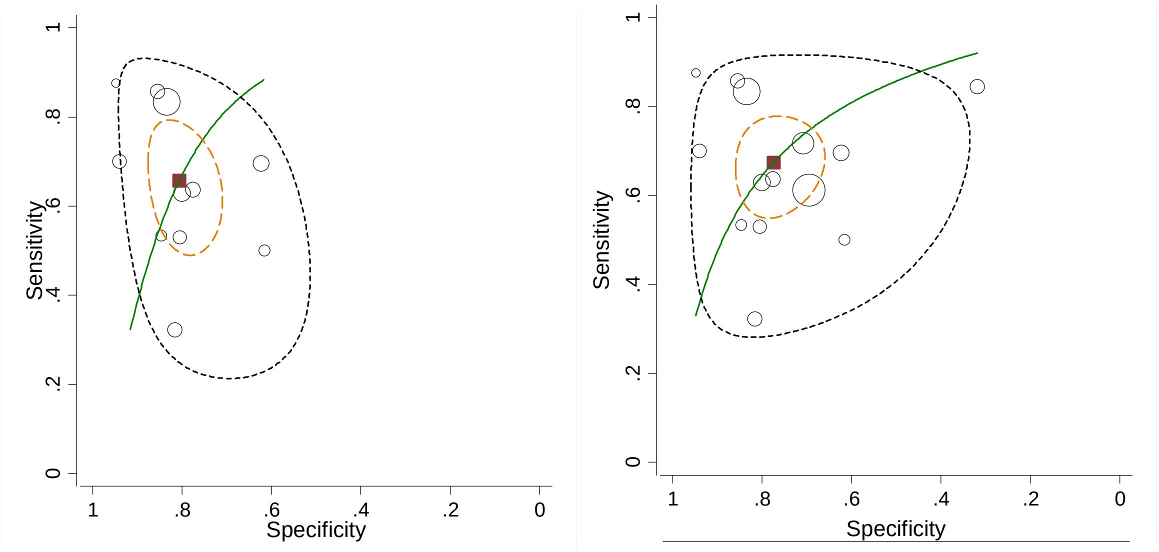

Supplement 8 Figure 2 Summary ROC plots for 11 (left) and 14 (right) studies of Infliximab trough levels

Adalimumab trough studies

Five new studies were identified reporting test accuracy of adalimumab trough levels to predict loss of response,[54-57] thereby bringing the total number of studies available for meta-analysis to nine. The meta-analysis summary estimates for the original four and for the nine studies are summarised in Supplement 8 Table 3.

Supplement 8 Table 3 Test accuracy statistics from hierarchical meta-analyses (Adalimumab studies)

**Trough Infliximab level as predictor of loss or absence of response**				
Studies included	parameter	SummaryPoint estimate	95% LCI	95% UCI
original 4 studies	Sens	0.684251	0.5914862	0.7643434
original 4 studies	Spec	0.7862228	0.6427244	0.8826122
original 4 studies	DOR	7.969987	3.64723	17.41615
original 4 studies	LR+	3.200767	1.823276	5.618956
original 4 studies	LR-	0.4016025	0.2973622	0.5423841
original 4 studies	1/LR-	2.490025	1.843712	3.362902
**Updated analysis including three new studies**				
all 9 studies	Sens	0.6357	0.547669	0.715498
all 9 studies	Spec	0.710633	0.591235	0.806565
all 9 studies	DOR	4.285374	1.929981	9.515341
all 9 studies	LR+	2.196862	1.378996	3.499796
all 9 studies	LR-	0.512642	0.363406	0.723164
all 9 studies	1/LR-	1.950679	1.382813	2.751747
**Change in summary estimates after including 5 new studies**				
	Sens	-0.04855	-0.04382	-0.04885
	Spec	-0.07559	-0.05149	-0.07605
	DOR	-3.68461	-1.71725	-7.90081
	LR+	-1.00391	-0.44428	-2.11916
	LR-	0.111039	0.066043	0.18078
	1/LR-	-0.53935	-0.4609	-0.61116
Sens = sensitivity; Spec = specificity; DOR = diagnostic odds ratio; LR+ = positive likelihood ratio; LR- = negative likelihood ratio; 1/LR- = inverse of negative likelihood ratio. Note: the outlier study of Chiu 2013_ENREF_47 has been omitted from the analyses				

With the exception of estimated DOR, most summary test statistics remain relatively unaltered by the addition of the five new studies. Introduction of the new studies has somewhat reduced the uncertainty of the estimates. The considerable heterogeneity amongst the studies is evident when comparing summary ROC plots for the four and nine studies (Supplement 8 Figure 3).

For peer review only - http://bmjopen.bmj.com/site/about/guidelines.xhtml

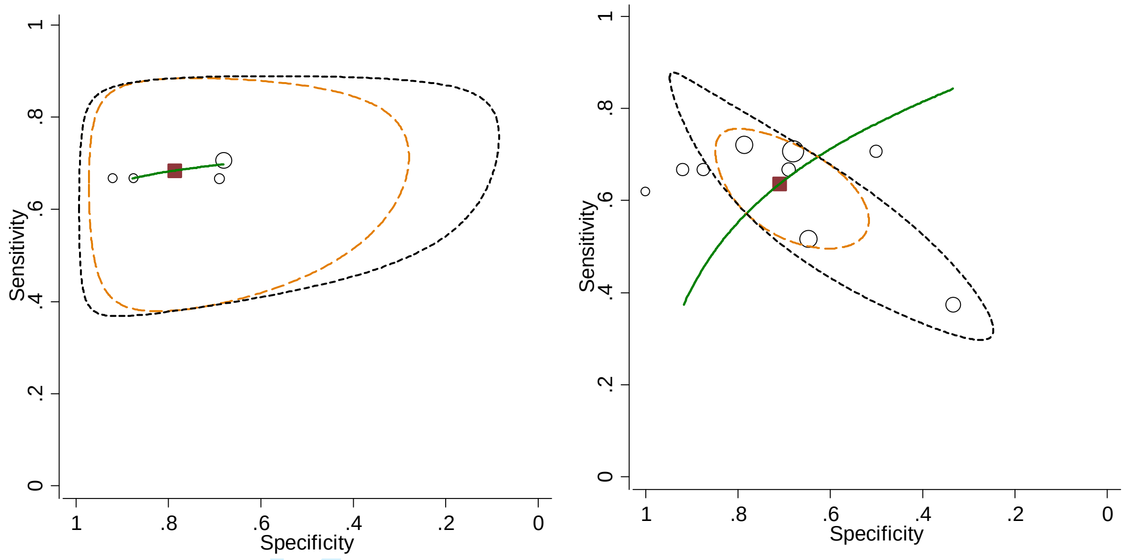

Supplement 8 Figure 3 Summary ROC plots for 4 (left) and 9 (right) studies of Adalimumab trough levels

Note: the outlier study of Chiu 2013 has been omitted from the analyses

For peer review only

For peer review only - http://bmjopen.bmj.com/site/about/guidelines.xhtml

Diagnostic Assessment Report commissioned by the NIHR HTA Programme on behalf of the National Institute for Health and Clinical Excellence – Final Protocol

Title of project

Crohn's disease: Tests for therapeutic monitoring of TNF inhibitors (LISA-TRACKER ELISA kits, TNFα-Blocker ELISA kits, and Promonitor ELISA kits)

Name of External Assessment Group (EAG) and project lead

Produced by: Warwick Evidence

Lead author: Karoline Freeman

Co-authors: Martin Connock
Hema Mistry
Sian Taylor-Phillips
Rachel Court
Alexander Tsertsvadze
Jason Madan
Ngianga-Bakwin Kandala
Ramesh Arasaradnam
Aileen Clarke
Paul Sutcliffe

Correspondence to: Dr Paul Sutcliffe
Associate Professor
Deputy Director for Warwick Evidence
Populations, Evidence and Technologies
Division of Health Sciences
Warwick Medical School
University of Warwick
Coventry CV4 7AL

Tel: 02476 150189

Fax: 02476 528375

Email: p.a.sutcliffe@warwick.ac.uk

Date completed: 29 October 2014

The views expressed in this protocol are those of the authors and not necessarily those of the NIHR HTA Programme. Any errors are the responsibility of the authors. The authors have no conflicts of interest.

For peer review only

For peer review only - http://bmjopen.bmj.com/site/about/guidelines.xhtml

Glossary of terms

Induction therapy	Treatment to induce remission
Maintenance therapy	Treatment to remain in remission
Remission	Period without or only mild symptoms
Biologics or biological therapy	A protein-based drug derived from living cells cultured in a laboratory
Immunosuppressant	A class of drugs that suppress or reduce the strength of the body's immune system
Resection	The removal by surgery of all or part of an organ such as the bowel
Ileostomy	Surgical procedure where the small intestine is diverted through an opening in the abdomen
Intestinal stricture	Narrowing of the intestine due to tissue scaring following inflammation
Fistulas	Channels formed from the digestive system to other parts of the digestive system or different organs
Azathioprine	Immunomodulator
Thiopurines	Group of drugs (purine antimetabolites) including azathioprine, 6-mercaptopurine and 6-thioguanine
Seton	A thread, wire, or gauze of cotton or other absorbent material passed below the skin and left with the ends protruding, to promote drainage of fluid
Methotrexate	Disease-modifying, antimetabolite

For peer review only

For peer review only - http://bmjopen.bmj.com/site/about/guidelines.xhtml

1. Plain English Summary

Crohn's disease is an uncommon long term disease involving painful and damaging inflammation of the gut lining. Damage can cause bloody stools, development of very narrow sections along the gut (strictures), and the formation of abnormal channels (fistulas) between different regions of the gut or between gut and body surface or between gut and nearby organs. Particularly distressing fistulas may occur between intestine and vagina in female patients. During a patient's life the severity of Crohn's disease fluctuates between remission (no symptoms) and relapse (active disease) and treatments aim to induce and maintain remission. Tumour necrosis factor (TNF) has been identified as a molecule important in the development of inflammation in Crohn's disease. Medicines called anti-TNF agents have been developed that counteract the action of TNF and have been found to benefit Crohn's disease patients; they are by far the most expensive medicines used for Crohn's disease and, like all Crohn's disease medicines, for some patients they are associated with unwanted side effects. Unfortunately many patients eventually develop resistance to anti-TNF agents and remission fails. One reason for failure is that some patients develop antibodies to anti-TNFs so that the amount of drug in the patient's blood decreases below levels that are effective. Test kits have been developed and marketed that allow estimation of the levels of anti-TNF and of antibodies to anti-TNF in a patient's blood sample. This information can aid clinicians and patients to decide on the best course of future treatment, and may help avoid continued use of expensive but ineffective medicine. The present project aims to examine evidence about the clinical and cost effectiveness of test kits. The current report will allow NICE to make recommendations about how well the kits work and whether the benefits are worth the cost of the tests for use in the NHS in England and Wales. The assessment will consider both potential for improvement in patients' symptoms associated with use of the tests and the cost of the tests.

2. Decision problem

The current report being undertaken for the NICE Diagnostics Assessment Programme examines the clinical and cost effectiveness of ELISA tests (LISA-TRACKER EISA kits, TNFα-Blocker ELISA kits, and Promonitor ELISA kits) for measuring patient blood levels of anti-TNF agents (Infliximab and Adalimumab; also known as TNF inhibitors) and of antibodies to these agents (i.e., anti-drug antibody levels, ADAbs) in people with Crohn's disease whose disease responds to treatment with TNF inhibitor or who experience secondary loss of response during a maintenance course of TNF inhibitor therapy.

2.1 Anti-tumour necrosis factor alpha (anti-TNFα) agents

TNFα is a small cell-signalling protein (cytokine) involved in inflammatory responses primarily by influencing regulation of various effector cells of the immune system. TNFα has been shown to have

For peer review only

a role in several inflammatory diseases including Crohn's disease, ulcerative colitis, rheumatoid arthritis and ankylosing spondylitis. Therapies have been developed that are directed at blocking the actions of TNFα and thereby reducing inflammation. Such anti-tumour necrosis factor alpha (anti-TNFα) agents bind to cell surface TNFα and free TNFα and block its activity. Blocking of TNFα with anti-TNF drugs has been shown to successfully reduce the inflammation for some patients with inflammatory diseases including Crohn's disease. As these drugs are expensive and can cause potentially serious adverse effects, in England, they are generally used as second or third line treatment in the management of Crohn's disease and are employed when other drugs have not worked or have caused major side effects, and when surgery is not considered the appropriate treatment option. The anti-TNF agents recommended by NICE for the treatment of Crohn's disease are infliximab (Remicade®, Schering-Plough) and adalimumab (Humira®, Abbott Laboratories). These are monoclonal antibodies introduced into the human body to bind and block TNFα. They are classed as monoclonal antibodies because they are derived from genetically engineered immune cells, which are all daughters of a single parent cell, so that in culture they generate and secrete antibodies that are all of identical structure and affinity for TNFα.

2.1.1 Infliximab

Infliximab is a chimeric (mouse-human) monoclonal antibody. It is said to be chimeric because the genetic code determining its amino acid sequences is partly derived from the mouse genome and partly from the human genome. Infliximab belongs to the IgG1 (immunoglobulin gamma type 1) group of antibody molecules (Figure 1). It should be born in mind that IgG1 molecules are globular (not linear as in the diagram) and that they are glycoproteins that have carbohydrate chains attached (not shown in Figure 1). As infliximab is generated from cultured mouse cells, the carbohydrate part of the molecules corresponds to that of mouse rather than human glycoproteins.

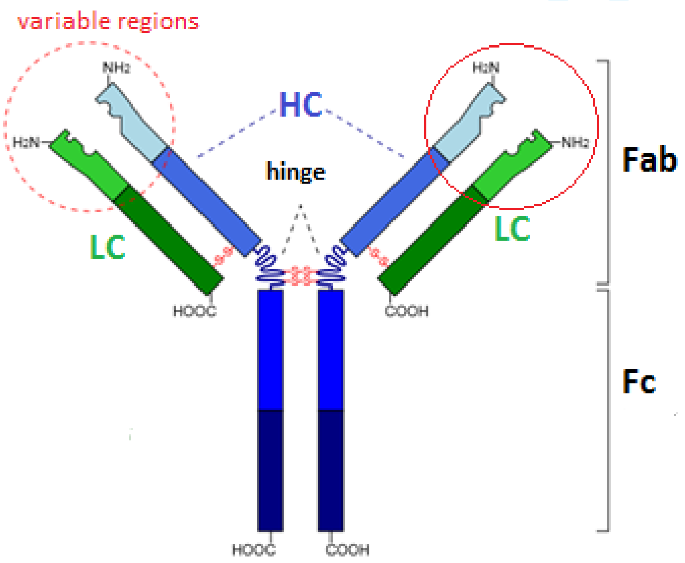

For peer review only - http://bmjopen.bmj.com/site/about/guidelines.xhtml

Figure 1. Diagrammatic representation of the structure of an IgG1 antibody molecule. *The molecule comprises two heavy chains (HC) and two light chains (LC); the HCs are joined together across disulphide bonds (S-S) and each LC is joined to a HC by S-S bonding. The LC and HC have a variable region (different from all other antibodies) at the amino (NH_2) end of the chain; these variable regions are responsible for binding antigen. The rest of the HC and LC are identical to other IgG1 antibodies and are called constant regions. Proteolytic enzymes papain and pepsin cut the molecule just above or below the S-S bonds holding the HC together. When below the HC S-S bond this generates an Fc (Fragment crystallising) and an Fab (Fragment antigen binding) product. When the split is above the HC S-S bond two antigen binding fragments are formed ($F(ab)_2$).*

Infliximab is composed of human IgG1 heavy chain constant regions and human Kappa light chain constant regions (together representing 70% of the genetic makeup of the molecule), plus mouse-derived heavy chain and light chain variable regions (30% of the genetic makeup, 4 out of 12 domains) which carry the binding sites with high affinity and specificity to TNFα (Figure 1). Infliximab was the first anti-TNF agent that was approved and licenced for treating severe active Crohn's disease and active fistulising Crohn's disease in adults and children over the age of six. It is administered intravenously over 1–2 hours. Details of the licenced indication are given in Appendix 1.

Side effects of infliximab include:

- Allergic reaction to the infusion (or infliximab) apparent by:
  - hives (red, raised, itchy patches of skin) or other skin rashes
  - difficulty swallowing or breathing
  - pains in the chest or muscle or joint pain fever or chills
  - swelling of the face or hands
  - headaches or a sore throat
- Serious viral or bacterial infections including tuberculosis, especially in people over 65
- Skin reactions including psoriasis (red scaly patches), rashes, skin lesions, ulcers and hives, and swollen face and lips
- Worsening of heart problems
- Increased risk of cancer or lymphoma
- Liver inflammation

Many of the side effects are reversible if the drug is stopped.

For peer review only - http://bmjopen.bmj.com/site/about/guidelines.xhtml

2.1.2 Adalimumab

Adalimumab is a human IgG1 monoclonal antibody with Kappa light chains. It consists of purely human antibody polypeptide domains (Figure 1). However, as adalimumab is generated from cultured Chinese hamster ovary cells, the carbohydrate part of the molecules corresponds to that of hamster rather than human glycoproteins. Adalimumab is a more recent anti-TNFα therapy that was approved for treating Crohn's disease in adults only. It is administered as a subcutaneous injection by a doctor or nurse or can be self-injected by the patient or a family member. Details of the licenced indication are given in Appendix 1.

Side effects of adalimumab include:

- Reactions to the injection including pain, swelling, redness, bruising and itching
- Allergic reaction to adalimumab including:
  - rashes or hives
  - swollen face, hands and feet
  - trouble breathing
- Greater susceptibility to infections such as colds, flu, pneumonia, sepsis and tuberculosis
- Skin reactions including psoriasis (scaly patches), eczema, other skin rashes and ulcers
- Skin cancer, lymphoma or leukaemia
- Damage to nerves (demyelination)
- Lupus

Many of the side effects are reversible if the drug is stopped.

2.2 Intervention technologies

The intervention technologies are the LISA-TRACKER ELISA kits (Theradiag / Alpha Laboratories), the TNFα-Blocker ELISA kits (Immundiagnostik AG), and the Promonitor ELISA kits (Proteomika). They estimate the following molecules in patient blood sera:

- Infliximab
- Adalimumab
- Anti-infliximab antibodies
- Anti-adalimumab antibodies

For peer review only

2.2.1 Anti-TNF monitoring using assays to measure the levels of anti-tumour necrosis factor-alpha agents (anti-TNFα drugs) and the anti-drug antibodies (ADAb) in the blood plasma or serum

Rationale

In some patients an initial or maintained response to anti-TNF therapy may disappear. This has been observed for all conditions in which these therapies have been used. The reasons for response failure may be various and are not fully understood, however loss of response has often been found to be associated with the generation of immune responses to the anti-TNF agent itself. In particular the patient may generate antibodies directed against the anti-TNF agent, these will bind to the administered anti-TNF agent, nullify its effectiveness and hasten its clearance from the circulation. These effects may explain or partially explain the phenomena of loss of response experienced by some patients. The generation of antibodies against infliximab may not be surprising since about 30% of the molecule has mouse identity. Adalimumab, although termed a fully humanised antibody, has potential to be antigenic since its carbohydrate moieties are mouse derived and because its binding site for anti-TNF is unique and could, according to the network hypothesis of Jerne,[1] lead to generation of antibodies directed against this "idiotypic" region of the drug.

Other patients may respond well to an induction phase of treatment with a TNF inhibitor. However, these patients may lose response in the future, may benefit from optimising dosing or may require review after 12 months of treatment with a TNF inhibitor. Management of responders could benefit from knowing levels of anti-TNF drug and anti-drug antibodies in the patients' blood.

Manufacturers and others have developed various assay procedures for anti-TNF agents and for anti-drug antibodies (ADAbs) in the belief that the levels of circulating anti-TNF and of ADAbs can provide information useful to clinicians in indicating potential reasons for treatment failure, and for dosage or treatment adjustment. The LISA-TRACKER, TNFα-Blocker, and Promonitor are particular examples of these assays and are classified as solid phase Enzyme Linked Immunosorbent Assays (ELISA assays). Other methodologies based on alternative principles of detection and measurement include: [a] radioimmunoassays; liquid phase assays [b] cell reporter assays based on genetically engineered cells incubated in culture medium; [c] mobility shift assays; liquid phase assays using size-exclusion HPLC and fluorescent dye detection. Brief descriptions of the assay methods follow.

ELISAs for infliximab and adalimumab

All three ELISA methods employ similar principles in which, typically, micro-titre plates with 96 wells coated with reagent receive the patient serum samples or various standards and calibrators. Reagents are added with wash steps between additions. The final step involves quantifying the

For peer review only - http://bmjopen.bmj.com/site/about/guidelines.xhtml

amount of a peroxidase label in the titre well, this amount being proportional to the amount of anti-TNF or ADAb in the patient's sample or in the calibrator standard.

The amount of peroxidase present in the well is quantified using a timed incubation with excess substrates (hydrogen peroxide + 3,3',5,5'-tetramethylbenzidine). Peroxidase catalyses the following reaction: Tetramethylbenzidine + hydrogen peroxide → chromogen + water
The incubation is stopped after an appropriate time by the addition of acid and the accumulated chromogen quantified by measuring optical density with a spectrophotometer.

The reagents used for coating the microtitre plate wells and the reagents used in subsequent steps of the assay procedure differ from each other according to manufacturer. The LISA-TRACKER assays for Infliximab and for Adalimumab are illustrated in Figure 2.

For peer review only

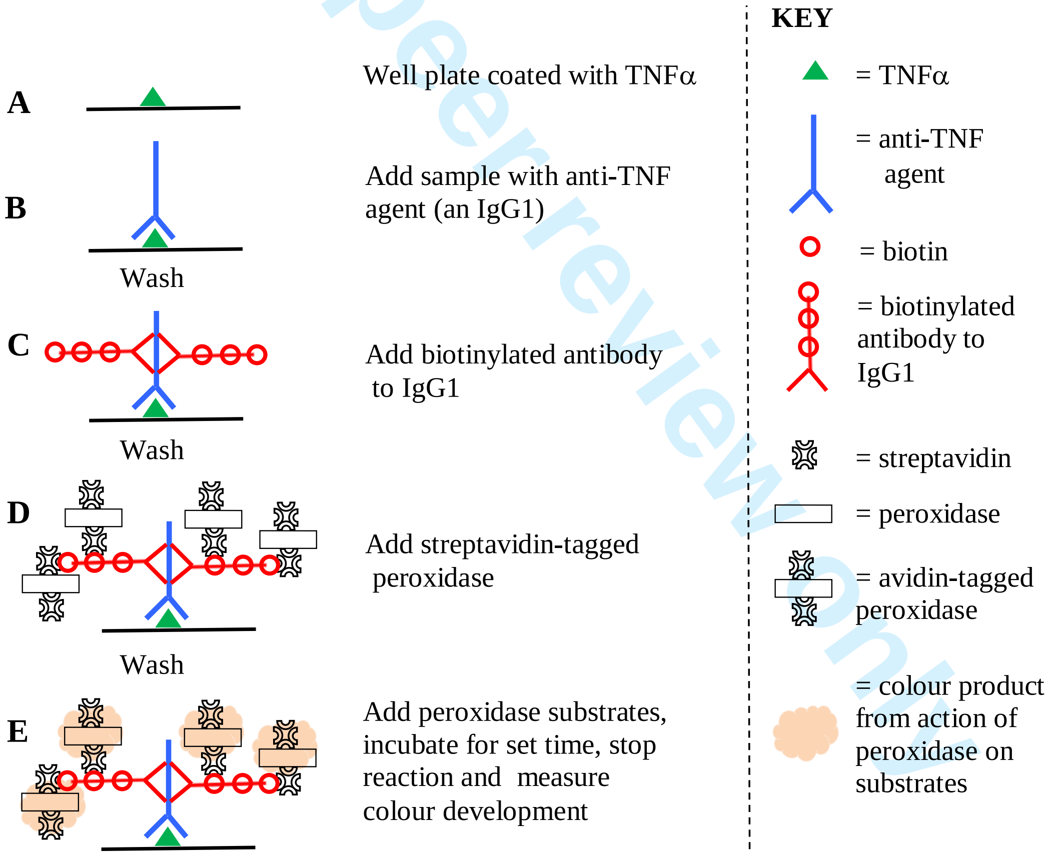

Figure 2. Diagrammatic representation of the LISA-TRACKER assay for infliximab and Adalimumab

Procedural steps C and D are detection steps that function to detect the anti-TNF that is bound to the well surface via TNFα, ensuring a quantitative relationship between anti-TNF and peroxidase. Step E quantifies the amount of peroxidase (and therefore anti-TNF) in the titre well (note: Streptavidin has four very high affinity binding sites for biotin).

Serum samples from patients may contain soluble TNFα receptors; these could compete with anti-TNF for the immobilised TNFα on the well plate and may potentially interfere with the assay. The assay quantifies free anti-TNF. Samples may contain anti-TNF bound to antibodies to anti-TNF, especially in patients who have lost a response to treatment. These anti-TNF-antibody complexes will be washed away at the first wash step leaving only free anti-TNF bound to immobilised TNFα. The amount of anti-TNF lost at the wash step is likely to vary between patients and is unknown; the practical implications of this are uncertain.

TNFα-Blocker and Promonitor differ from LISA-TRACKER in employing a single step and one reagent for detecting well-bound anti-TNF, rather than two steps (C and D in Figure 2) and two reagents. Table 1 summarises the information currently available describing the principle of these assays.

Table 1. Summary of ELISAs to be considered in this review for detection of infliximab and adalimumab

**Manufacturer (Kit)**	**Microplate pre-coat**	**Detection reagent(s)**	
LISA-TRACKER	TNFα	Biotinylated IgG1 antibody	Avidin-tagged peroxidase
TNFα-Blocker ELISA	Monoclonal anti-TNF antibody	Peroxidase labelled antibody	
Proteomika ELISA	Monoclonal anti-TNF antibody	Peroxidase labelled monoclonal anti-TNF antibody	

<u>ELISAs for anti-drug antibodies (ADAbs)</u>

These are available as commercial kits and several “in house” methods are mentioned in the literature. The majority of ELISAs only quantitatively measure “free” anti-TNF and “free” ADAbs and it is acknowledged that the level of the unmeasured “bound” anti-TNF and of “bound” ADAb may vary considerably between patients. The Immundiagnostik assays give semi-quantitative measurement of ‘total’ ADAbs. Thus for some patient samples there is an unknown and unmeasured amount of anti-TNF and of ADAb present, in addition to the measured “free” levels.

Below the LISA-TRACKER methods are reported and differences to TNFα-Blocker and Promonitor are described. The LISA-TRACKER assays for antibodies to infliximab and to adalimumab are illustrated in Figure 3.

For peer review only - http://bmjopen.bmj.com/site/about/guidelines.xhtml

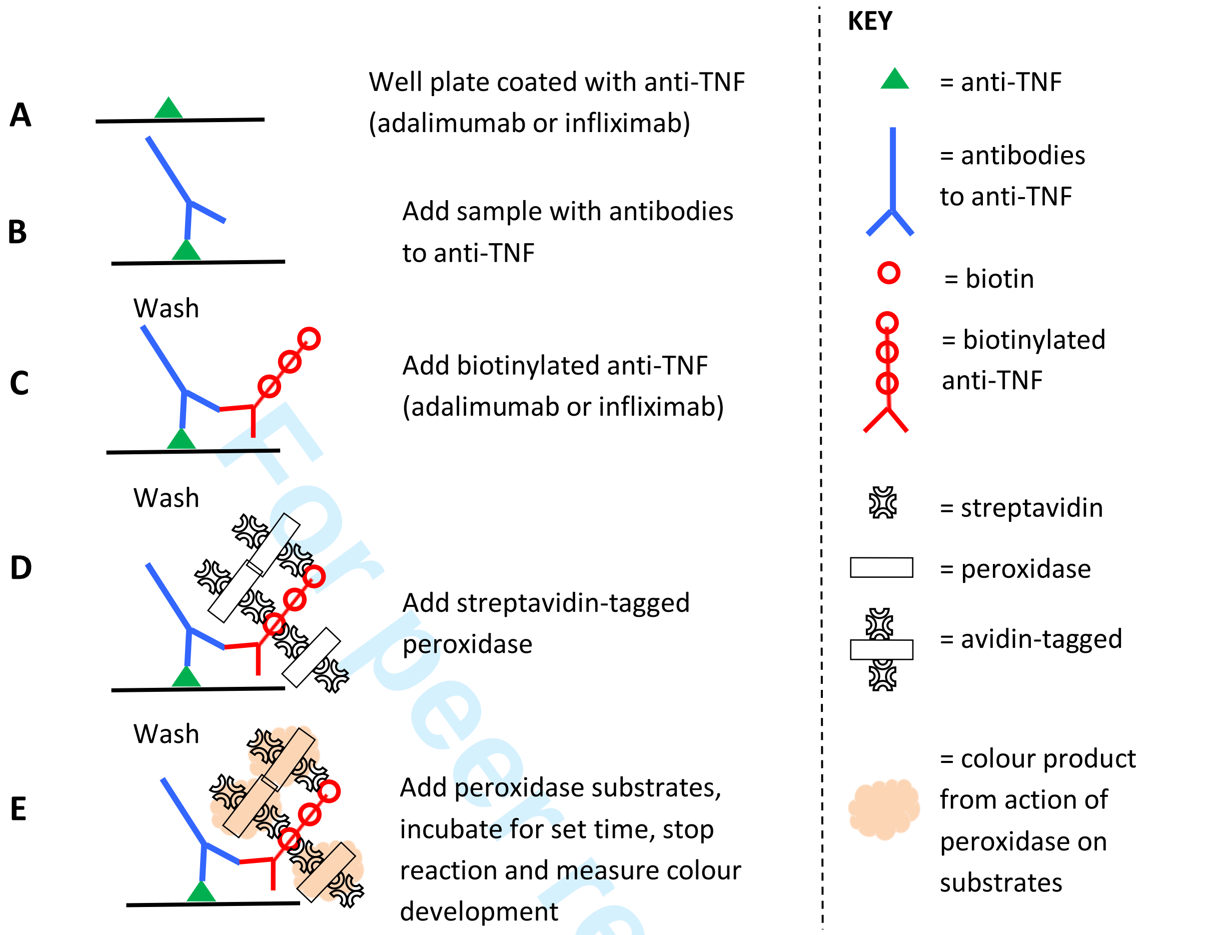

Figure 3. Diagrammatic representation of the LISA-TRACKER assay for antibodies to infliximab or to adalimumab.

Procedural steps C and D are detection steps that function to detect the sample antibodies, ensuring a quantitative relationship between anti-TNF antibodies and peroxidase. Step E quantifies the amount of peroxidase (and therefore anti-TNF antibodies) (note: Streptavidin has four very high affinity binding sites for biotin).

This assay only quantitatively estimates free antibodies to anti-TNF. Thus ADAbs bound to the drug are lost at the first wash. The amount of bound ADAb is likely to vary between patients and is unknown. Whether ADAbs directed at non-idiotypic regions of the drugs (e.g., glycoprotein moieties, variable non-idiotypic mouse regions of infliximab etc.) are detectable or present in samples appears to be uncertain.

TNFα-Blocker and Promonitor differ from LISA-TRACKER in employing a single step and reagent for detecting well-bound anti-TNF rather than two steps (C and D in Figure 2) and two reagents. Table 2 summarises the information currently available describing the principle of these assays.

For peer review only - http://bmjopen.bmj.com/site/about/guidelines.xhtml

Table 2. Summary of ELISAs to be considered in this review for detection of antibodies to infliximab and adalimumab

Manufacturer (Kit)	Microplate pre-coat	Detection reagent(s)	
LISA-TRACKER	Anti-TNF	Biotinylated anti-TNF	Avidin-tagged peroxidase
TNFα-Blocker ELISA infliximab	Infliximab F(ab)2	Peroxidase labelled infliximab	
TNFα-Blocker ELISA adalimumab	Adalimumab F(ab)2	Peroxidase labelled adalimumab	
Proteomika ELISA	Anti-TNF	Peroxidase labelled anti-TNF	

<u>Brief overview of identified non-ELISA assay methods</u>

There are no "gold standard" assays for measuring anti-TNF agents or for antibodies to anti-TNF agents which might provide a robust basis for comparisons between the performance of different assays. According to the US Medical Insurance assessments "candidate" gold standards have been insufficiently investigated to establish any as a gold standard, and according to Steenholdt et al. (2013)[2] it is unknown if and how these different assays compare.[3-7]

There appear to be four types of assay for measuring the levels of anti-TNF drugs and the levels of antibodies against TNF inhibitors in patient blood sera. which differ fundamentally from each other. In addition to ELISAs (solid phase assays) these are:

(a) Radioimmunoassays (RIA) – liquid phase. They appear to measure total anti-TNF and total ADAb (probably as long as the ADAb light chain is lambda class). These RIAs use 125 iodine-labelled human TNFα and 125 iodine-labelled anti-TNFs. In these assays the patient's sample is mixed with a solution containing a fixed amount of 125 iodine-labelled TNFα or 125 iodine-labelled anti-TNF further antibody (e.g., rabbit anti-human immunoglobulin λ-chain) which promotes the formation of immune complexes which are pelleted by centrifugation. Radio-iodine in the pellet is quantified in a gamma-counter. Characteristics of these assays include: i) radio-labelled reagents do not store indefinitely (125 iodine decays with a half-life of 59 days), ii) the laboratory needs to be equipped for handling hazardous (radioactive) material, iii) some staff training may be necessary, and iv) the laboratory requires a gamma counter (preferably automated for high throughput).

(b) Cell Reporter Assays. The reporter cells are genetically engineered to contain genes for two light producing enzymes "*luciferases*" (one from the firefly which can generate red light, and one from the sea pansy which can generate blue light). The firefly gene is under the control of a TNFα signalling

For peer review only - http://bmjopen.bmj.com/site/about/guidelines.xhtml

pathway so that when the cells are incubated in the presence of TNFα they synthesise the enzyme, after a standard incubation time appropriate substrates for the enzyme are added and the emitted red light measured with a luminometer. If anti-TNF is present the TNFα response is partially quenched and the quenching estimated. If ADAb is present, quenching by anti-TNF is reduced and this can be measured. The sea pansy gene is expressed during incubation after which appropriate substrates are added and the blue light emitted measured in the luminometer. The usefulness of the blue light measure is that it allows “normalisation” of the red light emission as interfering agents in patient blood samples equally affect both firefly and sea pansy systems. Requirements in addition to appropriate cell reporter cultures and reagents include requirement for a luminometer (although these are not necessarily routinely available) and equipment for culture of growth arrested genetically engineered cells under controlled conditions (oxygen, CO_2, humidity).

(c) The Mobility Shift Assay is a liquid phase assay based on size exclusion HPLC (SE-HPLC) which separates free probe (small size) from probe in an immune-complex (large size). The ADAb assays use fluorescent-dye-labelled anti-TNF (D*) as the probe. In the presence of antibodies to anti-TNF some D* form immune complexes with these (D*-ADAb complexes) and will exhibit a mobility shift on the SE-HPLC column relative to the D* which remains free. The amount of D* shifted to greater mobility is proportional to the amount of ADAb present. The amount of dye (*) present in the eluent stream coming from the HPLC column at different mobilities is measured with a fluorimeter.

The anti-TNF assay uses fluorescent-dye-labelled TNFα (TNF*) as the probe; in the presence of anti-TNF some TNF* forms immune-complexes with the anti-TNF and these have greater mobility on the SE-HPLC than the free TNF*. The amount of TNF* shifted to greater mobility is proportional to the amount of anti-TNF present. The amount of dye (*) present in the eluent stream coming from the HPLC column at different mobilities is measured with a fluorimeter.

In measuring ADAb the patient sample is subjected to an acid step which “unbinds” bound anti-TNF and ADAb so that all anti-TNF and ADAb are “free”; after neutralisation the sample is incubated with fluorescent-dye-labelled anti-TNF (D*) as described above. Some D* will form immune complexes with the sample ADAbs (D*-ADAb complexes) and these have a different mobility on SE-HPLC than D* thus the mobility of some of the D* is shifted, the proportion of D* shifted is dependent on the level of ADAb in the sample.

2.3 Timing and use of ELISAs

Scoping searches indicate that the anti-TNF and ADAb assays are most frequently administered just before the next administration of the anti-TNF agent. This is said to allow measurement of a “trough” level of anti-TNF and may have been adopted when ELISAs are used so as to minimise effects from

For peer review only - http://bmjopen.bmj.com/site/about/guidelines.xhtml

the presence of anti-TNF-ADAb immune-complexes in samples. For patients whose response to therapy has waned, the results of the tests are frequently dichotomised using a cut off assay result. Thus, on the basis of anti-TNF assays patients are classified as having therapeutic levels of anti-TNF or sub-therapeutic levels, and on the basis of ADAb assay results they are classified as having clinically significant levels of ADAbs or insignificant levels. Such classifications yield four categories of patient for whom different explanations of failed response are possible. Algorithms have been developed prescribing treatment pathways and / or further diagnostic tests (e.g., colonoscopy) based on such classification.

2.4 Target condition / indication

Anti-TNFα is commonly given to people with inflammatory bowel disease (IBD) including Crohn's disease. The general background and treatment pathway for Crohn's disease is summarised below.

2.4.1 Crohn's disease

Crohn's disease is a chronic fluctuating episodic inflammatory condition of the digestive tract; it is uncommon and is currently estimated to affect about 115,000 people in the UK.[8] Together with ulcerative colitis it comprises conditions classed as inflammatory bowel disease (IBD).

<u>Aetiology and pathology</u>

Crohn's disease can affect adults, adolescents or children. Crohn's disease manifests itself mainly during late adolescence or early adulthood. The first onset most commonly occurs between the ages of 16 and 30 with a second peak between the ages of 60 and 80. Women are slightly more frequently affected than men but in children it is seen more often in boys than in girls. The condition has highest prevalence among Jewish people with European descent.

Crohn's disease follows a pattern of acute disease interspersed with periods of remission. Crohn's disease causes inflammation of the lining of the digestive tract which, depending on the individual, occurs at any location from the mouth to the rectum, but most commonly affects the terminal ileum (35%) or the ileocaecal region (40%). Within individuals the disease location is fairly stable.

The main symptoms of Crohn's disease are dependent on disease location and include chronic or nocturnal diarrhoea, abdominal pain, anal lesions, rectal bleeding and weight loss. Clinical signs include pallor, cachexia, abdominal mass or tenderness, or perianal fissures, fistulas or abscesses. Systemic symptoms include malaise, anorexia or fever.[9-11] Extra-intestinal symptoms related to intestinal inflammation include spondyloarthritis (inflammatory rheumatic diseases which cause arthritis, most commonly ankylosing spondylitis), cutaneous manifestations or ocular inflammation.[11] In children, growth failure may be the primary manifestation of Crohn's disease.[12]

For peer review only - http://bmjopen.bmj.com/site/about/guidelines.xhtml

Classification of Crohn's disease disease states and measurement of disease activity

Several classification systems of Crohn's disease have been proposed. The Montreal[13] and Vienna[14] systems are summarised in Tables 3 and 4.

Table 3. Montreal classification of Crohn's disease

Age at diagnosis	Location	Behaviour
A1: <16 years	L1: Ileal	B1: Inflammatory
A2: 17-40 years	L2: Colonic	B2: Stricturing
A3: >40 years	L3: Ileocolonic	B3: Penetrating
	L4: Upper GI disease	P: Perianal disease

Table 4. Vienna classification of Crohn's disease

Age at diagnosis	Location	Behaviour
A1: <40 years of age	L1: Terminal ileum - limited to terminal ileum, with or without spill-over into the caecum	B1: Non-stricturing, non-penetrating
A2: ≥40 years of age	L2: Colon - any colonic location between the caecum and rectum, with no small bowel or upper GI involvement	B2: Stricturing - constant luminal narrowing demonstrated by radiological, endoscopic, or surgical-pathological methods, with pre-stenotic dilation or obstructive signs/symptoms, without the presence of penetrating disease, at any time in the course of the disease
	L3: Ileocolonic - disease of ileum and any location between the ascending colon and rectum	B3: Penetrating - occurrence of intra-abdominal or perianal fistulae, inflammatory masses, and/or abscesses at any time in the course of the disease. Perianal ulcers are included. Postoperative intra-abdominal complications and skin tags are excluded
	L4: Upper GI - any disease proximal to the terminal ileum (excluding mouth), regardless of additional involvement of the terminal ileum or colon	

"The severity of Crohn's disease is difficult to assess, and a global measure encompassing clinical, endoscopic, biochemical and pathological features is not available.[15] The most widely used disease activity measures include the Crohn's Disease Activity Index (CDAI), the Harvey-Bradshaw Index (HBI) or Simple Index (a simplified version of the CDAI), and the Perianal Disease Activity Index

For peer review only

(PDAI). A commonly used health related quality of life measure is the Inflammatory Bowel Disease questionnaire (IBDQ). Other measures include the Crohn's Disease Endoscopic Index of Severity (CDEIS).

The CDAI was developed in the 1970s when a need for a single index to assess disease severity was recognised. Variables measured include number of liquid stools, abdominal pain, general well-being, extra-intestinal complications, use of anti-diarrhoeal drugs, abdominal mass, haematocrit and body weight; scores range from 0 to approximately 600 (see Appendix 2 for a description of the index and the scoring system used). Values of below 150 are suggestive of quiescent disease (remission) and values above 450 are associated with very severe disease.[16] Some investigators have arbitrarily labelled CDAI scores of 150-219 as mildly active disease and scores of 220 to 450 as moderately active disease.[15]

The CDAI has been criticised for having limitations since it fails to encompass aspects of quality of life such as psychological, social, sexual wellbeing and occupational functioning. A patient with a low CDAI score may still be severely limited by these factors.[17] Substantial variability exists when different observers review the same case histories and calculate the CDAI score, although this can be reduced after discussion and education about the terminology. The calculation is based in part on a daily diary kept by the patient for seven days before the evaluation. In practice some investigators and study coordinators assist the patient to complete the diary retrospectively at the time of an evaluation visit; there is no information on the prevalence of this practice. The CDAI score may be low in patients whose primary symptom is drainage of enterocutaneous fistulas, presumably because the presence of an actively draining fistula contributes only 20 points to the score. The CDAI is therefore not an appropriate instrument for assessing the activity of draining abdominal or perianal enterocutaneous fistulas. The CDAI has been criticised for giving too much weight to 'general well-being' and 'intensity of abdominal pain' because these are relatively subjective items. However these aspects of disease are important to patients.[18] A paediatric CDAI has been developed.[18, 19]

The HBI or Simple Index is a modified/simplified version of the adult CDAI. It uses a single day's reading for diary entries and excludes three variables (body weight, haematocrit and use of drugs for diarrhoea). Code values are added together rather than summing the products of code values and coefficients. Scores range from 0 to 20. The CDAI can be predicted reasonably well from the HBI.[20] Other instruments derived from the CDAI are: the Cape Town Index (CTI), which includes parameters on subjective symptoms, physician clinical findings and laboratory data; the three-variable version of the CDAI used for survey research; and the Van Hees Index (VHI), which includes laboratory parameters, sex (male or female) and seven clinical features and excludes subjective patient related items such as well-being and pain.

For peer review only - http://bmjopen.bmj.com/site/about/guidelines.xhtml

The PDAI was developed to account for the morbidity and impairment of quality of life of patients with perianal disease, and to evaluate the effectiveness of perianal disease treatment. Variables include discharge, pain/restriction of activities, restriction of sexual activity, type of perianal disease (including number of fistulas) and degree of induration. Scores range from 0 to 20.[21]

The reliance on traditional disease activity measures (such as the CDAI) to measure treatment effectiveness fails to take into account the impaired quality of life experienced by Crohn's disease patients. The IBDQ is a 32 item health related quality of life measure. The questionnaire evaluates general activities of daily living, intestinal function, social performance, personal interactions and emotional status. Four-dimensional scores cluster items under bowel function, emotional function, systemic function and social function. Scores range from 32 to 224.[22]

The CDEIS was developed to take into account endoscopic data, such as lesion severity, when assessing severity of the disease. Variables include the presence or absence of deep or superficial ulceration in various segments of the intestinal tract, the surface involved (in cm), surface ulcerated (in cm) and presence of ulcerated stenosis. Scores range from 0 to 30.[23]

Clinical studies have variously defined a clinical response as a decrease in CDAI score of 50, 60, 70 or 100 points. In 2000 the FDA and EMEA suggested that a meaningful decrease in the CDAI score is a decrease of 100 points.[18]"{#19}

Working definitions of disease severity have been developed by the Practice Parameters Committee of the American College of Gastroenterology (2001).[11] These are:-

Mild-moderate disease:

- *"Mild-moderate disease applies to ambulatory patients able to tolerate oral alimentation without manifestations of dehydration, toxicity (high fevers, rigors, prostration), abdominal tenderness, painful mass, obstruction, or >10% weight loss"*

Moderate-severe disease:

- *"Moderate-severe disease applies to patients who have failed to respond to treatment for mild-moderate disease or those with more prominent symptoms of fever, significant weight loss, abdominal pain or tenderness, intermittent nausea or vomiting (without obstructive findings), or significant anaemia."*

Severe-fulminant disease:

For peer review only - http://bmjopen.bmj.com/site/about/guidelines.xhtml

- *“Severe-fulminant disease refers to patients with persisting symptoms despite the introduction of steroids as outpatients, or individuals presenting with high fever, persistent vomiting, evidence of intestinal obstruction, rebound tenderness, cachexia, or evidence of an abscess.”*

Remission:

- *“Remission” refers to patients who are asymptomatic or without inflammatory sequelae and includes patients who have responded to acute medical intervention or have undergone surgical resection without gross evidence of residual disease. Patients requiring steroids to maintain well-being are considered to be ‘steroid-dependent’ and are usually not considered to be ‘in remission’.”*

Anti-TNF monitoring in Crohn’s disease

Crohn’s disease is associated with elevated levels of the immune-regulatory protein TNFα. The reasons for this elevation in Crohn’s disease is still largely unknown. Anti-TNF therapies have been shown to block the action of TNFα and to improve outcomes for some patients. Patients receive anti-TNF therapy after failed attempts to improve the condition with first line glucocorticosteroids, 5-aminosalicylates, antibiotics and second line treatment (e.g., methothrexate). These patients have severe symptoms and they are at the end of the patient pathway with the only alternative option being surgery.

Like other treatment regimens anti-TNF treatment aims to induce remission (induction therapy) and prevent relapse (maintenance therapy). However failure to induce a response and relapse or loss of response are common. Approximately 10% of patients per year loose response to anti-TNF drugs.[24] The annual risk of response loss per patient has been estimated at about 13%.[25] During “episodic” infliximab therapy about 37-61% lose response.[26] Mechanisms of loss of response to anti-TNF agents and of failure to respond are still mainly unclear, however the fact that some patients generate immune responses to therapy offers one plausible contributory explanation. However other pharmacodynamics mechanisms may reduce the drug below therapeutic levels, furthermore there may be alternative secondary pathways of inflammation independent of TNFα that operate in some patients rendering anti-TNF of little use.

During scheduled infliximab therapy the incidence of antibodies is 6-16%.[27, 28] Anti-TNF antibody formation in patients treated with Infliximab has been shown to be as high as 37-61%.[29] Concomitant immunosuppressive therapy may decrease the formation of ADAbs.[26, 27, 29] Candidate risk factors for ADAb production include hereditary predisposition, a dysfunctional immune system, experience of infection(s) that trigger an abnormal response, smoking, environmental factors such as sanitation.

For peer review only - http://bmjopen.bmj.com/site/about/guidelines.xhtml

The ELISA assays could be used in good responders (i.e., those responding to initial induction course of anti-TNF treatment) as well as in patients with secondary loss of response (i.e., those initially responding to anti-TNF treatment but loosing this response over time). The use of these technologies provides a clinician with potentially useful information that may guide individual patient's future treatment. Such information may aid in anticipating the loss of response in responders, while for non-responders such analyses may help in estimating the likelihood of various candidate reasons for primary non-response or secondary loss of response. For example in non-responders with low levels of drug and high levels of ADAbs the loss or lack of response may be surmised to be due to rapid clearance of the drug due to action of ADAbs; on the other hand a low level of anti-TNF in the absence of ADAbs may be suggestive of non-immune mechanisms of rapid drug clearance, while high levels of drug in absence of antibodies in non-responders may be suggestive of a TNFα-independent pathology for the condition in a particular patient. Algorithms for future treatment based on anti-TNF and ADAb estimates have been published.

In theory the application of the tests in conjunction with an appropriate algorithm for treatment based on test results:

- May improve quality of life and other outcomes (e.g., faster healing of flare-ups, reduced abdominal pain and associated diarrhoea)
- May optimise the treatment plan (facilitate adoption of the most suitable future treatment for individual patients; this might involve a switch to an alternative anti-TNF or a biologic with an alternative mechanism of action)
- May minimise the risk of drug overdose and associated adverse events
- May allow earlier de-escalation of therapy, leading to a reduction in the overall drug used
- May help to reduce the amount of drugs used inappropriately, unnecessary hospital visits, risk of surgery, and associated costs

Crohn's disease: Management and Care pathway

The treatment of Crohn's disease is complex, which in general aims at: a) reducing symptoms through induction and maintenance of remission, b) minimising drug-related toxicity, and 3) reducing the risk of surgery. The management options for Crohn's disease include drug therapy (e.g., glucocorticosteroids, 5-aminosalicylate, antibiotics, immunosuppressives, TNFα inhibitors), enteral nutrition, smoking cessation and, in severe or chronic active disease, surgery (Table 5). The choice of treatment amongst the available drugs is influenced by patient age, site and activity of disease, previous drug tolerance and response to treatment, and the presence of extra-intestinal manifestations.[30, 31] Enteral nutrition is widely used as a first line treatment to facilitate growth and development in children and young people. Adjuvant therapy commonly coexists and includes

For peer review only - http://bmjopen.bmj.com/site/about/guidelines.xhtml

management of extra-intestinal manifestations, antibiotics, corticosteroids or immunomodulator therapy. Between 50% and 80% of people with Crohn's disease require surgery due to complications such as strictures causing symptoms of obstruction, fistula formation, perforation or failure of medical therapy.[32]

Once remission has been achieved, maintenance therapy can be considered following assessment of the course and extent of Crohn's disease, effectiveness and tolerance of previous treatments, presence of biological or endoscopic signs of inflammation, and potential for complications.

Table 5. Treatment options for patients with Crohn's disease[33]

Patient group	Treatment Line and Treatment
**Ileocaecal disease not fistulating with <100 cm of bowel affected: initial presentation or relapse**	
• mildly active	1st observation with monitoring or budesonide or 5-ASA therapy
• moderately active: initial presentation or non-corticosteroid-dependent/-refractory relapse	1st budesonide and/or 5-ASA therapy, or conventional oral corticosteroids (use previously effective treatment for relapse)
	2nd immunomodulator therapy + oral corticosteroid taper
	3rd anti-TNF therapy + oral corticosteroid taper
• moderately active: relapse corticosteroid-dependent/-refractory	1st consideration of early initiation of anti-TNF therapies + oral corticosteroid taper
	2nd surgery
• severely active: initial presentation or non-corticosteroid-dependent/-refractory relapse	1st hospitalisation + oral or intravenous conventional corticosteroids + consideration of surgery
	2nd anti-TNF therapy or surgery
• severely active: relapse corticosteroid-dependent/-refractory	1st hospitalisation + consideration of early initiation of anti-TNF therapy or surgery
**Colonic disease not fistulating: initial presentation or relapse**	
• mildly active	1st 5-ASA therapy or alternatively oral corticosteroids
	2nd surgery
• moderately or severely active:	1st oral or intravenous corticosteroids + immunomodulator therapy + consideration for surgery

For peer review only - http://bmjopen.bmj.com/site/about/guidelines.xhtml

initial presentation or non-corticosteroid-dependent/-refractory relapse	2nd anti-TNF therapy + consideration for surgery
	3rd surgery
• moderately or severely active: relapse corticosteroid-dependent/-refractory	1st early initiation of anti-TNF therapy or consideration for surgery
	2nd surgery
**Extensive small bowel disease (>100 cm of bowel affected) not fistulating: initial presentation or relapse**	1st oral corticosteroids + early introduction of immunomodulators
**Upper GI disease (oesophageal and/or gastroduodenal disease) not fistulating: initial presentation or relapse**	1st proton pump inhibitor
**Perianal or fistulating disease: initial presentation or relapse**	
• simple perianal fistula: symptomatic	1st loose seton + drainage of perianal abscess if present
• complex perianal fistulae	1st loose seton placement + drainage of perianal abscess if present
• non-perianal fistulae	1st multidisciplinary input + supportive care

Abbreviations: 5-ASA 5-Aminosalicylic Acid, TNF tumour necrosis factor, GI gastrointestinal

<u>Induction of remission</u>

Usually, at first presentation, people with active Crohn’s disease are recommended monotherapy with a conventional glucocorticosteroid (prednisolone, methylprednisolone or intravenous hydrocortisone), which is aimed at inducing remission as a first line treatment. Alternatively, treatment with budesonide, 5-ASA, or enteral nutrition may be offered to a group of people who do not choose to take or who are intolerant to glucocorticosteroid therapy.

The addition of an immunosuppressant (azathioprine, mercaptopurine or methotrexate) to a conventional glucocorticosteroid or budesonide as an add-on therapy for inducing remission is recommended for people who have active Crohn’s disease and have experienced two or more inflammatory exacerbations in a 12-month period, or in whom the glucocorticosteroid dose cannot be tapered. As advised in the current online version of the British national formulary (BNF)[34] or British National Formulary for Children (BNFC),[34] the effects of azathioprine, mercaptopurine, and methotrexate as well as levels of neutropenia (in people on azathioprine or mercaptopurine) should be monitored.

For peer review only

Adults with severe active Crohn's disease who fail to respond to the first line of treatment with conventional therapy (e.g., immunosuppressive drugs, corticosteroids), or who are intolerant of or have contraindications to the above-mentioned conventional therapy, anti-TNF alpha agents (infliximab and adalimumab) are recommended as treatment options within their licensed indications. The administration of anti TNF alpha agents is recommended until 12 months after the start of treatment or until treatment failure (including the need for surgery), depending on whichever occurs first. Periodic reassessment and monitoring of disease activity (at least every 12 months) is advised in order to ascertain the clinical appropriateness of ongoing treatment. Usually, treatment course needs to be initiated with the less expensive drug by considering drug administration costs, dose, and product price per dose. The use of anti-TNF-alpha drugs for the treatment of Crohn's disease is covered in the 2010 NICE technology appraisal guidance 187 (Infliximab (review) and adalimumab for the treatment of Crohn's disease).[35]

Surgery should be considered as an alternative to medical treatment early in the course of the disease for people (adults, children, and young people) whose disease is limited to the distal ileum or have growth impairment despite optimal medical treatment and/or refractory disease (children and young people).

Maintenance of remission

People with Crohn's disease in remission can be managed with or without maintenance treatment. The options for maintenance therapy (including treatment or no treatment) need to be discussed with patients, their parents, and/or carers. The discussion should include risk of inflammatory exacerbations (with and without drug treatment) and the potential side effects of drug treatment. People who decline to receive maintenance treatment should agree with follow-up plans (e.g., frequency and duration of visits) and receive information on symptoms related to relapse (e.g., unintended weight loss, abdominal pain, diarrhoea, general ill-health) to ensure timely consultations with their healthcare professional.

People with Crohn's disease in remission who choose to receive maintenance therapy may be offered azathioprine or mercaptopurine monotherapy if their remission was induced using a conventional glucocorticosteroid or budesonide. Methotrexate can be offered to people whose remission was induced by methotrexate or people who did not tolerate azathioprine or mercaptopurine for maintenance therapy or those who have contraindications to azathioprine or mercaptopurine. Treatment with 5-ASA can be recommended to maintain remission after surgery.

For peer review only - http://bmjopen.bmj.com/site/about/guidelines.xhtml

If remission has been achieved with anti-TNF medication, then maintenance with anti-TNF with or without combination with another immunomodulator can be recommended. Continuation of treatment with infliximab or adalimumab during remission is advised only if there is evidence of ongoing active disease given clinical symptoms, biological markers, including endoscopy if necessary. The balance between harms and benefits of ongoing treatment should be taken into account. People who relapse after treatment is stopped have the option to start this treatment again.

3 Decision questions and objectives

3.1 Decision questions

The decision questions for this project are shown in the box below:

1. *Does concurrent testing of TNF inhibitor levels and antibodies to TNF inhibitors represent a clinically and cost-effective use of NHS resources in people with Crohn's disease whose disease responds to treatment with TNF inhibitor?*

Testing will be carried out:

a) 3 to 4 months after start of treatment or

b) 3 to 4 months and every 12 months from start of treatment

2. *Does concurrent testing of TNF inhibitor levels and antibodies to TNF inhibitors represent a clinically and cost-effective use of NHS resources in people with Crohn's disease who experience secondary loss of response during maintenance treatment with TNF inhibitor?*

3. *Does testing of TNF inhibitor levels followed by reflex testing of antibodies to TNF inhibitors if drug level is undetectable represent a clinically and cost-effective use of NHS resources in people with Crohn's disease whose disease responds to treatment with TNF inhibitor?*

Testing will be carried out:

a) 3 to 4 months after start of treatment or

b) 3 to 4 months and every 12 months from start of treatment

4. *Does testing of TNF inhibitor levels followed by reflex testing of antibodies to TNF inhibitors if drug level is undetectable represent a clinically and cost-effective use of NHS resources in people with Crohn's disease who experience secondary loss of response during maintenance treatment with TNF inhibitor?*

3.2 Objectives

Given these decision questions the four main objectives for this report are:

For peer review only - http://bmjopen.bmj.com/site/about/guidelines.xhtml

A) To provide a technical description, and (where evidence allows) an evaluation, of the listed intervention tests used for Crohn's disease in therapeutic monitoring of TNF inhibitors (infliximab and adalimumab) and their respective antibodies. This will include what the assays measure and the mechanisms of the assays.

In addition, published studies which include a comparison (including relative test performance) of two or more intervention tests, or which compare an intervention test with a test method which can be used to perform a linked evidence assessment will be reviewed and critiqued. Data submitted by the manufacturers will be used to supplement published studies if deemed of sufficient detail and quality.

B) To describe algorithms used in studies which include data on one or more intervention test or on a test which allows a linked evidence approach to be performed (i.e., algorithms used in studies identified in Objective C). The studies are required to provide an algorithm and report clinical outcomes for the management of patients with Crohn's disease following measurement of serum levels of anti-TNF drug and anti-drug antibodies. To compare the algorithms used following therapeutic drug monitoring to the algorithms specified in the TAXIT study for responders,[36] and in the reporting of secondary loss of response (algorithm adapted from the study by Scott and Lichtenstein, 2014[37]).

C) To systematically review the literature comparing the clinical effectiveness of [a] the intervention assays for anti-TNF agents and/ or for ADAbs used in conjunction with a treatment algorithm in Crohn's patients treated with infliximab or adalimumab; with [b] standard care (no tests performed or test-informed algorithm used) in Crohn's disease patients treated with infliximab or adalimumab. Where evidence exists on the comparison of standard care with other test assays used in conjunction with an algorithm, this will be assessed and critiqued and test performance will be compared with that of the study interventions (LISA-TRACKER ELISA kits, TNFα-Blocker ELISA kits, and Promonitor ELISA kits) (see Objective A).

D) To assess the cost-effectiveness of employing anti-TNF monitoring with LISA-TRACKER ELISA kits, TNFα-Blocker ELISA kits, and Promonitor ELISA kits in patients with Crohn's disease compared with standard care (no anti-TNF monitoring). Where direct evidence is unavailable for this comparison, or where such a comparison is not well supported with evidence, a linked approach to evidence will be considered (see Objective C above) in which evidence of clinical effectiveness is taken from studies using alternative test methodology and an assessment is made of the relative performance this methodology relative to the intervention assays.

For peer review only - http://bmjopen.bmj.com/site/about/guidelines.xhtml

4. Methods for assessing clinical effectiveness

Systematic review methods will follow the principles outlined in the Centre for Reviews and Dissemination (CRD) guidance for undertaking reviews in health care[38] and the NICE Diagnostic Assessment Programme manual.[39]

4.1 Identification and selection of studies

4.1.1 Search strategies for clinical effectiveness

Scoping searches have been undertaken to inform the development of the search strategies. Additional phrases were added to the scoping searches to broaden the search to find other relevant articles that had no terms for the test name or type of test (e.g., Baert et al., 2003[26]) or population (e.g., Vande Casteele et al., 2012[40]) in title, abstract or indexing. Additional searches will be carried out where necessary. Searches for studies for cost and quality of life will be developed separately. An iterative procedure was used, with reference to scoping searches undertaken by information specialists at NICE. A copy of the main draft search strategy that is likely to be used in the major databases is provided in Appendix 3. This strategy may be further refined and other appropriate concepts may be added. This search strategy developed for Medline will be adapted as appropriate for other databases. All retrieved papers will be screened for potential inclusion.

The search strategy will comprise the following main elements:

- Searching of electronic bibliographic databases
- Contact with experts in the field
- Scrutiny of references of included studies
- Screening of manufacturer's and other relevant organisations' websites for relevant publications

Bibliographic databases will include:

MEDLINE; MEDLINE In-Process & Other Non-Indexed Citations; EMBASE; Cochrane Library (including Cochrane Systematic Reviews, DARE, CENTRAL, NHS EED, and HTA databases); Science Citation Index and Conference Proceedings (Web of Science); Index to Theses; DART-Europe; Dissertations & Theses; NIHR Health Technology Assessment Programme; PROSPERO (International Prospective Register of Systematic Reviews).

The following trial and patent databases will also be searched: Current Controlled Trials; ClinicalTrials.gov; UKCRN Portfolio Database; WHO International Clinical Trials Registry Platform; Espacenet (European Patent Office); Patentdocs (US Patents database).

For peer review only - http://bmjopen.bmj.com/site/about/guidelines.xhtml

Specific conference proceedings, to be selected with input from clinical experts and Specialist Committee Members, will be checked for the last five years.

The online resources of various health services research agencies, regulatory bodies, professional societies and manufacturers will be consulted via the Internet. These are likely to include:

- International Network of Agencies for Health Technology Assessment (INAHTA) Publication http://www.inahta.org/
- FDA medical devices: http://www.fda.gov/MedicalDevices/DeviceRegulationandGuidance/Databases/default.htm
- European Commission medical devices http://ec.europa.eu/health/medical-devices/
- Theradiag http://www.theradiag.com/en/
- Immundiagnostik http://www.immundiagnostik.com/en
- Proteomika http://www.proteomika.com/
- American college of gastroenterology http://gi.org/

This will be supplemented by web searching on specific test names using Google and a meta-search engine.

The reference lists of included studies and relevant review articles will be checked. Citation searches of selected included studies will be undertaken using Scopus. Identified references will be downloaded in Endnote X7 software. Included papers will be checked for errata using PubMed.

4.1.2 Inclusion and exclusion of relevant studies

Inclusion of relevant studies to address Objective A

Detailed information will be sought from manufacturers regarding mechanisms and reactants (in particular specificities and properties of antibodies and other reagents) employed in ELISA tests and radioimmunoassay, mobility shift assays and cell reporter tests (if used for a linked evidence approach).

In addition published studies which describe the intervention tests and tests used for a linked evidence approach will be identified. Those providing useful information about test mechanisms that is different or additional to that supplied by manufacturers of tests will be included. Assessment of inclusion will be based on the judgement of two reviewers.

For peer review only - http://bmjopen.bmj.com/site/about/guidelines.xhtml

Studies which compare test performance of two or more tests will be included either if they compare two or more intervention tests, or compare an intervention test with a test method which can be used to perform a linked evidence assessment.

All study designs will be considered for inclusion.

Inclusion criteria for studies to address Objective B

Studies that report an algorithm with the use of one of the intervention tests for the management of patients with Crohn's disease following measurement of serum levels of anti-TNF drug and anti-drug antibodies (infliximab or adalimumab). All study designs will be considered for inclusion.

Inclusion criteria for studies to address Objective C

Studies that satisfy the following criteria will be included:

Population	Crohn's disease patients (adults and children) receiving infliximab or adalimumab. If the evidence on Crohn's disease patients is limited, mixed patient groups containing Crohn's disease and ulcerative colitis patients will be included even if results are not reported separately. The limitations following from this will be discussed.
Intervention	Use of LISA-TRACKER ELISA kits, TNFα-Blocker ELISA kits, and Promonitor ELISA kits to estimate plasma or sera levels of anti-TNF agents and / or of ADAbs in which test results are employed in conjunction with a treatment algorithm (Table 6). Other assay methods will be considered should a linked evidence approach be adopted (Table 6).
Comparator	Standard care (Treatment decisions made on clinical judgement without measuring levels of TNF inhibitor and antibodies to TNF inhibitors).
Outcome	Any patient outcome (e.g., CDAI score based response rate, any measure of change in severity of Crohn's disease including physicians global assessment; Duration of response, relapse and remission; Rates of hospitalisation; Rates of surgical intervention; Time to surgical intervention; Adverse effects of treatment; Health related quality of life; and secondary if two strategies compared are found clinically equivalent: Time to result; Number of inconclusive results; Frequency of dose adjustment; Frequency of treatment switch).

For peer review only - http://bmjopen.bmj.com/site/about/guidelines.xhtml

Study design All study designs will be considered for inclusion.

Healthcare setting Secondary and tertiary care.

Meeting abstracts will be included if they provide sufficient data on type of ELISA assay, patient group, algorithm, measurements from assays and clinical outcomes.

Table 6. Assay methods included as interventions in the review

LISA-TRACKER assay kits (Theradig/Alpha Laboratories)

- LISA-TRACKER Adalimumab (LTA002)
- LISA-TRACKER Infliximab (LTI002)
- LISA-TRACKER anti-Adalimumab (LTA003)
- LISA-TRACKER anti-Infliximab (LTI003)
- LISA-TRACKER Duo Adalimumab (LTA005)
- LISA-TRACKER Duo Infliximab (LTI005)

Immundiagnostik TNFα-Blocker ELISA kits (Immundiagnostik/BioHit Healthcare):

- Immundiagnostik TNFα-Blocker ADA, antibodies against infliximab (e.g. Remicade®) ELISA (K9650)
- Immundiagnostik TNFα-Blocker ADA, antibodies against adalimumab (e.g. Humira®) ELISA (K9652)
- Immundiagnostik TNFα-Blocker ADA, TOTAL antibodies against infliximab (e.g. Remicade®) ELISA (K9654)
- Immundiagnostik TNFα-Blocker ADA, TOTAL antibodies against adalimumab (e.g. Humira®) ELISA (K9651)
- Immundiagnostik TNFα-Blocker monitoring, infliximab drug level (e.g. Remicade®) ELISA (K9655)
- Immundiagnostik TNFα-Blocker monitoring, adalimumab drug level (e.g. Humira®) ELISA (K9657)

Promonitor ELISA kits (Proteomika):

- Promonitor-ADL ELISA (5080230000)
- Promonitor-IFX ELISA (5060230000)
- Promonitor-ANTI-ADL ELISA (5090230000)
- Promonitor-ANTI-IFX ELISA (5070230000)

For Objective C test methods that are not included as an intervention but have evidence comparing it

For peer review only - http://bmjopen.bmj.com/site/about/guidelines.xhtml

to an intervention test and evidence reporting clinical outcomes, should be included for the purpose of performing linked evidence modelling only (including: radioimmunoassays, cell reporter assays, liquid-phase mobility shift assays and in-house ELISAs).

4.2 Review strategy

The general principles recommended in the PRISMA statement will be considered.[41] Records rejected at full text stage and reasons for exclusion will be documented. Two reviewers will independently screen the titles and abstracts of all records identified by the searches and discrepancies will be resolved through discussion. Disagreement will be resolved by retrieval of the full publication and consensus agreement. Full copies of all studies deemed potentially relevant, will be obtained and two reviewers will independently assess these for inclusion; any disagreements will be resolved by consensus or discussion with a third reviewer.

4.3 Data extraction strategy

Data will be extracted by one reviewer, using a piloted, data extraction form. A second reviewer will check the extracted data and any disagreements will be resolved by consensus or discussion with a third reviewer. Examples of data extraction sheets for patient-based and diagnostic accuracy studies are provided in Appendix 4.

4.4 Quality assessment strategy

Where appropriate, the quality of diagnostic accuracy studies will be assessed using QUADAS-2 (see Appendix 5).[42] As a broad range of study designs have been identified in the scoping searches, the use of a single checklist, in contrast to individual checklists for each study design, is considered appropriate. The Downs and Black checklist[43] will therefore be used to assess the quality of non-randomised studies meeting the inclusion criteria (see Appendix 5). This 27-item checklist provides both an overall score for study quality and a profile of scores not only for the quality of reporting, internal validity (bias and confounding) and power, but also for external validity. RCTs will be quality appraised using the Cochrane risk of bias tool (see Appendix 5).[44] The results of the quality assessment will provide an overall description of the quality of the included studies and will provide a transparent method of recommendation for design of any future studies. Quality assessment will be undertaken by one reviewer and checked by a second reviewer, any disagreements will be resolved by a third reviewer through discussion.

4.5 Methods of analysis/synthesis

Objective A

Narrative descriptions of tests in tables and texts will be undertaken.

For peer review only - http://bmjopen.bmj.com/site/about/guidelines.xhtml

Objective B

Algorithms will be narratively described and compared to the algorithm used in the TAXIT study (for good responders),[36] and the algorithm adapted from Scott and Lichtenstein (2014) (for secondary loss of response).[37] Non-compliant patients may be considered additionally in the algorithms. Time of testing, sequence of testing (drug and antibodies), sequence of analysis as well as thresholds used in the algorithms will be considered to address the research questions.

Objective C

Depending on the available evidence, analyses will be stratified according to the type of ELISA assay, type of drug (infliximab or adalimumab) and patient group (patients with secondary loss of response and patients with good response to anti-TNF treatment).

Study, treatment, population, and outcome characteristics will be summarised and compared qualitatively and, where possible, quantitatively in text, graphically and in evidence tables. Pooling studies results by meta-analysis will be considered. Where meta-analysis is considered unsuitable for some or all of the data identified (e.g., due to the heterogeneity and/or small numbers of studies), we will employ a narrative synthesis. Typically, this will involve the use of text, graphs and tables (as appropriate) to summarise data. These will allow the reader to consider any outcomes in the light of differences in study designs and potential sources of bias for each of the studies being reviewed. Studies will be organised by objective addressed. A detailed commentary on the major methodological problems or biases that affected the studies will also be included, together with a description of how this may have affected the individual study results.

For Objective C we aim to identify studies that compare treatment decisions made on clinical judgement without measuring levels of TNF inhibitor and antibodies to TNF inhibitors with treatment decisions based on measurement of TNF inhibitor and antibodies to TNF inhibitors. We will consider using a linked-evidence approach[45] in which studies report patient management informed by measurement of anti-TNF and antibodies by other methods (e.g., radioimmunoassay, liquid-phase mobility shift assay, in-house ELISAs); this will require an assessment of evidence relating to the comparable performance of ELISA assays with radioimmunoassay, liquid-phase mobility shift assays and in-house ELISAs.

In studies where an ELISA has been used but there is no comparator arm, or the comparator arm is a convenience sample (retrospective/historical population), outcomes will be listed and appraised. Time of testing, sequence of testing (drug and antibodies) and sequence of analysis will be considered to address the research questions.

For peer review only - http://bmjopen.bmj.com/site/about/guidelines.xhtml

5. Methods for synthesising cost-effectiveness evidence

5.1 Identifying and reviewing published cost-effectiveness studies

Published cost-effectiveness studies will be reviewed. All papers which present findings on the costs and outcomes of LISA-TRACKER ELISA kits, TNFα-Blocker ELISA kits, and Promonitor ELISA kits for measuring levels of TNF inhibitors and of anti-drug antibodies will be reviewed in detail. Information on assay procedures additional to ELISA methods will be sought for the purposes of providing data for a linked approach to evidence synthesis should this be required.

5.1.1 Search strategy and data extraction

A comprehensive search of the literature for published economic evaluations (including any existing models), cost studies and quality of life (utility) studies will be performed. The search strategy used will be based on the strategy developed for the clinical effectiveness review (see Appendix 3).

Databases will include:

- MEDLINE (Ovid)
- MEDLINE In-Process Citations and Daily Update (Ovid)
- EMBASE (Ovid)
- NHS Economic Evaluation Database (NHS EED) (Cochrane Library)
- Science Citation Index (Web of Knowledge)
- Cost-effectiveness analysis (CEA) registry
- Research Papers in Economics (REPAC)

Additional searches will be performed where necessary to identify other relevant information to support the development of an economic model for this project, these may be directed towards - costs, utilities and transition probabilities as required.

Data will be extracted by one reviewer and checked by a second, using a standardised data extraction form for the economic studies; this will be developed to summarise the main characteristics of the studies and to capture useful data that can inform the economic model. Any discrepancies will be resolved by discussion. If this is not feasible, a third reviewer will be consulted.

The quality of any full economic evaluation studies will be assessed using the CHEERS checklist (see Appendix 5).[46] Any studies containing an economic model will be further assessed using the framework for the quality assessment of decision analytic modelling (see Appendix 5).[47]

For peer review only - http://bmjopen.bmj.com/site/about/guidelines.xhtml

5.2 Evaluation of costs, quality of life and cost-effectiveness

5.2.1 Model structure, time horizon and transition probabilities

In developing the economic model we will consult the previous Health Technology Assessment report (HTA) conducted by Dretzke and colleagues (2011).[48] The main aim of this HTA report was to assess the cost-effectiveness of anti-TNFs in the management of moderate-to-severe Crohn's disease in the UK National Health Service (NHS). The authors developed a Markov model from an NHS and Personal Social Services (PSS) perspective to estimate the incremental cost per quality-adjusted life year (QALY) gained for both adalimumab and infliximab compared with standard care. The assumptions used in the model for the appraisal of Infliximab (review) and adalimumab for the treatment of Crohn's disease (technology appraisal 187)[48] may be used to inform the development of a de novo model. We will create a Markov-type model to assess the cost-effectiveness of LISA-TRACKER ELISA kits, TNFα-Blocker ELISA kits, and Promonitor ELISA kits compared with standard care. The perspective of the model will be that of the NHS and PSS. To assess the cost-effectiveness, the intervention tests (LISA-TRACKER ELISA kits, TNFα-Blocker ELISA kits, and Promonitor ELISA kits) will be compared with standard care in the following populations:

- In patients with secondary loss of response to anti-TNF treatment
- In patients who respond well to anti-TNF treatment

The following comparisons will be made where possible:

- Concurrent versus reflex testing
- Testing conducted every 3 to 4 months versus testing conducted at 3 to 4 months then yearly (in patients who respond well to anti-TNF treatment)

If data permits, we will compare the different LISA-TRACKER ELISA kits, TNFα-Blocker ELISA kits, and Promonitor ELISA kits with each other. In the absence of sufficient clinical data for specific ELISAs we will assume equal assay performance and compare ELISAs on the basis of cost only.

If data permits, a linked evidence approach will be adopted to compare LISA-TRACKER ELISA kits, TNFα-Blocker ELISA kits, and Promonitor ELISA kits with standard care in which clinical outcomes for the intervention arm are taken from studies in which the assay procedure was not one of the intervention assays; this will involve an assessment of the comparability of LISA-TRACKER ELISA kits, TNFα-Blocker ELISA kits, or Promonitor ELISA kits performance with that of the alternative procedure.

The model will have a one-year time horizon in line with the previous HTA report[48] and other studies we have found during our initial scoping search (e.g., Velayos et al., 2013).[49]

For peer review only - http://bmjopen.bmj.com/site/about/guidelines.xhtml

It is anticipated that information from the clinical effectiveness analyses will help inform the probabilities for each of the clinical pathways. Sensitivity analyses will be conducted in areas of uncertainty.

5.2.2 Resource use and costs

Resource use and costs will be estimated in line with the DAP programme manual. Information on resource use and costs associated with the different patient pathways (e.g., comparing clinical pathways followed when LISA-TRACKER ELISA kits, TNFα-Blocker ELISA kits, or Promonitor ELISA kits are employed, versus standard care pathway etc.) will be collected from systematic reviews of the literature, discussions with individual manufacturers and hospitals and if need be, by eliciting expert clinical advice. Any remaining gaps for resource use parameters will be filled by assumptions made by the research team.

Unit costs data will be based on national data were possible. For the different LISA-TRACKER ELISA kits, TNFα-Blocker ELISA kits, and Promonitor ELISA kits, costs will be from published list prices from the NHS supply chain, from the NHS reference costs,[50] or discussions with individual manufacturers or hospitals. Costs of consultations with secondary care staff will be drawn from Unit Costs of Health and Social Care[51] and drug costs will be obtained from the British National Formulary.[34]

5.2.3 Health outcomes

Health outcomes and utility data will be derived from the literature review including the previous HTA report and other sources. If direct measurements of utility or choice-based multi-attribute utility scales (such as the EQ-5D or SF-6D) suitable for calculation of QALYs for the economic model are not reported, we may need to use one of the algorithms for mapping from a clinical measure (e.g. CDAI) to a measure of utility. If insufficient information is available for utilities it may have to be elicited from an expert clinical panel or by assumptions made by the research team.

5.2.4 Cost-effectiveness analysis

The results of the cost-effectiveness analysis will be presented as an incremental cost per QALY gained for LISA-TRACKER ELISA kits, TNFα-Blocker ELISA kits, and Promonitor ELISA kits compared with standard care. If the data allows us to compare LISA-TRACKER ELISA kits, TNFα-Blocker ELISA kits, and Promonitor ELISA kits with each other, then we will undertake a rank comparison and exclude any options which are dominated or extended dominated. It may be necessary, in the absence of suitable clinical outcome data, to rank ELISAs on the basis of cost only.

We will use both simple and probabilistic sensitivity analysis to explore the robustness of the results and to estimate the impact of uncertainty over model parameters. The simple sensitivity analysis will

For peer review only - http://bmjopen.bmj.com/site/about/guidelines.xhtml

be used to assess the robustness of the results to changes in deterministic parameters such as costs, and utilities. The results from the probabilistic sensitivity analysis will be presented as cost-effectiveness acceptability curves. Decisions regarding mutually exclusive alternatives will be reflected using cost-effectiveness planes and cost-effectiveness acceptability curves or frontiers.

If a longer time horizon is chosen (more than one year), both costs and outcomes will be discounted using the recommended 3.5% discount rate by HM Treasury.

6. Handling of information from manufacturers

All data submitted by the manufacturers/sponsors will only be considered if received by the External Assessment Group before 27 January 2015. Data arriving after this date will not be considered. Any data that meets the inclusion criteria stated will be extracted and quality assessed as stated in the methods section of this protocol.

Any ‘commercial in confidence’ data provided by manufacturers, and specified as such, will be highlighted in blue and underlined in the assessment report (followed by company name in parentheses). Any ‘academic in confidence’ data provided by manufacturers, and specified as such, will be highlighted in yellow and underlined in the assessment report. All confidential data used in the cost-effectiveness models will also be highlighted.

7. Competing interests of authors and advisors

None of the authors have any competing interests.

8. Timetable/milestones

Draft assessment protocol	**06/10/2014**
Final protocol	**28/10/2014**
Progress report	**27/01/2015**
Draft assessment report	**24/03/2015**
Final assessment report	**23/04/2015**

9. Team members’ contributions

Warwick Evidence is an External Assessment Group located within Warwick Medical School. Warwick Evidence brings together experts in clinical and cost effectiveness reviewing, medical statistics, health economics and modelling. The team planned for the work include:

Lead: Mrs Karoline Freeman

For peer review only - http://bmjopen.bmj.com/site/about/guidelines.xhtml

Title: Research Fellow
Address: Warwick Evidence, Populations, Evidence and Technologies, Division of Health Sciences, Warwick Medical School, University of Warwick, Coventry CV4 7AL
Tel: 02476 574026
Email: K.Freeman@warwick.ac.uk
Contribution: Protocol development, assessment for eligibility, quality assessment of trials, data extraction, data entry, and report writing

Name: Dr Martin Connock
Title: Senior Research Fellow
Address: Warwick Evidence, Populations, Evidence and Technologies, Division of Health Sciences, Warwick Medical School, University of Warwick, Coventry CV4 7AL
Tel: 02476 574940
Email: M.Connock@warwick.ac.uk
Contribution: Protocol development, assessment for eligibility, quality assessment of trials, data analysis, statistical modelling, and report writing

Name: Dr Hema Mistry
Title: Health economist
Address: Warwick Evidence, Populations, Evidence and Technologies, Division of Health Sciences, Warwick Medical School, University of Warwick, Coventry CV4 7AL
Tel: 02476 574490
Email: Hema.Mistry@warwick.ac.uk
Contribution: Protocol development, health economics modeller, data analysis, and report writing

Name: Dr Sian Taylor-Phillips
Title: Senior Research Fellow
Address: Warwick Evidence, Populations, Evidence and Technologies, Division of Health Sciences, Warwick Medical School, University of Warwick, Coventry CV4 7AL
Tel: 02476 575882
Email: S.Taylor-Phillips@warwick.ac.uk
Contribution: Protocol development, data analysis, and report writing

Name: Ms Rachel Court
Title: Information Specialist
Address: Warwick Evidence, Populations, Evidence and Technologies, Division of Health Sciences, Warwick Medical School, University of Warwick, Coventry CV4 7AL

For peer review only - http://bmjopen.bmj.com/site/about/guidelines.xhtml

Tel: 02476 522427
Email: R.A.Court@warwick.ac.uk
Contribution: Protocol development, develop search strategy and undertake the electronic literature searches

Name: Dr Alexander Tsertsvadze
Title: Senior Research Fellow
Address: Warwick Evidence, Populations, Evidence and Technologies, Division of Health Sciences, Warwick Medical School, University of Warwick, Coventry CV4 7AL
Tel: 02476 574505
Email: a_tsertsvadze@hotmail.com
Contribution: Assessment for eligibility, quality assessment of trials, data extraction, data analysis, and report writing

Name: Dr Jason Madan
Title: Assistant Professor in Health Economics
Address: Clinical Trials Unit, University of Warwick, Coventry CV4 7AL
Tel: 024761 51254
Email: j.j.madan@warwick.ac.uk
Contribution: Provide health economic modelling support, data analysis, and report writing

Name: Dr Ngianga-Bakwin Kandala
Title: Principal Research Fellow
Address: Warwick Evidence, Populations, Evidence and Technologies, Division of Health Sciences, Warwick Medical School, University of Warwick, Coventry CV4 7AL
Tel: 02476 575054
Email: N-B.Kandala@warwick.ac.uk
Contribution: Data analysis and statistical modelling

Name: Professor Aileen Clarke
Title: Director of Warwick Evidence
Address: Warwick Evidence, Populations, Evidence and Technologies, Division of Health Sciences, Warwick Medical School, University of Warwick, Coventry CV4 7AL
Tel: 02476 150189
Email: Aileen.Clarke@warwick.ac.uk
Contribution: Co-ordinate review process, protocol development, synthesis of findings and report writing

For peer review only - http://bmjopen.bmj.com/site/about/guidelines.xhtml

Name: Dr Paul Sutcliffe
Title: Associate Professor
Address: Warwick Evidence, Populations, Evidence and Technologies, Division of Health Sciences, Warwick Medical School, University of Warwick, Coventry CV4 7AL
Tel: 02476 574505
Email: p.a.sutcliffe@warwick.ac.uk
Contribution: Co-ordinate review process, protocol development, assessment for eligibility, quality assessment of trials, data extraction, data entry, data analysis, and report writing

9.1 Expert advisors

Name: Dr Ramesh P Arasaradnam
Title: Hon Assoc. Prof of Medicine and Consultant Gastroenterologist
Address: Clinical Sciences Research Institute, Clifford Bridge Road, Coventry CV2 2DX
Tel: 02476 966087
Email: r.arasaradnam@warwick.ac.uk
Contribution: Provide expert clinical advice on Crohn's and care pathways

Name: Dr Ahmed Naher
Title: Academic clinical fellow in clinical pharmacology and therapeutics
Address: Institute of Translational Medicine, University of Liverpool
Tel: 07949170357
Email: al.naher@gmail.com
Contribution: Provide expert advice on Crohn's and care pathways

For peer review only - http://bmjopen.bmj.com/site/about/guidelines.xhtml

10. References

1. Jerne NK. Towards a network theory of the immune system. *Annales d'immunologie*. 1974;**125c**(1-2):373-89.

2. Steenholdt C. Use of infliximab and anti-infliximab antibody measurements to evaluate and optimize efficacy and safety of infliximab maintenance therapy in Crohn's disease. *Danish medical journal*. 2013;**60**(4):B4616.

3. Allez M, Karmiris K, Louis E, Van Assche G, Ben-Horin S, Klein A, *et al*. Report of the ECCO pathogenesis workshop on anti-TNF therapy failures in inflammatory bowel diseases: definitions, frequency and pharmacological aspects. *Journal of Crohn's & colitis*. 2010;**4**(4):355-66.

4. Ben-Horin S, Chowers Y. Review article: loss of response to anti-TNF treatments in Crohn's disease. *Alimentary pharmacology & therapeutics*. 2011;**33**(9):987-95.

5. Cassinotti A, Travis S. Incidence and clinical significance of immunogenicity to infliximab in Crohn's disease: a critical systematic review. *Inflammatory bowel diseases*. 2009;**15**(8):1264-75.

6. Lee LY, Sanderson JD, Irving PM. Anti-infliximab antibodies in inflammatory bowel disease: prevalence, infusion reactions, immunosuppression and response, a meta-analysis. *European journal of gastroenterology & hepatology*. 2012;**24**(9):1078-85.

7. Chaparro M, Guerra I, Munoz-Linares P, Gisbert JP. Systematic review: antibodies and anti-TNF-alpha levels in inflammatory bowel disease. *Alimentary pharmacology & therapeutics*. 2012;**35**(9):971-86.

8. NHS choices. Crohn's disease. 2013 [cited 06/10/2014]; Available from: http://www.nhs.uk/Conditions/Crohns-disease/Pages/Introduction.aspx.

9. Jewell DP. Crohn's disease. *Medicine*. 2007;**35**(5):283-9.

10. Carter MJ, Lobo AJ, Travis SP. Guidelines for the management of inflammatory bowel disease in adults. *Gut*. 2004;**53 Suppl 5**:V1-16.

11. Hanauer SB, Sandborn W. Management of Crohn's disease in adults. *The American journal of gastroenterology*. 2001;**96**(3):635-43.

12. Jenkins HR. Inflammatory bowel disease. *Archives of disease in childhood*. 2001;**85**(5):435-7.

13. Silverberg MS, Satsangi J, Ahmad T, Arnott ID, Bernstein CN, Brant SR, *et al*. Toward an integrated clinical, molecular and serological classification of inflammatory bowel disease: report of a Working Party of the 2005 Montreal World Congress of Gastroenterology. *Canadian journal of gastroenterology = Journal canadien de gastroenterologie*. 2005;**19 Suppl A**:5a-36a.

14. Gasche C, Scholmerich J, Brynskov J, D'Haens G, Hanauer SB, Irvine EJ, *et al*. A simple classification of Crohn's disease: report of the Working Party for the World Congresses of Gastroenterology, Vienna 1998. *Inflammatory bowel diseases*. 2000;**6**(1):8-15.

For peer review only - http://bmjopen.bmj.com/site/about/guidelines.xhtml

15. Sostegni R, Daperno M, Scaglione N, Lavagna A, Rocca R, Pera A. Review article: Crohn's disease: monitoring disease activity. *Alimentary pharmacology & therapeutics*. 2003;**17 Suppl 2**:11-7.

16. Best WR, Becktel JM, Singleton JW, Kern F, Jr. Development of a Crohn's disease activity index. National Cooperative Crohn's Disease Study. *Gastroenterology*. 1976;**70**(3):439-44.

17. Yoshida EM. The Crohn's Disease Activity Index, its derivatives and the Inflammatory Bowel Disease Questionnaire: a review of instruments to assess Crohn's disease. *Canadian journal of gastroenterology = Journal canadien de gastroenterologie*. 1999;**13**(1):65-73.

18. Sandborn WJ, Feagan BG, Hanauer SB, Lochs H, Lofberg R, Modigliani R, *et al*. A review of activity indices and efficacy endpoints for clinical trials of medical therapy in adults with Crohn's disease. *Gastroenterology*. 2002;**122**(2):512-30.

19. Hyams J, Crandall W, Kugathasan S, Griffiths A, Olson A, Johanns J, *et al*. Induction and maintenance infliximab therapy for the treatment of moderate-to-severe Crohn's disease in children. *Gastroenterology*. 2007;**132**(3):863-73; quiz 1165-6.

20. Best WR. Predicting the Crohn's disease activity index from the Harvey-Bradshaw Index. *Inflammatory bowel diseases*. 2006;**12**(4):304-10.

21. Irvine EJ. Usual therapy improves perianal Crohn's disease as measured by a new disease activity index. McMaster IBD Study Group. *Journal of clinical gastroenterology*. 1995;**20**(1):27-32.

22. Irvine EJ, Feagan B, Rochon J, Archambault A, Fedorak RN, Groll A, *et al*. Quality of life: a valid and reliable measure of therapeutic efficacy in the treatment of inflammatory bowel disease. Canadian Crohn's Relapse Prevention Trial Study Group. *Gastroenterology*. 1994;**106**(2):287-96.

23. Mary JY, Modigliani R. Development and validation of an endoscopic index of the severity for Crohn's disease: a prospective multicentre study. Groupe d'Etudes Therapeutiques des Affections Inflammatoires du Tube Digestif (GETAID). *Gut*. 1989;**30**(7):983-9.

24. Schnitzler F, Fidder H, Ferrante M, Noman M, Arijs I, Van Assche G, *et al*. Long-term outcome of treatment with infliximab in 614 patients with Crohn's disease: results from a single-centre cohort. *Gut*. 2009;**58**(4):492-500.

25. Gisbert JP, Panes J. Loss of response and requirement of infliximab dose intensification in Crohn's disease: a review. *The American journal of gastroenterology*. 2009;**104**(3):760-7.

26. Baert F, Noman M, Vermeire S, Van Assche G, G DH, Carbonez A, *et al*. Influence of immunogenicity on the long-term efficacy of infliximab in Crohn's disease. *The New England journal of medicine*. 2003;**348**(7):601-8.

27. Hanauer SB, Feagan BG, Lichtenstein GR, Mayer LF, Schreiber S, Colombel JF, *et al*. Maintenance infliximab for Crohn's disease: the ACCENT I randomised trial. *Lancet*. 2002;**359**(9317):1541-9.

28. Maser EA, Villela R, Silverberg MS, Greenberg GR. Association of trough serum infliximab to clinical outcome after scheduled maintenance treatment for Crohn's disease. *Clinical*

For peer review only - http://bmjopen.bmj.com/site/about/guidelines.xhtml

gastroenterology and hepatology : the official clinical practice journal of the American Gastroenterological Association. 2006;**4**(10):1248-54.

29. Hanauer SB, Wagner CL, Bala M, Mayer L, Travers S, Diamond RH, *et al*. Incidence and importance of antibody responses to infliximab after maintenance or episodic treatment in Crohn's disease. *Clinical gastroenterology and hepatology : the official clinical practice journal of the American Gastroenterological Association*. 2004;**2**(7):542-53.

30. Lichtenstein GR, Hanauer SB, Sandborn WJ. Management of Crohn's disease in adults. *The American journal of gastroenterology*. 2009;**104**(2):465-83; quiz 4, 84.

31. Dignass A, Van Assche G, Lindsay JO, Lemann M, Soderholm J, Colombel JF, *et al*. The second European evidence-based Consensus on the diagnosis and management of Crohn's disease: Current management. *Journal of Crohn's & colitis*. 2010;**4**(1):28-62.

32. National Institute for Health and Care Excellence. Crohn's disease: Management in adults, children and young people. CG152. 2012 [cited 06/10/2014]; Available from: https://www.nice.org.uk/guidance/cg152.

33. BMJ Best Practice. Crohn's disease. 2014 [cited 06/10/2014]; Available from: http://bestpractice.bmj.com/best-practice/monograph/42/treatment/details.html.

34. British Medical Assocation and Royal Pharmaceutical Society of Great Britain. British National Formulary and British National Formulary for Children. [cited 06/10/2014]; Available from: http://www.bnf.org/bnf/index.htm.

35. National Institute for Health and Care Excellence. Infliximab (review) and adalimumab for the treatment of Crohn's disease. TA187. 2010 [cited 06/10/2014]; Available from: http://www.nice.org.uk/guidance/TA187.

36. Vande Casteele N, Gils A, Ballet V, Compernolle G, Peeters M, Van Steen K, *et al*. Randomised Controlled Trial of Drug Level Versus Clinically Based Dosing of Infliximab Maintenance Therapy in IBD: Final Results of the TAXIT Study (OP001). *United European Gastroenterology Journal*. 2013;**1**(1s):A1.

37. Scott FI, Lichtenstein GR. Therapeutic Drug Monitoring of Anti-TNF Therapy in Inflammatory Bowel Disease. *Curr Treat Options Gastroenterol*. 2014;**12**(1):59-75.

38. NHS Centre for Reviews and Dissemination. Undertaking systematic reviews of research on effectiveness: CRD guidelines for those carrying out or commissioning reviews. CRD Report 4. 1999.

39. National Institute for Health and Care Excellence. Diagnostics Assessment Programme manual. London, UK: National Institute for Health and Care Excellence; 2011 [cited 16/10/2014]. Available from: http://www.nice.org.uk/media/A0B/97/DAPManualFINAL.pdf.

40. Vande Casteele N, Buurman DJ, Sturkenboom MG, Kleibeuker JH, Vermeire S, Rispens T, *et al*. Detection of infliximab levels and anti-infliximab antibodies: a comparison of three different assays. *Alimentary pharmacology & therapeutics*. 2012;**36**(8):765-71.

For peer review only - http://bmjopen.bmj.com/site/about/guidelines.xhtml

41. Moher D, Liberati A, Tetzlaff J, Altman DG, The PRISMA Group. Preferred Reporting Items for Systematic Reviews and Meta-Analyses: The PRISMA Statement. *BMJ*. 2009;**339**:b2535.

42. Whiting PF, Rutjes AWS, Westwood ME, Mallett S, Deeks JJ, Reitsma JB, *et al*. QUADAS-2: A Revised Tool for the Quality Assessment of Diagnostic Accuracy Studies. *Annals of Internal Medicine*. 2011;**155**(8):529-36.

43. Downs SH, Black N. The feasibility of creating a checklist for the assessment of the methodological quality both of randomised and non-randomised studies of health care interventions. *Journal of epidemiology and community health*. 1998;**52**(6):377-84.

44. Higgins JP, Altman DG, Gotzsche PC, Juni P, Moher D, Oxman AD, *et al*. The Cochrane Collaboration's tool for assessing risk of bias in randomised trials. *Bmj*. 2011;**343**:d5928.

45. Merlin T, Lehman S, Hiller J, Ryan P. The "linked evidence approach" to assess medical tests: A critical analysis. *International Journal of Technology Assessment in Health Care*. 2013;**29**(03):343-50.

46. Husereau D, Drummond M, Petrou S, Carswell C, Moher D, Greenberg D, *et al*. Consolidated Health Economic Evaluation Reporting Standards (CHEERS) statement. *Int J Technol Assess Health Care*. 2013;**29**(2):117-22.

47. Philips Z, Ginnelly L, Sculpher M, Claxton K, Golder S, Riemsma R, *et al*. Review of guidelines for good practice in decision-analytic modelling in health technology assessment. *Health Technology Assessment*. 2004;**8**(36):1-158.

48. Dretzke J, Edlin R, Round J, Connock M, Hulme C, Czeczot J, *et al*. A systematic review and economic evaluation of the use of tumour necrosis factor-alpha (TNF-α) inhibitors, adalimumab and infliximab, for Crohn's disease. *Health Technology Assessment*. 2011;**15**(6):1-244.

49. Velayos FS, Kahn JG, Sandborn WJ, Feagan BG. A test-based strategy is more cost effective than empiric dose escalation for patients with Crohn's disease who lose responsiveness to infliximab. *Clinical Gastroenterology and Hepatology*. 2013;**11**(6):654-66.

50. Department of Health. NHS reference costs 2012 to 2013. 2013 [cited 18/03/2014]; Available from: https://www.gov.uk/government/publications/nhs-reference-costs-2012-to-2013.

51. Curtis L. Unit Costs of Health and Social Care 2013 [cited 18/03/2014]; Available from: http://www.pssru.ac.uk/project-pages/unit-costs/2013/.

52. European Medicines Agency. Remicade : EPAR - Product Information : Annex I - Summary of product characteristics. 2014 [cited 06/10/2014]; Available from: http://www.ema.europa.eu/docs/en_GB/document_library/EPAR_-_Product_Information/human/000240/WC500050888.pdf.

53. European Medicines Agency. Humira : EPAR - Product Information : Annex I - Summary of product characteristics. 2014 [cited 06/10/2014]; Available from: http://www.emea.eu.int/humandocs/PDFs/EPAR/Humira/H-481-PI-en.pdf.

For peer review only - http://bmjopen.bmj.com/site/about/guidelines.xhtml

Appendix 1. Licenced indications for Infliximab and Adalimumab in Crohn's disease

The licence indication for Crohn's disease detailed in the European Medicines Agency Summary of Product Characteristics (Remicade)[52] is as follows:

"Adult Crohn's disease: Remicade is indicated for:

- treatment of moderately to severely active Crohn's disease, in adult patients who have not responded despite a full and adequate course of therapy with a corticosteroid and/or an immunosuppressant; or who are intolerant to or have medical contraindications for such therapies;
- treatment of fistulising, active Crohn's disease, in adult patients who have not responded despite a full and adequate course of therapy with conventional treatment (including antibiotics, drainage and immunosuppressive therapy).

Paediatric Crohn's disease

Remicade is indicated for treatment of severe, active Crohn's disease, in children and adolescents aged 6 to 17 years, who have not responded to conventional therapy including a corticosteroid, an immunomodulator and primary nutrition therapy; or who are intolerant to or have contraindications for such therapies. Remicade has been studied only in combination with conventional immunosuppressive therapy.

Moderately to severely active Crohn's disease

5 mg/kg given as an intravenous infusion followed by an additional 5 mg/kg infusion 2 weeks after the first infusion. If a patient does not respond after 2 doses, no additional treatment with infliximab should be given. Available data do not support further infliximab treatment, in patients not responding within 6 weeks of the initial infusion.

In responding patients, the alternative strategies for continued treatment are:

- Maintenance: Additional infusions of 5 mg/kg at 6 weeks after the initial dose, followed by infusions every 8 weeks or
- Re-administration: Infusion of 5 mg/kg if signs and symptoms of the disease recur

Fistulising, active Crohn's disease

5 mg/kg given as an intravenous infusion followed by additional 5 mg/kg infusions at 2 and 6 weeks after the first infusion. If a patient does not respond after 3 doses, no additional treatment with infliximab should be given.

For peer review only - http://bmjopen.bmj.com/site/about/guidelines.xhtml

In responding patients, the alternative strategies for continued treatment are:

- Maintenance: Additional infusions of 5 mg/kg every 8 weeks or
- Re-administration: Infusion of 5 mg/kg if signs and symptoms of the disease recur followed by infusions of 5 mg/kg every 8 weeks.

Although comparative data are lacking, limited data in patients who initially responded to 5 mg/kg but who lost response indicate that some patients may regain response with dose escalation. Continued therapy should be carefully reconsidered in patients who show no evidence of therapeutic benefit after dose adjustment.

In Crohn's disease, experience with re-administration if signs and symptoms of disease recur is limited and comparative data on the benefit/risk of the alternative strategies for continued treatment are lacking.

Crohn's disease (6 to 17 years)
5 mg/kg given as an intravenous infusion followed by additional 5 mg/kg infusion doses at 2 and 6 weeks after the first infusion, then every 8 weeks thereafter. Available data do not support further infliximab treatment in children and adolescents not responding within the first 10 weeks of treatment.

Some patients may require a shorter dosing interval to maintain clinical benefit, while for others a longer dosing interval may be sufficient. Patients who have had their dose interval shortened to less than 8 weeks may be at greater risk for adverse reactions. Continued therapy with a shortened interval should be carefully considered in those patients who show no evidence of additional therapeutic benefit after a change in dosing interval."

The Adalimumbab licence indication for Crohn's disease detailed in the European Medicines Agency Summary of Product Characteristics (Humira)[53] is as follows:

Paediatric Crohn's Disease
Humira is indicated for the treatment of severe active Crohn's disease in paediatric patients (from 6 years of age) who have had an inadequate response to conventional therapy including primary nutrition therapy, a corticosteroid, and an immunomodulator, or who are intolerant to or have contraindications for such therapies.

Paediatric Crohn's disease patients < 40 kg:
The recommended Humira induction dose regimen for paediatric subjects with severe Crohn's disease is 40 mg at Week 0 followed by 20 mg at Week 2. In case there is a need for a more rapid response to

For peer review only - http://bmjopen.bmj.com/site/about/guidelines.xhtml

therapy, the regimen 80 mg at Week 0 (dose can be administered as two injections in one day), 40 mg at Week 2 can be used, with the awareness that the risk for adverse events may be higher with use of the higher induction dose.

After induction treatment, the recommended dose is 20 mg every other week via subcutaneous injection. Some subjects who experience insufficient response may benefit from an increase in dosing frequency to 20 mg Humira every week.

Paediatric Crohn's disease patients ≥ 40 kg:
The recommended Humira induction dose regimen for paediatric subjects with severe Crohn's disease is 80 mg at Week 0 followed by 40 mg at Week 2. In case there is a need for a more rapid response to therapy, the regimen 160 mg at Week 0 (dose can be administered as four injections in one day or as two injections per day for two consecutive days), 80 mg at Week 2 can be used, with the awareness that the risk for adverse events may be higher with use of the higher induction dose.

After induction treatment, the recommended dose is 40 mg every other week via subcutaneous injection. Some subjects who experience insufficient response may benefit from an increase in dosing frequency to 40 mg Humira every week.

Continued therapy should be carefully considered in a subject not responding by Week 12. A 40 mg pen and a 40 mg prefilled syringe are also available for patients to administer a full 40 mg dose. There is no relevant use of Humira in children aged less than 6 years in this indication.

For peer review only - http://bmjopen.bmj.com/site/about/guidelines.xhtml

Appendix 2. The CDAI Calculation of Crohn's Disease Activity Index (adapted from Best et al., 1976)[16]

Variable	Description	Scoring	Multiplier
No. of liquid stools	Sum of 7 days		x 2
Abdominal pain	Sum of 7 days' ratings	0=none<br>1=mild<br>2=moderate<br>3=severe	x 5
General well-being	Sum of 7 days' ratings	0=generally well<br>1=slightly under par<br>2=poor<br>3=very poor<br>4=terrible	x 7
Extraintestinal complications	Number of complications listed	Arthritis/arthralgia, iritis/uveitis, erythema nodosum, pyoderma gangrenosum, aphtous stomatitis, anal fissure/fistula/abscess, fever >37.8 °C	x 20
Anti-diarrhoeal drugs	Use in the previous 7 days	0=no<br>1=yes	x 30
Abdominal mass		0= no<br>2=questionable<br>5=definite	x 10
Haematocrit	Expected-observed Hct	Men: 47-observed<br>Women: 42-observed	x 6
Body weight	Ideal/observed ratio	(1-(ideal/observed)) x 100	x 1 (NOT< -10)

For peer review only - http://bmjopen.bmj.com/site/about/guidelines.xhtml

Appendix 3. Draft search strategy

Ovid MEDLINE(R) 1946 to October Week 2 2014, searched on 22/10/2014

1	adalimumab.mp.	3597
2	ADA.tw.	7105
3	infliximab.mp.	8842
4	IFX.tw.	326
5	((anti-TNF* or antiTNF* or TNF*) adj2 inhibitor*).mp.	2577
6	anti* tumo?r* necrosis* factor*.mp.	3007
7	Tumor Necrosis Factor-alpha/ and Antibodies, Monoclonal/	7682
8	anti* drug* antibod*.tw.	186
9	ADAb.tw.	19
10	1 or 2 or 3 or 4 or 5 or 6 or 7 or 8 or 9	24181
11	lisa* tracker*.mp.	1
12	(immundiagnostik* or immunodiagnostik* or immunediagnostik*).mp.	159
13	(proteomika* or promonitor*).mp.	13
14	exp Enzyme-Linked Immunosorbent Assay/	129174
15	enzyme* link* immunoassay*.mp.	2873
16	enzyme* link* immuno* assay*.mp.	158537
17	ELISA*.mp.	113426
18	11 or 12 or 13 or 14 or 15 or 16 or 17	205224
19	*Radioimmunoassay/	7091
20	(radioimmuno* or radio immuno* or radio-immuno*).mp.	101819
21	RIA.tw.	17353
22	reporter* gene* assay*.mp.	3663
23	RGA.tw.	336
24	semi* fluid* phase* enzyme* immuno*.mp.	0
25	EIA.tw.	8288
26	((homogenous* or homogeneous*) adj1 mobilit* shift* assay*).mp.	4
27	HMSA.tw.	62
28	(Biomonitor* or iLite).tw.	4102
29	(Matriks* Biotek* or Shikari*).mp.	2
30	(Prometheus* or Anser IFX or Anser ADA).mp.	258
31	19 or 20 or 21 or 22 or 23 or 24 or 25 or 26 or 27 or 28 or 29 or 30	124775
32	((monitor* or pharmacokinetic* or measur* or level* or concentration*) adj3 (adalimumab or ADA or infliximab or IFX or Anti-TNF* or Anti-Tumour Necrosis Factor*)).mp.	1087

For peer review only - http://bmjopen.bmj.com/site/about/guidelines.xhtml

33	Inflammatory Bowel Diseases/	14444
34	Crohn Disease/	31596
35	crohn*.tw.	32370
36	inflammator* bowel* disease*.tw.	26840
37	IBD.tw.	11936
38	33 or 34 or 35 or 36 or 37	58401
39	(((monitor* or pharmacokinetic* or measur* or level* or concentration*) adj3 (adalimumab or infliximab or Anti-TNF* or AntiTNF* or Anti-Tumour Necrosis Factor*)) and (correlat* or associat* or test performance)).mp.	218
40	10 and 18 and 38	93
41	10 and 31 and 38	19
42	32 and 38	157
43	39 or 40 or 41 or 42	367
44	Animals/ not Humans/	3983380
45	43 not 44	349

For peer review only

For peer review only - http://bmjopen.bmj.com/site/about/guidelines.xhtml

Appendix 4. Data extraction form for clinical effectiveness studies

Data extraction form anti-TNF drug monitoring

Name of first reviewer: *Name of second reviewer:*

Study details	
Study ID (Endnote ref)	
First author surname	
Year of publication	
Country	
Study design	
Publication (full/abstract)	
Study setting	
Number of centres (by arm)	
Duration of study	
Follow up period	
Funding	
**Aim of the study**	
**Inclusion/exclusion criteria for patients**	
Inclusion criteria:	
Exclusion criteria:	

Study flow (consort diagram)			
**Item**	**Anti-TNF monitoring arm**	**Clinical judgement arm**	**All**
N of Screened			
N of excluded (ineligible)			
N of enrolled/included (eligible)			
N of non-participants at study entry (those refused, etc…)			
N Study sample at baseline randomised (if applicable)			
Withdrawals			
Lost to follow up/drop outs (sample attrition)			

Participants (characteristics and numbers)			
**Item**	**Anti-TNF monitoring arm N (%)**	**Clinical judgement arm N (%)**	**All**
Total number of participants at baseline (% CD)			
N (%) followed up			
N (%) included in analysis			
Patient group (responders / secondary loss of response)			
Age Mean (SD/range)<br>Median (range) years			
Sex Women n (%)			
Diagnostic criteria for CD			
Children n (%)			
Crohn's Disease Activity Score (CDAI) Mean (SD)			
N (%) patients in remission			

For peer review only - http://bmjopen.bmj.com/site/about/guidelines.xhtml

N (%) patients with active CD			
CD classification (Vienna / Montreal)			
Disease duration (years)			
Smoking n (%)			
Previous surgery n (%)			
Concomitant treatment (specify) n (%)			
Treatment duration at anti-TNF failure (days)			
Line of therapy<br>1st<br>2nd<br>3rd			
Previous anti-TNF therapy n (%)			
CRP (mg/mL)			
Calprotectin (µg/g)			
**Treatment**			
**Item**	**Anti-TNF monitoring arm**	**Clinical judgement arm**	
Anti-TNF drug (name)			
Anti-TNF dose			
Duration of treatment			
**Intervention test assay (please specify):**<br>**Technical aspects of test assay:**			
Manufacturer			
Time of anti-TNF, antibody measurement			
Assay type			
Assay name			
Type of ELISA (bridging / capture)			
Anti-TNF alpha detection:			
Micro plate pre-coat			
Drug detection (free / total)			
Detection reagents (one-step / two-step)			
Assay range			
Limit of detection			
Reagents			
Antibody reagent specificity for antigen			
Structural class of immunoglobulin of antibody			
Anti-body detection:			
Micro plate pre-coat			
Anti-body detection (free / total)			
Incubation times			
Assay range			
Limit of detection			
Standards/calibrators			
**Outcomes reported**			
**Item**	**Anti-TNF**	**Clinical**	**All**

For peer review only - http://bmjopen.bmj.com/site/about/guidelines.xhtml

	monitoring arm	judgement arm	
Primary outcome(s)			
Secondary study outcomes			
Timing of assessments (including info on parallel or sequential)			
Time to test result			
Number of inconclusive results n (%)			
Frequency of dose adjustment n (%)			
Frequency of treatment switch n (%)			
Measure of disease activity (e.g., CDAI, others?)			
Rates of<br>a) response y/n<br>b) relapse y/n<br>c) remission y/n			
Describe definition of progression:			
Describe definition of remission:			
Duration of<br>a) response<br>b) relapse<br>c) remission			
Rates of hospitalisation n (%)			
Rates of surgical intervention n (%)			
Time to surgical intervention y/n			
Health related quality of life y/n			
Length of follow up reported y/n			
Proportion progressing to surgery n (%)			
Time to surgical intervention			
**Incidence of adverse effects of treatment:**			
**Item**	**Anti-TNF monitoring arm**	**Clinical judgement arm**	**P value**

**Dose monitoring**		
**Item (Please define if necessary)**	**Anti-TNF monitoring arm**	**Clinical judgement arm**
Time of anti-TNF/ antibody measurement		
Frequency of anti-TNF/ antibody measurement		
Assay type		
Assay name		
Threshold of infliximab / adalimumab (therapeutic / sub-therapeutic) (in µg/mL)		
Limit of quantification of anti-TNF antibodies (in U/mL [arbitrary unit/mL]) for Ab		

For peer review only - http://bmjopen.bmj.com/site/about/guidelines.xhtml

detectable / non-detectable		
Algorithm specified for management y/n (specify)		
Algorithm provided		
Number of patients outside therapeutic range		
Mean anti-TNF (mg/m^3/wk) (SD)		
Number of patients dose increased		
Number of patients dose reduced		
Other		
**Health related quality of life**		
**Item**	**Anti-TNF monitoring arm**	**Clinical judgement arm**
**Test comparison**		
**Tests**		
Intervention test		
Comparison test 1 (specify)		
Comparison test 2 (specify)		
Comparison test 3 (specify)		
Comparison test 1: test specifications (if ELISA use items for intervention assay test above)		
Comparison test 2: test specifications (if ELISA use items for intervention assay test above)		
Comparison test 3: test specifications (if ELISA use items for intervention assay test above)		
Details of any repeat measurements (to check reliability, performance across different laboratories)		
**Selection and storage of patients/plasma samples**		
Description of method of selection		
Description of method and duration of storage		
Number of clinical samples		
Number of calibrator samples (spiked) for anti-TNF		
Number of calibrator samples (spiked) for antibodies		
Number of blank (control) samples		
Total number of plasma samples		

For peer review only

For peer review only - http://bmjopen.bmj.com/site/about/guidelines.xhtml

Results of comparison			
Item	Intervention test vs test comparison 1	Intervention test vs test comparison 2	Intervention test vs test comparison 3
**Correlation of drug measurement:**			
Regression method			
Linearity test/cusum test?			
R^2 (95%CI)			
Slope (95%CI)			
Intercept (95%CI)			
**From Bland-Altman plot for drug measurement:**			
Percent bias (95%CI)			
Upper limit of agreement			
Lower limit of agreement			
Details of outliers			
Visually is there a pattern between the mean value and the difference? (If no pattern are statistics from Bland-Altman plot interpretable)			
N (%) samples outside limits of quantification, if yes specify decision for them			
N (%) false positives			
N (%) false negatives			
**Correlation of antibody measurement:**			
Regression method			
Linearity test/cusum test?			
R^2 (95%CI)			
Slope (95%CI)			
Intercept (95%CI)			
**From Bland-Altman plot for antibody measurement:**			
Percent bias (95%CI)			
Upper limit of agreement			
Lower limit of agreement			
Details of outliers			
Visually is there a pattern between the mean value and the difference? (If no pattern are statistics from Bland-Altman plot interpretable)			
N (%) samples outside limits of quantification, if yes specify decision for them			
N (%) false positives			
N (%) false negatives			
**Authors' conclusion**			
**Reviewer's conclusion**			

For peer review only - http://bmjopen.bmj.com/site/about/guidelines.xhtml

Appendix 5. Quality assessment forms

A – QUADAS-2[42] tool with index questions adapted to the review for studies comparing performance of different tests

Name of first reviewer: Name of second reviewer:

Phase 1: State the review question

Patients (setting, intended use of index test, presentation, prior testing):
Index test(s):
Reference standard:

Phase 2: Draw a flow diagram for the primary study

Phase 3: Risk of bias and applicability judgements

QUADAS-2 is structured so that four key domains are each rated in terms of the risk of bias and the concern regarding applicability to the review question (as stated in Phase 1). Each key domain has a set of signalling questions to help reach the judgements regarding bias and applicability.

Domain 1: Patient selection

**A. Risk of bias**	
Describe methods of patient selection:	
**Was a consecutive or random sample of patients enrolled?**	
**Did the study avoid inappropriate exclusions?**	
**Could the selection of patients have introduced bias?**<br><br>**Risk:**	
**B. Concerns regarding applicability**	
Describe included patients (prior testing, presentation, intended use of intervention test and setting):<br><br>Range of drug / antibody concentrations:	
**Is there concern that the included patients or range of drug / antibody concentrations do not match the review question?**<br><br>**Concern:**	

Domain 2: Index test(s)

**A. Risk of bias**	
Describe the intervention test and how it was conducted and interpreted:	
Were the number of failed results and measurement repeats reported?	
**Could the conduct or interpretation of the intervention test have introduced bias?**<br><br>**Risk:**	

For peer review only - http://bmjopen.bmj.com/site/about/guidelines.xhtml

**B. Concerns regarding applicability**<br><br>Describe the preparation and storage of the sample before the intervention test was applied:
**Is there concern that the intervention test, its conduct, or interpretation differ from the review question?**<br><br>**Concern:**

Domain 3: Reference standard (Comparison test)

**A. Risk of bias**	
Describe the comparison test and how it was conducted and interpreted:	
**Is the comparison test likely to correctly classify the target condition?**	
**Could the comparison test, its conduct, or its interpretation have introduced bias?**<br><br>**Risk:**	
**B. Concerns regarding applicability**	
**Is there concern that the target condition as defined by the comparison test does not match the review question?**<br><br>**Concern:**	

Domain 4: Flow and timing

**A. Risk of bias**	
Describe any patients who did not receive the intervention test and/or comparison test(s) or who were excluded from the Bland-Altman plot:<br><br>Describe the time interval and any interventions between intervention test and comparison test(s):	
**Was there an appropriate interval between intervention test and comparison test(s)?**	
**Were both intervention test and reference standard conducted on all samples?**	
**Did patients receive the same comparison test(s)?**	
**Were all patients included in the Bland-Altman plot?**	
**Could the patient flow have introduced bias?**<br><br>**Risk:**	

For peer review only

B – Cochrane Collaboration's tool for assessing risk of bias for a randomised controlled trial (adapted from Higgins et al., 2011[44])

First author surname and year of publication:
Name of first reviewer: ***Name of second reviewer:***

**Domain**	**Description**	**Review authors' judgement**
**Sequence generation**	Describe the method used to generate the allocation sequence in sufficient detail to allow an assessment of whether it should produce comparable groups	Was the allocation sequence adequately generated?
**Allocation concealment**	Describe the method used to conceal the allocation sequence in sufficient detail to determine whether intervention allocations could have been foreseen in advance of, or during, enrolment	Was allocation adequately concealed?
**Blinding of participants, personnel and outcome assessors** *Assessments should be made for each main outcome (or class of outcomes)*	Describe all measures used, if any, to blind study participants and personnel from knowledge of which intervention a participant received. Provide any information relating to whether the intended blinding was effective	Was knowledge of the allocated intervention adequately prevented during the study?
**Incomplete outcome data** *Assessments should be made for each main outcome (or class of outcomes)*	Describe the completeness of outcome data for each main outcome, including attrition and exclusions from the analysis. State whether attrition and exclusions were reported, the numbers in each intervention group (compared with total randomized participants), reasons for attrition/exclusions where reported, and any re-inclusions in analyses performed by the review authors	Were incomplete outcome data adequately addressed?
**Selective outcome reporting**	State how the possibility of selective outcome reporting was examined by the review authors, and what was found	Are reports of the study free of suggestion of selective outcome reporting?
**Other sources of bias**	State any important concerns about bias not addressed in the other domains in the tool. If particular questions/entries were pre-specified in the review's protocol, responses should be provided for each question/entry	Was the study apparently free of other problems that could put it at a high risk of bias?

For peer review only - http://bmjopen.bmj.com/site/about/guidelines.xhtml

Summary assessment of the risk of bias across domains (please highlight overall risk of bias rating)

**Risk of bias across key domains**	**Interpretation**	**Summary risk of bias**
Low risk of bias for all key domains	Plausible bias unlikely to seriously alter the results	Low risk of bias
Unclear risk of bias for one or more key domains	Plausible bias that raises some doubt about the results	Unclear risk of bias
High risk of bias for one or more key domains	Plausible bias that seriously weakens confidence in the results	High risk of bias

For peer review only

For peer review only - http://bmjopen.bmj.com/site/about/guidelines.xhtml

C – Downs and Black checklist[43] for non-randomised primary clinical studies

First author (year) study ID:

Name of first reviewer: *Name of second reviewer:*

**Reporting**	**Rating**
1. Is the hypothesis/aim/objective of the study clearly described? **(Yes/No)**	
2. Are the main outcomes to be measured clearly described in the Introduction or Methods section? **(Yes/No)** *If the main outcomes are first mentioned in the Results section, the question should be answered "No"*	
3. Are the characteristics of the patients included in the study clearly described? **(Yes/No)** *In cohort studies and trials, inclusion and/or exclusion criteria should be given. In case-control studies, a case-definition and the source for controls should be givenFsan*	
4. Are the interventions of interest clearly described? **(Yes/No)** *Treatments and placebo (where relevant) that are to be compared should be clearly described*	
5. Are the distributions of principal confounders in each group of subjects to be compared clearly described? **(Yes/Partially/No)** *A list of principal confounders is provided*	
6. Are the main findings of the study clearly described? **(Yes/No)** *Simple outcome data (including denominators and numerators) should be reported for all major findings so that the reader can check the major analyses and conclusions (This question does not cover statistical tests which are considered below)*	
7. Does the study provide estimates of the random variability in the data for the main outcomes? **(Yes/No)** *In non-normally distributed data the inter-quartile range of results should be reported. In normally distributed data the standard error, standard deviation or confidence intervals should be reported. If the distribution of the data is not described, it must be assumed that the estimates used were appropriate and the question should be answered "Yes"*	
8. Have all important adverse events that may be a consequence of the intervention been reported? **(Yes/No)** *This should be answered "Yes" if the study demonstrates that there was a comprehensive attempt to measure adverse events. (A list of possible adverse events is provided)*	
9. Have the characteristics of patients lost to follow-up been described? **(Yes/No)** *This should be answered "Yes" where there were no losses to follow-up or where losses to follow-up were so small that findings would be unaffected by their inclusion. This should be answered "No" where a study does not report the number of patients lost to follow-up*	
10. Have actual probability values been reported (e.g., 0.035 rather than <0.05) for the main outcomes except where the probability value is less than 0.001? **(Yes/No)**	
**External validity**	**Rating**
11. Were the subjects asked to participate in the study representative of the entire population from which they were recruited? **(Yes/No/Unable to determine)** *The study must identify the source population for patients and describe how the patients were selected. Patients would be representative if they comprised the entire source population, an unselected sample of*	

For peer review only

For peer review only - http://bmjopen.bmj.com/site/about/guidelines.xhtml

consecutive patients, or a random sample. Random sampling is only feasible where a list of all members of the relevant	
12. Were those subjects who were prepared to participate representative of the entire population from which they were recruited? **(Yes/No/Unable to determine)** *The proportion of those asked who agreed should be stated. Validation that the sample was representative would include demonstrating that the distribution of the main confounding factors was the same in the study sample and the source population*	
13. Were the staff, places, and facilities where the patients were treated, representative of the treatment the majority of patients receive? **(Yes/No/Unable to determine)** *For the question to be answered "Yes" the study should demonstrate that the intervention was representative of that in use in the source population. The question should be answered "No" if, for example, the intervention was undertaken in a specialist centre unrepresentative of the hospitals most of the source population would attend*	
**Internal validity – bias**	**Rating**
14. Was an attempt made to blind study subjects to the intervention they have received? **(Yes/No/Unable to determine)** *For studies where the patients would have no way of knowing which intervention they received, this should be answered "Yes"*	
15. Was an attempt made to blind those measuring the main outcomes of the intervention? **(Yes/No/Unable to determine)**	
16. If any of the results of the study were based on "data dredging", was this made clear? **(Yes/No/Unable to determine)** *Any analyses that had not been planned at the outset of the study should be clearly indicated. If no retrospective unplanned subgroup analyses were reported, then answer "Yes"*	
17. In trials and cohort studies, do the analyses adjust for different lengths of follow-up of patients, or in case-control studies, is the time period between the intervention and outcome the same for cases and controls? **(Yes/No/Unable to determine)** *Where follow-up was the same for all study patients the answer should "Yes". If different lengths of follow-up were adjusted for by, for example, survival analysis the answer should be "Yes". Studies where differences in follow-up are ignored should be answered "No"*	
18. Were the statistical tests used to assess the main outcomes appropriate? **(Yes/No/Unable to determine)** *The statistical techniques used must be appropriate to the data. For example nonparametric methods should be used for small sample sizes. Where little statistical analysis has been undertaken but where there is no evidence of bias, the question should be answered "Yes". If the distribution of the data (normal or not) is not described it must be assumed that the estimates used were appropriate and the question should be answered "Yes"*	
19. Was compliance with the intervention/s reliable? **(Yes/No/Unable to determine)** *Where there was non-compliance with the allocated treatment or where there was contamination of one group, the question should be answered "No". For studies where the effect of any misclassification was likely to bias any association to the null, the question should be answered "Yes"*	

For peer review only - http://bmjopen.bmj.com/site/about/guidelines.xhtml

20. Were the main outcome measures used accurate valid and reliable? **(Yes/No/Unable to determine)** *For studies where the outcome measures are clearly described, the question should be answered "Yes". For studies which refer to other work or that demonstrates the outcome measures are accurate, the question should be answered as "Yes"*	
**Internal validity - confounding (selection bias)**	**Rating**
21. Were the patients in different intervention groups (trials and cohort studies) or were the cases and controls (case-control studies) recruited from the same population? **(Yes/No/Unable to determine)** *For example, patients for all comparison groups should be selected from the same hospital. The question should be answered "Unable to determine" for cohort and case-control studies where there is no information concerning the source of patients included in the study*	
22. Were study subjects in different intervention groups (trials and cohort studies) or were the cases and controls (case-control studies) recruited over the same period of time? **(Yes/No/Unable to determine)** *For a study which does not specify the time period over which patients were recruited, the question should be answered as "Unable to determine"*	
23. Were the subjects randomised to intervention groups? **(Yes/No/Unable to determine)** *Studies which state that subjects were randomised should be answered "Yes" except where method of randomisation would not ensure random allocation. For example alternate allocation would score "No" because it is predictable*	
24. Was the randomised intervention assignment concealed from both patients and health care staff until recruitment was complete and irrevocable? **(Yes/No/Unable to determine)** *All non-randomised studies should be answered "No". If assignment was concealed from patients but not from staff, it should be answered "No"*	
25. Was there adequate adjustment for confounding in the analyses from which the main findings were drawn? **(Yes/No/Unable to determine)** *This question should be answered "No" for trials if: the main conclusions of the study were based on analyses of treatment rather than intention to treat; the distribution of known confounders in the different treatment groups was not described; or the distribution of known confounders differed between the treatment groups but was not taken into account in the analyses. In nonrandomised studies if the effect of the main confounders was not investigated or confounding was demonstrated but no adjustment was made in the final analyses the question should be answered as "No"*	
26. Were losses of patients to follow-up taken into account? **(Yes/No/Unable to determine)** *If the numbers of patients lost to follow-up are not reported, the question should be answered as "Unable to determine". If the proportion lost to follow-up was too small to affect the main findings, the question should be answered "Yes"*	
**Power**	**Rating**
27. Did the study have sufficient power to detect a clinically important effect where the probability value for a difference being due to chance is less than 5%? **(Yes/No/Unable to determine)***	

For peer review only

For peer review only - http://bmjopen.bmj.com/site/about/guidelines.xhtml

D – Critical appraisal of the economic evaluation studies using the CHEERS checklist (adapted from Husereau et al, 2013[46])

Title and abstract				
1 Title: Identify the study as an economic evaluation, or use more specific terms such as ``cost-effectiveness analysis``, and describe the interventions compared.				
2 Abstract: Provide a structured summary of objectives, methods including study design and inputs, results including base case and uncertainty analyses, and conclusions.				
Introduction				
3 Background & objectives: Provide an explicit statement of the broader context for the study. Present the study question and its relevance for health policy or practice decisions.				
Methods				
4 Target Population and Subgroups: Describe characteristics of the base case population and subgroups analysed including why they were chosen.				
5 Setting and Location: State relevant aspects of the system(s) in which the decision(s) need(s) to be made.				
6 Study perspective: Describe the perspective of the study and relate this to the costs being evaluated.				
7 Comparators: Describe the interventions or strategies being compared and state why they were chosen.				
8 Time Horizon: State the time horizon(s) over which costs and consequences are being evaluated and say why appropriate.				
9 Discount Rate: Report the choice of discount rate(s) used for costs and outcomes and say why appropriate.				

For peer review only - http://bmjopen.bmj.com/site/about/guidelines.xhtml

10 Choice of Health Outcomes: Describe what outcomes were used as the measure(s) of benefit in the evaluation and their relevance for the type of analysis performed.				
11a Measurement of Effectiveness - Single Study-Based Estimates: Describe fully the design features of the single effectiveness study and why the single study was a sufficient source of clinical effectiveness data.				
11b Measurement of Effectiveness - Synthesis-based Estimates: Describe fully the methods used for identification of included studies and clinical effectiveness data synthesis of clinical effectiveness data.				
12 Measurement and Valuation of Preference-based Outcomes: If applicable, describe the population and methods used to elicit preferences for health outcomes.				
13a Estimating Resources and Costs - Single Study-based Economic evaluation: Describe approaches used to estimate resource use associated with the alternative interventions. Describe primary or secondary research methods for valuing each resource item in terms of its unit cost. Describe any adjustments made to approximate to opportunity costs.				
13b Estimating Resources and Costs - Model-based Economic Evaluation: Describe approaches and data sources used to estimate resource use associated with model health states. Describe primary or secondary research methods for valuing each resource item in terms of its unit cost. Describe any adjustments made to approximate to opportunity costs.				
14 Currency, Price Date and Conversion: Report the dates of the estimated resource quantities				

For peer review only

For peer review only - http://bmjopen.bmj.com/site/about/guidelines.xhtml

and unit costs. Describe methods for adjusting estimated unit costs to the year of reported costs if necessary. Describe methods for converting costs into a common currency base and the exchange rate.				
15 Choice of Model: Describe and give reasons for the specific type of decision-analytic model used. Providing a figure to show model structure is strongly recommended.				
16 Assumptions: Describe all structural or other assumptions underpinning the decision-analytic model.				
17 Analytic Methods: Describe all analytic methods supporting the evaluation. This could include methods for dealing with skewed, missing or censored data, extrapolation methods, methods for pooling data, approaches to validate a model, and methods for handling population heterogeneity and uncertainty.				
Results				
18 Study parameters: Report the values, ranges, references, and if used, probability distributions for all parameters. Report reasons or sources for distributions used to represent uncertainty where appropriate. We strongly recommend the use of a table to show the input values.				
19. Incremental costs and outcomes: For each intervention, report mean values for the main categories of estimated costs and outcomes of interest, as well as mean differences between the comparator groups. If applicable, report incremental cost-effectiveness ratios.				
20a Characterizing Uncertainty - Single study-based economic evaluation: Describe the effects of sampling uncertainty for the estimated incremental cost and incremental effectiveness,				

For peer review only

For peer review only - http://bmjopen.bmj.com/site/about/guidelines.xhtml

parameters together with the impact of methodological assumptions.				
20b Characterizing Uncertainty - Model-based economic evaluation: Describe the effects on the results of uncertainty for all input parameters, and uncertainty related to the structure of the model and assumptions.				
21 Characterizing Heterogeneity: If applicable, report differences in costs, outcomes or in cost-effectiveness that can be explained by variations between subgroups of patients with different baseline characteristics or other observed variability in effects that are not reducible by more information.				
Discussion				
22 Study Findings, Limitations, Generalizability, and Current Knowledge: Summarize key study findings and describe how they support the conclusions reached. Discuss limitations and the generalizability of the findings and how the findings fit with current knowledge.				
Other				
23 Source of Funding: Describe how the study was funded and the role of the funder in the identification, design, conduct and reporting of the analysis. Describe other non-monetary sources of support.				
24 Conflicts of Interest: Describe any potential for conflict of interest among study contributors in accordance with journal policy. In the absence of a journal policy, we recommend authors comply with International Committee of Medical Journal Editors' recommendations.				

*Key: Y = yes, No = no, N/A = not applicable and * = partially completed*

For peer review only

PRISMA 2009 Checklist

Section/topic	#	Checklist item	Reported on page #
**TITLE**			
Title	1	Identify the report as a systematic review, meta-analysis, or both.	1
**ABSTRACT**			
Structured summary	2	Provide a structured summary including, as applicable: background; objectives; data sources; study eligibility criteria, participants, and interventions; study appraisal and synthesis methods; results; limitations; conclusions and implications of key findings; systematic review registration number.	2
**INTRODUCTION**			
Rationale	3	Describe the rationale for the review in the context of what is already known.	4
Objectives	4	Provide an explicit statement of questions being addressed with reference to participants, interventions, comparisons, outcomes, and study design (PICOS).	5
**METHODS**			
Protocol and registration	5	Indicate if a review protocol exists, if and where it can be accessed (e.g., Web address), and, if available, provide registration information including registration number.	6
Eligibility criteria	6	Specify study characteristics (e.g., PICOS, length of follow-up) and report characteristics (e.g., years considered, language, publication status) used as criteria for eligibility, giving rationale.	5
Information sources	7	Describe all information sources (e.g., databases with dates of coverage, contact with study authors to identify additional studies) in the search and date last searched.	5
Search	8	Present full electronic search strategy for at least one database, including any limits used, such that it could be repeated.	Supplementary material
Study selection	9	State the process for selecting studies (i.e., screening, eligibility, included in systematic review, and, if applicable, included in the meta-analysis).	5
Data collection process	10	Describe method of data extraction from reports (e.g., piloted forms, independently, in duplicate) and any processes for obtaining and confirming data from investigators.	5
Data items	11	List and define all variables for which data were sought (e.g., PICOS, funding sources) and any assumptions and simplifications made.	Supplementary material
Risk of bias in individual studies	12	Describe methods used for assessing risk of bias of individual studies (including specification of whether this was done at the study or outcome level), and how this information is to be used in any data synthesis.	5
Summary measures	13	State the principal summary measures (e.g., risk ratio, difference in means).	6
Synthesis of results	14	Describe the methods of handling data and combining results of studies, if done, including measures of consistency (e.g., I^2) for each meta-analysis.	5,6

For peer review only

PRISMA 2009 Checklist

Section/topic	#	Checklist item	Reported on page #
Risk of bias across studies	15	Specify any assessment of risk of bias that may affect the cumulative evidence (e.g., publication bias, selective reporting within studies).	6
Additional analyses	16	Describe methods of additional analyses (e.g., sensitivity or subgroup analyses, meta-regression), if done, indicating which were pre-specified.	6
**RESULTS**			
Study selection	17	Give numbers of studies screened, assessed for eligibility, and included in the review, with reasons for exclusions at each stage, ideally with a flow diagram.	Fig1
Study characteristics	18	For each study, present characteristics for which data were extracted (e.g., study size, PICOS, follow-up period) and provide the citations.	Table 1 and supplementary material
Risk of bias within studies	19	Present data on risk of bias of each study and, if available, any outcome level assessment (see item 12).	Supplementary material
Results of individual studies	20	For all outcomes considered (benefits or harms), present, for each study: (a) simple summary data for each intervention group (b) effect estimates and confidence intervals, ideally with a forest plot.	Figures 2-6
Synthesis of results	21	Present results of each meta-analysis done, including confidence intervals and measures of consistency.	Fig 5-6
Risk of bias across studies	22	Present results of any assessment of risk of bias across studies (see Item 15).	Supplementary material
Additional analysis	23	Give results of additional analyses, if done (e.g., sensitivity or subgroup analyses, meta-regression [see Item 16]).	
**DISCUSSION**			
Summary of evidence	24	Summarize the main findings including the strength of evidence for each main outcome; consider their relevance to key groups (e.g., healthcare providers, users, and policy makers).	9
Limitations	25	Discuss limitations at study and outcome level (e.g., risk of bias), and at review-level (e.g., incomplete retrieval of identified research, reporting bias).	9
Conclusions	26	Provide a general interpretation of the results in the context of other evidence, and implications for future research.	9
**FUNDING**			
Funding	27	Describe sources of funding for the systematic review and other support (e.g., supply of data); role of funders for the systematic review.	online

For peer review only

For peer review only - http://bmjopen.bmj.com/site/about/guidelines.xhtml

PRISMA 2009 Checklist

From: Moher D, Liberati A, Tetzlaff J, Altman DG, The PRISMA Group (2009). Preferred Reporting Items for Systematic Reviews and Meta-Analyses: The PRISMA Statement. PLoS Med 6(6): e1000097. doi:10.1371/journal.pmed1000097

For more information, visit: **www.prisma-statement.org**.

For peer review only

For peer review only - http://bmjopen.bmj.com/site/about/guidelines.xhtml
